# Drought stress triggers proteomic changes involving lignin, flavonoids and fatty acids in tea plants

**DOI:** 10.1038/s41598-020-72596-1

**Published:** 2020-09-23

**Authors:** Honglian Gu, Yu Wang, Hui Xie, Chen Qiu, Shuning Zhang, Jun Xiao, Hongyan Li, Liang Chen, Xinghui Li, Zhaotang Ding

**Affiliations:** 1 Tea Research Institute, Qingdao Agricultural University, Qingdao, 266109 Shandong China; 2 School of Biological Science and Winery Engineering, Taishan University, Taian, 271000 Shandong China; 3 Haiyang Fruit Technology Promotion Station, Yantai, 265100 Shandong China; 4 Tea Research Institute, Chinese Academy of Agricultural Sciences, Hangzhou, 310008 Zhejiang China; 5 Tea Research Institute, Nanjing Agricultural University, Nanjing, 210095 Jiangsu China

**Keywords:** Drought, Secondary metabolism

## Abstract

Drought stress triggers a series of physiological and biochemical changes in tea plants. It is well known that flavonoids, lignin and long-chain fatty acids play important roles in drought resistance. However, changes in proteins related to these three metabolic pathways in tea plants under drought stress have not been reported. We analysed the proteomic profiles of tea plants by tandem mass tag and liquid chromatography-tandem mass spectrometry. A total of 4789 proteins were identified, of which 11 and 100 showed up- and downregulation, respectively. The proteins related to the biosynthesis of lignin, flavonoids and long-chain fatty acids, including phenylalanine ammonia lyase, cinnamoyl-CoA reductase, peroxidase, chalcone synthase, flavanone 3-hydroxylase, flavonol synthase, acetyl-CoA carboxylase 1,3-ketoacyl-CoA synthase 6 and 3-ketoacyl-CoA reductase 1, were downregulated. However, the contents of soluble proteins, malondialdehyde, total phenols, lignin and flavonoids in the tea plants increased. These results showed that tea plants might improve drought resistance by inhibiting the accumulation of synthases related to lignin, flavonoids and long-chain fatty acids. The proteomic spectrum of tea plants provides a scientific basis for studying the pathways related to lignin, flavonoid and long-chain fatty acid metabolism in response to drought stress.

## Introduction

Tea plants, which require relatively humid environment, are often confronted with drought stress throughout their lifecycle. Drought stress is one of the most important environmental stresses that adversely affects the growth and quality of tea plants. It has been reported that drought stress can reduce tea production by 14–33% and can increase tea plant mortality by 6–19%^1^. Tea plants undergo a series of complex morphological, physiological and molecular changes to resist drought stress. Previous studies have revealed several key features of tea plants under water deficit stress, including small leaves, well-developed root systems, relatively thick cuticles and palisade tissue. Moreover, their stomata become closed, there is a loss of cell wall semipermeability, respiration and photosynthesis decrease, proteolysis is accelerated, and carbohydrate synthesis is reduced. Free radical content, antioxidative systems, and osmo-protectant contents were enhanced. In addition, the contents of some characteristic tea plant components, such as the contents of caffeine, polyphenols, and theanine, which are components composing tea aroma, and the corresponding synthetic genes, together with the expression of many related genes, are also affected by drought stress^2–8^. Moreover, other plant species also show a series of complex changes related to oxidative stress and antioxidant defence to resist drought stress^9–11^. Many studies have shown that flavonoids, lignin and long-chain fatty acids (LCFAs) play important roles in drought resistance^12–14^. Nevertheless, the biosynthesis pathways of these components in response to drought stress are poorly understand.

With the improvement in proteomics technology, the study of stress proteins involved in key pathways of tea plants under drought stress has become possible. Previous proteomic analyses showed that many photosynthetic proteins were significantly downregulated in tea plants under drought stress^15^. Previous proteomic analysis also showed that 23 proteins involved in redox status, metabolism and the defence response were upregulated in tea plant seeds under desiccation^16^. However, to date, little research has focused on proteins related to the biosynthesis of lignin, flavonoids and fatty acids in tea plants in response to drought stress. Therefore, we used tandem mass tag (TMT) and liquid chromatography–mass spectrometry (LC–MS) to study the global profile of proteomes of tea plants under drought stress. We then used a bioinformatics method to analyse the Gene Ontology (GO) terms, Kyoto Encyclopedia of Genes and Genomes (KEGG) pathways and protein–protein interactions (PPIs). These results provide insights into the complex molecular mechanisms associated with lignin, flavonoid and fatty acid biosynthesis in tea plants under drought stress and provide an important basis for improving the quality of tea plants under drought conditions.

## Materials and methods

### Plant materials, stress treatments and physiological determinations

Two-year-old *Camellia sinensis* (L.) O. Kuntze ‘Zhongcha 108’ plants were cultivated under conditions of 12 h light (25 °C)/12 h dark (20 °C) in a growth chamber with 1800 µE m^−2^ s^−1^ light intensity and 75% humidity for 2 weeks. Drought stress was imposed by withholding watering for 96 h (DT). Moreover, well-watered plants (CK) were also included. Finally, the third and/or fourth mature leaf was taken from the terminal bud for further experiments. The samples were frozen in liquid nitrogen and then stored at − 80 °C for physiological and proteomic analyses. Three biological replicates were included.

According to the manufacturer’s instructions (Suzhou Comin Biotechnology Co., Ltd., China), the contents of Cpr, malondialdehyde (MDA), total phenols (TP), flavonoids, and lignin and the activity of phenylalanine ammonia lyase (PAL) were determined on a microplate reader using a BCA protein content kit (serial number: BCAP-1-W), an MDA kit (serial number: MDA-1-Y), a TP kit (serial number: TP-1-G), a plant flavonoid kit (serial number: LHT-1-G), a lignin content kit (serial number: MZS-1-G), and a PAL kit (serial number: PAL-1-Y), respectively. These physiological measurements were determined according to previously reported methods, with slight modifications.

### Protein extraction and trypsin digestion

The protein extraction and digestion of the samples were performed according to previous methods^17,18^. In brief, four volumes of lysis buffer were added to the samples, followed by sonication and centrifugation. The lysis buffer consisted of 8 M urea, 1% Triton-100, 10 mM dithiothreitol, 1% protease inhibitor cocktail, 3 μM TSA, 50 mM NAM inhibitor and 2 mM EDTA. Finally, the precipitate was reconstituted with 8 M urea, and the protein concentration was measured by a BCA kit. For digestion, the protein solution was reduced with 5 mM dithiothreitol at 56 °C for 30 min and then alkylated with 11 mM iodoacetamide at room temperature for 15 min in darkness.

### TMT labelling and HPLC fractionation

The peptides were desalted through a Strata X C18 SPE column (Phenomenex) and freeze dried under vacuum. The samples were then reconstituted in 0.5 M TEAB and processed according to the TMT kit instructions. The specific procedure was performed in accordance with the methods of previous research^18^.

For HPLC fractionation, the peptides were fractionated by high-pH reversed-phase HPLC using an Agilent 300 Extend C18 column. The specifications and dimensions of the C18 column were as follows: 5 μm and 4.6 × 250 mm. The operation was as follows: the peptide gradient involved 8–32% acetonitrile (pH 9) for 60 min, divided into 60 fractions, after which the peptides were combined into 18 fractions.

### LC–MS/MS analysis

The tryptic peptides were dissolved in 0.1% formic acid (solvent A) and separated using an EASY-nLC 1000 system. The gradient elution procedure was as follows: a linear gradient involving an increase from 7 to 25% of solvent B (0.1% formic acid in 98% acetonitrile) for 26 min, an increase from 25 to 36% of solvent B for 8 min, an increase to 80% of solvent B for 3 min, after which it was for 3 min. The flow rate was constant at 700 nL/min.

The peptides were subjected to an NSI source followed by tandem mass spectrometry (MS/MS) on an OrbitrapFusion™ instrument (Thermo). The electrospray voltage applied was 2.0 kV. Both the peptide precursor ion and its secondary fragments were detected and analysed using a high-resolution Orbitrap device. The primary mass spectrometer scan range was 350–1550 *m*/*z*, and intact peptides were detected in the Orbitrap at a resolution of 60,000. The fragments were detected in the Orbitrap at a resolution of 15,000. A data-dependent procedure alternated between one MS scan followed by 20 MS/MS scans, with a 15.0 s dynamic exclusion. Automatic gain control was set to 5E4. The mass spectrometry proteomic data are available via ProteomeXchange under identifier PXD011688.

### PRM analyses

PRM mass spectrometric analysis was performed using a Q Exactive™ Plus tandem MS/MS instrument (Thermo). The LC parameters, electrospray voltage, scan range, and Orbitrap resolution were the same as those of the TMT methods. The AGC was set at 3E6 for full MS and 1E5 for MS/MS. The maximum IT was set to 50 ms for full MS and ‘auto’ for MS/MS. The isolation window for MS/MS was set at 1.6 *m*/*z*. The enzyme was set as trypsin [KR/P], and the max missed cleavage was set as 0. The peptide length was set to 7–25. The product ions were set from ion 3 to the last ion, and the ion match tolerance was set to 0.02 Da.

### Database search and bioinformatic analysis

The MS/MS data were processed using the MaxQuant search engine (v.1.5.2.8). The tandem mass spectra were searched against the *Camellia sinensis* genome database (https://www.plantkingdomgdb.com/tea_tree/) concatenated with the reverse decoy database. Trypsin/P was specified as a cleavage enzyme allowing up to 1 missing cleavage. The minimum length of the peptide was set to 7 amino acid residues, and the maximum number of decoration settings was 5. The mass tolerance for precursor ions was set to 20 ppm in the first search range, 5 ppm in the main search and 0.02 Da for the fragment ions. The variable modification was set to ‘oxidation of methionine, acetylation of the N-terminus of the protein, Iodo TMT-6plex var’. The false discovery rate of protein identification and peptide spectrum match identification was set to 1%.

GO annotation and enrichment analyses were performed by the UniProt-GOA database (www.http://www.ebi.ac.uk/GOA/), and the KEGG database was adopted for the enrichment of pathways by the DAVID functional annotation tool against the background of tea plant. A PPI network analysis was performed via the STRING database (https://string-db.org/).

## Results

### Identification and quantification of proteins in tea plant

**Figure 1:**
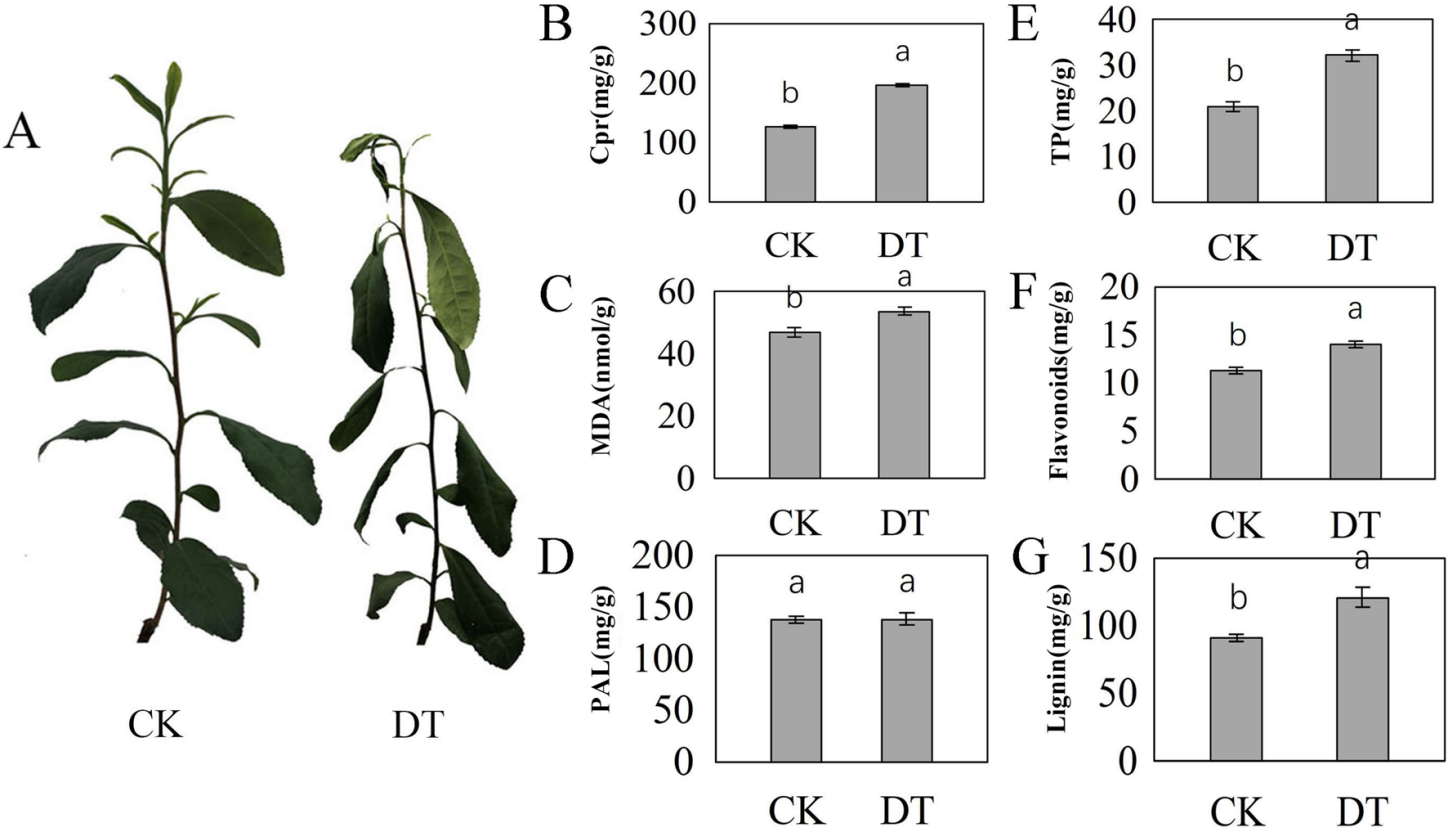
Morphological and physiological analyses of tea plants under drought stress. (**A**) The phenotypes of CK and DT, (**B**) the content of Cpr, (**C**) the content of MDA, (**D**) the activity of PAL, (**E**) the content of TP, (**F**) the content of flavonoids, and (**G**) the content of lignin were determined, with standard error bars from three replicates shown. The different letters within each column indicate significant differences between treatments at the *p* < 0.05 level.

To analyse the physiological and biochemical responses to drought, we analysed the contents of soluble proteins (Cpr), MDA, TP, flavonoids, and lignin and the activity of PAL in tea plants under drought stress from 0 to 96 h (Fig. 1) (Table 1). In turn, the tea plants became wrinkled and shrivelled during drought stress, which was especially severe at 96 h (Fig. 1A). The contents of Cpr and MDA increased under DT (Fig. 1B, C). The activity of PAL in the tea plants increased in response to drought stress (Fig. 1D). Accordingly, the contents of polyphenols, flavonoids and lignin increased under DT (Fig. 1E–G). These results showed that drought stress caused damage to tea plants and induced the synthesis of flavonoids and lignin.

**Table 1:** Statistical analysis of the physiological parameters of tea plants.

Sample	Cpr	MDA	PAL	TP	Flavonoids	Lignin
CK	126.25 ± 2.16b	46.85 ± 1.85b	137.06 ± 4.28a	21.01 ± 1.29b	11.20 ± 0.42b	91.24 ± 3.17b
DT	196.83 ± 3.92a	53.78 ± 1.31a	138.17 ± 7.51a	32.23 ± 1.66a	14.03 ± 0.47a	120.83 ± 8.55a

The mean values ± standard deviations (n = 6). The values with the same letter are not significantly different (*p* < 0.05).

*CK* control experiment, *DT* drought stress.

To analyse the changes in protein accumulation in tea plants under drought stress, we performed an analysis of the global proteome using TMT isobaric labelling technology. In total, 51,213 unique spectra were generated, which matched to 23,854 unique peptides (Supplementary Table S1). Correspondingly, 4789 proteins were identified, and 4242 proteins were quantified (Supplementary Table S2), of which 11 proteins were upregulated and 100 proteins downregulated, with high repeatability (FC > 1.5 and *p* < 0.05).

### Functional classification and enrichment of differentially expressed proteins (DEPs)

**Figure 2:**
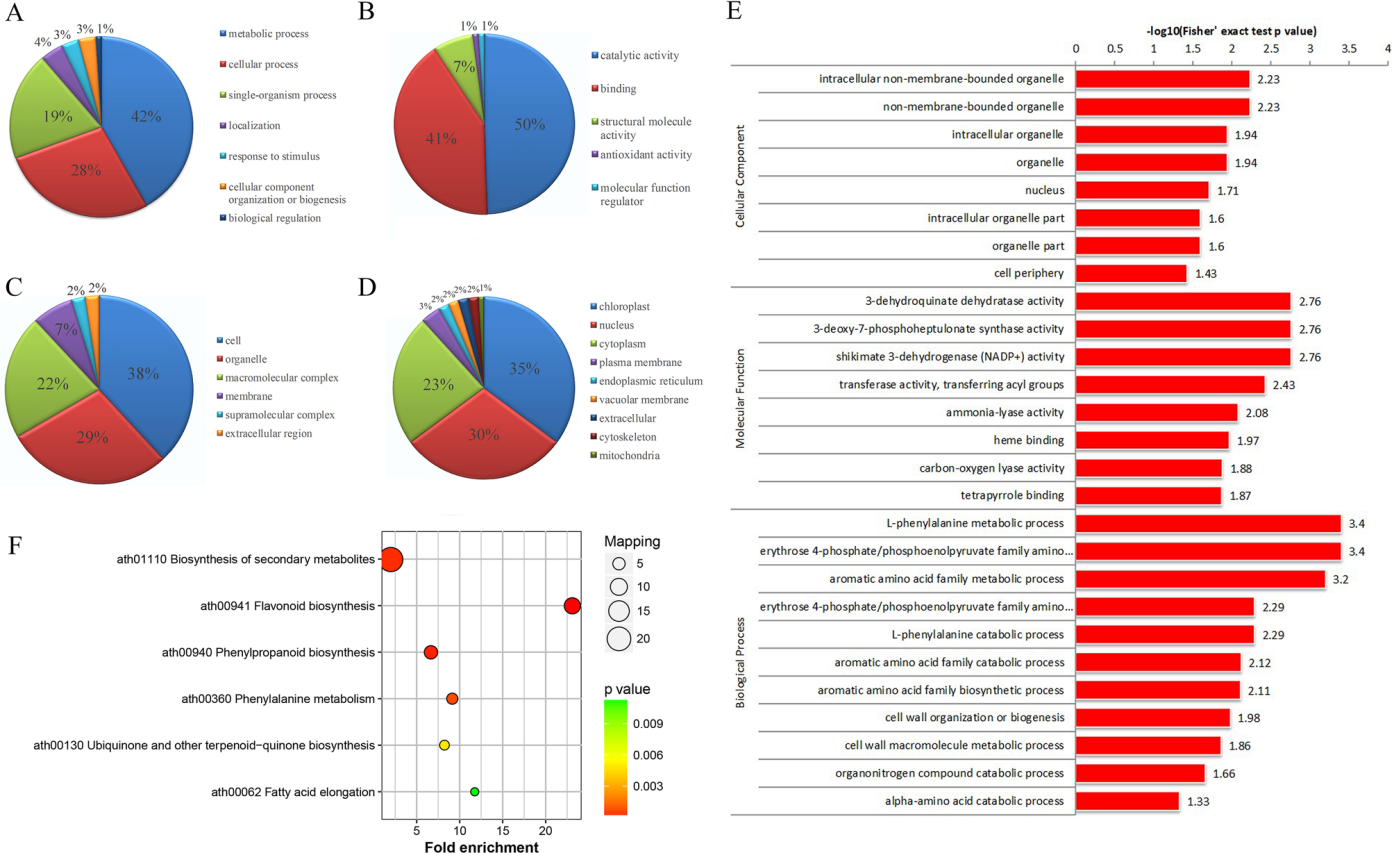
Functional classification and enrichment of DEPs. (**A**) Biological processes, (**B**) molecular functions, (**C**) cellular components, (**D**) subcellular localization, (**E**) GO enrichment and (**F**) KEGG enrichment.

To classify the functions of proteins in tea plant under drought stress, we performed GO functional classifications of DEPs based on their associated biological processes, molecular functions and cellular components (Fig. 2A–C). The results from bioinformatic predictions showed that the functions of the DEPs were mainly involved in metabolic processes involving catalytic activity and the cell. However, except for the metabolic processes and catalytic activity, the GO functional classifications of DEPs were mainly enriched in cellular processes involving binding and organelles.

WoLF PSORT software was used to predict and classify the subcellular structure of the differentially expressed proteins. Of the 111 DEPs, 39 were distributed in chloroplasts, 33 were distributed in the nucleus, 26 were in the cytoplasm, and the other proteins were distributed in the plasma membrane and vacuolar membrane. The subcellular localization for the global proteome was also calculated for comparison (Fig. 2D). According to the data, the subcellular localization of proteins in tea plants under drought stress and the global proteome showed no significant difference.

To obtain information on the functional enrichment of proteins in tea plants under drought stress, we conducted GO enrichment of DEPs based on clustering analysis (Fig. 2E; Supplementary Table S3). In the category of molecular function, the DEPs were mainly associated with 3-dehydroquinate dehydratase activity, 3-deoxy-7-phosphoheptulonate synthase activity and shikimate 3-dehydrogenase (NADP+) activity. In terms of biological processes, a large portion of the proteins mainly participated in the L-phenylalanine metabolic process, erythrose 4-phosphate/phosphoenolpyruvate family amino acid metabolic process and aromatic amino acid family metabolic process. The analysis of cellular components showed that the DEPs were mainly involved in intracellular nonmembrane-bound organelles and nonmembrane-bound organelles.

KEGG pathway analysis showed that all the identified proteins were downregulated. These proteins were strongly associated with secondary metabolite biosynthesis, such as flavonoid biosynthesis, phenylpropanoid biosynthesis and phenylalanine metabolism (Fig. 2F; Supplementary Table S4). The pathways were mainly related to the biosynthesis of polyphenols and lignin.

### Protein–protein interaction networks

**Figure 3:**
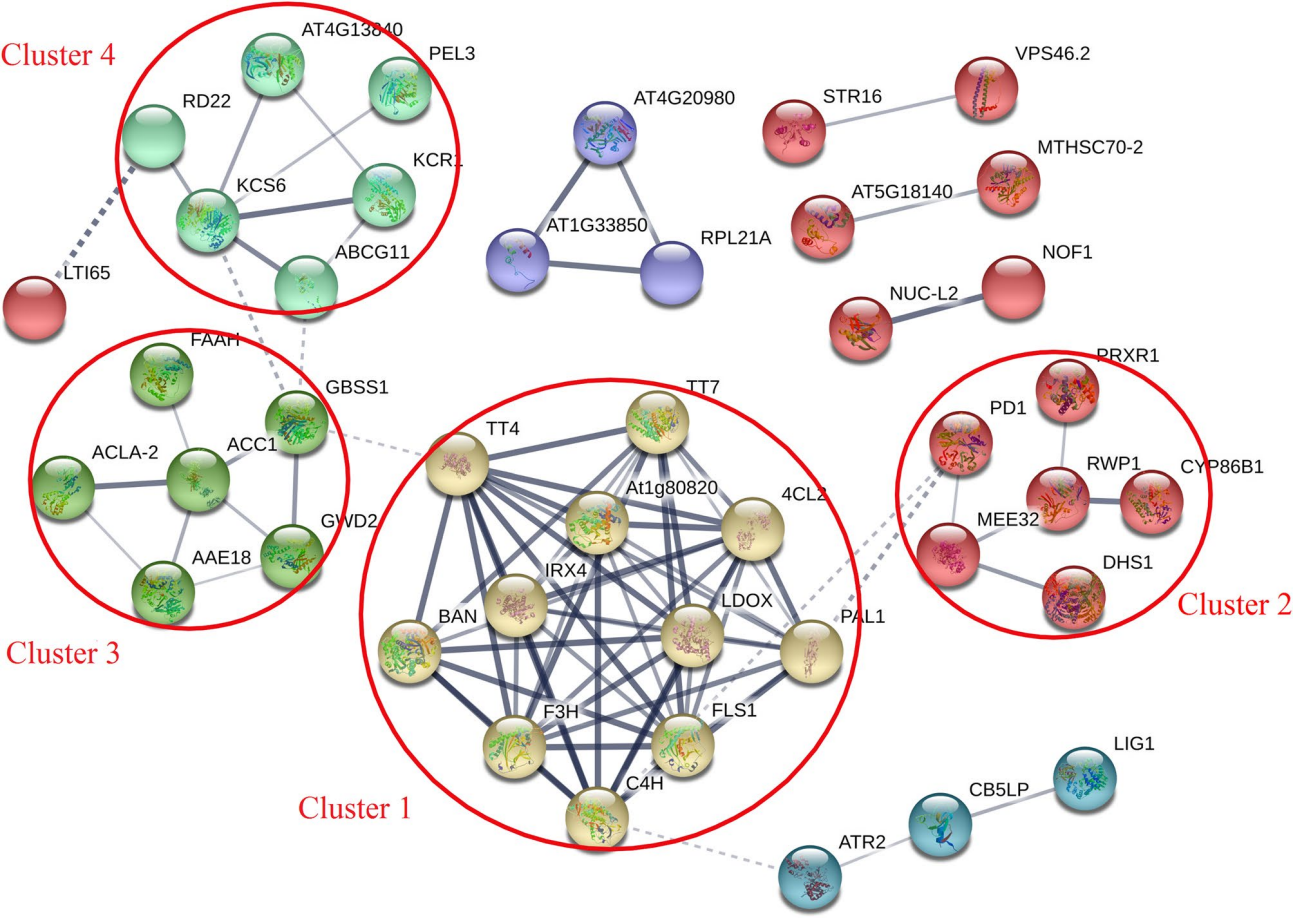
PPI networks of DEPs. The red circle represents clusters 1–4.

PPIs reflect the process by which two or more protein molecules form protein complexes through noncovalent bonds. To predict interactions between proteins, we generated PPI networks for all DEPs against the STRING database (Fig. 3; Supplementary Table S5). We extracted several interactive clusters from the entire interaction network by means of the MCODE plug-in tool kit. Among these proteins, 42 mapped to the PPI networks, and they were mainly clustered into 6 subnetworks. These subnetworks clearly show that there are different degrees of interaction between proteins. It can be predicted that DEPs, which are related to catechin biosynthesis, interact more strongly in many major metabolic processes of tea plants in response to drought stress. We will further study the specific interaction relationship in future work.

### Validation of DEPs by PRM

To verify the results of the TMT analysis at the protein level, PRM was performed among key enzymes that presented significant differences in tea plant (Table 2). In this study, 11 candidate proteins with potential drought tolerance-related functions were chosen; for each protein, two or more unique peptides with anticipated chemical stability were selected. C4H, cinnamoyl-CoA reductase (CCR), flavanone 3-hydroxylase (F3H), flavonol synthase (FLS), DFR, ANR, LDOX, GSTU, EMB, XTH, and GWD were downregulated to certain degrees, similar to the TMT results. Thus, our PRM assay revealed that the TMT results were credible, allowing further analysis.

**Table 2:** PRM analysis of eleven candidate proteins in tea plants under drought stress.

Protein accession	DT/CK ratio	DT/CK *p* value	DT/CK ratio
C4H	0.52	0.00031045	0.58
CCR	0.48	0.00112761	0.56
F3H	0.35	0.00033684	0.41
FLS	0.33	0.00017023	0.44
DFR	0.32	0.00001117	0.38
ANR	0.33	0.00095506	0.42
LDOX	0.28	0.00006974	0.42
GSTU	0.41	0.00206034	0.48
EMB	0.44	0.00060575	0.56
XTH	0.53	0.00004320	0.57
GWD	0.53	0.00017301	0.65

## Discussion

Drought stimuli induce the expression of a series of genes and the synthesis of proteins related to the drought response; this occurs via the cell perception, signalling and transport ability of plants, after which their physiological and biochemical metabolism is optimized to cope with the drought stress^19^. In this study, the accumulation of enzymes related to the biosynthesis of lignin, flavonoids and fatty acids was inhibited by drought stress, which provides a basis for further elucidating the drought resistance mechanism of tea plants.

### The accumulation of proteins related to lignin biosynthesis was inhibited by drought stress

**Figure 4:**
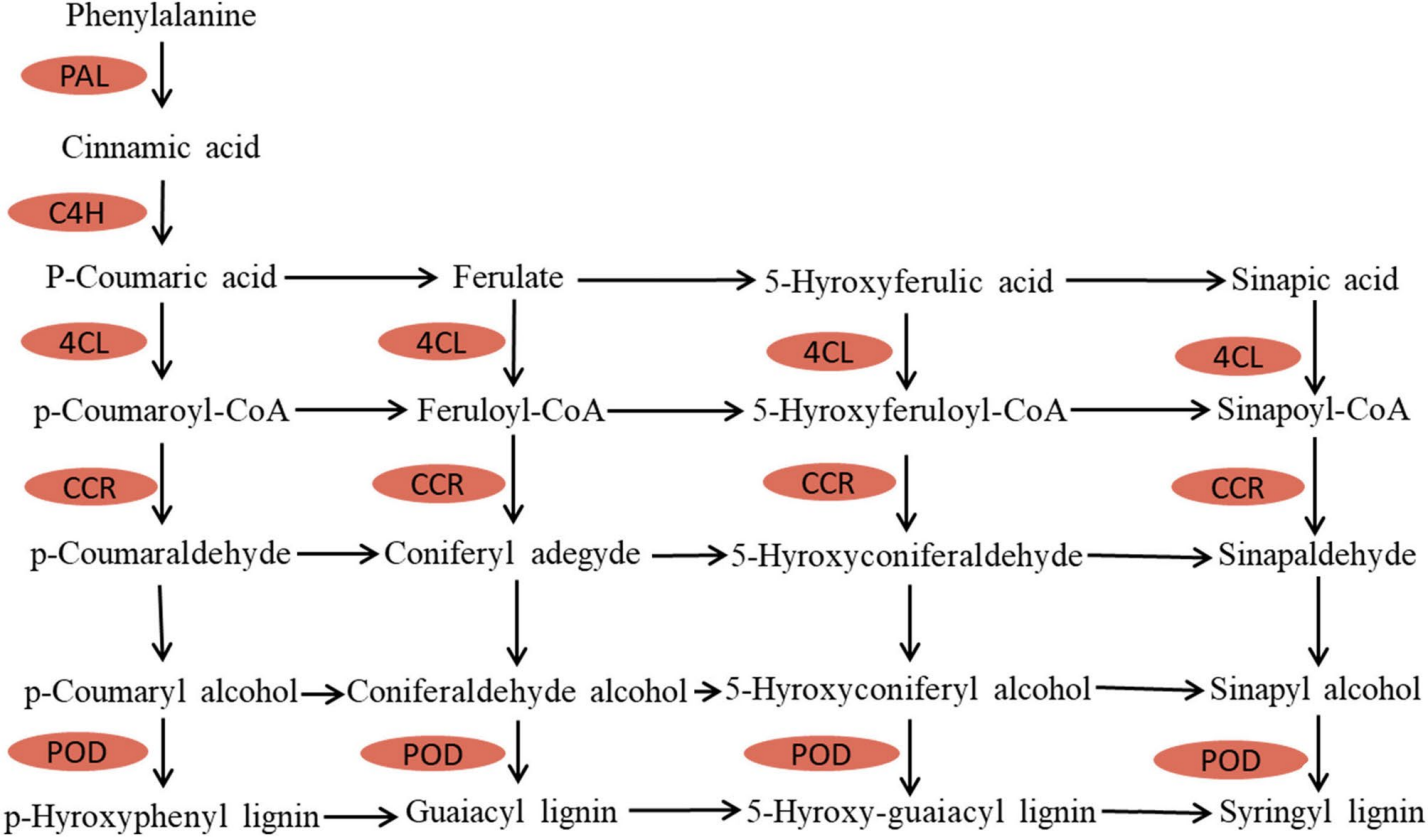
General biosynthesis pathway of lignin in plants. PAL, phenylalanine ammonia lyase; C4H, trans-cinnamate 4-monooxygenase; 4CL, 4-coumarate-CoA ligase; CCR, cinnamoyl-CoA reductase; POD, peroxidase. The orange colour indicates a downregulated protein.

As one of the important components of the plant cell wall, lignin is of great significance for plant growth and environmental adaptability. To determine the accumulation levels of proteins related to the lignin pathway, we evaluated the response of the phenylpropanoid pathway to drought stress (Fig. 4). The enzymatic steps leading to the synthesis of lignin monomers have been extensively reported^20–22^.

As the first step of the phenylpropanoid pathway, PAL mainly breaks down phenylalanine into cinnamic acid^23^. A previous study showed that the activity of PAL was significantly correlated with the content of lignin^24^. Additionally, inhibition of PAL activity can lead to a decrease in lignin biosynthesis^25–27^. In addition, a strong positive correlation between the expression of both *CsPAL* and *CsC4H* and catechin content in tea plants has been shown^28^. However, there is little research about the correlation of PAL accumulation with lignin content in tea plants under drought stress. In our study, the accumulation of PAL was downregulated, while the activity of PAL increased. The content of lignin also increased. Therefore, we speculated that the increase in lignin content under drought was not only positively correlated with the activity of PAL but also negatively correlated with the accumulation of PAL. In our previous study, we found that PAL in tea plants was ubiquitinated in response to drought stress, which was related to the L-phenylalanine metabolic process^18^. Whether ubiquitinated PAL or other factors are involved in the synthesis of lignin need further study.

As the first specific step in the synthesis of lignin monomers, CCR catalyses the conversion of cinnamoyl-CoA esters to their corresponding cinnamaldehydes^29^. A previous study showed that when CCR activity is severely inhibited, the lignin content decreases accordingly^27^. The present study showed that the lignin content increased during the development of leaves in both tea plant cultivars Fudingdabai and Suchazao, while the expression level of *CsCCR* was not uniform^30^. Recent research has shown that the accumulation of CCR increases in the roots and decreases in the leaves of tea plants under high concentrations of Al stress, while the lignin content decreases in both organs^31^. In our study, the accumulation of CCR was downregulated in tea plants under drought stress, while the content of lignin increased. These data indicate that drought stress may promote the accumulation of lignin by inhibiting the accumulation of CCR in tea plants.

As a specific step in the late stages of lignin synthesis, the polymerization of monolignols catalysed by peroxidase (POD) and/or laccase enzymes yields lignin^32^. Previous studies have shown that abiotic stresses generally induce POD activity until it reaches a specific threshold, leading to lignin accumulation^33,34^. A previous study also showed that overexpression of *POD* increases the contents of phenols and lignin in plants^35^. An abovementioned study showed that the accumulation of POD increased in the roots but decreased in the leaves of tea plants under high concentrations of Al stress, while the lignin content decreased in both organs^31^. In our study, the accumulation of POD was downregulated in tea plants under drought stress, while the content of lignin increased. We speculate that drought stress may promote the accumulation of lignin by inhibiting the accumulation of POD in tea plants.

### The accumulation of proteins related to flavonoid biosynthesis was inhibited by drought stress

**Figure 5:**
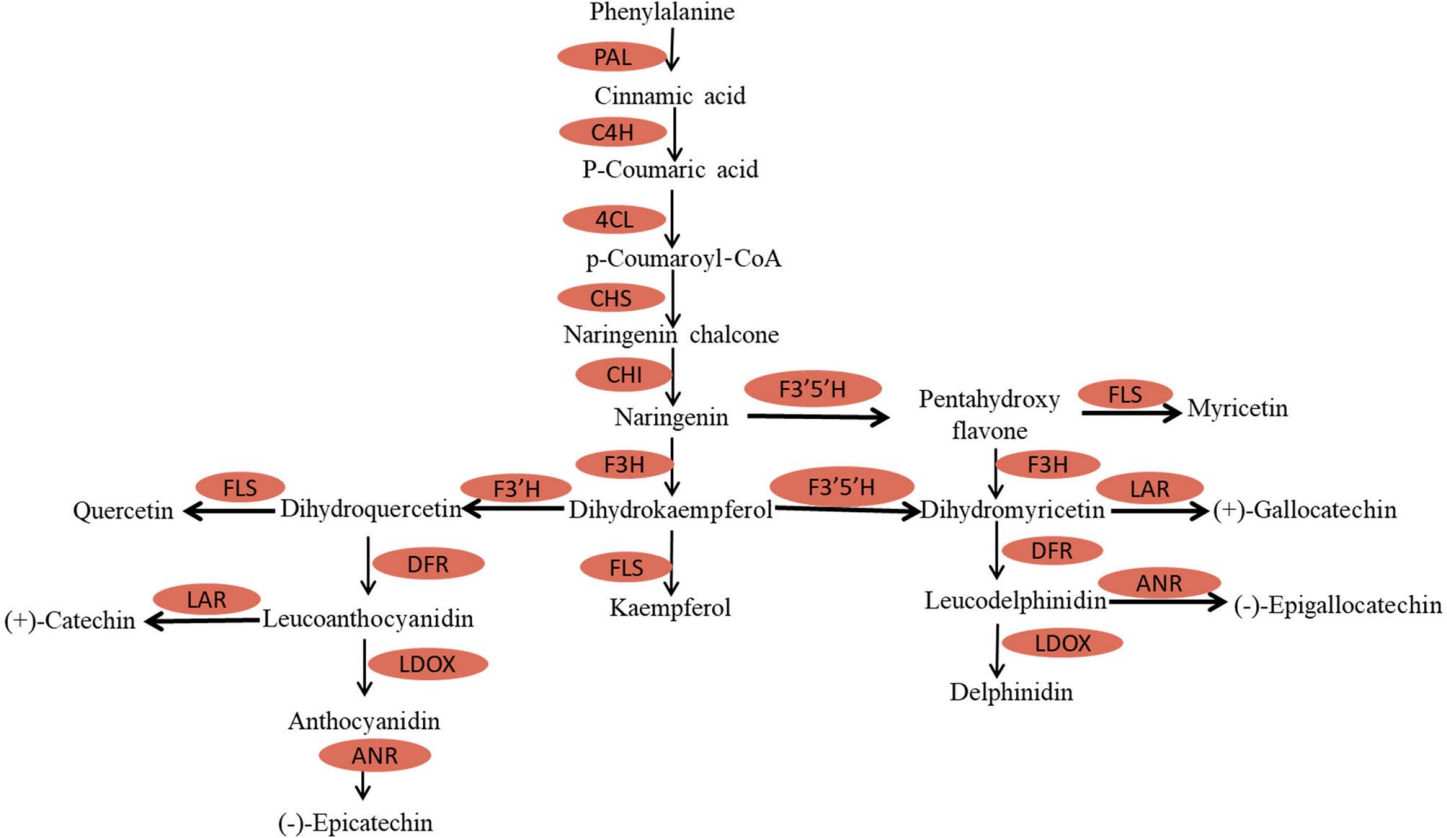
General biosynthesis pathway of flavonoids in tea plants. PAL, phenylalanine ammonia lyase; C4H, trans-cinnamate 4-monooxygenase; 4CL, 4-coumarate-CoA ligase; CHS, chalcone synthase; CHI, chalcone-flavanone isomerase; F3H, flavanone 3-hydroxylase; FLS, flavonol synthase; ANR, anthocyanidin reductase; LAR, leucoanthocyanidin reductase; LDOX, leucoanthocyanidin dioxygenase; F3′H, flavonoid 3′ hydroxylase; F3′5′H, flavonoid 3′,5′-hydroxylase; DFR, dihydroflavonol-4-reductase. The orange colour indicates a downregulated protein.

Flavonoids are important components of tea products and are closely related to the taste, flavour, and health benefits of tea. Recent studies have revealed that plants can improve their drought, salt and other abiotic resistance by increasing the accumulation of flavonoids^36–39^. In our study, to determine the influence of proteins related to catechin biosynthesis under drought stress, we mapped the pathway of proteins involved in the phenylpropanoid and flavonoid pathways (Fig. 5). The enzymatic steps leading to the synthesis of these flavonoid monomers have been extensively reviewed^40^.

As the first committed step of the flavonoid pathway, chalcone synthase (CHS) utilizes a tetraketide intermediate by condensation with one 4-coumaroyl-CoA and three malonyl-CoA compounds to form naringenin chalcone^41,42^. A previous study has showed that the activities of C4H, a key enzyme that functions upstream of baicalin biosynthesis, are consistent with the accumulation of baicalin^43^. In addition, the expression of multi-copy flavonoid pathway genes (*Chss*, *Chs2*, and *Chs3*) coincides with anthocyanin, flavonol and flavan-3-ol accumulation in grapevine^44^. In the present study, the expression level of *CHS* was not related to the concentration of catechin in tea leaves but was related to the concentration of O-glycosylated flavonols^45^. The expression levels of unigenes such as *CHS* tended to first decrease but then increase in tea plants in response to drought stress, while the total flavonoid content was upregulated^7^. However, relatively little is known about the regulation of CHS at the protein level of tea plants under drought stress. In this study, it is noteworthy that the accumulation of CHS under drought stress was lower than that in the controls, while the content of flavonoids increased. Taken together, these results demonstrate that drought stress may promote the accumulation of flavonoids by inhibiting the accumulation of CHS in tea plants.

F3H is an abundant enzyme in tea plants that catalyses the stereo-specific hydroxylation of (2S)-naringenin to form (2R,3R)-dihydrokaempferol^46^. A previous study showed that drought stress induced an increase in the expression of the *RsF3H* gene, enzyme activity and the contents of total flavonoid^47^. A recent study showed that the expression level of *F3H* was not related to the concentration of catechin in tea leaves but was related to the concentration of Pas^45^. In tea plants, the expression of *CsF3H* is downregulated in response to drought, abscisic acid and gibberellic acid treatment but upregulated in response to wounding, and the concentration of catechins paralleled the expression data^46^. Additionally, negative correlations between *F3H* and *anthocyanidin synthase* expression levels and catechin content were identified in spring tea plants, whereas the correlations were positive in autumn tea plants^48^. However, previous studies have primarily focused on the cloning, expression and regulation of the gene at the transcriptional level, and little is known F3H accumulation at the protein level. In this study, the accumulation of F3H was downregulated, and the contents of flavonoids increased in response to drought stress, indicating that drought stress may promote the accumulation of flavonoids by inhibiting the accumulation of F3H in tea plants.

FLS catalyses dihydroflavonols into flavonols, and FLS, as the key enzyme involved in flavonol synthesis, determines the final contents of flavonols in plants^49^. A previous study suggested that the activity of FLS was highly positively correlated with the total contents of flavonols during grape berry development, and FLS accumulation was essentially consistent with FLS activity at most stages^49^. Another study showed that the unigenes *FLS* and *flavone synthase* were continuously upregulated in tea plants under drought stress, which may be the reason for the increase in total flavonoid contents in response to drought stress^7^. However, in tea plants, there is little information about the regulation of FLS at the protein level in response to drought stress. In our study, the accumulation of FLS was downregulated in the tea plants, while the content of lignin increased, indicating that drought stress could affect the accumulation of flavonoids by inducing FLS accumulation.

### Response of LCFAs to drought stress

Fatty acids are the main components of cell membrane lipids and the precursor of some signalling molecules in plants. They are widely distributed on the cell surface to prevent moisture and heat loss and are closely related to cell recognition specificity and tissue immunity. However, relatively little is known about long-chain fatty acid amides: most of the well-defined physiological functions, the metabolism underlying their biosynthesis, their degradation, and their cellular transport remain elusive^50–52^. In this study, we focused on the accumulation of essential enzymes related to the biosynthesis of lignin, flavonoids and fatty acids in tea plants in response to drought stress at the protein level.

The first step of fatty acid biosynthesis catalysed by plastid ACCase activity is the main determinant of the overall rate of fatty acid synthesis^53^. Previous studies have shown that acetyl-CoA carboxylase 1 (ACC1) is essential for very long-chain fatty acid elongation and embryo development in *Arabidopsis*^54^. *P. simonii Populus simonii ACC1*, which is highly similar to the *Arabidopsis acc1* gene, increased in response to drought stress, which suggests that the biosynthesis of LCFAs may play an important role in the drought resistance of *Populus simonii*^55^. However, in tea plants, there is no information about the regulation of ACC1 in response to drought stress. In our study, the accumulation of ACC1 was downregulated, which indicated that tea plants could improve their drought resistance by inhibiting the accumulation of ACC1.

3-Ketoacyl-CoA synthase (KCS) catalyses a condensation reaction to form 3-ketoacyl-CoA during very long-chain fatty acid synthesis^52^. A previous study showed that the phenotypic changes of kcs1-1 mutants included relatively thin stems and reduced resistance to low-humidity stress at a young age^56^. Moreover, a recent study showed that the transcription of most lipid biosynthesis genes, such as *KCS6*, increases during the rapid elongation stage and is maintained at a high level; moreover, the fatty acid biosynthetic pathway is also significantly induced in AKR2A-57 fibres^57^. In the present study, KCS-6 accumulation was downregulated, which indicated that tea plants could improve drought resistance by inhibiting the accumulation of KCS-6.

A subsequent reaction involves the reduction to 3-hydroxyacyl-CoA catalysed by 3-ketoacyl-CoA reductase (KCR)^58^. Previous studies have shown that *GhKCR*s are involved in endoplasmic reticulum-associated very long-chain fatty acid elongation during cotton fibre development^59^. At present, there is no evidence on the response of proteins related to the LCFA synthesis pathway in response to drought stress in tea plants. Our data indicated that the accumulation of ACC1, KCS-6, and KCR in the LCFA synthesis pathway was downregulated, which indicated that tea plants could improve drought resistance by inhibiting the accumulation of essential enzymes related to the biosynthesis of lignin, flavonoids and fatty acids. With global climate change and increasing water scarcity, in-depth studies on the relationships between long-chain fatty acids and key enzymes involved in the biosynthesis pathway are of great significance to improve the drought resistance of tea plants, which needs further research.

## Supplementary information

Supplementary Table S1.

Supplementary Table S2.

Supplementary Table S3.

Supplementary Table S4.

Supplementary Table S5.

## Data Availability

The datasets generated during the current study are available via ProteomeXchange under identifier PXD011688, (https://www.ebi.ac.uk/pride).
