# Supplementary material for: Drought stress triggers proteomic changes involving lignin, flavonoids and fatty acids in tea plants
Source: Sci Rep. 2020 Sep 23;10:15504. doi: 10.1038/s41598-020-72596-1 (PMC7511325; doi:10.1038/s41598-020-72596-1)
Supplement: Supplementary file 2 — Supplementary Table S2. [file 41598_2020_72596_MOESM2_ESM.pdf]

Drought Stress Triggers the Proteomic Changes Involved in Lignins, Flavonoids and Fatty Acids in Tea Plants

Honglian Gu[1], Yu Wang[1], Hui Xie1, Chen Qiu[1], Shuning Zhang[1], Jun Xiao[2], Hongyan Li[3], Liang Chen[4], Xinghui Li[5], Zhaotang Ding[1,*]
1 Tea Research Institute, Qingdao Agricultural University, Qingdao, Shandong266109, China.
2 School of Biological Science and Winery Engineering, Taishan University, Taian, Shandong271000, China.
3 Haiyang fruit technology promotion station, Yantai, Shandong265100, China.
4 Tea Research Institute, Chinese Academy of Agricultural Sciences, Hangzhou, Zhejiang310008, China.
5 Tea Research Institute, Nanjing Agricultural University, Nanjing, Jiangsu210095, China.
guhongliantea@163.com (H.G.); wangyutea@163.com (Y.W.); xiehuitea@163.com (H.X.); qiuchentea@163.com (C.Q.); zhangshuningtea@163.com (S.Z.); xiaojun8299@163.com (J.X.); sunmijia0710@163.com (H.L.); liangchen@tricaas.com (L.C.); lxh@njau.edu.cn (X.L.).
*Correspondence: dzttea@163.com; Tel: +85-15853260396

Supplementary Table S2. Protein annotation

Protein accession	Protein description	MW [kDa]	Score	Coverage [%]	Peptides	PSMs	Unique peptides	CK1	CK2	CK3	DT1	DT2	DT3	DT/CK Ratio	DT/CK P value
cds.CL102Contig1_m.19364	PREDICTED: elongation factor 1-alpha [Juglans regia]	49.307	98.797	40	20	83	14	0.987	0.966	0.967	1.004	1.016	1.028	1.044	1.18E-02
cds.CL1030Contig1_m.1728	PREDICTED: myosin-11 [Jatropha curcas]	121.45	7.0306	3.7	4	4	4	1.026	1.075	1.026	0.93	0.953	0.984	0.917	1.83E-02
cds.CL1053Contig1_m.23076		46.32	10.38	13.5	5	5	5	0.985	0.956	0.974	1.037	1.022	1.068	1.073	1.09E-02

cds.CL106Contig2_m.1683	PREDICTED: serine/arginine-rich splicing factor SC35-like [Ipomoea nil]	31.737	2.4863	4.4	1	1	1	1.041	0.996	0.975	1.009	1.019	0.966	0.994	8.28E-01
cds.CL1156Contig1_m.12309	PREDICTED: probable aminopyrimidine aminohydrolase, mitochondrial isoform X2 [Pyrus x bretschnei	44.766	8.0998	8.6	4	5	3	0.972	0.95	1.004	1.042	1.014	1.12	1.085	7.33E-02
cds.CL1156Contig1_m.12310	PREDICTED: probable aminopyrimidine aminohydrolase, mitochondrial isoform X2 [Vitis	26.967	7.3566	8.8	2	3	1	1.019	0.892	0.973	1.004	1.087	1.041	1.086	1.39E-01

cds.CL116Contig1_m.2773	PREDICTED: alanine--tRNA ligase, chloroplastic/mitochondrial [Vitis vinifera]	39.694	13.502	16.5	6	6	4	1.018	1.095	0.974	1.013	0.991	0.918	0.947	2.95E-01
cds.CL116Contig1_m.2777	PREDICTED: probable alanine--tRNA ligase, chloroplastic [Jatropha curcas]	12.511	27.73	27	3	3	3	0.942	1.037	1.044	0.978	1.027	0.987	0.99	8.08E-01
cds.CL1177Contig1_m.9340	PREDICTED: ubiquitin carboxyl-terminal hydrolase MINDY-2 isoform X1 [Vitis vinifera]	62.451	6.3082	7.4	4	4	3	0.992	1.008	1.024	1.015	1.014	0.968	0.991	6.41E-01
cds.CL117Contig1_m.44135	PREDICTED: actin-7-like [Fragaria vesca subsp. vesca]	24.496	8.0323	48	10	83	0								

cds.CL1185Contig1_m.11093	PREDICTED: factor of DNA methylation 1 isoform X2 [Vitis vinifera]	73.837	2.2803	2.1	1	1	1	1.067	1.101	1.142	0.956	0.858	0.858	0.807	6.34E-03
cds.CL1210Contig1_m.9829	PREDICTED: serine protease SPPA, chloroplastic [Vitis vinifera]	77.864	82.858	26	18	24	18	1.023	1.064	1.026	0.942	0.934	0.995	0.922	2.60E-02
cds.CL1253Contig1_m.16152	PREDICTED: acidic leucine-rich nuclear phosphoprotein 32-related protein [Vitis vinifera]	52.56	9.4981	3.4	1	1	1								
cds.CL1254Contig1_m.23559	PREDICTED: serine carboxypeptidase-like 51 [Sesamum indicum]	27.468	8.0864	16.5	5	6	5	0.942	0.954	0.947	1.077	1.035	1.004	1.096	1.15E-02
cds.CL127Contig1_m.47294	PREDICTED: cullin-1-like isoform X4 [Nicotiana tabacum]	26.008	1.6763	9	2	2	1								

cds.CL1290Contig1_m.20447	PREDICTED: phospholipase SGR2-like [Nicotiana tabacum]	22.067	9.9686	9.5	1	1	1								
cds.CL1310Contig1_m.8622	Myc2 bHLH protein [Vitis vinifera]	68.241	1.1529	1.8	1	2	1	0.901	0.962	0.958	1.126	1.026	1.064	1.14	1.90E-02
cds.CL131Contig2_m.19755	PREDICTED: ATP-dependent zinc metalloprotease FTSH 11, chloroplastic/mitochondrial isoform X1 [Sesamum indicum]	57.774	11.676	9.1	4	4	4								
cds.CL1334Contig1_m.16732	PREDICTED: AP-1 complex subunit mu-2 [Nelumbo nucifera]	45.632	5.5313	6.5	3	4	3	0.96	1.021	1.044	1.017	0.972	0.944	0.97	4.06E-01

cds.CL1340Contig1_m.43986	PREDICTED: crocetin glucosyltransferase, chloroplastic-like [Nelumbo nucifera]	26.525	5.3964	13.2	3	3	3	0.943	1.056	0.978	0.986	1.025	1.027	1.02	5.86E-01
cds.CL1357Contig1_m.25269	PREDICTED: heparanase-like protein 3 [Ziziphus jujuba]	22.003	10.872	11.9	2	2	2	1.097	1.037	1.069	0.921	0.967	0.894	0.869	7.34E-03
cds.CL1367Contig1_m.12959	PREDICTED: 4-hydroxy-3-methylbut-2-en-1-yl diphosphate synthase (ferredoxin), chloroplastic [Vitis vinifera]	85.391	198.51	29.1	20	40	20	1.02	1.081	1.026	0.934	0.941	0.963	0.908	9.38E-03
cds.CL1371Contig1_m.20317	PREDICTED: UDP-glycosyltransferase 708A6-like [Ziziphus jujuba]	55.498	1.9449	3.4	2	2	2	1.291	1.277	1.364	0.711	0.649	0.62	0.504	1.04E-04

cds.CL1397Contig1_m.17638	PREDICTED: cytochrome P450 CYP72A219-like [Nicotiana tabacum]	60.644	8.7646	7	4	4	3	0.952	0.959	0.956	1.102	1.085	0.975	1.103	1.29E-01
cds.CL1408Contig1_m.16271	PREDICTED: ectonucleotide pyrophosphatase/phosphodiesterase family member 3-like [Nicotiana tomentosiformis]	54.985	1.5956	4.3	2	3	1	1.049	0.964	1.081	1.045	0.944	0.92	0.94	2.98E-01
cds.CL1412Contig1_m.34245	PREDICTED: alpha-soluble NSF attachment protein-like [Erythranthe guttata]	34.165	54.64	30.8	7	9	6	0.971	0.951	0.944	1.05	1.054	1.049	1.1	3.64E-04
cds.CL1426Contig1_m.12123	PREDICTED: glycerol kinase [Theobroma cacao]	36.076	65.239	18.3	6	11	6	0.919	0.906	0.91	1.108	1.112	1.047	1.195	8.82E-04

cds.CL1426Contig1_m.12124	PREDICTED: glycerol kinase-like [Sesamum indicum]	13.942	2.8054	14.1	2	3	2	0.878	0.905	0.922	1.037	1.067	1.109	1.188	2.04E-03
cds.CL1435Contig1_m.3955	PREDICTED: phagocyte signaling-impaired protein isoform X1 [Vitis vinifera]	69.451	3.0933	2.6	2	2	2	0.934	0.92	0.982	1.046	1.1	1.048	1.126	9.78E-03
cds.CL1435Contig1_m.3956	PREDICTED: phagocyte signaling-impaired protein isoform X3 [Cucumis melo]	27.59	3.7463	4.5	1	1	1	0.986	0.968	1.057	1.047	0.977	0.979	0.997	9.48E-01
cds.CL1436Contig1_m.4483	acetyltransferase-related family protein [Populus trichocarpa]	80.427	17.449	12.8	9	10	9	1.119	1.161	1.124	0.893	0.84	0.884	0.769	2.95E-04
cds.CL1444Contig1_m.12503	PREDICTED: pyruvate decarboxylase 1 [Erythranthe guttata]	69.705	6.991	7.3	5	7	3	0.976	0.928	0.997	1.087	1.001	1.008	1.067	1.28E-01

cds.CL1451Contig1_m.46213	transferring glycosyl group transferase [Medicago truncatula]	19.324	3.9875	7.3	1	2	1	0.985	1.043	0.991	0.988	1.04	0.964	0.991	7.66E-01
cds.CL1472Contig1_m.15003	PREDICTED: carboxyl-terminal-processing peptidase 2, chloroplastic-like isoform X2 [Juglans regia]	37.106	72.742	35.9	11	15	10	1.007	1.021	0.968	1.017	0.951	1.003	0.992	7.59E-01
cds.CL1488Contig1_m.8339	PREDICTED: conserved oligomeric Golgi complex subunit 2 [Ricinus communis]	83.986	3.4794	2.5	2	2	2	0.93	1.126	1.024	0.976	0.986	0.968	0.951	4.87E-01
cds.CL1496Contig1_m.28002	PREDICTED: cinnamoyl-CoA reductase 1 [Ipomoea nil]	40.663	18.225	17.8	7	14	7	0.947	0.967	0.958	1.035	1.043	1.024	1.08	6.64E-04

cds.CL1507Contig1_m.34653	PREDICTED: elongation factor 1-delta-like [Daucus carota subsp. sativus]	25.236	56.763	46.4	9	27	7	0.95	0.957	0.961	1.045	1.075	1.049	1.105	4.61E-04
cds.CL1528Contig1_m.12829	PREDICTED: uridine-cytidine kinase C isoform X1 [Jatropha curcas]	74.6	19.08	11.7	7	8	7	0.998	1.011	0.985	1.024	0.988	0.985	1.001	9.54E-01
cds.CL1535Contig1_m.21159	PREDICTED: 5'-nucleotidase SurE-like [Prunus mume]	32.59	4.3828	2.9	1	1	1	1.008	0.937	0.999	0.985	0.979	1.101	1.041	4.27E-01
cds.CL1559Contig1_m.17467	2-phosphoglycerate kinase [Medicago truncatula]	50.321	9.5714	2.2	1	1	1	0.909	1.027	1.061	0.951	1.044	1.024	1.007	8.82E-01

cds.CL1561Contig1_m.17435	PREDICTED: palmitoyl-protein thioesterase 1 isoform X1 [Theobroma cacao]	41.191	2.4941	4.3	2	2	2	1.039	1.101	1.046	0.942	0.948	0.915	0.88	3.96E-03
cds.CL1573Contig1_m.5993	PREDICTED: coatomer subunit gamma-2 [Jatropha curcas]	103.23	98.108	21.5	17	22	17	1.007	0.99	1.025	0.989	1.012	1.01	0.996	7.87E-01
cds.CL1574Contig1_m.10547	PREDICTED: leucine--tRNA ligase, cytoplasmic-like [Solanum pennellii]	87.197	44.376	20.2	13	15	8	0.947	0.99	0.973	0.993	1.053	1.018	1.053	7.36E-02
cds.CL1593Contig1_m.10476	PREDICTED: dnaJ homolog subfamily C member 16 [Jatropha curcas]	74.663	6.9919	3.6	2	2	2	0.904	1.105	1.147	0.941	1.011	0.902	0.904	3.00E-01

cds.CL1609Contig1_m.16739	PREDICTED: calcium-dependent protein kinase 1-like [Nelumbo nucifera]	57.202	39.498	21.6	9	10	5	0.876	0.994	0.935	1.11	1.012	1.035	1.125	6.08E-02
cds.CL1635Contig1_m.8106	PREDICTED: transketolase, chloroplastic-like [Gossypium hirsutum]	41.639	159.07	26.8	8	31	8	0.973	0.956	0.959	1.022	1.048	1.027	1.072	1.76E-03
cds.CL1635Contig1_m.8107	PREDICTED: transketolase, chloroplastic-like, partial [Juglans regia]	29.958	137.79	40.9	11	48	8	0.973	0.963	0.982	1.05	1.029	1.023	1.063	3.24E-03
cds.CL1645Contig1_m.49449	PREDICTED: exosome component 10 isoform X2 [Prunus	17.508	1.302	8.3	1	1	1								

cds.CL164Contig1_m.696	PREDICTED: plasma membrane ATPase 4 isoform X1 [Theobroma cacao]	105.14	131.71	35.3	31	66	12	1.021	0.977	1.032	0.986	1.011	0.998	0.988	5.66E-01
cds.CL164Contig1_m.698	PREDICTED: photosynthetic NDH subunit of lumenal location 5, chloroplastic [Ziziphus jujuba]	28.353	99.231	36	11	39	9	1.044	1.022	1.043	0.986	0.96	0.976	0.94	4.02E-03
cds.CL1670Contig1_m.28637	PREDICTED: CBS domain-containing protein CBSX1, chloroplastic-like isoform X3 [Nicotiana attenuata]	30.077	18.033	13.4	5	9	4	1.037	1.014	1.041	0.996	1	0.963	0.957	3.85E-02

cds.CL1675Contig1_m.13474	PREDICTED: multiprotein-bridging factor 1b [Vigna radiata var. radiata]	15.516	21.061	27.5	6	8	6	1.069	1.089	1.065	0.905	0.884	0.958	0.852	2.96E-03
cds.CL168Contig1_m.5686	PREDICTED: ribonuclease J isoform X1 [Theobroma cacao]	93.003	3.7114	4	4	5	3	1.215	1.183	1.09	0.843	0.809	0.809	0.706	6.01E-04
cds.CL1715Contig1_m.18765	PREDICTED: serine carboxypeptidase-like 20 [Citrus sinensis]	55.629	14.729	6	3	5	3	1.133	1.081	1.074	0.893	0.905	0.888	0.817	3.41E-04
cds.CL1729Contig1_m.14968	PREDICTED: regulator of nonsense transcripts 1 homolog isoform X1 [Malus domestica]	70.475	43.035	9.1	5	5	5	0.995	0.986	0.95	1.022	1.06	1.012	1.056	5.36E-02

cds.CL1797Contig1_m.33392	PREDICTED: proteasome subunit beta type-4-like [Juglans regia]	27.608	71.281	23.2	5	12	5	0.94	0.96	0.973	1.005	1.07	1.051	1.088	1.65E-02
cds.CL1865Contig1_m.42192	PREDICTED: 50S ribosomal protein L18, chloroplastic [Prunus mume]	18.583	6.2433	11.4	3	3	3	1.106	1.154	1.201	0.866	0.828	0.814	0.725	4.37E-04
cds.CL1876Contig1_m.33456	PREDICTED: protein TIC 22-like, chloroplastic [Pyrus x bretschneideri]	26.913	62.791	27.5	5	10	5	0.957	0.974	1.041	1.019	0.911	0.994	0.984	7.14E-01
cds.CL1906Contig1_m.9375	PREDICTED: U-box domain-containing protein 14 [Sesamum indicum]	68.421	1.9885	1.4	1	1	1								

cds.CL1933Contig1_m.27314	PREDICTED: double-stranded RNA-binding protein 4 isoform X3 [Vitis vinifera]	38.172	2.1287	9.2	4	4	2	1.068	1.09	1.037	0.881	1.058	0.853	0.874	1.12E-01
cds.CL194Contig1_m.32217	PREDICTED: ATPase 4, plasma membrane-type [Cicer	44.994	23.253	49	18	41	5	0.959	0.991	0.994	0.982	1.096	1.008	1.048	2.59E-01
cds.CL1995Contig1_m.25635	PREDICTED: GDSL esterase/lipase At5g33370 [Vitis vinifera]	40.32	1.5601	2.7	1	1	1								
cds.CL2003Contig1_m.12351	PREDICTED: glycine--tRNA ligase, mitochondrial 1-like [Nicotiana attenuata]	79.364	80.449	22.3	15	30	6	0.96	0.981	0.922	1.058	1.065	1.044	1.106	6.08E-03

cds.CL2126Contig1_m.4592	PREDICTED: muscle M-line assembly protein unc-89 [Citrus sinensis]	101.96	31.676	10	8	10	8	1.069	1.055	1.046	0.94	0.948	0.947	0.894	8.18E-05
cds.CL2137Contig1_m.26096	PREDICTED: putative 12-oxophytodienoate reductase 11 [Ipomoea nil]	42.45	4.5057	7.9	3	6	2	1.037	0.986	0.983	1.011	0.966	1.001	0.991	6.98E-01
cds.CL2147Contig1_m.5684	PREDICTED: alpha-mannosidase 2 [Vitis vinifera]	105.17	8.7406	6	6	8	6	1.058	1.141	1.086	0.93	0.865	0.881	0.815	2.72E-03
cds.CL228Contig2_m.587	PREDICTED: titin homolog [Ziziphus jujuba]	89.665	2.1739	2.3	2	3	2	0.993	0.89	0.925	1.081	1.07	1.067	1.146	5.12E-02
cds.CL230Contig1_m.8625	PREDICTED: 3-dehydroquinate synthase, chloroplastic-like [Juglans regia]	48.666	27.352	20.7	10	17	10	1.154	1.112	1.148	0.839	0.86	0.854	0.748	2.47E-05

cds.CL230Contig1_m.8626	PREDICTED: glycolipid transfer protein 1-like [Nelumbo nucifera]	22.569	7.0458	12.4	2	2	2	0.978	0.985	1.088	0.969	0.93	1.055	0.968	5.56E-01
cds.CL2331Contig1_m.13695	PREDICTED: ubiquitin carboxyl-terminal hydrolase 6 [Vitis vinifera]	54.732	28.128	13.8	6	7	6	1.007	0.982	1.013	0.982	0.983	1.04	1.001	9.74E-01
cds.CL2340Contig1_m.9723	PREDICTED: regulator of nonsense transcripts 1 homolog isoform X2 [Vitis vinifera]	69.374	6.555	3.3	2	2	2	0.958	0.967	0.92	1.032	1.005	1.138	1.116	5.63E-02
cds.CL2367Contig1_m.9697	PREDICTED: flagellar radial spoke protein 5 [Ziziphus jujuba]	43.671	26.89	18.6	6	11	6	0.895	1.004	1.034	0.995	0.988	0.999	1.017	7.16E-01

cds.CL2386Contig1_m.14397	PREDICTED: peptidyl-prolyl cis-trans isomerase FKBP62 [Vitis vinifera]	62.425	-2	21.6	14	20	1								
cds.CL238Contig1_m.244	PREDICTED: E3 ubiquitin-protein ligase UPL2 [Vitis vinifera]	338.38	73.196	4.8	14	18	12	1.05	1.12	1.062	0.926	0.93	0.907	0.855	1.84E-03
cds.CL238Contig1_m.245	PREDICTED: E3 ubiquitin-protein ligase UPL2 [Vitis vinifera]	70.383	6.4925	1.7	1	1	1	0.833	1.086	1.174	1.109	0.949	0.881	0.95	7.34E-01
cds.CL2400Contig1_m.14899	PREDICTED: peptidyl-prolyl cis-trans isomerase FKBP62 [Vitis vinifera]	63.76	45.213	21.3	14	20	1								

cds.CL2405Contig1_m.22784	PREDICTED: rab9 effector protein with kelch motifs [Vitis vinifera]	58.895	49.316	9.7	3	3	3	1.023	0.926	0.805	1.062	1.039	1.164	1.186	8.91E-02
cds.CL245Contig1_m.18020	PREDICTED: protein transport protein Sec61 subunit alpha-like [Juglans regia]	52.133	21.068	12.6	6	11	1	1.013	0.909	0.849	1.161	1.081	1.019	1.177	6.18E-02
cds.CL245Contig2_m.19427	PREDICTED: protein transport protein Sec61 subunit alpha-like [Daucus carota subsp. sativus]	51.988	-2	12.6	6	10	1	0.873	0.983	0.998	0.979	1.192	1.008	1.114	2.29E-01

cds.CL247Contig1_m.30180	PREDICTED: serine/threonine-protein phosphatase PP2A catalytic subunit [Eucalyptus grandis]	35.03	39.466	34.6	8	11	6	0.931	0.973	0.968	1.029	1.03	1.054	1.084	7.24E-03
cds.CL256Contig2_m.600	PREDICTED: helicase protein MOM1 isoform X1 [Vitis vinifera]	253.86	1.9074	0.4	1	4	1	0.951	0.937	0.968	1.036	1.041	1.084	1.107	4.12E-03
cds.CL2604Contig1_m.30733	PREDICTED: subtilisin-like protease SBT1.7 [Citrus sinensis]	41.299	23.066	7.6	2	4	2	0.967	0.975	0.976	1.027	1.053	1.073	1.081	4.04E-03
cds.CL2607Contig1_m.32294	PREDICTED: eukaryotic translation initiation factor 2 subunit beta [Sesamum indicum]	34.869	2.6801	8.1	2	2	2								

cds.CL260Contig1_m.2359	PREDICTED: golgin subfamily B member 1 isoform X4 [Vitis vinifera]	151.06	20.876	4.2	5	5	5	1.054	1.102	1.072	0.917	0.958	0.884	0.855	4.10E-03
cds.CL2654Contig1_m.34567	PREDICTED: myosin-11-like [Juglans regia]	43.435	9.8454	13.6	4	4	4	0.933	1.049	0.941	1.03	1.053	1.016	1.06	2.01E-01
cds.CL2675Contig1_m.36719	PREDICTED: proline synthase co-transcribed bacterial homolog protein isoform X2 [Vitis vinifera]	29.441	2.2478	10.1	3	5	2								
cds.CL2682Contig1_m.23652	PREDICTED: arogenate dehydratase/prephenate dehydratase 2, chloroplastic [Sesamum indicum]	46.301	9.521	7.9	4	5	3	0.939	0.912	0.963	1.057	1.095	1.042	1.135	4.18E-03

cds.CL2709Contig1_m.35266	PREDICTED: proteasome subunit alpha type-4 [Daucus carota subsp. sativus]	27.283	115.11	38.6	11	23	11	0.963	0.944	1.003	1.026	1.025	1.068	1.072	3.61E-02
cds.CL271Contig1_m.26532	PREDICTED: tobamovirus multiplication protein 2A-like [Gossypium hirsutum]	31.186	19.5	19.7	3	7	3	0.927	0.926	0.934	0.999	1.079	1.114	1.145	5.35E-02
cds.CL2759Contig1_m.27804	PREDICTED: reticulon-4-interacting protein 1, mitochondrial [Nicotiana tabacum]	16.774	51.014	21.3	3	5	3	0.959	0.993	1.001	1.024	1.059	1.013	1.048	6.53E-02
cds.CL2759Contig1_m.27806	PREDICTED: reticulon-4-interacting protein 1, mitochondrial [Ziziphus jujuba]	12.937	48.968	18.3	2	2	2	0.942	0.944	0.981	1.121	1.046	0.997	1.104	5.56E-02

cds.CL27Contig1_m.26912	PREDICTED: ubiquitin carboxyl-terminal hydrolase 13-like isoform X1 [Nelumbo nucifera]	36.84	42.511	16.6	7	7	6	0.997	0.976	1.01	1.03	0.996	1.01	1.018	2.75E-01
cds.CL2817Contig1_m.45088	PREDICTED: maf-like protein DDB_G0281937 [Ziziphus jujuba]	11.916	2.7867	20	2	2	2	0.757	1.404	0.972	0.948	0.933	1.002	0.92	8.04E-01
cds.CL2818Contig1_m.52277	PREDICTED: solanesyl diphosphate synthase 3, chloroplastic/mitochondrial isoform X5 [Citrus sinensis]	18.125	2.7812	6.1	1	1	1								

cds.CL2855Contig1_m.13226	PREDICTED: T-complex protein 1 subunit zeta-like isoform X2 [Nicotiana sylvestris]	59.14	116.37	40.9	17	27	17	0.939	0.936	0.932	1.078	1.083	1.036	1.139	1.18E-02
cds.CL2864Contig1_m.31320	PREDICTED: dihydrolipoyl dehydrogenase 2, chloroplastic [Vitis vinifera]	45.824	74.859	28.2	11	14	7	0.998	1.043	1.003	1.008	0.999	0.996	0.987	4.05E-01
cds.CL2872Contig1_m.26765	PREDICTED: mitotic checkpoint protein BUB3.1 isoform X2 [Nicotiana attenuata]	37.703	42.021	15	5	5	5	0.948	1.009	0.986	1.043	1.042	1.026	1.057	4.23E-02
cds.CL2893Contig1_m.49322	PREDICTED: cytochrome c oxidase assembly factor 6 isoform X2 [Erythranthe guttata]	12.615	1.1576	7.3	1	1	1								

cds.CL2899Contig1_m.9257	PREDICTED: probable polygalacturonase [Malus domestica]	33.787	3.5492	5.7	2	2	2	1.068	0.988	1.038	0.928	1.003	0.968	0.937	1.11E-01
cds.CL28Contig1_m.10182	PREDICTED: 6-phosphofructo-2-kinase/fructose-2,6-bisphosphatase [Vitis	55.994	7.6735	13.4	7	9	3	1.077	1.012	1.023	0.982	1.012	0.971	0.953	1.02E-01
cds.CL28Contig4_m.11804	PREDICTED: 6-phosphofructo-2-kinase/fructose-2,6-bisphosphatase [Vitis	61.645	57.006	17.9	9	11	4	0.979	1.038	1.029	1	1.01	0.955	0.973	3.44E-01
cds.CL2924Contig1_m.35673	PREDICTED: thaumatin-like protein [Theobroma cacao]	31.726	1.9529	3.4	1	1	1								
cds.CL300Contig2_m.5020	PREDICTED: AP-1 complex subunit gamma-2-like [Citrus sinensis]	44.087	85.051	13	4	6	4	0.952	0.949	0.961	0.989	1.062	1.093	1.099	8.98E-02

cds.CL3034Contig1_m.23283	PREDICTED: spermidine hydroxycinnamoyl transferase-like [Vitis vinifera]	53.446	5.4276	7.2	3	3	3	1.088	1.049	1.136	1.032	0.923	0.907	0.874	4.38E-02
cds.CL3080Contig1_m.32184	PREDICTED: carboxylesterase 1-like [Juglans regia]	37.081	7.4766	7.7	2	3	2	0.908	0.955	0.985	1.051	1.078	1.055	1.118	1.10E-02
cds.CL3085Contig1_m.12532	PREDICTED: endonuclease 4-like [Juglans regia]	12.539	2.1971	12.4	2	2	2	1.041	0.982	1.081	0.955	0.95	0.988	0.932	8.43E-02
cds.CL3102Contig1_m.6260	PREDICTED: UDP-D-apiose/UDP-D-xylose synthase 2-like [Nelumbo nucifera]	43.689	15.068	14.7	5	7	5	0.98	1.062	1.111	0.986	0.928	0.956	0.91	8.40E-02

cds.CL3102Contig1_m.6261	PREDICTED: polyadenylate-binding protein-interacting protein 12-like [Sesamum	36.614	2.4509	3.9	1	1	1								
cds.CL3107Contig1_m.13113	PREDICTED: protein ACCUMULATION AND REPLICATION OF CHLOROPLASTS 6, chloroplastic [Vitis vinifera]	52.03	4.6955	3.8	2	2	2	1.036	1.054	1.128	0.928	0.981	0.866	0.862	2.87E-02
cds.CL310Contig1_m.6128	PREDICTED: centrosomal protein of 164 kDa isoform X3 [Arachis	76.57	18.812	5.7	4	4	2	0.983	1.05	1.013	0.945	1.01	1.003	0.971	3.60E-01

cds.CL310Contig1_m.6129	PREDICTED: serine/threonine-protein kinase dst2-like isoform X7 [Lupinus angustifolius]	27.829	3.6606	3.5	1	1	1	1.052	1.012	0.903	1.031	1.005	1.003	1.024	6.08E-01
cds.CL3123Contig1_m.26192	PREDICTED: 2-dehydro-3-deoxyphosphooctonate aldolase 1 [Capsicum annuum]	31.744	16.186	24.1	5	6	5	1.074	1.056	1.055	0.959	0.936	0.953	0.894	2.77E-04
cds.CL3128Contig1_m.6449	Bifunctional aspartokinase/homoserine dehydrogenase [Morus notabilis]	100.28	284.78	32	23	43	13	0.975	1.015	0.961	1.055	1.027	1.023	1.052	5.49E-02

cds.CL3154Contig1_m.20832	PREDICTED: 2-oxoisovalerate dehydrogenase subunit alpha 1, mitochondrial-like isoform X1 [Nelumbo nucifera]	54.758	23.689	20.2	9	12	9	0.966	1.015	0.993	1.038	0.999	0.999	1.021	3.42E-01
cds.CL3164Contig1_m.13657	PREDICTED: probable inactive purple acid phosphatase 2 [Citrus sinensis]	73.307	27.801	16	9	12	9	1.054	1	1.015	0.971	0.946	0.99	0.947	5.71E-02
cds.CL3176Contig1_m.24113	PREDICTED: sulfite oxidase [Capsicum annuum]	37.417	3.9321	7.4	3	3	3	0.949	1.067	0.947	0.957	1.026	0.967	0.996	9.45E-01
cds.CL3183Contig1_m.34717	PREDICTED: 50S ribosomal protein L21, chloroplastic [Sesamum indicum]	20.722	2.9491	8.6	2	3	2	1.142	1.099	1.096	0.868	0.874	0.887	0.788	8.27E-05

cds.CL318Contig1_m.48735	PREDICTED: (+)-neomenthol dehydrogenase-like [Ziziphus jujuba]	14.599	67.431	57.7	7	20	7	0.965	0.951	0.967	1.068	1.052	1.019	1.089	4.58E-03
cds.CL3233Contig1_m.1855	PREDICTED: translocase of chloroplast 120, chloroplastic-like isoform X1 [Glycine max]	92.542	23.24	10.8	8	11	8	1.049	1.065	1.087	0.911	0.935	0.942	0.871	6.76E-04
cds.CL3233Contig1_m.1856	PREDICTED: translocase of chloroplast 120, chloroplastic isoform X1 [Vitis vinifera]	44.644	1.3694	1.9	1	1	1	1.097	1.138	1.116	0.922	0.844	0.858	0.783	1.10E-03
cds.CL323Contig1_m.7000	PREDICTED: vacuolar protein sorting-associated protein 35A [Theobroma cacao]	63.014	8.1278	8.2	5	5	5	0.926	0.975	1.027	1.04	0.991	1.056	1.054	2.07E-01

cds.CL323Contig1_m.7001	PREDICTED: vacuolar protein sorting-associated protein 35A-like [Malus domestica]	23.016	5.1691	13.1	3	3	3	0.932	1.065	0.945	1.139	1.003	0.944	1.049	5.41E-01
cds.CL3251Contig1_m.45324	PREDICTED: ubiquitin carboxyl-terminal hydrolase 12-like [Malus domestica]	28.091	5.2496	7.3	2	2	2								
cds.CL3258Contig1_m.25630	PREDICTED: cell division control protein 2 homolog A-like [Solanum tuberosum	33.865	-2	5.8	2	3	1	1.081	1.111	1.048	0.905	0.924	0.912	0.846	7.39E-04
cds.CL3259Contig1_m.25268	calmodulin-binding protein [Arabidopsis lyrata subsp. lyrata]	20.49	1.353	11.5	2	2	2	0.965	1.03	0.906	1.067	1.042	1.011	1.075	1.40E-01

cds.CL3262Contig1_m.34763	PREDICTED: protein EARLY RESPONSIVE TO DEHYDRATION 15-like [Daucus carota subsp. sativus]	24.665	15.181	7.1	1	2	1	0.853	0.957	0.976	1.14	1.037	1.083	1.17	3.44E-02
cds.CL3284Contig1_m.30235	PREDICTED: cytochrome P450 89A2-like [Nicotiana tabacum]	44.755	11.535	14.8	6	8	3	0.875	0.969	0.979	1.174	1.006	1.033	1.138	9.78E-02
cds.CL3299Contig1_m.20125	PREDICTED: 7-deoxyloganetin glucosyltransferase [Vitis vinifera]	40.612	2.9666	5.1	2	3	1								
cds.CL3323Contig1_m.32482	PREDICTED: thioredoxin-like 4, chloroplastic isoform X1 [Vitis vinifera]	21.703	1.2624	3.7	1	1	1	1.068	0.989	1.051	0.919	0.993	0.972	0.928	8.53E-02

cds.CL3325Contig1_m.15610	PREDICTED: luminal-binding protein 4-like [Nelumbo nucifera]	77.071	7.6128	13.1	9	16	3	1.177	1.137	1.128	0.887	0.88	0.763	0.735	3.60E-03
cds.CL3327Contig1_m.35996	PREDICTED: putative UDP-glucose flavonoid 3-O-glucosyltransferase 3 [Fragaria vesca subsp. vesca]	40.281	40.817	12.7	4	12	4	0.971	0.97	1.066	0.985	1.011	1.028	1.006	8.57E-01
cds.CL3334Contig1_m.18342	PREDICTED: protein HGV2 isoform X2 [Vitis vinifera]	50.412	5.8536	6.3	3	3	3	1.142	1.065	1.238	0.847	0.809	0.861	0.731	2.74E-03
cds.CL3362Contig1_m.38227	PREDICTED: REF/SRPP-like protein At3g05500 [Juglans regia]	31.545	21.731	16.6	4	7	4	0.905	0.955	0.998	1.059	1.075	1.034	1.108	2.72E-02

cds.CL337Contig1_m.34411	PREDICTED: uncharacterized isomerase BH0283-like isoform X1 [Citrus sinensis]	32.115	9.7744	7.5	2	4	2	0.962	0.857	1.003	1.045	1.049	1.11	1.135	6.48E-02
cds.CL3387Contig1_m.17553	PREDICTED: polyadenylate-binding protein RBP47 [Sesamum	47.629	20.274	2.1	1	1	1	0.979	0.997	0.947	1.064	1.037	0.996	1.06	7.75E-02
cds.CL3404Contig1_m.20723	PREDICTED: probable membrane metalloprotease ARASP2, chloroplastic [Vitis vinifera]	48.816	4.1136	7.5	3	5	3	0.985	0.94	1.115	0.942	1.013	1.013	0.976	7.17E-01
cds.CL3427Contig1_m.23699	PREDICTED: putative disease resistance protein At1g50180 isoform X2 [Vitis vinifera]	51.693	2.1801	2	1	1	1								

cds.CL3440Contig1_m.18521	PREDICTED: dol-P-Man:Man(6)GlcNAc(2)-PP-Dol alpha-1,2-mannosyltransferase [Nicotiana tomentosiformis]	25.787	34.245	7.8	1	1	1								
cds.CL3443Contig1_m.6184	PREDICTED: linoleate 13S-lipoxygenase 2-1, chloroplastic-like [Ziziphus jujuba]	105.36	11.229	36.8	37	96	1	0.909	0.939	0.97	1.153	1.007	1.096	1.155	3.06E-02
cds.CL3460Contig1_m.13358	PREDICTED: protein CASP isoform X1 [Vitis vinifera]	79.134	2.6519	2.2	2	2	2	1.041	1.008	1.048	0.966	0.962	0.973	0.937	6.36E-03
cds.CL3471Contig1_m.24104	PREDICTED: alpha-galactosidase 3 [Malus domestica]	46.934	2.5192	10.6	5	13	1	0.973	0.956	0.949	1.02	1.039	1.082	1.091	1.00E-02

cds.CL3474Contig1_m.38487	PREDICTED: acetyl-coenzyme A carboxylase carboxyl transferase subunit alpha, chloroplastic-like [Nicotiana tabacum]	26.019	2.1233	3.5	1	1	1	1.012	1.206	1.127	0.974	0.858	0.81	0.79	3.40E-02
cds.CL3478Contig1_m.24459	PREDICTED: protein SGT1 homolog B [Theobroma cacao]	40.61	30.532	21.8	9	15	8	1.04	0.986	0.973	1.004	1.038	1.006	1.016	5.13E-01
cds.CL3510Contig1_m.34721	PREDICTED: dnaJ protein homolog 2-like [Nelumbo nucifera]	39.427	1.8909	5.9	2	2	1	1.165	1.153	1.124	0.868	0.793	0.855	0.731	4.63E-04
cds.CL3522Contig1_m.48014	late embryogenesis abundant protein Lea5 [Citrus sinensis]	13.474	5.6271	13.7	2	2	2	1.092	1.116	1.143	0.855	0.868	0.898	0.782	2.37E-04

cds.CL3528Contig1_m.20155	plant/F10M23-360 protein [Medicago truncatula]	60.031	16.251	8.2	5	6	5	1.039	1.052	1.065	0.959	0.959	0.935	0.904	7.99E-04
cds.CL353Contig1_m.27496	PREDICTED: UDP-glycosyltransferase 87A1 [Vitis vinifera]	52.028	4.2687	6.9	2	2	2	0.554		1.451	1.283	0.7	1.117	1.031	8.14E-01
cds.CL3548Contig1_m.10891	PREDICTED: KH domain-containing protein At4g18375 [Vitis vinifera]	51.606	3.4907	2.8	1	1	1	1.087	1.152	1.143	0.864	0.838	0.885	0.765	3.76E-04
cds.CL3554Contig1_m.16489	PREDICTED: coatomer subunit delta [Vitis vinifera]	58.46	63.359	22.1	11	20	9	1.03	1.019	1.036	0.973	0.964	0.965	0.941	4.20E-04
cds.CL357Contig1_m.20184	PREDICTED: serine hydroxymethyltransferase, mitochondrial [Nicotiana attenuata]	57.213	181.09	41.6	18	71	14	1.01	1.016	1.011	0.977	0.996	0.981	0.973	1.07E-02

cds.CL36 Contig2_m .31756	PREDICTED: ras-related protein RABE1a-like [Tarenaya hassleriana]	24.068	71.755	43.8	10	22	3	0.974	0.937	0.939	1.12	1.031	1.05	1.123	1.46E-02
cds.CL379 Contig1_m .39933	PREDICTED: alpha-glucosidase 2 [Prunus	22.427	13.567	14.2	2	3	2	1.006	1.033	0.948	1.068	0.977	0.979	1.012	7.71E-01
cds.CL389 Contig1_m .20104	PREDICTED: autophagy-related protein 18a isoform X1 [Nelumbo nucifera]	42.791	2.5172	2.6	1	1	1								
cds.CL38 Contig5_m .23750	PREDICTED: aspartate--tRNA ligase, chloroplastic/mitochondrial [Vitis vinifera]	27.953	27.166	24	5	6	5	0.961	0.925	0.961	1.067	1.087	1.069	1.132	9.00E-04

cds.CL423 Contig1_m .10274	PREDICTED: heat shock 70 kDa protein 6, chloroplastic [Gossypium arboreum]	75.705	323.31	54.5	39	128	22	1.053	1.011	1.022	0.963	0.958	0.971	0.937	7.14E-03
cds.CL42 Contig3_m .9636	PREDICTED: protein RAE1 [Ricinus communis]	46.158	10.582	11.3	4	4	4	1.025	0.971	0.954	0.984	1.037	1.059	1.044	2.33E-01
cds.CL449 Contig1_m .2878	PREDICTED: topless-related protein 4-like [Nicotiana tabacum]	68.994	2.0319	4.9	3	4	1	1.009	0.962	1.008	0.978	1.079	0.976	1.018	6.65E-01
cds.CL449 Contig1_m .2879	PREDICTED: topless-related protein 4 isoform X1 [Solanum tuberosum	59.834	13.964	9.9	4	4	3	1.026	0.99	1.011	1.029	0.951	0.999	0.984	5.51E-01

cds.CL45 Contig1_m .14786	PREDICTED: DEAD-box ATP-dependent RNA helicase 56-like isoform X2 [Jatropha curcas]	39.04	39.163	30.2	10	21	1	1.105	1.044	1.051	0.923	0.918	0.941	0.869	1.98E-03
cds.CL45 Contig3_m .16586	PREDICTED: DEAD-box ATP-dependent RNA helicase 56-like isoform X2 [Jatropha curcas]	39.081	-2	30.2	10	21	1	1.102	1.044	1.026	0.901	0.951	0.958	0.886	1.39E-02
cds.CL471 Contig1_m .7053	PREDICTED: U-box domain-containing protein 44-like [Daucus carota subsp. sativus]	13.256	1.7795	7.8	1	1	1								

cds.CL488 Contig1_m .3995	PREDICTED: calcium-transporting ATPase 4, endoplasmic reticulum-type [Vitis vinifera]	116.63	187.78	21.9	20	34	17	1.028	1.034	1.027	0.947	0.992	0.96	0.938	4.47E-02
cds.CL494 Contig1_m .6764	PREDICTED: GBF-interacting protein 1-like isoform X1 [Vitis vinifera]	83.811	1.3491	1	1	1	1	1.09	1.129	1.162	0.857	0.862	0.87	0.766	1.45E-04
cds.CL500 Contig1_m .7530	PREDICTED: glucose-6-phosphate 1-dehydrogenase, cytoplasmic isoform [Citrus sinensis]	59.16	90.935	33.1	16	31	15	0.992	1.004	0.971	1.04	0.991	0.993	1.019	3.66E-01
cds.CL503 Contig1_m .27374	PREDICTED: cellulose synthase-like protein G3 [Sesamum indicum]	51.484	1.3148	2.6	1	1	1								

cds.CL504Contig1_m.33268	PREDICTED: putative quinone-oxidoreductase homolog, chloroplastic [Populus euphratica]	34.622	24.52	29.2	9	21	9	0.892	0.942	0.98	1.058	1.066	1.055	1.13	4.66E-02
cds.CL520Contig1_m.30441	PREDICTED: ATPase GET3 [Sesamum indicum]	38.279	26.525	10	3	6	3	0.995	0.99	1.022	1.019	0.986	1.03	1.009	6.06E-01
cds.CL52Contig2_m.22591	PREDICTED: E3 ubiquitin-protein ligase RGLG2 [Vitis vinifera]	27.973	8.8601	8.7	1	1	1	1.061	1.129	0.819	1.055	1.04	0.9	0.995	9.85E-01
cds.CL539Contig1_m.56311	PREDICTED: universal stress protein A-like protein [Ziziphus jujuba]	13.724	13.932	37.6	3	5	3	0.91	0.897	0.856	1.058	1.084	1.146	1.235	2.16E-03

cds.CL546 Contig1_m .8418	PREDICTED: cell division cycle protein 48 homolog [Jatropha curcas]	91.241	298.93	49.5	34	84	6	1.051	1.056	1.075	0.927	0.934	0.938	0.88	7.79E-05
cds.CL546 Contig2_m .7929	PREDICTED: cell division cycle protein 48 homolog [Gossypium hirsutum]	89.542	84.355	43.1	30	70	2	1.061	1.119	1.115	1.063	0.922	0.838	0.857	8.91E-02
cds.CL563 Contig1_m .8180	PREDICTED: probable cytosolic oligopeptidase A [Nicotiana attenuata]	91.055	82.411	26.3	21	32	21	0.96	0.969	0.975	1.063	1.054	1.026	1.082	2.42E-03
cds.CL568 Contig1_m .38094	PREDICTED: ADP,ATP carrier protein 1, mitochondrial-like [Musa acuminata subsp. malaccensis]	24.335	14.379	27.2	6	28	2	1.045	1.066	1.02	0.915	0.904	1.039	0.913	1.15E-01

cds.CL624 Contig1_m .19144	PREDICTED: glyceraldehyde-3-phosphate dehydrogenase GAPCP2, chloroplastic-like [Nelumbo nucifera]	44.8	21.881	15.3	6	15	4	1.128	1.053	1.063	0.916	0.916	0.929	0.851	1.88E-03
cds.CL627 Contig1_m .6284	PREDICTED: probable inactive ATP-dependent zinc metalloprotease FTSHI 4, chloroplastic [Gossypium hirsutum]	96.864	11.934	7.1	7	8	7	0.985	0.997	1.025	1.02	0.997	0.989	1	9.86E-01
cds.CL646 Contig1_m .14438	PREDICTED: WD-40 repeat-containing protein MSI1 [Sesamum indicum]	48.38	31.607	13.7	5	7	5	0.926	1.044	0.967	1.042	0.98	1.062	1.05	3.10E-01
cds.CL663 Contig1_m .36704	PREDICTED: nigrin b-like [Malus domestica]	33.44	4.5206	9.4	3	3	3	0.987	0.959	0.942	1.031	1.048	1.052	1.084	5.62E-03

cds.CL670 Contig1_m .41479	PREDICTED: 1-aminocyclopropane-1-carboxylate oxidase homolog 1 [Vitis vinifera]	28.5	35.266	18.9	3	6	3	1.032	1.029	1.123	0.927	1.024	0.982	0.921	1.14E-01
cds.CL677 Contig1_m .26553	PREDICTED: FAM10 family protein At4g22670-like [Nicotiana tabacum]	42.365	19.308	17.9	8	8	8	1.096	1.048	0.987	0.883	0.914	0.983	0.888	5.33E-02
cds.CL680 Contig1_m .5310	PLAC8 family protein [Medicago truncatula]	28.559	2.2328	6.6	2	2	2	0.874	0.889	0.862	1.16	1.095	1.172	1.306	3.18E-04
cds.CL685 Contig1_m .9727	PREDICTED: probable serine/threonine-protein kinase At5g41260 [Ipomoea	57.737	6.8604	7.6	5	5	3	0.897	0.924	0.901	1.196	1.01	1.117	1.221	1.69E-02

cds.CL686Contig1_m.20439	PREDICTED: putative RNA-binding protein Luc7-like 2 isoform X5 [Nelumbo nucifera]	33.344	6.3235	8.6	3	3	3	1.103	1.049	1.05	0.936	0.908	0.92	0.863	1.44E-03
cds.CL6Contig1_m.11214	PREDICTED: G-type lectin S-receptor-like serine/threonine-protein kinase At4g27290 [Vitis vinifera]	91.978	2.7721	1.3	1	1	1	0.995	0.987	0.947	0.981	1.102	1.002	1.053	2.61E-01
cds.CL708Contig1_m.16547	PREDICTED: apoptosis inhibitor 5 [Sesamum indicum]	34.799	9.9676	20.4	7	8	7	1.013	1.007	0.991	1.006	1.001	0.992	0.996	6.35E-01
cds.CL708Contig1_m.16548	PREDICTED: apoptosis inhibitor 5-like protein API5 isoform X1 [Vitis vinifera]	31.314	37.299	28.3	7	9	7	1.037	1.02	0.984	0.974	0.963	0.99	0.963	9.47E-02

cds.CL709 Contig1_m .3289	PREDICTED: probable alpha-mannosidase At5g13980 isoform X1 [Nicotiana attenuata]	115.27	254.14	28	27	70	25	0.973	0.987	0.993	1.024	1.012	1.009	1.031	1.51E-02
cds.CL740 Contig1_m .21417	PREDICTED: acyl-coenzyme A oxidase 4, peroxisomal isoform X2 [Vitis vinifera]	53.939	6.8311	22.8	11	15	2								
cds.CL74 Contig3_m .5858	PREDICTED: K(+) efflux antiporter 2, chloroplastic [Malus domestica]	42.401	13.099	11.2	5	7	5	1.089	1.023	1.11	0.918	0.92	0.911	0.853	2.38E-02
cds.CL763 Contig1_m .5271	PREDICTED: ATP-dependent zinc metalloprotease FTSH 4, mitochondrial [Vitis vinifera]	45.041	11.704	10.2	4	4	2	0.999	0.996	0.981	1.06	0.971	1.006	1.02	4.93E-01

cds.CL772Contig1_m.15228	PREDICTED: glutamate-1-semialdehyde 2,1-aminomutase 2, chloroplastic [Citrus sinensis]	50.993	137.82	37.5	16	39	16	1.069	1.05	1.057	0.909	0.949	0.947	0.883	1.14E-03
cds.CL776Contig1_m.41420	PREDICTED: glycerophosphodiester phosphodiesterase GDPD1, chloroplastic-like [Pyrus x bretschneideri]	18.072	11.28	29.8	5	8	5	1.104	1.06	1.117	0.876	0.901	0.911	0.819	5.39E-04
cds.CL782Contig1_m.4507	PREDICTED: importin subunit beta-1 [Vitis vinifera]	96.404	15.599	9.6	6	10	3	0.985	1.032	1.026	1.014	0.939	1.01	0.974	4.03E-01

cds.CL798 Contig1_m .5776	PREDICTED: ATP-dependent Clp protease ATP-binding subunit ClpA homolog CD4B, chloroplastic-like [Nicotiana tabacum]	104.65	323.31	46.7	43	138	42	1.024	1.021	1.017	0.98	0.987	0.977	0.961	4.02E-04
cds.CL817 Contig1_m .5545	PREDICTED: cytosolic Fe-S cluster assembly factor NBP35-like [Sesamum indicum]	37.852	4.2373	4.5	2	2	2	0.964	1.003	0.959	0.977	0.992	1.117	1.055	3.12E-01
cds.CL83 Contig1_m .15259	PREDICTED: tubulin alpha chain [Ziziphus jujuba]	49.677	103.8	32	12	24	2	1.164	1.213	1.213	0.788	0.835	0.757	0.663	2.00E-04

cds.CL883Contig1_m.18608	PREDICTED: sphingosine kinase 2-like isoform X3 [Juglans regia]	22.618	1.7371	6.4	1	2	1	0.901	0.964	0.955	1.053	1.101	1.059	1.139	6.72E-03
cds.CL883Contig1_m.18609	PREDICTED: sphingosine kinase 1 isoform X4 [Jatropha curcas]	20.216	4.4335	8.1	1	1	1	1.007	0.937	1.067	1.069	0.958	0.975	0.997	9.59E-01
cds.CL89Contig1_m.16639	PREDICTED: phosphoenolpyruvate carboxylase [Theobroma cacao]	47.931	55.278	47.5	22	47	5	1.048	1.056	1.052	0.963	0.892	0.914	0.877	2.86E-02
cds.CL89Contig1_m.16640	PREDICTED: phosphoenolpyruvate carboxylase, housekeeping isozyme [Juglans	21.338	14.112	37.9	7	15	2	0.907	1.147	1.223	0.962	0.761	0.997	0.83	2.10E-01

cds.CL905Contig1_m.52008	NAD(P)-binding Rossmann-fold superfamily protein [Arabidopsis thaliana]	13.125	9.1757	27.6	3	3	3	1.086	1.043	0.975	0.938	0.914	1.012	0.923	1.41E-01
cds.CL976Contig1_m.11581	PREDICTED: apoptotic chromatin condensation inducer in the nucleus isoform X2 [Vitis vinifera]	65.388	52.01	24.6	12	14	12	1.104	1.085	1.119	0.877	0.879	0.881	0.797	1.58E-03
cds.CL977Contig1_m.7693	PREDICTED: LETM1 and EF-hand domain-containing protein 1, mitochondrial isoform X1 [Theobrom	85.465	30.62	11.9	8	10	8	1.128	1.056	1.129	0.848	0.909	0.936	0.813	4.82E-03

cds.CL988Contig1_m.34155	PREDICTED: non-functional NADPH-dependent codeinone reductase 2-like [Jatropha curcas]	42.05	5.5254	6.2	2	4	2	0.975	1.074	1.065	1.005	0.946	0.958	0.934	1.34E-01
cds.CL99Contig2_m.1015	PREDICTED: glutamate synthase 1 [NADH], chloroplastic isoform X1 [Vitis vinifera]	122.3	4.3976	5.1	5	5	2	0.864	1.014	0.914	0.98	1.165	1.096	1.161	9.73E-02
cds.Camellia_sinensis.comp10002_c0_seq1_m.13184	PREDICTED: histone deacetylase 5 [Theobroma cacao]	56.377	10.34	5.4	2	2	2	1.051	1.029	1.025	1.018	0.936	0.94	0.932	6.66E-02
cds.Camellia_sinensis.comp10009_c0_seq1_m.34659	PREDICTED: UPF0587 protein C1orf123 homolog [Vitis vinifera]	21.445	9.3395	22	4	4	4	0.907	0.937	0.868	1.046	1.156	1.123	1.226	5.37E-03

cds.Camellia_sinensis.comp10027_c0_seq1_m.52968	PREDICTED: pyruvate dehydrogenase E1 component subunit alpha-3, chloroplastic isoform X1 [Citrus sinensis]	17.263	1.8233	8.2	1	1	1								
cds.Camellia_sinensis.comp10031_c0_seq1_m.34320	PREDICTED: probable pyridoxal 5'-phosphate synthase subunit PDX2 isoform X1 [Pyrus x bretschneideri]	27.039	52.302	21.4	3	3	3	0.956	1.012	1.136	0.922	1.012	0.967	0.935	3.16E-01
cds.Camellia_sinensis.comp10045_c0_seq1_m.58892	PREDICTED: calcium-transporting ATPase 4, endoplasmic reticulum-type [Daucus carota subsp. sativus]	11.199	6.5161	31.1	3	4	2	0.966	0.947	0.985	1.028	1.043	1.051	1.077	4.68E-03

cds.Camellia_sinensis.comp10076_c0_seq1_m.41904	PREDICTED: mitochondrial import inner membrane translocase subunit tim16 [Nelumbo nucifera]	12.913	6.0369	24.8	2	2	2	0.83	0.963	1.007	1.094	1.085	1.066	1.159	1.25E-01
cds.Camellia_sinensis.comp10119_c0_seq1_m.18571	PREDICTED: alanine--glyoxylate aminotransferase 2 homolog 1, mitochondrial [Nelumbo	50.652	6.5426	7.5	3	3	3	1.011	1.102	1.095	0.878	0.921	0.982	0.867	2.84E-02
cds.Camellia_sinensis.comp10251_c0_seq1_m.35387	PREDICTED: ras-related protein Rab7-like [Juglans regia]	23.156	69.963	55.8	9	19	4	0.995	0.998	0.957	1.024	1.011	1.052	1.046	6.39E-02
cds.Camellia_sinensis.comp10277_c0_seq2_m.42068	PREDICTED: thioredoxin-like 3-2, chloroplastic isoform X1 [Ziziphus jujuba]	21.499	3.4925	10.5	2	3	1	0.984	0.968	0.951	1.017	1.042	1.055	1.073	8.62E-03

cds.Camellia_sinensis.comp103677_c0_seq1_m.50947	PREDICTED: E3 ubiquitin-protein ligase RGLG2 [Prunus mume]	20.388	1.4193	3.9	1	1	1	0.895	1.345	0.937	0.965	0.945	0.918	0.89	5.23E-01
cds.Camellia_sinensis.comp103776_c0_seq1_m.58345	PREDICTED: chitotriosidase-1 [Theobroma cacao]	11.646	21.655	15	1	2	1	0.882	0.868	0.809	1.12	1.157	1.215	1.365	9.41E-04
cds.Camellia_sinensis.comp104279_c0_seq1_m.44387	PREDICTED: flavin-dependent oxidoreductase FOX3 [Eucalyptus grandis]	27.511	5.1642	12.1	4	8	4	0.914	0.922	0.869	1.099	1.119	1.118	1.233	4.15E-04
cds.Camellia_sinensis.comp10428_c0_seq1_m.47545	PREDICTED: guanylate-binding protein 4-like [Daucus carota subsp. sativus]	25.314	5.9305	29.7	6	8	3	0.839	1.184	0.925	1.031	1.028	1.02	1.044	6.50E-01
cds.Camellia_sinensis.comp10437_c0_seq1_m.48501	PREDICTED: antifungal protein ginkbilobin-2-like [Juglans regia]	14.951	2.4097	13.1	2	2	2								

cds.Camellia_sinensis.comp10466_c0_seq1_m.43937	PREDICTED: non-specific lipid-transfer protein-like 1 isoform X1 [Vitis vinifera]	13.756	10.589	41.1	6	10	6	1.038	1.041	1.047	0.934	0.938	0.948	0.902	3.77E-05
cds.Camellia_sinensis.comp105256_c0_seq1_m.46391	PREDICTED: beta-glucosidase 24-like isoform X2 [Erythranthe guttata]	20.853	5.1094	13	2	3	2								
cds.Camellia_sinensis.comp10550_c0_seq1_m.39041	PREDICTED: 3-hydroxyacyl-[acyl-carrier-protein] dehydratase FabZ-like [Nicotiana tabacum]	25.576	62.654	30	6	16	6	0.993	1.004	1.018	0.985	1.005	1.023	0.999	9.58E-01
cds.Camellia_sinensis.comp10577_c0_seq1_m.58057	PREDICTED: mitochondrial dicarboxylate/tricarboxylate transporter DTC-like [Sesamum indicum]	12.213	6.2969	11.8	1	2	1	1.062	1.038	1.03	0.903	0.958	0.997	0.913	3.86E-02

cds.Camellia_sinensis.comp10582_c0_seq1_m.24052	PREDICTED: pyruvate dehydrogenase E1 component subunit alpha, mitochondrial [Malus domestica]	48.512	80.594	34.9	19	40	19	1.016	1.001	0.996	0.985	0.994	0.986	0.984	7.28E-02
cds.Camellia_sinensis.comp10602_c0_seq1_m.38915	PREDICTED: AT-hook motif nuclear-localized protein 10 [Prunus mume]	14.394	10.531	8.8	1	3	1	1.057	0.935	1.022	0.956	1.026	1.005	0.991	8.57E-01
cds.Camellia_sinensis.comp10604_c0_seq1_m.33368	PREDICTED: phosphoglycolate phosphatase-like [Prunus mume]	30.956	17.021	14.2	3	4	3	0.974	0.945	0.885	1.042	1.076	1.087	1.143	1.24E-02
cds.Camellia_sinensis.comp10649_c0_seq1_m.43648	PREDICTED: phosphoenolpyruvate carboxykinase [ATP] [Ziziphus jujuba]	24.519	4.0395	8.9	2	2	1								

cds.Camellia_sinensis.comp10650_c0_seq1_m.12647	PREDICTED: phosphomethylpyrimidine synthase, chloroplastic-like isoform X3 [Daucus carota subsp. sativus]	71.575	19.171	12.4	7	8	7	0.947	0.934	0.985	1.074	1.019	1.09	1.111	1.56E-02
cds.Camellia_sinensis.comp10670_c0_seq1_m.13958	PREDICTED: phytoene synthase 2, chloroplastic [Vitis vinifera]	47.654	10.005	8.5	3	3	3	0.84	1.058	0.978	1.051	1.168	0.944	1.1	3.54E-01
cds.Camellia_sinensis.comp10679_c0_seq1_m.20913	PREDICTED: hsp70-binding protein 1 [Solanum lycopersicum]	43.71	50.961	16.8	7	13	7	0.974	0.939	0.951	1.072	1.047	1.058	1.109	1.22E-03
cds.Camellia_sinensis.comp10702_c0_seq1_m.27407	PREDICTED: UBP1-associated protein 2A [Daucus carota subsp. sativus]	41.879	1.7058	3.8	2	2	2	1.154	1.093	1.082	0.9	0.894	0.846	0.793	1.16E-03

cds.Camellia_sinensis.comp10716_c0_seq1_m.30794	PREDICTED: lon protease 2-like [Ziziphus jujuba]	32.11	32.236	9.5	2	3	2	0.955	0.916	0.95	1.137	1.058	1.017	1.139	2.15E-02
cds.Camellia_sinensis.comp10740_c0_seq1_m.6969	PREDICTED: uncharacterized aarF domain-containing protein kinase At1g79600, chloroplastic [Jatropha curcas]	86.068	53.07	16.7	12	19	11	0.989	0.976	0.96	1.037	1.065	1.026	1.069	8.80E-03
cds.Camellia_sinensis.comp107685_c0_seq1_m.5269		18.556	2.2212	5.6	1	2	1	0.978	1.006	1.029	1.013	0.984	1.001	0.995	7.89E-01
cds.Camellia_sinensis.comp10794_c0_seq1_m.22161	PREDICTED: BAG family molecular chaperone regulator 7 [Nicotiana sylvestris]	30.069	6.3271	10.6	3	4	3	0.989	1.058	1.07	1.035	0.947	0.909	0.927	1.70E-01

cds.Camellia_sinensis.comp10794_c0_seq1_m.22162	PREDICTED: BAG family molecular chaperone regulator 7-like [Juglans regia]	15.821	12.688	10.8	1	1	1	0.925	0.94	1.249	1.002	0.832	1.063	0.93	6.09E-01
cds.Camellia_sinensis.comp10805_c0_seq1_m.49876	PREDICTED: glycine-rich RNA-binding protein RZ1A-like [Gossypium hirsutum]	17.868	12.882	21.3	3	5	3	1.145	1.153	1.244	0.744	0.842	0.799	0.673	8.95E-04
cds.Camellia_sinensis.comp10829_c0_seq1_m.25743	PREDICTED: AT-hook motif nuclear-localized protein 14 [Vitis vinifera]	39.991	164.91	33	9	11	9	1.024	0.977	0.978	0.98	1.041	1.003	1.015	5.59E-01
cds.Camellia_sinensis.comp10859_c0_seq2_m.23812	PREDICTED: COP9 signalosome complex subunit 3 [Nicotiana	48.594	37.069	18.9	7	9	7	0.982	0.945	1.015	1.018	1.056	1.059	1.065	5.97E-02
cds.Camellia_sinensis.comp10873_c0_seq1_m.42113	PREDICTED: 14-3-3-like protein B [Cicer arietinum]	22.119	34.154	40.7	7	20	5	1.001	0.976	0.974	0.973	1.025	1.027	1.025	2.82E-01

cds.Camellia_sinensis.comp10899_c0_seq1_m.41027	PREDICTED: transmembrane protein 205 [Vigna radiata var. radiata]	24.758	5.8363	5.5	1	4	1	0.991	0.976	1.056	1.038	0.985	0.999	1	1.00E+00
cds.Camellia_sinensis.comp10922_c0_seq1_m.28802	PREDICTED: succinyl-CoA ligase [ADP-forming] subunit alpha-1, mitochondrial [Solanum tuberosum	36.392	31.068	20.6	6	14	6	1.02	0.968	1.05	0.981	0.994	0.983	0.974	3.40E-01
cds.Camellia_sinensis.comp10926_c0_seq1_m.45554	PREDICTED: acyl carrier protein 1, mitochondrial [Sesamum indicum]	18.28	2.3596	5.5	1	2	1	1.066	1.02	1.07	0.905	0.979	0.949	0.898	1.71E-02
cds.Camellia_sinensis.comp10929_c0_seq1_m.22775	PREDICTED: phosphoserine aminotransferase 2, chloroplastic [Vitis vinifera]	47.171	111.85	38.5	13	28	13	0.993	1.043	1.023	1.023	0.962	0.97	0.966	2.21E-01

cds.Camellia_sinensis.comp10930_c0_seq1_m.21645	PREDICTED: glucan endo-1,3-beta-glucosidase [Erythrant	50.098	5.7764	3.9	2	2	2	0.999	1.023	1.061	1.018	0.979	0.927	0.948	1.74E-01
cds.Camellia_sinensis.comp10937_c0_seq2_m.44764	PREDICTED: photosystem II D1 precursor processing protein PSB27-H2, chloroplastic [Ipomoea nil]	15.999	31.45	40.6	6	7	6	1.071	1.075	1.093	0.884	0.932	0.91	0.842	4.77E-04
cds.Camellia_sinensis.comp10941_c0_seq1_m.18623	PREDICTED: aldehyde dehydrogenase family 3 member F1-like [Daucus carota subsp. sativus]	25.483	3.5866	4.8	1	1	1								
cds.Camellia_sinensis.comp10943_c0_seq1_m.42550	PREDICTED: glutaredoxin-C4 [Populus euphratica]	16.37	22.511	15.4	2	2	2	1.098	1.028	0.843	0.976	1.003	1.048	1.02	7.75E-01

cds.Camellia_sinensis.comp11072_c0_seq1_m.44776	PREDICTED: succinate dehydrogenase subunit 6, mitochondrial-like [Solanum tuberosum	19.945	11.896	29.4	4	5	4	1.013	0.982	1.002	1.052	1.058	0.992	1.035	2.04E-01
cds.Camellia_sinensis.comp11086_c0_seq1_m.29628	PREDICTED: calcium sensing receptor, chloroplastic [Citrus sinensis]	43.539	156.22	42.3	16	36	16	0.984	0.992	0.981	1.014	1.018	1.004	1.027	7.60E-03
cds.Camellia_sinensis.comp11136_c0_seq1_m.33507	Proliferating cell nuclear antigen [Morus notabilis]	33.406	14.977	19.1	5	7	5	0.978	0.98	1.008	1.011	1.046	1.02	1.037	5.98E-02
cds.Camellia_sinensis.comp11149_c0_seq1_m.18881	PREDICTED: ammonium transporter 3 member 1-like [Jatropha	52.367	2.0667	3.5	2	2	2	1.166	1.133	1.051	0.91	0.831	0.874	0.781	3.66E-03

cds.Camellia_sinensis.comp11151_c0_seq1_m.35921	PREDICTED: pleiotropic drug resistance protein 2-like, partial [Erythranthe guttata]	40.419	9.4332	13.5	5	6	3	0.931	0.995	0.906	1.079	1.064	1.023	1.118	2.51E-02
cds.Camellia_sinensis.comp11161_c0_seq1_m.44788	PREDICTED: 60S ribosomal protein L4 [Ziziphus jujuba]	28.225	18.139	35.5	8	11	3	1.114	1.147	1.114	0.822	0.84	0.926	0.767	2.14E-03
cds.Camellia_sinensis.comp11194_c0_seq1_m.50433	PREDICTED: disease resistance protein RPM1 [Vitis vinifera]	21.26	1.2005	5.9	1	1	1	0.73	1.04	1.353	1.193	0.899	0.835	0.937	8.38E-01
cds.Camellia_sinensis.comp11219_c0_seq1_m.30122	PREDICTED: peroxisomal 2,4-dienoyl-CoA reductase [Vitis vinifera]	31.846	1.3712	6.1	2	2	2								

cds.Camellia_sinensis.comp11229_c0_seq2_m.47116	PREDICTED: phosphomannomutase/phosphoglucomutase [Prunus mume]	24.123	16.236	20.6	4	6	4	0.999	0.978	0.978	0.99	1.017	1.005	1.019	1.44E-01
cds.Camellia_sinensis.comp11267_c0_seq1_m.29394	PREDICTED: 26S proteasome non-ATPase regulatory subunit 7 homolog A [Nicotiana	34.835	46.161	17.7	5	9	5	1.012	0.97	0.962	1	1.028	1.047	1.044	1.02E-01
cds.Camellia_sinensis.comp11318_c0_seq1_m.52741	PREDICTED: ferredoxin--NADP reductase, leaf isozyme, chloroplastic [Glycine max]	11.816	20.431	70.7	8	29	4	0.978	0.947	0.959	1.021	1.067	1.037	1.084	7.34E-03
cds.Camellia_sinensis.comp11344_c0_seq1_m.52995	PREDICTED: STS14 protein [Vitis vinifera]	16.43	4.0858	13.2	2	2	2	0.995	1.062	1.048	1.073	0.876	0.953	0.935	3.19E-01

cds.Camellia_sinensis.comp11350_c0_seq1_m.32544	PREDICTED: uncharacterized oxidoreductase At4g09670-like [Nicotiana attenuata]	41.58	16.216	9.6	3	6	3	0.958	1.014	1.066	0.942	1.028	1.01	0.981	6.65E-01
cds.Camellia_sinensis.comp11374_c0_seq1_m.28394	PREDICTED: WD-40 repeat-containing protein MSI3 [Vitis vinifera]	46.422	3.9703	6.1	3	3	3	0.872	1.135	0.927	1.111	0.968	1.017	1.055	5.48E-01
cds.Camellia_sinensis.comp11379_c0_seq1_m.29727	PREDICTED: acetylglutamate kinase, chloroplastic [Vitis vinifera]	41.239	15.931	14.3	4	5	4	1.075	1.016	1.002	0.954	0.998	0.916	0.927	8.23E-02
cds.Camellia_sinensis.comp11391_c0_seq1_m.35926	PREDICTED: NADH dehydrogenase [ubiquinone] iron-sulfur protein 8, mitochondrial [Ipomoea nil]	26.285	66.654	35.5	9	18	9	1.02	0.984	1.048	0.98	0.969	0.983	0.961	1.01E-01

cds.Camellia_sinensis.comp11398_c0_seq1_m.34837	PREDICTED: glyoxylate/succinic semialdehyde reductase 1 [Vitis vinifera]	32.042	91.026	44.8	10	26	10	0.995	0.992	1.003	1.017	1.01	1.027	1.021	2.24E-02
cds.Camellia_sinensis.comp11416_c0_seq1_m.21653	PREDICTED: 4-hydroxy-3-methylbut-2-enyl diphosphate reductase, chloroplastic-like [Daucus carota subsp.sativus]	51.808	77.835	40.7	19	34	19	0.991	1.039	1.01	0.997	0.978	0.993	0.976	1.86E-01
cds.Camellia_sinensis.comp11449_c0_seq1_m.31170	PREDICTED: phosphoglycolate phosphatase 1B, chloroplastic [Cucumis sativus]	35.737	145.09	41.5	12	33	12	1.016	1.014	1.016	0.982	0.985	0.972	0.965	9.22E-04

cds.Camellia_sinensis.comp11457_c0_seq1_m.35713	PREDICTED: structural maintenance of chromosomes protein 1A [Sesamum indicum]	19.921	14.027	43	9	10	9	1	0.977	0.991	1.008	1.01	1.01	1.02	9.84E-02
cds.Camellia_sinensis.comp11498_c0_seq1_m.44271	PREDICTED: glucan endo-1,3-beta-glucosidase 12-like [Glycine	27.773	27.131	8.9	2	2	2	0.942	0.927	0.942	1.047	1.084	1.086	1.144	4.97E-04
cds.Camellia_sinensis.comp11527_c0_seq1_m.47902	PREDICTED: molybdopterin synthase sulfur carrier subunit [Ipomoea nil]	11.663	3.8458	9.4	1	1	1	0.978	0.897	0.898	1.065	1.081	1.109	1.174	6.54E-03
cds.Camellia_sinensis.comp11529_c0_seq2_m.48725	PREDICTED: peptidyl-prolyl cis-trans isomerase FKBP12 [Fragaria vesca subsp. vesca]	11.929	44.809	30.4	3	13	3	0.995	0.987	1.011	1	1.011	1.018	1.012	2.43E-01

cds.Camellia_sinensis.comp11557_c0_seq1_m.4291	PREDICTED: probable glutamyl endopeptidase, chloroplastic isoform X2 [Vitis vinifera]	110.49	119.62	22.3	16	29	16	1.003	1.041	1.014	0.967	0.961	0.992	0.955	3.49E-02
cds.Camellia_sinensis.comp11579_c0_seq1_m.31962	PREDICTED: 3-hydroxybutyryl-CoA dehydrogenase [Prunus mume]	31.825	26.559	9.8	3	3	3	0.929	0.941	0.979	1.043	1.044	1.09	1.115	7.08E-03
cds.Camellia_sinensis.comp11588_c0_seq1_m.34643	PREDICTED: peptide methionine sulfoxide reductase B1, chloroplastic [Ziziphus jujuba]	21.568	15.256	14.9	3	3	3	1.03	0.963	1.013	0.97	1.009	1.019	0.997	9.26E-01
cds.Camellia_sinensis.comp11591_c0_seq1_m.53844	PREDICTED: catalase isozyme 1 [Vitis vinifera]	17.178	35.749	40.9	5	20	1	0.931	0.982	0.865	0.961	1.093	1.13	1.146	9.29E-02

cds.Camellia_sinensis.comp11597_c0_seq1_m.23792	PREDICTED: putative MO25-like protein At5g47540 [Vitis vinifera]	39.236	9.8383	18.8	7	13	7	0.927	0.952	0.966	1.056	1.052	1.021	1.1	4.10E-03
cds.Camellia_sinensis.comp11628_c0_seq1_m.34499	PREDICTED: vacuolar protein sorting-associated protein 28 homolog 1 [Populus euphratica]	23.354	3.7567	12	2	2	2	0.853	1.064	1.016	1.151	1.022	0.933	1.059	5.57E-01
cds.Camellia_sinensis.comp11639_c0_seq1_m.49146	PREDICTED: probable plastid-lipid-associated protein 4, chloroplastic isoform X2 [Vitis vinifera]	19.698	4.8786	9.1	2	6	2	1.043	1.022	0.971	0.928	1.059	0.931	0.961	4.44E-01
cds.Camellia_sinensis.comp11661_c0_seq2_m.16778	PREDICTED: protochlorophyllide-dependent translocon component 52, chloroplastic [Vitis vinifera]	66.107	9.7587	9.8	7	8	6	1.02	1.015	1.002	1.033	0.982	0.953	0.977	3.85E-01

cds.Camellia_sinensis.comp11716_c0_seq1_m.37295	PREDICTED: 40S ribosomal protein S7 [Nelumbo nucifera]	26.673	10.971	19.4	5	8	5	1.188	1.23	1.177	0.751	0.773	0.773	0.639	1.55E-05
cds.Camellia_sinensis.comp11738_c0_seq1_m.37320	PREDICTED: outer envelope pore protein 16, chloroplastic [Nicotiana tomentosiformis]	15.6	94.818	39	5	24	5	1.008	0.989	0.986	1.01	1.047	0.989	1.021	3.15E-01
cds.Camellia_sinensis.comp117518_c0_seq1_m.43727	PREDICTED: cytochrome P450 87A3-like [Juglans regia]	26.7	1.7493	4.7	2	2	2	0.865	0.824	0.852	1.094	1.164	1.254	1.382	1.54E-03
cds.Camellia_sinensis.comp11764_c1_seq1_m.32328	PREDICTED: short-chain dehydrogenase TIC 32, chloroplastic [Vitis vinifera]	33.759	159.67	55.8	13	40	13	1.03	1.011	1.031	0.975	0.99	0.966	0.954	8.02E-03
cds.Camellia_sinensis.comp11800_c0_seq1_m.29878	PREDICTED: glutathione S-transferase L3 [Vitis vinifera]	31.872	156.82	47	15	64	14	0.949	0.908	0.925	1.062	1.106	1.085	1.169	8.40E-04

cds.Camellia_sinensis.comp11805_c0_seq1_m.42615	profilin-1 [Jatropha curcas]	14.199	2.6814	24.4	3	10	2	0.977	0.884	1.086	1.193	0.967	0.923	1.046	6.91E-01
cds.Camellia_sinensis.comp11843_c0_seq1_m.25706	PREDICTED: malate dehydrogenase, mitochondrial-like [Nelumbo nucifera]	36.749	181.98	40.1	13	35	12	1.028	1.007	1.027	0.944	0.976	1.009	0.957	9.30E-02
cds.Camellia_sinensis.comp11884_c0_seq1_m.37327	PREDICTED: OTU domain-containing protein At3g57810 [Jatropha curcas]	26.012	4.7359	4.3	1	1	1	1.148	1.042	1.194	0.805	0.911	0.865	0.763	7.48E-03
cds.Camellia_sinensis.comp11900_c0_seq1_m.29683	PREDICTED: peroxidase 4 [Citrus sinensis]	35.543	92.403	36.8	9	22	8	1.125	1.091	1.049	0.908	0.876	0.901	0.822	1.06E-03
cds.Camellia_sinensis.comp11934_c0_seq1_m.57022	PREDICTED: heat shock cognate 70 kDa protein 2-like [Erythranthe guttata]	12.911	8.5621	53.8	5	23	1	1.065	1.041	0.973	0.93	1.02	0.964	0.946	2.23E-01

cds.Camellia_sinensis.comp11945_c0_seq1_m.33686	PREDICTED: stem-specific protein TSJT1-like [Sesamum indicum]	25.441	18.713	14.4	3	7	3	0.924	0.963	0.886	1.078	1.104	1.105	1.185	2.56E-03
cds.Camellia_sinensis.comp11995_c0_seq1_m.39460	PREDICTED: small ubiquitin-related modifier 1-like [Fragaria vesca subsp. vesca]	13.624	4.9855	29.4	4	9	1	1.017	0.921	1.125	0.943	1.032	0.968	0.961	5.89E-01
cds.Camellia_sinensis.comp12026_c0_seq1_m.47397	PREDICTED: photosystem I reaction center subunit psaK, chloroplastic [Vitis vinifera]	13.451	2.7599	12.2	2	5	2	1.006	1.01	0.984	0.991	0.985	1.046	1.007	7.53E-01
cds.Camellia_sinensis.comp12032_c0_seq1_m.38308	PREDICTED: probable histone H2A.1 [Nicotiana attenuata]	13.993	19.493	33.6	6	16	3	1.184	1.141	1.088	0.827	0.805	0.938	0.753	5.80E-03

cds.Camellia_sinensis.comp12035_c0_seq1_m.47225	PREDICTED: photosystem I reaction center subunit V, chloroplastic [Vitis vinifera]	15.891	12.339	17.8	3	4	3	0.928	0.99	0.985	1.007	1.065	0.998	1.058	1.27E-01
cds.Camellia_sinensis.comp12046_c0_seq1_m.35841	PREDICTED: HMG1/2-like protein isoform X2 [Vitis vinifera]	24.621	4.1536	12.7	3	3	2	0.919	1.002	1.037	0.971	0.999	1.089	1.034	5.36E-01
cds.Camellia_sinensis.comp12057_c0_seq1_m.27251	cysteine protease RD19A-like [Cajanus cajan]	45.032	16.485	14.4	6	12	4	1.053	1.02	1.001	0.928	1.001	0.981	0.947	1.11E-01
cds.Camellia_sinensis.comp12070_c0_seq1_m.37627	PREDICTED: protein CURVATURE THYLAKOID 1B, chloroplastic [Juglans regia]	18.713	7.7143	13.6	4	10	4	1.046	1.002	1.046	0.984	0.985	0.964	0.948	2.88E-02

cds.Camellia_sinensis.comp12084_c0_seq1_m.30795	PREDICTED: probable carboxylesterase 5 [Juglans regia]	33.135	145.62	56.2	15	57	15	1.047	1.06	1.045	0.954	0.93	0.953	0.9	3.64E-04
cds.Camellia_sinensis.comp12137_c0_seq1_m.17932	PREDICTED: glyceraldehyde-3-phosphate dehydrogenase A, chloroplastic [Nelumbo nucifera]	43.261	323.31	49.1	19	127	16	0.995	1	1.002	1.003	0.989	1.004	1	9.50E-01
cds.Camellia_sinensis.comp12164_c0_seq1_m.26995	PREDICTED: leucoanthocyanidin reductase [Theobroma cacao]	37.588	1.2298	4.1	2	2	2	1.429	1.296	1.581	0.591	0.449	0.524	0.363	4.95E-04
cds.Camellia_sinensis.comp12185_c0_seq1_m.39884	PREDICTED: stress-response A/B barrel domain-containing protein At5g22580 [Theobrom	12.035	9.14	35.8	3	4	3	0.968	0.924	0.998	1.026	1.11	0.99	1.082	1.27E-01

cds.Camellia_sinensis.comp12192_c0_seq1_m.22171	PREDICTED: fructose-bisphosphate aldolase 1, chloroplastic [Solanum pennellii]	42.925	323.31	63.2	21	101	20	1.051	1.044	1.042	0.93	0.957	0.938	0.901	2.97E-04
cds.Camellia_sinensis.comp12208_c0_seq1_m.38668	PREDICTED: photosystem I reaction center subunit N, chloroplastic-like [Gossypium hirsutum]	18.763	55.291	33.9	6	30	6	0.99	0.989	1.004	1.013	1.004	0.983	1.006	6.09E-01
cds.Camellia_sinensis.comp12243_c0_seq1_m.43261	PREDICTED: photosystem II repair protein PSB27-H1, chloroplastic [Solanum pennellii]	21.983	80.639	23.3	6	28	6	1.021	1.019	0.988	1.004	1.007	0.998	0.994	6.03E-01

cds.Camellia_sinensis.comp12293_c0_seq1_m.10889	RUBISCO SUBUNIT BINDING-protein ALPHA SUBUNIT [Populus trichocarpa]	62.205	323.31	42.5	22	74	21	1.025	0.988	0.994	1.007	1.016	0.987	1.001	9.45E-01
cds.Camellia_sinensis.comp12317_c0_seq1_m.25908	PREDICTED: probable fructokinase-4 [Vitis vinifera]	34.089	90.402	44.3	11	25	11	1.037	1.107	1.042	0.929	0.936	0.924	0.875	3.42E-03
cds.Camellia_sinensis.comp12370_c1_seq10_c1_seq1_m.15160	PREDICTED: patellin-3-like [Gossypium arboreum]	64.688	110.89	43.4	21	36	20	0.871	0.863	0.889	1.115	1.153	1.163	1.308	6.31E-05
cds.Camellia_sinensis.comp12396_c0_seq1_m.16096	PREDICTED: T-complex protein 1 subunit beta [Ziziphus jujuba]	57.154	181.76	45.9	21	40	21	0.941	0.969	0.922	1.057	1.048	1.064	1.119	1.72E-03

cds.Camellia_sinensis.comp12441_c0_seq1_m.14154	PREDICTED: bifunctional 3-dehydroquinate dehydratase/shikimate dehydrogenase, chloroplastic [Vitis vinifera]	58.235	113.88	32.7	17	30	16	1.237	1.275	1.282	0.707	0.716	0.69	0.557	1.48E-06
cds.Camellia_sinensis.comp12442_c0_seq1_m.43097	PREDICTED: probable xyloglucan endotransglucosylase/hydrolase protein 7 [Prunus mume]	31.109	10.601	22.1	4	7	2	0.993	0.836	1.026	1.15	1.073	0.955	1.113	2.65E-01
cds.Camellia_sinensis.comp12443_c0_seq1_m.27897	PREDICTED: chloroplast stem-loop binding protein of 41 kDa a, chloroplastic-like [Populus euphratica]	43.629	157.32	44.8	17	54	17	1.04	1.023	1.036	0.954	0.979	0.968	0.936	1.82E-03

cds.Camellia_sinensis.comp12477_c0_seq1_m.17378	PREDICTED: glucan endo-1,3-beta-glucosidase 7-like [Jatropha	28.881	3.5986	7.4	2	3	1								
cds.Camellia_sinensis.comp12500_c0_seq1_m.21591	PREDICTED: serine carboxypeptidase-like 7 [Vitis vinifera]	54.522	17.149	11.5	5	5	5	1.056	1.042	1.002	0.927	0.993	0.925	0.918	3.75E-02
cds.Camellia_sinensis.comp12573_c0_seq1_m.29104	PREDICTED: transcription factor VIP1 [Vitis vinifera]	37.75	7.2577	7.9	3	6	3	1.038	1.032	1.055	0.978	0.941	0.989	0.931	1.18E-02
cds.Camellia_sinensis.comp12605_c0_seq1_m.52149	PREDICTED: coatomer subunit delta [Prunus mume]	13.687	2.1048	17.6	2	2	1	1.079	0.987	1.028	0.9	0.983	1.012	0.936	2.00E-01
cds.Camellia_sinensis.comp12611_c0_seq1_m.30871	PREDICTED: protein BOLA4, chloroplastic/mitochondrial [Ricinus communis]	20.032	29.401	17.2	2	3	2	1.015	1.083	0.97	0.987	0.953	0.985	0.953	2.40E-01

cds.Camellia_sinensis.comp12679_c0_seq1_m.35855	PREDICTED: aquaporin TIP1-3 [Eucalyptus grandis]	26.001	3.5046	6.3	2	3	2	0.853	0.798	0.853	1.104	1.169	1.278	1.418	1.92E-03
cds.Camellia_sinensis.comp12682_c0_seq1_m.34284	Rieske (2Fe-2S) domain protein [Medicago truncatula]	31.181	224.77	48.3	11	44	11	1.026	1.037	1.043	0.971	0.963	0.964	0.933	2.22E-04
cds.Camellia_sinensis.comp12697_c0_seq1_m.39056	F1N21.17 [Arabidopsis lyrata subsp. lyrata]	11.838	26.808	47.5	3	5	3	1.014	1.076	1.038	0.947	0.933	0.982	0.915	1.81E-02
cds.Camellia_sinensis.comp12720_c0_seq1_m.56493	PREDICTED: nitronate monooxygenase [Daucus carota subsp. sativus]	12.511	7.9073	9.5	1	1	1	0.901	0.976	0.902	1.143	1.081	1.038	1.174	1.41E-02
cds.Camellia_sinensis.comp12723_c0_seq1_m.32948	PREDICTED: cytochrome c oxidase subunit 6b-1 [Sesamum indicum]	19.951	8.4083	22.7	4	8	4	0.993	1.029	1.043	0.98	0.964	0.94	0.941	3.30E-02

cds.Camellia_sinensis.comp12739_c0_seq1_m.34624	PREDICTED: 40S ribosomal protein S3-3-like [Nelumbo nucifera]	21.394	39.616	73.4	12	20	7	0.99	0.995	0.983	1.043	1.047	1.002	1.042	4.82E-02
cds.Camellia_sinensis.comp12750_c0_seq1_m.38253	PREDICTED: transmembrane emp24 domain-containing protein p24delta3 [Vitis vinifera]	24.849	4.1806	11.4	3	4	3	0.969	0.952	0.955	1.047	1.036	0.997	1.071	1.25E-02
cds.Camellia_sinensis.comp12764_c0_seq1_m.17074	PREDICTED: vegetative cell wall protein gp1 [Capsicum annuum]	13.5	17.706	30.5	4	8	4	1.017	0.994	1.008	0.982	0.979	0.983	0.975	2.07E-02
cds.Camellia_sinensis.comp12781_c0_seq1_m.25470	PREDICTED: dihydroflavonol-4-reductase-like [Daucus carota subsp. sativus]	38.694	20.965	19.6	9	16	9	1.433	1.386	1.417	0.53	0.54	0.534	0.379	5.31E-08

cds.Camellia_sinensis.comp12805_c0_seq1_m.26397	PREDICTED: cathepsin B-like [Nelumbo nucifera]	45.009	21.051	15	5	11	5	1.045	1.079	1.049	0.938	0.964	0.905	0.885	4.18E-03
cds.Camellia_sinensis.comp12825_c0_seq1_m.44303	PREDICTED: probable prefoldin subunit 5 [Theobroma cacao]	20.62	3.3063	10.8	2	2	2	0.882	0.931	0.846	1.145	1.014	1.226	1.273	1.82E-02
cds.Camellia_sinensis.comp12828_c1_seq1_m.58512	60S ribosomal protein L35a-3 [Morus notabilis]	12.254	1.9256	13.2	2	2	2	1.098	1.204	1.164	0.907	0.783	0.811	0.722	3.22E-03
cds.Camellia_sinensis.comp12830_c0_seq1_m.44966	latex abundant protein 1 [Populus trichocarpa]	17.592	183.19	71.2	10	24	10	1.134	1.077	1.012	0.896	0.94	0.931	0.859	1.36E-02
cds.Camellia_sinensis.comp12874_c0_seq1_m.59108	PREDICTED: probable methionine--tRNA ligase [Daucus carota subsp. sativus]	11.747	1.1922	36.3	3	3	1	1.246	1.051	1.165	0.777	0.91	0.799	0.718	8.84E-03

cds.Camellia_sinensis.comp12954_c0_seq1_m.32734	PREDICTED: histone H1-like isoform X1 [Ipomoea nil]	29.007	11.118	23.9	7	10	7	1.05	1.019	1.07	0.943	0.927	0.966	0.903	5.46E-03
cds.Camellia_sinensis.comp13022_c0_seq1_m.21360	PREDICTED: rhodanese-like domain-containing protein 4, chloroplastic [Prunus mume]	47.668	172.77	34.7	16	47	16	1.003	0.996	1.008	0.981	1.019	0.999	0.997	8.22E-01
cds.Camellia_sinensis.comp13036_c0_seq1_m.55058	PREDICTED: pectinesterase [Ricinus communis]	14.909	7.6807	16.8	2	3	2	0.999	0.994	0.963	0.996	0.996	1.048	1.028	2.45E-01
cds.Camellia_sinensis.comp13044_c0_seq1_m.53290	PREDICTED: putative cytochrome c oxidase subunit 5b-like [Nicotiana tabacum]	13.886	31.738	35.2	4	8	4	1.003	1.09	1.027	1.025	0.947	0.907	0.923	1.36E-01

cds.Camellia_sinensis.comp13050_c0_seq1_m.35139	PREDICTED: methyl-CpG-binding domain-containing protein 11 [Theobroma cacao]	32.049	57.584	35.1	9	17	7	1.174	1.121	1.111	0.804	0.822	0.82	0.718	5.85E-05
cds.Camellia_sinensis.comp13073_c0_seq1_m.39800	PREDICTED: asparagine synthetase [glutamine-hydrolyzing] [Nicotiana tomentosiformis]	26.758	1.7498	6.4	1	1	1								
cds.Camellia_sinensis.comp13077_c0_seq1_m.36053	PREDICTED: disease resistance protein At4g27190-like isoform X3 [Ziziphus jujuba]	41.085	-2	3.3	1	1	1	0.983	0.89	0.939	1.119	1.125	0.976	1.145	7.00E-02

cds.Camellia_sinensis.comp13115_c0_seq1_m.35237	PREDICTED: eukaryotic translation initiation factor NCBP-like [Nelumbo nucifera]	26.529	11.516	21.7	3	3	3	0.661	1.136	0.969	1.3	0.987	1.019	1.195	3.45E-01
cds.Camellia_sinensis.comp13136_c0_seq1_m.26302	PREDICTED: protein COFACTOR ASSEMBLY OF COMPLEX C SUBUNIT B CCB1, chloroplastic [Citrus sinensis]	35.525	21.397	17.4	5	8	5	1.055	1.016	1.019	0.956	0.992	0.99	0.951	4.15E-02
cds.Camellia_sinensis.comp13142_c0_seq1_m.52933	PREDICTED: alcohol dehydrogenase-like, partial [Arachis ipaensis]	16.871	2.0232	7	1	1	1	0.932	1.116	1.089	1.081	0.893	0.902	0.917	3.55E-01

cds.Camellia_sinensis.comp13259_c0_seq1_m.37930	PREDICTED: outer envelope pore protein 24A, chloroplastic-like [Nelumbo nucifera]	28.734	3.8169	3.1	1	1	1	1.01	1.005	0.909	0.986	1.024	1.076	1.055	2.71E-01
cds.Camellia_sinensis.comp13300_c0_seq1_m.40162	PREDICTED: glucan endo-1,3-beta-glucosidase 12 [Vitis	24.224	2.2618	4.7	1	1	1	0.845	0.928	0.913	1.148	1.16	1.06	1.254	5.06E-03
cds.Camellia_sinensis.comp133106_c0_seq1_m.54569	PREDICTED: dammarenediol II synthase-like [Sesamum indicum]	14.114	3.3692	15.2	2	2	2	1.409	1.282	1.25	0.624	0.618	0.707	0.495	2.40E-04
cds.Camellia_sinensis.comp13328_c0_seq1_m.30564	PREDICTED: prohibitin-1, mitochondrial-like [Nicotiana tomentosiformis]	31.89	53.102	37.8	8	14	8	0.991	0.996	0.989	0.98	1.018	1.011	1.011	4.11E-01

cds.Camellia_sinensis.comp13338_c0_seq1_m.40775	PREDICTED: protein SPA, chloroplastic [Prunus mume]	22.737	52.392	11.2	1	1	1	0.88	1.133	1.07	1.045	0.703	1.177	0.949	7.07E-01
cds.Camellia_sinensis.comp13351_c0_seq1_m.35324	PREDICTED: putative glucose-6-phosphate 1-epimerase isoform X2 [Vitis vinifera]	37.453	92.128	52	13	20	8	0.94	0.915	1	1.087	1.099	1.051	1.134	1.29E-02
cds.Camellia_sinensis.comp13361_c0_seq1_m.13837	PREDICTED: polyadenylate-binding protein-interacting protein 4 isoform X1 [Vitis	69.81	17.606	6.6	5	6	5	1.081	1.096	1.01	0.868	0.913	1.012	0.876	6.15E-02
cds.Camellia_sinensis.comp13400_c0_seq1_m.38479	3-demethylubiquinone-9 3-methyltransferase domain protein [Medicago truncatula]	16.924	78.972	65.6	8	22	8	0.912	0.911	0.906	1.111	1.043	1.115	1.198	1.43E-02

cds.Camellia_sinensis.comp13434_c0_seq1_m.32107	PREDICTED: probable signal peptidase complex subunit 2 [Ricinus communis]	26.467	14.892	24.2	5	7	5	0.983	0.939	0.969	1.017	1.062	1.014	1.07	2.86E-02
cds.Camellia_sinensis.comp13435_c0_seq1_m.13067	PREDICTED: asparagine synthetase [glutamine-hydrolyzing] 3 [Juglans regia]	65.75	28.875	16.4	7	10	7	0.961	0.988	1.011	1.032	1.01	1.088	1.057	1.04E-01
cds.Camellia_sinensis.comp13455_c0_seq1_m.37151	ripening regulated protein DDTFR8 [Solanum lycopersicum]	20.784	2.0867	9.7	2	2	2								
cds.Camellia_sinensis.comp13487_c0_seq1_m.27899	PREDICTED: quinone oxidoreductase-like [Nicotiana tabacum]	40.012	85.314	45.9	13	20	13	0.94	0.965	1.021	1.026	1.065	1.029	1.066	7.60E-02

cds.Camellia_sinensis.comp13488_c0_seq1_m.33920	PREDICTED: protein CHAPERONE-LIKE PROTEIN OF POR1, chloroplastic [Vitis vinifera]	37.877	15.687	11	3	4	3	1.044	0.946	1.024	0.926	0.978	1.079	0.99	8.42E-01
cds.Camellia_sinensis.comp1349_c0_seq1_m.56647	PREDICTED: NADH kinase isoform X1 [Ziziphus jujuba]	13.873	1.6191	7.4	1	1	1	0.991	1.035	1.074	0.94	1.066	0.899	0.937	2.98E-01
cds.Camellia_sinensis.comp13521_c0_seq1_m.24653	PREDICTED: plasma membrane ATPase 1-like [Nicotiana tabacum]	56.45	-2	16.7	9	27	1	1.029	0.997	0.96	0.99	0.957	1.07	1.01	8.15E-01
cds.Camellia_sinensis.comp135558_c0_seq1_m.58691	PREDICTED: plasma membrane ATPase 4-like [Gossypium hirsutum]	12.185	3.7779	42.9	4	6	2								

cds.Camellia_sinensis.comp13598_c0_seq1_m.48857	PREDICTED: glycine-rich RNA-binding protein 2, mitochondrial-like [Populus euphratica]	15.038	74.468	27.7	3	5	3	1.014	0.987	0.995	0.971	1.004	1.017	0.999	9.31E-01
cds.Camellia_sinensis.comp13601_c0_seq1_m.43173	PREDICTED: probable prefoldin subunit 4 [Nelumbo nucifera]	14.742	40.049	29.4	3	6	3	0.955	0.977	0.967	1.099	1.029	0.971	1.069	1.46E-01
cds.Camellia_sinensis.comp13626_c0_seq1_m.14698	PREDICTED: pentatricopeptide repeat-containing protein At3g49240 [Solanum	64.039	32.252	18.1	10	17	10	0.979	1.024	0.967	1.008	1.011	0.992	1.014	4.89E-01
cds.Camellia_sinensis.comp13654_c0_seq1_m.41269	PREDICTED: glutaredoxin-like [Juglans regia]	11.293	36.926	37.4	3	5	3	0.936	0.893	0.916	1.067	1.12	1.038	1.175	3.50E-03

cds.Camellia_sinensis.comp13714_c0_seq1_m.40317	PREDICTED: acyl-coenzyme A thioesterase 13 [Jatropha curcas]	18.83	3.3492	6.4	1	1	1	1.022	1.03	1.03	0.963	0.99	0.966	0.947	3.96E-03
cds.Camellia_sinensis.comp13716_c0_seq1_m.40622		16.969	6.2586	16.8	2	2	2	1.132	1.115	1.021	0.833	0.945	0.924	0.827	1.94E-02
cds.Camellia_sinensis.comp13771_c0_seq1_m.36982	transmembrane protein, putative [Medicago truncatula]	30.502	9.6132	12.9	2	3	2	0.992	0.902	0.948	1.015	1.016	1.144	1.117	8.51E-02
cds.Camellia_sinensis.comp13825_c0_seq1_m.4415	PREDICTED: protein FAR1-RELATED SEQUENCE 3 [Vitis	98.894	41.633	16.9	13	15	10	0.969	0.987	0.956	1.069	0.998	1.033	1.065	4.67E-02
cds.Camellia_sinensis.comp13828_c0_seq1_m.31187	PREDICTED: protein usf-like [Gossypium hirsutum]	32.97	125.84	42.4	11	30	11	0.976	0.97	0.966	1.021	1.064	1.029	1.069	6.76E-03

cds.Camellia_sinensis.comp13829_c0_seq1_m.38050	PREDICTED: nucleoside diphosphate kinase 2, chloroplastic isoform X2 [Vitis vinifera]	26.49	35.449	27.8	6	14	6	0.993	1.001	1.009	0.971	0.976	0.988	0.977	2.95E-02
cds.Camellia_sinensis.comp13875_c0_seq1_m.28867	PREDICTED: galactinol synthase 1 [Vitis vinifera]	45.691	119.14	28.5	10	38	10	1.008	0.994	0.992	0.99	1.013	0.992	1	9.74E-01
cds.Camellia_sinensis.comp13898_c0_seq1_m.43751	PREDICTED: auxin-binding protein ABP19a-like [Nelumbo nucifera]	28.03	23.122	8.4	3	13	3	0.937	0.91	0.913	1.079	1.116	1.088	1.189	2.23E-04
cds.Camellia_sinensis.comp13953_c0_seq1_m.37154	SOUL heme-binding family protein [Medicago truncatula]	22.706	12.149	22.4	4	4	4	0.993	0.954	0.986	1.008	0.983	1.052	1.038	1.92E-01

cds.Camellia_sinensis.comp13994_c0_seq1_m.19902	PREDICTED: protein FATTY ACID EXPORT 3, chloroplastic-like isoform X1 [Nicotiana tabacum]	35.682	42.138	25.4	10	18	10	1.039	0.973	0.959	0.974	1.027	1.013	1.014	6.42E-01
cds.Camellia_sinensis.comp14014_c0_seq1_m.31949	PREDICTED: protein argonaute 4A [Vitis vinifera]	40.561	3.6431	14.2	6	8	2	1.039	1.018	1.074	0.964	0.947	0.955	0.915	5.80E-03
cds.Camellia_sinensis.comp14021_c0_seq1_m.42083	PREDICTED: protein LHCP TRANSLOCATION DEFECT [Fragaria vesca subsp. vesca]	19.534	24.398	17.2	3	5	3	1.111	1.082	1.033	0.88	0.883	0.955	0.843	7.36E-03
cds.Camellia_sinensis.comp14034_c0_seq1_m.41985	PREDICTED: inorganic pyrophosphatase 2 [Theobroma cacao]	30.709	1.8505	3.7	1	1	1	1.046	1.07	1.047	0.876	0.922	1.025	0.893	6.88E-02

cds.Camellia_sinensis.comp14043_c0_seq1_m.732	PREDICTED: callose synthase 9 [Jatropha curcas]	219.55	12.406	2.6	5	6	4	0.962	1.005	1.02	0.985	1.043	0.999	1.013	6.17E-01
cds.Camellia_sinensis.comp14102_c0_seq1_m.37770	PREDICTED: 50S ribosomal protein L6, chloroplastic-like [Malus domestica]	24.949	17.897	40.7	9	12	9	1.123	1.114	1.143	0.835	0.862	0.826	0.746	3.98E-05
cds.Camellia_sinensis.comp14106_c0_seq1_m.25350	PREDICTED: D-amino-acid transaminase, chloroplastic [Vitis vinifera]	43.381	3.1102	2.5	1	1	1								
cds.Camellia_sinensis.comp14113_c0_seq1_m.25364	PREDICTED: pheophytinase, chloroplastic [Ziziphus jujuba]	41.845	2.6148	4.5	2	2	2	1.088	1.012	1.018	0.891	0.973	1.003	0.919	1.17E-01

cds.Camellia_sinensis.comp14132_c0_seq2_m.40264	PREDICTED: ATP-dependent Clp protease proteolytic subunit 2, mitochondrial-like [Nicotiana tabacum]	28.391	15.948	26.7	6	9	6	1.041	1.027	0.957	0.976	1.025	1.007	0.994	8.71E-01
cds.Camellia_sinensis.comp14159_c0_seq1_m.5231	PREDICTED: protein argonaute 5 [Vitis vinifera]	109.24	39.289	13	13	17	13	0.997	1.003	1.05	1.008	0.96	0.989	0.97	2.27E-01
cds.Camellia_sinensis.comp14167_c0_seq1_m.35045	PREDICTED: glutathione S-transferase U17 [Eucalyptus grandis]	25.834	33.405	19.5	5	8	5	1.064	1.078	1.037	0.963	0.884	0.967	0.885	1.74E-02
cds.Camellia_sinensis.comp14201_c0_seq1_m.20780	PREDICTED: hydroquinone glucosyltransferase-like [Nicotiana sylvestris]	58.009	4.1695	6.1	4	4	3	1.061	1.119	1.038	0.944	0.917	0.886	0.854	5.42E-03

cds.Camellia_sinensis.comp14270_c0_seq1_m.33370	PREDICTED: enoyl-[acyl-carrier-protein] reductase [NADH], chloroplastic [Nelumbo nucifera]	31.24	6.3352	31.4	9	25	3	1.012	1.036	0.98	1.055	1.018	0.935	0.993	8.55E-01
cds.Camellia_sinensis.comp14278_c0_seq1_m.18955	PREDICTED: zinc finger CCCH domain-containing protein 37 isoform X1 [Nelumbo nucifera]	58.591	68.879	12.9	6	8	6	0.979	1.021	0.981	1.005	0.992	0.994	1.003	8.17E-01
cds.Camellia_sinensis.comp14306_c0_seq1_m.53197	centrosomal protein [Arabidopsis thaliana]	17.68	1.8398	12.5	2	2	2								
cds.Camellia_sinensis.comp14308_c0_seq1_m.56539		12.959	49.047	26	3	5	3	1.149	1.123	1.164	0.844	0.808	0.854	0.729	9.55E-05

cds.Camellia_sinensis.comp14350_c0_seq1_m.44674	PREDICTED: CAP-Gly domain-containing linker protein 1 isoform X1 [Sesamum indicum]	16.367	75.071	25.3	3	5	3	0.956	1.008	0.999	1.077	0.973	1.031	1.04	3.17E-01
cds.Camellia_sinensis.comp14364_c0_seq1_m.35071	chromosome-associated kinesin [Arabidopsis thaliana]	28.048	52.699	30	7	8	7	1.009	0.974	1.036	0.97	0.974	1.013	0.979	4.13E-01
cds.Camellia_sinensis.comp14428_c0_seq1_m.32371	PREDICTED: protein-L-isoaspartate O-methyltransferase 1 isoform X2 [Sesamum indicum]	33.385	12.93	17.6	5	5	5	0.96	0.959	0.99	1.083	0.981	1.025	1.062	1.23E-01
cds.Camellia_sinensis.comp14487_c0_seq1_m.36591	triosephosphate isomerase, cytosolic [Capsicum annuum]	27.853	219.05	53.5	11	43	11	1.024	1.003	0.995	1	0.997	0.985	0.987	2.44E-01
cds.Camellia_sinensis.comp14530_c0_seq1_m.42158	Thioredoxin-like protein 4A [Morus notabilis]	16.561	1.899	9.9	1	1	1	0.845	1.177	0.822	1.08	1.038	1.07	1.121	3.85E-01

cds.Camellia_sinensis.comp145391_c0_seq1_m.34585	PREDICTED: alpha-amylase-like isoform X1 [Solanum tuberosum	42.66	9.2585	11	3	5	3	0.963	0.945	0.984	1.1	1.069	0.993	1.093	5.35E-02
cds.Camellia_sinensis.comp145442_c0_seq2_m.28332	PREDICTED: aldehyde dehydrogenase family 2 member B7, mitochondrial-like [Nelumbo nucifera]	42.468	43.081	24.3	10	28	4	0.949	0.934	0.954	1.165	1.069	0.985	1.135	1.26E-01
cds.Camellia_sinensis.comp145446_c0_seq1_m.32603	PREDICTED: derlin-1-like [Nicotiana tabacum]	27.492	4.7639	9.2	2	2	2	1.019	0.976	1.016	1.046	0.947	1.006	0.996	8.92E-01
cds.Camellia_sinensis.comp145663_c0_seq1_m.17079	PREDICTED: glycerol-3-phosphate 2-O-acyltransferase 4 [Capsicum annuum]	57.936	4.0159	5.2	3	3	3								

cds.Camellia_sinensis.comp14596_c0_seq1_m.39671	PREDICTED: 1-aminocyclopropane-1-carboxylate oxidase homolog 1-like [Prunus mume]	27.748	11.908	22.7	5	7	5	0.984	0.922	0.982	1.003	1.07	1.003	1.065	1.06E-01
cds.Camellia_sinensis.comp14651_c0_seq1_m.44151	anthocyanidin reductase [Vitis vinifera]	13.547	15.52	37.4	3	5	3	1.259	1.307	1.22	0.707	0.747	0.665	0.56	1.19E-04
cds.Camellia_sinensis.comp14653_c0_seq2_m.41836	PREDICTED: peptide methionine sulfoxide reductase B5-like [Pyrus x bretschneideri]	23.11	82.647	34.3	5	11	4	0.995	0.928	0.917	1.034	1.069	1.045	1.108	1.92E-02
cds.Camellia_sinensis.comp14661_c0_seq1_m.38530	PREDICTED: thioredoxin H9 [Erythranthe guttata]	15.55	2.905	9.4	1	1	1								
cds.Camellia_sinensis.comp14664_c0_seq1_m.4137	PREDICTED: histone H1 [Vitis vinifera]	46.714	6.9213	5.9	3	4	3								

cds.Camellia_sinensis.comp14674_c0_seq2_m.51551	PREDICTED: peroxidase 4-like [Vitis vinifera]	17.319	42.14	44.8	6	10	4	0.979	1.025	1.084	0.919	0.946	1.042	0.941	2.73E-01
cds.Camellia_sinensis.comp14680_c0_seq1_m.21638	Chaperone protein DnaJ [Morus notabilis]	57.761	11.501	13	7	8	7	1.112	1.034	1.129	0.936	0.859	0.902	0.824	6.20E-03
cds.Camellia_sinensis.comp14695_c0_seq1_m.52872	myosin-G heavy chain-like protein [Arabidopsis thaliana]	16.92	1.4243	9.6	1	1	1								
cds.Camellia_sinensis.comp14706_c0_seq1_m.41134	PREDICTED: hydroquinone glucosyltransferase-like [Nicotiana sylvestris]	32.264	6.2764	11.6	4	5	2								
cds.Camellia_sinensis.comp14707_c0_seq1_m.20162	Sugar transport protein 5 [Morus notabilis]	43.697	4.1782	3.6	1	1	1	1.047	1.061	1.036	0.957	0.945	0.947	0.906	2.41E-04
cds.Camellia_sinensis.comp14709_c0_seq1_m.42842	PREDICTED: 60S ribosomal protein L26-1-like [Nicotiana tabacum]	20.09	8.8667	22.5	4	9	4	1.053	1.059	1.01	0.96	0.943	0.922	0.905	6.22E-03

cds.Camellia_sinensis.comp14741_c0_seq1_m.57407	PREDICTED: proteasome subunit alpha type-3-like [Populus euphratica]	12.794	4.354	43.5	5	9	2	0.876	0.844	1.141	1.109	1.201	0.877	1.114	4.63E-01
cds.Camellia_sinensis.comp14760_c0_seq1_m.25844	PREDICTED: mitochondrial import inner membrane translocase subunit TIM50-like [Solanum tuberosum	41.721	2.1041	2.5	1	1	1	0.994	0.971	0.989	0.973	1.055	1.029	1.035	2.48E-01
cds.Camellia_sinensis.comp14761_c0_seq1_m.43823	PREDICTED: pyruvate kinase isozyme G, chloroplastic [Theobrom	21.999	9.8489	9.5	2	2	2	0.969	0.997	0.99	0.99	1.033	1.035	1.035	1.15E-01
cds.Camellia_sinensis.comp14771_c0_seq1_m.16671	PREDICTED: NADP-dependent glyceraldehyde-3-phosphate dehydrogenase [Sesamum indicum]	57.801	286.17	49.7	21	62	21	0.997	1.033	1.013	1.006	0.972	1.005	0.98	2.61E-01

cds.Camellia_sinensis.comp14778_c0_seq1_m.21986	PREDICTED: phytochrome-associated serine/threonine-protein phosphatase [Ipomoea	34.961	32.52	9.5	3	5	3	0.94	0.962	0.999	1.037	1.048	1.023	1.071	2.22E-02
cds.Camellia_sinensis.comp14779_c0_seq1_m.55154	PREDICTED: AAA-ATPase At5g57480-like [Juglans regia]	13.848	1.7459	8.2	1	1	1	1.079	1.108	1.064	1.036	0.748	0.949	0.841	2.03E-01
cds.Camellia_sinensis.comp14794_c0_seq1_m.31056	PREDICTED: glutathionyl-hydroquinone reductase YqjG [Ricinus communis]	42.705	127.24	21.7	7	18	7	0.935	0.958	0.963	1.076	1.061	1.026	1.107	3.72E-03
cds.Camellia_sinensis.comp14803_c0_seq2_m.38105	PREDICTED: ubiquitin thioesterase OTU1 isoform X1 [Nicotiana attenuata]	23.159	21.482	18.4	3	5	3	0.958	0.97	0.929	1.079	0.993	1.096	1.109	3.66E-02

cds.Camellia_sinensis.comp14827_c0_seq1_m.33282	PREDICTED: chlorophyll a-b binding protein 6A, chloroplastic [Nicotiana attenuata]	26.332	36.237	21.2	9	29	9	0.947	0.896	0.882	1.09	1.122	1.103	1.217	1.06E-03
cds.Camellia_sinensis.comp14849_c0_seq1_m.54978	PREDICTED: HMG-Y-related protein A [Theobroma cacao]	14.829	3.189	10.9	1	1	1	0.962	1.355	1.214	0.823	0.859	0.761	0.692	2.83E-02
cds.Camellia_sinensis.comp14856_c0_seq1_m.31110	PREDICTED: BAHD acyltransferase DCR [Ricinus communis]	48.112	1.166	3	1	1	1								
cds.Camellia_sinensis.comp14866_c0_seq1_m.29987	PREDICTED: chlorophyll a-b binding protein 7, chloroplastic [Solanum tuberosum	33.769	72.027	23.7	4	14	4	0.97	0.943	0.979	0.994	1.018	1.031	1.052	3.07E-02

cds.Camellia_sinensis.comp14868_c0_seq1_m.51576	PREDICTED: heavy metal-associated isoprenylated plant protein 26-like [Sesamum indicum]	15.323	1.323	5.1	1	1	1	1.373	1.289	1.244	0.632	0.668	0.691	0.51	6.24E-05
cds.Camellia_sinensis.comp14885_c0_seq1_m.20656	serine carboxypeptidase S10 family protein [Populus trichocarpa]	51.194	15.302	6.3	3	4	3	1.039	1.043	1.045	0.946	0.96	0.99	0.926	2.95E-02
cds.Camellia_sinensis.comp14900_c0_seq1_m.9568	PREDICTED: glutamine-dependent NAD(+) synthetase [Sesamum indicum]	89.256	5.7361	3.1	3	4	3	0.972	1.023	1.019	1.006	0.988	0.976	0.985	4.80E-01
cds.Camellia_sinensis.comp14948_c0_seq1_m.21121	PREDICTED: 26S protease regulatory subunit 7 [Gossypium hirsutum]	47.654	165.82	55.9	22	48	21	1.004	1.018	1.019	1.008	0.986	1.007	0.987	2.00E-01

cds.Camellia_sinensis.comp14952_c0_seq1_m.34818	PREDICTED: probable carboxylesterase 8 [Theobroma cacao]	37.333	2.5966	4.7	1	2	1	1.029	0.979	1.107	1.054	0.789	1.042	0.926	4.53E-01
cds.Camellia_sinensis.comp14985_c0_seq1_m.42840	PREDICTED: thioredoxin X, chloroplastic [Prunus mume]	21.136	21.583	15.4	3	6	3	0.957	1.042	0.996	0.967	1.04	0.956	0.989	7.79E-01
cds.Camellia_sinensis.comp14986_c0_seq1_m.37943	PREDICTED: ribulose bisphosphate carboxylase small chain, chloroplastic-like [Juglans regia]	19.795	23.148	40.3	10	96	6	0.997	1.029	1.061	0.982	0.979	0.969	0.949	4.79E-02
cds.Camellia_sinensis.comp15033_c0_seq1_m.11003	PREDICTED: nudix hydrolase 26, chloroplastic-like isoform X2 [Nicotiana attenuata]	20.647	2.9544	11.6	1	1	1	1.255	0.954	1.01	0.974	0.774	0.998	0.853	2.46E-01

cds.Camellia_sinensis.comp15033_c0_seq1_m.11004	PREDICTED: photosystem II 10 kDa polypeptide, chloroplastic [Juglans regia]	14.263	26.492	23.2	3	13	3	1.13	1.056	1.106	0.885	0.916	0.884	0.816	9.21E-04
cds.Camellia_sinensis.comp15037_c0_seq1_m.35395	PREDICTED: thylakoid membrane protein slr0575 [Solanum tuberosum	31.624	89.845	34.1	9	17	9	0.981	1.035	1.009	1.006	1.024	0.972	0.992	7.44E-01
cds.Camellia_sinensis.comp15078_c0_seq1_m.40747	PREDICTED: putative 4-hydroxy-4-methyl-2-oxoglutarate aldolase 2 [Nicotiana attenuata]	17.8	23.837	37.3	6	8	5	1.008	1	1.001	0.982	0.969	1.005	0.982	1.78E-01
cds.Camellia_sinensis.comp15079_c0_seq1_m.27628	PREDICTED: bifunctional protein FolD 4, chloroplastic isoform X2 [Citrus sinensis]	38.976	37.062	22	9	15	9	1	1.046	1.009	0.951	0.972	0.977	0.949	3.17E-02

cds.Camellia_sinensis.comp15085_c0_seq1_m.32666	PREDICTED: non-functional NADPH-dependent codeinone reductase 2-like [Nicotiana tabacum]	35.677	11.693	14.4	6	11	4	0.924	0.889	0.857	1.116	1.156	1.103	1.264	8.22E-04
cds.Camellia_sinensis.comp15105_c0_seq1_m.45326	PREDICTED: galactokinase-like, partial [Ziziphus jujuba]	22.37	55.949	46.6	7	10	5	0.98	0.99	1.033	0.972	1.018	0.99	0.992	7.35E-01
cds.Camellia_sinensis.comp15118_c0_seq1_m.21662	PREDICTED: chromatin modification-related protein eaf-1-like isoform X2 [Pyrus x bretschneideri]	53.075	20.007	23.8	9	10	8	0.995	1.027	0.995	0.986	1.013	1.018	1	9.99E-01
cds.Camellia_sinensis.comp15125_c0_seq1_m.36668	PREDICTED: oxygen-evolving enhancer protein 2, chloroplastic-like [Nelumbo nucifera]	28.472	234.15	45.3	12	82	12	1.017	1.002	0.994	1.014	0.977	0.985	0.988	3.97E-01

cds.Camellia_sinensis.comp15130_c0_seq1_m.33712	PREDICTED: 2-C-methyl-D-erythritol 2,4-cyclodiphosphate synthase, chloroplastic [Ipomoea nil]	27.806	45.091	28.2	6	9	6	1.036	1.035	0.951	1.052	1.047	0.916	0.998	9.50E-01
cds.Camellia_sinensis.comp15131_c0_seq1_m.44648	PREDICTED: peptidyl-prolyl cis-trans isomerase FKBP17-1, chloroplastic-like isoform X1 [Nicotiana tabacum]	24.294	1.4097	4.5	1	1	1	0.971	1.092	0.867	1.076	1.04	0.973	1.054	4.82E-01
cds.Camellia_sinensis.comp15138_c0_seq1_m.58116	PREDICTED: LOW QUALITY PROTEIN: hydroquinone glucosyltransferase [Ricinus communis]	11.945	27.267	19.3	1	2	1	0.93	1.038	0.971	1.106	0.939	1.04	1.05	4.57E-01

cds.Camellia_sinensis.comp15156_c0_seq2_m.19699	PREDICTED: protease Do-like 8, chloroplastic isoform X1 [Juglans regia]	53.255	61.765	22.8	11	23	11	0.99	0.976	0.978	1.016	1.028	1.023	1.042	1.86E-03
cds.Camellia_sinensis.comp151911_c0_seq1_m.50408	PREDICTED: EG45-like domain containing protein [Juglans regia]	12.876	57.379	42.4	3	7	3	0.68	0.698	0.747	1.243	1.34	1.394	1.872	1.39E-04
cds.Camellia_sinensis.comp15207_c0_seq1_m.26369	PREDICTED: lipoyl synthase 2, mitochondrial-like [Nicotiana attenuata]	44.761	7.6395	9	3	3	3	1.01	0.919	1.001	1.041	1.067	0.98	1.054	2.50E-01
cds.Camellia_sinensis.comp15246_c0_seq1_m.45602	PREDICTED: phosphatidylinositol 3,4,5-trisphosphate 3-phosphatase and protein-tyrosine-phosphatase PTEN2A[Vitis vinifera]	14.7	1.9197	6.4	1	1	1	1.196	1.115	1.227	0.844	0.752	0.817	0.682	1.02E-03

cds.Camellia_sinensis.comp15287_c0_seq1_m.18815	PREDICTED: 6-phosphogluconate dehydrogenase, decarboxylating 3 [Ziziphus jujuba]	53.493	131.91	42.7	19	44	17	1.015	1.001	0.986	0.997	1.016	0.989	1	1.00E+00
cds.Camellia_sinensis.comp15297_c0_seq1_m.4262	ATP synthase CF1 alpha subunit (chloroplast) [Camellia pubicosta]	55.468	323.31	38.7	18	112	16	0.987	0.976	0.992	1.008	1.017	1.02	1.03	7.38E-03
cds.Camellia_sinensis.comp15298_c0_seq1_m.35004	PREDICTED: psbP domain-containing protein 6, chloroplastic [Vitis vinifera]	29.406	109.67	47.2	11	22	11	0.991	0.982	0.967	1.034	1.053	1.032	1.061	3.54E-03
cds.Camellia_sinensis.comp15306_c0_seq1_m.24456	PREDICTED: vacuolar protein sorting-associated protein 26B [Vitis vinifera]	35.199	2.1279	10.6	3	3	2								

cds.Camellia_sinensis.comp15326_c0_seq1_m.35297	PREDICTED: superoxide dismutase [Cu-Zn], chloroplastic [Daucus carota subsp. sativus]	21.644	166.42	50.2	6	21	4	0.954	0.932	0.928	1.056	1.101	1.065	1.145	9.18E-04
cds.Camellia_sinensis.comp15329_c0_seq1_m.14629	PREDICTED: nucleobase-ascorbate transporter 3 [Solanum tuberosum	60.284	2.4328	1.8	1	1	1	0.894	1.048	0.969	1.134	1.032	0.957	1.073	3.56E-01
cds.Camellia_sinensis.comp15334_c0_seq1_m.36519	PREDICTED: oxygen-evolving enhancer protein 3-2, chloroplastic [Pyrus x bretschneideri]	24.704	144.61	42.5	12	86	12	1.003	1.003	0.989	1	1.003	0.993	1	9.53E-01
cds.Camellia_sinensis.comp15342_c0_seq1_m.36209	PREDICTED: protein ABHD11 [Vitis vinifera]	20.277	1.8572	15.9	3	3	3	1.055	1.021	0.995	1.076	0.926	0.931	0.955	4.07E-01

cds.Camellia_sinensis.comp15342_c0_seq1_m.36210	PREDICTED: alpha/beta hydrolase domain-containing protein 11 [Citrus sinensis]	14.115	9.2371	36.8	5	5	5	0.947	0.956	1.055	1.044	1.031	0.989	1.036	3.98E-01
cds.Camellia_sinensis.comp15355_c0_seq1_m.41637	photosystem I reaction center subunit IV A [Medicago truncatula]	16.886	88.761	50	6	41	6	1.071	1.014	1.012	0.93	0.986	0.958	0.928	4.09E-02
cds.Camellia_sinensis.comp15359_c0_seq1_m.28895	PREDICTED: cytochrome b-c1 complex subunit Rieske-4, mitochondrial-like [Sesamum indicum]	29.243	104.76	20.9	5	12	5	0.991	1.019	1.017	0.99	1.02	1.003	0.995	7.29E-01
cds.Camellia_sinensis.comp15364_c0_seq1_m.17207	PREDICTED: GMP synthase [glutamine-hydrolyzing]-like [Daucus carota subsp. sativus]	63.741	65.823	20.5	10	13	10	0.945	0.966	0.925	1.052	1.057	1.085	1.126	1.66E-03

cds.Camellia_sinensis.comp15365_c0_seq1_m.25957	PREDICTED: psbP domain-containing protein 1, chloroplastic [Vitis vinifera]	30.511	110.59	34.1	9	14	9	1.02	1.024	1.006	1.006	0.971	1.009	0.979	1.88E-01
cds.Camellia_sinensis.comp15366_c0_seq1_m.9995	PREDICTED: glutamate--tRNA ligase, cytoplasmic isoform X1 [Vitis vinifera]	83.157	82.443	23.2	18	23	18	0.968	1.009	0.975	1.011	1.005	0.998	1.021	1.91E-01
cds.Camellia_sinensis.comp15377_c0_seq1_m.51446	PREDICTED: WD repeat-containing protein 20-like [Nicotiana tomentosiformis]	11.18	5.3034	19.4	1	1	1	0.949	0.974	0.77	1.233	0.894	1.217	1.242	1.76E-01
cds.Camellia_sinensis.comp15383_c0_seq2_m.37999	transmembrane protein, putative [Medicago truncatula]	26.885	21.071	9.6	2	3	2	0.95	1.023	1.09	0.934	1.033	0.952	0.953	3.98E-01

cds.Camellia_sinensis.comp15418_c0_seq1_m.38718	PREDICTED: chlorophyll a-b binding protein, chloroplastic [Sesamum indicum]	20.814	9.0459	5.8	1	3	1	0.771	1.127	1.133	1.001	0.941	1.057	0.989	9.79E-01
cds.Camellia_sinensis.comp15473_c0_seq1_m.58569	PREDICTED: DEAD-box ATP-dependent RNA helicase 31-like isoform X2 [Juglans regia]	11.445	1.6649	12.3	1	1	1	0.854	0.882	1.116	1.063	0.941	1.179	1.116	3.49E-01
cds.Camellia_sinensis.comp15485_c0_seq1_m.44167	DUF1230 family protein [Medicago truncatula]	23.827	3.3517	5.5	1	1	1	0.965	0.988	0.96	1.064	0.983	1.059	1.066	7.91E-02
cds.Camellia_sinensis.comp15493_c0_seq1_m.30430	PREDICTED: heat shock cognate 70 kDa protein 2-like [Solanum pennellii]	48.561	213.42	56.1	23	92	6	1.051	1.025	1.028	0.959	0.953	0.976	0.93	2.48E-03

cds.Camellia_sinensis.comp15502_c0_seq1_m.12796	PREDICTED: phosphoglucan phosphatase LSF1, chloroplastic isoform X1 [Vitis vinifera]	55.465	4.08	3.2	2	2	2	0.97	0.964	0.972	1.113	1.087	0.92	1.074	3.77E-01
cds.Camellia_sinensis.comp15502_c0_seq1_m.12797	PREDICTED: phosphoglucan phosphatase LSF1, chloroplastic [Ipomoea nil]	11.887	1.2785	6.4	1	1	1	1.034	0.899	1.017	1.027	1.005	1.029	1.038	4.35E-01
cds.Camellia_sinensis.comp15518_c0_seq1_m.22248	PREDICTED: dnaJ protein homolog 2 isoform X2 [Solanum pennellii]	46.487	54.094	16.9	7	12	2	1.26	1.201	1.286	0.744	0.787	0.751	0.609	4.47E-05
cds.Camellia_sinensis.comp15527_c0_seq1_m.24033	DUF1995 domain protein, putative (DUF1995) [Arabidopsis thaliana]	41.608	54.694	19.2	5	7	5	1.028	1.038	1.059	0.989	0.903	0.967	0.915	3.55E-02

cds.Camellia_sinensis.comp15528_c0_seq1_m.32944	PREDICTED: protein GrpE [Ricinus communis]	34.762	34.77	28.4	11	18	11	1.002	1.041	1.019	0.988	0.962	0.991	0.96	5.02E-02
cds.Camellia_sinensis.comp15601_c0_seq1_m.41643	PREDICTED: protochlorophyllide reductase, chloroplastic [Ricinus communis]	30.961	2.1983	24.2	6	6	3	0.906	0.963	0.98	1.059	1.076	1.048	1.117	1.12E-02
cds.Camellia_sinensis.comp15602_c0_seq1_m.26722	PREDICTED: ABC transporter I family member 6, chloroplastic-like [Nicotiana tabacum]	38.475	60.888	18.4	5	9	5	1.029	1.007	1.007	0.968	0.975	1.018	0.973	1.87E-01
cds.Camellia_sinensis.comp15618_c0_seq1_m.27755	PREDICTED: BRCA1-A complex subunit Abraxas [Jatropha curcas]	39.021	5.8587	12.5	4	4	4	0.998	1.053	0.986	1.021	0.945	1.007	0.979	5.30E-01

cds.Camellia_sinensis.comp15666_c0_seq1_m.32513	PREDICTED: vacuolar protein sorting-associated protein 20 homolog 2-like [Nicotiana tabacum]	26.302	5.3656	12.8	3	3	3	1.069	1.098	1.08	0.905	0.898	0.933	0.843	2.59E-04
cds.Camellia_sinensis.comp15676_c0_seq1_m.56970	PREDICTED: photosystem II 22 kDa protein, chloroplastic [Vitis vinifera]	12.716	57.07	35.3	3	7	1	1.06	1.032	1.081	0.936	0.992	0.947	0.906	1.14E-02
cds.Camellia_sinensis.comp15677_c0_seq1_m.38526	PREDICTED: metal-independent phosphoserine phosphatase [Gossypium arboreum]	26.6	6.9332	13	2	2	2	1.08	0.851	0.919	1.238	1.015	0.925	1.115	3.92E-01
cds.Camellia_sinensis.comp15686_c0_seq1_m.2101	PREDICTED: protein NBR1 homolog isoform X2 [Citrus sinensis]	92.885	4.9446	5.4	3	3	3								

cds.Camellia_sinensis.comp15686_c0_seq1_m.2103	PREDICTED: adenosine kinase 2 [Vitis vinifera]	19.639	95.336	31.1	3	5	3	1.027	1.116	1.029	0.859	1.018	0.939	0.888	9.81E-02
cds.Camellia_sinensis.comp15686_c0_seq1_m.2104	PREDICTED: adenosine kinase 1-like [Gossypium hirsutum]	16.822	97.817	48	5	18	5	1.042	1.045	0.999	0.982	0.942	0.982	0.942	4.04E-02
cds.Camellia_sinensis.comp15711_c0_seq1_m.22211	PREDICTED: fatty-acid-binding protein 1 [Vitis vinifera]	11.424	3.8027	10.8	1	1	1								
cds.Camellia_sinensis.comp15716_c0_seq1_m.31680	PREDICTED: putative glucose-6-phosphate 1-epimerase isoform X1 [Ricinus communis]	34.406	29.71	19.1	5	11	5	1.066	1.021	1.032	0.932	0.943	0.949	0.905	2.10E-03
cds.Camellia_sinensis.comp15718_c0_seq1_m.36938	PREDICTED: peroxidase 73 [Vitis vinifera]	34.324	129.59	39.7	10	20	8	1.022	0.986	1.024	0.983	0.992	0.981	0.975	1.19E-01

cds.Camellia_sinensis.comp15730_c0_seq1_m.27007	PREDICTED: dihydrolipoyllysine-residue acetyltransferase component 1 of pyruvate dehydrogenase complex,mitochondrial-like isoform X7 [Gossypium hirsutum]	40.459	16.019	18.8	7	8	6	0.941	1	0.969	1.018	1.049	1.044	1.069	2.80E-02
cds.Camellia_sinensis.comp15731_c0_seq1_m.30644	PREDICTED: clathrin heavy chain 1-like isoform X2 [Juglans regia]	50.439	4.2616	21.9	9	19	2	0.945	1.162	1.025	0.959	0.869	1.04	0.916	3.35E-01
cds.Camellia_sinensis.comp15748_c0_seq1_m.44277	PREDICTED: glyceraldehyde-3-phosphate dehydrogenase B, chloroplastic [Malus domestica]	19.306	253.51	85.1	13	60	10	1.003	0.994	1.003	0.999	1.008	0.983	0.997	6.92E-01

cds.Camellia_sinensis.comp15756_c0_seq1_m.36168	PREDICTED: UBP1-associated protein 2C isoform X2 [Theobroma cacao]	36.561	1.4143	2.7	1	1	1								
cds.Camellia_sinensis.comp15767_c0_seq1_m.45369	PREDICTED: myosin-11 isoform X2 [Citrus sinensis]	13.944	11.937	34.1	5	6	5	0.986	0.972	0.924	1.011	1.034	1.109	1.094	5.81E-02
cds.Camellia_sinensis.comp15790_c1_seq1_m.53352	PREDICTED: SKP1-like protein 1B [Citrus sinensis]	11.715	1.3124	39	6	6	2	0.92	0.907	0.892	1.157	1.077	1.089	1.222	1.12E-03
cds.Camellia_sinensis.comp15819_c0_seq1_m.3488	PREDICTED: nodal modulator 1 [Vitis vinifera]	131.25	110	15.3	15	21	15	0.991	0.983	1.013	1.002	0.976	0.992	0.994	6.55E-01
cds.Camellia_sinensis.comp15853_c0_seq1_m.14378	PREDICTED: U1 small nuclear ribonucleoprotein A-like [Gossypium raimondii]	36.069	40.859	28.3	8	9	8	0.93	0.943	0.999	1.062	1.13	1.036	1.124	2.62E-02

cds.Camellia_sinensis.comp15864_c0_seq1_m.58049	PREDICTED: heat shock cognate 70 kDa protein 2 [Ricinus communis]	12.663	18.529	51.8	8	34	4	1.081	0.992	0.997	0.947	0.99	0.995	0.955	2.28E-01
cds.Camellia_sinensis.comp15872_c0_seq1_m.39633	PREDICTED: probable U6 snRNA-associated Sm-like protein LSm4 [Ipomoea nil]	16.096	1.7176	9.3	2	2	2								
cds.Camellia_sinensis.comp15888_c0_seq1_m.4259	oxidoreductase/transition metal ion-binding protein, putative [Medicago truncatula]	101.7	177.94	27.1	24	30	24	1.024	0.981	1.029	0.98	0.988	0.986	0.974	1.60E-01
cds.Camellia_sinensis.comp15966_c0_seq1_m.15878	PREDICTED: O-acyltransferase WSD1-like [Daucus carota subsp. sativus]	60.034	90.549	26.2	14	32	14	1.114	1.1	1.11	0.874	0.876	0.885	0.793	8.60E-07

cds.Camellia_sinensis.comp15985_c0_seq1_m.20246	stress up-regulated Nod 19 protein [Medicago truncatula]	51.937	52.957	14.8	7	11	7	1.005	1.065	1.056	0.978	0.954	0.959	0.925	1.65E-02
cds.Camellia_sinensis.comp15996_c0_seq1_m.35151	electron carrier/ oxidoreductase [Arabidopsis lyrata subsp. lyrata]	32.317	23.083	27.6	6	8	6	0.992	1.073	1.012	0.936	0.954	0.968	0.929	4.51E-02
cds.Camellia_sinensis.comp16016_c0_seq1_m.17394	PREDICTED: WPP domain-interacting protein 1 [Theobroma cacao]	65.063	9.7238	7.8	4	5	4	0.969	1.113	0.994	0.952	1.003	0.974	0.952	3.54E-01
cds.Camellia_sinensis.comp16030_c0_seq1_m.20464	PREDICTED: low-temperature-induced cysteine proteinase [Nelumbo nucifera]	48.443	30.205	19.1	6	9	6	0.905	0.989	0.942	1.086	1.023	1.074	1.122	2.14E-02
cds.Camellia_sinensis.comp16051_c0_seq1_m.15178	PREDICTED: aspartic proteinase A1-like [Nelumbo nucifera]	55.138	56.905	21.5	10	28	10	1.053	1.05	1.033	0.935	0.94	0.959	0.904	4.83E-04

cds.Camellia_sinensis.comp16063_c0_seq1_m.46848	NtPRp27-like protein precursor [Solanum tuberosum	25.568	14.293	9.3	2	5	1	0.932	0.867	0.836	1.12	1.183	1.106	1.294	2.46E-03
cds.Camellia_sinensis.comp16098_c0_seq1_m.39069	PREDICTED: rhodanese-like domain-containing protein 10 isoform X1 [Vitis vinifera]	24.434	14.456	24.1	4	5	4	1.086	1.064	1.066	0.885	0.934	0.927	0.854	9.39E-04
cds.Camellia_sinensis.comp16103_c0_seq1_m.15293	PREDICTED: magnesium transporter MRS2-11, chloroplastic [Jatropha	40.64	22.585	22	7	8	7	1.025	1.023	1.075	0.979	0.954	0.938	0.919	1.47E-02
cds.Camellia_sinensis.comp16110_c0_seq1_m.43593	PREDICTED: glycosyltransferase-like protein gnt13 [Nicotiana tomentosiformis]	19.215	6.9989	10.7	2	7	2	0.991	0.948	0.917	1.002	1.093	1.015	1.089	7.32E-02

cds.Camellia_sinensis.comp16116_c0_seq1_m.38134	PREDICTED: remorin [Daucus carota subsp. sativus]	30.655	1.4493	3.4	1	1	1								
cds.Camellia_sinensis.comp16127_c0_seq1_m.46989	PREDICTED: NAD(P)H-quinone oxidoreductase subunit L, chloroplastic [Vitis vinifera]	23.33	3.1694	11	2	3	2	0.955	0.96	0.997	0.991	1.075	1.042	1.067	7.67E-02
cds.Camellia_sinensis.comp16154_c0_seq1_m.42494	PREDICTED: glycine-rich protein 2-like [Juglans regia]	19.893	132.3	26.6	4	9	4	1.083	1.06	1.034	0.937	0.904	0.946	0.877	2.44E-03
cds.Camellia_sinensis.comp16161_c0_seq1_m.34022	PREDICTED: pleiotropic drug resistance protein 2-like [Fragaria vesca subsp. vesca]	43.652	7.0768	4.4	1	1	1	0.813	1.007	0.971	1.097	1.084	1.074	1.166	1.40E-01

cds.Camellia_sinensis.comp16161_c0_seq2_m.41376	PREDICTED: pleiotropic drug resistance protein 2-like isoform X3 [Nelumbo nucifera]	33.68	4.0356	6.2	2	2	2	1.02	1.068	1.052	0.934	0.947	0.974	0.909	6.54E-03
cds.Camellia_sinensis.comp16164_c0_seq1_m.43742	PREDICTED: NAD(P)H-quinone oxidoreductase subunit O, chloroplastic [Citrus sinensis]	21.496	2.7668	8.4	2	3	2	1.049	1.104	1.05	0.998	0.918	0.873	0.871	3.04E-02
cds.Camellia_sinensis.comp16191_c0_seq1_m.18823	DNA binding protein [Arabidopsis thaliana]	47.717	9.9999	8.8	3	3	3	1.035	1.065	0.961	0.869	0.985	1.075	0.957	5.33E-01
cds.Camellia_sinensis.comp16207_c0_seq1_m.48693	PREDICTED: hypersensitive-induced response protein 2 [Erythranthe guttata]	23.34	3.8695	11.7	3	6	1	1.008	0.983	0.853	1.111	0.939	1.124	1.116	2.34E-01

cds.Camellia_sinensis.comp16225_c0_seq2_m.40027	TPR repeat protein [Medicago truncatula]	26.525	1.3692	3.3	1	1	1								
cds.Camellia_sinensis.comp16250_c0_seq1_m.38511	PREDICTED: profilin-1 [Vitis vinifera]	14.145	13.6	29.8	3	3	2	0.936	1.015	1.124	1.111	0.903	0.93	0.957	6.19E-01
cds.Camellia_sinensis.comp16265_c0_seq1_m.29040	PREDICTED: exosome complex component RRP4 homolog [Vitis vinifera]	37.256	7.6043	6.7	2	2	2								
cds.Camellia_sinensis.comp16285_c0_seq1_m.52727	PREDICTED: 40S ribosomal protein S4-3-like [Pyrus x bretschneideri]	18.551	-2	26.7	5	11	1	1.184	1.142	1.179	0.822	0.83	0.796	0.698	2.49E-05
cds.Camellia_sinensis.comp16348_c0_seq1_m.43055	PREDICTED: 40S ribosomal protein S17-4-like [Daucus carota subsp. sativus]	17.554	18.444	37.1	5	8	5	1.143	1.189	1.055	0.855	0.898	0.806	0.756	4.00E-03

cds.Camellia_sinensis.comp16373_c0_seq1_m.51330	PREDICTED: chalcone synthase [Pyrus x bretschneideri]	19.938	47.804	46.6	6	27	1	1.467	1.452	1.298	0.561	0.511	0.575	0.391	6.01E-05
cds.Camellia_sinensis.comp16389_c0_seq1_m.25964	CAAX amino terminal protease family protein [Medicago truncatula]	20.84	1.3771	5.5	1	1	1	1.014	1.01	1.052	0.962	0.891	1.069	0.95	3.77E-01
cds.Camellia_sinensis.comp16399_c0_seq1_m.26573	PREDICTED: heterogeneous nuclear ribonucleoprotein 1-like [Sesamum indicum]	40.377	2.6678	2.9	1	1	1								
cds.Camellia_sinensis.comp16436_c0_seq1_m.39676	PREDICTED: importin subunit alpha-like [Sesamum indicum]	31.172	13.912	37.9	10	11	4	0.998	1.063	0.993	0.834	1.094	1.015	0.964	6.35E-01

cds.Camellia_sinensis.comp16445_c0_seq1_m.37082	PREDICTED: hydroxyacylglutathione hydrolase cytoplasmic [Nicotiana sylvestris]	28.84	43.735	36.4	9	20	9	0.908	0.93	0.926	1.078	1.068	1.073	1.165	4.07E-05
cds.Camellia_sinensis.comp16447_c0_seq1_m.16705	PREDICTED: ribosome-recycling factor, chloroplastic-like [Gossypium hirsutum]	19.694	95.317	61.8	12	36	12	1.018	1.013	1	1	0.984	0.995	0.983	7.26E-02
cds.Camellia_sinensis.comp16470_c0_seq2_m.43636	transmembrane protein, putative (DUF3464) [Arabidopsis thaliana]	20.415	10.396	12.8	2	2	2	0.985	1.023	1.022	1.051	0.913	1.016	0.983	7.01E-01
cds.Camellia_sinensis.comp165067_c0_seq1_m.53440	PREDICTED: bark storage protein A [Vitis vinifera]	16.928	5.69	16.4	2	2	2	0.924	0.985	1.037	1.201	0.908	0.979	1.048	6.72E-01

cds.Camellia_sinensis.comp16506_c0_seq1_m.29027	PREDICTED: inactive protein RESTRICTED TEV MOVEMENT 2-like [Nicotiana sylvestris]	46.902	28.374	20.1	7	7	7	1.058	1.102	1.119	0.938	0.899	0.903	0.836	1.12E-03
cds.Camellia_sinensis.comp16515_c0_seq1_m.17608	PREDICTED: plant UBX domain-containing protein 10 [Ipomoea nil]	50.334	7.0513	6.7	3	4	3	0.952	1	0.995	0.996	1.027	1.026	1.035	1.40E-01
cds.Camellia_sinensis.comp16538_c0_seq1_m.13562	PREDICTED: dynamin-related protein 5A [Sesamum indicum]	68.501	59.536	28.6	15	20	14	0.973	0.95	1.012	1.053	1.027	1.014	1.054	6.95E-02
cds.Camellia_sinensis.comp16576_c0_seq1_m.36226	PREDICTED: glutathione S-transferase-like [Nelumbo nucifera]	15.216	42.262	42.1	5	15	5	1.019	0.983	0.985	1.014	0.995	1.046	1.023	2.96E-01
cds.Camellia_sinensis.comp16577_c0_seq1_m.15298	PREDICTED: UBP1-associated protein 2A [Vitis vinifera]	54.081	9.8999	7.7	4	5	4	1.129	1.066	1.142	0.974	0.856	0.825	0.796	1.35E-02

cds.Camellia_sinensis.comp16582_c0_seq1_m.22277	PREDICTED: rhodanese-like domain-containing protein 4A, chloroplastic [Vitis vinifera]	42.729	1.6561	4.7	2	2	2	0.916	1.068	1.103	0.926	1.023	0.975	0.947	4.64E-01
cds.Camellia_sinensis.comp16588_c0_seq1_m.38680	PREDICTED: photosystem II reaction center PSB28 protein, chloroplastic [Prunus mume]	21.185	7.5307	18.3	3	4	3	0.971	1.023	0.988	0.989	0.979	0.985	0.99	5.74E-01
cds.Camellia_sinensis.comp16591_c0_seq1_m.34054	PREDICTED: elongation factor 1-beta-like [Sesamum indicum]	24.443	58.605	40	8	24	7	0.994	0.98	0.976	1.013	1.034	1.018	1.039	9.98E-03
cds.Camellia_sinensis.comp16592_c0_seq1_m.51984	PREDICTED: ADP-ribosylation factor-like protein 8B [Musa acuminata subsp. malaccensis]	13.145	19.264	53.8	5	8	4	0.972	1.008	0.945	1.088	1.015	1.008	1.064	1.16E-01

cds.Camellia_sinensis.comp16595_c0_seq1_m.26718	PREDICTED: DNA-directed RNA polymerase V subunit 1 [Vitis	45.641	8.681	6.7	2	2	2								
cds.Camellia_sinensis.comp16607_c0_seq1_m.12090	PREDICTED: mitotic spindle checkpoint protein MAD1 [Vitis vinifera]	51.684	1.7796	2.7	1	1	1								
cds.Camellia_sinensis.comp16608_c0_seq1_m.28401	PREDICTED: pleiotropic drug resistance protein 2-like [Sesamum indicum]	50.888	1.8817	5.7	3	3	0								
cds.Camellia_sinensis.comp16608_c0_seq2_m.32924	PREDICTED: pleiotropic drug resistance protein 2-like [Sesamum indicum]	43.489	16.949	13.3	5	5	3	0.892	0.958	0.897	1.128	1.098	1.07	1.2	2.68E-03

cds.Camellia_sinensis.comp16615_c0_seq1_m.17318	PREDICTED: aminomethyltransferase, mitochondrial [Nelumbo nucifera]	37.162	131.88	46.4	15	56	15	1.045	1	1.016	0.979	0.993	0.984	0.966	6.20E-02
cds.Camellia_sinensis.comp16620_c0_seq1_m.38325	PREDICTED: peroxiredoxin-2B [Populus euphratica]	17.412	44.896	30.9	4	13	4	1.057	1.016	1.059	0.944	0.987	0.952	0.92	1.25E-02
cds.Camellia_sinensis.comp16662_c0_seq1_m.54393	PREDICTED: beta-glucosidase BoGH3B-like [Prunus mume]	13.029	15.962	37.8	4	5	4	1.012	1.03	1.052	0.981	0.97	0.995	0.952	2.18E-02
cds.Camellia_sinensis.comp16665_c1_seq1_m.38592	PREDICTED: kirola-like [Sesamum indicum]	20.034	135.19	51.4	9	31	9	0.969	0.963	0.975	1.042	1.017	1.037	1.065	1.58E-03
cds.Camellia_sinensis.comp16671_c0_seq1_m.25835	PREDICTED: chaperonin CPN60-2, mitochondrial [Theobroma cacao]	43.716	168.18	54.2	22	49	15	0.98	0.954	0.962	1.049	1.045	1.041	1.083	6.56E-04

cds.Camellia_sinensis.comp16680_c0_seq1_m.14842	PREDICTED: pentatricopeptide repeat-containing protein At3g02490, mitochondrial [Vitis vinifera]	73.699	4.7248	3.2	2	2	2	1.013	1.014	1.058	1.055	0.907	0.961	0.947	2.95E-01
cds.Camellia_sinensis.comp16688_c0_seq1_m.25660	PREDICTED: pyridoxal kinase [Nicotiana attenuata]	37.385	20.742	19.8	7	10	7	1.04	0.934	0.976	1.014	1.032	1.022	1.04	2.73E-01
cds.Camellia_sinensis.comp16722_c0_seq1_m.19086	PREDICTED: BRO1 domain-containing protein BROX [Citrus sinensis]	47.506	9.2122	13.1	4	5	4	1.047	0.989	0.964	0.985	1.046	0.973	1.001	9.68E-01
cds.Camellia_sinensis.comp16730_c0_seq1_m.27278	PREDICTED: annexin D1 [Populus	35.899	127.46	47.2	16	37	16	0.96	0.961	0.937	1.053	1.068	1.061	1.113	3.05E-04
cds.Camellia_sinensis.comp16741_c0_seq1_m.53860	PREDICTED: CBS domain-containing protein CBSX3, mitochondrial [Citrus sinensis]	11.542	11.159	15.1	1	2	1	0.886	0.875	0.923	1.111	1.12	1.129	1.252	1.80E-04

cds.Camellia_sinensis.comp16765_c0_seq1_m.35556	PREDICTED: probable bifunctional TENA-E protein [Solanum tuberosum	26.374	1.4183	6.5	2	2	2	0.986	0.959	0.987	0.991	1.057	1.035	1.052	7.80E-02
cds.Camellia_sinensis.comp16766_c0_seq1_m.28858	PREDICTED: FAD-dependent urate hydroxylase isoform X1 [Solanum pennellii]	47.061	93.521	28.8	10	18	10	0.912	0.919	0.938	1.101	1.103	1.073	1.183	1.61E-04
cds.Camellia_sinensis.comp16769_c0_seq1_m.31560	PREDICTED: mediator of RNA polymerase II transcription subunit 36a-like [Daucus carota subsp. sativus]	33.21	52.822	26.7	8	16	8	1.033	1.058	1.037	0.971	0.95	0.975	0.926	2.14E-03
cds.Camellia_sinensis.comp16770_c0_seq1_m.38569	PREDICTED: protein SPIRAL1-like 1 [Populus euphratica]	11.306	72.789	46.1	3	7	3	1.12	1.016	1.052	0.922	0.948	0.917	0.874	1.16E-02

cds.Camellia_sinensis.comp16800_c0_seq1_m.15124	PREDICTED: protein INVOLVED IN DE NOVO 2 [Solanum tuberosum	73.091	4.6843	6.6	3	3	3								
cds.Camellia_sinensis.comp16821_c0_seq1_m.45046	PREDICTED: peptidyl-prolyl cis-trans isomerase E [Nicotiana attenuata]	20.846	25.661	32.8	5	5	5	1.041	0.967	1.014	1.028	0.956	1.016	0.993	8.25E-01
cds.Camellia_sinensis.comp168375_c0_seq1_m.48819	PREDICTED: G-type lectin S-receptor-like serine/threonine-protein kinase SD2-5 isoform X1 [Vitis vinifera]	17.803	2.5583	6.9	1	1	1	0.965	0.992	1.072	0.993	1.041	0.95	0.985	7.39E-01
cds.Camellia_sinensis.comp16853_c0_seq1_m.35026	PREDICTED: mini-ribonuclease 3 [Capsicum annuum]	26.541	1.8457	4.3	1	1	1								

cds.Camellia_sinensis.comp16868_c0_seq1_m.32972	PREDICTED: nuclear transcription factor Y subunit C-1-like [Solanum pennellii]	30.353	4.0613	10	3	3	2								
cds.Camellia_sinensis.comp16881_c0_seq1_m.43054	PREDICTED: protein ALUMINUM SENSITIVE 3 [Juglans regia]	21.705	4.3573	15.7	2	2	2								
cds.Camellia_sinensis.comp16928_c0_seq1_m.14871	PREDICTED: external alternative NAD(P)H-ubiquinone oxidoreductase B2, mitochondrial-like [Juglans	66.127	29.291	22.5	12	13	11	0.991	0.968	0.945	1.019	1.068	1.041	1.077	1.83E-02
cds.Camellia_sinensis.comp16963_c0_seq1_m.38523	PREDICTED: ycf3-interacting protein 1, chloroplastic [Nelumbo nucifera]	30.199	57.802	17.5	4	9	4	1.225	1.268	1.168	0.741	0.855	0.751	0.641	9.77E-04

cds.Camellia_sinensis.comp16965_c1_seq1_m.43577	PREDICTED: photosynthetic NDH subunit of lumenal location 2, chloroplastic [Vitis vinifera]	18.219	41.309	39.5	5	6	5	0.999	1.003	1.045	0.985	1.012	0.951	0.968	2.24E-01
cds.Camellia_sinensis.comp16976_c0_seq1_m.36981	PREDICTED: heat shock cognate 70 kDa protein 2-like [Phoenix dactylifera]	39.107	81.65	47.6	19	57	6	1.05	1.008	0.982	0.971	0.967	1.007	0.969	2.48E-01
cds.Camellia_sinensis.comp16981_c0_seq1_m.10785	PREDICTED: ABC transporter F family member 5 [Vitis vinifera]	57.2	6.22	2	1	1	1	1.046	1.057	1.083	1.037	0.867	0.905	0.882	7.91E-02
cds.Camellia_sinensis.comp16981_c0_seq1_m.10786	PREDICTED: ABC transporter F family member 5 [Ziziphus jujuba]	26.967	5.4578	14.9	3	4	3	1.024	1.017	0.976	1.081	0.908	1.001	0.991	8.41E-01

cds.Camellia_sinensis.comp17055_c0_seq1_m.38022	PREDICTED: serine carboxypeptidase II-2 [Fragaria vesca subsp. vesca]	32.13	7.1604	10.1	2	2	2	0.891	0.884	1.062	1.151	1.152	0.907	1.131	2.91E-01
cds.Camellia_sinensis.comp17061_c0_seq1_m.30094	pantothenate kinase [Medicago truncatula]	42.208	44.734	20.3	10	12	10	0.961	0.996	0.961	1.033	1.023	1.024	1.056	1.17E-02
cds.Camellia_sinensis.comp17087_c0_seq1_m.36448	PREDICTED: vacuolar protein sorting-associated protein 29 [Setaria italica]	11.371	2.7415	8.1	1	2	1	0.97	0.996	0.923	1.057	1.036	1.039	1.084	2.48E-02
cds.Camellia_sinensis.comp17135_c0_seq1_m.34251	PREDICTED: peroxidase 64 [Nelumbo nucifera]	37.084	33.723	19.5	6	8	6	1.019	1.057	1.073	0.919	0.932	0.938	0.886	1.82E-03
cds.Camellia_sinensis.comp17139_c0_seq1_m.20770	PREDICTED: pinin [Sesamum indicum]	32.316	2.0729	3.9	1	1	1								

cds.Camellia_sinensis.comp17147_c0_seq1_m.50824	PREDICTED: BRISC and BRCA1-A complex member 1 [Cucumis	14.116	1.6234	6.2	1	1	1	0.981	1.022	1.042	0.998	1.016	0.952	0.974	3.74E-01
cds.Camellia_sinensis.comp17169_c0_seq1_m.46387	PREDICTED: mannan endo-1,4-beta-mannosidase 7-like [Nelumbo nucifera]	22.309	2.4031	9.7	1	1	1								
cds.Camellia_sinensis.comp17175_c0_seq1_m.36851	PREDICTED: protein LOW PSII ACCUMULATION 1, chloroplastic [Nelumbo nucifera]	38.066	17.386	9.3	3	5	3	1.008	0.984	0.979	1.05	0.984	1.028	1.031	2.30E-01
cds.Camellia_sinensis.comp17237_c0_seq1_m.50048	PREDICTED: protein SIEVE ELEMENT OCCLUSION B-like [Juglans regia]	21.273	25.063	22	3	5	3	0.931	0.917	0.969	1.213	1.02	1.134	1.195	2.94E-02

cds.Camellia_sinensis.comp17242_c0_seq1_m.26782	PREDICTED: PI-PLC X-box domain-containing protein DDB_G0293730 [Ziziphus jujuba]	36.317	4.9145	8.5	3	5	3	1.012	1.033	1.001	0.972	1	0.991	0.973	9.10E-02
cds.Camellia_sinensis.comp17285_c0_seq1_m.21253	PREDICTED: aspartic proteinase-like protein 2 [Nelumbo nucifera]	54.216	1.438	1.4	1	1	1	1.002	1.056	1.125	0.952	0.923	0.938	0.884	2.41E-02
cds.Camellia_sinensis.comp17289_c0_seq1_m.33204	PREDICTED: proteasome subunit alpha type-5 [Juglans regia]	25.983	122.46	40.5	9	28	9	0.999	0.98	0.959	1.034	1.031	1.045	1.059	1.03E-02
cds.Camellia_sinensis.comp17316_c0_seq1_m.34900	PREDICTED: coatomer subunit beta-1 [Cucumis sativus]	33.782	3.8181	3.4	1	2	1	1.024	1.035	1.042	0.938	0.945	1.013	0.934	5.10E-02
cds.Camellia_sinensis.comp173194_c0_seq1_m.53015	PREDICTED: peroxidase A2-like [Juglans regia]	17.197	63.203	28.5	4	9	4	0.969	0.886	0.886	1.105	1.134	1.071	1.208	5.16E-03

cds.Camellia_sinensis.comp17320_c0_seq30_m.36333	PREDICTED: DNA repair RAD52-like protein 2, chloroplastic [Ipomoea nil]	28.007	42.357	25.7	6	14	6	0.95	0.982	1.024	1.009	1.036	1.016	1.036	2.01E-01
cds.Camellia_sinensis.comp17327_c0_seq1_m.47678	PREDICTED: triphosphate tunel metalloenzyme 3-like [Capsicum annuum]	14.817	1.5128	6	1	1	1	0.953	1.053	0.964	1.034	1.019	0.995	1.026	4.68E-01
cds.Camellia_sinensis.comp17330_c0_seq1_m.25747	PREDICTED: transmembrane protein 184 homolog DDB_G0279555 [Ricinus communis]	34.047	1.7759	2.7	1	2	1	0.917	1.029	1.017	0.993	1.033	1.03	1.031	4.55E-01
cds.Camellia_sinensis.comp17389_c0_seq1_m.5176	PREDICTED: 26S proteasome non-ATPase regulatory subunit 2 homolog A [Prunus mume]	97.952	237.79	32.4	24	37	24	1.001	1.014	1.024	1.003	0.983	1	0.983	1.25E-01

cds.Camellia_sinensis.comp17393_c0_seq1_m.55351	PREDICTED: galactokinase isoform X2 [Gossypium raimondii]	14.78	1.4127	17.2	3	3	2	0.97	0.981	0.96	0.964	1.027	1.111	1.066	2.75E-01
cds.Camellia_sinensis.comp17394_c1_seq14_c1_seq1_m.57350	PREDICTED: thioredoxin H-type [Prunus mume]	13.475	10.011	15.7	2	9	1	0.938	0.951	0.969	1.058	1.134	1.035	1.129	1.43E-02
cds.Camellia_sinensis.comp17413_c0_seq1_m.32892	PREDICTED: bark storage protein A [Vitis vinifera]	40.465	11.414	3.8	1	1	1	1.034	0.89	0.911	0.908	1.083	1.182	1.119	2.95E-01
cds.Camellia_sinensis.comp17414_c0_seq1_m.7290	PREDICTED: subtilisin-like protease SBT1.4 [Nicotiana tabacum]	96.47	70.889	14.4	11	25	11	1.036	1.068	1.037	0.965	0.956	0.943	0.912	1.54E-03
cds.Camellia_sinensis.comp17446_c0_seq1_m.36742	PREDICTED: probable methyltransferase PMT2 [Sesamum indicum]	28.568	2.1685	3.1	1	1	1	1.239	1.309	1.203	0.721	0.729	0.723	0.579	2.08E-03

cds.Camellia_sinensis.comp17454_c0_seq1_m.52470	PREDICTED: mitochondrial import inner membrane translocase subunit Tim9 [Erythranthe guttata]	12.886	38.635	60.7	6	15	6	0.983	1.022	1.036	1.007	1.018	0.986	0.99	6.23E-01
cds.Camellia_sinensis.comp17457_c0_seq1_m.39054	PREDICTED: protein YLS3 [Vitis vinifera]	20.165	1.9	7.4	1	1	1								
cds.Camellia_sinensis.comp17491_c0_seq1_m.52292	PREDICTED: 28 kDa ribonucleoprotein, chloroplastic [Ziziphus jujuba]	17.716	11.9	8.4	1	4	1	0.984	1.041	0.98	0.958	1.034	1.01	0.999	9.72E-01
cds.Camellia_sinensis.comp17538_c0_seq2_m.4420	PREDICTED: glycine--tRNA ligase, chloroplastic/mitochondrial 2 isoform X1 [Vitis vinifera]	122.03	62.461	13.4	13	15	13	0.957	0.986	1.009	1.002	1.023	1.045	1.04	1.15E-01

cds.Camellia_sinensis.comp17559_c0_seq1_m.32084	PREDICTED: dehydrogenase/reductase SDR family member 12 [Prunus mume]	35.364	24.506	22.3	8	9	8	1.007	0.973	0.997	0.984	1.001	0.991	1	9.83E-01
cds.Camellia_sinensis.comp17573_c0_seq1_m.30215	PREDICTED: eukaryotic translation initiation factor 4B2-like [Juglans regia]	37.334	12.135	17.4	5	5	3	1.357	1.197	1.342	0.656	0.678	0.675	0.516	9.51E-05
cds.Camellia_sinensis.comp17573_c0_seq2_m.32838	PREDICTED: eukaryotic translation initiation factor 4B2-like [Juglans regia]	37.239	2.9224	11.9	4	4	2	1.211	1.176	1.074	0.822	0.809	0.857	0.719	1.20E-03
cds.Camellia_sinensis.comp17619_c0_seq1_m.33696	PREDICTED: protein EXORDIUM-like 2 [Nicotiana tomentosiformis]	34.923	1.1819	3.1	1	1	1								

cds.Camellia_sinensis.comp176480_c0_seq1_m.50697	PREDICTED: retrovirus-related Pol polyprotein from transposon TNT 1-94 isoform X7 [Daucus carota subsp.sativus]	20.037	1.494	4.7	1	1	1	0.952	0.777	0.823	0.974	1.476	1.044	1.369	1.02E-01
cds.Camellia_sinensis.comp17650_c0_seq1_m.41675	FAM210B-like protein [Medicago truncatula]	21.997	46.705	18.2	3	4	3	1.096	1.012	0.983	0.906	0.974	0.996	0.93	1.72E-01
cds.Camellia_sinensis.comp17652_c0_seq1_m.47249	PREDICTED: non-specific lipid-transfer protein 2-like [Nicotiana tabacum]	16.696	11.88	23.9	3	3	3	1.04	1.041	1.067	1.008	0.888	0.953	0.905	5.48E-02
cds.Camellia_sinensis.comp17723_c0_seq1_m.35916	PREDICTED: mRNA-decapping enzyme-like protein [Daucus carota subsp. sativus]	39.075	1.862	3.1	1	1	1								

cds.Camellia_sinensis.comp17725_c0_seq1_m.42198	PREDICTED: protein argonaute 4A-like [Juglans regia]	21.705	8.487	14.4	2	3	2	1.144	1.123	1.093	0.955	0.756	0.898	0.776	2.24E-02
cds.Camellia_sinensis.comp17755_c0_seq1_m.43934	PREDICTED: 14-3-3-like protein [Juglans regia]	21.218	72.505	68.3	10	28	8	1.034	1.005	0.889	1.04	1.017	1.043	1.059	2.77E-01
cds.Camellia_sinensis.comp17761_c0_seq1_m.35615	PREDICTED: uncharacterized methyltransferase At1g78140, chloroplastic isoform X1 [Juglans regia]	35.471	6.772	10.7	3	4	3	0.906	1.039	0.869	1.023	1.129	1.064	1.143	9.06E-02
cds.Camellia_sinensis.comp17833_c0_seq1_m.45194	DNA-binding family protein [Populus trichocarpa]	17.9	68.535	27	4	6	3	0.969	1.002	0.959	1.042	1.004	1.046	1.055	4.44E-02

cds.Camellia_sinensis.comp17862_c0_seq1_m.51544	PREDICTED: plasma membrane-associated cation-binding protein 1-like isoform X1 [Nicotiana tomentosiformis]	14.382	74.525	39	6	17	6	0.972	0.913	0.941	1.051	1.07	1.057	1.125	3.34E-03
cds.Camellia_sinensis.comp17865_c0_seq1_m.38533	PREDICTED: caffeoyl-CoA O-methyltransferase isoform X1 [Ricinus communis]	26.74	3.3912	7.2	2	3	2	0.981	0.943	0.942	1.031	1.095	1.024	1.099	2.00E-02
cds.Camellia_sinensis.comp17870_c0_seq1_m.40532	PREDICTED: psbP domain-containing protein 2, chloroplastic [Prunus mume]	27.21	49.923	20	4	10	4	0.971	0.976	0.949	1.03	1.045	1.059	1.082	2.58E-03

cds.Camellia_sinensis.comp17872_c0_seq1_m.38423	PREDICTED: peptidyl-prolyl cis-trans isomerase CYP18-2 [Vigna radiata var. radiata]	18.195	15.309	29.3	4	4	4	1.004	1.011	0.985	0.982	1.026	1.001	1.003	8.55E-01
cds.Camellia_sinensis.comp17904_c0_seq1_m.26470	PREDICTED: aconitate hydratase, cytoplasmic [Sesamum indicum]	37.957	95.837	45.2	16	40	8	0.968	0.993	0.919	1.044	1.052	1.048	1.092	6.12E-02
cds.Camellia_sinensis.comp17913_c0_seq1_m.31582	PREDICTED: probable choline kinase 1 [Sesamum indicum]	25.128	3.1171	3.7	1	2	1	0.903	0.961	0.976	1.017	1.106	1.067	1.123	2.68E-02
cds.Camellia_sinensis.comp17964_c0_seq1_m.38271	PREDICTED: probable plastid-lipid-associated protein 13, chloroplastic [Juglans regia]	36.09	110.56	41	11	28	11	0.985	0.955	0.963	1.03	1.042	1.05	1.075	2.52E-03

cds.Camellia_sinensis.comp17975_c0_seq1_m.28168	PREDICTED: protein AIG1 [Jatropha curcas]	38.3	12.175	9.4	3	3	3	0.91	0.965	0.949	1.107	1.021	1.081	1.136	1.27E-02
cds.Camellia_sinensis.comp18017_c1_seq1_m.55054	ATP synthase gamma chain 1 family protein [Populus trichocarpa]	15.19	55.873	44.5	6	16	4	1.094	1.046	1.046	0.919	0.932	0.914	0.868	9.16E-04
cds.Camellia_sinensis.comp18036_c0_seq1_m.53688	ferritin-2, chloroplastic [Jatropha curcas]	16.137	33.967	23.2	3	8	3	0.956	1.022	1.04	0.929	0.99	1.067	0.989	8.19E-01
cds.Camellia_sinensis.comp18056_c0_seq1_m.41640	PREDICTED: AP-4 complex subunit sigma [Ipomoea nil]	17.976	8.4773	7.9	1	2	1	0.968	1.01	0.946	1.032	1.047	1.015	1.058	5.50E-02
cds.Camellia_sinensis.comp18073_c0_seq1_m.53732	PREDICTED: basic endochitinase [Eucalyptus grandis]	16.084	60.577	34	3	9	3	0.893	0.904	0.931	1.138	1.107	1.092	1.223	3.16E-04
cds.Camellia_sinensis.comp18087_c0_seq1_m.16587	Transmembrane protein 87A [Morus notabilis]	59.375	2.3081	3.2	2	2	2								

cds.Camellia_sinensis.comp18089_c0_seq1_m.28576	PREDICTED: methylthioribose-1-phosphate isomerase [Populus euphratica]	41.504	41.181	22	8	13	8	0.946	0.958	0.931	1.082	1.039	1.009	1.104	1.07E-02
cds.Camellia_sinensis.comp181019_c0_seq1_m.53257	PREDICTED: UDP-glycosyltransferase 73C2 [Vitis vinifera]	17.497	1.9577	7.7	1	1	1								
cds.Camellia_sinensis.comp18152_c0_seq1_m.49380	PREDICTED: hypersensitive-induced response protein 2 [Theobroma cacao]	18.171	3.8593	30.7	5	5	4	0.911	0.953	0.938	1.068	1.063	1.095	1.151	9.38E-04
cds.Camellia_sinensis.comp18157_c0_seq1_m.44976	PREDICTED: cytochrome c [Arachis ipaensis]	12.331	11.21	38.4	4	8	4	1.04	0.98	0.984	1.026	1.001	0.976	1	9.95E-01
cds.Camellia_sinensis.comp18188_c0_seq1_m.33943	PREDICTED: probable ribosome-binding factor A, chloroplastic [Theobroma cacao]	25.487	3.8121	10.2	2	3	2	1.153	1.153	1.158	0.927	0.792	0.874	0.749	2.39E-02

cds.Camellia_sinensis.comp182174_c0_seq1_m.46675	PREDICTED: D-3-phosphoglycerate dehydrogenase 3, chloroplastic [Vitis vinifera]	20.892	4.3712	10.1	2	4	1	0.95	0.939	0.984	1.031	0.993	1.125	1.096	8.39E-02
cds.Camellia_sinensis.comp18218_c0_seq1_m.53618	PREDICTED: ATP-dependent zinc metalloprotease FTSH 11, chloroplastic/mitochondrial-like [Nelumbo nucifera]	16.927	8.9212	19.2	2	2	2								
cds.Camellia_sinensis.comp18252_c0_seq1_m.24814	PREDICTED: protein tas-like [Nicotiana tabacum]	48.881	71.7	29.9	12	24	12	0.965	0.939	0.96	1.059	1.059	1.027	1.098	2.10E-03
cds.Camellia_sinensis.comp18262_c0_seq1_m.23547	PREDICTED: rubisco accumulation factor 1, chloroplastic [Juglans regia]	46.625	44.25	23.2	9	14	9	1.007	0.999	1.037	0.986	0.981	1.013	0.979	2.39E-01

cds.Camellia_sinensis.comp18275_c0_seq1_m.34006	PREDICTED: ribulose-phosphate 3-epimerase, cytoplasmic isoform [Juglans regia]	23.882	55.258	16.7	3	7	3	1.018	1.002	0.975	0.975	1.053	1.047	1.027	4.04E-01
cds.Camellia_sinensis.comp18302_c0_seq1_m.26529	PREDICTED: protein PTST, chloroplastic [Vitis vinifera]	34.652	22.395	22.7	8	10	8	0.998	1.052	1.032	0.966	1.004	1.016	0.969	2.18E-01
cds.Camellia_sinensis.comp18333_c0_seq1_m.25382	PREDICTED: putative hydrolase YtaP [Theobroma cacao]	39.856	31.934	29.7	9	12	9	1.055	1.036	1.024	0.943	0.963	0.935	0.912	1.72E-03
cds.Camellia_sinensis.comp18340_c0_seq1_m.45325	PREDICTED: nudC domain-containing protein 2 [Vitis vinifera]	16.52	3.266	11.3	2	2	2	1.013	0.998	0.996	1.019	1.005	0.979	0.999	9.16E-01
cds.Camellia_sinensis.comp18343_c0_seq1_m.1417	photosystem II CP43 chlorophyll apoprotein (chloroplast) [Actinidia deliciosa]	51.821	237.61	32.3	14	78	14	1.006	1.003	1.016	0.991	0.999	0.989	0.985	3.66E-02

cds.Camellia_sinensis.comp18343_c0_seq1_m.1418	photosystem II protein D2 (chloroplast) [Erythranthe lutea]	39.549	151.14	25.2	7	71	7	0.964	0.986	0.975	1.016	1.03	1.007	1.044	9.80E-03
cds.Camellia_sinensis.comp18377_c0_seq1_m.650	PREDICTED: nuclear-pore anchor isoform X1 [Vitis vinifera]	235.5	21.868	6.1	13	13	13	1.082	1.093	1.149	0.897	0.84	0.852	0.779	7.82E-04
cds.Camellia_sinensis.comp18428_c0_seq1_m.45049	PREDICTED: protochlorophyllide reductase [Vitis vinifera]	18.192	18.879	40	7	11	5	0.959	0.948	0.949	1.03	1.081	1.071	1.114	2.12E-03
cds.Camellia_sinensis.comp18441_c0_seq1_m.49795	PREDICTED: tubulin beta chain [Ricinus communis]	14.998	4.0042	11.1	1	2	1	1.17	1.161	1.167	0.852	0.799	0.806	0.702	3.26E-03
cds.Camellia_sinensis.comp18455_c0_seq1_m.34968	transmembrane protein, putative [Medicago truncatula]	26.104	-2	7	2	2	2								

cds.Camellia_sinensis.comp18460_c0_seq1_m.14574	PREDICTED: leukotriene A-4 hydrolase homolog isoform X1 [Nelumbo nucifera]	68.846	52.409	22.5	12	18	12	0.962	0.955	0.94	1.062	1.029	1.064	1.104	1.50E-03
cds.Camellia_sinensis.comp18462_c0_seq2_m.21658	PREDICTED: CDK5RAP1-like protein [Ipomoea nil]	39.829	1.7968	2.6	1	1	1	1.025	0.964	0.973	1.128	0.93	0.996	1.031	6.66E-01
cds.Camellia_sinensis.comp18478_c0_seq1_m.19072	plant inositol phosphorylceramide synthase [Medicago truncatula]	54.099	1.3204	1.5	1	1	1	0.969	1.082	1.112	0.928	0.934	0.975	0.897	7.50E-02
cds.Camellia_sinensis.comp18488_c0_seq1_m.48004	PREDICTED: protein yippee-like At4g27745 [Sesamum indicum]	12.123	8.056	23.6	2	3	2	1.037	0.952	1.056	0.997	0.993	0.97	0.972	4.55E-01
cds.Camellia_sinensis.comp18510_c0_seq1_m.49141	PREDICTED: dnaJ homolog 1, mitochondrial-like [Sesamum	21.984	2.0803	19	3	4	1								

cds.Camellia_sinensis.comp18512_c0_seq1_m.27140	PREDICTED: protein SLOW GREEN 1, chloroplastic [Cucumis sativus]	35.31	18.754	15.8	6	8	6	1.013	1.048	1.06	0.976	0.951	0.914	0.91	1.59E-02
cds.Camellia_sinensis.comp18556_c0_seq1_m.41305	PREDICTED: probable glutathione S-transferase [Jatropha curcas]	28.249	11.49	14.9	3	5	3	1.029	0.803	0.971	1.115	0.958	1.07	1.121	2.49E-01
cds.Camellia_sinensis.comp18571_c0_seq1_m.11928	PREDICTED: peroxisomal acyl-coenzyme A oxidase 1-like [Nelumbo nucifera]	74.191	134.07	26.7	18	33	18	0.999	0.992	0.998	1.011	0.99	0.995	1.002	7.48E-01
cds.Camellia_sinensis.comp18585_c0_seq1_m.48894	PREDICTED: high mobility group B protein 1 [Vigna radiata var. radiata]	13.703	3.5973	10.9	1	1	1	0.938	1.009	0.974	1.043	1.054	1.005	1.062	7.74E-02

cds.Camellia_sinensis.comp18602_c0_seq1_m.17410	PREDICTED: aldehyde dehydrogenase family 3 member F1-like [Nicotiana sylvestris]	54.591	21.556	12.7	6	7	5	1.152	1.168	1.139	0.856	0.831	0.792	0.717	1.45E-04
cds.Camellia_sinensis.comp18626_c0_seq1_m.19241	PREDICTED: LL-diaminopimelate aminotransferase, chloroplastic-like [Juglans regia]	50.211	64.002	23.3	9	18	9	1.003	1	1.005	1.019	0.992	0.981	0.995	6.79E-01
cds.Camellia_sinensis.comp18674_c0_seq1_m.44439	PREDICTED: histone H1 [Jatropha curcas]	22.575	6.4584	16.4	3	6	3	0.936	0.907	0.951	1.124	1.127	1.095	1.198	4.17E-04
cds.Camellia_sinensis.comp18704_c0_seq1_m.42749	PREDICTED: auxin-binding protein T85 [Juglans regia]	23.213	1.3899	5.9	1	1	1								
cds.Camellia_sinensis.comp18707_c0_seq1_m.44851	maternal effect embryo arrest protein, putative [Medicago truncatula]	21.877	5.5153	9.9	2	3	2	1.001	0.962	0.976	1.002	0.994	0.993	1.017	2.28E-01

cds.Camellia_sinensis.comp18719_c0_seq1_m.23468	PREDICTED: UBX domain-containing protein 4 [Vitis vinifera]	46.727	33.359	22.1	8	13	8	1.061	1.061	1.045	0.953	0.931	0.955	0.896	3.39E-04
cds.Camellia_sinensis.comp18724_c0_seq1_m.36095	PREDICTED: DNA-damage-repair/toleration protein DRT111, chloroplastic [Vitis vinifera]	22.122	2.7568	13.2	2	2	2	0.905	1.536	0.842	0.875	0.845	0.98	0.822	4.58E-01
cds.Camellia_sinensis.comp18726_c0_seq1_m.54824	PREDICTED: leucine aminopeptidase 2, chloroplastic-like [Erythranthe guttata]	13.911	11.898	56.1	7	12	3	0.955	0.982	0.981	1.058	1.039	1.036	1.074	3.20E-03
cds.Camellia_sinensis.comp18746_c0_seq2_m.39329	PREDICTED: stem-specific protein TSJT1-like [Daucus carota subsp. sativus]	27.702	10.723	16.3	3	4	3	0.823	0.992	0.811	1.265	1.082	1.143	1.329	2.25E-02

cds.Camellia_sinensis.comp18795_c0_seq1_m.49744	PREDICTED: acyl-coenzyme A thioesterase 9, mitochondrial-like isoform X2 [Sesamum indicum]	15.618	5.7794	8.1	1	2	1	0.956	1.029	1.02	0.956	1.029	1.022	1.001	9.85E-01
cds.Camellia_sinensis.comp18839_c0_seq1_m.18683	PREDICTED: 4-coumarate--CoA ligase-like 5 [Sesamum indicum]	59.674	81.874	25.3	10	13	10	0.952	0.967	0.979	1.022	1.035	0.996	1.053	2.01E-02
cds.Camellia_sinensis.comp18855_c0_seq1_m.21765	isocitrate dehydrogenase family protein [Populus trichocarpa]	47.749	319.14	48.8	22	63	19	0.987	0.971	0.958	1.027	1.04	1.028	1.061	3.32E-03
cds.Camellia_sinensis.comp18859_c0_seq3_m.27103	PREDICTED: protein LOW PSII ACCUMULATION 1, chloroplastic-like isoform X2 [Malus domestica]	34.093	28.674	8.1	2	3	2	0.983	1.035	1.049	1.014	1.076	0.949	0.991	8.19E-01

cds.Camellia_sinensis.comp18859_c0_seq3_m.27104	PREDICTED: protein LOW PSII ACCUMULATION 1, chloroplastic isoform X2 [Ziziphus jujuba]	25.304	36.274	31.4	7	13	7	1	0.997	1.006	1.003	1.001	0.96	0.987	4.13E-01
cds.Camellia_sinensis.comp18882_c0_seq1_m.9549	high chlorophyll fluorescence phenotype 173 [Arabidopsis thaliana]	67.559	24.091	14.3	9	10	9	1.061	1.022	1.111	0.938	0.945	0.903	0.872	8.18E-03
cds.Camellia_sinensis.comp18903_c0_seq1_m.19833	Succinyl-CoA ligase [ADP-forming] subunit beta [Morus notabilis]	45.919	208.93	48.9	20	50	20	1.017	1.012	0.989	0.977	1.004	0.987	0.983	2.27E-01
cds.Camellia_sinensis.comp18909_c0_seq1_m.37086	DUF1499 family protein [Medicago truncatula]	24.841	19.971	16.4	3	5	3	0.974	0.944	0.979	1.005	1.002	1.088	1.068	8.88E-02

cds.Camellia_sinensis.comp18914_c0_seq1_m.37497	PREDICTED: ER membrane protein complex subunit 8/9 homolog [Daucus carota subsp. sativus]	28.247	40.867	27.5	6	8	6	1.003	0.95	0.968	1.034	1.054	1.027	1.066	2.19E-02
cds.Camellia_sinensis.comp18919_c0_seq1_m.59329	PREDICTED: 4-coumarate--CoA ligase-like 9 [Glycine max]	10.726	19.238	28	2	2	2								
cds.Camellia_sinensis.comp18932_c0_seq1_m.32317	PREDICTED: ATP synthase subunit gamma, mitochondrial-like [Pyrus x bretschneideri]	35.44	121.97	42.4	13	37	13	1.071	1.05	1.01	0.943	0.963	0.955	0.914	7.92E-03
cds.Camellia_sinensis.comp18936_c0_seq1_m.23704	PREDICTED: malonyl-CoA-acyl carrier protein transacylase, mitochondrial [Sesamum indicum]	44.298	39.715	24.9	9	17	9	1	1.009	1.056	0.965	0.976	0.965	0.948	3.74E-02

cds.Camellia_sinensis.comp18938_c0_seq1_m.40311	Heat shock 70 kDa protein [Morus notabilis]	32.963	7.7704	28.2	9	25	1	1.299	1.277	1.335	0.661	0.684	0.653	0.511	1.90E-06
cds.Camellia_sinensis.comp18945_c0_seq1_m.40452	PREDICTED: 50S ribosomal protein L27, chloroplastic [Nelumbo nucifera]	20.981	3.3878	5.7	1	1	1	1.088	1.033	1.089	0.977	0.996	0.809	0.867	9.86E-02
cds.Camellia_sinensis.comp18951_c0_seq1_m.49344	PREDICTED: chaperonin CPN60-2, mitochondrial [Nelumbo nucifera]	21.297	81.819	41.7	7	21	5	0.961	0.979	0.948	1.046	1.036	1.045	1.083	1.26E-03
cds.Camellia_sinensis.comp18993_c0_seq1_m.44402	PREDICTED: dCTP pyrophosphatase 1-like [Jatropha curcas]	14.966	1.7745	7.5	1	1	1	0.922	0.986	0.96	1.151	0.951	1.062	1.103	1.79E-01
cds.Camellia_sinensis.comp19039_c0_seq1_m.30178	PREDICTED: glucuronokinase 1-like [Nelumbo nucifera]	39.919	6.2353	6.9	2	2	2								

cds.Camellia_sinensis.comp19060_c0_seq1_m.4720	PREDICTED: phosphoenolpyruvate carboxylase 2-like isoform X3 [Ziziphus jujuba]	110.47	323.31	53.6	55	133	27	1.001	0.997	1.001	1.019	0.999	1.008	1.009	2.04E-01
cds.Camellia_sinensis.comp19077_c0_seq1_m.41235	PREDICTED: ubiquitin-fold modifier-conjugating enzyme 1 [Jatropha curcas]	19.622	3.7751	17.8	3	4	3	1.011	1.007	0.924	1.077	0.956	1.039	1.044	4.03E-01
cds.Camellia_sinensis.comp19093_c0_seq1_m.2096	PREDICTED: protein RETICULATA-RELATED 4, chloroplastic-like [Nelumbo nucifera]	30.657	31.311	28.4	8	24	8	0.983	0.981	0.974	1.003	1.024	1.025	1.039	7.50E-03
cds.Camellia_sinensis.comp19098_c0_seq2_m.32157	PREDICTED: eyes absent homolog 2 isoform X1 [Vitis vinifera]	38.711	1.48	3.5	1	1	1								

cds.Camellia_sinensis.comp19099_c0_seq1_m.55604	PREDICTED: pectinesterase 2.2-like [Juglans regia]	14.265	17.85	29.8	3	5	3	1.037	1	0.985	0.977	1.006	0.949	0.97	2.54E-01
cds.Camellia_sinensis.comp19106_c0_seq1_m.54673	PREDICTED: TVP38/TMEM64 family membrane protein slr0305 [Solanum tuberosum	15.644	10.132	17.7	4	6	4	1.031	1.049	1.045	0.946	0.922	0.976	0.91	5.44E-03
cds.Camellia_sinensis.comp19124_c0_seq1_m.32589	PREDICTED: zinc-finger homeodomain protein 1-like [Nelumbo nucifera]	25.921	1.7656	4.3	1	1	1								
cds.Camellia_sinensis.comp19145_c0_seq1_m.46398	PREDICTED: profilin-1 [Vitis vinifera]	15.464	4.5685	22.7	3	9	1	1.156	1.047	1.031	0.901	0.927	0.911	0.847	1.10E-02
cds.Camellia_sinensis.comp19170_c0_seq1_m.26266	PREDICTED: COP9 signalosome complex subunit 2 [Sesamum	51.367	2.4245	6.6	2	2	2	1.012	1.143	1.135	0.845	0.818	1.025	0.817	6.35E-02

cds.Camellia_sinensis.comp19187_c0_seq1_m.33599	plant/F3H 11-7 protein [Medicago truncatula]	37.918	5.0296	11.1	4	4	4								
cds.Camellia_sinensis.comp19201_c0_seq1_m.34376	Myo28B1, putative [Medicago truncatula]	33.259	1.2155	4	1	1	1								
cds.Camellia_sinensis.comp1924_c0_seq1_m.51405	PREDICTED: probable glutathione S-transferase [Populus euphratica]	20.04	2.3827	10.3	2	2	2								
cds.Camellia_sinensis.comp19292_c0_seq1_m.44318	PREDICTED: selenoprotein H [Vitis vinifera]	18.474	11.985	20.1	4	7	4	1.185	1.132	1.144	0.781	0.844	0.818	0.706	1.96E-04
cds.Camellia_sinensis.comp19327_c0_seq1_m.32266	PREDICTED: plastid-lipid-associated protein, chloroplastic-like [Juglans regia]	37.698	230.66	28.6	10	35	10	0.966	0.946	0.941	1.063	1.042	1.051	1.106	5.18E-04
cds.Camellia_sinensis.comp19342_c0_seq1_m.8600	transmembrane protein [Arabidopsis thaliana]	91.748	12.705	5.8	5	5	5	1.075	1.036	1.096	0.948	0.912	0.92	0.867	2.14E-03

cds.Camellia_sinensis.comp19347_c0_seq1_m.29962	PREDICTED: inositol oxygenase 1-like [Juglans regia]	36.11	9.2103	3.9	1	1	1	1.258	1.238	1.051	0.813	0.773	0.806	0.674	2.78E-03
cds.Camellia_sinensis.comp19413_c0_seq1_m.56055	PREDICTED: mitochondrial import inner membrane translocase subunit TIM10-like [Juglans regia]	14.691	5.7108	13.5	2	2	2	0.951	0.935	0.919	1.025	1.098	1.099	1.149	5.32E-03
cds.Camellia_sinensis.comp19426_c0_seq1_m.51061	PREDICTED: protein DCL, chloroplastic [Prunus mume]	19.488	14.194	6.7	1	1	1	1.292	1.245	1.275	0.674	0.73	0.7	0.552	2.00E-05
cds.Camellia_sinensis.comp19448_c0_seq2_m.39614	PREDICTED: NAD(P)H-quinone oxidoreductase subunit T, chloroplastic [Theobroma cacao]	33.63	11.637	14.8	3	3	3								

cds.Camellia_sinensis.comp19456_c0_seq1_m.42200	PREDICTED: subtilisin-like protease SBT1.7 [Solanum pennellii]	28.352	4.9272	8.7	2	3	2	0.981	1.036	0.966	0.984	1.113	0.933	1.016	8.25E-01
cds.Camellia_sinensis.comp19456_c1_seq1_m.50009	PREDICTED: subtilisin-like protease SBT1.7 [Ricinus communis]	17.152	13.568	14.4	2	4	2	0.97	0.98	0.979	1.035	1.042	1.013	1.055	4.22E-03
cds.Camellia_sinensis.comp19463_c0_seq1_m.40348	PREDICTED: serine hydroxymethyltransferase, mitochondrial [Nelumbo nucifera]	34.043	18.047	30.2	6	20	2	1.1	1.11	1.048	0.935	0.88	0.949	0.848	4.86E-03
cds.Camellia_sinensis.comp19503_c0_seq1_m.33408	PREDICTED: ubiquitin-activating enzyme E1 1 isoform X2 [Vitis vinifera]	45.411	7.1689	11.3	6	7	5	1.006	0.987	1.016	1.012	1.004	0.994	1	9.72E-01

cds.Camellia_sinensis.comp19518_c0_seq1_m.41879	PREDICTED: glycine cleavage system H protein 2, mitochondrial-like isoform X2 [Daucus carota subsp. sativus]	16.799	11.036	18.8	3	7	3	1.063	1.061	1.071	0.94	0.915	0.975	0.886	2.27E-02
cds.Camellia_sinensis.comp19519_c0_seq1_m.29936	PREDICTED: nuclear transcription factor Y subunit B-3 [Vitis vinifera]	21.98	5.425	10.1	2	2	2	0.966	0.949	1.032	0.856	1.078	1.126	1.038	7.43E-01
cds.Camellia_sinensis.comp19526_c0_seq1_m.10489	PREDICTED: protein LUTEIN DEFICIENT 5, chloroplastic isoform X2 [Jatropha curcas]	39.348	4.1567	12.2	3	3	3	0.999	1.057	1.003	0.992	0.921	1.03	0.962	3.53E-01

cds.Camellia_sinensis.comp19526_c0_seq1_m.10490	PREDICTED: 1-aminocyclopropane-1-carboxylate oxidase [Juglans regia]	36.206	33.361	31.2	9	18	9	1.05	1.051	1.094	0.909	0.936	0.934	0.87	1.08E-03
cds.Camellia_sinensis.comp19532_c0_seq1_m.38865	PREDICTED: guanine deaminase isoform X2 [Prunus	24.129	6.119	9.8	2	2	2	1.037	1.176	0.992	0.958	0.846	0.978	0.868	1.08E-01
cds.Camellia_sinensis.comp19546_c0_seq2_m.43914	PREDICTED: ras-related protein RHN1-like isoform X3 [Ziziphus jujuba]	20.218	2.5034	14.1	2	4	1	0.864	1.074	0.882	1.263	0.982	0.983	1.145	2.89E-01
cds.Camellia_sinensis.comp19566_c0_seq1_m.28513	PREDICTED: caffeoylshikimate esterase isoform X1 [Nicotiana attenuata]	38.707	4.4981	7.3	2	2	2	1.002	1.103	0.974	0.91	0.982	1.026	0.948	3.57E-01

cds.Camellia_sinensis.comp19664_c1_seq1_m.22757	PREDICTED: inner membrane protein PPF-1, chloroplastic isoform X2 [Nelumbo nucifera]	43.585	25.629	15.1	5	8	5	1.136	1.121	1.084	0.837	0.825	0.888	0.763	5.19E-04
cds.Camellia_sinensis.comp19683_c0_seq1_m.22038	PREDICTED: LETM1 and EF-hand domain-containing protein 1, mitochondrial [Vitis vinifera]	53.297	3.9721	4.9	2	2	2	1.068	1.053	0.996	1.041	0.891	0.948	0.924	1.82E-01
cds.Camellia_sinensis.comp19699_c0_seq1_m.35253	PREDICTED: peptidyl-prolyl cis-trans isomerase FKBP18, chloroplastic [Sesamum indicum]	24.57	10.477	16.7	4	6	4	1.043	1.073	1.04	0.922	0.985	0.943	0.903	9.38E-03

cds.Camellia_sinensis.comp19702_c0_seq1_m.40855	PREDICTED: peptidyl-prolyl cis-trans isomerase CYP18-1 [Daucus carota subsp. sativus]	21.608	29.916	19	3	4	3	0.952	0.933	0.98	0.969	1.084	1.078	1.093	8.89E-02
cds.Camellia_sinensis.comp19715_c0_seq1_m.24588	PREDICTED: BRCA1-A complex subunit BRE [Capsicum annuum]	43.376	7.1822	11.1	4	4	4	0.94	0.975	0.988	1.085	1.134	0.909	1.078	3.67E-01
cds.Camellia_sinensis.comp19728_c0_seq6_m.10243	PREDICTED: DUF724 domain-containing protein 6 isoform X4 [Vitis vinifera]	36.148	37.362	19.6	6	10	6	0.947	0.959	1.03	1.042	0.99	1.033	1.044	2.28E-01
cds.Camellia_sinensis.comp19751_c0_seq1_m.30726	PREDICTED: pyruvate kinase isozyme G, chloroplastic isoform X2 [Vitis	40.765	20.58	25.7	8	15	8	1.031	1.005	0.997	0.962	1.016	1.011	0.985	5.03E-01

cds.Camellia_sinensis.comp19772_c0_seq1_m.47210	PREDICTED: abscisic acid receptor PYL8 [Vitis vinifera]	22.239	11.666	9.7	2	3	2	0.945	0.939	1.016	1.06	1.039	1.027	1.078	4.84E-02
cds.Camellia_sinensis.comp19851_c0_seq1_m.25609	PREDICTED: coproporphyrinogen-III oxidase 1, chloroplastic [Solanum tuberosum	44.998	128.93	37.2	16	28	16	1.07	1.059	1.073	0.923	0.925	0.936	0.869	1.94E-05
cds.Camellia_sinensis.comp19898_c0_seq1_m.41293	PREDICTED: succinate dehydrogenase assembly factor 2, mitochondrial [Solanum lycopersicum]	23.208	10.405	15.3	3	5	3	1.067	1.009	1.07	0.955	0.919	0.942	0.895	7.56E-03
cds.Camellia_sinensis.comp19929_c0_seq1_m.31347	PREDICTED: clathrin light chain 1-like [Capsicum annuum]	37.317	14.334	9.5	3	3	2	1.269	1.241	1.205	0.735	0.758	0.719	0.595	1.87E-05

cds.Camellia_sinensis.comp19939_c0_seq1_m.14793	PREDICTED: primary amine oxidase [Vitis vinifera]	44.631	72.491	21	8	17	8	0.953	0.968	0.96	1.066	1.052	1.035	1.094	7.36E-04
cds.Camellia_sinensis.comp19939_c0_seq1_m.14794	PREDICTED: primary amine oxidase [Vitis vinifera]	42.04	52.822	17.2	6	11	6	0.961	0.966	0.944	1.083	1.001	1.046	1.09	2.26E-02
cds.Camellia_sinensis.comp19941_c0_seq1_m.6705	PREDICTED: golgin candidate 6 isoform X2 [Jatropha curcas]	101.47	29.054	7.6	6	8	6	1.037	1.035	0.99	0.982	0.926	1.006	0.952	1.59E-01
cds.Camellia_sinensis.comp20002_c0_seq1_m.34779	PREDICTED: zinc finger CCCH domain-containing protein 14-like [Ziziphus jujuba]	31.895	60.409	35.2	8	18	6	0.944	0.98	1.032	0.986	1.009	1.034	1.025	4.43E-01

cds.Camellia_sinensis.comp20012_c0_seq1_m.22176	PREDICTED: ATP-dependent Clp protease proteolytic subunit-related protein 1, chloroplastic-like [Juglans regia]	43.231	58.739	29.7	9	15	9	1.017	1.028	1.013	0.984	0.946	0.981	0.952	2.10E-02
cds.Camellia_sinensis.comp20019_c0_seq1_m.41433	PREDICTED: chaperone protein DnaJ-like [Setaria italica]	14.528	2.5125	8.7	1	1	1	1.009	1.006	1.066	0.936	0.965	1.018	0.947	1.54E-01
cds.Camellia_sinensis.comp20043_c0_seq2_m.7680	PREDICTED: spermatogenesis-associated protein 20 [Prunus mume]	95.843	99.765	28	23	31	23	0.953	0.937	0.929	1.073	1.084	1.104	1.157	2.01E-04
cds.Camellia_sinensis.comp20064_c0_seq1_m.24530	PREDICTED: probable protein phosphatase 2C 9 [Sesamum indicum]	30.483	61.517	54.3	12	18	12	0.94	0.992	0.934	1.089	1.032	1.079	1.117	1.21E-02

cds.Camellia_sinensis.comp20071_c0_seq1_m.17552	PREDICTED: glucose-6-phosphate 1-dehydrogenase 1, chloroplastic [Ricinus communis]	67.78	83.071	31	17	25	12	0.964	0.948	0.988	1.048	1.039	1.029	1.074	5.38E-03
cds.Camellia_sinensis.comp20082_c1_seq1_m.14890	PREDICTED: ketol-acid reductoisomerase, chloroplastic [Juglans regia]	66.766	177.9	25.3	14	44	14	0.977	0.998	1.007	1.02	1.002	1.012	1.017	1.69E-01
cds.Camellia_sinensis.comp20096_c0_seq1_m.15861	PREDICTED: vacuolar fusion protein CCZ1 homolog isoform X2 [Theobroma cacao]	55.404	20.741	15.4	8	10	8	0.887	0.938	0.937	1.117	1.073	1.083	1.185	1.54E-03
cds.Camellia_sinensis.comp20121_c0_seq1_m.29155	PREDICTED: trichohyalin-like [Malus domestica]	19.675	1.2552	4.2	1	1	1	1.082	1.031	1.033	0.882	0.861	1.091	0.901	2.26E-01

cds.Camellia_sinensis.comp20127_c0_seq1_m.15233	PREDICTED: phospho-2-dehydro-3-deoxyheptonate aldolase 2, chloroplastic [Eucalyptus grandis]	57.502	122.44	34.7	18	36	12	1.088	1.055	1.113	0.919	0.918	0.87	0.831	1.50E-03
cds.Camellia_sinensis.comp20145_c0_seq1_m.31992	PREDICTED: uncharacterized aarF domain-containing protein kinase At1g71810, chloroplastic isoform X1[Nelumbo nucifera]	17.901	7.6004	14.6	2	2	2	0.906	0.977	0.942	0.99	1.019	1.191	1.133	1.18E-01

cds.Camellia_sinensis.comp20145_c1_seq1_m.40226	PREDICTED: uncharacterized aarF domain-containing protein kinase At1g71810, chloroplastic-like isoform X3 [Nicotiana tabacum]	29.818	3.4974	7.1	2	3	2	0.921	0.953	0.995	1.005	1.071	1.125	1.116	5.12E-02
cds.Camellia_sinensis.comp20146_c0_seq1_m.59240		10.949	1.5604	12.9	1	1	1								
cds.Camellia_sinensis.comp20165_c0_seq1_m.36124	transmembrane protein, putative [Medicago truncatula]	34.814	8.2128	6.3	2	3	2	0.938	0.908	0.919	1.116	1.124	1.033	1.184	4.22E-03
cds.Camellia_sinensis.comp20176_c0_seq1_m.38992	PREDICTED: myosin heavy chain, muscle [Nicotiana attenuata]	19.655	33.817	17.3	3	4	3	1.028	0.985	0.928	0.997	1.025	1.04	1.041	2.71E-01

cds.Camellia_sinensis.comp20180_c0_seq1_m.9372	PREDICTED: UTP--glucose-1-phosphate uridylyltransferase-like [Juglans regia]	56.669	10.518	4.3	2	10	2	1.018	0.966	0.999	1.001	1.012	1.033	1.021	3.05E-01
cds.Camellia_sinensis.comp20197_c0_seq1_m.39887	PREDICTED: NADH dehydrogenase [ubiquinone] 1 alpha subcomplex subunit 6 [Theobroma cacao]	14.729	44.974	76.9	9	20	9	1.038	1.022	1.02	0.977	1.002	0.979	0.96	1.46E-02
cds.Camellia_sinensis.comp20212_c0_seq1_m.13881	PREDICTED: pentatricopeptide repeat-containing protein At1g02150 [Eucalyptu	62.958	5.9796	6.9	4	5	4	0.92	1.023	1.043	1.058	1.008	0.996	1.025	5.75E-01
cds.Camellia_sinensis.comp20228_c0_seq1_m.42581	PREDICTED: V-type proton ATPase subunit G 1-like [Nelumbo	12.309	33.272	39.1	5	10	5	0.907	0.92	0.837	1.103	1.033	1.106	1.217	5.98E-03

cds.Camellia_sinensis.comp20235_c0_seq1_m.17959	PREDICTED: tryptophan synthase beta chain 1 [Populus euphratica]	55.113	34.523	15.9	6	6	6	1.039	0.994	1.074	0.964	0.973	1.065	0.966	4.20E-01
cds.Camellia_sinensis.comp20326_c0_seq2_m.55870	PREDICTED: puromycin-sensitive aminopeptidase isoform X1 [Vitis vinifera]	14.287	40.178	52.7	5	12	4	0.987	0.932	0.9	1.029	1.072	1.081	1.129	1.70E-02
cds.Camellia_sinensis.comp20332_c0_seq1_m.49077	PREDICTED: 60S ribosomal protein L19-3 [Ziziphus jujuba]	11.977	2.2131	32.7	3	5	1								
cds.Camellia_sinensis.comp20339_c0_seq1_m.25394	PREDICTED: eukaryotic translation initiation factor 4E-1-like [Arachis duranensis]	26.025	3.4247	8.6	2	2	2								
cds.Camellia_sinensis.comp20339_c1_seq1_m.9048	PREDICTED: phospholipase D alpha 1 [Sesamum indicum]	92.214	249.4	35.8	23	57	21	0.923	0.912	0.914	1.095	1.114	1.067	1.192	1.79E-04

cds.Camellia_sinensis.comp20344_c1_seq1_m.50331	seed maturation protein PM35 [Glycine max]	11.078	25.316	37.4	3	4	3	0.938	1.134	0.852	1.004	0.981	1.159	1.075	4.80E-01
cds.Camellia_sinensis.comp20346_c0_seq1_m.45093	PREDICTED: photosynthetic NDH subunit of subcomplex B 3, chloroplastic [Daucus carota subsp. sativus]	21.322	20.289	21.1	3	5	3	1.043	1.062	1.058	0.923	0.939	0.99	0.902	8.84E-03
cds.Camellia_sinensis.comp20347_c0_seq1_m.30439	PREDICTED: cysteine protease RD19A [Ipomoea nil]	40.173	6.8764	9	3	7	2	1.101	1.134	1.055	0.871	0.874	0.886	0.8	4.98E-04
cds.Camellia_sinensis.comp20349_c0_seq1_m.22493	PREDICTED: ferredoxin--NADP reductase, leaf isozyme, chloroplastic [Ricinus communis]	40.573	293.46	56.9	20	103	7	1.043	1.018	0.971	0.982	0.984	0.989	0.975	3.54E-01

cds.Camellia_sinensis.comp20349_c0_seq2_m.32225	ferredoxin--NADP reductase, leaf-type isozyme, chloroplastic [Capsicum annuum]	29.897	17.759	49.5	11	56	2	1.016	1.156	1.074	0.903	0.861	1.054	0.868	1.15E-01
cds.Camellia_sinensis.comp20350_c0_seq1_m.20984	PREDICTED: enolase 1, chloroplastic [Jatropha curcas]	52.778	38.528	8.9	4	6	4	1.068	1.052	1.08	0.976	0.913	0.881	0.866	9.40E-03
cds.Camellia_sinensis.comp20356_c0_seq1_m.38217	PREDICTED: photosynthetic NDH subunit of subcomplex B 5, chloroplastic [Nelumbo nucifera]	25.565	5.5998	11.5	2	3	2	0.981	1.002	1.011	0.978	1.034	1.005	1.008	7.05E-01
cds.Camellia_sinensis.comp20364_c0_seq1_m.41438	heat shock cognate 70 kDa protein 2-like [Ananas comosus]	27.816	33.941	59.8	13	71	2	1.012	0.988	0.988	0.977	1.044	1.003	1.012	6.06E-01

cds.Camellia_sinensis.comp20367_c0_seq1_m.18869	PREDICTED: flowering locus K homology domain isoform X1 [Ricinus communis]	46.807	138.72	33.3	13	28	11	0.962	1.014	0.994	1.018	1.003	0.995	1.015	4.05E-01
cds.Camellia_sinensis.comp20368_c0_seq2_m.30637	PREDICTED: UPF0613 protein PB24D3.06c [Populus euphratica]	27.726	26.399	4.8	1	1	1	1.015	0.934	0.995	0.943	1.134	0.99	1.042	5.60E-01
cds.Camellia_sinensis.comp20369_c0_seq1_m.35674	PREDICTED: thioredoxin-like protein CDSP32, chloroplastic [Nelumbo nucifera]	33.819	205	43.9	13	41	13	1.063	1.04	1.039	0.96	0.928	0.959	0.906	1.82E-03
cds.Camellia_sinensis.comp20371_c0_seq2_m.34391	PREDICTED: probable trans-2-enoyl-CoA reductase, mitochondrial isoform X3 [Juglans	38.069	4.0837	6.6	2	3	2	0.926	0.995	1.04	1.012	1.04	1.008	1.033	3.90E-01

cds.Camellia_sinensis.comp20380_c0_seq1_m.14804	PREDICTED: probable carotenoid cleavage dioxygenase 4, chloroplastic [Nicotiana attenuata]	47.042	65.941	18.3	8	13	8	1.005	1.005	1.021	1.011	0.968	0.965	0.971	1.39E-01
cds.Camellia_sinensis.comp20382_c0_seq1_m.29159	PREDICTED: trafficking protein particle complex subunit 12-like [Vitis	40.524	1.272	2.5	1	1	1								
cds.Camellia_sinensis.comp20385_c0_seq1_m.15507	PREDICTED: zeta-carotene desaturase, chloroplastic/chromoplastic [Vitis vinifera]	64.292	12.08	9.3	5	6	5	1.057	1.056	0.933	0.9	0.896	0.987	0.914	1.60E-01
cds.Camellia_sinensis.comp20387_c0_seq1_m.31718	PREDICTED: flavonol synthase/flavanone 3-hydroxylase-like [Erythranthe guttata]	38.417	15.974	6.2	2	2	2	0.95	1.008	0.948	1.026	1.046	1.043	1.072	2.92E-02

cds.Camellia_sinensis.comp20390_c0_seq1_m.30556	PREDICTED: protein SRC2 [Vitis vinifera]	30.677	2.8683	3.5	1	1	1	0.976	0.976	0.982	1.036	1.031	1.019	1.052	6.84E-04
cds.Camellia_sinensis.comp20401_c0_seq1_m.19860	PREDICTED: kynurenine--oxoglutarate transaminase [Jatropha curcas]	36.248	7.9103	14.9	5	6	5	0.976	0.977	1.076	0.971	0.997	0.99	0.977	5.32E-01
cds.Camellia_sinensis.comp20401_c0_seq1_m.19861	PREDICTED: probable N-succinyldiaminopimelate aminotransferase DapC [Gossypium hirsutum]	22.039	29.962	32.8	5	7	5	1.031	0.971	1.011	1.011	0.95	1.021	0.99	7.32E-01
cds.Camellia_sinensis.comp20402_c0_seq1_m.28705	PREDICTED: titin-like [Fragaria vesca subsp. vesca]	19.986	4.7872	15.1	3	5	3	1.118	1.203	1.17	0.851	0.85	0.771	0.708	9.00E-04

cds.Camellia_sinensis.comp20403_c0_seq1_m.21322	oligouridylate-binding protein 1B-like [Cajanus	46.646	25.619	12.9	5	8	4	0.979	0.951	1.003	1.051	1.025	0.93	1.025	5.92E-01
cds.Camellia_sinensis.comp20404_c0_seq1_m.31114	PREDICTED: beta-carotene hydroxylase 2, chloroplastic [Vitis vinifera]	39.932	9.2243	5.6	1	1	1	0.749	0.985	0.955	1.074	1.044	1.246	1.251	9.00E-02
cds.Camellia_sinensis.comp20406_c0_seq2_m.22795	PREDICTED: heterogeneous nuclear ribonucleoprotein R [Sesamum indicum]	47.84	5.6169	5.7	2	2	2	0.949	0.988	1.108	1.032	0.924	1.013	0.975	6.95E-01
cds.Camellia_sinensis.comp20407_c0_seq1_m.12567	PREDICTED: ALBINO3-like protein 1, chloroplastic [Vitis vinifera]	59.727	42.745	8.1	4	7	4	1.016	0.97	1.074	0.981	0.968	0.947	0.946	1.56E-01

cds.Camellia_sinensis.comp20412_c0_seq1_m.33374	PREDICTED: proteasome subunit alpha type-6 [Musa acuminata subsp. malaccensis]	27.384	94.253	54.9	15	27	15	0.97	0.98	0.944	1.043	1.042	1.056	1.085	2.38E-03
cds.Camellia_sinensis.comp20418_c0_seq1_m.35269	PREDICTED: tropinone reductase homolog At5g06060-like isoform X1 [Nelumbo nucifera]	29.741	8.531	12	4	11	2	0.972	0.94	0.981	1.064	1.003	1.062	1.082	2.84E-02
cds.Camellia_sinensis.comp20423_c0_seq1_m.35425	PREDICTED: rhodanese-like domain-containing protein 14, chloroplastic [Theobroma cacao]	25.321	57.434	31.3	7	14	7	0.99	0.997	0.993	1.009	1.002	0.998	1.01	6.34E-02
cds.Camellia_sinensis.comp20429_c0_seq1_m.24635	PREDICTED: ultraviolet-B receptor UVR8 [Sesamum indicum]	43.849	16.632	16.7	7	9	7	0.985	1	1.022	1.002	0.993	1.025	1.004	7.76E-01

cds.Camellia_sinensis.comp20430_c0_seq1_m.35205	PREDICTED: 6,7-dimethyl-8-ribityllumazine synthase, chloroplastic [Ipomoea nil]	27.433	9.152	10.2	4	6	4	0.986	0.93	0.951	1.071	1.056	1.037	1.104	7.06E-03
cds.Camellia_sinensis.comp20433_c0_seq1_m.46290	hydrogen peroxide induced protein, putative [Medicago truncatula]	14.815	29.97	25.2	2	2	2	1.04	1.033	0.967	0.992	1.031	0.941	0.975	5.09E-01
cds.Camellia_sinensis.comp20436_c0_seq1_m.16763	PREDICTED: splicing factor SF3a60 homolog [Juglans regia]	61.133	19.596	11.2	5	5	5	1.084	1.119	1.126	0.871	0.892	0.851	0.785	1.78E-04
cds.Camellia_sinensis.comp20438_c0_seq1_m.37911	PREDICTED: outer envelope pore protein 24A, chloroplastic-like [Nelumbo nucifera]	25.464	14.584	28.3	7	9	7	1.036	0.996	1.014	1.006	0.983	0.985	0.976	1.54E-01

cds.Camellia_sinensis.comp20441_c0_seq3_m.33434	RING-H2 zinc finger protein [Arabidopsis thaliana]	31.331	4.4074	6.1	1	2	1	1.022	1.15	1.041	1.072	0.714	0.993	0.865	2.89E-01
cds.Camellia_sinensis.comp20446_c0_seq1_m.28374	PREDICTED: transcription factor DIVARICATA-like [Juglans regia]	33.955	10.11	6	2	2	2	1.087	1.005	1.057	1.023	0.906	0.916	0.903	8.53E-02
cds.Camellia_sinensis.comp20451_c0_seq1_m.36083	PREDICTED: salicylic acid-binding protein 2 isoform X1 [Vitis vinifera]	29.648	41.836	29.7	6	13	6	0.962	1.003	0.983	1.074	1.021	1.041	1.064	3.16E-02
cds.Camellia_sinensis.comp20457_c0_seq1_m.38890	PREDICTED: 22.0 kDa class IV heat shock protein-like [Solanum pennellii]	21.783	12.871	23.4	5	5	5	1.14	1.035	1.042	0.906	0.956	0.899	0.858	1.42E-02
cds.Camellia_sinensis.comp20458_c0_seq1_m.27298	PREDICTED: SEC14 cytosolic factor [Jatropha curcas]	14.124	2.8167	9.4	1	1	1								

cds.Camellia_sinensis.comp20463_c0_seq1_m.19640	PREDICTED: probable serine protease EDA2 [Ziziphus jujuba]	54.281	109.9	25.5	10	20	10	1.016	1.029	1.012	0.938	1.006	0.992	0.96	1.37E-01
cds.Camellia_sinensis.comp20464_c0_seq1_m.30668	PREDICTED: acyl-protein thioesterase 2 [Arachis duranensis]	27.2	4.8794	11.4	4	5	4	1.084	0.983	1.03	0.95	0.967	0.988	0.938	1.06E-01
cds.Camellia_sinensis.comp20467_c0_seq1_m.38457	PREDICTED: probable N-acetyltransferase san [Juglans regia]	19.308	1.8495	5.9	1	1	1	1.027	1.069	1.025	0.921	0.984	0.967	0.92	2.58E-02
cds.Camellia_sinensis.comp20469_c0_seq1_m.23763	PREDICTED: RAN GTPase-activating protein 1 [Vitis vinifera]	50.407	26.233	13.4	5	6	5	1.136	1.092	1.042	0.905	0.932	0.905	0.839	2.82E-03
cds.Camellia_sinensis.comp20474_c0_seq1_m.33064	PREDICTED: GTP-binding nuclear protein Ran-3 [Camelina sativa]	26.072	85.929	38.1	8	20	8	0.999	0.963	0.987	0.998	1.038	1.031	1.04	7.32E-02

cds.Camellia_sinensis.comp20478_c0_seq1_m.32271	PREDICTED: reticulon-like protein B1 [Ricinus communis]	33.99	15.592	17.9	6	8	6	0.991	0.982	1	0.974	1.042	1.026	1.023	3.45E-01
cds.Camellia_sinensis.comp20486_c0_seq1_m.10822	PREDICTED: glutamate--tRNA ligase, chloroplastic/mitochondrial [Ziziphus jujuba]	44.706	15.758	17.9	7	11	7	1.046	0.99	1.041	1.001	0.973	0.965	0.955	9.30E-02
cds.Camellia_sinensis.comp20486_c0_seq1_m.10823	PREDICTED: glutamate--tRNA ligase, chloroplastic/mitochondrial [Ipomoea nil]	32.179	7.6481	3.1	1	1	1	1.006	1.07	1.102	0.956	0.935	0.927	0.887	1.35E-02
cds.Camellia_sinensis.comp20488_c0_seq1_m.22800	PREDICTED: TOM1-like protein 2 [Vitis vinifera]	44.052	62.16	40.1	16	22	16	0.972	0.987	0.981	1.026	1.031	1.014	1.045	2.80E-03

cds.Camellia_sinensis.comp20489_c0_seq1_m.16840	PREDICTED: ribose-phosphate pyrophosphokinase 1 [Theobroma cacao]	46.525	21.156	7.5	3	4	2	0.964	0.984	0.981	0.946	0.98	1.039	1.012	7.03E-01
cds.Camellia_sinensis.comp20489_c0_seq2_m.22330	PREDICTED: ribose-phosphate pyrophosphokinase 1 [Juglans regia]	16.247	30.19	26	3	3	2	0.893	1.007	0.978	1.085	1.071	0.999	1.096	1.05E-01
cds.Camellia_sinensis.comp20490_c0_seq1_m.37682	PREDICTED: U2 small nuclear ribonucleoprotein B" [Ipomoea nil]	29.103	1.5748	6.1	2	2	2	1.068	1.045	1.064	0.957	0.977	0.882	0.886	1.90E-02
cds.Camellia_sinensis.comp20491_c0_seq1_m.30669	PREDICTED: palmitoyl-protein thioesterase 1 [Prunus	34.868	12.475	9.5	3	4	3	0.999	0.921	1.002	1.023	1.014	1.09	1.07	1.30E-01
cds.Camellia_sinensis.comp20492_c0_seq1_m.1946	putative serine/threonine-protein kinase dyrk2 [Morus notabilis]	133.28	7.2607	1.5	1	1	1	1.264	1.312	1.033	0.733	0.772	0.812	0.642	5.38E-03

cds.Camellia_sinensis.comp20498_c0_seq1_m.16852	PREDICTED: non-specific phospholipase C1 isoform X1 [Vitis vinifera]	37.641	14.439	12.4	4	6	3	1.108	1.066	1.092	0.885	0.88	0.891	0.813	6.15E-05
cds.Camellia_sinensis.comp20504_c0_seq1_m.22343	PREDICTED: elongation factor Tu, chloroplastic [Juglans regia]	54.717	323.31	52.8	23	115	21	1.033	1.017	1.001	0.986	0.975	0.976	0.963	1.76E-02
cds.Camellia_sinensis.comp20504_c0_seq2_m.31348	PREDICTED: ATP-dependent Clp protease proteolytic subunit-related protein 4, chloroplastic [Prunus mume]	33.119	67.173	22.7	5	16	5	1.031	1.004	1.01	0.983	0.987	0.996	0.974	4.24E-02
cds.Camellia_sinensis.comp20509_c0_seq1_m.40649	tail fiber [Arabidopsis thaliana]	24.764	1.7292	4	1	1	1	0.988	0.961	1.005	0.958	1.048	1.05	1.035	3.70E-01

cds.Camellia_sinensis.comp20515_c0_seq1_m.41744	PREDICTED: pentatricopeptide repeat-containing protein At1g62350 [Beta vulgaris subsp. vulgaris]	29.148	6.2141	8.2	2	2	2								
cds.Camellia_sinensis.comp20529_c0_seq1_m.34425	PREDICTED: activating signal cointegrator 1-like [Nicotiana tabacum]	28.621	2.825	7	2	2	2	0.944	0.978	1	1.058	1.036	1.007	1.061	5.41E-02
cds.Camellia_sinensis.comp20534_c0_seq1_m.34889	PREDICTED: stem-specific protein TSJT1 [Daucus carota subsp. sativus]	27.284	11.146	24	7	10	7	0.981	0.979	0.984	1.038	1.049	1.002	1.049	7.53E-02
cds.Camellia_sinensis.comp20535_c0_seq2_m.43958	PREDICTED: nifU-like protein 3, chloroplastic [Nicotiana tomentosiformis]	17.897	14.895	17.7	2	4	2	1.045	1.001	1.038	0.986	0.976	1.042	0.974	3.37E-01

cds.Camellia_sinensis.comp20538_c0_seq1_m.37612	PREDICTED: small heat shock protein, chloroplastic-like [Juglans regia]	32.824	43.486	23.8	7	16	7	1.078	1.071	1.037	0.951	0.941	0.915	0.881	1.60E-03
cds.Camellia_sinensis.comp20539_c0_seq1_m.30179	PREDICTED: ATP-dependent Clp protease proteolytic subunit 3, chloroplastic-like [Nicotiana tabacum]	39.032	93.776	18.2	6	10	6	1.012	1.016	1.006	0.994	1.011	0.976	0.983	1.69E-01
cds.Camellia_sinensis.comp20545_c0_seq1_m.27639	PREDICTED: short-chain dehydrogenase reductase 2a [Theobroma cacao]	41.456	1.597	3.1	1	1	1	0.872	1.16	1.051	0.98	0.965	0.986	0.951	6.54E-01
cds.Camellia_sinensis.comp20551_c0_seq1_m.32591	PREDICTED: photosystem I chlorophyll a/b-binding protein 3-1, chloroplastic [Malus domestica]	29.762	153.04	48.9	10	64	10	0.996	0.948	0.947	1.021	1.069	1.037	1.082	2.14E-02

cds.Camellia_sinensis.comp20552_c0_seq1_m.15501	PREDICTED: ATP sulfurylase 2 [Vitis vinifera]	44.513	33.222	26.1	10	18	9	0.994	0.984	0.979	1.029	0.985	1.018	1.025	1.47E-01
cds.Camellia_sinensis.comp20559_c0_seq1_m.21086	PREDICTED: protein bem46 [Vitis vinifera]	35.166	9.5137	7.9	2	2	2	1.026	1.019	0.974	1.022	0.981	0.984	0.989	6.41E-01
cds.Camellia_sinensis.comp20562_c0_seq1_m.18527	UTP--glucose-1-phosphate uridylyltransferase [Ananas comosus]	61.834	242.88	44.9	21	66	21	0.976	0.969	0.984	1.014	1.037	1.027	1.051	3.24E-03
cds.Camellia_sinensis.comp20563_c0_seq1_m.32355	PREDICTED: annexin D2-like [Jatropha curcas]	25.672	5.4555	14.7	3	3	3	1.048	1.005	1.013	1.006	0.91	1.016	0.956	2.89E-01
cds.Camellia_sinensis.comp20568_c0_seq1_m.47712	PREDICTED: 60S ribosomal protein L35-like [Gossypium hirsutum]	16.123	8.0699	24.8	5	5	2								
cds.Camellia_sinensis.comp20572_c0_seq1_m.45714	PREDICTED: vesicle transport v-SNARE 12 [Prunus mume]	18.709	5.5645	17.7	4	4	4	1.036	1.079	1.044	0.934	0.905	0.993	0.896	2.15E-02

cds.Camellia_sinensis.comp20590_c0_seq1_m.31254	PREDICTED: pectinesterase 31 [Vitis vinifera]	37.035	7.4034	11	3	3	3	0.956	0.998	0.948	0.985	1.043	1.088	1.074	9.87E-02
cds.Camellia_sinensis.comp20591_c0_seq1_m.37440	PREDICTED: single-stranded DNA-bindig protein WHY2, mitochondrial-like isoform X3 [Nicotiana tabacum]	28.229	7.3975	11	3	4	3	0.951	0.96	1	0.958	1.012	1.092	1.052	2.95E-01
cds.Camellia_sinensis.comp20592_c0_seq2_m.49303	PREDICTED: 40S ribosomal protein S23 [Arachis ipaensis]	14.037	5.4191	9.5	2	3	2	1.02	0.998	1.045	0.947	0.957	1.033	0.959	2.34E-01
cds.Camellia_sinensis.comp20608_c0_seq1_m.16069	PREDICTED: T-complex protein 1 subunit eta [Nelumbo nucifera]	60.742	78.299	30.5	15	22	15	0.951	0.915	0.917	1.059	1.08	1.064	1.151	5.24E-04
cds.Camellia_sinensis.comp20612_c0_seq1_m.52175	PREDICTED: protein argonaute 4 [Vitis vinifera]	18.614	3.692	42.5	6	9	1								

cds.Camellia_sinensis.comp20614_c0_seq1_m.31139	PREDICTED: proline synthase co-transcribed bacterial homolog protein isoform X1 [Vitis vinifera]	30.587	14.091	16.8	5	7	4	1.005	0.981	0.973	1.005	1.039	1.009	1.032	9.41E-02
cds.Camellia_sinensis.comp20616_c0_seq1_m.32068	PREDICTED: oxygen-evolving enhancer protein 1, chloroplastic [Ziziphus jujuba]	35.011	323.31	52.7	21	149	21	1.019	0.965	0.982	0.997	1.015	1.014	1.02	3.01E-01
cds.Camellia_sinensis.comp20617_c0_seq1_m.35166	nucleic acid-binding, OB-fold-like protein [Medicago truncatula]	17.371	3.8574	19.3	4	5	3	0.949	0.97	0.957	1.117	1.027	1.008	1.096	4.81E-02
cds.Camellia_sinensis.comp20617_c0_seq2_m.40687	nucleic acid-binding, OB-fold-like protein [Medicago truncatula]	20.852	11.63	20.4	4	9	3	0.953	0.956	0.96	1.016	1.052	1.088	1.1	4.11E-02

cds.Camellia_sinensis.comp20618_c0_seq1_m.37751	PREDICTED: ubiquitin-conjugating enzyme E2 27-like [Populus euphratica]	21.23	8.4106	16	3	3	3	0.906	0.971	1.048	1.132	0.957	1.017	1.062	4.10E-01
cds.Camellia_sinensis.comp20621_c0_seq1_m.25426	PREDICTED: AT-hook motif nuclear-localized protein 8 isoform X1 [Vitis vinifera]	36.627	16.336	3.9	1	1	1	1.063	1.015	1.043	0.985	0.969	0.921	0.921	2.75E-02
cds.Camellia_sinensis.comp20622_c0_seq1_m.34048	glycine-rich RNA-binding family protein [Populus trichocarpa]	31.011	10.65	6.8	2	3	2	1.238	1.288	1.278	0.667	0.746	0.716	0.56	7.79E-05
cds.Camellia_sinensis.comp20624_c0_seq1_m.11184	vestitone reductase-related family protein [Populus trichocarpa]	44.537	111.49	29.7	12	28	12	1.02	1.019	1.018	0.972	0.979	0.986	0.961	1.06E-02
cds.Camellia_sinensis.comp20625_c0_seq1_m.16923	PREDICTED: protein argonaute 4A [Vitis vinifera]	62.952	41.399	15.8	8	14	8	1.062	1.08	1.083	0.911	0.935	0.905	0.853	1.64E-04

cds.Camellia_sinensis.comp20627_c0_seq1_m.29194	PREDICTED: methionine aminopeptidase 1D, chloroplastic/mitochondrial isoform X1 [Vitis vinifera]	39.177	8.288	10.1	4	5	4	1.028	0.993	1.065	0.946	0.993	0.956	0.938	6.35E-02
cds.Camellia_sinensis.comp20631_c0_seq1_m.25567	PREDICTED: heme-binding protein 2 [Pyrus x bretschneideri]	25.013	71.351	46.5	8	22	8	0.992	1.021	0.967	1.037	0.991	0.998	1.015	5.07E-01
cds.Camellia_sinensis.comp20633_c0_seq1_m.22719	PREDICTED: ubiquitin receptor RAD23c-like isoform X2 [Gossypium raimondii]	41.877	98.504	30.6	9	16	5	1.08	0.933	1.036	0.933	0.988	1.012	0.962	4.93E-01
cds.Camellia_sinensis.comp20634_c0_seq1_m.34445	PREDICTED: nitrogen regulatory protein P-II homolog isoform X2 [Theobroma cacao]	21.593	16.839	27.9	6	9	6	0.978	0.984	1.001	1.011	1	1.024	1.024	6.98E-02

cds.Camellia_sinensis.comp20635_c0_seq1_m.28179	PREDICTED: RNA-binding protein CP29B, chloroplastic [Prunus mume]	29.591	186.76	47.8	11	38	11	1.001	0.998	0.994	0.995	0.998	1.014	1.005	4.97E-01
cds.Camellia_sinensis.comp20635_c0_seq2_m.39693	PREDICTED: 29 kDa ribonucleoprotein A, chloroplastic-like [Nelumbo nucifera]	17.576	13.773	13	4	13	4	0.978	0.992	0.981	1.035	0.999	1.049	1.045	4.56E-02
cds.Camellia_sinensis.comp20637_c0_seq1_m.49637	PREDICTED: heterogeneous nuclear ribonucleoprotein 1 isoform X2 [Gossypium arboreum]	10.588	2.6968	9.7	1	1	1								
cds.Camellia_sinensis.comp20648_c0_seq1_m.55086	PREDICTED: 14-3-3-like protein D isoform X2 [Vitis vinifera]	15.517	2.1677	10.9	2	4	2	1.002	0.984	1.016	1.006	0.995	1.006	1.002	8.70E-01

cds.Camellia_sinensis.comp20649_c0_seq1_m.31482	PREDICTED: 2-keto-3-deoxy-L-rhamnonate aldolase-like [Solanum tuberosum	38.631	7.8286	9.8	3	4	3	1.031	1.046	1.074	0.889	0.9	1.005	0.887	4.06E-02
cds.Camellia_sinensis.comp20654_c0_seq1_m.39964	PREDICTED: LDLR chaperone MESD [Vitis vinifera]	28.854	14.722	18.9	5	9	5	1.093	1.116	1.05	0.934	0.9	0.885	0.834	1.64E-03
cds.Camellia_sinensis.comp20665_c0_seq1_m.20922	PREDICTED: enolase [Sesamum indicum]	52.402	303.44	49.8	21	68	15	0.984	0.965	0.932	1.048	1.052	1.047	1.092	3.09E-02
cds.Camellia_sinensis.comp20673_c0_seq1_m.13833	PREDICTED: endoplasmin homolog [Ziziphus alba	72.803	107.79	27.7	16	33	16	0.953	0.964	0.963	1.025	1.059	1.059	1.091	1.60E-03
cds.Camellia_sinensis.comp20678_c0_seq1_m.44148	DNA/RNA-binding protein [Medicago truncatula]	22.119	6.4583	19.6	5	8	1	0.929	1.005	1.05	0.995	0.968	1.069	1.016	7.43E-01

cds.Camellia_sinensis.comp20681_c0_seq1_m.43982	PREDICTED: NAD(P)H-quinone oxidoreductase subunit S, chloroplastic isoform X1 [Theobroma cacao]	18.151	54.107	45.5	7	14	7	0.991	0.994	1.016	1.025	1.028	1.002	1.018	1.89E-01
cds.Camellia_sinensis.comp20685_c0_seq2_m.34273	PREDICTED: sec-independent protein translocase protein TATB, chloroplastic isoform X1 [Vitis vinifera]	29.162	45.476	30.1	7	11	7	0.995	0.999	0.98	0.975	1.019	0.98	1	9.92E-01
cds.Camellia_sinensis.comp20686_c0_seq1_m.38674	PREDICTED: acyl carrier protein 1, chloroplastic [Vitis vinifera]	16.625	4.9406	9.7	1	4	1	1.018	0.948	1.063	0.946	1.057	1.014	0.996	9.37E-01

cds.Camellia_sinensis.comp20687_c0_seq2_m.46943	PREDICTED: flowering locus K homology domain-like isoform X1 [Nicotiana tabacum]	20.68	9.5695	10.4	2	3	2	0.997	1.03	1.043	0.991	0.952	0.991	0.956	7.53E-02
cds.Camellia_sinensis.comp20690_c0_seq2_m.20996	PREDICTED: peptidyl-prolyl cis-trans isomerase, chloroplastic [Ziziphus jujuba]	48.218	223.3	44.1	17	71	17	0.983	0.988	1.002	1.016	1.031	1.014	1.03	1.98E-02
cds.Camellia_sinensis.comp20693_c0_seq1_m.16158	PREDICTED: 2,3-bisphosphoglycerate-independent phosphoglycerate mutase [Sesamum indicum]	60.685	138.69	37.9	23	64	23	1.025	1.009	1.013	0.983	1.003	0.979	0.973	3.67E-02
cds.Camellia_sinensis.comp20696_c0_seq1_m.14529	PREDICTED: transmembrane protein 214 [Nelumbo nucifera]	67.561	10.572	9.6	5	5	5	1.08	1.093	1.085	0.896	0.913	0.911	0.835	1.63E-05

cds.Camellia_sinensis.comp20697_c0_seq1_m.39788	transmembrane protein, putative [Medicago truncatula]	19.409	6.1872	9.2	2	2	2	0.839	0.926	0.893	1.129	1.139	1.127	1.277	1.37E-02
cds.Camellia_sinensis.comp20698_c0_seq1_m.44790	PREDICTED: universal stress protein PHOS32 [Theobroma cacao]	17.911	29.132	34.8	6	7	5	1.01	0.986	0.989	1.052	0.941	1.036	1.015	7.25E-01
cds.Camellia_sinensis.comp20699_c0_seq1_m.26825	alpha/beta fold hydrolase [Medicago truncatula]	43.135	2.959	7.6	2	2	2	1.096	0.949	1.053	0.949	1.071	0.878	0.935	3.97E-01
cds.Camellia_sinensis.comp207013_c0_seq1_m.50709	PREDICTED: vinorine synthase-like [Nicotiana tabacum]	20.017	52.278	26.2	4	8	4	1.1	1.1	1.152	0.873	0.92	0.846	0.787	1.16E-03
cds.Camellia_sinensis.comp20701_c0_seq1_m.31979	PREDICTED: ABC transporter I family member 20 isoform X1 [Vitis vinifera]	36.665	18.931	8.5	2	4	2	0.979	0.979	1.054	0.977	1.03	0.964	0.986	6.93E-01

cds.Camellia_sinensis.comp20705_c0_seq1_m.39969	PREDICTED: inositol polyphosphate multikinase beta [Capsicum annuum]	27.911	12.609	5.6	1	2	1	1.003	1.013	0.934	0.994	0.969	1.095	1.037	4.79E-01
cds.Camellia_sinensis.comp20709_c0_seq1_m.51241	DNA-binding family protein [Populus trichocarpa]	17.137	42.84	45.2	9	19	8	0.927	0.911	0.886	1.087	1.082	1.125	1.209	4.59E-04
cds.Camellia_sinensis.comp20711_c0_seq1_m.43518	PREDICTED: thioredoxin H2-like [Cicer arietinum]	15.388	24.333	28.5	3	6	3	0.969	1.032	0.932	1.067	0.989	0.995	1.04	3.59E-01
cds.Camellia_sinensis.comp20712_c1_seq1_m.35145	PREDICTED: coatomer subunit epsilon-1 [Ziziphus jujuba]	33.522	2.0324	7.3	2	2	1								
cds.Camellia_sinensis.comp20714_c0_seq3_m.46434	PREDICTED: protein FAM192A [Capsicum annuum]	17.526	1.9464	11.1	1	1	1	0.936	1.159	1.003	0.968	0.987	0.954	0.939	4.52E-01

cds.Camellia_sinensis.comp20717_c0_seq1_m.12353	heat shock cognate 70 kDa protein 2 [Gossypium hirsutum]	71.179	323.31	54.3	36	156	11	1.051	1.029	1.008	0.973	0.973	0.965	0.943	8.90E-03
cds.Camellia_sinensis.comp20726_c0_seq26_m.34782	cyclin delta-3, putative [Medicago truncatula]	38.31	7.057	12.5	5	6	5	0.967	1.003	1.044	1.008	0.945	1.002	0.98	5.50E-01
cds.Camellia_sinensis.comp20737_c0_seq1_m.32462	PREDICTED: UBP1-associated protein 2C [Nelumbo nucifera]	36.101	49.378	19.4	7	11	6	1.001	0.963	1.023	0.975	1.012	1.023	1.008	7.51E-01
cds.Camellia_sinensis.comp20738_c0_seq1_m.25402	PREDICTED: probable plastid-lipid-associated protein 12, chloroplastic [Juglans regia]	48.665	76.647	27.5	11	19	11	1.03	0.998	1.054	0.947	0.992	0.997	0.953	1.00E-01
cds.Camellia_sinensis.comp20742_c0_seq1_m.46842	PREDICTED: 40S ribosomal protein S10-1-like [Gossypium hirsutum]	11.919	46.608	55.6	7	22	6	0.886	0.913	0.95	1.106	1.09	1.054	1.182	2.48E-03

cds.Camellia_sinensis.comp20748_c1_seq1_m.45323	PREDICTED: protein bicaudal C homolog 1-like [Nicotiana attenuata]	15.038	3.3975	8.7	1	1	1								
cds.Camellia_sinensis.comp20751_c0_seq1_m.43541	PREDICTED: probable small nuclear ribonucleoprotein G [Ziziphus jujuba]	12.136	7.049	35.8	4	6	4	0.985	0.941	0.946	1.063	1.01	1.035	1.082	1.87E-02
cds.Camellia_sinensis.comp20756_c0_seq1_m.21710	PREDICTED: SUN domain-containing protein 3-like [Jatropha curcas]	50.359	40.23	29.8	12	15	12	1.02	1.037	1.012	0.962	1.013	0.975	0.961	8.04E-02
cds.Camellia_sinensis.comp20758_c0_seq1_m.42404	PREDICTED: nicotinamidase 2-like [Nelumbo nucifera]	22.435	2.518	12.5	3	3	3	0.955	1.007	0.97	0.983	1.066	1.035	1.052	1.52E-01

cds.Camellia_sinensis.comp20763_c0_seq2_m.44518	PREDICTED: DNA-directed RNA polymerases II, IV and V subunit 11 isoform X1 [Theobroma cacao]	13.569	15.954	34.5	4	7	4	0.967	0.969	0.937	1.042	1.017	1.031	1.076	4.90E-03
cds.Camellia_sinensis.comp20766_c0_seq1_m.23754	PREDICTED: probable sugar phosphate/phosphate translocator At5g25400 [Nelumbo	38.615	1.3266	3.2	1	1	1								
cds.Camellia_sinensis.comp20768_c0_seq1_m.35796	PREDICTED: anthranilate synthase beta subunit 1, chloroplastic-like [Nicotiana tabacum]	33.21	14.393	14	4	8	4	1.008	0.992	1.02	0.995	0.974	0.968	0.973	7.41E-02
cds.Camellia_sinensis.comp20770_c0_seq1_m.3876	elongation factor Ts (DUF810) [Arabidopsis thaliana]	89.098	4.6025	2.1	2	2	2	1.042	1.075	1.025	0.907	0.979	0.963	0.907	2.22E-02

cds.Camellia_sinensis.comp20772_c0_seq1_m.32739	PREDICTED: vestitone reductase-like [Vitis vinifera]	36.6	6.3923	3.9	1	1	1	0.896	0.897	0.851	1.139	1.095	1.167	1.286	5.82E-04
cds.Camellia_sinensis.comp20772_c0_seq2_m.32953	PREDICTED: vestitone reductase-like [Vitis vinifera]	36.68	11.953	6.6	2	2	2								
cds.Camellia_sinensis.comp20774_c0_seq1_m.26562	PREDICTED: 60S acidic ribosomal protein P0 [Vitis vinifera]	34.131	19.249	18.5	5	12	5	1.039	1.04	1.026	0.94	0.969	0.99	0.934	1.17E-02
cds.Camellia_sinensis.comp20777_c0_seq1_m.37508	PREDICTED: RNA-binding protein 7 [Vitis vinifera]	22.001	1.4441	5.6	1	1	1								
cds.Camellia_sinensis.comp20780_c0_seq1_m.41262	PREDICTED: pre-mRNA cleavage factor Im 25 kDa subunit 2 [Citrus sinensis]	22.727	11.722	24.1	4	5	4	0.952	0.959	1.122	1.16	0.883	0.943	0.985	8.52E-01

cds.Camellia_sinensis.comp20783_c0_seq1_m.41641	PREDICTED: peptidyl-prolyl cis-trans isomerase Pin1 isoform X2 [Juglans regia]	12.926	20.673	30.5	4	7	4	0.99	1.041	0.923	1.033	0.978	1.037	1.032	4.63E-01
cds.Camellia_sinensis.comp20786_c0_seq1_m.22268	PREDICTED: magnesium-chelatase subunit ChlI, chloroplastic-like isoform X3 [Juglans	46.468	165.09	42.9	17	35	17	1.005	1.003	1.01	0.991	0.983	0.99	0.982	5.32E-03
cds.Camellia_sinensis.comp20791_c0_seq1_m.16520	PREDICTED: probable zinc metalloprotease EGY2, chloroplastic isoform X2 [Nelumbo nucifera]	56.973	9.562	7.9	4	6	4	0.958	1.041	1.032	0.932	0.991	1.01	0.968	4.12E-01

cds.Camellia_sinensis.comp20803_c0_seq1_m.21237	PREDICTED: branched-chain-amino-acid aminotransferase 3, chloroplastic [Erythranthe guttata]	45.781	56.399	29	13	19	13	1.012	1.005	1.02	0.959	0.98	0.986	0.963	1.64E-02
cds.Camellia_sinensis.comp20807_c0_seq1_m.40489	PREDICTED: elongation factor P-like [Gossypium hirsutum]	24.476	58.747	44.5	9	17	9	1.041	1.06	1.052	0.946	0.944	0.941	0.898	3.96E-05
cds.Camellia_sinensis.comp20815_c0_seq1_m.23773	PREDICTED: cysteine synthase [Vitis vinifera]	41.013	6.1021	12.8	5	7	4	0.961	0.994	1.006	1.058	1.026	0.998	1.041	1.40E-01
cds.Camellia_sinensis.comp20820_c0_seq1_m.38571	PREDICTED: glutathione S-transferase F9-like [Jatropha curcas]	25.679	30.602	29	7	21	7	0.998	0.971	0.962	1.012	1.031	1.003	1.039	4.80E-02

cds.Camellia_sinensis.comp20832_c0_seq1_m.31547	PREDICTED: eukaryotic translation initiation factor 3 subunit G-B [Vitis vinifera]	32.579	43.56	30.7	9	13	7	0.978	0.999	0.964	1.036	1.027	1.017	1.047	1.66E-02
cds.Camellia_sinensis.comp20839_c0_seq2_m.26371	PREDICTED: uncharacterized PKHD-type hydroxylase At1g22950-like [Nelumbo nucifera]	29.493	3.2205	4.3	1	1	1								
cds.Camellia_sinensis.comp20841_c0_seq1_m.9399	PREDICTED: subtilisin-like protease [Nicotiana sylvestris]	82.946	2.3826	4.1	3	3	3	0.969	0.937	1.029	1.065	1.052	0.971	1.052	2.72E-01
cds.Camellia_sinensis.comp20842_c0_seq1_m.13440	PREDICTED: AT-hook motif nuclear-localized protein 9 [Ricinus communis]	34.521	11.057	11.2	3	4	3	1.034	0.939	0.982	0.979	0.953	1.043	1.007	8.69E-01

cds.Camellia_sinensis.comp20845_c1_seq1_m.52039	PREDICTED: importin subunit alpha-1 [Cucumis melo]	18.79	2.0114	18.3	4	4	1	0.957	0.995	0.976	1.015	1.029	1.045	1.055	1.88E-02
cds.Camellia_sinensis.comp20846_c0_seq1_m.30367	PREDICTED: prolyl-tRNA synthetase associated domain-containing protein 1 [Solanum tuberosum	34.965	17.346	32	8	9	8	0.973	0.967	0.936	1.018	1.067	1.045	1.088	9.60E-03
cds.Camellia_sinensis.comp20847_c0_seq1_m.23896	condensation domain protein [Medicago truncatula]	50.77	16.636	14.1	6	6	6	1.03	1.041	1.087	1.016	0.926	0.955	0.917	5.29E-02
cds.Camellia_sinensis.comp20851_c0_seq1_m.29429	PREDICTED: fructose-bisphosphate aldolase, cytoplasmic isozyme 1 [Theobroma cacao]	36.427	140.53	41.1	9	27	9	1.034	1.054	1.02	0.96	0.93	0.981	0.924	1.20E-02
cds.Camellia_sinensis.comp20857_c0_seq2_m.40730	PREDICTED: histone H2A-like [Sesamum indicum]	15.635	1.4511	15.6	3	8	1	1.11	1.094	1.112	0.866	0.903	0.888	0.801	7.69E-05

cds.Camellia_sinensis.comp20858_c0_seq1_m.19553	PREDICTED: ethanolamine-phosphate cytidylyltransferase [Sesamum indicum]	46.774	29.793	8.4	4	5	4	1.009	1.004	1.006	0.975	0.972	1.003	0.977	1.48E-01
cds.Camellia_sinensis.comp20861_c0_seq1_m.45654	transmembrane protein [Arabidopsis thaliana]	12.461	33.199	18.9	2	7	2	0.92	0.983	1.03	1.061	0.931	1.075	1.046	4.82E-01
cds.Camellia_sinensis.comp20862_c0_seq1_m.10575	PREDICTED: dipeptidyl-peptidase 5 [Vitis vinifera]	80.797	105.65	32.5	23	42	23	0.982	0.973	1.019	1.038	1.021	0.988	1.025	2.97E-01
cds.Camellia_sinensis.comp20864_c0_seq1_m.32920	Caffeoyl-CoA O-methyltransferase [Morus notabilis]	27.553	18.841	33.1	7	10	7	1.168	1.096	1.118	0.852	0.881	0.893	0.776	4.19E-04
cds.Camellia_sinensis.comp20865_c0_seq1_m.20015	PREDICTED: switch-associated protein 70 [Vitis vinifera]	52.855	70.111	38	15	24	15	0.971	0.966	0.992	1.025	1.018	1.021	1.046	5.72E-03

cds.Camellia_sinensis.comp20867_c0_seq1_m.15016	Protein kinase capable of phosphorylating tyrosine family protein [Populus trichocarpa]	44.664	1.2566	4.5	2	2	2	1.005	0.975	1.074	1.03	0.894	1.028	0.967	5.55E-01
cds.Camellia_sinensis.comp20873_c0_seq1_m.13915	PREDICTED: synaptotagmin-2-like [Nelumbo nucifera]	61.594	5.0706	12.3	8	13	3	0.952	0.99	0.94	1.038	1.057	1.047	1.09	6.24E-03
cds.Camellia_sinensis.comp20874_c0_seq1_m.44718	dihydroorotate dehydrogenase, putative [Medicago truncatula]	15.902	4.0248	6.9	1	3	1	1.057	0.967	0.918	1.003	1.082	0.98	1.042	4.58E-01
cds.Camellia_sinensis.comp20877_c0_seq1_m.38225	PREDICTED: calcineurin subunit B-like [Daucus carota subsp. sativus]	19.878	9.9636	26.3	4	4	3	1.045	1.032	1.124	1.012	0.92	0.863	0.873	6.21E-02

cds.Camellia_sinensis.comp20881_c0_seq2_m.37337	PREDICTED: abscisic acid receptor PYL8 [Vitis vinifera]	19.935	1.6165	6.2	1	1	1								
cds.Camellia_sinensis.comp20882_c0_seq1_m.11112	PREDICTED: protein TIC 62, chloroplastic [Sesamum indicum]	31.947	55.397	52.8	10	37	6	1.119	1.059	1.04	0.916	0.909	0.924	0.854	2.22E-03
cds.Camellia_sinensis.comp20882_c0_seq2_m.17004	PREDICTED: protein TIC 62, chloroplastic [Populus euphratica]	39.27	323.31	59.1	19	67	15	1.066	1.075	1.059	0.895	0.927	0.947	0.865	1.04E-03
cds.Camellia_sinensis.comp20883_c0_seq1_m.38158	PREDICTED: inosine triphosphate pyrophosphatase isoform X2 [Juglans regia]	22.643	9.507	17	3	3	3	1.003	0.992	0.928	1.057	0.965	1.068	1.057	2.44E-01
cds.Camellia_sinensis.comp20894_c0_seq2_m.33751	Putative syntaxin-131 [Morus notabilis]	15.86	3.1934	17.2	3	3	3	1.115	1.107	1.123	0.877	0.874	0.891	0.79	2.64E-06

cds.Camellia_sinensis.comp20894_c0_seq2_m.33752	PREDICTED: syntaxin-132-like [Prunus mume]	14.651	24.676	10.3	1	2	1	1.043	0.968	1.105	0.948	0.993	0.94	0.925	1.39E-01
cds.Camellia_sinensis.comp20896_c0_seq1_m.33618	PREDICTED: biotin carboxyl carrier protein of acetyl-CoA carboxylase 1, chloroplastic-like [Nicotiana sylvestris]	34.621	24.88	10.5	4	5	4	0.996	1.063	1.021	0.935	0.951	0.96	0.924	1.84E-02
cds.Camellia_sinensis.comp20897_c0_seq2_m.55053	oxidoreductase family protein [Populus trichocarpa]	14.805	1.6042	5.8	1	1	1	0.927	1.014	0.908	1.086	1.068	1.028	1.117	4.13E-02
cds.Camellia_sinensis.comp20901_c0_seq1_m.28740	40S ribosomal protein S2-4 [Medicago truncatula]	30.281	128.94	48	16	33	10	1.096	1.067	1.087	0.927	0.913	0.865	0.832	1.20E-03

cds.Camellia_sinensis.comp20902_c0_seq1_m.42673	PREDICTED: ATP-dependent Clp protease adapter protein CLPS1, chloroplastic-like [Juglans regia]	18.629	2.1974	11.1	3	3	3	0.969	1.039	1.042	0.956	1.034	0.969	0.97	4.22E-01
cds.Camellia_sinensis.comp20904_c0_seq1_m.35646	PREDICTED: psbP-like protein 1, chloroplastic [Sesamum indicum]	28.08	75.084	29.6	9	20	9	1.013	0.993	0.989	0.968	1.023	1.007	1.001	9.67E-01
cds.Camellia_sinensis.comp20914_c0_seq1_m.43257	PREDICTED: dirigent protein 5 [Eucalyptus grandis]	26.43	111.95	29.8	5	22	5	1.03	1.106	1.1	0.926	0.904	0.89	0.841	2.50E-03
cds.Camellia_sinensis.comp20920_c0_seq1_m.53904	PREDICTED: probable inactive receptor kinase RLK902 [Nicotiana attenuata]	16.646	11.416	28.4	3	4	3	0.98	1.024	1.005	1.083	0.909	1.012	0.998	9.42E-01
cds.Camellia_sinensis.comp20921_c0_seq1_m.19574	PREDICTED: ruvB-like 2 [Sesamum indicum]	51.623	74.693	36.8	16	24	16	0.941	0.95	0.995	1.042	1.035	1.058	1.086	1.08E-02

cds.Camellia_sinensis.comp20923_c0_seq1_m.1885	ketose-bisphosphate aldolase class-II family protein [Arabidopsis	148.61	87.697	18.4	23	29	23	0.979	0.959	0.941	1.032	1.055	1.035	1.084	3.72E-03
cds.Camellia_sinensis.comp20930_c0_seq10_c0_seq1_m.34135	transmembrane protein [Arabidopsis thaliana]	19.655	16.86	12.8	1	2	1	1.055	0.944	0.952	0.939	1.03	1.081	1.034	5.83E-01
cds.Camellia_sinensis.comp20933_c0_seq13_c0_seq1_m.35813	YbaK/prolyl-tRNA synthetase-related family protein [Populus trichocarpa]	26.48	8.7232	9.7	2	3	2	0.897	0.951	1.049	1.101	1.034	1.002	1.083	2.04E-01
cds.Camellia_sinensis.comp20935_c0_seq2_m.43476	PREDICTED: KH domain-containing protein At4g18375-like [Sesamum indicum]	24.413	23.783	14.1	3	3	3	0.985	1.067	1.078	1.027	0.963	0.888	0.919	1.70E-01

cds.Camellia_sinensis.comp20936_c0_seq1_m.31963	PREDICTED: tropinone reductase homolog isoform X1 [Sesamum indicum]	33.917	3.5372	9	3	3	2	1.037	1.033	1.008	0.952	0.984	0.985	0.949	2.14E-02
cds.Camellia_sinensis.comp20938_c0_seq1_m.40912	PREDICTED: translationally-controlled tumor protein homolog [Prunus mume]	25.486	28.697	29.1	8	20	5	1.045	0.957	0.989	0.965	1.04	1.016	1.01	7.79E-01
cds.Camellia_sinensis.comp20939_c0_seq1_m.24698	PREDICTED: protein LOW PSII ACCUMULATION 3, chloroplastic [Ziziphus jujuba]	43.645	34.669	23.3	9	12	9	1.095	1.059	1.034	0.941	0.95	0.962	0.895	3.40E-03
cds.Camellia_sinensis.comp20944_c0_seq1_m.33041	PREDICTED: peroxidase 47 [Sesamum indicum]	34.936	131.45	34.8	9	10	9	0.928	0.929	0.88	1.082	1.109	1.101	1.203	6.63E-04

cds.Camellia_sinensis.comp20950_c0_seq1_m.28165	nuclear protein [Arabidopsis thaliana]	33.521	87.907	52.9	14	27	14	1.009	1.02	1.033	0.972	0.997	0.976	0.962	2.00E-02
cds.Camellia_sinensis.comp20951_c0_seq1_m.28527	PREDICTED: oligouridylate-binding protein 1-like [Prunus	39.588	22.353	22.7	8	12	7	0.991	0.971	0.971	1.005	0.993	1.015	1.027	4.43E-02
cds.Camellia_sinensis.comp20958_c0_seq1_m.33512	PREDICTED: heterogeneous nuclear ribonucleoprotein H2 isoform X1 [Vitis vinifera]	29.599	16.925	18.9	4	6	4								
cds.Camellia_sinensis.comp20960_c0_seq1_m.41449	copper ion-binding protein [Medicago truncatula]	12.664	3.4564	12.8	2	5	2	1.04	1.013	1.022	0.957	0.979	0.973	0.946	5.72E-03
cds.Camellia_sinensis.comp20962_c0_seq1_m.8182	PREDICTED: serine/threonine-protein kinase SAPK3 [Eucalyptus grandis]	38.534	46.405	21.6	8	10	5	0.998	1.109	1.006	0.868	0.98	0.947	0.898	9.50E-02

cds.Camellia_sinensis.comp20972_c0_seq1_m.50792	PREDICTED: nectarin-1 [Eucalyptus grandis]	13.072	5.6161	14.5	2	4	2	0.967	0.929	0.884	1.063	1.102	1.084	1.169	5.00E-03
cds.Camellia_sinensis.comp20973_c0_seq1_m.5748	PREDICTED: protein TIC 100 [Solanum pennellii]	98.759	3.4677	1.3	1	1	1	0.903	1.088	1.08	1.015	0.954	0.977	0.959	5.80E-01
cds.Camellia_sinensis.comp20974_c0_seq1_m.17772	PREDICTED: probable nucleoredoxin 1 [Ziziphus jujuba]	64.438	116.53	37.8	19	36	19	0.898	0.887	0.894	1.104	1.126	1.113	1.248	2.60E-06
cds.Camellia_sinensis.comp20976_c0_seq1_m.44117		23.212	16.949	35.8	6	10	6	0.948	0.925	0.927	1.068	1.096	1.126	1.175	7.19E-04
cds.Camellia_sinensis.comp20979_c0_seq1_m.1888	PREDICTED: splicing factor 3B subunit 1 [Vitis vinifera]	122.24	46.564	14.1	14	15	14	1.011	1.089	1.1	0.91	0.94	0.937	0.871	8.52E-03
cds.Camellia_sinensis.comp20979_c0_seq1_m.1889	PREDICTED: splicing factor 3B subunit 1 [Nelumbo nucifera]	23.939	38.865	24	5	6	5	1.091	1.144	1.176	0.906	0.893	0.764	0.751	8.04E-03

cds.Camellia_sinensis.comp20982_c0_seq1_m.38132	PREDICTED: U1 small nuclear ribonucleoprotein A-like isoform X1 [Sesamum indicum]	28.725	6.9912	15.3	3	3	3	0.907	1.004	0.997	1.149	1.015	1.033	1.099	1.37E-01
cds.Camellia_sinensis.comp20992_c0_seq1_m.20718	PREDICTED: WD-40 repeat-containing protein MSI4-like [Juglans regia]	51.128	15.053	12.2	4	7	3	1.044	1.008	0.972	1.018	0.972	0.99	0.985	5.90E-01
cds.Camellia_sinensis.comp21000_c0_seq1_m.31884	PREDICTED: sufE-like protein 1, chloroplastic/mitochondrial [Daucus carota subsp. sativus]	39.422	26.638	19.6	5	6	5	0.941	1.003	1.067	1.033	1.042	0.94	1.001	9.76E-01
cds.Camellia_sinensis.comp21005_c0_seq4_m.24618	PREDICTED: protein phosphatase 2C 57 [Prunus mume]	43.893	95.916	21.8	9	14	9	1.016	1.016	1.05	0.983	0.972	0.976	0.951	1.19E-02

cds.Camellia_sinensis.comp21006_c0_seq1_m.35903	PREDICTED: tetrapyrrole-binding protein, chloroplastic [Juglans regia]	30.146	2.4352	4.5	1	2	1	1.009	1.032	1.001	0.944	1.007	1.009	0.973	3.07E-01
cds.Camellia_sinensis.comp21006_c1_seq1_m.26070	tyrosine phosphatase, putative [Medicago truncatula]	41.261	246.04	51	20	64	20	1.127	1.101	1.119	0.865	0.884	0.876	0.784	1.65E-05
cds.Camellia_sinensis.comp21009_c0_seq1_m.28964	PREDICTED: bifunctional L-3-cyanoalanine synthase/cysteine synthase 1, mitochondrial isoform X2 [Vitis	41.896	86.35	24.1	9	24	8	1.013	1.023	1.016	1.015	0.943	0.978	0.962	2.09E-01
cds.Camellia_sinensis.comp21010_c0_seq1_m.33119	PREDICTED: S-formylglutathione hydrolase [Ipomoea nil]	32.06	129.44	37.7	9	18	9	0.976	0.97	0.986	1.027	1.028	1.057	1.061	4.90E-03

cds.Camellia_sinensis.comp21016_c0_seq1_m.34450	PREDICTED: translocon-associated protein subunit alpha-like [Juglans regia]	24.909	10.233	5.7	1	2	1	1.07	1.097	1.027	0.957	0.891	0.944	0.874	9.88E-03
cds.Camellia_sinensis.comp21017_c0_seq1_m.40078	PREDICTED: ATP-dependent Clp protease ATP-binding subunit CLPT2, chloroplastic [Juglans regia]	15.194	17.81	38.4	5	11	4	1.07	0.946	1.021	0.98	0.99	0.966	0.967	4.23E-01
cds.Camellia_sinensis.comp21018_c0_seq1_m.6880	PREDICTED: histidine--tRNA ligase, cytoplasmic isoform X1 [Nelumbo nucifera]	98.124	66.042	21.7	18	26	18	1.005	0.988	1.002	1.002	0.998	0.973	0.993	5.22E-01
cds.Camellia_sinensis.comp21019_c0_seq1_m.29951	PREDICTED: bromodomain-containing protein 4-like [Ziziphus jujuba]	32.216	119.45	48.4	13	28	13	0.995	0.992	1.008	1.007	1.007	0.988	1.002	7.87E-01

cds.Camellia_sinensis.comp21021_c0_seq2_m.32602	PREDICTED: pre-mRNA-splicing factor ISY1 homolog [Beta vulgaris subsp. vulgaris]	35.444	3.7839	7.9	3	3	3	1.017	1.092	1.047	0.983	0.948	0.912	0.901	2.51E-02
cds.Camellia_sinensis.comp21022_c0_seq1_m.24264	PREDICTED: GDSL esterase/lipase APG-like [Capsicum annuum]	38.148	9.9561	12.9	4	5	4	1.01	1.11	1.039	0.825	1.02	0.983	0.895	1.84E-01
cds.Camellia_sinensis.comp21024_c0_seq1_m.14426	PREDICTED: eukaryotic peptide chain release factor GTP-binding subunit ERF3A-like isoform X2 [Ipomoea nil]	52.283	32.7	24.4	11	19	11	0.986	0.966	0.946	1.028	1.003	1.055	1.065	2.92E-02

cds.Camellia_sinensis.comp21027_c0_seq1_m.39798	PREDICTED: 14 kDa zinc-binding protein-like [Sesamum indicum]	20.492	74.708	28.3	5	14	5	0.976	0.98	0.985	1.02	1.022	1.018	1.04	1.61E-04
cds.Camellia_sinensis.comp21030_c0_seq1_m.29788	PREDICTED: tropinone reductase homolog [Nicotiana attenuata]	24.516	62.669	25.6	5	23	3	0.957	1.006	0.998	1.039	1.029	1.023	1.044	5.54E-02
cds.Camellia_sinensis.comp21036_c0_seq1_m.27186	PREDICTED: grpE protein homolog, mitochondrial [Sesamum indicum]	41.759	90.694	24	9	21	9	1.134	1.11	1.119	0.869	0.876	0.898	0.786	3.63E-05
cds.Camellia_sinensis.comp21039_c0_seq1_m.29908	PREDICTED: 20 kDa chaperonin, chloroplastic-like [Nicotiana tabacum]	21.783	76.983	40.9	9	26	9	1.053	0.985	1.001	0.979	0.994	0.993	0.976	3.12E-01

cds.Camellia_sinensis.comp21042_c0_seq1_m.43112	PREDICTED: ferredoxin-thioredoxin reductase, variable chain [Theobroma cacao]	22.79	105.78	34	7	18	7	1.031	0.988	0.977	0.995	1.002	0.998	1	9.97E-01
cds.Camellia_sinensis.comp21045_c0_seq1_m.37610	PREDICTED: probable plastid-lipid-associated protein 4, chloroplastic [Nicotiana attenuata]	28.484	75.261	39.5	9	21	9	1.005	0.986	1.01	1.012	1.026	0.979	1.005	7.59E-01
cds.Camellia_sinensis.comp21051_c0_seq1_m.38150	PREDICTED: membrane steroid-binding protein 2-like [Arachis duranensis]	23.941	95.77	35	6	15	6	0.958	0.961	0.961	1.016	1.014	1.064	1.074	4.54E-02
cds.Camellia_sinensis.comp21052_c0_seq1_m.35948	PREDICTED: 40S ribosomal protein S10-1-like [Gossypium hirsutum]	20.106	35.307	39.6	7	20	6	0.963	1.008	0.98	0.976	1.01	1.013	1.016	4.17E-01

cds.Camellia_sinensis.comp21053_c0_seq1_m.8628	PREDICTED: U3 small nucleolar RNA-associated protein 4 [Juglans regia]	95.92	1.5142	0.8	1	1	1								
cds.Camellia_sinensis.comp210541_c0_seq1_m.51069	PREDICTED: protein SRG1 [Vitis vinifera]	17.632	1.1946	4.5	1	1	1	0.969	0.998	1.025	1.029	1.04	0.956	1.011	7.51E-01
cds.Camellia_sinensis.comp21054_c0_seq1_m.28357	PREDICTED: protein CHAPERONE-LIKE PROTEIN OF POR1, chloroplastic [Vitis vinifera]	30.851	1.4176	2.9	1	1	1	0.985	0.989	1.074	0.957	0.961	1.041	0.971	4.93E-01
cds.Camellia_sinensis.comp21055_c0_seq1_m.33724	PREDICTED: serine/arginine-rich splicing factor SR30-like isoform X1 [Ipomoea nil]	28.857	9.9276	12.7	3	3	3	0.993	1.065	1.053	0.99	0.965	0.938	0.93	5.34E-02

cds.Camellia_sinensis.comp21056_c0_seq2_m.25852	PREDICTED: 2,3-dimethylmalate lyase isoform X1 [Theobroma cacao]	37.181	181.62	53.7	15	35	15	0.977	0.993	0.978	1.015	1.033	1.021	1.041	5.46E-03
cds.Camellia_sinensis.comp21057_c0_seq1_m.43189	PREDICTED: photosystem I reaction center subunit VI, chloroplastic-like [Populus euphratica]	15.264	11.344	13.2	3	10	3	0.996	0.977	1.02	0.976	1.006	1.004	0.998	8.92E-01
cds.Camellia_sinensis.comp21061_c0_seq1_m.23387	glycerol-3-phosphate acyltransferase 3 [Ricinus communis]	42.707	1.1689	2.7	1	1	1	0.97	1.114	0.955	0.993	0.998	0.978	0.977	7.13E-01
cds.Camellia_sinensis.comp21115_c0_seq1_m.30691	PREDICTED: J domain-containing protein DDB_G0295729-like [Gossypium raimondii]	39.797	2.0973	4.5	2	3	2	0.959	0.938	0.958	1.069	1.07	1.033	1.111	1.52E-03

cds.Camellia_sinensis.comp21134_c0_seq2_m.41738	PREDICTED: single-stranded DNA-binding protein, mitochondrial-like [Nicotiana tabacum]	25.165	5.5732	8.9	2	2	2								
cds.Camellia_sinensis.comp21139_c0_seq1_m.45245	late embryogenesis abundant protein, putative [Medicago truncatula]	18.743	9.1112	18.8	3	6	3	0.984	1.014	1.038	1.008	0.964	1.021	0.986	5.72E-01
cds.Camellia_sinensis.comp21151_c0_seq2_m.38950	PREDICTED: tropomyosin [Theobroma cacao]	30.66	3.5383	5.7	1	1	1								
cds.Camellia_sinensis.comp21152_c0_seq1_m.42139	PREDICTED: osmotin-like protein TPM-1 [Eucalyptus grandis]	26.182	45.316	10.6	2	10	2	0.95	0.926	0.963	1.076	1.061	1.076	1.132	5.62E-04
cds.Camellia_sinensis.comp21166_c0_seq1_m.51507	PREDICTED: heparanase-like protein 3 [Juglans regia]	15.463	1.3491	5.7	1	2	1	1.089	1.001	1.058	0.963	0.958	0.923	0.903	2.28E-02

cds.Camellia_sinensis.comp21169_c0_seq3_m.46243	PREDICTED: probable protein phosphatase 2C 55 isoform X1 [Vitis vinifera]	20.308	1.5837	5.7	1	1	1								
cds.Camellia_sinensis.comp21179_c0_seq1_m.19872	PREDICTED: probable aspartyl aminopeptidase isoform X1 [Nelumbo nucifera]	58.858	14.243	9.2	5	7	5	0.951	0.986	0.982	1.002	1.029	1.052	1.056	3.95E-02
cds.Camellia_sinensis.comp21197_c0_seq3_m.57054	plant/T7H20-70 protein [Medicago truncatula]	12.866	24.987	26.3	2	2	2	0.751	0.648	0.629	1.42	1.348	1.319	2.015	2.82E-04
cds.Camellia_sinensis.comp21209_c0_seq1_m.29021	DUF1262 family protein [Medicago truncatula]	44.286	28.627	14.4	5	8	5	0.862	0.92	0.882	1.129	1.16	1.109	1.276	4.60E-04
cds.Camellia_sinensis.comp21227_c0_seq1_m.42571	PREDICTED: D-tyrosyl-tRNA(Tyr) deacylase [Nelumbo nucifera]	17.985	1.3983	4.9	1	1	1								

cds.Camellia_sinensis.comp21264_c0_seq1_m.20635	PREDICTED: lysine histidine transporter 1-like [Lupinus angustifolius]	50.233	1.9656	2.7	1	1	1	0.82	0.924	1.067	1.133	1.016	1.089	1.152	1.51E-01
cds.Camellia_sinensis.comp21275_c0_seq1_m.12998	PREDICTED: chaperonin 60 subunit alpha 2, chloroplastic-like [Populus euphratica]	61.818	1.1618	6.2	3	5	2	1.088	0.963	0.975	0.98	0.892	1.095	0.981	7.75E-01
cds.Camellia_sinensis.comp21315_c0_seq2_m.24203	PREDICTED: fruit protein pKIWI502 [Vitis vinifera]	32.794	36.921	29.5	7	10	6	1.02	0.979	0.991	1.002	1.013	0.993	1.006	6.73E-01
cds.Camellia_sinensis.comp21350_c0_seq2_m.15219	PREDICTED: carotenoid 9,10(9',10')-cleavage dioxygenase 1 [Juglans regia]	61.464	148.92	41.7	19	42	19	0.969	0.99	0.977	1.023	1.024	1.018	1.044	2.70E-03

cds.Camellia_sinensis.comp21382_c0_seq1_m.50500	PREDICTED: uncharacterized N-acetyltransferase p20-like [Daucus carota subsp. sativus]	20.838	1.9119	9.2	1	1	1								
cds.Camellia_sinensis.comp21392_c0_seq2_m.8276	PREDICTED: probable galactinol--sucrose galactosyltransferase 2 [Juglans regia]	92.522	41.424	11.5	9	12	9	0.931	0.946	0.944	1.083	1.064	1.055	1.135	1.61E-04
cds.Camellia_sinensis.comp21420_c0_seq1_m.45997	PREDICTED: hevein-like preproprotein [Cicer arietinum]	21.021	31.624	6.5	1	3	1	1.092	1.114	1.158	0.921	0.878	0.908	0.805	6.22E-04
cds.Camellia_sinensis.comp21421_c0_seq2_m.33075	PREDICTED: bifunctional bis(5'-adenosyl)-triphosphatase/adenylylsulfatase FHIT [Juglans regia]	24.71	5.2553	12.3	3	4	3	1.048	1.046	1.036	0.956	0.949	0.96	0.915	5.78E-05

cds.Camellia_sinensis.comp21484_c0_seq1_m.37700	PREDICTED: peptidyl-prolyl cis-trans isomerase FKBP20-2, chloroplastic [Jatropha curcas]	33.366	32.118	21.6	6	8	6	0.955	0.962	1.048	1.036	0.985	0.996	1.018	6.19E-01
cds.Camellia_sinensis.comp21558_c0_seq1_m.41743	PREDICTED: aspartokinase 1, chloroplastic isoform X1 [Theobroma cacao]	24.629	1.5891	9.9	2	2	1								
cds.Camellia_sinensis.comp21576_c0_seq1_m.22086	PREDICTED: homogentisate 1,2-dioxygenase isoform X1 [Sesamum indicum]	50.845	6.2946	5	2	2	2	0.858	0.991	0.93	1.056	1.088	1.115	1.173	2.26E-02
cds.Camellia_sinensis.comp21606_c0_seq1_m.17315	PREDICTED: 4-alpha-glucanotransferase, chloroplastic/amyloplastic [Vitis vinifera]	65.701	7.3173	8.1	4	4	4	0.974	0.998	0.917	1.138	0.996	1.003	1.086	1.81E-01

cds.Camellia_sinensis.comp21721_c0_seq1_m.30161	PREDICTED: 2-C-methyl-D-erythritol 4-phosphate cytidylyltransferase, chloroplastic [Vitis vinifera]	35.778	49.58	38.6	13	24	13	1.015	1.009	0.986	1.004	0.99	0.982	0.989	3.60E-01
cds.Camellia_sinensis.comp21779_c0_seq1_m.32092	PREDICTED: heme-binding-like protein At3g10130, chloroplastic isoform X2 [Theobroma cacao]	33.993	75.585	48.8	15	48	15	0.897	0.896	0.918	1.127	1.079	1.111	1.224	1.80E-04
cds.Camellia_sinensis.comp21841_c0_seq1_m.43956	PREDICTED: calcium-transporting ATPase 1, chloroplastic [Sesamum indicum]	27.654	5.9469	7.7	2	2	1	0.932	0.932	1.067	0.977	1.06	1.052	1.054	3.59E-01

cds.Camellia_sinensis.comp21864_c0_seq1_m.30565	PREDICTED: uncharacterized oxidoreductase C663.09c [Populus euphratica]	32.941	7.4056	12	4	4	4	1.038	1.063	1	0.983	0.926	0.976	0.93	4.94E-02
cds.Camellia_sinensis.comp21888_c0_seq1_m.48398	PREDICTED: aldehyde dehydrogenase family 2 member B4, mitochondrial-like [Ziziphus jujuba]	22.893	90.865	40.7	7	19	5	0.994	0.996	1.018	1.031	0.966	1.017	1.002	9.41E-01
cds.Camellia_sinensis.comp21911_c0_seq1_m.38430	PREDICTED: stromal 70 kDa heat shock-related protein, chloroplastic-like [Nelumbo nucifera]	26.375	107.44	50.8	11	30	7	0.916	0.925	0.918	1.086	1.111	1.108	1.198	2.01E-05
cds.Camellia_sinensis.comp21927_c0_seq1_m.31407	PREDICTED: cytochrome P450 82C4-like [Prunus mume]	48.089	17.329	12.3	5	7	5	0.864	0.929	0.909	1.12	1.126	1.107	1.241	6.04E-04

cds.Camellia_sinensis.comp21931_c0_seq1_m.22916	Gut esterase (DUF1350) [Arabidopsis thaliana]	49.425	60.939	23.6	8	11	8	0.973	0.931	0.973	1.037	1.031	1.001	1.067	2.42E-02
cds.Camellia_sinensis.comp22064_c0_seq1_m.46500	PREDICTED: chorismate synthase 1, chloroplastic [Beta vulgaris subsp. vulgaris]	16.648	6.2342	11.3	1	2	1	1.064	1.095	1.036	1.021	0.872	0.902	0.875	5.48E-02
cds.Camellia_sinensis.comp22069_c0_seq1_m.21640	sodium/calcium exchanger family protein [Populus trichocarpa]	61.145	39.276	11.6	5	9	5	0.963	0.985	0.962	0.996	0.998	1.019	1.035	3.06E-02
cds.Camellia_sinensis.comp22081_c0_seq2_m.50310	PREDICTED: soluble inorganic pyrophosphatase 1 [Vitis vinifera]	14.788	13.996	22	3	9	1	1.044	1.038	1.109	0.916	1.004	0.882	0.878	4.05E-02
cds.Camellia_sinensis.comp22085_c0_seq1_m.53888	PREDICTED: IAA-amino acid hydrolase ILR1-like 4 [Cucumis sativus]	16.799	1.9779	8.8	1	1	1								

cds.Camellia_sinensis.comp22097_c0_seq1_m.39846	PREDICTED: small heat shock protein, chloroplastic [Theobroma cacao]	24.684	4.7051	12.4	2	3	2	1.319	1.149	1.122	0.825	0.757	0.761	0.653	1.80E-03
cds.Camellia_sinensis.comp22099_c0_seq1_m.36672	PREDICTED: cationic peroxidase 1-like [Pyrus x bretschneideri]	33.167	113.45	35.1	7	18	5	0.965	0.957	0.96	1.039	1.039	1.012	1.072	1.58E-03
cds.Camellia_sinensis.comp22099_c0_seq5_m.53430	PREDICTED: cationic peroxidase 1 [Vitis vinifera]	15.99	19.699	32.7	4	7	2	1.064	0.959	1.133	0.934	0.965	0.994	0.917	1.76E-01
cds.Camellia_sinensis.comp22154_c0_seq1_m.29275	PREDICTED: putative methyltransferase At1g22800 [Jatropha curcas]	26.418	2.2054	8.7	2	2	2	0.955	0.991	1.07	1.024	0.946	1.03	0.995	9.15E-01
cds.Camellia_sinensis.comp22155_c0_seq1_m.29099	PREDICTED: chromatin modification-related protein eaf-1 [Vitis vinifera]	50.077	1.2709	2.2	1	1	1								

cds.Camellia_sinensis.comp22240_c0_seq1_m.28792	PREDICTED: basic 7S globulin-like [Juglans regia]	46.374	117.73	26.5	9	24	9	0.871	0.931	0.903	1.125	1.112	1.116	1.24	3.84E-04
cds.Camellia_sinensis.comp22253_c0_seq1_m.45388	PREDICTED: major allergen Pru ar 1-like [Nelumbo nucifera]	17.754	87.068	73.1	11	35	11	0.848	0.819	0.827	1.191	1.207	1.175	1.433	5.00E-06
cds.Camellia_sinensis.comp22261_c0_seq1_m.29034	PREDICTED: pentatricopeptide repeat-containing protein At5g66520 [Vitis vinifera]	40.697	-2	2.2	1	1	1	0.99	1.025	1.084	1.047	0.949	0.914	0.939	2.57E-01
cds.Camellia_sinensis.comp22286_c0_seq1_m.20934	PREDICTED: zinc transporter 4, chloroplastic-like [Sesamum indicum]	32.578	10.504	11.2	2	2	2	1.044	1.252	0.935	0.987	0.965	0.807	0.854	2.20E-01

cds.Camellia_sinensis.comp22317_c0_seq4_m.51213	PREDICTED: GRIP and coiled-coil domain-containing protein 2 [Vitis vinifera]	12.78	-2	7.3	1	1	1	1.427	1.238	1.435	0.553	0.626	0.6	0.434	1.60E-04
cds.Camellia_sinensis.comp22344_c0_seq1_m.13415	PREDICTED: plant intracellular Ras-group-related LRR protein 4 [Ziziphus jujuba]	63.672	2.5108	2.6	1	1	1								
cds.Camellia_sinensis.comp22415_c0_seq1_m.35402	PREDICTED: DNA-directed RNA polymerases II and IV subunit 5A [Citrus sinensis]	24.382	6.2704	9.2	2	2	2	0.967	1.002	1.01	0.999	0.928	1.105	1.018	7.85E-01
cds.Camellia_sinensis.comp22451_c0_seq1_m.12748	PREDICTED: heparanase-like protein 1 [Vitis vinifera]	59.731	4.192	3.9	2	3	2	1.098	1.081	1.072	1.036	0.859	0.941	0.872	1.25E-01

cds.Camellia_sinensis.comp22492_c0_seq1_m.22766	PREDICTED: beta-glucosidase BoGH3B-like [Ziziphus jujuba]	54.998	11.594	8.2	3	3	3								
cds.Camellia_sinensis.comp22506_c0_seq1_m.10624	PREDICTED: probably inactive leucine-rich repeat receptor-like protein kinase At5g48380 [Theobrom	68.3	1.8432	3.1	2	2	2	0.931	0.917	0.917	1.139	1.16	0.98	1.186	8.89E-02
cds.Camellia_sinensis.comp22532_c0_seq1_m.36840	PREDICTED: probable carboxylesterase SOBER1-like [Vitis vinifera]	29.776	1.2974	6.2	2	2	2								
cds.Camellia_sinensis.comp22552_c0_seq1_m.40751	PREDICTED: plasma membrane-associated cation-binding protein 1 [Solanum tuberosum	21.783	34.546	28.5	6	10	6	1.025	0.947	0.952	1.037	1.042	1.037	1.066	1.29E-01

cds.Camellia_sinensis.comp22553_c0_seq2_m.39461	DUF3119 family protein [Medicago truncatula]	21.637	18.46	14.3	2	5	2	1.011	0.978	1.03	1.005	0.986	0.973	0.982	3.64E-01
cds.Camellia_sinensis.comp22574_c0_seq1_m.45157	PREDICTED: lactoylglutathione lyase isoform X1 [Theobroma cacao]	16.159	8.1878	16.1	2	2	2	0.991	0.963	0.932	1.045	1.003	1.083	1.085	4.57E-02
cds.Camellia_sinensis.comp22579_c0_seq1_m.25302	PREDICTED: scopoletin glucosyltransferase-like [Juglans regia]	49.205	20.501	4.2	2	3	2	0.927	0.931	0.971	1.088	1.081	1.035	1.133	4.48E-03
cds.Camellia_sinensis.comp22613_c0_seq1_m.28090	PREDICTED: protein FMP32, mitochondrial [Ricinus communis]	27.587	3.2806	8.6	2	2	2	0.956	1.02	1.033	0.984	0.986	1.033	0.998	9.56E-01
cds.Camellia_sinensis.comp22625_c0_seq2_m.34958	serotonin N-acetyltransferase 1, chloroplastic [Cajanus cajan]	22.051	7.1629	11.7	2	3	2	0.939	1.012	1.023	1.035	0.963	1.032	1.019	6.27E-01

cds.Camellia_sinensis.comp22684_c0_seq1_m.56844	PREDICTED: V-type proton ATPase subunit B 2 [Elaeis	12.837	1.2244	26.7	2	2	1								
cds.Camellia_sinensis.comp22694_c0_seq1_m.17208	PREDICTED: dihydrolipoyllysine-residue succinyltransferase component of 2-oxoglutarate dehydrogenase complex 2, mitochondrial-like [Nelumbo nucifera]	51.101	107.25	28.5	14	31	8	1	0.995	0.988	0.992	1.001	1.018	1.009	3.28E-01
cds.Camellia_sinensis.comp22699_c0_seq1_m.2313	PREDICTED: intron-binding protein aquarius [Juglans regia]	160.36	16.429	4.2	5	5	5	0.941	1.032	1.079	0.964	0.983	1.011	0.969	5.22E-01
cds.Camellia_sinensis.comp22782_c0_seq1_m.41856	cAMP-regulated phosphoprotein-like protein [Medicago truncatula]	17.387	1.3302	6.5	1	1	1	0.936	1.099	1.022	1.05	0.937	0.97	0.967	6.04E-01

cds.Camellia_sinensis.comp22795_c1_seq1_m.34061	PREDICTED: mitochondrial uncoupling protein 1 [Ziziphus jujuba]	28.825	42.105	19.8	5	7	5	1.036	1.084	1.031	0.934	0.991	0.941	0.91	1.80E-02
cds.Camellia_sinensis.comp22798_c0_seq1_m.33202	PREDICTED: RPM1-interacting protein 4 [Vitis vinifera]	28.326	17.158	11.7	2	2	2								
cds.Camellia_sinensis.comp22831_c0_seq1_m.33671	PREDICTED: chloroplastic lipocalin [Vitis vinifera]	42.169	118.47	26.1	9	22	9	1.006	0.923	0.959	1	1.059	1.005	1.061	1.27E-01
cds.Camellia_sinensis.comp22839_c0_seq1_m.26890	PREDICTED: peptide chain release factor APG3, chloroplastic [Vitis vinifera]	47.322	60.411	18.7	8	11	8	1.012	1.03	1.025	0.948	0.965	0.981	0.944	6.54E-03
cds.Camellia_sinensis.comp22895_c0_seq1_m.31295	PREDICTED: aspartic proteinase PCS1 [Sesamum indicum]	44.538	1.3414	2.5	1	1	1	0.941	1.138	1	0.996	0.964	0.969	0.951	4.57E-01

cds.Camellia_sinensis.comp22898_c0_seq1_m.56873	Phosphoenolpyruvate carboxylase 2 [Morus notabilis]	13.764	4.6863	25.8	3	6	1	0.886	0.945	0.955	1.136	1.088	1.031	1.168	1.32E-02
cds.Camellia_sinensis.comp22944_c0_seq2_m.24036	PREDICTED: protein IQ-DOMAIN 32 isoform X2 [Vitis vinifera]	44.643	4.092	5.8	2	2	2	0.963	1.031	1.124	0.955	0.947	0.984	0.926	1.78E-01
cds.Camellia_sinensis.comp22990_c0_seq1_m.25216	PREDICTED: putative transferase At1g60990, chloroplastic [Juglans regia]	45.821	1.5124	2.6	1	1	1								
cds.Camellia_sinensis.comp23016_c0_seq1_m.19417	PREDICTED: transketolase, chloroplastic [Nelumbo nucifera]	54.239	62.708	13.6	7	23	4	1.021	0.967	1.005	0.995	1.014	1.034	1.017	4.42E-01
cds.Camellia_sinensis.comp23018_c0_seq1_m.18026	PREDICTED: dnaJ homolog 1, mitochondrial-like [Sesamum	55.904	65.371	28.8	16	27	9	1.149	1.133	1.113	0.831	0.85	0.894	0.758	2.98E-04

cds.Camellia_sinensis.comp23042_c0_seq1_m.25951	PREDICTED: formamidase-like isoform X1 [Ziziphus jujuba]	51.648	73.137	30	12	23	12	0.96	0.947	0.933	1.032	1.073	1.052	1.112	1.62E-03
cds.Camellia_sinensis.comp23049_c0_seq1_m.34340	PREDICTED: omega-amidase, chloroplastic [Theobroma cacao]	29.561	1.4661	3.4	1	1	1								
cds.Camellia_sinensis.comp23119_c0_seq1_m.42782	PREDICTED: disease resistance RPP13-like protein 4 [Capsicum annuum]	31.325	20.403	9.1	2	2	2	0.853	1.096	0.878	1.067	1.126	1.018	1.136	1.90E-01
cds.Camellia_sinensis.comp23125_c0_seq1_m.49092	Ras-related protein Rab11D [Morus notabilis]	14.03	18.378	26.8	3	4	3	0.913	0.915	1	1.059	1.008	1.136	1.133	5.36E-02
cds.Camellia_sinensis.comp23136_c0_seq1_m.40278	PREDICTED: probable glutathione S-transferase [Vitis vinifera]	25.414	15.839	29.7	8	9	7	0.978	0.947	0.937	1.032	1.108	1.045	1.113	1.37E-02

cds.Camellia_sinensis.comp23155_c0_seq2_m.45028	PREDICTED: peroxidase 17-like [Nicotiana tabacum]	24.545	2.1519	6.3	1	1	1								
cds.Camellia_sinensis.comp23155_c0_seq3_m.55573	PREDICTED: peroxidase 17-like [Prunus mume]	14.426	1.4836	6.9	1	1	1	0.965	0.996	0.965	1.003	0.989	1.096	1.055	1.93E-01
cds.Camellia_sinensis.comp23164_c0_seq1_m.40447	PREDICTED: sorting nexin 2B [Daucus carota subsp. sativus]	23.891	5.0371	12.4	2	2	1								
cds.Camellia_sinensis.comp23171_c0_seq1_m.26030	PREDICTED: heat shock 70 kDa protein 8 [Ipomoea nil]	49.514	4.7757	3.7	2	2	2	0.915	0.953	0.934	1.076	1.083	1.074	1.154	2.82E-04
cds.Camellia_sinensis.comp23173_c0_seq1_m.24596	PREDICTED: TOM1-like protein 2 isoform X2 [Populus euphratica]	50.344	5.0933	5.8	3	4	3	0.972	0.987	0.974	1.075	1.081	1.032	1.087	5.68E-03

cds.Camellia_sinensis.comp23185_c0_seq1_m.52057	PREDICTED: protein SIEVE ELEMENT OCCLUSION B [Vitis vinifera]	19.138	17.087	23.2	4	6	4	0.937	0.917	0.911	1.061	1.101	1.106	1.182	4.39E-04
cds.Camellia_sinensis.comp23200_c0_seq3_m.35606	PREDICTED: red chlorophyll catabolite reductase [Vitis vinifera]	36.553	34.865	23.9	7	11	7	1.024	0.995	1.037	1.02	0.996	0.994	0.985	3.65E-01
cds.Camellia_sinensis.comp23202_c0_seq1_m.39021	glycoprotein family protein [Medicago truncatula]	27.823	2.7613	4.1	1	1	1	0.966	1.203	0.843	0.991	1.01	0.993	0.994	9.67E-01
cds.Camellia_sinensis.comp23207_c0_seq2_m.46038	DNA-directed RNA polymerase subunit beta [Arabidopsis thaliana]	26.32	5.4891	9.3	2	2	2								
cds.Camellia_sinensis.comp23208_c0_seq1_m.32620	PREDICTED: cannabidiolic acid synthase-like [Sesamum indicum]	40.208	1.5719	2.6	1	1	1								

cds.Camellia_sinensis.comp23208_c0_seq2_m.46046	PREDICTED: berberine bridge enzyme-like 21 [Vitis vinifera]	21.744	13.087	29.8	6	8	6	0.957	0.953	0.983	1.073	1.033	1.055	1.093	3.78E-03
cds.Camellia_sinensis.comp23213_c0_seq1_m.25257	PREDICTED: aspartate aminotransferase, mitochondrial isoform X1 [Sesamum	48.715	23.843	23	10	13	10	1.056	1.035	1.002	0.967	0.975	1.004	0.952	6.34E-02
cds.Camellia_sinensis.comp23229_c0_seq2_m.32276	PREDICTED: ubiquinol oxidase 4, chloroplastic/chromoplastic [Vitis vinifera]	40.851	61.033	26.4	9	21	9	1.031	1.004	1.044	0.952	0.969	0.978	0.942	1.26E-02
cds.Camellia_sinensis.comp23234_c0_seq1_m.28763	virus resistant/susceptible lipocalin [Solanum lycopersicum]	34.159	5.3054	12.1	3	4	3	0.976	0.975	1.046	0.978	0.962	1.074	1.006	9.10E-01

cds.Camellia_sinensis.comp23253_c0_seq1_m.58877	PREDICTED: haloacid dehalogenase-like hydrolase domain-containing protein Sgpp [Populus euphratica]	11.591	1.3079	7.7	1	1	1	1.167	1.211	1.113	0.835	0.836	0.794	0.706	3.03E-04
cds.Camellia_sinensis.comp23310_c0_seq2_m.42008	PREDICTED: glycine-rich RNA-binding protein 4, mitochondrial [Vitis vinifera]	15.692	27.621	41.4	5	5	5	1.012	0.963	1.012	1.074	0.952	1.043	1.027	5.49E-01
cds.Camellia_sinensis.comp23332_c0_seq1_m.48713	glutaredoxin 2 [Medicago truncatula]	18.18	10.309	18.4	3	3	3	0.969	0.931	1.005	1.051	1.039	1.027	1.073	3.72E-02
cds.Camellia_sinensis.comp23350_c0_seq1_m.44664	PREDICTED: ABC transporter G family member 15-like isoform X1 [Nicotiana attenuata]	18.589	2.1519	5.6	1	1	1								

cds.Camellia_sinensis.comp23354_c0_seq2_m.40883	PREDICTED: probable ubiquitin-conjugating enzyme E2 18 [Jatropha curcas]	18.272	2.774	9.3	2	4	2	0.976	1.011	0.974	0.996	1.061	0.955	1.017	6.47E-01
cds.Camellia_sinensis.comp23356_c0_seq1_m.39580	PREDICTED: NADP-dependent malic enzyme-like isoform X1 [Nicotiana tabacum]	31.6	3.3679	14.6	3	7	2								
cds.Camellia_sinensis.comp23375_c0_seq1_m.15225	bZIP transcription factor TRAB1-like [Nicotiana tabacum]	47.506	2.0024	7.5	2	2	2	1.008	1.122	1.073	0.956	0.866	0.967	0.871	4.15E-02
cds.Camellia_sinensis.comp23383_c0_seq1_m.31016	PREDICTED: peroxidase 31 [Vitis vinifera]	36.737	2.4008	3.6	1	1	1	0.976	1.283	1.093	0.91	0.905	0.82	0.786	5.24E-02

cds.Camellia_sinensis.comp23407_c0_seq1_m.35915	PREDICTED: selT-like protein isoform X4 [Nicotiana tomentosiformis]	25.471	1.1979	4.8	1	1	1								
cds.Camellia_sinensis.comp23411_c0_seq1_m.20884	PREDICTED: UDP-arabinose 4-epimerase 1 [Prunus mume]	45.894	1.4122	2.9	1	1	1								
cds.Camellia_sinensis.comp23442_c0_seq2_m.23113	PREDICTED: protein TRIGALACTOSYLDIACYLGLYCEROL 1, chloroplastic isoform X1 [Juglans regia]	38.318	2.6413	6.2	2	3	2	0.929	0.952	1.046	1.086	1.004	1.01	1.059	2.58E-01
cds.Camellia_sinensis.comp23458_c0_seq1_m.12251	PREDICTED: probable methyltransferase PMT14 [Theobroma cacao]	69.76	13.154	8.9	5	5	5	1.031	1.151	1.183	0.903	0.731	0.976	0.776	5.39E-02

cds.Camellia_sinensis.comp23469_c0_seq2_m.34279	3-oxo-5-alpha-steroid 4-dehydrogenase [Medicago truncatula]	25.884	1.4806	8.4	2	3	2	0.932	1.01	0.986	1.032	1.061	1.002	1.057	1.27E-01
cds.Camellia_sinensis.comp23481_c0_seq1_m.49036	PREDICTED: plant intracellular Ras-group-related LRR protein 1-like [Nicotiana attenuata]	20.063	2.1263	8.5	1	1	1								
cds.Camellia_sinensis.comp23488_c0_seq1_m.40789	PREDICTED: glutaredoxin-like [Juglans regia]	12.711	16.597	31.7	3	5	3	0.934	0.973	0.949	1.001	1.089	1.083	1.111	2.44E-02
cds.Camellia_sinensis.comp23492_c0_seq1_m.39466	glutathione S-transferase DHAR2-like [Sesamum indicum]	26.223	82.852	30.2	5	14	4	0.97	0.994	1.044	1.011	0.975	1.001	0.993	7.97E-01
cds.Camellia_sinensis.comp23496_c0_seq1_m.44487		20.864	10.646	6.3	1	2	1	0.947	0.979	1.028	0.937	1.025	1.096	1.035	5.54E-01

cds.Camellia_sinensis.comp23504_c0_seq1_m.19034	PREDICTED: mitogen-activated protein kinase kinase 2 [Ziziphus jujuba]	40.538	54.412	13.5	4	7	4	0.967	0.999	0.998	1.046	1.008	0.978	1.023	3.69E-01
cds.Camellia_sinensis.comp23511_c0_seq1_m.35001	universal stress family protein [Populus trichocarpa]	20.237	5.7714	16.8	3	4	3	0.96	1.153	1.009	1.072	0.912	0.903	0.925	3.72E-01
cds.Camellia_sinensis.comp23511_c0_seq2_m.39360	PREDICTED: universal stress protein A-like protein [Nelumbo nucifera]	21.602	12.205	25	5	8	5	0.993	1.016	1.032	0.982	0.947	1.055	0.981	5.88E-01
cds.Camellia_sinensis.comp23527_c0_seq1_m.22234	PREDICTED: alpha-L-fucosidase 1 [Juglans regia]	60.611	3.8695	1.9	1	2	1	0.944	0.927	1.018	1.078	0.978	1.08	1.085	1.33E-01

cds.Camellia_sinensis.comp23540_c0_seq1_m.17687	PREDICTED: protein TRIGALACTOSYLDIACYLGLYCEROL 2, chloroplastic isoform X2 [Vitis vinifera]	30.947	47.709	16.2	5	6	5	0.961	0.994	1.008	1.019	1.021	1.046	1.042	6.85E-02
cds.Camellia_sinensis.comp23540_c0_seq1_m.17688	PREDICTED: protein TRIGALACTOSYLDIACYLGLYCEROL 2, chloroplastic [Eucalyptus grandis]	11.466	32.127	42.2	3	8	3	0.971	0.993	0.947	1.071	0.956	1.099	1.074	1.96E-01
cds.Camellia_sinensis.comp23543_c0_seq1_m.33026	PREDICTED: cinnamoyl-CoA reductase 1 [Solanum tuberosum	36.641	29.051	21.3	7	16	7	1.264	1.283	1.25	0.716	0.719	0.699	0.562	4.89E-07
cds.Camellia_sinensis.comp23549_c0_seq1_m.33670	PREDICTED: proactivator polypeptide-like 1 [Theobroma cacao]	27.651	46.085	18.1	4	9	4	0.995	0.965	0.977	1.052	1.038	1.034	1.064	3.90E-03

cds.Camellia_sinensis.comp23551_c0_seq1_m.11022	PREDICTED: transcription initiation factor TFIID subunit 15b [Sesamum indicum]	41.732	30.181	13	5	8	5	1.125	1.148	1.05	0.864	0.864	0.914	0.795	2.24E-03
cds.Camellia_sinensis.comp23552_c0_seq1_m.31138	PREDICTED: ubiquitin-like domain-containing CTD phosphatase [Vitis vinifera]	37.965	8.6382	6.6	2	2	2	1.068	1.062	1.041	0.916	0.962	0.938	0.888	1.82E-03
cds.Camellia_sinensis.comp23582_c0_seq1_m.19162	PREDICTED: acetolactate synthase small subunit 1, chloroplastic-like [Solanum tuberosum	54.117	56.209	26.6	10	20	10	0.955	0.994	0.995	1.045	1.027	1.037	1.056	1.92E-02
cds.Camellia_sinensis.comp23592_c0_seq1_m.24273	endoribonuclease E-like protein [Medicago truncatula]	48.129	1.3727	1.9	1	1	1	1.046	1.02	0.918	1.045	0.978	0.999	1.013	7.71E-01

cds.Camellia_sinensis.comp23594_c0_seq1_m.30848	PREDICTED: translation initiation factor IF-2, chloroplastic isoform X1 [Sesamum indicum]	28.92	6.8192	20.5	5	6	5	1.15	1.22	1.173	0.827	0.811	0.791	0.686	6.16E-05
cds.Camellia_sinensis.comp23603_c1_seq1_m.31837	PREDICTED: secretory carrier-associated membrane protein 2-like [Nicotiana tabacum]	34.192	63.879	26.5	6	7	6	1.014	0.964	1.017	1.033	0.951	0.98	0.99	7.38E-01
cds.Camellia_sinensis.comp23604_c0_seq1_m.10644	muniscin carboxy-terminal mu-like domain protein [Medicago truncatula]	32.731	4.8523	10	3	3	3	1.079	0.978	0.962	0.988	0.98	1.002	0.984	6.99E-01
cds.Camellia_sinensis.comp23607_c0_seq1_m.42110	NtPRp27-like protein precursor [Solanum tuberosum	27.16	28.377	23.3	5	15	4	0.942	0.916	0.935	1.077	1.09	1.059	1.155	2.60E-04

cds.Camellia_sinensis.comp23618_c0_seq1_m.33504	PREDICTED: thioredoxin-like protein AAED1, chloroplastic [Pyrus x bretschneideri]	29.437	56.459	36.4	10	14	10	1.035	1.015	1.044	0.947	0.988	0.952	0.933	1.16E-02
cds.Camellia_sinensis.comp23629_c1_seq1_m.46035	PREDICTED: nuclear transcription factor Y subunit B-3 [Vitis vinifera]	14.512	39.23	11.3	1	2	1	0.986	1.104	1.046	0.962	0.899	1.001	0.913	1.14E-01
cds.Camellia_sinensis.comp23638_c0_seq2_m.33624	PREDICTED: iron-sulfur cluster co-chaperone protein HscB, mitochondrial isoform X1 [Vitis	31.34	3.3966	4.1	1	1	1	1.041	1.033	0.906	0.879	0.966	1.167	1.011	9.55E-01
cds.Camellia_sinensis.comp23640_c0_seq1_m.22313	PREDICTED: formate dehydrogenase, mitochondrial [Theobroma cacao]	42.427	133.56	38.3	14	67	14	0.988	0.975	0.983	1.021	1.021	1.019	1.039	6.15E-04

cds.Camellia_sinensis.comp23644_c0_seq1_m.2851	PREDICTED: DNA-directed RNA polymerase II subunit RPB2 [Ziziphus	134.92	9.3801	3.1	3	4	3	1.122	1.136	1.161	0.891	0.862	0.848	0.761	1.01E-04
cds.Camellia_sinensis.comp23661_c0_seq1_m.36437	PREDICTED: vesicle-associated membrane protein 711 [Nicotiana tabacum]	27.066	3.5117	16.2	4	6	4	1.019	1.016	1.016	0.998	0.99	0.976	0.971	4.81E-02
cds.Camellia_sinensis.comp23677_c0_seq1_m.29418	PREDICTED: protein STRICTOSIDINE SYNTHASE-LIKE 4-like [Juglans regia]	41.47	23.551	18.5	7	11	7	1.009	0.995	0.97	0.951	1.029	1.018	1.008	7.95E-01
cds.Camellia_sinensis.comp23686_c0_seq1_m.36715	PREDICTED: probable thylakoid lumen protein sll1769 isoform X2 [Solanum tuberosum	24.783	93.386	27.3	6	18	6	0.905	0.874	0.887	1.117	1.108	1.127	1.257	3.71E-05

cds.Camellia_sinensis.comp23687_c0_seq2_m.41874	PREDICTED: universal stress protein PHOS32 [Eucalyptus grandis]	20.396	10.02	19.7	3	5	3	0.855	1.029	0.945	1.152	1.06	1.046	1.152	8.04E-02
cds.Camellia_sinensis.comp23689_c0_seq1_m.18354	PREDICTED: xylose isomerase [Jatropha curcas]	52.848	165.26	49.2	20	56	20	0.875	0.865	0.882	1.149	1.167	1.133	1.315	1.58E-05
cds.Camellia_sinensis.comp23691_c0_seq1_m.18836	PREDICTED: uridine kinase-like protein 1, chloroplastic [Nicotiana tabacum]	54.733	13.916	9.4	4	4	4	1.007	1.118	1.016	0.929	0.984	0.985	0.923	1.08E-01
cds.Camellia_sinensis.comp23696_c0_seq1_m.41908	PREDICTED: 28 kDa ribonucleoprotein, chloroplastic-like [Nelumbo nucifera]	18.588	84.4	62.4	10	31	8	1.017	1.007	1.037	0.976	0.973	0.971	0.954	5.76E-03
cds.Camellia_sinensis.comp23709_c0_seq1_m.40618	embryo-specific protein ATS3B-like [Cajanus cajan]	21.885	79.669	19.4	3	12	3	0.955	0.917	0.828	1.107	1.112	1.106	1.231	3.86E-02

cds.Camellia_sinensis.comp23718_c0_seq2_m.39301	PREDICTED: serine/arginine-rich splicing factor RSZ21-like isoform X1 [Arachis ipaensis]	20.113	2.1786	7.3	1	1	1	1.057	1.037	0.991	0.944	0.997	0.97	0.944	7.96E-02
cds.Camellia_sinensis.comp23719_c0_seq1_m.9918	PREDICTED: THO complex subunit 4A [Arachis duranensis]	24.963	31.904	30.7	7	11	5	1.093	1.122	1.048	0.887	0.914	0.948	0.842	3.44E-03
cds.Camellia_sinensis.comp23725_c0_seq1_m.39683	transferring glycosyl group transferase [Arabidopsis thaliana]	25.464	7.86	8.6	2	4	2	0.959	0.974	0.965	1.005	1.111	1.008	1.078	1.56E-01
cds.Camellia_sinensis.comp23726_c0_seq2_m.17993	PREDICTED: polyadenylate-binding protein RBP47 [Vitis	47.033	6.6267	6.3	3	3	3	0.918	1.006	1.03	1.026	1.054	0.99	1.039	3.75E-01

cds.Camellia_sinensis.comp23731_c0_seq1_m.24376	PREDICTED: ATP phosphoribosyltransferase 2, chloroplastic-like [Nicotiana tabacum]	44.373	48.254	21.1	8	11	8	0.975	1.036	0.99	1.019	1.001	0.988	1.002	9.05E-01
cds.Camellia_sinensis.comp23736_c0_seq2_m.11787	PREDICTED: translation factor GUF1 homolog, chloroplastic [Nelumbo nucifera]	75.161	6.6564	5.9	4	4	4	1.066	1.089	1.062	0.899	0.939	0.948	0.866	1.36E-03
cds.Camellia_sinensis.comp23737_c0_seq1_m.46339		18.499	49.485	17.3	2	4	2	1.037	0.987	0.985	1.014	0.954	1.027	0.995	8.71E-01
cds.Camellia_sinensis.comp23742_c0_seq1_m.51948	PREDICTED: probable glutathione S-transferase [Ricinus communis]	12.013	1.1714	7.5	1	1	1								
cds.Camellia_sinensis.comp23767_c0_seq6_m.6889	PREDICTED: pre-mRNA-processing factor 39 isoform X1 [Vitis vinifera]	97.418	49.581	11.6	11	14	11	0.98	0.997	0.988	1.011	1.012	1.011	1.023	4.37E-02

cds.Camellia_sinensis.comp23775_c0_seq1_m.27773	PREDICTED: probable 3-hydroxyisobutyrate dehydrogenase-like 2, mitochondrial [Fragaria vesca subsp. vesca]	31.692	33.947	34.1	8	13	8	1.014	1.031	1.051	1.001	0.921	0.984	0.939	8.04E-02
cds.Camellia_sinensis.comp23787_c0_seq1_m.25725	PREDICTED: sporulation protein RMD1-like [Sesamum indicum]	29.51	3.6775	7.1	2	2	2	0.892	0.999	0.988	1.014	1.062	1.073	1.094	8.63E-02
cds.Camellia_sinensis.comp23789_c0_seq1_m.35175	fiber protein Fb34 [Medicago truncatula]	19.236	1.5592	5.1	1	1	1	0.9	0.889	0.891	1.198	1.074	1.097	1.257	2.12E-02

cds.Camellia_sinensis.comp23812_c0_seq3_m.16318	PREDICTED: probable pre-mRNA-splicing factor ATP-dependent RNA helicase [Vigna radiata var. radiata]	60.597	27.547	13.1	7	11	7	0.972	0.994	1.049	0.997	1.004	0.996	0.994	8.32E-01
cds.Camellia_sinensis.comp23814_c0_seq1_m.30203	PREDICTED: reticulon-like protein B8 [Vitis vinifera]	27.492	28.028	23.7	6	10	6	1.047	0.999	1	0.983	0.978	0.992	0.969	1.27E-01
cds.Camellia_sinensis.comp23815_c0_seq2_m.36254	PREDICTED: ras-related protein RABC1 [Vitis vinifera]	23.477	32.347	24.3	4	9	3	0.988	0.969	1.047	1.095	1.067	1.009	1.056	1.82E-01
cds.Camellia_sinensis.comp23816_c0_seq1_m.50338	PREDICTED: photosynthetic NDH subunit of subcomplex B 3, chloroplastic [Jatropha curcas]	19.385	4.4973	5.6	1	2	1	1.155	1.115	1.098	0.94	0.836	0.826	0.773	4.08E-03

cds.Camellia_sinensis.comp23817_c0_seq1_m.16439	PREDICTED: ATP-dependent RNA helicase DBP2-like [Nelumbo nucifera]	60.974	3.7866	8	5	9	3	0.933	0.975	1.017	1.066	1.01	1.024	1.06	1.20E-01
cds.Camellia_sinensis.comp23822_c0_seq1_m.12574	PREDICTED: phenylalanine--tRNA ligase beta subunit, cytoplasmic-like [Nelumbo nucifera]	66.507	25.283	21.1	11	15	11	0.944	1	0.948	1.076	1.071	1.042	1.103	9.66E-03
cds.Camellia_sinensis.comp23829_c0_seq1_m.33036	PREDICTED: annexin D4 [Vitis vinifera]	37.33	25.58	25	9	14	9	1.078	1.078	1.043	0.901	0.926	0.929	0.862	5.25E-04

cds.Camellia_sinensis.comp23849_c0_seq15_m.12318	PREDICTED: bifunctional 3-dehydroquinate dehydratase/shikimate dehydrogenase, chloroplastic-like isoform X1 [Daucus carota subsp. sativus]	57.516	88.486	21.9	11	17	10	1.032	1.081	1.068	0.91	0.975	0.911	0.879	8.38E-03
cds.Camellia_sinensis.comp23854_c0_seq1_m.38600	ribosomal RNA small subunit methyltransferase G [Arabidopsis sis	30.251	74.71	39.6	10	19	10	0.958	0.967	0.974	1.074	1.056	1.027	1.089	3.60E-03
cds.Camellia_sinensis.comp23861_c0_seq11_c0_seq1_m.37897	probable calcium-binding protein CML21 [Cajanus cajan]	26.354	16.126	19.2	4	4	4	1.121	1.01	1.03	1.01	0.945	0.921	0.91	8.92E-02

cds.Camellia_sinensis.comp23862_c0_seq2_m.17829	PREDICTED: fe-S cluster assembly factor HCF101, chloroplastic [Vitis vinifera]	42.728	68.602	19.4	6	9	6	1.04	1.004	1.055	0.97	0.937	0.941	0.919	9.98E-03
cds.Camellia_sinensis.comp23866_c0_seq1_m.32836	PREDICTED: probable prolyl 4-hydroxylase 4 [Ricinus communis]	35.937	3.9901	6.8	2	2	2	1.071	1.074	0.945	0.945	0.976	0.98	0.939	2.32E-01
cds.Camellia_sinensis.comp23873_c0_seq2_m.6052	PREDICTED: DNA gyrase subunit A, chloroplastic/mitochondrial isoform X1 [Vitis vinifera]	102.56	35.639	13.3	12	16	12	0.975	0.984	0.981	1.038	1.019	1.02	1.047	2.30E-03
cds.Camellia_sinensis.comp23877_c0_seq1_m.18718	PREDICTED: galactokinase-like [Ziziphus jujuba]	54.462	184.09	45.3	18	36	15	0.981	0.967	0.964	1.026	0.996	1.01	1.041	1.63E-02

cds.Camellia_sinensis.comp23881_c0_seq1_m.36848	PREDICTED: photosynthetic NDH subunit of subcomplex B 4, chloroplastic-like [Vitis vinifera]	21.646	11.759	17.2	4	6	4	1.006	1.008	0.989	0.974	0.993	1.019	0.994	7.07E-01
cds.Camellia_sinensis.comp23884_c0_seq1_m.19157	PREDICTED: divinyl chlorophyllide a 8-vinyl-reductase, chloroplastic [Jatropha curcas]	46.245	63.188	21	8	11	8	1.003	1.03	1.045	0.976	1.031	0.953	0.962	2.06E-01
cds.Camellia_sinensis.comp23885_c0_seq1_m.9327	PREDICTED: glycine-rich domain-containing protein 1-like [Juglans regia]	87.586	3.4479	1.4	1	1	1	0.973	1.077	1.054	1.064	0.953	0.891	0.937	3.29E-01

cds.Camellia_sinensis.comp23891_c0_seq2_m.26046	PREDICTED: aminoacyl tRNA synthase complex-interacting multifunctional protein 1 isoform X2 [Vitis vinifera]	41.356	14.133	13.7	6	7	6	1.019	1.007	1.01	0.989	1.002	0.96	0.972	9.59E-02
cds.Camellia_sinensis.comp23905_c0_seq1_m.37019	PREDICTED: haloacid dehalogenase-like hydrolase domain-containing protein At3g48420 [Nicotiana	27.422	72.802	40.3	10	23	10	1.027	1.02	1.029	0.969	1.002	0.973	0.957	1.56E-02
cds.Camellia_sinensis.comp23914_c0_seq1_m.17817	PREDICTED: aminopeptidase M1-like [Juglans regia]	56.968	96.165	35.9	21	39	21	0.95	0.944	0.95	1.076	1.054	1.05	1.118	1.42E-04

cds.Camellia_sinensis.comp23927_c0_seq1_m.51036	PREDICTED: thioredoxin M-type, chloroplastic-like [Fragaria vesca subsp. vesca]	13.205	3.8723	15.3	2	8	1	1.042	1.05	0.985	1.032	0.961	0.954	0.958	2.49E-01
cds.Camellia_sinensis.comp23928_c0_seq1_m.4633	PREDICTED: coatomer subunit beta-1 [Theobroma cacao]	105.71	98.623	23.8	19	29	19	1.027	1.017	0.99	0.97	1.026	1.006	0.989	6.15E-01
cds.Camellia_sinensis.comp23930_c0_seq1_m.9730	PREDICTED: long chain acyl-CoA synthetase 8 [Sesamum indicum]	80.241	84.322	21.6	13	19	13	1.008	0.988	1.013	1.005	0.982	0.993	0.99	3.95E-01
cds.Camellia_sinensis.comp23931_c0_seq1_m.30689	PREDICTED: thylakoid membrane protein ssl2009-like [Nicotiana tabacum]	24.727	82.523	13.3	3	5	3	1.079	1.085	1.047	0.937	0.969	0.902	0.874	4.60E-03

cds.Camellia_sinensis.comp23961_c0_seq1_m.41248	nuclear transport factor 2 (NTF2) family protein [Medicago truncatula]	23.204	19.266	45.8	10	20	10	1.048	1.024	1.011	0.943	0.986	0.999	0.95	6.40E-02
cds.Camellia_sinensis.comp23966_c0_seq1_m.36614	PREDICTED: 25.3 kDa vesicle transport protein [Ziziphus jujuba]	25.392	49.328	28.9	7	11	7	0.899	0.908	0.991	1.056	1.062	1.054	1.134	5.53E-02
cds.Camellia_sinensis.comp23975_c0_seq3_m.15198	PREDICTED: YTH domain-containing family protein 1-like isoform X2 [Pyrus x bretschneideri]	59.744	4.3401	4.1	2	2	2	0.966	1.01	0.929	1.007	1.085	1.021	1.072	1.06E-01
cds.Camellia_sinensis.comp23980_c0_seq1_m.36708	PREDICTED: 28 kDa ribonucleoprotein, chloroplastic [Vitis vinifera]	21.557	15.883	18.5	4	5	4	1.014	1.063	1.028	0.938	1.029	0.983	0.95	1.62E-01

cds.Camellia_sinensis.comp23986_c0_seq2_m.42970	PREDICTED: prefoldin subunit 1 isoform X1 [Vitis vinifera]	14.505	16.108	36.5	5	6	5	0.886	0.897	0.946	1.065	1.067	1.142	1.2	3.98E-03
cds.Camellia_sinensis.comp23989_c0_seq1_m.34980	calcium-binding EF hand family protein [Populus trichocarpa]	36.215	19.815	16.2	5	6	5	0.997	1.035	1.036	0.992	1.003	0.958	0.963	1.11E-01
cds.Camellia_sinensis.comp23991_c0_seq1_m.8691	PREDICTED: long chain acyl-CoA synthetase 9, chloroplastic [Vitis vinifera]	76.838	53.751	17.2	9	17	9	1.036	0.991	1.018	0.981	0.985	0.977	0.967	6.11E-02
cds.Camellia_sinensis.comp23998_c0_seq1_m.18906	PREDICTED: DEAD-box ATP-dependent RNA helicase 20 [Ziziphus jujuba]	55.594	71.087	36.3	17	23	15	0.989	1.015	0.985	0.981	1.029	0.999	1.007	7.16E-01

cds.Camellia_sinensis.comp24002_c0_seq1_m.29975	PREDICTED: probable hydroxyacylglutathione hydrolase 2, chloroplastic [Capsicum annuum]	39.225	29.218	20.1	6	9	6	1.05	1.081	1.073	0.948	0.915	0.903	0.863	9.64E-04
cds.Camellia_sinensis.comp24003_c0_seq1_m.33142	PREDICTED: phospholipid hydroperoxide glutathione peroxidase 1, chloroplastic-like [Sesamum indicum]	31.524	76.61	34	8	26	8	1.051	0.992	1.015	0.955	0.981	0.978	0.953	6.32E-02
cds.Camellia_sinensis.comp24028_c0_seq1_m.36099	PREDICTED: UPF0235 protein At5g63440 [Ricinus communis]	25.76	1.8266	6.9	2	2	2	1	1.346	0.933	0.894	0.879	0.928	0.824	2.52E-01
cds.Camellia_sinensis.comp24036_c0_seq2_m.16475	PREDICTED: probable beta-D-xylosidase 5 [Theobroma cacao]	73.884	57.593	13.3	7	9	7	0.879	0.921	0.92	1.102	1.062	1.139	1.214	1.58E-03

cds.Camellia_sinensis.comp24048_c0_seq3_m.28524	PREDICTED: histidine biosynthesis bifunctional protein hisIE, chloroplastic [Vitis vinifera]	32.742	16.105	21.7	6	7	6	1.005	1.05	1.033	0.992	1.005	0.927	0.947	1.22E-01
cds.Camellia_sinensis.comp24049_c0_seq1_m.38295	peptide methionine sulfoxide reductase [Solanum lycopersicum]	24.571	8.2113	16.5	4	5	4	1.037	1.019	0.992	1.004	0.931	1.014	0.968	3.24E-01
cds.Camellia_sinensis.comp24050_c0_seq1_m.19288	PREDICTED: protein RETICULATA-RELATED 1, chloroplastic-like [Solanum tuberosum	60.605	5.2906	5	3	3	3	1.11	1.168	1.096	0.849	0.895	0.85	0.769	5.43E-04
cds.Camellia_sinensis.comp24053_c0_seq1_m.10074	PREDICTED: probable Xaa-Pro aminopeptidase P isoform X1 [Juglans regia]	57.853	27.96	17.1	8	11	8	0.978	1.012	1.015	1.061	0.999	0.959	1.005	9.07E-01

cds.Camellia_sinensis.comp24053_c0_seq4_m.11134	PREDICTED: probable Xaa-Pro aminopeptidase P [Vitis vinifera]	23.791	8.4049	8.8	3	4	3	0.994	1.055	0.933	1.004	0.997	1.015	1.011	7.59E-01
cds.Camellia_sinensis.comp24057_c0_seq1_m.30493	PREDICTED: nuclear pore complex protein NUP35 [Ipomoea nil]	35.989	7.8803	10.3	3	3	3	0.881	1.251	0.974	1.001	0.885	1.017	0.935	6.33E-01
cds.Camellia_sinensis.comp24068_c0_seq1_m.34682	calcium-binding EF hand family protein [Populus trichocarpa]	18.464	8.907	23.6	4	10	4	0.962	1.028	0.961	1.016	1.028	1.033	1.043	1.38E-01
cds.Camellia_sinensis.comp24075_c0_seq6_m.36125	PREDICTED: rhodanese-like domain-containing protein 9, chloroplastic [Vitis vinifera]	25.977	18.236	24.7	6	11	6	1.041	1.005	1.039	0.961	0.959	0.979	0.94	9.22E-03

cds.Camellia_sinensis.comp24077_c0_seq1_m.23151	PREDICTED: 3-isopropylmalate dehydrogenase, chloroplastic [Vitis vinifera]	43.362	45.791	26.2	11	21	11	1.015	1.045	1.008	1	0.976	0.972	0.961	4.81E-02
cds.Camellia_sinensis.comp24078_c0_seq2_m.19441	PREDICTED: SURP and G-patch domain-containing protein 1-like protein isoform X1 [Vitis vinifera]	47.91	2.7308	5.7	2	2	2								
cds.Camellia_sinensis.comp24094_c0_seq1_m.8183	PREDICTED: ATP-dependent DNA helicase RecQ-like [Prunus mume]	99.53	1.4642	1.8	2	2	2	0.975	0.911	1.011	1.068	1.017	1.04	1.079	8.54E-02

cds.Camellia_sinensis.comp24100_c0_seq1_m.27039	PREDICTED: LOW QUALITY PROTEIN: cell division protein FtsZ homolog 1, chloroplastic-like [Capsicum	44.362	25.589	14.9	5	6	5	1.083	1.073	1.113	0.844	0.86	1.001	0.827	2.52E-02
cds.Camellia_sinensis.comp24104_c0_seq1_m.25122	PREDICTED: pentatricopeptide repeat-containing protein At1g61870, mitochondrial [Vitis vinifera]	44.525	2.8009	3.8	2	2	2	1.108	1.105	1.078	0.922	0.918	0.847	0.816	2.16E-03
cds.Camellia_sinensis.comp24112_c0_seq1_m.37222	resistance to phytophthora protein, putative [Medicago	26.883	1.3934	3.4	1	1	1	1.03	0.98	1.028	0.93	1.031	1.002	0.975	5.01E-01

cds.Camellia_sinensis.comp24113_c1_seq1_m.35659	PREDICTED: alpha-1,4 glucan phosphorylase L-2 isozyme, chloroplastic/amyloplastic-like [Ipomoea nil]	18.337	14.069	30	5	8	4	0.952	1.048	0.957	0.967	1.075	0.982	1.023	6.49E-01
cds.Camellia_sinensis.comp24118_c0_seq1_m.41021	PREDICTED: phenylalanine ammonia-lyase [Eucalyptus grandis]	31.858	3.0121	22.6	5	9	2	1.095	1.193	1.162	0.847	0.873	0.795	0.729	1.08E-03
cds.Camellia_sinensis.comp24120_c0_seq2_m.27368	catalytic/coenzyme binding protein [Arabidopsis lyrata subsp. lyrata]	38.646	84.985	29.6	11	24	11	0.987	0.959	0.932	1.016	1.014	1.085	1.082	4.66E-02
cds.Camellia_sinensis.comp24125_c0_seq1_m.34400	PREDICTED: nuclear pore complex protein NUP54-like [Gossypium hirsutum]	38.664	3.1382	3.4	1	1	1	0.918	1.473	0.889	1.043	0.883	0.793	0.829	4.12E-01

cds.Camellia_sinensis.comp24128_c0_seq1_m.15428	PREDICTED: cytochrome P450 97B2, chloroplastic [Vitis vinifera]	58.713	41.12	16	8	18	8	1.016	1.031	1.059	1.038	0.945	0.942	0.942	1.48E-01
cds.Camellia_sinensis.comp24139_c0_seq1_m.34363	PREDICTED: probable aminopyrimidine aminohydrolase, mitochondrial [Nicotiana tabacum]	43.544	3.955	5.7	2	3	1	0.952	1.013	0.997	1.027	0.96	1.067	1.031	4.53E-01
cds.Camellia_sinensis.comp24152_c0_seq1_m.44297	PREDICTED: cysteine proteinase 15A-like [Ziziphus jujuba]	28.609	7.9937	16.3	4	5	3	1.014	0.938	0.957	0.982	1.065	1.063	1.069	1.34E-01
cds.Camellia_sinensis.comp24163_c0_seq1_m.42497	PREDICTED: ribosome-binding factor PSRP1, chloroplastic [Citrus sinensis]	25.735	3.7748	4.3	2	3	2	1.181	1.215	1.211	0.784	0.813	0.762	0.654	3.69E-05

cds.Camellia_sinensis.comp24164_c0_seq1_m.25907	PREDICTED: protein ECERIFERUM 26 [Vitis vinifera]	49.78	18.322	13.3	5	8	5	1.223	1.255	1.319	0.68	0.807	0.629	0.557	1.56E-03
cds.Camellia_sinensis.comp24165_c0_seq1_m.18657	PREDICTED: serine-threonine kinase receptor-associated protein-like [Ziziphus jujuba]	37.337	75.928	29	9	15	9	0.962	0.987	0.958	1.028	1.038	1.024	1.063	3.84E-03
cds.Camellia_sinensis.comp24168_c0_seq1_m.26608	ribosomal RNA small subunit methyltransferase J [Arabidopsis	41.529	44.173	17.8	5	9	5	1.074	1.107	1.229	0.87	0.803	0.843	0.738	2.98E-03
cds.Camellia_sinensis.comp24176_c0_seq1_m.23265	PREDICTED: mediator-associated protein 1 [Cucumis sativus]	41.899	10.368	9.3	3	4	3	1.019	0.993	1.034	0.948	0.863	1.079	0.949	4.38E-01

cds.Camellia_sinensis.comp24182_c0_seq1_m.28653	PREDICTED: L-galactose dehydrogenase [Fragaria vesca subsp. vesca]	34.57	51.297	27.6	6	8	6	0.965	1.022	1.006	0.951	1.022	1.009	0.996	8.97E-01
cds.Camellia_sinensis.comp24187_c0_seq2_m.39619	PREDICTED: protein PEROXIN-4 [Ipomoea nil]	17.701	2.7964	10.8	1	1	1								
cds.Camellia_sinensis.comp24196_c0_seq1_m.46591	PREDICTED: calcium-binding allergen Ole e 8 [Solanum tuberosum	19.086	63.181	36.4	5	10	5	0.954	0.948	0.977	1.064	1.04	1.037	1.091	2.08E-03
cds.Camellia_sinensis.comp24198_c0_seq1_m.52314	PREDICTED: glutathione S-transferase L3 isoform X1 [Vitis vinifera]	18.659	23.355	31.5	4	8	4	1.01	1.078	1.062	0.925	0.984	0.984	0.918	4.05E-02

cds.Camellia_sinensis.comp24205_c0_seq1_m.43355	PREDICTED: ferredoxin-thioredoxin reductase catalytic chain, chloroplastic [Vitis	16.333	32.415	36.1	6	12	6	0.987	1.03	0.971	1.012	1.022	0.997	1.014	4.87E-01
cds.Camellia_sinensis.comp24206_c0_seq1_m.41922	PREDICTED: CASP-like protein 1 [Sesamum indicum]	20.47	14.223	26.4	5	9	5	0.785	0.771	0.76	1.206	1.294	1.253	1.62	2.38E-05
cds.Camellia_sinensis.comp24207_c0_seq1_m.26848	PREDICTED: ATP-citrate synthase alpha chain protein 2 [Vitis vinifera]	46.486	26.406	12.8	6	12	3	1.043	0.971	1.002	1.007	1.021	1.04	1.017	4.85E-01
cds.Camellia_sinensis.comp24208_c0_seq1_m.8550	PREDICTED: DEAD-box ATP-dependent RNA helicase 37 [Vitis vinifera]	66.283	26.201	23.9	12	14	11	0.936	0.993	0.962	1.027	1.042	1.037	1.074	1.50E-02

cds.Camellia_sinensis.comp24209_c0_seq1_m.15120	PREDICTED: fatty acid amide hydrolase-like [Solanum tuberosum	41.129	9.147	4.4	1	1	1	1.068	0.937	0.798	0.999	1.129	1.081	1.145	1.98E-01
cds.Camellia_sinensis.comp24209_c0_seq1_m.15121	PREDICTED: fatty acid amide hydrolase-like [Nicotiana attenuata]	27.133	10.564	4.6	1	1	1	1.03	1.116	1.046	0.957	0.9	0.942	0.877	1.29E-02
cds.Camellia_sinensis.comp24213_c0_seq1_m.7983	PREDICTED: ABC transporter F family member 3 [Vitis vinifera]	79.627	23.515	10.5	8	8	8	0.964	1.023	0.953	1.066	0.969	1.024	1.04	3.27E-01
cds.Camellia_sinensis.comp24217_c0_seq2_m.21346	PREDICTED: acyl-coenzyme A oxidase 4, peroxisomal isoform X2 [Vitis vinifera]	47.726	56.518	26.1	11	16	2	0.997	0.905	0.914	0.987	1.098	1.118	1.137	6.09E-02
cds.Camellia_sinensis.comp24234_c0_seq2_m.45260	PREDICTED: EKC/KEOPS complex subunit Tprkb [Jatropha curcas]	18.591	4.9074	17	2	3	2	1.012	0.944	0.9	1.024	1	1.134	1.106	1.23E-01

cds.Camellia_sinensis.comp24236_c0_seq1_m.38194	PREDICTED: protein LSM12 homolog A [Sesamum	22.235	1.1947	3.5	1	1	1	0.987	1.01	1.014	0.957	0.979	1.059	0.995	8.55E-01
cds.Camellia_sinensis.comp24239_c0_seq1_m.27428	S-adenosylmethionine synthase 2 [Nicotiana tabacum]	40.335	3.5347	21.9	8	18	3	1.115	1.095	1.068	0.893	0.923	0.882	0.823	4.57E-04
cds.Camellia_sinensis.comp24241_c0_seq1_m.41166	PREDICTED: polyubiquitin 9 [Nelumbo nucifera]	15.746	11.901	33.8	3	3	3	1.01	1.063	0.995	0.977	0.981	0.978	0.957	1.62E-01
cds.Camellia_sinensis.comp24243_c0_seq8_m.7109	PREDICTED: CCR4-NOT transcription complex subunit 3 isoform X2 [Vitis vinifera]	94.569	4.8684	4.2	4	4	4	1.069	1.088	1.149	0.887	0.924	0.888	0.816	1.36E-03

cds.Camellia_sinensis.comp24249_c0_seq1_m.39115	PREDICTED: aminoacyl tRNA synthase complex-interacting multifunctional protein 1 [Juglans regia]	32.157	16.547	19	5	7	5	0.952	1.078	1.003	1.019	1.012	0.921	0.973	6.10E-01
cds.Camellia_sinensis.comp24253_c0_seq1_m.39812	PREDICTED: probable protein phosphatase 2C 39 [Nicotiana tabacum]	22.663	11.711	19.3	4	8	2	0.969	1.027	0.988	1.014	1.039	0.979	1.016	5.47E-01
cds.Camellia_sinensis.comp24256_c0_seq1_m.23885	PREDICTED: GDT1-like protein 2, chloroplastic [Vitis vinifera]	30.909	27.167	10	2	4	2	1.012	0.938	0.913	1.014	1.022	1.053	1.079	7.98E-02
cds.Camellia_sinensis.comp24269_c0_seq1_m.20207	PREDICTED: histone acetyltransferase type B catalytic subunit [Populus euphratica]	55.461	5.7439	3.1	1	1	1	0.837	1.028	0.977	1.053	0.833	1.301	1.121	5.15E-01

cds.Camellia_sinensis.comp24271_c0_seq1_m.20938	PREDICTED: glucan endo-1,3-beta-glucosidase 7 [Ricinus	50.935	-2	4.2	2	3	1								
cds.Camellia_sinensis.comp24271_c0_seq2_m.36515	PREDICTED: glucan endo-1,3-beta-glucosidase 7-like [Nicotiana tomentosif	37.043	1.851	2.6	1	1	1								
cds.Camellia_sinensis.comp24298_c0_seq1_m.23229	PREDICTED: 3-ketoacyl-CoA synthase 6 [Sesamum indicum]	56.181	4.7365	4.4	2	3	2	1.558	1.615	1.613	0.265	0.345	0.41	0.213	6.60E-03
cds.Camellia_sinensis.comp24302_c0_seq1_m.35823	PREDICTED: 2-methoxy-6-polyprenyl-1,4-benzoquinol methylase, mitochondrial-like [Jatropha	32.437	6.4349	7.3	2	2	2	0.975	1	0.984	1.01	0.972	1.071	1.032	3.54E-01

cds.Camellia_sinensis.comp24308_c0_seq1_m.21367	retinal-binding protein [Arabidopsis thaliana]	50.785	15.149	11.8	5	5	5	0.971	1.029	0.989	1.022	0.989	1.014	1.012	5.72E-01
cds.Camellia_sinensis.comp24317_c0_seq1_m.36665	PREDICTED: peptide methionine sulfoxide reductase B5-like [Juglans regia]	25.267	5.6683	12.1	3	3	2	0.979	1.059	1.049	0.972	0.987	0.96	0.946	1.00E-01
cds.Camellia_sinensis.comp24327_c0_seq1_m.33972	PREDICTED: probable plastid-lipid-associated protein 8, chloroplastic [Nicotiana attenuata]	27.086	24.958	22.3	5	11	5	1.013	1.074	1.022	1.068	0.966	0.98	0.969	4.31E-01
cds.Camellia_sinensis.comp24332_c0_seq1_m.38647	PREDICTED: exosome complex component CSL4 [Juglans regia]	21.225	1.578	5.1	1	1	1	0.952	1.094	0.884	1.072	1.036	0.981	1.054	4.53E-01

cds.Camellia_sinensis.comp24339_c0_seq1_m.48113	PREDICTED: protein TOC75-3, chloroplastic [Nelumbo nucifera]	22.376	1.9765	4.2	1	2	1	0.963	1.001	1.027	0.959	1.127	0.939	1.011	8.98E-01
cds.Camellia_sinensis.comp24347_c0_seq2_m.31018	PREDICTED: WD repeat-containing protein VIP3 [Nelumbo nucifera]	34.135	5.0643	8.1	3	3	3	1.028	0.958	0.977	0.943	1.05	1.057	1.029	5.46E-01
cds.Camellia_sinensis.comp24348_c0_seq1_m.32293	PREDICTED: inositol polyphosphate multikinase beta [Vitis vinifera]	35.803	8.9307	14.2	4	7	4	0.991	0.992	1.008	1.019	0.977	0.997	1.001	9.69E-01
cds.Camellia_sinensis.comp24354_c0_seq1_m.32753	PREDICTED: ankyrin repeat and SOCS box protein 13 [Citrus sinensis]	23.706	3.5733	8.7	2	2	2	0.896	1.068	0.91	1.061	1.087	1.008	1.098	1.86E-01
cds.Camellia_sinensis.comp24358_c0_seq1_m.18142	neuronal PAS domain protein [Arabidopsis thaliana]	61.288	2.8167	1.8	1	1	1								

cds.Camellia_sinensis.comp24369_c0_seq1_m.18459	PREDICTED: vacuolar protein sorting-associated protein 4-like [Sesamum indicum]	48.009	10.19	13.2	4	5	4								
cds.Camellia_sinensis.comp24377_c0_seq1_m.25327	PREDICTED: ATG8-interacting protein 1 [Vitis vinifera]	35	2.0884	3.8	1	1	1								
cds.Camellia_sinensis.comp24380_c0_seq2_m.32547	PREDICTED: cytochrome c oxidase subunit 5b-1, mitochondrial [Erythranthe guttata]	18.348	48.906	41.2	7	14	7	0.961	0.97	0.95	1.048	1.068	1.008	1.084	1.11E-02
cds.Camellia_sinensis.comp24387_c0_seq1_m.37312	PREDICTED: temperature-induced lipocalin-1-like [Cucumis melo]	21.394	4.5388	18.4	4	8	2	1.025	1.141	1.112	0.822	0.997	0.885	0.825	3.99E-02

cds.Camellia_sinensis.comp24387_c0_seq2_m.41361	PREDICTED: apolipoprotein D [Populus euphratica]	21.366	133.38	56.5	12	51	10	0.942	0.896	0.908	1.084	1.102	1.097	1.196	3.42E-04
cds.Camellia_sinensis.comp24391_c0_seq1_m.25946	PREDICTED: aminoacylase-1-like [Juglans regia]	53.18	14.995	18.9	9	9	9	1.055	1.074	1.042	0.91	0.956	0.933	0.883	1.72E-03
cds.Camellia_sinensis.comp24396_c0_seq1_m.28042	PREDICTED: probable methyltransferase PMT2 [Fragaria vesca subsp. vesca]	40.978	1.4661	5	2	2	2	1.294	1.166	1.112	0.787	0.737	0.835	0.66	1.96E-03
cds.Camellia_sinensis.comp24397_c0_seq1_m.33080	PREDICTED: LOW QUALITY PROTEIN: basic endochitinase [Vitis vinifera]	36.672	21.069	4.1	1	1	1	0.826	0.854	0.872	1.105	1.272	1.132	1.375	2.38E-03
cds.Camellia_sinensis.comp24400_c0_seq1_m.35973	PREDICTED: ER membrane protein complex subunit 7 homolog [Theobroma cacao]	23.115	4.7878	11.4	2	3	2	0.95	1.073	0.976	1.104	0.94	0.976	1.007	9.27E-01

cds.Camellia_sinensis.comp24411_c0_seq1_m.4822	PREDICTED: ER membrane protein complex subunit 1 isoform X2 [Theobroma cacao]	57.181	39.598	16	7	11	7	0.954	0.965	0.93	1.092	1.042	1.046	1.116	4.10E-03
cds.Camellia_sinensis.comp24411_c0_seq1_m.4823	PREDICTED: ER membrane protein complex subunit 1 [Jatropha curcas]	54.821	30.399	18.2	7	12	7	0.942	0.953	1.009	1.041	1.052	1.017	1.071	4.26E-02
cds.Camellia_sinensis.comp24412_c0_seq2_m.45149	histone H2A variant 1 [Ananas comosus]	18.569	10.066	20.5	4	8	3	1.096	1.131	1.175	0.896	0.838	0.84	0.757	7.21E-04
cds.Camellia_sinensis.comp24413_c0_seq3_m.43397	PREDICTED: putative phosphatidylglycerol/phosphatidylinositol transfer protein DDB_G0282179 [Vitis vinifera]	21.198	28.096	25.3	5	14	5	1.01	0.987	1.018	1.001	0.985	1.007	0.993	5.57E-01

cds.Camellia_sinensis.comp24422_c1_seq1_m.11497	PREDICTED: protein SUPPRESSOR OF GENE SILENCING 3 [Vitis vinifera]	73.283	35.512	14.4	9	10	9	1.125	1.127	1.177	0.849	0.871	0.817	0.74	2.21E-04
cds.Camellia_sinensis.comp24434_c0_seq1_m.27593	Leucine-rich repeat (LRR) family protein [Arabidopsis thaliana]	49.839	5.4427	10.3	4	5	4								
cds.Camellia_sinensis.comp24440_c0_seq1_m.28957	PREDICTED: ATP synthase gamma chain, chloroplastic [Nelumbo nucifera]	41.352	259.9	41.3	17	65	15	1.016	0.977	0.966	1.028	1.017	1.019	1.035	8.83E-02
cds.Camellia_sinensis.comp24446_c0_seq1_m.37788	PREDICTED: 30S ribosomal protein S9, chloroplastic-like [Nelumbo nucifera]	22.414	6.9776	14.5	3	4	3	1.162	1.171	1.17	0.787	0.83	0.798	0.689	1.88E-03

cds.Camellia_sinensis.comp24452_c0_seq2_m.45813	PREDICTED: histone-lysine N-methyltransferase setd3 [Nelumbo nucifera]	26.399	1.3065	5.4	1	1	1								
cds.Camellia_sinensis.comp24460_c0_seq1_m.6305	PREDICTED: isoamylase 2, chloroplastic [Juglans regia]	96.791	8.0471	5.9	5	5	5	1.01	1.016	1.025	0.949	0.989	0.969	0.953	1.87E-02
cds.Camellia_sinensis.comp24465_c0_seq1_m.43939	PREDICTED: histone deacetylase complex subunit SAP18 [Sesamum indicum]	18.517	6.1553	15.2	3	7	3	1.005	1	0.966	1	1.042	1.01	1.027	2.00E-01
cds.Camellia_sinensis.comp24471_c0_seq1_m.35807	PREDICTED: REF/SRPP-like protein At1g67360 [Nicotiana	28.646	37.627	28.5	6	10	6	0.915	0.878	0.892	1.106	1.092	1.106	1.231	8.17E-05

cds.Camellia_sinensis.comp24482_c0_seq1_m.56983	PREDICTED: guanylate-binding protein 7 isoform X2 [Ziziphus jujuba]	13.418	1.8617	19.3	3	3	1	0.955	1.088	1.011	1.011	0.91	1.034	0.968	5.77E-01
cds.Camellia_sinensis.comp24497_c0_seq17_m.37976	PREDICTED: 50S ribosomal protein L9, chloroplastic [Daucus carota subsp. sativus]	23.954	10.995	24.6	6	8	6	1.073	1.061	1.102	0.881	0.884	0.94	0.836	1.68E-03
cds.Camellia_sinensis.comp24498_c0_seq3_m.31193	PREDICTED: phosphoserine phosphatase, chloroplastic [Vitis vinifera]	32.001	66.03	32.9	8	15	8	1.004	1.077	1.107	0.942	0.942	0.952	0.89	5.81E-02
cds.Camellia_sinensis.comp24515_c0_seq2_m.18878	PREDICTED: scarecrow-like protein 21 [Juglans regia]	59.071	1.6917	1.7	1	1	1								
cds.Camellia_sinensis.comp24526_c0_seq2_m.10718	DUF639 family protein [Medicago truncatula]	61.831	-2	1.3	1	1	1	1.092	0.849	1.006	0.931	1.088	1.035	1.036	6.78E-01

cds.Camellia_sinensis.comp24527_c0_seq1_m.54512	palmitoyl-protein thioesterase 1-like [Cajanus cajan]	15.457	17.119	33.8	3	5	3	1.068	1.056	1.053	0.917	0.995	0.947	0.9	1.18E-02
cds.Camellia_sinensis.comp24528_c0_seq1_m.3142	NADH-plastoquinone oxidoreductase subunit K (chloroplast) [Camellia pubicosta]	32.237	48.051	12.4	3	5	3	0.919	0.963	0.97	1.026	0.988	1.059	1.077	4.69E-02
cds.Camellia_sinensis.comp24528_c0_seq1_m.3143	NADH-plastoquinone oxidoreductase subunit J (chloroplast) [Camellia crapnelliana]	18.603	14.93	22.2	3	6	3	0.945	0.991	0.969	0.994	1.062	1.068	1.075	5.43E-02
cds.Camellia_sinensis.comp24531_c1_seq1_m.29838	PREDICTED: activator of 90 kDa heat shock protein ATPase homolog [Juglans regia]	39.988	69.611	24.1	8	14	8	1.033	1.023	1.024	0.93	0.987	0.985	0.942	9.36E-02

cds.Camellia_sinensis.comp24543_c0_seq1_m.41193	PREDICTED: putative phosphatidylglycerol/phosphatidylinositol transfer protein DDB_G0282179 [Jatropha curcas]	21.35	14.003	13.8	2	4	2	1.161	1	0.962	0.887	0.983	0.985	0.914	2.61E-01
cds.Camellia_sinensis.comp24545_c0_seq1_m.37377	PREDICTED: multiple organellar RNA editing factor 8, chloroplastic/mitochondrial-like [Nicotiana tabacum]	35.289	75.116	14	4	9	4	0.968	1.006	0.986	0.985	1.045	1.027	1.033	1.98E-01
cds.Camellia_sinensis.comp24556_c0_seq1_m.40240	PREDICTED: CDGSH iron-sulfur domain-containing protein NEET [Nelumbo nucifera]	12.864	65.622	46.1	5	9	5	1.002	0.964	0.983	1.037	1.071	0.997	1.053	9.49E-02

cds.Camellia_sinensis.comp24560_c0_seq1_m.30511	PREDICTED: high mobility group B protein 1-like [Nelumbo nucifera]	19.386	3.8063	13.2	3	5	3	0.995	0.99	0.988	0.996	0.98	1.029	1.011	5.44E-01
cds.Camellia_sinensis.comp24563_c0_seq1_m.34737	PREDICTED: proteasome subunit alpha type-1-B [Sesamum indicum]	30.23	110.66	48.7	13	29	13	0.983	0.972	0.967	1.024	1.031	1.04	1.059	9.58E-04
cds.Camellia_sinensis.comp24569_c0_seq1_m.41022	Ran-binding protein 1-b-like protein [Morus notabilis]	25.916	22.214	20.2	5	9	3	1.098	1.084	1.033	0.898	0.921	0.905	0.847	1.18E-03
cds.Camellia_sinensis.comp24569_c0_seq2_m.50549	Ran-binding protein 1-b-like protein [Morus notabilis]	20.64	-2	28.3	4	6	1								
cds.Camellia_sinensis.comp24570_c0_seq1_m.46922	PREDICTED: S-adenosylmethionine synthase 5 [Prunus mume]	15.734	-2	31.2	4	11	1	1.165	1.143	1.073	0.821	0.9	0.858	0.763	1.74E-03

cds.Camellia_sinensis.comp24575_c0_seq1_m.42598	PREDICTED: stress enhanced protein 2, chloroplastic [Vitis vinifera]	20.571	5.1062	6.9	1	2	1	1.031	1.032	1.05	0.969	0.924	0.992	0.927	2.44E-02
cds.Camellia_sinensis.comp24576_c0_seq1_m.41411	PREDICTED: aquaporin TIP2-1 [Theobroma cacao]	27.788	13.581	4.1	1	5	1	0.984	0.957	1.038	1.018	1.008	0.966	1.004	8.79E-01
cds.Camellia_sinensis.comp24592_c0_seq1_m.28083	PREDICTED: fructose-bisphosphate aldolase-lysine N-methyltransferase, chloroplastic [Gossypium raimondii]	35.355	82.904	29.7	7	14	7	0.999	0.994	0.91	1.051	1.011	1.05	1.072	9.95E-02
cds.Camellia_sinensis.comp24611_c0_seq1_m.24117	PREDICTED: probable purple acid phosphatase 20 [Nicotiana tomentosiformis]	47.727	6.0496	4.7	2	2	2	0.858	0.818	0.778	1.242	1.167	1.204	1.472	3.20E-04

cds.Camellia_sinensis.comp24615_c0_seq1_m.20658	PREDICTED: pentatricopeptide repeat-containing protein At1g08070, chloroplastic [Ricinus communis]	51.183	-2	3.1	1	1	1	0.818	0.982	1.062	1.109	1.148	0.93	1.114	3.41E-01
cds.Camellia_sinensis.comp24618_c0_seq1_m.22659	PREDICTED: acyl-coenzyme A thioesterase 9, mitochondrial [Ricinus communis]	58.206	5.288	3.9	3	3	3	1.101	1.114	1.069	0.928	0.867	0.899	0.82	1.02E-03
cds.Camellia_sinensis.comp24620_c0_seq1_m.39124	PREDICTED: ELAV-like protein 4 [Capsicum annuum]	16.488	8.0107	17.4	3	4	3	0.93	0.946	0.981	1.036	1.058	1.056	1.103	4.64E-03
cds.Camellia_sinensis.comp24637_c0_seq1_m.8160	PREDICTED: subtilisin-like protease SBT1.7 [Theobroma cacao]	81.53	12.217	1.9	1	4	1	0.975	1.027	1.047	0.964	1.026	0.967	0.97	3.57E-01

cds.Camellia_sinensis.comp24647_c0_seq1_m.25981	PREDICTED: pentatricopeptide repeat-containing protein At3g13160, mitochondrial-like [Vitis vinifera]	44.264	8.8348	15.5	7	8	7	0.971	0.975	0.985	0.982	1	1.016	1.023	1.03E-01
cds.Camellia_sinensis.comp24652_c0_seq1_m.29323	PREDICTED: COP9 signalosome complex subunit 5b [Vitis	45.285	7.5576	4.7	2	2	2	0.986	0.955	0.974	1.097	1.001	1.008	1.066	1.12E-01
cds.Camellia_sinensis.comp24653_c0_seq2_m.31027	PREDICTED: 2-alkenal reductase (NADP(+)-dependent) [Vitis vinifera]	37.835	71.031	33.7	11	31	11	0.927	0.907	0.89	1.103	1.096	1.111	1.215	9.79E-05
cds.Camellia_sinensis.comp24656_c0_seq1_m.43752	PREDICTED: acyl-coenzyme A thioesterase 9, mitochondrial-like [Nelumbo nucifera]	22.055	3.1935	15.3	2	2	2								

cds.Camellia_sinensis.comp24675_c0_seq1_m.31705	PREDICTED: malate dehydrogenase, glyoxysomal [Vitis vinifera]	38.02	120.06	36.8	11	36	6	1.007	1.015	1.017	1.008	0.979	1.01	0.986	2.54E-01
cds.Camellia_sinensis.comp24686_c0_seq1_m.24876	PREDICTED: developmentally-regulated G-protein 3 [Arachis ipaensis]	41.255	4.0422	6	2	3	2								
cds.Camellia_sinensis.comp24691_c0_seq1_m.35394	PREDICTED: peptide deformylase 1A, chloroplastic [Nelumbo nucifera]	31.64	20.343	21.1	6	10	6	0.999	1.034	1.01	0.99	0.997	1.004	0.983	1.92E-01
cds.Camellia_sinensis.comp24697_c0_seq1_m.34694	PREDICTED: ABC transporter I family member 17 [Citrus sinensis]	31.094	15.69	25	6	7	6	1.011	1.059	1.068	0.907	0.946	0.954	0.895	8.54E-03

cds.Camellia_sinensis.comp24698_c0_seq1_m.27987	PREDICTED: protein THYLAKOID FORMATION1, chloroplastic [Theobroma cacao]	33.604	36.759	24.9	9	19	7	1.002	0.999	1.021	0.97	1.01	1.004	0.987	4.23E-01
cds.Camellia_sinensis.comp24698_c0_seq2_m.30932	PREDICTED: protein THYLAKOID FORMATION1, chloroplastic [Theobroma cacao]	33.385	33.489	15.8	4	6	2	0.979	0.984	0.976	1.01	1.064	0.919	1.018	7.39E-01
cds.Camellia_sinensis.comp24699_c0_seq1_m.33754		44.555	3.2901	3.8	1	1	1								
cds.Camellia_sinensis.comp24702_c0_seq1_m.41887	PREDICTED: flavonoid 3'-monooxygenase-like [Sesamum indicum]	32.159	10.81	11.5	4	5	3	1.072	1.069	1.069	0.934	0.856	0.983	0.864	6.71E-02

cds.Camellia_sinensis.comp24714_c0_seq1_m.50851	PREDICTED: superoxide dismutase [Cu-Zn], chloroplastic [Ziziphus jujuba]	16.015	46.255	24.2	3	7	2	0.914	0.901	0.887	1.087	1.112	1.129	1.232	1.20E-04
cds.Camellia_sinensis.comp24729_c0_seq1_m.48307	PREDICTED: 50S ribosomal protein L31, chloroplastic [Vitis vinifera]	15.72	8.7437	8.6	1	2	1	1.267	1.176	1.112	0.759	0.825	0.797	0.67	8.78E-04
cds.Camellia_sinensis.comp24732_c0_seq1_m.20638	PREDICTED: ankyrin-1 [Theobroma cacao]	49.125	25.226	8.3	3	4	3	0.97	1.004	0.992	1.072	0.989	0.991	1.029	3.80E-01
cds.Camellia_sinensis.comp24762_c0_seq1_m.20309	PREDICTED: pentatricopeptide repeat-containing protein At1g60770 isoform X1 [Vitis vinifera]	56.417	1.8303	1.8	1	1	1	1.029	1.195	1.103	0.876	0.898	0.878	0.797	6.88E-03

cds.Camellia_sinensis.comp24764_c0_seq1_m.35070	PREDICTED: 2-hydroxyisoflavanone dehydratase-like [Juglans regia]	36.259	45.113	23.2	8	15	8	1.104	1.077	1.072	0.899	0.892	0.891	0.824	3.77E-05
cds.Camellia_sinensis.comp24776_c0_seq1_m.41694	PREDICTED: V-type proton ATPase subunit E-like [Nelumbo	26.154	-2	18.3	4	6	1								
cds.Camellia_sinensis.comp24793_c0_seq2_m.35781	PREDICTED: thiosulfate sulfurtransferase 18 isoform X2 [Solanum tuberosum	18.216	1.5694	6.1	1	2	1								
cds.Camellia_sinensis.comp24796_c0_seq1_m.33620	PREDICTED: malignant T-cell-amplified sequence 1 homolog [Vitis vinifera]	20.114	38.701	23.2	4	4	4	1.005	1.05	0.931	1.013	1.001	1.007	1.012	7.50E-01

cds.Camellia_sinensis.comp24798_c0_seq3_m.39242	PREDICTED: protein OSB2, chloroplastic-like [Nicotiana tomentosiformis]	29.829	11.605	8.8	2	2	2	0.956	0.995	1.02	1.01	0.947	1.085	1.024	6.34E-01
cds.Camellia_sinensis.comp24800_c0_seq1_m.24723	PREDICTED: cysteine desulfurase 1, chloroplastic [Juglans regia]	38.377	12.252	7.2	2	3	2	0.99	1.026	1.057	1.076	0.985	0.88	0.957	4.86E-01
cds.Camellia_sinensis.comp24800_c0_seq1_m.24724	PREDICTED: cysteine desulfurase 1, chloroplastic [Juglans regia]	13.55	30.472	28.5	4	5	4	1.005	1.04	1.021	0.961	0.979	0.991	0.956	2.79E-02
cds.Camellia_sinensis.comp24811_c0_seq1_m.33849	PREDICTED: polyadenylate-binding protein 2-like isoform X1 [Juglans	25.195	2.9257	8.6	2	5	1	0.999	0.979	1.003	1.031	1.016	0.978	1.015	4.53E-01

cds.Camellia_sinensis.comp24815_c0_seq1_m.38451	PREDICTED: sm-like protein LSM1B [Ziziphus jujuba]	14.502	1.6751	12.5	1	1	1	0.876	1.278	1.054	1.151	0.925	0.738	0.877	4.66E-01
cds.Camellia_sinensis.comp24818_c0_seq1_m.28200	PREDICTED: phenylalanine ammonia-lyase 1 [Daucus carota subsp. sativus]	45.753	19.992	40.3	16	32	7	1.376	1.315	1.268	0.616	0.661	0.625	0.48	2.07E-05
cds.Camellia_sinensis.comp24820_c0_seq1_m.37845	PREDICTED: expansin-like A2 [Ricinus communis]	16.401	18.858	37.2	6	13	6	0.795	0.877	0.785	1.213	1.189	1.19	1.462	4.37E-04
cds.Camellia_sinensis.comp24823_c0_seq2_m.39697	PREDICTED: surfeit locus protein 1 isoform X1 [Theobroma cacao]	27.913	5.6172	8.4	1	1	1	0.94	1.094	1.075	1.003	0.946	0.952	0.933	2.60E-01
cds.Camellia_sinensis.comp24827_c1_seq1_m.11253	PREDICTED: outer envelope protein 80, chloroplastic-like [Gossypium hirsutum]	74.32	60.844	20.8	15	21	15	0.977	0.983	0.986	1.035	1.049	1.022	1.054	2.72E-03

cds.Camellia_sinensis.comp24834_c0_seq1_m.22223	PREDICTED: 30S ribosomal protein S1, chloroplastic [Nelumbo nucifera]	44.723	86.905	30.7	12	36	12	1.022	1.003	1.026	0.974	0.979	0.981	0.962	5.92E-03
cds.Camellia_sinensis.comp24836_c0_seq1_m.42562	PREDICTED: protein translation factor SUI1 homolog [Nelumbo nucifera]	11.503	2.1725	8.8	1	1	1	1.004	0.985	0.998	0.965	1.068	0.989	1.012	7.47E-01
cds.Camellia_sinensis.comp24839_c0_seq2_m.23278	PREDICTED: thiol-disulfide oxidoreductase LTO1-like [Juglans regia]	36.864	2.5888	3.8	1	1	1								
cds.Camellia_sinensis.comp24851_c0_seq1_m.28271	PREDICTED: 40S ribosomal protein S18 [Erythranthe guttata]	17.698	121.85	50	7	15	7	1.128	1.127	1.147	0.82	0.861	0.833	0.739	4.01E-05
cds.Camellia_sinensis.comp24864_c0_seq1_m.40533		19.3	7.8641	11	2	4	2	1.148	1.107	1.083	0.881	0.869	0.88	0.788	1.76E-04

cds.Camellia_sinensis.comp24869_c0_seq1_m.29354	PREDICTED: pyridoxal reductase, chloroplastic isoform X2 [Vitis vinifera]	42.971	56.826	13.5	4	8	4	0.934	0.961	1.011	1.024	1.048	1.041	1.071	4.56E-02
cds.Camellia_sinensis.comp24875_c0_seq1_m.21153	PREDICTED: 60S ribosomal protein L4 [Ziziphus jujuba]	44.76	78.066	38	13	22	8	1.125	1.15	1.145	0.848	0.853	0.857	0.748	1.25E-06
cds.Camellia_sinensis.comp24877_c0_seq2_m.56801	PREDICTED: probable carboxylesterase 2 [Vitis vinifera]	12.678	4.7303	25.8	3	5	2	0.929	0.932	0.946	1.031	1.147	1.047	1.149	1.51E-02
cds.Camellia_sinensis.comp24881_c0_seq1_m.50454	PREDICTED: aquaporin PIP1-3 [Theobroma cacao]	15.394	5.476	19.9	2	2	2	0.943	0.949	0.904	1.123	1.049	1.066	1.158	4.62E-03
cds.Camellia_sinensis.comp24882_c0_seq1_m.23939	PREDICTED: aspartic proteinase nepenthesin-1 [Sesamum indicum]	46.761	37.286	11.7	4	8	4	0.984	0.92	0.979	1.092	1.065	1.054	1.114	1.08E-02

cds.Camellia_sinensis.comp24886_c1_seq1_m.31333	PREDICTED: protein PAT1 homolog 1 [Vitis vinifera]	47.621	2.0401	4.2	1	1	1								
cds.Camellia_sinensis.comp24889_c0_seq2_m.48066	PREDICTED: putative uridine kinase C227.14 isoform X2 [Theobroma cacao]	18.111	1.2708	8	1	1	1								
cds.Camellia_sinensis.comp24904_c0_seq1_m.37443	PREDICTED: histone H1.1-like [Tarenaya hassleriana]	25.451	2.4282	4.1	1	3	1	1.067	1.012	0.968	0.936	1.041	0.972	0.968	4.77E-01
cds.Camellia_sinensis.comp24910_c0_seq1_m.13294	PREDICTED: endo-1,3;1,4-beta-D-glucanase [Vitis vinifera]	28.153	86.932	35.6	10	32	10	1.005	0.988	0.989	0.977	0.997	1.005	0.999	9.22E-01
cds.Camellia_sinensis.comp24910_c0_seq1_m.13295	PREDICTED: probable chalcone--flavonone isomerase 3 [Prunus mume]	23.229	80.777	23.4	5	10	5	1.326	1.256	1.273	0.771	0.733	0.635	0.555	6.03E-04

cds.Camellia_sinensis.comp24911_c0_seq1_m.38588	PREDICTED: 40S ribosomal protein S11-like [Daucus carota subsp. sativus]	17.858	12.377	35.2	7	8	7	1.2	1.215	1.197	0.766	0.767	0.774	0.639	7.59E-08
cds.Camellia_sinensis.comp24917_c0_seq1_m.41745	PREDICTED: ATP synthase subunit b', chloroplastic [Theobroma cacao]	24.011	64.13	36.2	8	42	8	1.003	0.995	0.976	1.006	1.005	1.046	1.028	1.51E-01
cds.Camellia_sinensis.comp24926_c0_seq1_m.49132	Translation machinery associated TMA7 [Arabidopsis thaliana]	11.151	2.0296	11.8	1	2	1	0.988	1.052	1.043	0.986	1.04	0.899	0.949	3.13E-01
cds.Camellia_sinensis.comp24932_c0_seq1_m.38826	PREDICTED: 17.3 kDa class II heat shock protein-like [Nicotiana attenuata]	18.083	62.033	48.8	5	15	5	1.028	1.046	0.97	0.96	1.016	0.983	0.972	3.75E-01

cds.Camellia_sinensis.comp24934_c0_seq1_m.49073	photosystem II 5 kDa protein, chloroplastic-like [Cajanus cajan]	11.748	15.377	11.5	1	9	1	0.985	1.05	0.961	0.989	1.035	0.987	1.005	8.67E-01
cds.Camellia_sinensis.comp24944_c0_seq1_m.7395	PREDICTED: heat shock 70 kDa protein 16 [Nicotiana tomentosiformis]	85.424	3.8599	6	6	9	4	0.973	0.953	0.982	1.068	1.018	0.989	1.057	8.30E-02
cds.Camellia_sinensis.comp24950_c0_seq1_m.34056	PREDICTED: chlorophyll a-b binding protein 4, chloroplastic [Vitis vinifera]	28.417	38.296	25	5	17	5	0.892	0.848	0.915	1.095	1.127	1.18	1.281	1.36E-03
cds.Camellia_sinensis.comp24956_c0_seq1_m.23294	PREDICTED: peroxidase 42 [Eucalyptus grandis]	37.589	27.808	24	6	6	6	1.202	1.251	1.235	0.749	0.773	0.725	0.609	2.14E-05

cds.Camellia_sinensis.comp24958_c0_seq1_m.31985	PREDICTED: ribulose bisphosphate carboxylase small chain, chloroplastic isoform X1 [Juglans regia]	20.359	132.75	61.5	14	95	10	1.042	1.151	1.169	0.88	0.876	0.857	0.777	2.46E-03
cds.Camellia_sinensis.comp24983_c0_seq1_m.16904	BTB/POZ domain-containing protein [Morus notabilis]	63.368	6.9201	5.5	3	3	2	0.772	0.994	1.05	1.161	1.159	0.923	1.152	3.01E-01
cds.Camellia_sinensis.comp24992_c0_seq1_m.29544	PREDICTED: probable aquaporin PIP-type 7a [Gossypium raimondii]	31.672	3.8657	8.8	3	3	3	1.019	0.969	1.049	0.996	1.026	0.97	0.985	6.32E-01
cds.Camellia_sinensis.comp25004_c0_seq2_m.48898	PREDICTED: histone H4-like [Zea mays]	11.409	80.118	58.3	7	40	7	1.105	1.078	1.089	0.875	0.898	0.858	0.804	1.35E-04
cds.Camellia_sinensis.comp25017_c0_seq1_m.47244	PREDICTED: 40S ribosomal protein S27-2-like [Nelumbo nucifera]	14.794	5.6452	15.9	2	6	2	0.991	0.951	1.024	0.997	1.017	0.988	1.012	6.18E-01

cds.Camellia_sinensis.comp25019_c0_seq1_m.18730	PREDICTED: beta-glucosidase 12 [Vitis vinifera]	57.105	33.283	17.6	10	17	9	1.054	0.987	0.993	0.957	1.019	0.964	0.969	3.37E-01
cds.Camellia_sinensis.comp25031_c0_seq2_m.30005	selenocysteine methyltransferase family protein [Populus trichocarpa]	37.444	4.5281	10.2	4	4	4	0.958	0.951	0.993	1.027	1.06	1.033	1.075	1.18E-02
cds.Camellia_sinensis.comp25034_c0_seq2_m.32478	PREDICTED: L-ascorbate peroxidase 1, cytosolic isoform X2 [Phoenix dactylifera]	27.549	64.454	41.6	9	46	9	1.003	1.001	1.026	0.982	1.005	1.002	0.986	2.74E-01
cds.Camellia_sinensis.comp25035_c0_seq1_m.34370	PREDICTED: photosystem II 22 kDa protein, chloroplastic [Vitis vinifera]	28.292	96.184	29.2	7	25	5	1.031	1.045	1.029	0.963	0.976	0.962	0.934	5.39E-04

cds.Camellia_sinensis.comp25036_c0_seq1_m.20734	PREDICTED: S-adenosylmethionine synthase 2 [Eucalyptus grandis]	43.081	157.68	42.7	15	44	7	1.149	1.106	1.14	0.849	0.875	0.863	0.762	4.41E-05
cds.Camellia_sinensis.comp25037_c0_seq1_m.30194	PREDICTED: flavonol synthase/flavanone 3-hydroxylase-like [Prunus mume]	37.581	105.57	29.6	10	30	10	1.37	1.357	1.379	0.599	0.605	0.604	0.44	6.50E-09
cds.Camellia_sinensis.comp25050_c0_seq1_m.47802	PREDICTED: histone H2A.1-like [Prunus mume]	19.906	2.7071	13.4	4	8	2	1.226	1.072	1.062	0.776	0.808	1.004	0.77	4.54E-02
cds.Camellia_sinensis.comp25052_c0_seq1_m.4227	PREDICTED: 1,4-alpha-glucan-branching enzyme 3, chloroplastic/amyloplastic isoform X2 [Ziziphus jujuba]	88.202	2.5393	1.8	1	1	1								

cds.Camellia_sinensis.comp25080_c0_seq1_m.33148	PREDICTED: signal peptidase complex catalytic subunit SEC11A-like [Glycine max]	22.599	6.3722	5.4	1	1	1	0.948	0.986	1.004	1.077	0.993	1.014	1.05	1.80E-01
cds.Camellia_sinensis.comp25087_c0_seq1_m.41266	PREDICTED: 60S ribosomal protein L22-2-like [Ziziphus jujuba]	13.995	24.424	25.8	3	4	3	1.024	1.122	1.104	0.916	0.871	0.879	0.82	3.56E-03
cds.Camellia_sinensis.comp25092_c0_seq2_m.43164	PREDICTED: 60S ribosomal protein L36-2 [Eucalyptus grandis]	12.443	2.8655	9.1	1	2	1	1.009	1.037	1.068	0.909	0.962	1.011	0.925	8.84E-02
cds.Camellia_sinensis.comp25104_c0_seq1_m.37456	tubulin alpha-6 chain, putative [Medicago truncatula]	24.424	4.2641	8.8	2	2	2	1.055	1.028	0.979	0.919	0.961	1.052	0.958	3.83E-01

cds.Camellia_sinensis.comp25121_c0_seq21_m.47958	PREDICTED: cell division topological specificity factor homolog, chloroplastic-like [Sesamum indicum]	18.014	13.464	16.9	2	3	2	0.982	1.013	1.031	0.955	0.992	1.034	0.985	6.01E-01
cds.Camellia_sinensis.comp25125_c0_seq15_c0_seq1_m.14406	PREDICTED: lycopene beta cyclase, chloroplastic [Vitis vinifera]	56.64	4.5273	4.2	2	3	2	1.164	1.126	1.032	0.902	0.859	0.884	0.796	4.18E-03
cds.Camellia_sinensis.comp25139_c0_seq1_m.10006	PREDICTED: protein transport protein SEC23 [Citrus sinensis]	36.04	5.3776	10.8	2	3	1	0.9	1.183	0.954	0.93	1.018	1.026	0.979	8.75E-01
cds.Camellia_sinensis.comp25156_c0_seq1_m.24729	transmembrane protein, putative [Medicago truncatula]	32.997	3.4805	7.1	2	2	2								

cds.Camellia_sinensis.comp25164_c0_seq1_m.15760	PREDICTED: beta-hexosaminidase 1 isoform X2 [Ziziphus jujuba]	25.448	14.385	24.4	5	7	4	1.035	1.045	1.006	0.996	0.979	0.946	0.947	4.40E-02
cds.Camellia_sinensis.comp25164_c0_seq2_m.15762	PREDICTED: beta-hexosaminidase 1 [Vitis vinifera]	36.014	28.244	17.4	5	10	5	1.039	1.006	1.053	0.932	0.954	0.965	0.92	8.12E-03
cds.Camellia_sinensis.comp25169_c0_seq1_m.2215	PREDICTED: 5-oxoprolinase [Vitis vinifera]	138.23	69.564	12.9	14	19	14	0.92	0.985	0.978	1.042	1.017	1.056	1.08	3.29E-02
cds.Camellia_sinensis.comp25190_c0_seq1_m.35088	PREDICTED: 40S ribosomal protein S6-like [Cucumis sativus]	28.255	52.56	31.7	7	12	4	1.158	1.139	1.154	0.799	0.829	0.86	0.721	1.17E-04
cds.Camellia_sinensis.comp25196_c0_seq1_m.48078	PREDICTED: 14 kDa proline-rich protein DC2.15-like [Erythranthe guttata]	17.32	1.5248	5.5	1	1	1								

cds.Camellia_sinensis.comp25199_c0_seq1_m.17068	PREDICTED: ATP-dependent 6-phosphofructokinase 4, chloroplastic [Eucalyptus grandis]	62.915	12.251	14.4	8	12	5	1.016	1.028	1.051	0.943	1.026	0.952	0.944	1.09E-01
cds.Camellia_sinensis.comp25200_c0_seq2_m.44828	PREDICTED: 60S ribosomal protein L44-like [Gossypium hirsutum]	12.015	2.2006	14.3	2	2	2	1.061	1.143	1.055	0.891	0.862	0.967	0.835	1.33E-02
cds.Camellia_sinensis.comp25206_c0_seq1_m.25459	PREDICTED: cellulose synthase-like protein E6 [Vitis vinifera]	16.051	1.3796	6.3	1	1	1	1.023	0.991	0.968	0.998	1.026	1.001	1.014	4.72E-01
cds.Camellia_sinensis.comp25217_c0_seq2_m.33462	PREDICTED: F-box only protein 21 isoform X2 [Nicotiana tomentosiformis]	24.903	54.619	22.2	4	8	4	1.052	1.01	1.025	0.974	0.953	0.989	0.945	2.38E-02

cds.Camellia_sinensis.comp25221_c0_seq1_m.44708	PREDICTED: protein mago nashi homolog [Theobrom	20.474	23.169	36.8	5	9	5	0.966	1.009	0.975	1.059	0.984	0.979	1.024	4.55E-01
cds.Camellia_sinensis.comp25223_c0_seq1_m.19626	PREDICTED: patatin-like protein 2 [Vitis vinifera]	57.26	41.313	21.2	10	15	10	0.974	0.987	1.01	1.003	0.993	1.023	1.016	3.07E-01
cds.Camellia_sinensis.comp25228_c1_seq6_m.28157	transmembrane protein [Arabidopsis thaliana]	40.642	43.251	21.7	8	11	8	1.043	1.066	1.022	0.932	0.925	0.973	0.904	7.08E-03
cds.Camellia_sinensis.comp25234_c0_seq1_m.45771	PREDICTED: EG45-like domain containing protein [Prunus mume]	13.442	132.07	50	5	17	5	1.009	1.02	0.97	0.989	0.999	0.996	0.995	7.74E-01
cds.Camellia_sinensis.comp25240_c0_seq1_m.1075	NADH-plastoquinone oxidoreductase subunit 5 (chloroplast) [Camellia leptophylla]	85.037	26.413	1.5	1	2	1	0.996	0.917	1.015	1.034	1.034	1.022	1.055	2.23E-01

cds.Camellia_sinensis.comp25250_c0_seq1_m.27012	PREDICTED: stress-induced-phosphoprotein 1 [Gossypium hirsutum]	13.865	1.5886	7.9	1	1	1								
cds.Camellia_sinensis.comp25270_c0_seq1_m.513	PREDICTED: dnaJ homolog subfamily C GRV2 isoform X2 [Prunus	244.54	25.411	4.1	7	7	7	0.93	1	1.019	1.013	0.963	1.022	1.017	6.40E-01
cds.Camellia_sinensis.comp25271_c1_seq1_m.35482	PREDICTED: heterodimeric geranylgeranyl pyrophosphate synthase small subunit, chloroplastic-like [Populus euphratica]	37.437	35.722	22.8	8	11	8	1.037	1.058	1.076	0.93	0.947	0.952	0.892	8.79E-04
cds.Camellia_sinensis.comp25276_c0_seq1_m.33734	PREDICTED: dihydroflavonol 4-reductase-like [Vitis vinifera]	38.478	14.842	9.9	3	4	3	0.931	1.011	0.957	1.063	1.056	0.982	1.07	1.28E-01

cds.Camellia_sinensis.comp25284_c0_seq1_m.47591	Dihydrolipoyl dehydrogenase [Morus notabilis]	12.205	6.3063	36	3	5	2	1.032	0.959	0.993	1.055	0.902	1.067	1.013	8.61E-01
cds.Camellia_sinensis.comp25296_c0_seq1_m.51429	PREDICTED: probable non-specific lipid-transfer protein AKCS9 [Fragaria vesca subsp. vesca]	13.126	3.9386	19.2	2	2	2	0.925	0.955	0.92	1.14	1.022	1.073	1.155	1.30E-02
cds.Camellia_sinensis.comp25299_c0_seq1_m.39018	50S ribosomal protein L34 [Arabidopsis thaliana]	22.839	15.99	19.6	4	5	4	1.016	0.969	1.018	1.007	1.001	0.98	0.995	8.03E-01
cds.Camellia_sinensis.comp25300_c0_seq1_m.38412	PREDICTED: 50S ribosomal protein L29, chloroplastic [Gossypium raimondii]	24.866	26.5	19	4	5	4	1.097	1.071	1.093	0.97	0.889	0.892	0.844	4.40E-03

cds.Camellia_sinensis.comp25311_c0_seq1_m.7542	PREDICTED: calnexin homolog 1-like [Nicotiana tabacum]	62.823	92.933	33.2	15	21	15	1.029	0.988	1.01	1.008	0.995	0.971	0.982	3.34E-01
cds.Camellia_sinensis.comp25312_c0_seq3_m.20634	PREDICTED: 5'-adenylylsulfate reductase 3, chloroplastic-like [Nicotiana tabacum]	52.173	23.034	12	5	6	5	0.914	0.944	0.974	1.036	1.119	1.103	1.15	9.50E-03
cds.Camellia_sinensis.comp25316_c0_seq1_m.8737	PREDICTED: translation initiation factor IF-2, chloroplastic-like [Nelumbo nucifera]	81.562	49.172	14.6	10	12	10	1.103	1.11	1.107	0.897	0.895	0.862	0.799	3.38E-03
cds.Camellia_sinensis.comp25317_c0_seq1_m.7453	PREDICTED: magnesium-chelatase subunit ChlD, chloroplastic isoform X1 [Nelumbo	77.766	15.358	12	8	9	8	1.088	1.042	1.085	0.986	0.836	0.861	0.835	2.54E-02

cds.Camellia_sinensis.comp25344_c0_seq1_m.53989		9.9658	12.967	18.3	1	1	1	0.886	0.734	0.986	1.152	1.15	1.149	1.324	7.91E-02
cds.Camellia_sinensis.comp25349_c0_seq1_m.49225	PREDICTED: thioredoxin-like protein CXXS1 [Theobroma cacao]	16.177	4.8822	7	1	2	1	0.989	0.865	0.886	1.091	1.135	1.067	1.202	1.47E-02
cds.Camellia_sinensis.comp25361_c0_seq1_m.33769	Heavy metal transport/detoxification superfamily protein [Arabidopsis thaliana]	22.167	50.225	18	2	4	2	1.052	1.063	0.973	0.918	0.952	0.989	0.926	9.50E-02
cds.Camellia_sinensis.comp25366_c0_seq1_m.40729	ATP-dependent Clp protease ATP-binding subunit [Arabidopsis thaliana]	27.259	15.557	6.4	1	1	1	1.007	1.004	1.074	0.928	1.068	0.923	0.946	3.38E-01
cds.Camellia_sinensis.comp25367_c0_seq1_m.46441	calcium-dependent kinase [Medicago truncatula]	11.617	5.8756	25.5	2	2	2	1.02	0.959	0.941	1.147	0.918	1.033	1.061	4.64E-01

cds.Camellia_sinensis.comp25370_c0_seq1_m.26624	PREDICTED: translocon-associated protein subunit beta [Vitis vinifera]	21.146	46.895	20.5	2	5	2	0.937	1.004	1.016	1.038	1.043	1.038	1.055	1.66E-01
cds.Camellia_sinensis.comp25383_c0_seq1_m.46956	polyketide cyclase/dehydrase and lipid transporter [Medicago truncatula]	16.041	2.5326	11.3	1	1	1								
cds.Camellia_sinensis.comp25396_c0_seq1_m.35631	PREDICTED: premnaspirodiene oxygenase-like [Vitis vinifera]	38.624	3.331	3.5	1	1	1	1.074	0.895	1.051	1.027	0.928	1.027	0.987	8.82E-01
cds.Camellia_sinensis.comp25398_c0_seq1_m.42073	PREDICTED: eukaryotic translation initiation factor 3 subunit G [Vitis vinifera]	26.215	101.71	41.2	9	12	7	1.036	0.996	1.066	0.988	0.982	0.98	0.952	1.34E-01
cds.Camellia_sinensis.comp25412_c0_seq1_m.11339	PREDICTED: 26S proteasome non-ATPase regulatory subunit 6 [Ziziphus jujuba]	44.12	58.21	21.4	7	15	7	0.958	0.954	0.954	1.039	1.032	1.044	1.087	2.07E-05

cds.Camellia_sinensis.comp25412_c0_seq1_m.11340	PREDICTED: succinate dehydrogenase [ubiquinone] iron-sulfur subunit 2, mitochondrial-like [Nelumbo nucifera]	31.441	81.003	29.4	7	16	4	1.047	0.99	0.956	0.998	0.983	1.027	1.005	8.60E-01
cds.Camellia_sinensis.comp25412_c0_seq2_m.34327	PREDICTED: succinate dehydrogenase [ubiquinone] iron-sulfur subunit 2, mitochondrial-like [Nelumbo nucifera]	34.504	11.8	20.3	6	10	3	0.97	1.073	1.028	0.988	0.945	0.926	0.931	1.12E-01
cds.Camellia_sinensis.comp25416_c0_seq1_m.20324	PREDICTED: eukaryotic translation initiation factor 3 subunit J-like [Juglans regia]	25.642	4.8247	6.6	1	1	1	0.997	1.114	1.076	0.821	0.888	1.087	0.877	2.02E-01

cds.Camellia_sinensis.comp25419_c0_seq1_m.11908	PREDICTED: 3-oxoacyl-[acyl-carrier-protein] synthase II, chloroplastic [Vitis	61.051	31.903	10.6	5	6	5	0.963	1	0.997	1.072	0.988	0.996	1.032	3.35E-01
cds.Camellia_sinensis.comp25420_c0_seq1_m.34045	PREDICTED: protein BOBBER 1 [Sesamum indicum]	33.459	43.941	37.2	13	21	13	1.078	1.075	1.083	0.912	0.91	0.936	0.852	6.36E-05
cds.Camellia_sinensis.comp25432_c0_seq1_m.14970	PREDICTED: beta-glucosidase BoGH3B-like [Malus domestica]	71.667	77.376	23	11	27	10	0.925	0.915	0.921	1.104	1.087	1.075	1.183	3.80E-05
cds.Camellia_sinensis.comp25440_c0_seq1_m.32595	PREDICTED: 2-hydroxyisoflavanone dehydratase-like [Vitis vinifera]	37.254	15.621	2.9	1	1	1	0.942	0.961	0.979	1.038	1.045	1.058	1.09	2.28E-03
cds.Camellia_sinensis.comp25442_c0_seq1_m.40798	PREDICTED: 40S ribosomal protein S15 [Ricinus communis]	17.202	10.646	19.2	2	5	1	1.215	1.125	1.264	0.756	0.871	0.713	0.649	3.18E-03

cds.Camellia_sinensis.comp25442_c0_seq2_m.41805	PREDICTED: 40S ribosomal protein S15 [Ricinus communis]	17.174	-2	19.2	2	3	1								
cds.Camellia_sinensis.comp25445_c0_seq1_m.13251	PREDICTED: plastidic glucose transporter 4-like [Juglans regia]	57.807	2.5803	2.9	2	2	2	0.967	1.009	0.97	1.012	1.055	1.002	1.042	1.22E-01
cds.Camellia_sinensis.comp25446_c0_seq1_m.13292	SLH domain protein [Arabidopsis thaliana]	27.662	8.3149	8.4	2	2	2	1.037	1.05	0.951	0.995	0.93	1.036	0.975	5.93E-01
cds.Camellia_sinensis.comp25446_c0_seq1_m.13293	SLH domain protein [Arabidopsis thaliana]	25.983	13.56	11.1	2	2	2	1.003	1.049	1.092	1.029	0.961	0.872	0.91	1.51E-01
cds.Camellia_sinensis.comp25448_c0_seq1_m.6626	plant/MEB5-like protein [Medicago truncatula]	94.205	4.9459	3.1	2	2	2	1.07	0.971	1.094	1.025	0.793	1.04	0.912	3.59E-01

cds.Camellia_sinensis.comp25454_c0_seq1_m.28314	PREDICTED: naringenin,2-oxoglutarate 3-dioxygenase [Ziziphus	44.491	178.49	49.4	18	49	18	1.386	1.375	1.399	0.556	0.589	0.561	0.41	5.66E-07
cds.Camellia_sinensis.comp25455_c0_seq1_m.29329	FAD-dependent oxidoreductase family protein [Populus trichocarpa]	40.614	5.0158	5.7	2	2	2								
cds.Camellia_sinensis.comp25470_c0_seq1_m.21842	PREDICTED: outer envelope pore protein 37, chloroplastic [Solanum pennellii]	41.219	26.377	20.6	6	9	6	0.99	0.998	0.968	1.012	1.013	1	1.023	8.23E-02
cds.Camellia_sinensis.comp25474_c0_seq1_m.11244	PREDICTED: probable tRNA (guanine(26)-N(2))-dimethyltransferase 2 isoform X1 [Vitis vinifera]	66.23	1.6165	1.5	1	1	1	1.025	0.983	1.169	0.928	0.929	0.96	0.887	9.23E-02

cds.Camellia_sinensis.comp25476_c0_seq1_m.42537	alba DNA/RNA-binding protein [Medicago truncatula]	14.425	26.296	30.2	5	8	1	0.923	0.943	0.939	1.074	1.173	0.985	1.152	1.12E-01
cds.Camellia_sinensis.comp25488_c0_seq1_m.16338	PREDICTED: protein disulfide isomerase-like 1-4 [Populus euphratica]	64.24	102.49	28.5	15	20	15	1.115	1.113	1.082	0.912	0.881	0.883	0.808	1.38E-04
cds.Camellia_sinensis.comp25494_c0_seq1_m.22116	PREDICTED: casein kinase II subunit alpha-like isoform X1 [Nicotiana tabacum]	51.354	12.596	12.9	5	6	5	0.974	1.02	0.989	1.077	0.98	0.99	1.021	5.66E-01
cds.Camellia_sinensis.comp25497_c0_seq1_m.43426	PREDICTED: NADH dehydrogenase [ubiquinone] 1 beta subcomplex subunit 9 [Eucalyptus grandis]	13.387	38.348	45.2	6	13	6	1.017	1.025	1.098	0.966	0.967	0.941	0.915	2.74E-02
cds.Camellia_sinensis.comp25498_c0_seq1_m.39612	transmembrane protein, putative [Medicago truncatula]	26.761	3.9404	12.4	3	3	3	0.985	0.987	0.984	0.992	1.01	1.008	1.018	8.84E-02

cds.Camellia_sinensis.comp25502_c0_seq1_m.37641	abscisic stress-ripening protein 2-like [Citrus sinensis]	23.762	59.028	34.3	5	17	5	1.231	1.097	1.134	0.802	0.836	0.855	0.72	1.10E-03
cds.Camellia_sinensis.comp25510_c0_seq3_m.18506	PREDICTED: ribulose-1,5 bisphosphate carboxylase/oxygenase large subunit N-methyltransferase, chloroplastic[Citrus sinensis]	35.027	1.3106	3.9	1	1	1								
cds.Camellia_sinensis.comp25510_c0_seq3_m.18507	PREDICTED: ribulose-1,5 bisphosphate carboxylase/oxygenase large subunit N-methyltransferase, chloroplastic-like isoform X4 [Theobroma cacao]	26.164	2.7583	5.3	1	1	1	0.968	1.121	1.047	0.948	1.016	0.903	0.914	1.77E-01

cds.Camellia_sinensis.comp25522_c0_seq1_m.12388	PREDICTED: dihydrolipoyllysine-residue acetyltransferase component 2 of pyruvate dehydrogenase complex,mitochondrial isoform X1 [Sesamum indicum]	37.435	184.02	41.9	12	24	12	1.021	0.986	0.983	1.015	1.002	1.005	1.011	4.46E-01
cds.Camellia_sinensis.comp25522_c0_seq1_m.12389	PREDICTED: dihydrolipoyllysine-residue acetyltransferase component 2 of pyruvate dehydrogenase complex,mitochondrial-like [Juglans regia]	30.446	49.675	26.2	7	13	7	1.014	0.978	0.976	0.997	1	1.035	1.022	2.84E-01
cds.Camellia_sinensis.comp25540_c0_seq1_m.38260		23.186	19.294	20.2	4	6	4	1.049	1.043	1.026	0.948	0.94	0.959	0.913	5.00E-04

cds.Camellia_sinensis.comp25544_c0_seq1_m.33642	PREDICTED: UPF0301 protein Cpha266_0885 [Citrus sinensis]	31.92	1.1885	9.2	2	2	2	1.12	1.31	1.04	0.819	0.752	0.912	0.716	1.93E-02
cds.Camellia_sinensis.comp25549_c0_seq1_m.3438	PREDICTED: hexokinase-1 isoform X1 [Vitis vinifera]	59.434	103.99	33.8	14	27	14	0.933	0.955	1.021	1.049	1.029	1.034	1.07	6.91E-02
cds.Camellia_sinensis.comp25551_c0_seq2_m.25546	transglutaminase family protein [Medicago truncatula]	25.884	2.0705	4.1	1	1	1	0.878	0.961	1.008	0.98	1.162	1.043	1.119	1.58E-01
cds.Camellia_sinensis.comp25552_c0_seq1_m.33134	PREDICTED: probable carboxylesterase 12 [Juglans regia]	37.866	16.149	13.9	4	5	4	0.985	1.084	1.088	0.901	0.959	0.954	0.891	4.02E-02
cds.Camellia_sinensis.comp25559_c0_seq1_m.36815	PREDICTED: outer envelope pore protein 16-3, chloroplastic/mitochondrial [Ricinus communis]	24.653	4.1982	12.4	3	5	3	0.988	1.024	0.97	1.02	1.028	0.935	1	9.93E-01

cds.Camellia_sinensis.comp25562_c0_seq2_m.49216	PREDICTED: 1-aminocyclopropane-1-carboxylate oxidase [Gossypium hirsutum]	11.278	1.4641	8.9	1	1	1	1.056	1.22	1.184	0.926	0.742	0.843	0.726	1.43E-02
cds.Camellia_sinensis.comp25568_c0_seq1_m.32625	transmembrane protein [Arabidopsis thaliana]	33.457	27.254	15.3	4	8	4	1.039	1.034	1.033	0.936	0.976	0.967	0.927	2.72E-02
cds.Camellia_sinensis.comp25586_c0_seq1_m.20566	PREDICTED: aminoacylase-1 isoform X2 [Vitis vinifera]	49.15	88.904	33.2	12	19	12	0.948	0.984	0.955	1.053	1.052	1.048	1.092	1.65E-02
cds.Camellia_sinensis.comp25603_c0_seq1_m.23574	uncharacterized LOC107793530 [Nicotiana tabacum]	44.036	20.097	14.8	5	5	5	1.017	1.003	1.033	0.93	0.991	1.025	0.965	2.88E-01
cds.Camellia_sinensis.comp25606_c0_seq1_m.24811	PREDICTED: signal recognition particle 43 kDa protein, chloroplastic [Solanum tuberosum	47.758	49.651	28.7	14	17	14	0.992	0.973	1.004	1.015	1.019	0.988	1.018	2.55E-01

cds.Camellia_sinensis.comp25607_c0_seq1_m.14266	PREDICTED: adenosylhomocysteinase [Ziziphus jujuba]	53.495	190.33	59	25	75	20	1.048	1.055	1.05	0.937	0.957	0.943	0.9	8.35E-05
cds.Camellia_sinensis.comp25618_c0_seq1_m.43246	PREDICTED: polyadenylate-binding protein RBP45B-like isoform X3 [Solanum lycopersic	23.911	6.1499	5.3	1	2	1								
cds.Camellia_sinensis.comp25621_c0_seq1_m.42677	PREDICTED: protein transport protein Sec61 subunit beta [Cucumis sativus]	11.735	1.5189	9.9	1	1	1	1.016	1.01	0.995	1.034	0.927	1.023	0.988	7.22E-01
cds.Camellia_sinensis.comp25622_c0_seq1_m.8997	PREDICTED: DNA repair helicase UVH6 isoform X2 [Sesamum indicum]	31.119	1.927	3.3	1	1	1								

cds.Camellia_sinensis.comp25623_c0_seq1_m.20018	PREDICTED: rhomboid-like protein 20 [Vitis vinifera]	32.669	2.6345	6.5	2	2	2								
cds.Camellia_sinensis.comp25629_c0_seq1_m.41703	PREDICTED: probable NADH dehydrogenase [ubiquinone] 1 alpha subcomplex subunit 5, mitochondrial [Sesamum indicum]	21.912	60.521	61.1	10	16	10	1.034	1.041	0.991	0.955	0.956	0.976	0.942	2.41E-02
cds.Camellia_sinensis.comp25645_c0_seq1_m.23361	PREDICTED: delta-aminolevulinic acid dehydratase, chloroplastic [Populus euphratica]	46.982	69.501	27.9	10	20	10	1.028	1.081	1.065	0.913	0.929	0.964	0.884	4.90E-03

cds.Camellia_sinensis.comp25651_c0_seq1_m.34569	PREDICTED: 50S ribosomal protein L13, chloroplastic-like [Capsicum annuum]	22.568	11.41	17.6	3	4	3	1.036	1.024	1.038	0.982	0.99	0.955	0.945	8.26E-03		
cds.Camellia_sinensis.comp25652_c0_seq1_m.11281	PREDICTED: paramyosin [Erythranthe guttata]	17.273	1.9099	7.3	1	1	1										
cds.Camellia_sinensis.comp25652_c0_seq2_m.20881	PREDICTED: paramyosin isoform X2 [Vitis vinifera]	43.386	2.4085	6.5	3	3	3										
cds.Camellia_sinensis.comp25658_c0_seq1_m.38497	Cu/Zn superoxide dismutase family protein [Populus trichocarpa]	15.167	67.447	40.1	5	20	4	0.932	0.931	0.941	1.055	1.095	1.091	1.156	3.00E-04		
cds.Camellia_sinensis.comp25663_c0_seq1_m.7758	PREDICTED: protein SMAX1-LIKE 4 [Vitis vinifera]	106.65	1.6125	1.3	1	1	1	1.085	1.001	1.118	1.062	0.893	0.837	0.871	1.45E-01		

cds.Camellia_sinensis.comp25664_c0_seq2_m.46175	PREDICTED: protein disulfide isomerase-like 5-1 [Vitis vinifera]	16.911	2.3361	12.8	2	3	1	1.003	1.062	0.978	0.979	1.006	0.976	0.973	3.64E-01
cds.Camellia_sinensis.comp25664_c0_seq3_m.47339	PREDICTED: protein disulfide isomerase-like 5-1 [Vitis vinifera]	16.908	8.5579	15.4	3	5	2	1.038	1.079	1.023	0.977	0.832	1.043	0.908	2.19E-01
cds.Camellia_sinensis.comp25680_c0_seq1_m.17593	PSI reaction center subunit II [Citrus sinensis]	25.308	137.71	48.7	11	83	11	1.017	1.033	1.01	0.986	0.997	0.976	0.967	2.08E-02
cds.Camellia_sinensis.comp25691_c0_seq2_m.8971	PREDICTED: 5'-nucleotidase domain-containing protein 4-like isoform X2 [Juglans regia]	67.005	98.762	20	10	18	10	0.938	0.99	0.964	1.03	1.032	1.029	1.069	5.05E-02
cds.Camellia_sinensis.comp25692_c0_seq2_m.33412	PREDICTED: urease accessory protein G [Ziziphus jujuba]	32.046	71.654	30.6	7	14	7	1.038	1.046	1.066	0.919	0.966	0.947	0.899	2.96E-03

cds.Camellia_sinensis.comp25696_c0_seq1_m.13773	PREDICTED: threonine dehydratase biosynthetic, chloroplastic [Nelumbo nucifera]	66.833	10.193	8	5	6	5	1.021	1.025	1.01	1.019	0.989	0.973	0.975	1.54E-01
cds.Camellia_sinensis.comp25697_c0_seq1_m.5308	PREDICTED: lon protease homolog, mitochondrial-like [Nelumbo nucifera]	109.28	41.408	16.5	12	15	12	1	0.961	0.951	0.994	1.027	1.003	1.038	1.05E-01
cds.Camellia_sinensis.comp25700_c0_seq1_m.51328	PREDICTED: 40S ribosomal protein S6 [Musa acuminata subsp. malaccensis]	20.093	5.7845	13.1	2	2	1								
cds.Camellia_sinensis.comp25711_c0_seq1_m.4368	PREDICTED: lethal(2) giant larvae protein homolog SRO77-like isoform X1 [Prunus	121.56	8.1733	3.5	4	5	4	1.031	0.901	1.035	0.984	1.046	1.012	1.025	6.14E-01

cds.Camellia_sinensis.comp25717_c0_seq2_m.450	PREDICTED: 30S ribosomal protein S20, chloroplastic-like [Nicotiana attenuata]	20.293	4.4965	4.9	1	1	1	1.08	1.091	1.205	0.873	0.901	0.825	0.77	3.86E-03
cds.Camellia_sinensis.comp25720_c0_seq2_m.26153	actin cross-linking protein [Medicago truncatula]	33.561	1.3064	3	1	1	1	1.034	1.197	1.049	0.894	0.838	0.968	0.823	3.60E-02
cds.Camellia_sinensis.comp25723_c0_seq1_m.34156	PREDICTED: fatty-acid-binding protein 1 [Jatropha curcas]	33.225	59.404	25.7	8	11	8	0.995	0.991	0.993	1.038	1.011	1.001	1.024	1.64E-01
cds.Camellia_sinensis.comp25724_c0_seq1_m.19500	nucleoporin protein Ndc1-Nup [Medicago truncatula]	62.648	1.4679	3.4	1	1	1								
cds.Camellia_sinensis.comp25726_c0_seq1_m.23728	PREDICTED: prostaglandin E synthase 2 [Jatropha curcas]	27.428	5.8107	10.5	4	5	3	0.998	0.974	1.023	0.984	1.049	0.944	0.994	8.51E-01

cds.Camellia_sinensis.comp25728_c0_seq2_m.49862	PREDICTED: thioredoxin-1 isoform X1 [Juglans regia]	15.412	13.626	7.7	1	7	1	1.009	0.975	0.993	1.025	0.985	1.008	1.014	4.20E-01
cds.Camellia_sinensis.comp25744_c0_seq1_m.47627	PREDICTED: dolichyl-diphosphooligosaccharide--protein glycosyltransferase subunit DAD1 [Nicotiana tabacum]	12.779	4.9561	8.6	1	1	1	1.056	0.884	0.906	1.048	1.02	1.101	1.113	1.40E-01
cds.Camellia_sinensis.comp25745_c0_seq1_m.29044	PREDICTED: mitogen-activated protein kinase kinase 5-like [Capsicum annuum]	39.991	9.3595	7.2	2	4	2	0.914	0.979	0.914	1.217	1.032	1.043	1.173	5.37E-02
cds.Camellia_sinensis.comp25747_c1_seq1_m.2467	PREDICTED: splicing factor 3B subunit 3 [Vitis vinifera]	135	53.97	15.3	18	22	18	0.975	0.975	0.985	1.054	1.026	1.022	1.057	5.76E-03

cds.Camellia_sinensis.comp25759_c0_seq1_m.36958	PAP fibrillin domain protein,expressed protein [Medicago truncatula]	29.924	62.065	23.3	6	8	6	1.017	0.996	1.006	1.023	1.032	0.987	1.008	6.44E-01
cds.Camellia_sinensis.comp25763_c0_seq1_m.4976	PREDICTED: DExH-box ATP-dependent RNA helicase DExH10 [Nelumbo nucifera]	114.92	1.6781	2.5	3	3	3	0.969	0.968	1.026	0.941	1.11	1	1.03	6.27E-01
cds.Camellia_sinensis.comp25769_c0_seq1_m.24383	PREDICTED: peroxisomal adenine nucleotide carrier 1 [Citrus sinensis]	36.089	2.25	4	1	1	1	0.843	0.986	1.124	0.885	0.608	1.557	1.033	9.05E-01
cds.Camellia_sinensis.comp25800_c0_seq1_m.33226	PREDICTED: peroxidase 12 [Fragaria vesca subsp. vesca]	39.358	117.25	30.7	12	28	12	0.978	1.031	1.027	1.019	0.99	1.009	0.994	7.78E-01

cds.Camellia_sinensis.comp25801_c0_seq3_m.46501	PREDICTED: probable plastid-lipid-associated protein 11 isoform X3 [Juglans regia]	22.254	19.049	34.7	7	8	7	0.946	0.991	1.021	1.113	0.971	0.982	1.037	5.22E-01
cds.Camellia_sinensis.comp25806_c0_seq1_m.33587	PREDICTED: NAD(P)H-quinone oxidoreductase subunit M, chloroplastic-like [Gossypium hirsutum]	25.57	45.372	39.7	10	12	10	1.013	1.009	0.959	1.002	1.029	1.005	1.018	3.97E-01
cds.Camellia_sinensis.comp25808_c0_seq1_m.685	PREDICTED: protein TSS [Vitis vinifera]	181.39	34.367	6.1	10	10	8	1.096	1.067	1.138	0.942	0.839	0.864	0.801	4.88E-03
cds.Camellia_sinensis.comp25808_c0_seq1_m.686	PREDICTED: protein TSS [Ricinus communis]	35.378	4.5563	6.4	2	2	2	0.968	0.883	0.937	1.111	1.034	1.097	1.163	1.23E-02

cds.Camellia_sinensis.comp25819_c1_seq1_m.34973	PREDICTED: protein TIC 21, chloroplastic [Sesamum indicum]	23.708	6.6089	6.2	1	2	1								
cds.Camellia_sinensis.comp25823_c0_seq1_m.34065	PREDICTED: B-cell receptor-associated protein 31-like [Nicotiana tabacum]	25.52	13.796	19	4	6	4	1.053	1.007	1.052	0.957	1.014	0.915	0.927	8.37E-02
cds.Camellia_sinensis.comp25824_c0_seq1_m.44079	plastid transcriptionally active protein [Medicago truncatula]	16.976	3.6322	5.6	1	2	1	0.982	1.012	0.956	1.038	0.985	1.042	1.039	1.94E-01
cds.Camellia_sinensis.comp25834_c0_seq1_m.24919	PREDICTED: probable fructose-bisphosphate aldolase 3, chloroplastic [Sesamum indicum]	44.523	93.59	42.6	14	24	13	1.013	1.011	1.02	0.983	0.969	1.014	0.974	1.28E-01
cds.Camellia_sinensis.comp25838_c0_seq1_m.32758	HN1-like protein [Medicago truncatula]	37.07	10.787	18.6	4	5	3	0.942	1.056	1.027	0.996	1	0.974	0.982	6.51E-01

cds.Camellia_sinensis.comp25843_c0_seq1_m.35993	PREDICTED: NADP-dependent malic enzyme isoform X4 [Nelumbo nucifera]	33.802	2.0501	7.4	3	3	2	1.018	0.912	0.973	1.064	1.035	1.017	1.073	1.07E-01
cds.Camellia_sinensis.comp25849_c0_seq1_m.26650	PREDICTED: NEDD8-activating enzyme E1 catalytic subunit isoform X1 [Eucalyptus grandis]	49.6	42.073	21.2	7	8	7	0.913	0.902	0.981	1.088	1.073	1.027	1.14	1.39E-02
cds.Camellia_sinensis.comp25857_c0_seq1_m.29195	PREDICTED: aldose 1-epimerase [Vitis vinifera]	40.357	18.702	11.2	4	6	4	1.037	0.987	0.908	1.017	0.972	1.058	1.039	4.39E-01
cds.Camellia_sinensis.comp25860_c0_seq1_m.29489	PREDICTED: pirin-like protein [Vitis vinifera]	38.139	5.561	4.4	1	1	1	0.987	1.205	1.038	0.96	0.902	0.901	0.855	7.36E-02
cds.Camellia_sinensis.comp25862_c0_seq1_m.10282	PREDICTED: ABC transporter G family member 7 [Vitis vinifera]	82.033	19.904	10.2	7	8	7	1.008	1.026	1.016	0.964	0.96	1.005	0.96	5.85E-02

cds.Camellia_sinensis.comp25863_c0_seq1_m.22745	acetyl-CoA carboxylase beta subunit (chloroplast) [Camellia sinensis]	57.987	20.483	18	7	8	7	1.034	0.985	0.989	1.035	0.967	0.959	0.984	6.05E-01
cds.Camellia_sinensis.comp25866_c0_seq1_m.12227	PREDICTED: NADH dehydrogenase [ubiquinone] iron-sulfur protein 1, mitochondrial isoform X1 [Capsicum	81.741	130.03	27.4	17	31	17	0.992	1.022	0.993	1.012	1.003	0.994	1.001	9.50E-01
cds.Camellia_sinensis.comp25868_c0_seq1_m.24454	PREDICTED: heterogeneous nuclear ribonucleoprotein 1-like [Malus domestica]	36.016	52.275	22.9	6	16	5	1.008	0.996	0.989	1.014	0.993	0.979	0.998	8.45E-01

cds.Camellia_sinensis.comp25879_c0_seq1_m.41545	PREDICTED: serine/threonine-protein phosphatase 6 regulatory subunit 2 isoform X1 [Vitis vinifera]	23.261	70.13	24.4	3	3	3	1.062	0.991	1.002	0.999	0.907	1.009	0.954	3.02E-01
cds.Camellia_sinensis.comp25884_c0_seq1_m.21957	PREDICTED: DNA ligase 1 [Camelina sativa]	42.672	5.2113	6.6	2	3	2	1.187	1.256	1.178	0.711	0.73	0.871	0.638	2.48E-03
cds.Camellia_sinensis.comp25886_c0_seq2_m.41532	PREDICTED: AP-2 complex subunit sigma [Sesamum indicum]	17.123	4.5003	12.7	2	2	2	0.915	0.911	0.964	1.078	1.085	1.082	1.163	1.42E-02
cds.Camellia_sinensis.comp25907_c0_seq2_m.21991	PREDICTED: ADP,ATP carrier protein 3, mitochondrial [Eucalyptus grandis]	41.431	62.364	23	10	40	5	0.955	0.957	1.003	1.041	1.047	1.023	1.067	2.01E-02

cds.Camellia_sinensis.comp25913_c0_seq1_m.35215	PREDICTED: multiple organellar RNA editing factor 2, chloroplastic [Nelumbo nucifera]	26.916	8.1784	15.9	4	8	4	1.045	1.044	1.072	0.922	0.894	0.96	0.878	4.34E-03
cds.Camellia_sinensis.comp25919_c0_seq1_m.29927	PREDICTED: RNA-binding protein sym-2 isoform X1 [Daucus carota subsp. sativus]	43.031	16.416	19.8	6	8	6	0.981	0.999	0.967	1	1.04	1.066	1.054	6.63E-02
cds.Camellia_sinensis.comp25924_c0_seq1_m.26348	glycoside hydrolase family 28 family protein [Populus trichocarpa]	49.412	37.121	11.6	5	5	5	0.929	0.965	1.012	0.994	1.024	1.062	1.06	1.35E-01
cds.Camellia_sinensis.comp25931_c0_seq1_m.22736	PREDICTED: sugar transport protein 14-like [Ipomoea nil]	56.199	24.9	8.9	3	5	3	0.913	0.983	0.925	1.103	1.098	0.908	1.102	2.40E-01

cds.Camellia_sinensis.comp25952_c0_seq2_m.14551	PREDICTED: cyclin-dependent kinase C-2 [Vitis vinifera]	57.561	2.5708	4.6	3	4	2	1.031	0.972	0.996	1.09	0.956	0.967	1.005	9.48E-01
cds.Camellia_sinensis.comp25955_c0_seq1_m.37552	PREDICTED: 60S ribosomal protein L6-1-like [Pyrus x bretschneideri]	26.046	12.725	28.8	7	11	4	1.132	1.114	1.149	0.884	0.875	0.848	0.768	6.36E-05
cds.Camellia_sinensis.comp25955_c0_seq2_m.40421	PREDICTED: 60S ribosomal protein L6-1-like [Pyrus x bretschneideri]	27.725	26.374	31.4	8	13	5	1.092	1.153	1.116	0.868	0.907	0.855	0.783	4.97E-04
cds.Camellia_sinensis.comp25987_c0_seq1_m.41894	PREDICTED: malate dehydrogenase, glyoxysomal [Fragaria vesca subsp. vesca]	16.653	9.0496	35.4	5	8	3	0.927	1.032	0.988	1.071	1.03	1.021	1.059	1.65E-01
cds.Camellia_sinensis.comp25989_c0_seq1_m.19437	PREDICTED: cysteine proteinase COT44 [Citrus sinensis]	40.341	127.46	32.5	9	20	8	1.097	1.086	1.059	0.909	0.895	0.93	0.843	3.65E-04

cds.Camellia_sinensis.comp25990_c0_seq1_m.47421	PREDICTED: probable glutathione S-transferase [Nicotiana attenuata]	16.041	6.961	27.7	7	20	5	1.027	0.975	0.947	0.974	0.974	1.056	1.019	6.37E-01
cds.Camellia_sinensis.comp25995_c0_seq1_m.33788	PREDICTED: multiple organellar RNA editing factor 1, mitochondrial [Vitis vinifera]	42.7	7.3164	9.4	3	4	3	1.055	0.98	1.015	0.966	0.955	1.027	0.967	3.34E-01
cds.Camellia_sinensis.comp25996_c0_seq2_m.49305		15.109	50.048	24.4	2	3	2	1.032	0.969	0.878	0.983	1.078	1.071	1.088	1.99E-01
cds.Camellia_sinensis.comp25998_c0_seq1_m.11174	PREDICTED: protein FRIGIDA [Sesamum indicum]	78.87	9.8811	5.8	4	4	4	1.023	1.059	0.999	0.959	0.948	1.028	0.953	1.83E-01
cds.Camellia_sinensis.comp26010_c0_seq1_m.30863	PREDICTED: putative 12-oxophytodienoate reductase 11 [Theobroma cacao]	40.996	102.17	30.2	9	17	8	0.942	0.998	0.973	1.002	1.012	1.023	1.043	7.75E-02

cds.Camellia_sinensis.comp26013_c0_seq1_m.23914	PREDICTED: triose phosphate/phosphate translocator, chloroplastic [Vitis vinifera]	48.08	45.616	12.5	6	12	6	0.921	0.913	0.958	1.073	1.083	1.082	1.16	6.00E-04
cds.Camellia_sinensis.comp26015_c0_seq1_m.29474	thiamine monophosphate synthase [Medicago truncatula]	32.552	43.382	30.5	8	11	8	1.008	0.977	0.974	1.001	1.067	0.986	1.032	3.06E-01
cds.Camellia_sinensis.comp26016_c0_seq1_m.33501	PREDICTED: V-type proton ATPase subunit E [Sesamum	26.333	69.18	45.9	10	23	7	1	0.982	0.962	1.01	1.026	1.04	1.045	3.52E-02
cds.Camellia_sinensis.comp26018_c0_seq1_m.23404	PREDICTED: serine--tRNA ligase [Vitis vinifera]	46.11	37.742	23.9	9	10	9	1.019	1.043	1.027	0.984	0.977	0.976	0.951	2.32E-03
cds.Camellia_sinensis.comp26023_c0_seq1_m.31133	uncharacterized LOC107830728 [Nicotiana tabacum]	31.217	67.113	19.8	5	9	5	1.054	1.056	0.95	0.983	1.028	0.94	0.964	4.57E-01

cds.Camellia_sinensis.comp26027_c0_seq1_m.7122	PREDICTED: probable galactinol--sucrose galactosyltransferase 6 isoform X1 [Populus euphratica]	93.927	323.31	34.1	25	62	25	1.11	1.106	1.111	0.88	0.89	0.872	0.794	1.43E-06
cds.Camellia_sinensis.comp26033_c0_seq1_m.41032	PREDICTED: coiled-coil domain-containing protein 25 isoform X1 [Vitis vinifera]	25.144	1.1908	7	2	2	2	1.059	1.163	1.098	0.854	0.818	0.979	0.798	2.00E-02
cds.Camellia_sinensis.comp26040_c0_seq1_m.20949	uncharacterized LOC107810057 [Nicotiana tabacum]	47.193	1.5038	1.9	1	1	1								
cds.Camellia_sinensis.comp26042_c0_seq1_m.30245	PREDICTED: putative lactoylglutathione lyase isoform X2 [Vitis vinifera]	32.799	25.911	45	12	48	1	0.942	0.955	0.989	1.045	1.08	1.023	1.091	1.54E-02

cds.Camellia_sinensis.comp26042_c0_seq2_m.31341	PREDICTED: putative lactoylglutathione lyase isoform X2 [Vitis vinifera]	32.712	144.92	49	13	56	2	0.942	0.966	0.953	1.057	1.051	1.043	1.101	3.01E-04
cds.Camellia_sinensis.comp26046_c0_seq1_m.33895	PREDICTED: DNA-damage-repair/toleration protein DRT102 [Nelumbo nucifera]	35.947	96.526	33.2	9	18	9	0.953	0.963	0.958	1.04	1.03	1.036	1.081	4.33E-05
cds.Camellia_sinensis.comp260594_c0_seq1_m.50831	PREDICTED: non-specific phospholipase C4 isoform X2 [Vitis vinifera]	20.397	9.6276	17.1	3	4	3	0.933	1.044	1.122	0.85	0.966	1.085	0.936	4.85E-01
cds.Camellia_sinensis.comp26059_c0_seq1_m.33749	PREDICTED: NADH--cytochrome b5 reductase 1 [Jatropha curcas]	33.016	29.643	20	7	10	6	0.991	0.971	1.002	0.973	1.012	0.977	0.999	9.64E-01

cds.Camellia_sinensis.comp26061_c0_seq1_m.36997	PREDICTED: aminopeptidase M1 isoform X2 [Vitis vinifera]	39.536	29.099	17.5	6	12	5	0.956	0.98	0.922	1.052	1.073	1.055	1.113	4.66E-03
cds.Camellia_sinensis.comp26068_c0_seq1_m.26871	PREDICTED: biotin carboxyl carrier protein of acetyl-CoA carboxylase [Prunus mume]	30.685	13.609	18.4	5	7	5	1.013	1.07	1.021	0.98	0.991	0.978	0.95	4.42E-02
cds.Camellia_sinensis.comp26073_c0_seq1_m.30288	PREDICTED: 33 kDa ribonucleoprotein, chloroplastic [Juglans regia]	35.214	49.763	19.3	6	10	6	1.016	1.004	1.07	0.981	0.969	0.976	0.947	5.27E-02
cds.Camellia_sinensis.comp26081_c0_seq1_m.45814	PREDICTED: L10-interacting MYB domain-containing protein-like [Cucumis melo]	19.175	3.2047	6.1	1	1	1	0.907	0.959	0.958	0.993	1.252	0.966	1.137	2.22E-01

cds.Camellia_sinensis.comp26091_c0_seq1_m.17776	PREDICTED: protein TIC 56, chloroplastic-like [Gossypium hirsutum]	59.411	12.805	9.8	5	6	5	0.933	1.032	1.008	1.044	1.029	1.003	1.035	3.46E-01
cds.Camellia_sinensis.comp26098_c0_seq4_m.50935	PREDICTED: macrophage migration inhibitory factor homolog [Eucalyptu	13.099	46.866	15.6	2	5	2	1.024	0.992	1.011	1.005	0.991	1.017	0.995	7.18E-01
cds.Camellia_sinensis.comp26102_c0_seq1_m.29197	PREDICTED: phosphopantothenate--cysteine ligase 2-like [Juglans regia]	36.207	22.255	12.9	4	4	4	1.062	1.021	0.974	0.998	1.009	0.922	0.958	3.20E-01
cds.Camellia_sinensis.comp26103_c1_seq1_m.10502	PREDICTED: violaxanthin de-epoxidase, chloroplastic [Vitis vinifera]	53.757	137.54	38.3	14	25	14	0.96	0.988	0.965	1.052	1.026	1.06	1.077	4.94E-03

cds.Camellia_sinensis.comp26108_c0_seq1_m.26404	PREDICTED: GDSL esterase/lipase At1g09390-like isoform X1 [Juglans regia]	42.298	2.0821	2.6	1	1	1								
cds.Camellia_sinensis.comp26112_c0_seq1_m.42689	PREDICTED: dihydrolipoyllysine-residue succinyltransferase component of 2-oxoglutarate dehydrogenase complex 1, mitochondrial-like isoform X3 [Populus euphratica]	21.758	-2	33.3	7	11	1								
cds.Camellia_sinensis.comp26118_c0_seq1_m.40510	PREDICTED: auxin-binding protein ABP19a [Eucalyptus grandis]	22.317	22.198	15.6	3	14	3	0.992	0.94	0.987	1.009	1.029	1.008	1.044	8.08E-02

cds.Camellia_sinensis.comp26123_c0_seq1_m.25847	PREDICTED: heavy metal-associated isoprenylated plant protein 3-like isoform X2 [Juglans regia]	39.119	2.0889	3.3	1	1	1	0.896	0.973	1.006	1.137	0.991	1.033	1.099	1.51E-01
cds.Camellia_sinensis.comp26126_c0_seq1_m.48294	PREDICTED: ras-related protein RABD2a-like [Nicotiana tabacum]	17.122	1.5292	28	5	11	1	1.01	1.027	0.996	1.008	0.978	0.989	0.981	1.97E-01
cds.Camellia_sinensis.comp26133_c0_seq1_m.13320	PREDICTED: regulation of nuclear pre-mRNA domain-containing protein 1B-like [Prunus mume]	60.867	3.8386	4.3	3	3	3	0.993	1.007	0.98	1.027	1.001	1.005	1.018	1.90E-01

cds.Camellia_sinensis.comp26136_c0_seq1_m.26384	PREDICTED: probable tocopherol O-methyltransferase, chloroplastic isoform X4 [Juglans regia]	39.994	31.387	23.6	7	10	7	1.004	1.005	1.049	0.982	0.986	0.975	0.962	6.18E-02
cds.Camellia_sinensis.comp26137_c0_seq1_m.14029	PREDICTED: flavonoid 3',5'-hydroxylase 2-like [Jatropha curcas]	57.074	16.113	17.6	8	10	8	1.242	1.363	1.379	0.639	0.682	0.588	0.479	1.65E-04
cds.Camellia_sinensis.comp26138_c0_seq1_m.38810	PREDICTED: non-symbiotic hemoglobin 1 [Ricinus communis]	26.452	34.161	31.9	8	16	8	0.979	0.979	0.974	1.03	1.065	1.003	1.057	8.81E-02
cds.Camellia_sinensis.comp26139_c1_seq1_m.19052	PREDICTED: UDP-glucose 4-epimerase GEPI48 [Sesamum indicum]	37.964	139.53	51.1	15	39	14	0.974	0.94	0.941	1.059	1.092	1.045	1.119	2.98E-03

cds.Camellia_sinensis.comp26157_c0_seq1_m.39234	PREDICTED: proteasome subunit beta type-1-like [Nicotiana tabacum]	24.645	6.4214	32.3	6	9	2	0.938	0.973	0.983	1.082	1.023	1.006	1.075	5.15E-02
cds.Camellia_sinensis.comp26157_c0_seq2_m.39673	PREDICTED: proteasome subunit beta type-1 [Ziziphus jujuba]	25.058	111.77	54.4	10	15	6	0.994	0.959	0.94	1.022	1.059	1.031	1.076	2.00E-02
cds.Camellia_sinensis.comp26160_c0_seq1_m.26066	PREDICTED: probable alpha-amylase 2 [Jatropha curcas]	46.952	4.5604	6.3	3	3	3	1.097	0.953	0.976	0.972	0.982	1.015	0.981	7.23E-01
cds.Camellia_sinensis.comp26168_c0_seq1_m.29601	PREDICTED: peroxidase 73 [Vitis vinifera]	35.455	29.482	34.9	9	17	7	1.011	1.044	1.031	0.986	0.945	0.946	0.932	1.40E-02
cds.Camellia_sinensis.comp26171_c0_seq2_m.40947	PREDICTED: NADPH--cytochrome P450 reductase 2 [Prunus mume]	20.603	4.2638	7.5	1	1	1	1.296	1.3	1.218	0.754	0.658	0.691	0.551	1.85E-04

cds.Camellia_sinensis.comp26177_c0_seq1_m.41667	PREDICTED: 60S ribosomal protein L31 [Juglans regia]	14.069	19.705	33.1	3	3	3	1.113	1.042	1.099	0.951	0.87	0.913	0.84	5.96E-03
cds.Camellia_sinensis.comp26178_c0_seq1_m.25671	E3 ubiquitin-protein ligase [Arabidopsis thaliana]	52.465	13.562	8.9	4	5	4	1.016	0.858	0.981	0.951	0.985	1.166	1.087	3.73E-01
cds.Camellia_sinensis.comp26181_c0_seq1_m.29403	PREDICTED: protein LOW PSII ACCUMULATION 3, chloroplastic [Vitis vinifera]	37.155	8.0744	8.6	3	6	3	1.007	0.993	1.016	0.987	1.024	0.992	0.996	7.57E-01
cds.Camellia_sinensis.comp26184_c0_seq1_m.32281	PREDICTED: guanine nucleotide-binding protein subunit beta-like protein [Erythranthe guttata]	35.931	136.94	36.7	10	29	10	0.959	0.959	0.954	1.043	1.052	1.017	1.084	1.48E-03

cds.Camellia_sinensis.comp26187_c0_seq1_m.24832	PREDICTED: 40S ribosomal protein S3a [Sesamum indicum]	29.806	68.408	48.7	10	22	10	1.097	1.12	1.091	0.845	0.876	0.866	0.782	5.51E-05
cds.Camellia_sinensis.comp26191_c0_seq11_c0_seq1_m.25177	PREDICTED: embryogenesis-associated protein EMB8 isoform X1 [Nelumbo nucifera]	44.928	3.538	5.8	1	1	1	0.707	1.247	1.171	1.188	1.033	0.704	0.936	8.15E-01
cds.Camellia_sinensis.comp26199_c0_seq1_m.17298	PREDICTED: pto-interacting protein 1 isoform X2 [Sesamum indicum]	38.825	74.727	34.4	11	19	10	0.986	0.957	0.995	1.016	1.012	1.022	1.038	3.62E-02
cds.Camellia_sinensis.comp26206_c0_seq2_m.39660	PREDICTED: binding partner of ACD11 1 [Theobroma cacao]	27.643	22.639	31.5	8	13	6	1.103	1.02	1.045	0.97	0.957	0.945	0.907	1.60E-02
cds.Camellia_sinensis.comp26206_c0_seq3_m.40252	PREDICTED: binding partner of ACD11 1 [Theobroma cacao]	27.591	12.816	25.6	7	10	6	0.985	0.966	0.997	0.972	1.055	1.045	1.042	2.13E-01

cds.Camellia_sinensis.comp26210_c0_seq1_m.33336	PREDICTED: protein SGT1 homolog A-like [Jatropha curcas]	36.949	14.116	26.7	9	10	8	0.973	0.959	0.971	1.036	1.001	1.041	1.06	1.13E-02
cds.Camellia_sinensis.comp26219_c0_seq1_m.29148	PREDICTED: protein SLOW GREEN 1, chloroplastic-like isoform X2 [Gossypium hirsutum]	45.082	4.3655	7.6	3	3	3	0.957	0.898	0.947	1.096	1.082	1.052	1.153	3.52E-03
cds.Camellia_sinensis.comp26222_c0_seq1_m.35005	PREDICTED: GDT1-like protein 4 [Cucumis melo]	24.773	1.5575	3.9	1	1	1	0.981	0.977	0.983	1.061	0.978	1.037	1.046	2.10E-01
cds.Camellia_sinensis.comp26231_c0_seq1_m.27340	PREDICTED: isocitrate dehydrogenase [NAD] regulatory subunit 1, mitochondrial isoform X1 [Ricinus	40.735	51.525	25	9	16	9	0.976	0.998	0.986	1.036	1.015	1.034	1.042	1.07E-02

cds.Camellia_sinensis.comp26232_c0_seq32_m.36763	DNA-binding protein [Medicago truncatula]	24.035	2.8459	5.1	1	1	1	0.98	1.047	1.134	0.96	0.947	0.933	0.898	7.04E-02
cds.Camellia_sinensis.comp26236_c1_seq1_m.48309		18.403	4.3686	10.6	1	1	1								
cds.Camellia_sinensis.comp26246_c0_seq1_m.30448	bifunctional dTDP-4-dehydrorhamnose 3,5-epimerase/dTDP-4-dehydrorhamnose reductase-like [Gossypium m	35.01	7.5402	13.8	4	5	3	1.053	1.05	1.026	0.914	1.018	0.932	0.915	5.87E-02
cds.Camellia_sinensis.comp26248_c0_seq1_m.28827	PREDICTED: 3-deoxy-manno-octulosonate cytidylyltransferase, mitochondrial-like isoform X1 [Nicotiana attenuata]	34.261	105.94	32.6	6	11	6	0.998	0.978	0.982	1.015	1.014	0.992	1.021	9.58E-02

cds.Camellia_sinensis.comp26250_c0_seq1_m.2532	PREDICTED: starch synthase 3, chloroplastic/amyloplastic isoform X1 [Ziziphus jujuba]	83.105	47.688	21.2	12	13	12	1.055	1.097	1.072	0.909	0.914	0.91	0.848	4.78E-03
cds.Camellia_sinensis.comp26250_c0_seq1_m.2533	PREDICTED: starch synthase 3, chloroplastic/amyloplastic [Vitis vinifera]	62.555	13.859	11.7	6	7	6	1.182	1.131	1.058	0.849	0.829	0.917	0.77	4.12E-03
cds.Camellia_sinensis.comp26251_c0_seq1_m.32703	Glutelin type-B 5 [Morus notabilis]	39.432	54.639	30.6	11	18	11	0.909	0.925	0.921	1.077	1.076	1.09	1.177	1.84E-05
cds.Camellia_sinensis.comp26252_c0_seq1_m.25284	PREDICTED: beta-glucosidase 12-like [Nicotiana sylvestris]	56.574	24.816	12	6	9	5	1.011	0.973	0.967	1.039	1.005	1.026	1.04	7.97E-02
cds.Camellia_sinensis.comp26255_c0_seq2_m.14870	PREDICTED: peptidyl-prolyl cis-trans isomerase FKBP20-1 isoform X1 [Nelumbo nucifera]	20.505	4.3603	6.4	1	1	1	1.112	1.17	1.064	0.903	0.839	0.884	0.785	2.32E-03

cds.Camellia_sinensis.comp26268_c0_seq1_m.37228	PREDICTED: probable protein phosphatase 2C 58 [Sesamum indicum]	31.569	10.595	10.8	3	5	1	0.965	0.874	1.065	1.041	1.013	1.063	1.073	2.77E-01
cds.Camellia_sinensis.comp26270_c0_seq1_m.8002	PREDICTED: casein kinase 1-like protein HD16 isoform X2 [Ipomoea nil]	78.982	1.5441	2.1	1	1	1	0.997	0.859	1.104	0.986	1.017	1.05	1.031	6.58E-01
cds.Camellia_sinensis.comp26273_c0_seq1_m.33649	PREDICTED: adenylate kinase, chloroplastic-like [Populus euphratica]	33.475	28.9	21.3	6	14	5	0.99	1.034	0.937	1.013	0.992	1.002	1.016	6.08E-01
cds.Camellia_sinensis.comp26283_c0_seq1_m.30031	PREDICTED: signal recognition particle receptor subunit beta-like [Sesamum indicum]	29.25	16.622	23.4	5	9	5	0.982	0.951	0.947	1.103	0.987	1.047	1.089	6.81E-02

cds.Camellia_sinensis.comp26285_c0_seq1_m.46008	PREDICTED: glutathione S-transferase T1 [Ricinus communis]	19.873	10.854	14.1	2	2	2	0.99	0.986	1.066	0.957	1.016	0.992	0.975	4.57E-01
cds.Camellia_sinensis.comp26288_c0_seq1_m.43846	PREDICTED: subtilisin-like protease SBT3.11 [Pyrus x bretschneideri]	14.398	36.904	34.1	4	7	4	0.945	0.92	0.901	1.095	1.104	1.134	1.205	4.24E-04
cds.Camellia_sinensis.comp26290_c0_seq1_m.21347	PREDICTED: eukaryotic initiation factor 4A-3 [Vitis vinifera]	46.559	78.079	33.5	16	28	14	0.971	0.961	0.977	1.031	1.021	1.037	1.062	8.19E-04
cds.Camellia_sinensis.comp26296_c0_seq1_m.39538	PREDICTED: phosphopantetheine adenylyltransferase [Theobroma cacao]	26.013	19.037	30.8	7	9	7	0.988	1.026	0.97	1.025	0.996	1.012	1.016	4.23E-01
cds.Camellia_sinensis.comp26305_c0_seq1_m.2721	PREDICTED: protein TPLATE [Ipomoea nil]	101.42	7.7555	3.2	2	2	2	1.148	1.054	0.924	0.896	0.98	0.978	0.913	2.76E-01

cds.Camellia_sinensis.comp26309_c0_seq1_m.35452	PREDICTED: mitochondrial import receptor subunit TOM20 [Vitis vinifera]	27.97	57.458	37.2	10	15	10	0.915	0.958	0.969	1.044	1.093	1.092	1.136	5.28E-03
cds.Camellia_sinensis.comp26322_c0_seq3_m.1497	PREDICTED: ARF guanine-nucleotide exchange factor GNOM [Jatropha curcas]	139.95	1.9317	2.2	3	3	3	1.193	0.963	1.029	0.957	0.879	0.955	0.876	1.36E-01
cds.Camellia_sinensis.comp26336_c0_seq1_m.25208	PREDICTED: glutamate dehydrogenase 2 [Theobroma cacao]	47.284	9.4171	13.9	5	7	2	0.96	1.212	0.925	0.97	0.977	0.958	0.938	5.72E-01
cds.Camellia_sinensis.comp26365_c0_seq1_m.37925	PREDICTED: aldose 1-epimerase-like [Nicotiana tabacum]	34.723	12.984	17.5	6	9	6	0.972	0.936	0.924	1.028	1.04	1.086	1.114	8.86E-03

cds.Camellia_sinensis.comp26377_c0_seq1_m.30105	PREDICTED: protein farnesyltransferase/geranylgeranyltransferase type-1 subunit alpha [Vitis vinifera]	28.17	24.113	18.3	4	4	4	0.943	0.992	0.986	1.044	1.029	1.023	1.06	2.68E-02
cds.Camellia_sinensis.comp26384_c0_seq1_m.38788	PREDICTED: 40S ribosomal protein S12 [Nelumbo nucifera]	15.184	18.403	56.2	6	11	3	1.021	0.983	1.141	0.968	0.838	1.043	0.906	2.67E-01
cds.Camellia_sinensis.comp26384_c0_seq2_m.39037	PREDICTED: 40S ribosomal protein S12 [Nelumbo nucifera]	15.152	27.678	62.5	7	12	4	0.959	0.986	0.959	1.025	1.08	1.013	1.074	3.12E-02
cds.Camellia_sinensis.comp26388_c0_seq1_m.18287	PREDICTED: probable transaldolase [Sesamum indicum]	48.969	95.198	35.3	13	20	13	0.954	1.013	0.974	1.042	1.037	1.015	1.052	5.80E-02

cds.Camellia_sinensis.comp26389_c0_seq1_m.39277	PREDICTED: ESCRT-related protein CHMP1B-like [Nelumbo nucifera]	22.603	28.494	32.5	7	8	7	1.042	1.06	1.064	0.979	0.914	0.951	0.898	6.78E-03
cds.Camellia_sinensis.comp26398_c0_seq2_m.42894	PREDICTED: miraculin [Theobroma cacao]	24.856	4.7454	9.9	2	3	2	0.733	0.826	0.764	1.235	1.352	1.182	1.622	7.95E-04
cds.Camellia_sinensis.comp26401_c0_seq4_m.10602	PREDICTED: uncharacterized aarF domain-containing protein kinase At1g79600, chloroplastic [Nicotiana sylvestris]	80.481	8.1099	6.8	5	5	4								
cds.Camellia_sinensis.comp26404_c0_seq1_m.35741	PREDICTED: superoxide dismutase [Fe], chloroplastic [Vitis vinifera]	34.096	33.438	22.7	6	11	6	1.011	0.999	0.963	0.966	1.044	1.013	1.017	5.76E-01

cds.Camellia_sinensis.comp26405_c0_seq1_m.42130	PREDICTED: eukaryotic translation initiation factor [Vitis vinifera]	22.537	21.756	23.2	5	7	5	0.954	0.984	0.977	1.025	1.006	1.009	1.043	1.88E-02
cds.Camellia_sinensis.comp26408_c0_seq1_m.45653	PREDICTED: brain acid soluble protein 1 homolog [Pyrus x bretschneideri]	17.82	8.249	7.3	1	1	1	0.911	0.924	0.917	1.162	1.025	1.1	1.194	4.04E-02
cds.Camellia_sinensis.comp26411_c0_seq1_m.4265	PREDICTED: vacuolar protein-sorting-associated protein 11 homolog isoform X3 [Vitis vinifera]	108.63	10.183	8	8	9	8	1.04	0.988	1.039	0.987	0.972	0.978	0.958	6.99E-02
cds.Camellia_sinensis.comp26419_c0_seq1_m.19908	PREDICTED: probable xyloglucan endotransglucosylase/hydrolase protein 6 [Juglans regia]	32.893	26.298	26.8	8	11	6	1.24	1.256	1.279	0.758	0.72	0.685	0.573	5.66E-05

cds.Camellia_sinensis.comp26421_c0_seq2_m.36917	PREDICTED: peptidyl-prolyl cis-trans isomerase CYP23 [Populus euphratica]	26.056	1.1538	4.3	1	1	1	1.042	0.983	1.005	0.98	0.949	1.042	0.981	5.66E-01
cds.Camellia_sinensis.comp26429_c0_seq1_m.30304	PREDICTED: protein SLOW GREEN 1, chloroplastic-like [Nelumbo nucifera]	37.781	6.4794	9.3	3	4	3	0.912	1.008	0.974	1.049	1.033	1.051	1.083	5.50E-02
cds.Camellia_sinensis.comp26433_c0_seq1_m.39369	PREDICTED: signal peptidase complex subunit 3B [Arachis ipaensis]	19.062	8.4826	6.6	1	1	1	0.986	0.918	0.95	1.044	1.079	1.046	1.11	1.07E-02
cds.Camellia_sinensis.comp26441_c0_seq1_m.17335	PREDICTED: protein DETOXIFICATION 40-like [Daucus carota subsp. sativus]	59.44	2.5212	3.8	2	2	2	1.05	1.075	1.09	0.828	0.826	1.107	0.859	2.40E-01

cds.Camellia_sinensis.comp26443_c0_seq1_m.27088	PREDICTED: guanylate kinase 2, chloroplastic/mitochondrial [Ipomoea nil]	33.997	3.1263	5.9	2	2	2	0.943	0.941	0.91	1.069	1.115	1.054	1.159	1.98E-03
cds.Camellia_sinensis.comp26447_c0_seq1_m.21594	import inner membrane translocase subunit [Arabidopsis thaliana]	41.221	20.511	19.9	6	7	6	1.035	1.034	0.947	0.959	1.17	1.015	1.042	5.88E-01
cds.Camellia_sinensis.comp26451_c0_seq1_m.29519	PREDICTED: nitrilase-like protein 2 isoform X1 [Solanum pennellii]	35.051	224.18	67.1	18	44	16	1.019	1.024	1.007	0.981	0.986	0.973	0.964	4.30E-03
cds.Camellia_sinensis.comp26453_c0_seq1_m.38607	PREDICTED: chaperone protein DnaJ-like [Gossypium hirsutum]	13.938	4.1846	18.3	3	3	3	0.943	0.935	0.957	1.033	1.083	1.077	1.126	1.92E-03

cds.Camellia_sinensis.comp26455_c0_seq1_m.33143	PREDICTED: proline--tRNA ligase, cytoplasmic [Arachis ipaensis]	45.73	11.776	30	10	13	4	1.066	1.06	1.088	0.901	0.938	0.954	0.869	1.68E-03
cds.Camellia_sinensis.comp26455_c1_seq1_m.45521	PREDICTED: proline--tRNA ligase, cytoplasmic-like [Nelumbo nucifera]	17.849	18.132	34	5	5	3	1.167	1.049	1.073	0.802	0.936	0.879	0.796	1.43E-02
cds.Camellia_sinensis.comp26458_c0_seq2_m.35806	dimethylallyl, adenosine tRNA methylthiotransferase [Arabidopsis thaliana]	27.97	75.965	28.4	7	17	7	0.993	0.964	0.982	0.993	1.034	1.009	1.033	9.06E-02
cds.Camellia_sinensis.comp26460_c0_seq1_m.7641	PREDICTED: ferric reduction oxidase 7, chloroplastic isoform X1 [Vitis vinifera]	83.622	31.494	8.9	6	7	6	1.031	1.042	1.059	0.938	0.953	0.944	0.905	3.84E-04

cds.Camellia_sinensis.comp26464_c0_seq1_m.23457	PREDICTED: aspartate aminotransferase, cytoplasmic [Theobroma cacao]	52.11	52.291	30.5	12	18	11	0.936	0.965	0.989	1.032	1.032	1.023	1.068	1.50E-02
cds.Camellia_sinensis.comp26465_c0_seq1_m.51897	PREDICTED: 3-hydroxybenzoate 6-hydroxylase 1 [Ricinus communis]	16.335	1.425	6.2	1	1	1	0.975	1.013	0.939	1.022	1.002	1.063	1.055	1.29E-01
cds.Camellia_sinensis.comp26474_c0_seq1_m.6815	PREDICTED: WD repeat-containing protein 75 [Juglans regia]	91.437	4.875	2.7	2	2	2	0.961	1.096	0.989	1.068	0.866	1.03	0.973	7.15E-01
cds.Camellia_sinensis.comp26478_c0_seq1_m.44479	PREDICTED: adrenodoxin-like protein 2, mitochondrial [Sesamum indicum]	19.988	1.6865	7.2	1	2	1								
cds.Camellia_sinensis.comp26484_c0_seq1_m.27782	PREDICTED: protein PELOTA 1 [Nicotiana	42.383	2.0467	2.1	1	1	1								

cds.Camellia_sinensis.comp26490_c0_seq1_m.14796	PREDICTED: carboxyl-terminal-processing peptidase 3, chloroplastic [Sesamum indicum]	59.942	11.041	7.8	4	5	4	0.973	1.025	1.004	1.024	0.995	0.99	1.002	9.01E-01
cds.Camellia_sinensis.comp26493_c0_seq1_m.11502	PREDICTED: mitochondrial Rho GTPase 1 [Vitis vinifera]	71.486	26.78	16.7	11	14	9	0.991	0.987	1.026	0.987	1.019	0.985	0.996	8.07E-01
cds.Camellia_sinensis.comp26495_c1_seq2_m.22967	PREDICTED: nudix hydrolase 19, chloroplastic [Solanum tuberosum	21.613	34.288	25.5	5	6	5	1.121	0.961	0.989	1.064	0.794	1.063	0.951	6.27E-01
cds.Camellia_sinensis.comp26495_c1_seq2_m.22968	PREDICTED: nudix hydrolase 19, chloroplastic-like [Malus domestica]	21.464	15.087	15.6	3	3	3	1.02	0.982	0.997	1.014	0.998	1	1.004	7.34E-01

cds.Camellia_sinensis.comp26498_c0_seq1_m.17905	PREDICTED: probable 26S proteasome non-ATPase regulatory subunit 3 [Vitis vinifera]	55.354	107.85	41.1	18	30	18	0.996	1	1.018	1.002	1.024	0.961	0.991	6.63E-01
cds.Camellia_sinensis.comp26504_c0_seq2_m.48391	PREDICTED: replication factor C subunit 2 [Lupinus angustifolius]	16.02	18.242	28.4	3	3	3	0.986	0.945	0.934	1.106	1.036	1.018	1.103	3.16E-02
cds.Camellia_sinensis.comp26506_c0_seq1_m.23107	PREDICTED: altered inheritance of mitochondria protein 32 [Vitis vinifera]	44.506	53.279	21.8	8	22	7	1.077	1.077	1.052	0.913	0.923	0.904	0.855	9.56E-05
cds.Camellia_sinensis.comp26512_c0_seq1_m.11629	PREDICTED: chaperone protein DnaJ [Citrus sinensis]	34.913	49.411	31.8	8	11	8	1.042	1.05	1.092	0.926	0.947	0.936	0.882	1.40E-03

cds.Camellia_sinensis.comp26513_c0_seq2_m.30635	PREDICTED: THO complex subunit 3 [Sesamum indicum]	35.12	4.2404	6.4	2	2	2	1.014	1.001	1.083	0.991	1.001	0.915	0.938	1.63E-01
cds.Camellia_sinensis.comp26525_c0_seq1_m.24481	PREDICTED: 26S protease regulatory subunit S10B homolog B [Vigna radiata var. radiata]	44.597	124.64	49.5	18	32	17	0.991	0.987	0.964	1.043	1.006	1.029	1.046	2.94E-02
cds.Camellia_sinensis.comp26526_c0_seq2_m.10699	PREDICTED: actin-related protein 5-like isoform X2 [Juglans regia]	35.546	1.2195	3.2	1	1	1								
cds.Camellia_sinensis.comp26531_c0_seq1_m.8009	PREDICTED: phospholipase D alpha 1 [Sesamum indicum]	101.33	104.61	33	27	40	23	0.968	0.968	0.959	1.046	1.005	1.065	1.076	1.36E-02

cds.Camellia_sinensis.comp26543_c0_seq1_m.53696	PREDICTED: probable mediator of RNA polymerase II transcription subunit 37c, partial [Raphanus sativus]	16.527	3.4618	19.9	3	7	1	0.944	0.972	1.242	0.901	0.914	1.028	0.9	3.63E-01
cds.Camellia_sinensis.comp26551_c0_seq1_m.14734	PREDICTED: GPI transamidase component PIG-T [Vitis vinifera]	74.92	1.9067	1.3	1	3	1	1.025	0.983	1.035	0.94	1.046	0.986	0.977	5.22E-01
cds.Camellia_sinensis.comp26555_c0_seq1_m.49470	PREDICTED: 60S ribosomal protein L28-2-like [Nelumbo nucifera]	19.166	18.148	32.2	6	10	6	1.141	1.081	1.164	0.847	0.835	0.855	0.749	2.40E-04
cds.Camellia_sinensis.comp26557_c0_seq1_m.48630	PREDICTED: phenylalanine ammonia-lyase [Nelumbo nucifera]	14.501	3.5862	33.8	4	4	2	1.442	1.345	1.454	0.473	0.552	0.597	0.382	1.83E-04

cds.Camellia_sinensis.comp26559_c0_seq1_m.17417	PREDICTED: aspartate--tRNA ligase 2, cytoplasmic-like [Nelumbo nucifera]	60.841	128.93	33.9	17	31	17	1.02	0.999	1.004	0.992	0.992	0.984	0.982	5.49E-02
cds.Camellia_sinensis.comp26584_c0_seq2_m.37309	PREDICTED: universal stress protein PHOS32 [Ipomoea nil]	25.465	51.074	24.8	5	9	5	0.998	1.012	1.033	0.984	0.989	0.952	0.961	6.40E-02
cds.Camellia_sinensis.comp26587_c0_seq1_m.16802		50.306	1.4097	2.3	1	1	1	0.942	0.961	0.955	1.102	0.91	1.154	1.108	3.14E-01
cds.Camellia_sinensis.comp26589_c0_seq1_m.29291	26S proteasome regulatory subunit RPN13 [Cajanus cajan]	40.619	24.896	12	3	3	3	0.979	0.979	1.033	1.022	1.003	1.021	1.018	3.83E-01
cds.Camellia_sinensis.comp26590_c0_seq1_m.37732	putative linoleate 9S-lipoxygenase 5 [Morus notabilis]	37.758	8.548	17	5	7	3	1.073	1.068	1.028	0.88	1.014	0.923	0.889	5.28E-02

cds.Camellia_sinensis.comp26592_c1_seq1_m.15592	PREDICTED: betaine aldehyde dehydrogenase 1, chloroplastic [Ziziphus jujuba]	57.088	109.51	26.4	11	20	11	1.009	0.987	0.973	0.996	1.008	1.015	1.017	2.32E-01
cds.Camellia_sinensis.comp26604_c0_seq4_m.22113	PREDICTED: phosphatidylinositol 3,4,5-trisphosphate 3-phosphatase and protein-tyrosine-phosphatase PTEN2A[Ricinus communis]	47.181	8.6407	13.6	5	5	5	0.976	1.123	1.047	1.001	0.918	0.937	0.908	1.18E-01
cds.Camellia_sinensis.comp26610_c0_seq1_m.1613	PREDICTED: ABC transporter B family member 19 [Ricinus	136.05	1.8935	3.3	4	4	2								

cds.Camellia_sinensis.comp26614_c0_seq1_m.15318	PREDICTED: beta-fructofuranosidase, insoluble isoenzyme CWINV1 [Eucalyptus grandis]	65.106	62.079	23.1	13	20	13	0.937	0.99	0.984	1.036	1.046	1.039	1.072	1.66E-02
cds.Camellia_sinensis.comp26617_c0_seq1_m.24495	DUF4336 domain protein [Medicago truncatula]	50.59	55.652	15.4	8	11	8	0.997	0.982	0.954	1.026	0.995	1.03	1.04	8.01E-02
cds.Camellia_sinensis.comp26626_c0_seq1_m.37458	rubredoxin family protein [Populus trichocarpa]	17.331	7.4221	15.7	3	6	3	0.993	0.977	0.99	0.989	0.99	1.051	1.024	3.29E-01
cds.Camellia_sinensis.comp26628_c0_seq1_m.25079	Protein XAP5 CIRCADIAN TIMEKEEPER [Morus notabilis]	33.574	5.7626	7.2	1	1	1	0.818	1.194	1.05	1.022	0.935	1.003	0.967	8.53E-01
cds.Camellia_sinensis.comp26630_c0_seq1_m.40606	PREDICTED: 2-methyl-6-phytyl-1,4-hydroquinone methyltransferase, chloroplastic-like [Jatropha curcas]	16.555	1.8012	14.6	2	3	2	1.033	0.912	1.076	0.842	1.074	1.06	0.985	8.52E-01

cds.Camellia_sinensis.comp26631_c0_seq1_m.33058	PREDICTED: non-functional NADPH-dependent codeinone reductase 2-like [Daucus carota subsp. sativus]	36.923	145.33	55.2	18	56	16	0.924	0.934	0.942	1.063	1.084	1.064	1.147	8.28E-05
cds.Camellia_sinensis.comp26640_c0_seq1_m.35494	PREDICTED: endonuclease 1 [Nicotiana attenuata]	35.181	3.5282	2.3	1	1	1	1.014	1.011	1.067	0.96	0.972	0.978	0.941	3.01E-02
cds.Camellia_sinensis.comp26646_c0_seq1_m.24007	saccharopine dehydrogenase [Medicago truncatula]	33.778	39.351	15.9	5	7	5	0.957	0.956	0.992	1.056	1.004	1.071	1.078	3.18E-02
cds.Camellia_sinensis.comp26646_c0_seq1_m.24008	Saccharopine dehydrogenase [Arabidopsis thaliana]	17.77	7.0217	10.6	1	1	1								
cds.Camellia_sinensis.comp26652_c1_seq1_m.22429	PREDICTED: protein BTR1 isoform X2 [Vitis vinifera]	34.778	42.686	26.6	8	11	6	0.984	1.032	1.047	0.962	0.954	0.965	0.941	3.30E-02

cds.Camellia_sinensis.comp26652_c1_seq3_m.38958	PREDICTED: protein BTR1 isoform X2 [Vitis vinifera]	26.963	9.3883	26	6	7	4	1.093	0.839	0.735	1.072	1.143	1.138	1.257	1.10E-01
cds.Camellia_sinensis.comp26653_c0_seq1_m.18036	PREDICTED: transducin beta-like protein 2 [Nicotiana sylvestris]	46.342	23.642	14	5	8	5	1.004	1.042	1.033	1.076	0.923	0.964	0.962	4.39E-01
cds.Camellia_sinensis.comp26664_c0_seq1_m.10560	PREDICTED: N6-adenosine-methyltransferase MT-A70-like [Vitis vinifera]	80.741	5.5668	4.1	3	3	3	1.053	1.095	1.001	1.048	0.99	0.814	0.906	2.67E-01
cds.Camellia_sinensis.comp26664_c1_seq1_m.34799	PREDICTED: ATP-dependent Clp protease proteolytic subunit 4, chloroplastic [Theobroma cacao]	26.034	52.37	30.3	9	19	9	1.027	1.02	0.987	0.982	1.01	0.991	0.983	3.18E-01

cds.Camellia_sinensis.comp26669_c0_seq1_m.26830	PREDICTED: ubiquitin carboxyl-terminal hydrolase 12 isoform X2 [Jatropha curcas]	50.13	21.586	24.6	11	12	9	1.005	1.078	1.027	0.997	0.985	0.911	0.93	1.07E-01
cds.Camellia_sinensis.comp26669_c0_seq2_m.41168	PREDICTED: ubiquitin carboxyl-terminal hydrolase 12 isoform X2 [Vitis vinifera]	34.753	3.3538	13.6	4	5	2								
cds.Camellia_sinensis.comp26669_c0_seq3_m.54299	PREDICTED: ubiquitin carboxyl-terminal hydrolase 12 [Beta vulgaris subsp. vulgaris]	13.355	1.9094	23.2	3	4	1	0.946	1.048	0.947	1.114	0.981	0.986	1.048	4.40E-01
cds.Camellia_sinensis.comp26675_c0_seq1_m.28276	PREDICTED: translation initiation factor eIF-2B subunit alpha-like [Nicotiana tabacum]	44.337	18.744	18.5	6	7	6	0.897	0.935	0.969	1.139	1.036	1.064	1.156	1.57E-02

cds.Camellia_sinensis.comp26676_c0_seq2_m.53628	PREDICTED: ER membrane protein complex subunit 10 [Gossypium raimondii]	16.642	2.4278	6.6	2	2	2	0.974	1.044	0.978	0.992	1.039	0.985	1.007	8.18E-01
cds.Camellia_sinensis.comp26677_c1_seq1_m.34526	PREDICTED: cysteine proteinase inhibitor 6 [Theobroma cacao]	27.371	40.686	31.6	6	9	6	1.001	0.995	0.963	1	1.036	1.03	1.036	9.41E-02
cds.Camellia_sinensis.comp26685_c0_seq3_m.18193	PREDICTED: asparagine--tRNA ligase, chloroplastic/mitochondrial isoform X1 [Vitis vinifera]	46.624	1.9691	2.1	1	2	1	0.953	0.941	1.027	1.036	1.042	1.024	1.062	9.35E-02
cds.Camellia_sinensis.comp26685_c0_seq3_m.18196	PREDICTED: asparagine--tRNA ligase, chloroplastic/mitochondrial-like [Ipomoea nil]	11.378	13.148	40.4	4	9	3	0.953	0.957	0.985	1.022	1.022	1.028	1.061	4.90E-03

cds.Camellia_sinensis.comp26692_c0_seq1_m.29062	PREDICTED: ACT domain-containing protein ACR11-like [Daucus carota subsp. sativus]	31.506	97.028	31.3	9	32	9	1.009	0.994	0.985	1.01	1.016	0.982	1.007	6.28E-01
cds.Camellia_sinensis.comp26701_c0_seq2_m.39566	PREDICTED: universal stress protein PHOS34 [Eucalyptus grandis]	18.265	28.105	14.1	2	4	2	1.005	1.048	1.083	0.943	0.975	1.021	0.937	1.09E-01
cds.Camellia_sinensis.comp26706_c0_seq1_m.26738	esterase/lipase/thioesterase family protein [Populus trichocarpa]	44.093	4.2696	5.3	2	2	2	1.039	0.978	1.006	1.027	0.985	0.971	0.987	6.13E-01
cds.Camellia_sinensis.comp26707_c0_seq1_m.474	PREDICTED: pre-mRNA-processing-splicing factor 8A [Theobroma cacao]	268.39	25.359	3.9	9	10	9	1.162	1.193	1.139	0.882	0.833	0.843	0.732	1.42E-04

cds.Camellia_sinensis.comp26709_c0_seq1_m.43631	PREDICTED: non-specific lipid-transfer protein 1-like [Pyrus x bretschneideri]	12.235	1.3564	7.6	1	1	1	0.946	1.007	1.001	1.043	0.999	1.023	1.038	1.89E-01
cds.Camellia_sinensis.comp26709_c0_seq2_m.48095	PREDICTED: non-specific lipid-transfer protein 1-like [Pyrus x bretschneideri]	12.209	6.9332	28.8	3	6	3	0.824	0.811	0.787	1.247	1.173	1.205	1.497	5.73E-05
cds.Camellia_sinensis.comp26712_c0_seq1_m.45153	PREDICTED: NHP2-like protein 1 [Prunus mume]	16.063	30.39	26.5	3	6	3	0.973	0.994	0.982	0.996	1.028	1.053	1.043	7.05E-02
cds.Camellia_sinensis.comp26713_c0_seq1_m.41573	PREDICTED: probable NAD(P)H dehydrogenase (quinone) FQR1-like 1 [Vigna radiata var. radiata]	21.664	47.978	46.3	8	17	3	1.053	1.024	1.047	0.912	0.988	1.067	0.95	3.10E-01

cds.Camellia_sinensis.comp26714_c0_seq2_m.30909	PREDICTED: peptidyl-tRNA hydrolase 2, mitochondrial isoform X2 [Ipomoea	19.996	1.4244	9.4	2	2	2	1.034	1.046	1.006	1.071	0.901	0.946	0.946	3.29E-01
cds.Camellia_sinensis.comp26720_c0_seq1_m.28521	PREDICTED: probable fructokinase-7 [Prunus mume]	41.129	7.6147	13.2	5	8	4	0.874	1.088	1.05		0.856	1.05	0.949	6.80E-01
cds.Camellia_sinensis.comp26723_c0_seq1_m.17388	PREDICTED: 11-oxo-beta-amyrin 30-oxidase isoform X2 [Vitis vinifera]	60.915	5.9051	3.5	2	2	2	0.931	0.957	0.922	1.053	1.067	1.1	1.146	1.34E-03
cds.Camellia_sinensis.comp26729_c0_seq1_m.8066	PREDICTED: beta-amyrin synthase [Sesamum indicum]	87.518	16.013	5.9	4	4	4	0.92	0.931	1.032	1.046	1.061	1.089	1.109	5.30E-02

cds.Camellia_sinensis.comp26732_c0_seq1_m.22473	PREDICTED: tetratricopeptide repeat protein 38-like isoform X3 [Gossypium raimondii]	53.338	1.649	4.9	2	2	2	0.981	1.013	0.853	1.051	1.071	1.051	1.115	1.72E-01
cds.Camellia_sinensis.comp26744_c0_seq1_m.29380	PREDICTED: thioredoxin reductase 1-like [Nelumbo	36.433	108.08	26.5	7	14	7	0.99	0.935	0.981	1.028	1.026	1.033	1.062	7.65E-02
cds.Camellia_sinensis.comp26747_c0_seq1_m.38478	PREDICTED: arogenate dehydrogenase 1, chloroplastic isoform X1 [Vitis vinifera]	31.278	41.475	16.7	5	6	5	0.818	0.848	0.827	1.224	1.13	1.184	1.419	1.62E-04
cds.Camellia_sinensis.comp26749_c0_seq1_m.45343	PREDICTED: myosin-5-like [Glycine max]	15.088	12.69	27.3	4	19	4	1.081	1.059	1.057	0.889	0.919	0.95	0.863	1.94E-03

cds.Camellia_sinensis.comp26757_c0_seq1_m.10442	PREDICTED: probable inactive serine/threonine-protein kinase scy1 [Ziziphus jujuba]	89.205	12.923	2.7	1	1	1	0.868	1.012	1.018	1.257	1.014	0.879	1.087	5.53E-01
cds.Camellia_sinensis.comp26760_c0_seq1_m.52107	PREDICTED: pectinesterase-like [Eucalyptus grandis]	12.065	3.3867	22.9	2	2	2	0.977	1.007	0.986	1.037	1.065	0.945	1.026	5.49E-01
cds.Camellia_sinensis.comp26767_c0_seq1_m.54690	PREDICTED: adenosylhomocysteinase-like [Phoenix dactylifera]	15.401	10.569	25	3	9	1								
cds.Camellia_sinensis.comp26768_c0_seq1_m.36038	PREDICTED: carbonic anhydrase 2 isoform X1 [Ricinus communis]	31.143	100.27	57.1	13	36	11	0.95	0.947	0.976	1.006	1.017	1.037	1.065	8.46E-03
cds.Camellia_sinensis.comp26774_c0_seq1_m.44152	PREDICTED: sm-like protein LSM2 [Vitis vinifera]	14.696	7.3939	10.2	1	1	1								

cds.Camellia_sinensis.comp26781_c0_seq1_m.31960	PREDICTED: methionine aminopeptidase 1B, chloroplastic [Juglans regia]	40.922	24.306	15.2	7	10	7	1.058	1.119	1.097	0.932	0.892	0.914	0.836	9.99E-04
cds.Camellia_sinensis.comp26783_c0_seq1_m.33779	PREDICTED: translin-associated protein X [Nelumbo nucifera]	32.049	1.8397	3.2	1	1	1	0.991	0.865	1.019	1.046	1.043	1.058	1.095	2.09E-01
cds.Camellia_sinensis.comp26785_c0_seq1_m.34625	NAD-dependent epimerase/dehydratase family protein [Medicago truncatula]	31.725	104.5	24.3	6	15	6	0.957	0.895	0.906	1.076	1.089	1.099	1.183	1.42E-03
cds.Camellia_sinensis.comp26789_c0_seq2_m.42230	PREDICTED: universal stress protein A-like protein [Vitis vinifera]	17.846	9.5695	19.9	3	5	3	0.978	0.97	0.926	1.079	1.031	1.107	1.119	1.38E-02

cds.Camellia_sinensis.comp26795_c0_seq1_m.42493	PREDICTED: protein CURVATURE THYLAKOID 1C, chloroplastic [Eucalyptus grandis]	16.607	61.411	18.4	3	8	3	0.948	0.971	0.907	1.093	1.026	1.012	1.108	2.99E-02
cds.Camellia_sinensis.comp26800_c0_seq1_m.39501	PREDICTED: thioredoxin [Vitis vinifera]	22.95	75.526	25.2	5	21	4	1.013	1.057	1.006	0.974	0.951	0.969	0.941	2.36E-02
cds.Camellia_sinensis.comp26812_c0_seq1_m.34139	PREDICTED: transmembrane ascorbate ferrireductase 1 [Sesamum indicum]	25.265	34.592	4.8	1	4	1	0.834	0.748	0.738	1.216	1.26	1.272	1.616	3.02E-04
cds.Camellia_sinensis.comp26815_c0_seq1_m.28445	PREDICTED: peroxidase 3-like [Nelumbo nucifera]	35.276	83.046	36.5	11	19	11	1	1.012	1.014	0.979	0.997	0.995	0.982	6.38E-02
cds.Camellia_sinensis.comp26817_c0_seq1_m.39868	PREDICTED: outer envelope pore protein 21, chloroplastic-like [Juglans regia]	19.349	30.634	25.1	3	4	3	1.007	1.019	0.982	1.006	0.974	0.884	0.952	2.79E-01

cds.Camellia_sinensis.comp26819_c0_seq1_m.6333	PREDICTED: alpha-amylase 3, chloroplastic [Vitis vinifera]	102.14	72.676	19.9	17	25	17	1.068	1.049	1.072	0.898	0.939	0.951	0.874	1.94E-03
cds.Camellia_sinensis.comp26828_c0_seq1_m.56452	PREDICTED: beta-galactosidase 1-like isoform X3 [Eucalyptus grandis]	12.448	1.3163	7.3	1	2	1	0.73	0.768	0.694	1.271	1.311	1.322	1.781	5.63E-05
cds.Camellia_sinensis.comp26832_c0_seq1_m.42570	PREDICTED: cytochrome b5 [Nicotiana attenuata]	15.231	2.5349	35.8	3	6	1	0.97	0.969	1.103	0.972	0.992	1.004	0.976	6.31E-01
cds.Camellia_sinensis.comp26832_c0_seq2_m.43366	PREDICTED: cytochrome b5 [Nicotiana attenuata]	15.213	31.552	44	5	10	3	1.005	0.968	1.014	1.008	1.047	1.016	1.028	2.04E-01
cds.Camellia_sinensis.comp26838_c0_seq2_m.16845	FAD/NAD(P)-binding oxidoreductase family protein [Arabidopsis thaliana]	54.792	1.2285	3	1	1	1								

cds.Camellia_sinensis.comp26839_c0_seq3_m.11391	FG-GAP repeat-containing family protein [Populus trichocarpa]	74.282	6.56	4	3	3	3	1.015	1.001	1.019	0.923	1.051	0.993	0.978	5.91E-01
cds.Camellia_sinensis.comp26850_c0_seq1_m.10545	PREDICTED: SUMO-activating enzyme subunit 2 [Prunus mume]	72.285	49.95	11	7	8	7	0.941	0.958	0.947	1.08	1.067	1.035	1.118	1.24E-03
cds.Camellia_sinensis.comp26854_c0_seq2_m.50755	PREDICTED: NAD-dependent malic enzyme 59 kDa isoform, mitochondrial [Sesamum indicum]	11.659	5.1399	19.5	3	5	2	0.987	0.922	1.014	1.015	1.037	1.042	1.059	1.22E-01
cds.Camellia_sinensis.comp26865_c0_seq1_m.29633	PREDICTED: ycf54-like protein [Nicotiana tabacum]	25.386	107.35	49.6	9	21	9	1.092	1.031	1.058	0.939	0.901	0.926	0.87	2.46E-03

cds.Camellia_sinensis.comp26866_c0_seq1_m.30905	PREDICTED: soluble inorganic pyrophosphatase 6, chloroplastic-like [Nicotiana attenuata]	33.053	157.73	38.2	11	33	11	1.031	1.03	1.034	0.963	0.966	0.977	0.939	1.58E-04
cds.Camellia_sinensis.comp26867_c0_seq1_m.23170	PREDICTED: fructose-1,6-bisphosphatase, chloroplastic [Glycine max]	45.155	247.91	39.1	16	42	16	1.005	0.945	0.946	1.022	1.058	1.02	1.07	4.41E-02
cds.Camellia_sinensis.comp26874_c0_seq2_m.34359	PREDICTED: binding partner of ACD11 1-like [Nicotiana tabacum]	27.172	48.964	31.6	8	15	5	0.93	1.005	0.988	1.021	1.023	1.073	1.066	8.69E-02
cds.Camellia_sinensis.comp26874_c0_seq3_m.40819	PREDICTED: binding partner of ACD11 1 [Vitis vinifera]	23.873	44.805	30.2	6	8	5	0.983	1.028	1.002	1.033	0.996	1.011	1.009	6.20E-01

cds.Camellia_sinensis.comp26887_c0_seq1_m.34748	PREDICTED: neurofilament heavy polypeptide [Vitis vinifera]	29.224	40.323	27.8	6	9	6	1.203	1.22	1.195	0.795	0.754	0.794	0.648	1.97E-05
cds.Camellia_sinensis.comp26888_c0_seq1_m.47435	PREDICTED: endochitinase EP3 [Daucus carota subsp. sativus]	16.327	13.09	19.5	5	8	5	0.952	0.951	0.944	1.063	1.07	1.028	1.11	1.18E-03
cds.Camellia_sinensis.comp26892_c0_seq1_m.37174	PREDICTED: ras-related protein RABB1b [Ricinus communis]	23.346	21.749	30.8	5	9	3	1.003	0.939	1.067	0.981	1.033	1.036	1.014	7.40E-01
cds.Camellia_sinensis.comp26895_c0_seq1_m.15701	PREDICTED: polyadenylate-binding protein 2-like [Jatropha	63.383	53.805	19.5	8	10	7	0.939	1.038	0.932	1.004	0.986	1.091	1.059	2.84E-01

cds.Camellia_sinensis.comp26900_c0_seq1_m.38174	PREDICTED: histidine-containing phosphotransfer protein 1-like isoform X1 [Erythranthe guttata]	19.911	6.3023	13.2	2	3	2	0.8	1.147	0.835	1.126	1.017	1.118	1.172	2.22E-01
cds.Camellia_sinensis.comp26907_c0_seq5_m.36751	plasma membrane fusion protein [Arabidopsis thaliana]	25.319	47.2	30.7	5	12	5	0.946	1.031	1.072	1.028	1.016	0.972	0.989	8.21E-01
cds.Camellia_sinensis.comp26914_c0_seq1_m.26134	PREDICTED: peroxisomal and mitochondrial division factor 2 [Daucus carota subsp. sativus]	47.848	34.428	25.5	13	15	13	0.977	0.978	0.979	1.032	0.954	1.05	1.035	3.76E-01
cds.Camellia_sinensis.comp26921_c1_seq1_m.42103	PREDICTED: cytochrome b-c1 complex subunit 7-2 isoform X1 [Vitis vinifera]	14.728	26.058	42.3	4	12	4	0.96	0.989	0.956	1.008	1.024	1.013	1.048	1.55E-02

cds.Camellia_sinensis.comp26923_c0_seq1_m.22885	PREDICTED: vacuolar protein sorting-associated protein 9A isoform X1 [Vitis vinifera]	53.125	5.3548	3.8	1	1	1	0.929	1.107	0.833	1.094	1.067	0.997	1.101	3.07E-01
cds.Camellia_sinensis.comp26927_c0_seq1_m.27964	PREDICTED: 4-sulfomuconolactone hydrolase [Prunus mume]	36.974	5.0534	10.8	4	4	4	0.996	1.026	1.006	0.957	0.998	1.022	0.983	4.59E-01
cds.Camellia_sinensis.comp26935_c0_seq3_m.37978	PREDICTED: monothiol glutaredoxin-S7, chloroplastic [Populus euphratica]	19.607	16.85	25	3	6	3	0.975	0.978	1.036	1.032	0.969	1.006	1.006	8.33E-01
cds.Camellia_sinensis.comp26950_c0_seq5_m.39635	acetyltransferase (GNAT) domain protein [Medicago truncatula]	20.016	1.3934	4.6	1	1	1	1.08	1.105	1.056	0.985	0.887	0.874	0.847	1.38E-02
cds.Camellia_sinensis.comp26957_c0_seq1_m.38574	transmembrane protein [Arabidopsis thaliana]	23.198	4.0528	8.1	2	4	2	1.002	0.997	0.956	1.014	0.987	1.055	1.034	2.43E-01

cds.Camellia_sinensis.comp26961_c0_seq2_m.46775	PREDICTED: superoxide dismutase [Cu-Zn] 2 [Eucalyptus grandis]	16.883	30.185	47.9	7	12	7	0.994	0.952	0.957	1.042	1.022	1.064	1.078	1.40E-02
cds.Camellia_sinensis.comp26963_c0_seq1_m.25102	PREDICTED: protein RETICULATA-RELATED 3, chloroplastic-like [Pyrus x bretschneideri]	24.801	6.4192	14.2	3	3	3	0.989	1.052	1.044	0.942	0.963	1.011	0.945	1.19E-01
cds.Camellia_sinensis.comp26967_c0_seq1_m.16638	PREDICTED: citrate synthase, mitochondrial [Ipomoea nil]	52.713	77.626	26.1	12	23	12	1.028	1.034	1.005	0.98	0.986	0.983	0.962	1.15E-02
cds.Camellia_sinensis.comp26973_c0_seq1_m.39125	PREDICTED: CBL-interacting serine/threonine-protein kinase 23 isoform X2 [Ziziphus jujuba]	14.419	1.5675	6.2	1	1	1	1.037	1.055	1.076	0.959	0.967	0.9	0.892	1.02E-02

cds.Camellia_sinensis.comp26976_c0_seq1_m.27661	PREDICTED: translation initiation factor IF-3 [Ricinus communis]	31.233	28.789	13.9	3	6	3	1.234	1.202	1.17	0.792	0.79	0.772	0.653	1.82E-05
cds.Camellia_sinensis.comp26980_c0_seq1_m.12038	PREDICTED: transmembrane 9 superfamily member 8-like [Solanum tuberosum	74.085	14.942	5.6	4	4	4	1.035	1.029	1.049	0.946	0.971	0.891	0.902	1.64E-02
cds.Camellia_sinensis.comp26983_c0_seq12_m.33669	PREDICTED: THO complex subunit 4A-like [Juglans regia]	28.209	15.891	24.8	8	13	1	1.134	1.08	1.206	0.834	0.851	0.859	0.744	8.78E-04
cds.Camellia_sinensis.comp26983_c0_seq13_m.33647	PREDICTED: THO complex subunit 4A-like isoform X1 [Nelumbo nucifera]	28.224	4.6827	25	8	12	2	1.054	1.095	1.07	0.847	0.94	0.976	0.858	2.44E-02
cds.Camellia_sinensis.comp26983_c0_seq15_m.36240	PREDICTED: THO complex subunit 4B-like [Juglans regia]	28.281	2.4998	8	2	2	1	1.007	0.996	0.902	0.95	1.039	1.114	1.068	3.24E-01

cds.Camellia_sinensis.comp26983_c0_seq93_m.28144	PREDICTED: THO complex subunit 4D [Solanum pennellii]	22.581	6.3836	14.7	3	4	3	1.156	1.179	1.26	0.781	0.879	0.697	0.656	4.06E-03
cds.Camellia_sinensis.comp26989_c0_seq19_c0_seq1_m.41111	PREDICTED: putative L-ascorbate peroxidase 6 isoform X1 [Vitis vinifera]	17.682	17.501	39.5	6	10	6	0.979	0.968	1.014	1.036	1.031	1.005	1.037	9.38E-02
cds.Camellia_sinensis.comp26991_c0_seq1_m.16685	PREDICTED: signal recognition particle 54 kDa protein 2 [Theobroma cacao]	55.31	21.603	15.2	6	7	6	0.978	1.007	1.047	0.99	1.02	1	0.993	7.63E-01
cds.Camellia_sinensis.comp26996_c0_seq1_m.34338	PREDICTED: THO complex subunit 7A [Sesamum indicum]	27.826	3.4659	9.2	2	2	2	0.97	1.059	1.032	1.008	0.895	1.042	0.962	4.92E-01
cds.Camellia_sinensis.comp27000_c0_seq1_m.42120	PREDICTED: mitochondrial acidic protein MAM33 isoform X2 [Solanum tuberosum	25.95	2.3507	5.2	1	1	1								

cds.Camellia_sinensis.comp27010_c0_seq1_m.9726	PREDICTED: putative beta-D-xylosidase [Jatropha curcas]	83.377	148.26	26.1	16	42	16	0.922	0.934	0.918	1.074	1.051	1.093	1.16	2.83E-04
cds.Camellia_sinensis.comp27016_c0_seq1_m.34053		26.014	26.705	14	3	6	3	1.129	1.104	1.004	0.881	0.879	0.91	0.825	7.08E-03
cds.Camellia_sinensis.comp27021_c0_seq1_m.30589	uncharacterized LOC100274051 [Zea mays]	21.662	11.98	19.6	3	6	3	0.925	0.999	0.96	0.956	1.115	1.066	1.088	1.80E-01
cds.Camellia_sinensis.comp27022_c0_seq1_m.35616	PREDICTED: B-cell receptor-associated protein 31-like [Nicotiana tabacum]	24.807	35.457	14.4	3	9	3	1.005	1.004	0.99	1.004	0.969	1.023	0.999	9.45E-01
cds.Camellia_sinensis.comp27034_c0_seq1_m.33320	PREDICTED: dihydrofolate reductase [Vitis vinifera]	32.39	61.654	9.1	2	3	2	1.401	1.392	1.43	0.561	0.682	0.507	0.414	9.54E-03

cds.Camellia_sinensis.comp27037_c0_seq1_m.58749	PREDICTED: putative quinone-oxidoreductase homolog, chloroplastic [Prunus mume]	11.67	1.4625	16.3	2	2	2								
cds.Camellia_sinensis.comp27042_c0_seq1_m.32393	heme oxygenase 1, chloroplastic-like [Nicotiana tabacum]	33.028	29.896	25.1	7	8	4	1.039	0.976	1.023	0.984	0.991	0.975	0.971	2.08E-01
cds.Camellia_sinensis.comp27042_c0_seq2_m.32684	heme oxygenase 1, chloroplastic-like [Nicotiana tabacum]	31.142	1.9489	16.2	5	5	2								
cds.Camellia_sinensis.comp27045_c0_seq1_m.3580	PREDICTED: nuclear pore complex protein NUP96 [Vitis vinifera]	118.16	1.225	2.8	3	3	2								
cds.Camellia_sinensis.comp27046_c0_seq1_m.56718	PREDICTED: nitrilase-like protein 2 [Ricinus communis]	13.564	1.2095	30.6	3	4	1	0.953	1.033	1.073	0.961	0.997	0.992	0.964	3.93E-01

cds.Camellia_sinensis.comp27049_c0_seq1_m.21867	PREDICTED: protein disulfide-isomerase 5-2 [Ricinus communis]	48.982	32.986	24.1	11	14	11	0.946	0.949	0.976	1.035	1.074	1.056	1.102	2.60E-03
cds.Camellia_sinensis.comp27051_c0_seq1_m.17273	PREDICTED: anthranilate synthase alpha subunit 2, chloroplastic-like isoform X2 [Nicotiana tabacum]	65.075	190.48	45.6	23	48	23	1.013	1.013	1	0.982	0.984	0.988	0.976	6.70E-03
cds.Camellia_sinensis.comp27055_c0_seq1_m.10230	PREDICTED: coronatine-insensitive protein 1 [Theobroma cacao]	66.812	12.836	11.3	6	7	6	0.87	0.958	1.015	1.02	1.042	1.084	1.107	1.00E-01
cds.Camellia_sinensis.comp27058_c0_seq2_m.48495	PREDICTED: LOW QUALITY PROTEIN: auxilin-related protein 1-like [Malus domestica]	12.134	1.7325	9.5	1	3	1	1.021	0.923	0.953	1.014	1.1	1.044	1.09	8.56E-02

cds.Camellia_sinensis.comp27064_c0_seq1_m.17481	PREDICTED: putative clathrin assembly protein At2g01600 [Nelumbo	62.405	5.1308	8.9	5	5	4	1.05	0.979	0.96	1.092	0.942	0.985	1.01	8.73E-01
cds.Camellia_sinensis.comp27065_c0_seq2_m.4908	PREDICTED: DUF724 domain-containing protein 6-like isoform X1 [Ipomoea nil]	107.66	3.7258	2.4	2	2	2	1.266	1.394	1.269	0.768	0.586	0.631	0.505	1.36E-03
cds.Camellia_sinensis.comp27076_c0_seq1_m.35798	PREDICTED: electron transfer flavoprotein subunit beta, mitochondrial [Ziziphus jujuba]	27.725	9.3105	18.3	5	6	5	0.965	0.978	1.006	0.993	1.04	1.023	1.036	1.23E-01
cds.Camellia_sinensis.comp27084_c0_seq1_m.9619	PREDICTED: KH domain-containing protein At4g18375-like [Glycine max]	30.529	8.7639	6.2	1	1	1								

cds.Camellia_sinensis.comp27087_c0_seq1_m.46535	PREDICTED: UPF0664 stress-induced protein C29B12.11c [Nelumbo nucifera]	23.128	1.9467	12.3	2	2	2	0.87	0.884	0.917	1.307	1.02	1.061	1.268	4.17E-02
cds.Camellia_sinensis.comp27088_c0_seq1_m.12753	PREDICTED: probable Xaa-Pro aminopeptidase P isoform X1 [Jatropha curcas]	72.33	43.018	18.3	10	15	10	0.97	1.005	0.992	1.009	1.012	1.008	1.021	1.84E-01
cds.Camellia_sinensis.comp27093_c0_seq2_m.50004	PREDICTED: non-specific lipid transfer protein GPI-anchored 2 [Vitis vinifera]	12.438	2.2611	15	1	1	1								
cds.Camellia_sinensis.comp27102_c0_seq1_m.14624	PREDICTED: T-complex protein 1 subunit epsilon [Nicotiana tabacum]	59.216	55.034	27.1	15	23	15	0.933	0.937	0.926	1.065	1.072	1.054	1.141	2.27E-05

cds.Camellia_sinensis.comp27106_c0_seq1_m.24540	Urease accessory protein UreH [Morus notabilis]	39.907	10.675	3.2	1	2	1	0.968	1.035	1.088	0.997	0.89	1.029	0.943	3.48E-01
cds.Camellia_sinensis.comp27110_c0_seq1_m.8651	PREDICTED: conserved oligomeric Golgi complex subunit 7 [Theobroma cacao]	92.826	6.2268	3.6	3	3	3	1.046	0.962	1.029	1.042	0.958	0.968	0.977	5.66E-01
cds.Camellia_sinensis.comp27112_c0_seq1_m.40457	PREDICTED: inactive protein RESTRICTED TEV MOVEMENT 2-like [Daucus carota subsp. sativus]	20.996	11.634	11.1	2	2	2	0.995	1.005	0.993	0.951	1.055	1.008	1.007	8.59E-01
cds.Camellia_sinensis.comp27114_c0_seq1_m.26893	PREDICTED: GDSL esterase/lipase At3g26430-like [Juglans regia]	46.596	2.4876	4	2	2	2	0.925	0.988	0.984	1.049	1.017	1.063	1.08	3.66E-02

cds.Camellia_sinensis.comp27117_c0_seq1_m.17061	PREDICTED: monodehydroascorbate reductase isoform X1 [Vitis vinifera]	47.223	214.76	54.6	22	77	21	0.937	0.907	0.932	1.071	1.113	1.082	1.177	4.42E-04
cds.Camellia_sinensis.comp27120_c0_seq1_m.24219	PREDICTED: yrdC domain-containing protein, mitochondrial isoform X1 [Ricinus	31.143	3.5953	8.7	2	2	2	0.953	0.977	1.138	1.004	1.036	0.908	0.961	6.04E-01
cds.Camellia_sinensis.comp27125_c0_seq4_m.4548	PREDICTED: phytochrome E isoform X4 [Jatropha curcas]	62.441	4.3643	4.3	3	3	3	0.975	0.901	1.349	0.893	0.868	1.01	0.859	3.54E-01
cds.Camellia_sinensis.comp27125_c0_seq4_m.4549	PREDICTED: phytochrome E-like [Juglans regia]	26.036	2.4561	6.1	1	1	1								

cds.Camellia_sinensis.comp27127_c0_seq1_m.44386	PREDICTED: 3-ketoacyl-CoA synthase 11 isoform X2 [Gossypium raimondii]	24.21	1.1574	3.8	1	1	1								
cds.Camellia_sinensis.comp27140_c0_seq1_m.23629	PREDICTED: anthocyanidin 3-O-glucosyltransferase 7 [Vitis vinifera]	50.677	82.478	25	10	18	10	1.134	1.114	1.12	0.837	0.859	0.885	0.766	9.79E-05
cds.Camellia_sinensis.comp27153_c0_seq1_m.21972	PREDICTED: structural maintenance of chromosomes protein 2-1-like [Ziziphus jujuba]	42.454	40.153	22	7	10	2	1.04	1.067	1.099	0.912	0.946	0.926	0.868	1.82E-03
cds.Camellia_sinensis.comp27154_c0_seq1_m.24675	PREDICTED: probable xyloglucan endotransglucosylase/hydrolase protein B [Populus euphratica]	35.618	2.2642	10.4	3	3	2	0.917	1.087	0.855	1.051	1.023	1.091	1.107	2.22E-01

cds.Camellia_sinensis.comp27157_c0_seq1_m.20519	PREDICTED: putative GEM-like protein 3 [Erythranthe guttata]	46.435	1.5661	2.1	1	1	1	1.059	1.019	1.02	0.933	1.003	0.96	0.935	5.10E-02
cds.Camellia_sinensis.comp27164_c0_seq1_m.29784	PREDICTED: hypersensitive-induced response protein 2 [Solanum tuberosum	31.467	86.661	43.7	12	33	9	0.927	0.915	0.898	1.104	1.091	1.084	1.197	7.64E-05
cds.Camellia_sinensis.comp27166_c0_seq1_m.22873	PREDICTED: U5 small nuclear ribonucleoprotein 40 kDa protein [Ziziphus jujuba]	39.294	13.206	12.1	4	4	4	1.016	1.005	1.01	0.994	1.01	1.02	0.998	7.87E-01
cds.Camellia_sinensis.comp27168_c0_seq1_m.28325	PREDICTED: SUMO-activating enzyme subunit 1B-1 isoform X1 [Vitis vinifera]	36.092	19.113	24.8	7	11	7	0.972	0.944	0.962	1.016	1.058	1.073	1.093	8.46E-03

cds.Camellia_sinensis.comp27172_c0_seq1_m.28250	PREDICTED: protein phosphatase methylesterase 1 [Sesamum indicum]	42.216	8.1527	7.4	3	3	3	0.926	1.074	1.021	1.032	1.044	0.923	0.993	9.11E-01
cds.Camellia_sinensis.comp27178_c0_seq1_m.9522	PREDICTED: peroxisomal fatty acid beta-oxidation multifunctional protein AIM1 [Theobroma cacao]	78.867	84.702	22.7	17	23	17	0.933	0.941	0.944	1.07	1.06	1.081	1.139	4.00E-05
cds.Camellia_sinensis.comp27182_c0_seq1_m.41959	PREDICTED: peptidyl-prolyl cis-trans isomerase CYP19-3 [Ziziphus jujuba]	22.574	4.7934	17.9	5	10	3	1.01	0.99	0.985	1.019	1.063	0.94	1.012	7.79E-01
cds.Camellia_sinensis.comp27190_c0_seq1_m.27944	PREDICTED: 60S ribosomal protein L13a-4 [Sesamum indicum]	23.641	14.441	27.7	6	9	2	1.094	1.124	1.142	0.87	0.869	0.873	0.777	2.50E-03

cds.Camellia_sinensis.comp27190_c0_seq2_m.27972	PREDICTED: 60S ribosomal protein L13a-4 [Sesamum indicum]	29.869	1.7124	18.8	5	8	1	1.209	1.188	1.133	0.771	0.818	0.824	0.684	1.83E-04
cds.Camellia_sinensis.comp27196_c0_seq1_m.30399	PREDICTED: ubiquitin carboxyl-terminal hydrolase 2 [Vitis vinifera]	28.068	38.259	14.4	3	4	3	0.99	0.944	0.963	1.075	1.013	1.039	1.079	2.58E-02
cds.Camellia_sinensis.comp27198_c0_seq1_m.23934	PREDICTED: obg-like ATPase 1 [Vigna radiata var. radiata]	29.577	54.091	50	12	19	12	1.004	0.999	1.03	1.016	0.979	0.996	0.986	3.84E-01
cds.Camellia_sinensis.comp27198_c0_seq1_m.23935	PREDICTED: obg-like ATPase 1 [Prunus mume]	18.39	44.921	42.9	7	10	7	1.046	1.04	1.053	0.975	0.971	0.941	0.92	2.06E-03
cds.Camellia_sinensis.comp27203_c0_seq1_m.29469	PREDICTED: disease resistance RPP13-like protein 4 [Capsicum annuum]	48.541	2.4185	6.6	3	3	3								

cds.Camellia_sinensis.comp27207_c0_seq1_m.37062	PREDICTED: vesicle transport v-SNARE 13 [Vitis vinifera]	24.858	23.796	32.1	6	7	6	1.012	1.051	1.007	0.989	0.967	0.978	0.956	3.94E-02
cds.Camellia_sinensis.comp27211_c0_seq2_m.37491	50S ribosomal protein L10, chloroplastic [Nicotiana tabacum]	11.866	24.576	51.4	6	15	6	1.022	1.02	0.976	0.968	1.011	0.994	0.985	4.89E-01
cds.Camellia_sinensis.comp27212_c0_seq1_m.12901	PREDICTED: delta-1-pyrroline-5-carboxylate dehydrogenase 12A1, mitochondrial isoform	67.784	114.71	33.6	18	29	18	0.941	0.957	0.924	1.047	1.04	1.059	1.115	6.80E-04
cds.Camellia_sinensis.comp27213_c0_seq1_m.21055	PREDICTED: mitochondrial carnitine/acylcarnitine carrier-like protein [Juglans regia]	30.569	54.968	45.8	13	18	13	1.028	1.051	1.03	0.985	0.952	0.941	0.926	7.42E-03

cds.Camellia_sinensis.comp27214_c0_seq5_m.34668	PREDICTED: peptidyl-prolyl cis-trans isomerase FKBP16-1, chloroplastic isoform X2 [Vitis vinifera]	15.426	6.9752	17.3	1	1	1	1.002	0.899	1.018	1.085	1.08	0.939	1.063	3.79E-01
cds.Camellia_sinensis.comp27215_c0_seq1_m.14883	PREDICTED: heterogeneous nuclear ribonucleoprotein Q [Sesamum indicum]	53.327	3.4461	2.3	1	1	1	1.103	1.038	1.079	1.033	0.791	0.943	0.859	1.22E-01
cds.Camellia_sinensis.comp27216_c0_seq1_m.30162	PREDICTED: early nodulin-like protein 2 [Ipomoea nil]	35.952	4.6579	9.5	3	3	3								
cds.Camellia_sinensis.comp27224_c0_seq2_m.34734	PREDICTED: macro domain-containing protein VPA0103-like isoform X3 [Nicotiana tabacum]	30.955	84.034	29.5	6	9	6	1.028	1.019	1.018	0.956	1.085	0.926	0.968	5.47E-01

cds.Camellia_sinensis.comp27225_c0_seq1_m.28449	anthocyanidin reductase [Vitis vinifera]	36.273	62.198	30.3	11	25	6	1.404	1.342	1.365	0.559	0.608	0.576	0.424	3.32E-06
cds.Camellia_sinensis.comp27225_c0_seq2_m.28877	anthocyanidin reductase [Vitis vinifera]	37.459	8.2769	20.5	7	18	2	1.311	1.253	1.313	0.684	0.687	0.663	0.525	2.24E-06
cds.Camellia_sinensis.comp27225_c1_seq1_m.31047	PREDICTED: PLASMODESMATA CALLOSE-BINDING PROTEIN 3 [Vitis vinifera]	20.119	4.4973	5	1	1	1	0.991	0.99	0.976	0.99	1.061	1.005	1.033	2.07E-01
cds.Camellia_sinensis.comp27228_c0_seq2_m.28667	PREDICTED: pentatricopeptide repeat-containing protein At4g36680, mitochondrial [Vitis vinifera]	43.584	3.058	3.1	1	1	1								

cds.Camellia_sinensis.comp27229_c0_seq1_m.34263	PREDICTED: NADH dehydrogenase [ubiquinone] 1 alpha subcomplex subunit 8-B-like [Sesamum indicum]	12.075	1.42	15	2	3	1	0.988	0.898	1.032	1.085	1.033	0.987	1.064	2.70E-01
cds.Camellia_sinensis.comp27229_c0_seq5_m.51936	PREDICTED: NADH dehydrogenase [ubiquinone] 1 alpha subcomplex subunit 8-B-like [Ipomoea nil]	11.971	3.7679	28.3	4	5	3	1.111	1.049	1.091	0.918	0.853	0.955	0.839	8.92E-03
cds.Camellia_sinensis.comp27241_c0_seq1_m.23938	PREDICTED: IAA-amino acid hydrolase ILR1-like 3 [Vitis vinifera]	48.017	8.8507	6.7	3	3	2	0.99	0.914	1.051	1.037	0.962	1.061	1.036	5.16E-01
cds.Camellia_sinensis.comp27255_c0_seq1_m.26215	PREDICTED: GEM-like protein 1 [Theobroma cacao]	37.906	21.443	8	3	4	3	0.974	1.024	0.969	1.017	0.996	1.005	1.017	4.05E-01

cds.Camellia_sinensis.comp27256_c0_seq1_m.18069	PREDICTED: triose phosphate/phosphate translocator, non-green plastid, chloroplastic-like [Nicotiana attenuata]	53.315	6.5504	5.6	2	3	2								
cds.Camellia_sinensis.comp27260_c0_seq2_m.11556	PREDICTED: protein MODIFIER OF SNC1 11 isoform X1 [Vitis vinifera]	22.318	45.149	41.2	6	10	6	1.021	1.05	0.99	1	0.975	1	0.972	2.10E-01
cds.Camellia_sinensis.comp27260_c0_seq3_m.14275	PREDICTED: dihydrolipoyllysine-residue acetyltransferase component 5 of pyruvate dehydrogenase complex,chloroplastic [Vitis vinifera]	49.889	51.545	28.3	11	15	10	0.998	1.017	0.979	1.014	1.011	0.979	1.003	8.43E-01

cds.Camellia_sinensis.comp27269_c0_seq1_m.35694	PREDICTED: thylakoid lumenal 16.5 kDa protein, chloroplastic [Ziziphus jujuba]	25.127	42.701	32.3	6	16	6	1.061	1.02	0.969	0.994	0.953	0.988	0.962	2.66E-01
cds.Camellia_sinensis.comp27274_c0_seq1_m.37043	PREDICTED: RPM1-interacting protein 4-like [Sesamum indicum]	26.671	1.4361	5.3	1	1	1	1.154	0.967	1.008	0.97	0.952	0.934	0.913	1.79E-01
cds.Camellia_sinensis.comp27275_c0_seq2_m.35346	PREDICTED: 60S ribosomal protein L15 [Nicotiana tabacum]	24.139	2.7825	6.9	2	3	2	1.041	1.087	1.091	0.959	0.923	0.9	0.864	3.60E-03
cds.Camellia_sinensis.comp27280_c0_seq1_m.38417	PREDICTED: mitochondrial fission 1 protein A-like [Nicotiana tabacum]	18.391	1.9523	9.4	2	2	2	1.044	1.018	1.06	0.955	0.966	0.954	0.921	2.72E-03
cds.Camellia_sinensis.comp27284_c0_seq1_m.31468	PREDICTED: 60S ribosomal protein L17-2 [Ziziphus jujuba]	24.958	43.431	25.7	5	9	3	1.08	1.096	1.122	0.918	0.932	0.854	0.82	2.40E-03

cds.Camellia_sinensis.comp27284_c0_seq2_m.32028	PREDICTED: 60S ribosomal protein L17-2 [Juglans regia]	20.667	5.7439	21.3	4	7	2	1.074	1.111	1.136	0.891	0.901	0.866	0.8	3.80E-04
cds.Camellia_sinensis.comp27285_c0_seq1_m.35727	putative mannitol dehydrogenase [Morus notabilis]	31.595	16.138	10.5	3	5	3	0.947	0.976	0.941	1.087	1.042	1.041	1.107	5.04E-03
cds.Camellia_sinensis.comp27285_c0_seq3_m.45741	PREDICTED: probable mannitol dehydrogenase [Ricinus communis]	23.235	2.5407	7	2	3	2	0.934	0.942	0.976	1.064	1.073	1.039	1.114	2.88E-03
cds.Camellia_sinensis.comp27288_c0_seq1_m.48383	60S ribosomal protein L37a-2 [Medicago truncatula]	12.721	6.8785	21.2	2	3	2	0.932	1.233	1.076	0.895	0.915	0.943	0.849	1.29E-01
cds.Camellia_sinensis.comp27289_c0_seq2_m.43364		10.996	2.0836	11.9	1	2	1								

cds.Camellia_sinensis.comp27290_c0_seq4_m.33441	PREDICTED: peptidyl-prolyl cis-trans isomerase FKBP16-4, chloroplastic isoform X3 [Vitis vinifera]	24.796	43.753	32.5	7	21	7	1.019	0.977	0.962	0.995	0.979	1.011	1.009	6.59E-01
cds.Camellia_sinensis.comp27292_c0_seq12_m.33110	PREDICTED: 14-3-3 protein 7 [Ricinus communis]	28.896	141.62	60.5	17	48	14	0.977	0.949	0.905	1.04	1.063	1.086	1.126	9.52E-03
cds.Camellia_sinensis.comp27300_c1_seq1_m.38184	PREDICTED: chlorophyll a-b binding protein CP29.1, chloroplastic [Ricinus communis]	24.283	115.72	46.2	10	50	7	1.036	0.955	0.969	0.982	1.034	1.008	1.022	4.96E-01
cds.Camellia_sinensis.comp27300_c1_seq2_m.42952	PREDICTED: chlorophyll a-b binding protein CP29.1, chloroplastic [Ricinus communis]	30.03	74.473	34.8	8	26	5	1.063	1.05	1.013	0.965	0.936	0.985	0.923	1.82E-02

cds.Camellia_sinensis.comp27314_c0_seq1_m.30117	PREDICTED: eukaryotic translation initiation factor 2 subunit alpha-like [Sesamum indicum]	41.484	31.367	14.7	5	6	5	1.008	0.995	1.011	0.86	1.082	1.044	0.991	8.60E-01
cds.Camellia_sinensis.comp27316_c0_seq1_m.17284	PREDICTED: extensin-like [Nicotiana tabacum]	63.117	16.687	11	6	7	6	1.016	1.018	0.957	0.97	0.939	1.028	0.982	6.08E-01
cds.Camellia_sinensis.comp27319_c0_seq1_m.22934	PREDICTED: acetylornithine aminotransferase, mitochondrial-like [Jatropha curcas]	49.89	110.15	31.2	11	18	10	1.007	1.021	1.013	0.957	0.999	0.97	0.962	4.37E-02
cds.Camellia_sinensis.comp27322_c0_seq1_m.25140	PREDICTED: patatin-like protein 2 isoform X2 [Arachis duranensis]	49.106	1.5436	1.6	1	2	1	0.93	0.977	0.966	1.026	1.039	1.086	1.097	1.55E-02

cds.Camellia_sinensis.comp27322_c0_seq2_m.31542	Patatin group A-3 [Morus notabilis]	44.871	21.276	17.2	6	7	6	1.073	0.942	0.948	1.035	1.065	0.946	1.028	6.38E-01
cds.Camellia_sinensis.comp27327_c0_seq1_m.25811	PREDICTED: poly(U)-specific endoribonuclease-B [Vitis vinifera]	51.393	4.3687	5.7	2	2	2								
cds.Camellia_sinensis.comp27330_c0_seq1_m.15051	PREDICTED: protein ASPARTIC PROTEASE IN GUARD CELL 1 [Ricinus communis]	47.863	11.358	10	4	6	4	1.101	1.086	1.071	0.925	0.942	0.94	0.862	1.15E-04
cds.Camellia_sinensis.comp27335_c0_seq1_m.32917	PREDICTED: beta-glucosidase 13 [Vitis vinifera]	42.59	22.017	12.4	5	10	5	1.093	1.083	1.122	0.943	0.886	0.886	0.823	1.12E-03
cds.Camellia_sinensis.comp27336_c0_seq2_m.51245	PREDICTED: ubiquitin carboxyl-terminal hydrolase 12-like [Nicotiana attenuata]	20.875	25.691	33.3	8	8	3	0.989	1.039	0.942	0.992	0.994	1.056	1.024	5.25E-01

cds.Camellia_sinensis.comp27339_c0_seq2_m.35031	PREDICTED: chlorophyll a-b binding protein 13, chloroplastic-like [Nelumbo nucifera]	28.901	23.494	26.1	5	48	3	0.947	0.941	0.969	1.045	1.047	1.02	1.089	2.22E-03
cds.Camellia_sinensis.comp27339_c0_seq4_m.43220	PREDICTED: chlorophyll a-b binding protein 5, chloroplastic [Sesamum indicum]	20.154	58.365	44.4	6	59	2	1.021	0.927	1.019	0.964	1.051	0.98	1.009	8.27E-01
cds.Camellia_sinensis.comp27339_c0_seq6_m.50345	PREDICTED: chlorophyll a-b binding protein of LHCII type 1 [Musa acuminata subsp. malaccensi	11.014	1.5478	75	3	7	1	0.922	1.005	1.007	1.095	0.999	1	1.055	2.76E-01
cds.Camellia_sinensis.comp27341_c0_seq3_m.47505	PREDICTED: autophagy-related protein 8C [Sesamum indicum]	13.707	2.3951	19.3	3	3	3	0.965	0.962	0.942	1.055	1.022	1.076	1.099	4.96E-03

cds.Camellia_sinensis.comp27350_c0_seq1_m.24260	PREDICTED: chalcone synthase 1 [Juglans regia]	48.966	67.923	32.2	11	35	2	1.518	1.391	1.424	0.472	0.54	0.506	0.35	2.17E-05
cds.Camellia_sinensis.comp27350_c0_seq2_m.26012	PREDICTED: chalcone synthase 1 [Juglans regia]	49.033	-2	30.6	10	33	1								
cds.Camellia_sinensis.comp27354_c0_seq3_m.28181	PREDICTED: geranylgeranyl pyrophosphate synthase, chloroplastic-like [Sesamum indicum]	40.703	5.1704	8.8	3	3	3	1.031	1.057	1.072	0.979	0.946	0.911	0.897	1.02E-02
cds.Camellia_sinensis.comp27359_c0_seq1_m.43821	PREDICTED: delta(1)-pyrroline-2-carboxylate reductase [Prunus mume]	21.424	49.743	28.1	5	10	5	0.939	0.953	0.983	1.059	1.069	1.042	1.103	3.16E-03

cds.Camellia_sinensis.comp27362_c0_seq1_m.50066	PREDICTED: pyrophosphate-energized vacuolar membrane proton pump 1-like isoform X1 [Nicotiana sylvestris]	17.116	54.456	44.8	7	15	2	0.969	0.918	1.023	0.97	1.016	1.051	1.044	3.30E-01
cds.Camellia_sinensis.comp27363_c0_seq2_m.28875	PREDICTED: protein FLX-like 4 [Jatropha curcas]	34.278	12.847	22.5	7	10	7	0.958	0.987	0.986	1.015	1.034	1.013	1.045	2.04E-02
cds.Camellia_sinensis.comp27367_c0_seq1_m.14464	PREDICTED: probable inorganic phosphate transporter 1-3 [Sesamum indicum]	59.078	28.096	11.1	6	9	5	1.007	0.983	0.99	0.962	1.015	1.005	1.001	9.81E-01

cds.Camellia_sinensis.comp27369_c0_seq2_m.41742	PREDICTED: NADH dehydrogenase [ubiquinone] iron-sulfur protein 4, mitochondrial-like [Sesamum indicum]	19.867	8.1251	18.9	4	10	4	1.067	1.048	1.031	0.972	0.949	0.936	0.908	2.88E-03
cds.Camellia_sinensis.comp27370_c0_seq1_m.26641	PREDICTED: DNA damage-inducible protein 1 [Pyrus x bretschneideri]	44.15	132.92	44.1	13	17	13	0.958	0.998	0.933	1.049	1.043	1.062	1.092	1.22E-02
cds.Camellia_sinensis.comp27372_c0_seq1_m.36694	PREDICTED: probable carbohydrate esterase At4g34215 [Lupinus angustifolius]	27.091	2.8298	8.5	2	3	2	0.945	0.927	0.973	0.919	1.164	1.093	1.116	2.18E-01

cds.Camellia_sinensis.comp27373_c0_seq1_m.22325	PREDICTED: diphosphomevalonate decarboxylase MVD2-like [Nicotiana attenuata]	46.451	19.857	19.7	9	14	9	0.997	0.964	0.968	1.02	1.03	1.019	1.048	1.37E-02
cds.Camellia_sinensis.comp27376_c0_seq1_m.23506	PREDICTED: adenylyltransferase and sulfurtransferase MOCS3 [Ricinus communis]	55.286	12.52	11	5	6	5	0.966	0.944	0.945	1.056	1.062	1.077	1.119	2.97E-04
cds.Camellia_sinensis.comp27377_c0_seq1_m.43994	PREDICTED: protease Do-like 5, chloroplastic isoform X1 [Nelumbo nucifera]	26.525	35.48	13.5	3	6	3	1.008	0.985	0.955	1.018	1.047	1.029	1.05	5.15E-02
cds.Camellia_sinensis.comp27380_c1_seq1_m.39390	COPI associated protein [Medicago truncatula]	22.395	6.7332	9.1	2	2	2	0.932	0.991	1.012	1.045	1.045	0.999	1.052	1.49E-01

cds.Camellia_sinensis.comp27381_c0_seq1_m.37044	DNA-binding family protein [Populus trichocarpa]	15.279	38.968	34.3	4	8	4	0.959	0.961	0.993	1.026	1.021	1.046	1.062	1.12E-02
cds.Camellia_sinensis.comp27384_c0_seq1_m.32444	PREDICTED: xyloglucan endotransglucosylase/hydrolase protein 9 [Gossypium arboreum]	33.85	4.9624	12.5	4	5	4	1.145	1.211	1.132	0.887	0.77	0.834	0.714	1.82E-03
cds.Camellia_sinensis.comp27385_c1_seq2_m.39231	PREDICTED: ADP-ribosylation factor 1 isoform X1 [Fragaria vesca subsp. vesca]	20.666	80.293	32	6	24	6	1.012	0.994	0.98	1.006	1.008	0.991	1.006	5.83E-01
cds.Camellia_sinensis.comp27386_c1_seq1_m.47223	PREDICTED: 60S acidic ribosomal protein P3-like [Nelumbo nucifera]	12.775	42.232	21.6	2	5	2	1.006	1.061	1.1	0.937	0.981	0.924	0.897	2.69E-02

cds.Camellia_sinensis.comp27397_c0_seq1_m.13150	PREDICTED: clathrin interactor EPSIN 1 [Vitis vinifera]	41.831	6.3554	8.5	2	2	2	1.166	1.098	1.042	0.852	0.853	0.952	0.804	1.12E-02
cds.Camellia_sinensis.comp27397_c0_seq1_m.13151	PREDICTED: clathrin interactor EPSIN 1 isoform X2 [Theobroma cacao]	15.393	2.8204	21.5	3	4	3	1.086	1.064	1.06	0.943	0.904	0.929	0.865	5.57E-04
cds.Camellia_sinensis.comp27402_c0_seq1_m.37995	PREDICTED: membrane steroid-binding protein 2-like [Sesamum indicum]	21.237	5.9064	12.4	2	2	2	1.07	1.159	1.04	0.89	0.898	0.921	0.829	5.34E-03
cds.Camellia_sinensis.comp27422_c0_seq1_m.41621	PREDICTED: purple acid phosphatase 3-like [Ziziphus jujuba]	32.283	80.407	16.6	4	7	4	0.992	1.02	0.957	1.055	0.956	1.007	1.017	6.64E-01
cds.Camellia_sinensis.comp27425_c0_seq2_m.35521	PREDICTED: PHD finger protein Alfin1 [Vitis vinifera]	27.815	1.9488	4	1	1	1								

cds.Camellia_sinensis.comp27426_c0_seq1_m.30120	PREDICTED: ras-related protein RABA1f [Tarenaya hassleriana]	24.061	37.428	42.1	8	17	3	0.977	0.978	1.01	1.039	1.037	1.01	1.041	4.81E-02
cds.Camellia_sinensis.comp27440_c0_seq2_m.52441		14.454	3.4121	8.5	1	1	1	0.995	0.971	0.919	1.028	1.021	1.082	1.085	5.12E-02
cds.Camellia_sinensis.comp27449_c0_seq1_m.35876	PREDICTED: alpha carbonic anhydrase 1, chloroplastic [Prunus mume]	30.787	10.077	9.7	3	5	3	0.927	0.937	0.961	1.024	1.109	1.017	1.115	2.21E-02
cds.Camellia_sinensis.comp27451_c0_seq1_m.19603	C2 domain-containing family protein [Populus trichocarpa]	45.301	6.4934	4.6	2	3	2	0.989	0.93	0.916	1.024	1.097	1.065	1.124	1.91E-02
cds.Camellia_sinensis.comp27452_c0_seq1_m.38379	PREDICTED: acyl carrier protein 1, chloroplastic-like [Citrus sinensis]	19.958	11.939	8.1	1	2	1	1.035	1.178	1.142	0.8	0.903	0.915	0.78	1.27E-02

cds.Camellia_sinensis.comp27462_c0_seq1_m.16642	PREDICTED: protein DEK [Solanum lycopersicum]	31.461	2.6542	4.4	1	1	1	0.956	0.895	1.039	1.081	1.05	1.008	1.086	1.53E-01
cds.Camellia_sinensis.comp27462_c0_seq1_m.16643	PREDICTED: DNA ligase 1 [Theobroma cacao]	25.547	-2	3.5	1	1	1								
cds.Camellia_sinensis.comp27465_c0_seq4_m.42355	PREDICTED: 2-phytyl-1,4-beta-naphthoquinone methyltransferase, chloroplastic isoform X2 [Citrus sinensis]	14.761	2.5349	12.7	2	3	2								
cds.Camellia_sinensis.comp27469_c0_seq1_m.34207	cupin family protein [Medicago truncatula]	22.025	28.122	19.7	3	7	3	1.034	1.013	0.959	0.98	1.003	1.005	0.994	8.27E-01
cds.Camellia_sinensis.comp27470_c0_seq2_m.29756	plasma protein [Arabidopsis thaliana]	21.261	1.8331	10.9	2	2	2	0.903	0.888	1.055	1.091	1.007	1.092	1.121	1.29E-01

cds.Camellia_sinensis.comp27471_c0_seq1_m.27836	PREDICTED: probable plastid-lipid-associated protein 3, chloroplastic [Nelumbo nucifera]	42.804	41.71	26.3	8	16	7	1.092	1.141	1.127	0.883	0.815	0.867	0.763	6.02E-04
cds.Camellia_sinensis.comp27471_c0_seq2_m.31372	PREDICTED: plastoglobulin-1, chloroplastic-like [Ziziphus jujuba]	39.315	207.37	41.1	12	34	11	0.954	0.962	0.947	1.052	1.062	1.043	1.103	1.43E-04
cds.Camellia_sinensis.comp27478_c0_seq1_m.19271	PREDICTED: histone deacetylase 6 [Vitis vinifera]	53.96	8.1069	7.6	3	3	3	0.845	0.985	1	1.115	0.984	1.036	1.108	1.85E-01
cds.Camellia_sinensis.comp27480_c0_seq2_m.34221	PREDICTED: ATP-dependent Clp protease proteolytic subunit-related protein 2, chloroplastic [Nelumbo nucifera]	33.025	62.439	18.1	6	15	6	0.97	1.026	1.05	0.994	0.955	0.965	0.957	1.72E-01

cds.Camellia_sinensis.comp27483_c0_seq1_m.24196	PREDICTED: UDP-glycosyltransferase 84B1 [Vitis vinifera]	53.436	106.81	10.9	4	5	4	1.012	1.001	0.96	1.025	1.014	1.029	1.032	1.30E-01
cds.Camellia_sinensis.comp27490_c0_seq1_m.38291	PREDICTED: acyl-acyl carrier protein thioesterase ATL3, chloroplastic-like [Ziziphus jujuba]	25.853	3.4594	8.3	2	2	2	1.046	1.066	0.967	0.959	0.882	1.072	0.946	4.17E-01
cds.Camellia_sinensis.comp27497_c0_seq1_m.32426	PREDICTED: translin [Theobroma cacao]	33.965	13.927	13.4	4	4	4	1.057	0.99	0.987	0.961	0.983	1.02	0.977	4.60E-01
cds.Camellia_sinensis.comp27498_c0_seq1_m.4383	PREDICTED: protein QUIRKY [Capsicum annuum]	89.603	12.862	5.6	3	3	3	0.967	0.826	1.02	1.065	0.964	1.078	1.105	2.32E-01

cds.Camellia_sinensis.comp27502_c0_seq1_m.16363	PREDICTED: ribonuclease III domain-containing protein RNC1, chloroplastic-like isoform X1 [Daucus carota subsp. sativus]	62.479	3.4285	6.6	4	4	4	0.998	0.922	0.954	1.035	1.069	1.042	1.095	2.20E-02
cds.Camellia_sinensis.comp27505_c0_seq1_m.31043	PREDICTED: magnesium protoporphyrin IX methyltransferase, chloroplastic-like [Ipomoea	36.571	105.07	37.5	11	18	11	1.091	1.059	1.061	0.932	0.874	0.956	0.86	5.96E-03
cds.Camellia_sinensis.comp27506_c0_seq1_m.52015	PREDICTED: glutamate synthase [NADH], amyloplastic-like [Nicotiana tomentosiformis]	18.876	2.0804	4.7	1	1	1	0.95	0.999	0.964	1.037	0.992	1.077	1.066	8.54E-02

cds.Camellia_sinensis.comp27508_c0_seq1_m.20620	PREDICTED: inter-alpha-trypsin inhibitor heavy chain H3-like [Zea mays]	55.889	11.287	6.6	3	4	3	1.033	1.094	0.984	0.906	0.963	0.977	0.915	8.21E-02
cds.Camellia_sinensis.comp27511_c0_seq1_m.39327	PREDICTED: lipid phosphate phosphatase gamma, chloroplastic-like [Sesamum indicum]	25.546	8.7639	6.7	2	2	2	0.944	1.027	1.023	1.012	0.978	1.031	1.009	7.78E-01
cds.Camellia_sinensis.comp27513_c0_seq1_m.27540	PREDICTED: thiol protease aleurain-like [Ziziphus jujuba]	42.854	42.194	19.2	7	16	7	1.049	1.012	1.032	0.974	0.956	0.973	0.939	6.22E-03
cds.Camellia_sinensis.comp27514_c0_seq1_m.30024	PREDICTED: reticulon-like protein B9 [Nicotiana tabacum]	25.305	1.3201	4.1	1	1	1	1.007	1.01	1.018	1.02	0.904	1.047	0.979	6.58E-01

cds.Camellia_sinensis.comp27520_c0_seq1_m.29804	PREDICTED: probable serine/threonine-protein kinase roco9 [Arachis ipaensis]	21.767	49.026	41.5	7	10	7	1.029	0.976	0.952	1.01	0.98	1.023	1.019	5.06E-01
cds.Camellia_sinensis.comp27520_c0_seq1_m.29805	PREDICTED: probable serine/threonine-protein kinase roco9 [Vigna radiata var. radiata]	14.541	8.1939	24	3	7	3	0.99	0.98	1.027	1.025	1.011	0.956	0.998	9.44E-01
cds.Camellia_sinensis.comp27533_c1_seq1_m.26941	PREDICTED: protein PHR1-LIKE 1-like [Malus domestica]	26.186	2.2212	3.7	1	1	1	1.077	1.104	1.031	0.994	0.938	0.845	0.865	4.60E-02
cds.Camellia_sinensis.comp27536_c0_seq1_m.28571	PREDICTED: spermidine sinapoyl-CoA acyltransferase [Juglans regia]	47.381	17.178	5.4	2	2	2	1.008	1.061	1.102	0.96	0.987	0.881	0.892	5.51E-02

cds.Camellia_sinensis.comp27550_c0_seq1_m.19114	PREDICTED: cinnamate beta-D-glucosyltransferase-like [Juglans regia]	54.496	68.16	26.1	11	20	11	1.055	1.122	1.066	0.936	0.944	0.921	0.864	2.00E-03
cds.Camellia_sinensis.comp27555_c0_seq1_m.29295	PREDICTED: threonine synthase, chloroplastic [Sesamum indicum]	50.394	4.5286	12.9	5	6	2	1.023	0.956	1.038	1.057	1.033	0.906	0.993	8.82E-01
cds.Camellia_sinensis.comp27557_c0_seq2_m.24018	PREDICTED: alpha-1,4 glucan phosphorylase L isozyme, chloroplastic/amyloplastic [Vitis vinifera]	50.548	2.3968	5.7	3	3	1	0.998	1.124	1.067	0.9	0.958	0.946	0.879	3.14E-02
cds.Camellia_sinensis.comp27561_c0_seq1_m.6073	PREDICTED: probable plastidic glucose transporter 1 isoform X1 [Vitis vinifera]	61.63	18.549	5.5	3	4	3	1.021	0.953	1.017	0.998	1.02	1.038	1.022	4.32E-01

cds.Camellia_sinensis.comp27561_c0_seq1_m.6074	PREDICTED: fruit protein pKIWI501-like [Juglans regia]	17.479	80.553	61	11	28	11	1.073	1.06	1.069	0.864	0.951	0.954	0.865	4.65E-02
cds.Camellia_sinensis.comp27564_c0_seq1_m.19725	PREDICTED: low-temperature-induced cysteine proteinase [Theobroma cacao]	57.02	5.8985	3.1	1	1	1	0.876	0.79	0.712	1.169	1.275	1.245	1.551	2.48E-03
cds.Camellia_sinensis.comp27568_c0_seq1_m.34771	PREDICTED: multiple organellar RNA editing factor 3, mitochondrial [Vitis vinifera]	30.604	1.4425	4	1	1	1								
cds.Camellia_sinensis.comp27568_c0_seq3_m.38351	PREDICTED: DAG protein, chloroplastic [Daucus carota subsp. sativus]	25.737	56.057	13.7	2	6	2	1.151	1.15	1.042	0.877	0.879	0.869	0.785	1.85E-02
cds.Camellia_sinensis.comp27570_c0_seq1_m.24938	ATPase subunit 1 (mitochondrion) [Nelumbo nucifera]	50.087	197.27	47.5	21	64	19	1.021	1.017	1.021	0.982	0.977	0.977	0.96	4.20E-05

cds.Camellia_sinensis.comp27573_c0_seq1_m.18451	PREDICTED: long chain base biosynthesis protein 2a [Vitis vinifera]	57.173	20.358	9.6	6	6	6	0.977	0.983	1.031	1.025	1.025	1.018	1.026	2.73E-01
cds.Camellia_sinensis.comp27578_c0_seq1_m.30937	transmembrane protein, putative [Medicago truncatula]	28.655	2.1442	8.5	2	2	2								
cds.Camellia_sinensis.comp27580_c0_seq1_m.32417	PREDICTED: 3-ketoacyl-CoA thiolase 2, peroxisomal [Prunus mume]	41.837	7.6884	11.8	4	9	2	1.097	0.946	1.029	0.985	0.998	0.941	0.952	3.61E-01
cds.Camellia_sinensis.comp27582_c0_seq1_m.20285	PREDICTED: cytochrome P450 71A1-like isoform X2 [Populus euphratica]	58.265	37.586	6.4	3	4	3	0.934	1.02	0.947	1.07	1.034	1.065	1.092	3.88E-02
cds.Camellia_sinensis.comp27590_c0_seq1_m.35340	PREDICTED: psbP domain-containing protein 5, chloroplastic [Vitis vinifera]	28.41	24.618	22.4	5	7	5	1.01	1.039	1.087	0.952	0.957	0.93	0.905	1.28E-02

cds.Camellia_sinensis.comp27591_c1_seq1_m.40290	PREDICTED: probable calcium-binding protein CML13 [Sesamum indicum]	20.266	57.214	74	12	20	12	1.021	0.971	1.02	0.993	0.992	1.004	0.992	6.86E-01
cds.Camellia_sinensis.comp27592_c0_seq1_m.4230	PREDICTED: serine/threonine-protein phosphatase PP1 [Citrus sinensis]	36.258	7.066	12.2	4	4	3	0.996	0.934	1.002	1.005	1.05	1.027	1.051	1.23E-01
cds.Camellia_sinensis.comp27596_c0_seq1_m.19392	PREDICTED: methylmalonate-semialdehyde dehydrogenase [acylating], mitochondrial [Ipomoea nil]	60.211	84.661	20	9	14	9	0.986	1.018	1.008	1.016	1.019	0.981	1.001	9.38E-01

cds.Camellia_sinensis.comp27604_c0_seq1_m.28659	PREDICTED: zinc finger CCCH domain-containing protein 14 [Vitis vinifera]	31.76	44.513	23.4	6	10	5	1.001	0.984	1.038	1.003	0.991	0.949	0.974	3.08E-01
cds.Camellia_sinensis.comp27615_c0_seq1_m.25272	uncharacterized LOC8278573 precursor [Ricinus communis]	43.521	24.778	14.6	4	5	4	0.927	0.827	0.888	1.108	1.174	1.15	1.299	2.16E-03
cds.Camellia_sinensis.comp27622_c0_seq1_m.29090	PREDICTED: protein CHAPERONE-LIKE PROTEIN OF POR1, chloroplastic [Erythranthe guttata]	28.077	22.179	6.4	1	2	1	0.958	0.984	1.071	0.966	1.09	0.947	0.997	9.44E-01
cds.Camellia_sinensis.comp27628_c0_seq1_m.33687	PREDICTED: formimidoyltransferase-cyclodeaminase-like [Juglans regia]	32.116	12.239	13.8	4	6	4	0.969	0.983	1.052	0.994	0.984	0.964	0.979	4.93E-01

cds.Camellia_sinensis.comp27630_c0_seq1_m.38510	PREDICTED: 40S ribosomal protein S8 isoform X2 [Zea mays]	28.739	2.8368	19.8	4	5	1	0.994	1.088	1.265	0.882	0.889	0.869	0.789	8.13E-02
cds.Camellia_sinensis.comp27630_c0_seq2_m.38611	PREDICTED: 40S ribosomal protein S8-like [Oryza brachyantha]	25.06	34.033	29.3	5	9	2	1.172	0.984	1.124	0.826	0.895	0.992	0.827	6.40E-02
cds.Camellia_sinensis.comp27634_c0_seq1_m.32545	rubredoxin family protein [Arabidopsis lyrata subsp. lyrata]	28.596	35.847	22.9	6	10	6	1.097	1.038	1.011	0.941	0.955	0.976	0.913	2.61E-02
cds.Camellia_sinensis.comp27636_c0_seq1_m.40910	PREDICTED: basic transcription factor 3 [Eucalyptus grandis]	21.115	4.7737	9.7	3	5	3	1.141	1.131	1.128	0.85	0.849	0.829	0.744	2.38E-06
cds.Camellia_sinensis.comp27639_c0_seq1_m.6860	PREDICTED: ubiquitin carboxyl-terminal hydrolase 14 [Vitis vinifera]	63.227	59.972	15.2	7	11	7	0.95	0.938	0.984	1.051	1.058	1.06	1.103	2.48E-03

cds.Camellia_sinensis.comp27639_c0_seq1_m.6861	PREDICTED: ubiquitin carboxyl-terminal hydrolase 14 isoform X2 [Ricinus communis]	35.039	8.6086	13.1	4	7	4	0.953	1.015	0.988	1.051	1.034	0.984	1.038	2.37E-01
cds.Camellia_sinensis.comp27643_c0_seq3_m.32360	PREDICTED: phosphatidate cytidylyltransferase 4, chloroplastic [Theobroma cacao]	39.497	2.1629	4.6	2	2	2	0.78	1.023	1	1.041	1.127	1.076	1.157	1.64E-01
cds.Camellia_sinensis.comp27644_c0_seq2_m.7911	PREDICTED: neutral ceramidase [Gossypium raimondii]	86.825	15.218	8.1	6	7	6	0.988	1.046	0.998	1.02	0.936	1.028	0.984	6.59E-01
cds.Camellia_sinensis.comp27648_c0_seq1_m.18793	PREDICTED: serine carboxypeptidase-like 17 [Juglans regia]	53.498	4.0866	4.5	2	2	2	1.12	1.125	0.993	0.909	0.899	0.929	0.845	1.66E-02

cds.Camellia_sinensis.comp27652_c0_seq1_m.42586	PREDICTED: iron-sulfur assembly protein IscA-like 1, mitochondrial [Ricinus communis]	15.238	1.8241	8.5	1	1	1	0.992	1.034	0.931	1.066	0.967	1.023	1.033	4.71E-01
cds.Camellia_sinensis.comp27657_c0_seq1_m.25906	sucrase-related family protein [Populus trichocarpa]	47.584	2.3719	5.1	2	2	2	1.055	1.015	0.978	1.056	0.965	0.935	0.97	5.00E-01
cds.Camellia_sinensis.comp27658_c0_seq1_m.25591	plant/F14D7-9 protein [Medicago truncatula]	32.41	40.931	17.4	4	4	3	0.983	0.985	0.991	1.066	0.955	1.035	1.033	4.44E-01
cds.Camellia_sinensis.comp27664_c0_seq1_m.40058	PREDICTED: SKP1-like protein 1A isoform X1 [Nicotiana tomentosiformis]	17.537	35.622	35.5	9	17	6	1.004	0.975	0.984	1.013	1.044	1.017	1.037	4.56E-02
cds.Camellia_sinensis.comp27671_c0_seq1_m.35572	PREDICTED: eukaryotic initiation factor 4A-8 [Pyrus x bretschneideri]	28.743	112.64	53	14	44	5	0.933	0.965	0.931	1.047	1.053	1.124	1.14	6.98E-03

cds.Camellia_sinensis.comp27684_c0_seq1_m.34303	protein ACI14 [Solanum lycopersicum]	35.892	9.3945	9.4	3	3	3	0.962	1.128	0.956	1.016	0.936	1.01	0.972	6.92E-01
cds.Camellia_sinensis.comp27692_c0_seq1_m.36580		21.543	48.246	37.8	6	7	6	0.927	0.974	1.022	1.063	0.97	1.038	1.051	2.77E-01
cds.Camellia_sinensis.comp27695_c0_seq1_m.29118	Translation initiation factor eIF-2B subunit delta [Morus notabilis]	42.783	3.6592	5.5	2	3	2	1.136	1.078	1.101	0.868	0.867	0.919	0.801	8.19E-04
cds.Camellia_sinensis.comp27701_c0_seq1_m.17363	PREDICTED: plant intracellular Ras-group-related LRR protein 1-like [Ziziphus jujuba]	57.25	2.0581	1.9	1	2	1	0.96	0.937	1.032	1.077	0.959	1.056	1.056	3.09E-01
cds.Camellia_sinensis.comp27704_c0_seq1_m.12820	PREDICTED: hsp70-Hsp90 organizing protein 3-like [Ipomoea nil]	65.897	183.96	54.5	32	54	24	1.031	1.057	1.082	0.946	0.941	0.94	0.892	1.48E-02

cds.Camellia_sinensis.comp27704_c0_seq2_m.25696	PREDICTED: hsp70-Hsp90 organizing protein 3-like [Ipomoea nil]	53.506	93.35	39.8	20	28	12	1.026	1.006	1.059	0.987	0.999	0.973	0.957	6.07E-02
cds.Camellia_sinensis.comp27710_c0_seq1_m.9599	PREDICTED: KH domain-containing protein HEN4 isoform X1 [Vitis vinifera]	74.492	20.156	10	7	8	7	1.01	1.011	1.007	1.017	0.993	0.981	0.988	3.64E-01
cds.Camellia_sinensis.comp27714_c0_seq1_m.38025	PREDICTED: 50S ribosomal protein L19-1, chloroplastic-like isoform X1 [Juglans regia]	25.873	3.04	10.9	3	4	3	0.976	0.979	0.901	1.164	1.011	1.011	1.116	1.18E-01
cds.Camellia_sinensis.comp27728_c0_seq1_m.13497	PREDICTED: histone-lysine N-methyltransferase setd3 isoform X1 [Vitis vinifera]	39.396	4.1534	4.9	1	1	1								

cds.Camellia_sinensis.comp27728_c0_seq2_m.30113	PREDICTED: histone-lysine N-methyltransferase setd3-like [Nicotiana tabacum]	16.996	1.3464	5.9	1	1	1	1.035	0.953	1.114	0.993	0.936	0.97	0.935	2.43E-01
cds.Camellia_sinensis.comp27742_c0_seq1_m.29999	PREDICTED: methyltransferase-like protein 7A [Vitis vinifera]	35.948	9.6453	6.6	2	4	2	0.953	0.977	1.015	1.04	1.004	1.084	1.062	1.05E-01
cds.Camellia_sinensis.comp27744_c0_seq1_m.47324	PREDICTED: probable glutathione S-transferase parC [Nelumbo nucifera]	13.161	8.4596	35.7	6	14	6	1.006	0.983	0.958	0.985	1.04	1.028	1.036	1.80E-01
cds.Camellia_sinensis.comp27745_c0_seq1_m.30721	PREDICTED: protein FLX-like 3 [Nicotiana tabacum]	30.174	36.913	32.1	10	15	9	0.967	0.948	0.984	1.029	1.025	1.049	1.07	6.24E-03

cds.Camellia_sinensis.comp27752_c0_seq1_m.38156	PREDICTED: protein LOW PSII ACCUMULATION 2, chloroplastic [Jatropha curcas]	11.417	14.846	26.4	3	4	3	1.028	1.084	1.292	0.749	0.988	0.834	0.755	5.64E-02
cds.Camellia_sinensis.comp27753_c0_seq1_m.42051	PREDICTED: peroxidase 12 [Eucalyptus grandis]	22.094	34.611	25.5	4	7	4	0.96	1.005	0.998	1.044	0.996	1.015	1.031	1.96E-01
cds.Camellia_sinensis.comp27764_c0_seq3_m.29841	starch-binding domain-containing protein [Solanum lycopersicum]	47.353	8.2614	9.3	3	3	3	0.997	1.073	1.043	0.989	0.876	1.021	0.927	2.06E-01
cds.Camellia_sinensis.comp27766_c0_seq2_m.40392	PREDICTED: serine/threonine-protein phosphatase 2A 65 kDa regulatory subunit A beta isoform-like [Ziziphus jujuba]	23.479	21.093	26.8	6	10	5	0.956	0.942	0.98	1.077	1.034	1.029	1.091	9.26E-03

cds.Camellia_sinensis.comp27767_c0_seq1_m.16273	PREDICTED: abscisate beta-glucosyltransferase [Eucalyptus grandis]	53.201	65.096	17.5	9	15	9	0.952	0.982	0.981	1.07	0.993	1.015	1.056	9.11E-02
cds.Camellia_sinensis.comp27776_c0_seq1_m.46698	transmembrane protein, putative [Medicago truncatula]	18.786	3.3119	12.2	2	2	2	0.995	1.033	1.001	0.92	1.087	0.97	0.983	7.18E-01
cds.Camellia_sinensis.comp27782_c0_seq1_m.29375	PREDICTED: ATP synthase subunit O, mitochondrial [Erythranthe guttata]	32.228	78.023	39.3	12	34	12	1.036	0.998	1.03	0.986	0.978	0.968	0.957	2.63E-02
cds.Camellia_sinensis.comp27787_c0_seq2_m.44511	PREDICTED: ras-related protein RABB1c isoform X1 [Amborella trichopoda]	21.341	95.928	43.1	6	12	4	0.998	0.993	0.974	0.998	1.041	1.076	1.051	1.01E-01

cds.Camellia_sinensis.comp27788_c0_seq1_m.26729	PREDICTED: Golgi to ER traffic protein 4 homolog [Sesamum indicum]	37.336	4.5838	7	2	2	2	0.995	1.059	0.91	1.009	0.947	1.087	1.027	6.77E-01
cds.Camellia_sinensis.comp27792_c0_seq1_m.11601	PREDICTED: mannosyl-oligosaccharide 1,2-alpha-mannosidase MNS1-like [Nicotiana attenuata]	23.408	14.129	25.7	5	7	5	1.05	1.069	1.157	0.897	0.884	0.926	0.826	4.42E-03
cds.Camellia_sinensis.comp27796_c0_seq4_m.39734	PREDICTED: 5-formyltetrahydrofolate cyclo-ligase-like protein COG0212 isoform X2 [Nicotiana tomentosiformis]	28.227	8.1476	15	4	4	4	0.969	0.954	1.027	0.992	1.034	1.002	1.026	3.61E-01

cds.Camellia_sinensis.comp27798_c0_seq1_m.6496	PREDICTED: protein WEAK CHLOROPLAST MOVEMENT UNDER BLUE LIGHT 1-like [Solanum tuberosum	100.35	7.4288	6.9	7	8	7	1.06	1.048	0.999	0.98	0.93	0.943	0.918	2.37E-02
cds.Camellia_sinensis.comp27802_c0_seq1_m.27276	PREDICTED: V-type proton ATPase subunit C [Nicotiana	42.381	76.035	25.9	11	16	11	0.993	1.005	0.988	1.02	1.005	1.013	1.017	5.97E-02
cds.Camellia_sinensis.comp27806_c0_seq1_m.43057	PREDICTED: 60S ribosomal protein L24-like [Jatropha curcas]	18.518	11.774	12.3	3	4	3	1.077	1.122	1.101	0.895	0.914	0.895	0.819	1.36E-04
cds.Camellia_sinensis.comp27811_c0_seq1_m.22502	PREDICTED: protein ABCI7, chloroplastic [Vitis vinifera]	55.575	29.628	9.7	5	7	4	0.992	1.027	1.03	0.964	0.983	0.986	0.962	5.06E-02

cds.Camellia_sinensis.comp27812_c0_seq1_m.19185	PREDICTED: inorganic phosphate transporter 1-4-like [Sesamum indicum]	58.905	11.545	7.3	5	5	4	0.867	0.915	0.95	1.184	1.017	1.102	1.209	2.13E-02
cds.Camellia_sinensis.comp27818_c0_seq1_m.24693	PREDICTED: alpha-1,4-glucan-protein synthase [UDP-forming] 2 [Malus domestica]	49.056	79.383	35.3	15	32	7	1.038	1.036	1.018	0.995	0.982	0.973	0.954	6.32E-03
cds.Camellia_sinensis.comp27818_c0_seq2_m.27266	PREDICTED: alpha-1,4-glucan-protein synthase [UDP-forming] [Pyrus x bretschneideri]	48.174	8.9578	23.5	11	25	3	1.083	1.004	1.054	0.968	0.971	0.923	0.911	2.81E-02
cds.Camellia_sinensis.comp27819_c0_seq2_m.28471	PREDICTED: high mobility group B protein 10 isoform X2 [Vitis vinifera]	36.777	1.3388	2.1	1	1	1	0.988	1.107	1.038	0.93	1.04	0.898	0.915	1.81E-01

cds.Camellia_sinensis.comp27828_c0_seq1_m.36752	PREDICTED: 3-oxoacyl-[acyl-carrier-protein] reductase FabG-like [Nicotiana tabacum]	29.725	18.371	19.4	5	7	5	0.961	0.949	0.942	1.081	1.039	1.039	1.108	2.12E-03
cds.Camellia_sinensis.comp27829_c0_seq1_m.21294	PREDICTED: calcium-dependent protein kinase SK5-like [Solanum tuberosum	48.345	14.644	12.1	4	7	4	0.958	0.956	1.055	1.079	0.943	1.03	1.028	6.24E-01
cds.Camellia_sinensis.comp27847_c0_seq1_m.40758	PREDICTED: ADP-ribosylation factor-like protein 8A isoform X1 [Capsicum annuum]	25.298	24.817	27.7	5	9	4	0.995	0.99	0.999	1.049	1.065	0.95	1.027	5.56E-01
cds.Camellia_sinensis.comp27855_c0_seq2_m.26247	PREDICTED: very-long-chain enoyl-CoA reductase isoform X1 [Vitis vinifera]	36.396	6.2256	9	3	4	3	1.099	1.257	1.193	0.876	0.743	0.791	0.679	3.32E-03

cds.Camellia_sinensis.comp27863_c0_seq1_m.16442	PREDICTED: internal alternative NAD(P)H-ubiquinone oxidoreductase A1, mitochondrial-like [Sesamum	56.426	7.8437	6.8	4	5	4	0.919	0.957	1.018	0.999	1.016	1.029	1.052	1.73E-01
cds.Camellia_sinensis.comp27864_c0_seq1_m.32651	PREDICTED: 40S ribosomal protein S6-like [Musa acuminata subsp. malaccensis]	28.348	5.7212	17.7	4	7	1	1.013	1.393	0.983	0.898	0.851	0.836	0.763	8.52E-02
cds.Camellia_sinensis.comp27865_c0_seq1_m.35278	PREDICTED: protein CURVATURE THYLAKOID 1D, chloroplastic isoform X2 [Vitis vinifera]	23.502	19.589	16.2	3	4	3	0.959	1.017	1.037	1.011	1.016	0.971	0.995	8.74E-01
cds.Camellia_sinensis.comp27866_c0_seq2_m.50182	PREDICTED: 21 kDa protein [Prunus mume]	14.686	11.886	16.2	2	2	2	1.001	0.972	0.987	0.992	1.056	1.005	1.031	2.16E-01

cds.Camellia_sinensis.comp27870_c0_seq1_m.37067	PREDICTED: clathrin light chain 2-like [Cucumis sativus]	29.03	11.628	20.5	5	8	4	1.029	1.071	1.043	0.995	0.93	0.949	0.914	1.81E-02
cds.Camellia_sinensis.comp27877_c0_seq1_m.49510	PREDICTED: prolyl endopeptidase [Jatropha curcas]	22.529	29.787	30.6	6	21	4	0.994	0.991	0.975	0.986	1	0.985	1.004	6.55E-01
cds.Camellia_sinensis.comp27879_c0_seq1_m.18135	aspartate aminotransferase 2 family protein [Populus trichocarpa]	24.805	55.619	56.4	12	23	12	1.016	1.005	1.003	0.979	1	0.972	0.976	6.00E-02
cds.Camellia_sinensis.comp27879_c0_seq2_m.30639	PREDICTED: aspartate aminotransferase, chloroplastic [Vitis vinifera]	26.489	196.54	56	12	38	11	0.982	1.007	0.985	1.028	0.999	0.998	1.017	2.47E-01
cds.Camellia_sinensis.comp27880_c1_seq2_m.22949	PREDICTED: pectinesterase [Ricinus communis]	53.21	6.199	2	1	2	1	0.958	1.07	1.033	1.027	0.917	1.004	0.963	4.72E-01

cds.Camellia_sinensis.comp27898_c0_seq1_m.15788	PREDICTED: protein EDS1L-like [Nicotiana tabacum]	70.017	2.1	3.4	2	2	2	1.125	1.007	1.024	0.89	0.887	1.046	0.894	1.53E-01
cds.Camellia_sinensis.comp27901_c0_seq1_m.30576	PREDICTED: phosphomannomutase [Vitis vinifera]	35.57	31.106	24	7	11	7	1.062	1.025	1.04	0.97	0.96	0.965	0.926	1.98E-03
cds.Camellia_sinensis.comp27903_c0_seq1_m.9824	PREDICTED: E3 ubiquitin-protein ligase KEG isoform X2 [Vitis vinifera]	56.061	59.81	20.2	9	12	9	0.955	1.038	0.952	1.059	1.021	1.042	1.06	1.22E-01
cds.Camellia_sinensis.comp27907_c0_seq1_m.42167	PREDICTED: protein SRG1 [Theobroma cacao]	32.276	4.8274	15.2	4	5	4	0.923	1	1.013	1.041	1.036	0.964	1.036	4.08E-01
cds.Camellia_sinensis.comp27911_c0_seq1_m.14961	PREDICTED: CBS domain-containing protein CBSCBSPB5 isoform X2 [Eucalyptu	24.753	5.6274	6.6	1	1	1								

cds.Camellia_sinensis.comp27922_c0_seq2_m.27931	PREDICTED: SAL1 phosphatase-like isoform X2 [Glycine max]	15.268	18.903	26.6	4	7	4	1.012	0.992	0.982	1.021	0.985	1.019	1.013	4.27E-01
cds.Camellia_sinensis.comp27928_c0_seq2_m.41003	PREDICTED: FAD synthetase 2, chloroplastic [Daucus carota subsp. sativus]	23.008	1.8726	4.4	1	1	1	0.977	1.073	0.93	1.056	0.912	1.062	1.017	8.18E-01
cds.Camellia_sinensis.comp27929_c0_seq1_m.29995	PREDICTED: probable 3-hydroxyisobutyrate dehydrogenase-like 1, mitochondrial [Theobroma cacao]	36.431	37.046	13.6	3	4	3	0.942	0.923	0.93	1.032	1.117	1.085	1.157	3.68E-03
cds.Camellia_sinensis.comp27931_c0_seq1_m.36596	structural constituent of ribosome protein [Medicago truncatula]	30.169	5.015	11.5	3	5	3	0.882	1.038	0.976	1.023	1.089	1.043	1.089	1.61E-01

cds.Camellia_sinensis.comp27939_c0_seq1_m.20010	PREDICTED: bifunctional purple acid phosphatase 26-like [Juglans regia]	56.306	6.5291	3.9	1	1	1	1.106	0.797	1.048	1.106	1.126	0.834	1.039	7.95E-01
cds.Camellia_sinensis.comp27947_c0_seq1_m.40362	PREDICTED: eukaryotic translation initiation factor 4B3-like [Nicotiana tabacum]	33.886	3.253	3.6	1	1	1								
cds.Camellia_sinensis.comp27949_c0_seq1_m.37702	PREDICTED: ras-related protein RHN1 isoform X2 [Vitis vinifera]	20.595	56.108	41.8	7	15	5	0.952	0.935	0.946	1.01	1.066	1.095	1.119	9.96E-03
cds.Camellia_sinensis.comp27949_c0_seq2_m.38344	PREDICTED: ras-related protein RHN1 isoform X1 [Vitis vinifera]	22.398	4.9477	25.5	4	10	2	0.936	0.937	0.902	1.055	1.142	1.085	1.183	3.14E-03

cds.Camellia_sinensis.comp27950_c0_seq1_m.26267	PREDICTED: diaminopimelate epimerase, chloroplastic [Vitis vinifera]	33.412	47.806	20.2	5	8	5	1.044	0.98	0.981	0.976	0.993	1.003	0.989	6.59E-01
cds.Camellia_sinensis.comp27953_c0_seq1_m.41972	PREDICTED: neutral/alkaline invertase 3, chloroplastic [Vitis vinifera]	23.421	8.1818	5.1	1	1	1	0.953	1.002	1.06	0.957	1.015	1.024	0.994	8.84E-01
cds.Camellia_sinensis.comp27960_c0_seq1_m.32009	PREDICTED: calnexin homolog [Nelumbo nucifera]	35.4	18.662	9.3	2	3	2	1.27	0.94	1.088	0.845	0.817	0.993	0.805	1.14E-01
cds.Camellia_sinensis.comp27965_c0_seq1_m.30992	PREDICTED: persulfide dioxygenase ETHE1 homolog, mitochondrial-like [Nicotiana attenuata]	24.57	4.618	10.7	2	2	2								

cds.Camellia_sinensis.comp27972_c0_seq1_m.37314	tagatose-6-phosphate ketose/aldose isomerase, putative (Mog1/PsbP/DUF1795-like photosystem II reaction center PsbP family protein) [Arabidopsis thaliana]	24.541	4.8023	20.1	4	5	4	1.006	0.969	0.994	0.995	1.007	1.039	1.024	2.32E-01
cds.Camellia_sinensis.comp27975_c0_seq1_m.40790	PREDICTED: calmodulin-binding protein 60 C isoform X2 [Vitis vinifera]	28.407	1.5849	9.4	3	3	1	0.943	1.024	1.05	0.941	1.013	1.039	0.992	8.68E-01
cds.Camellia_sinensis.comp27976_c0_seq2_m.13381	PREDICTED: glutamyl-tRNA(Gln) amidotransferase subunit A, chloroplastic/mitochondrial [Ipomoea	57.917	158.92	35.4	14	23	14	1.016	0.992	1.02	0.999	0.963	0.982	0.972	1.09E-01

cds.Camellia_sinensis.comp27982_c0_seq1_m.46320	PREDICTED: thioredoxin-like protein Clot [Prunus mume]	15.003	4.7147	14.4	2	3	2	1.008	0.973	0.997	0.938	1.067	1.024	1.017	7.11E-01
cds.Camellia_sinensis.comp27989_c0_seq2_m.7925	PREDICTED: ABC transporter F family member 4-like [Cucumis melo]	76.984	11.67	8.2	4	4	4	1.004	1.096	1.027	1.006	0.878	1.042	0.936	3.07E-01
cds.Camellia_sinensis.comp27991_c0_seq2_m.43729	PREDICTED: probable calcium-binding protein CML20 [Pyrus x bretschneideri]	19.305	15.527	26.6	4	6	4	1.092	1.115	1.023	0.914	0.896	0.939	0.851	5.36E-03
cds.Camellia_sinensis.comp27994_c0_seq1_m.4637	PREDICTED: protein ENHANCED DISEASE RESISTANCE 2 isoform X2 [Prunus	52.503	1.2203	2.8	1	1	1								

cds.Camellia_sinensis.comp27995_c0_seq2_m.34073	PREDICTED: NADPH-dependent pterin aldehyde reductase [Vitis vinifera]	26.09	13.616	30.8	8	11	8	0.932	0.995	1.028	0.964	0.936	1.018	0.987	7.60E-01
cds.Camellia_sinensis.comp27998_c0_seq1_m.15252	PREDICTED: glucose-1-phosphate adenylyltransferase large subunit 3, chloroplastic/amyloplastic [Vitis vinifera]	57.648	216.71	40	17	31	16	1.034	0.982	1.011	0.996	1.023	0.977	0.99	6.36E-01
cds.Camellia_sinensis.comp28002_c0_seq1_m.43612	PREDICTED: S-adenosylmethionine synthase 5 [Vitis vinifera]	16.905	8.329	33.8	5	13	1	1.185	1.116	1.175	0.749	0.859	0.866	0.712	2.52E-03
cds.Camellia_sinensis.comp28011_c0_seq1_m.30896	PREDICTED: 1-aminocyclopropane-1-carboxylate oxidase homolog 1-like [Nelumbo nucifera]	43.315	43.911	18.7	7	12	7	0.984	1.005	1.044	1	1.003	0.967	0.979	3.76E-01

cds.Camellia_sinensis.comp28015_c0_seq1_m.40723	PREDICTED: 2-alkenal reductase (NADP(+)-dependent) isoform X2 [Sesamum indicum]	15.401	3.0054	19	2	3	1	0.917	1.047	0.97	1.119	0.962	1.011	1.054	4.27E-01
cds.Camellia_sinensis.comp28022_c0_seq1_m.46866	PREDICTED: remorin [Cucumis melo]	18.086	53.093	39.9	10	18	9	0.93	0.915	0.938	1.075	1.098	1.099	1.176	9.69E-05
cds.Camellia_sinensis.comp28024_c0_seq1_m.49734	PREDICTED: 5-methyltetrahydropteroyltriglutamate--homocysteine methyltransferase-like [Malus domestica]	13.603	1.6773	13.9	2	6	1	1.014	0.982	0.958	0.978	1.035	1.018	1.026	3.36E-01

cds.Camellia_sinensis.comp28027_c0_seq2_m.24842	PREDICTED: probable L-ascorbate peroxidase 6, chloroplastic isoform X1 [Ipomoea nil]	46.949	227.17	54.2	22	70	22	1.031	1.005	1.005	0.954	0.995	0.983	0.964	7.29E-02
cds.Camellia_sinensis.comp28030_c0_seq10_c0_seq1_m.24237	enoyl-CoA hydratase/isomerase family protein [Populus trichocarpa]	50.105	27.293	12.9	6	8	6	1.425	1.421	1.381	0.502	0.508	0.547	0.368	2.02E-06
cds.Camellia_sinensis.comp28032_c0_seq1_m.26675	PREDICTED: NADP-dependent D-sorbitol-6-phosphate dehydrogenase [Ricinus communis]	38.055	20.426	22.1	8	11	8	0.991	0.996	0.971	1.021	1.012	1.04	1.039	2.70E-02

cds.Camellia_sinensis.comp28043_c0_seq1_m.15070	PREDICTED: AT-rich interactive domain-containing protein 5 isoform X1 [Ipomoea nil]	50.837	4.2775	5.3	3	3	2	1.006	0.98	1.047	0.927	1.038	1.006	0.98	6.11E-01
cds.Camellia_sinensis.comp28047_c0_seq1_m.35838	PREDICTED: GTP-binding protein YPTM2 [Populus euphratica]	22.733	104.91	56.2	9	17	3	0.943	0.99	0.975	1.066	0.993	1.075	1.078	6.21E-02
cds.Camellia_sinensis.comp28047_c0_seq2_m.37543	PREDICTED: GTP-binding protein YPTM2 [Populus euphratica]	22.663	7.9338	48.3	8	15	2	0.955	0.985	0.974	0.971	1.047	1.083	1.064	1.42E-01
cds.Camellia_sinensis.comp28047_c0_seq3_m.38110	PREDICTED: GTP-binding protein YPTM2 [Theobroma cacao]	22.397	11.844	43.8	7	14	3	0.938	0.968	0.9	1.076	1.059	1.049	1.135	4.78E-03
cds.Camellia_sinensis.comp28052_c0_seq1_m.12818	PREDICTED: quinolinate synthase, chloroplastic [Juglans regia]	77.597	4.4983	3.7	3	3	3	0.913	0.927	0.991	1.034	1.107	1.103	1.146	1.53E-02

cds.Camellia_sinensis.comp28061_c0_seq1_m.27880	PREDICTED: cell division cycle protein 27 homolog isoform X1 [Prunus	36.516	40.68	18.7	5	8	5	1.042	0.972	1.01	0.989	0.962	1.022	0.983	5.60E-01
cds.Camellia_sinensis.comp28068_c0_seq1_m.20528	PREDICTED: cysteine desulfurase, mitochondrial [Nelumbo nucifera]	52.03	30.089	18.7	8	10	8	1.01	1.002	0.982	1.004	0.985	0.957	0.984	3.73E-01
cds.Camellia_sinensis.comp28071_c0_seq1_m.22627	chloroplast sedoheptulose-1,7-bisphosphatase [Solanum lycopersicum]	42.433	175.21	52.7	20	78	20	1.05	1.031	0.999	0.954	0.989	0.969	0.945	3.52E-02
cds.Camellia_sinensis.comp28079_c0_seq1_m.3191	PREDICTED: NF-X1-type zinc finger protein NFXL1 [Vitis vinifera]	121.82	1.9529	0.9	1	1	1								

cds.Camellia_sinensis.comp28080_c0_seq2_m.39935	PREDICTED: 60S ribosomal protein L11 [Ricinus communis]	21.368	16.851	21.3	4	10	1	1.144	1.1	1.132	0.835	0.894	0.859	0.767	3.20E-04
cds.Camellia_sinensis.comp28080_c0_seq3_m.40155	PREDICTED: 60S ribosomal protein L11 [Amborella trichopoda]	24.404	20.326	22.3	5	11	2	1.092	1.04	1.128	0.873	0.929	0.894	0.827	3.10E-03
cds.Camellia_sinensis.comp28105_c0_seq1_m.34296	PREDICTED: probable thiol methyltransferase 2 isoform X3 [Citrus sinensis]	27.905	8.3268	12.1	2	2	2								
cds.Camellia_sinensis.comp28106_c0_seq1_m.46951	PREDICTED: 60S ribosomal protein L21-1 [Daucus carota subsp. sativus]	18.716	16.424	30.5	6	12	6	1.198	1.207	1.205	0.774	0.798	0.807	0.659	2.54E-06
cds.Camellia_sinensis.comp28111_c0_seq1_m.41736	PREDICTED: thioredoxin H-type [Prunus mume]	13.804	19.626	52.8	7	20	6	1.015	1.01	0.92	1.077	0.999	1.022	1.052	2.59E-01

cds.Camellia_sinensis.comp28122_c0_seq1_m.8123	PREDICTED: G-type lectin S-receptor-like serine/threonine-protein kinase RLK1 isoform X2 [Populus euphratica]	91.731	4.2612	2.8	2	2	2								
cds.Camellia_sinensis.comp28123_c0_seq1_m.42227	PREDICTED: glycine-rich RNA-binding protein GRP1A-like [Nelumbo nucifera]	16.786	140.3	50	7	26	5	1.047	1.04	1.041	0.955	0.958	0.965	0.92	2.19E-05
cds.Camellia_sinensis.comp28131_c0_seq1_m.18968	PREDICTED: riboflavin biosynthesis protein PYRR, chloroplastic isoform X2 [Vitis vinifera]	64.245	5.2259	3.2	3	3	3	0.963	1.013	0.995	1.006	1.026	1.012	1.025	1.99E-01

cds.Camellia_sinensis.comp28134_c0_seq1_m.30420	PREDICTED: ABC transporter I family member 19 [Daucus carota subsp.	33.344	3.7229	7.1	2	2	2	0.951	1.029	0.929	1.097	1.068	0.953	1.072	2.66E-01
cds.Camellia_sinensis.comp28139_c0_seq1_m.39401	PREDICTED: protein EMBRYO SAC DEVELOPMENT ARREST 3, chloroplastic [Citrus sinensis]	23.422	1.2262	7	1	1	1								
cds.Camellia_sinensis.comp28142_c0_seq1_m.18076	PREDICTED: trans-cinnamate 4-monooxygenase [Jatropha curcas]	60.624	61.458	26.1	13	20	13	1.245	1.257	1.245	0.711	0.741	0.714	0.578	1.10E-06
cds.Camellia_sinensis.comp28153_c0_seq1_m.44111	PREDICTED: pentatricopeptide repeat-containing protein At1g12620-like [Nicotiana sylvestris]	23.375	-2	4.8	1	2	1	0.954	0.927	0.958	1.061	1.099	1.03	1.124	5.56E-03

cds.Camellia_sinensis.comp28154_c0_seq1_m.48869	L-ascorbate peroxidase 5 [Solanum lycopersicum]	18.71	1.2985	4.7	1	1	1								
cds.Camellia_sinensis.comp28155_c0_seq1_m.11399	PREDICTED: probable alpha-galactosidase B [Citrus sinensis]	40.988	114.72	34.8	10	21	10	1.001	1	1.012	1.005	1.03	0.99	1.004	7.67E-01
cds.Camellia_sinensis.comp28155_c0_seq1_m.11400	PREDICTED: probable alpha-galactosidase B [Citrus sinensis]	33.929	2.7771	5.7	2	3	2	0.951	0.969	0.946	1.061	1.046	1.052	1.102	3.17E-04
cds.Camellia_sinensis.comp28156_c0_seq5_m.31264	PREDICTED: haloacid dehalogenase-like hydrolase domain-containing protein At4g39970 [Ipomoea	34.039	27.98	16.8	5	10	5	1.045	1.057	1.019	0.949	0.973	0.914	0.909	1.05E-02

cds.Camellia_sinensis.comp28158_c0_seq1_m.21407	PREDICTED: isoaspartyl peptidase/L-asparaginase 1 [Vitis vinifera]	34.597	58.738	20.8	6	9	6	1.004	0.963	0.982	1.035	0.984	1.035	1.036	1.68E-01
cds.Camellia_sinensis.comp28166_c0_seq1_m.24152	PREDICTED: calreticulin-3-like [Populus euphratica]	46.459	17.718	21.8	8	12	6	1.056	1.083	1.075	0.921	0.906	0.896	0.847	1.16E-04
cds.Camellia_sinensis.comp28167_c0_seq1_m.27410	PREDICTED: patatin-like protein 2 [Vitis vinifera]	45.447	55.067	23.2	10	10	10	0.961	0.99	0.97	1.076	1.017	1.005	1.061	6.25E-02
cds.Camellia_sinensis.comp28168_c1_seq6_m.40232	PREDICTED: ubiquitin-conjugating enzyme E2 10-like [Gossypium hirsutum]	16.547	10.366	12.8	2	3	2	1.071	0.995	1.056	0.914	0.981	0.955	0.913	4.07E-02
cds.Camellia_sinensis.comp28169_c0_seq1_m.38581	PREDICTED: 60S ribosomal protein L13-1 [Nelumbo nucifera]	23.908	22.169	23.7	5	8	5	1.136	1.116	1.11	0.855	0.887	0.865	0.775	3.95E-05

cds.Camellia_sinensis.comp28178_c0_seq1_m.24102	PREDICTED: hepatocyte growth factor-regulated tyrosine kinase substrate isoform X3 [Juglans regia]	32.126	7.1292	10.6	2	2	2								
cds.Camellia_sinensis.comp28181_c0_seq1_m.12362	PREDICTED: DEAD-box ATP-dependent RNA helicase 53 [Vitis vinifera]	41.29	6.3789	12.3	4	5	4	0.843	0.938	0.859	1.169	1.101	1.145	1.294	2.28E-03
cds.Camellia_sinensis.comp28181_c0_seq1_m.12363	PREDICTED: DEAD-box ATP-dependent RNA helicase 53-like [Citrus sinensis]	28.392	8.1285	12.5	3	3	3	0.923	0.933	0.959	1.056	1.054	1.105	1.142	2.30E-03

cds.Camellia_sinensis.comp28208_c0_seq1_m.9743	PREDICTED: delta-1-pyrroline-5-carboxylate synthase [Sesamum indicum]	78.684	19.134	12.9	10	13	10	0.982	1.024	1.008	0.998	0.998	1.038	1.007	7.32E-01
cds.Camellia_sinensis.comp28215_c0_seq8_m.14740	PREDICTED: heterogeneous nuclear ribonucleoprotein 1-like [Juglans regia]	47.577	138.5	13.7	5	16	4	1.001	0.983	0.975	1.012	0.997	1.019	1.023	8.44E-02
cds.Camellia_sinensis.comp28217_c0_seq1_m.36291	glycoprotein family protein [Medicago truncatula]	28.787	8.2185	8.2	2	3	2	0.997	1.008	0.987	1.051	0.932	1.028	1.006	9.00E-01
cds.Camellia_sinensis.comp28217_c0_seq2_m.36876	glycoprotein family protein [Medicago truncatula]	32.255	5.1714	5.9	2	2	2	1.096	1.088	1.054	0.901	0.957	0.885	0.847	3.30E-03
cds.Camellia_sinensis.comp28222_c0_seq1_m.16700	PREDICTED: glutathione reductase, cytosolic isoform X1 [Vitis vinifera]	53.876	138.97	40.8	18	30	18	0.964	0.94	0.979	1.082	1.046	1.058	1.105	2.94E-03

cds.Camellia_sinensis.comp28223_c0_seq1_m.29677	PREDICTED: peptidyl-prolyl cis-trans isomerase [Vitis vinifera]	28.234	86.54	23.5	7	31	6	0.989	1.022	0.991	0.99	0.991	0.985	0.988	3.30E-01
cds.Camellia_sinensis.comp28225_c0_seq1_m.30489	PREDICTED: 50S ribosomal protein L35, chloroplastic-like [Erythranthe guttata]	16.558	2.1519	9.3	1	1	1								
cds.Camellia_sinensis.comp28227_c0_seq1_m.17295	PREDICTED: sterol 14-demethylase-like [Ziziphus jujuba]	55.457	4.7626	3.9	2	3	2	1.046	1.069	1.092	0.936	0.906	0.898	0.854	8.82E-04
cds.Camellia_sinensis.comp28228_c0_seq1_m.35208	PREDICTED: eukaryotic translation initiation factor 3 subunit A [Erythranthe guttata]	35.716	8.756	27.5	4	6	1	1.045	0.98	1.008	1.022	1.003	0.947	0.98	5.26E-01

cds.Camellia_sinensis.comp28228_c0_seq2_m.35783	PREDICTED: eukaryotic translation initiation factor 3 subunit A [Erythranthe guttata]	35.827	1.2063	27.5	4	6	1	1.086	1.057	1.039	0.934	0.978	0.894	0.882	1.20E-02
cds.Camellia_sinensis.comp28231_c0_seq1_m.12401	PREDICTED: protein NUCLEAR FUSION DEFECTIVE 4-like [Jatropha curcas]	64.249	10.79	5.8	4	4	4	1.031	1.046	1.001	0.903	0.972	1.05	0.95	3.10E-01
cds.Camellia_sinensis.comp28234_c0_seq1_m.37114	PREDICTED: thioredoxin-like protein slr0233 [Prunus mume]	23.685	66.066	27.1	6	13	6	1.032	1.033	1.007	1.007	0.953	0.981	0.957	7.16E-02
cds.Camellia_sinensis.comp28236_c1_seq1_m.20964	PREDICTED: monodehydroascorbate reductase 4, peroxisomal [Vitis vinifera]	53.216	31.229	17.8	8	13	8	0.968	0.95	0.966	1.029	1.056	1.065	1.092	1.78E-03

cds.Camellia_sinensis.comp28244_c0_seq1_m.32818	PREDICTED: NADH dehydrogenase [ubiquinone] flavoprotein 2, mitochondrial [Sesamum indicum]	28.356	7.0782	16.5	4	7	4	1.006	0.944	1.041	0.97	0.988	1.005	0.991	7.89E-01
cds.Camellia_sinensis.comp28247_c0_seq1_m.14164	GTPase Era [Morus notabilis]	54.209	3.5653	4.2	2	2	2	1.032	1.094	1.129	0.89	0.922	0.918	0.839	3.58E-03
cds.Camellia_sinensis.comp28252_c0_seq1_m.26270	PREDICTED: protein SLOW GREEN 1, chloroplastic-like [Nicotiana attenuata]	40.33	17.016	6.8	3	6	3	1.009	0.953	0.969	1.007	1.058	1.036	1.058	6.37E-02
cds.Camellia_sinensis.comp28255_c0_seq1_m.3638	PREDICTED: mitochondrial arginine transporter BAC2-like isoform X1 [Nicotiana tabacum]	41.072	1.1869	1.9	1	1	1	0.951	0.977	0.985	1.023	1.039	1.045	1.067	6.44E-03

cds.Camellia_sinensis.comp28256_c0_seq2_m.21404	PREDICTED: probable endo-1,3(4)-beta-glucanase ARB_01444 [Daucus carota subsp. sativus]	64.753	15.364	9.6	5	5	5	0.902	0.927	0.884	1.091	1.085	1.077	1.199	2.19E-04
cds.Camellia_sinensis.comp28274_c0_seq1_m.4439	PREDICTED: calmodulin-binding transcription activator 6 isoform X1 [Vitis vinifera]	106.06	6.616	3.7	4	4	4	1.135	1.067	1.102	0.877	0.877	0.9	0.803	3.81E-04
cds.Camellia_sinensis.comp28278_c0_seq1_m.37133	PREDICTED: EKC/KEOPS complex subunit bud32 [Juglans regia]	29.411	6.4551	8.8	2	2	2	1.085	0.992	1.13	0.94	0.888	0.951	0.867	3.17E-02

cds.Camellia_sinensis.comp28287_c0_seq1_m.16003	PREDICTED: methionine--tRNA ligase, chloroplastic/mitochondrial [Vitis vinifera]	69.586	55.997	15.6	8	9	8	1.038	0.993	0.975	0.998	0.996	0.986	0.991	6.82E-01
cds.Camellia_sinensis.comp28290_c0_seq1_m.27632	PREDICTED: ribokinase isoform X1 [Theobroma cacao]	42.09	3.4232	7.5	4	4	4	0.993	1.001	0.984	0.99	1.015	1	1.009	3.63E-01
cds.Camellia_sinensis.comp28292_c0_seq3_m.27179	PREDICTED: thylakoid lumenal protein TL20.3, chloroplastic isoform X1 [Juglans regia]	30.293	275.84	57.6	13	30	13	0.994	0.999	1.002	0.975	1.008	0.998	0.995	6.62E-01
cds.Camellia_sinensis.comp28297_c0_seq1_m.31083	NAD(P)-binding rossmann-fold protein [Medicago truncatula]	39.069	74.143	46.1	15	37	6	0.979	0.998	0.967	1.033	1.025	1.017	1.044	1.30E-02

cds.Camellia_sinensis.comp28297_c0_seq2_m.34773	NAD-dependent epimerase/dehydratase [Zea mays]	27.944	158.94	61.2	15	31	6	1.066	1.011	1.038	0.926	0.966	0.958	0.915	1.14E-02
cds.Camellia_sinensis.comp28300_c0_seq1_m.40644	PREDICTED: 50S ribosomal protein L4, chloroplastic-like [Nelumbo nucifera]	33.539	11.727	9.6	3	4	3	0.933	0.915	0.94	1.059	1.119	1.098	1.175	8.76E-04
cds.Camellia_sinensis.comp28302_c1_seq1_m.15926	PREDICTED: TOM1-like protein 2 [Vitis vinifera]	67.699	11.354	2.3	1	1	1	0.989	0.851	0.992	1.127	1.115	0.959	1.13	1.67E-01
cds.Camellia_sinensis.comp28303_c2_seq1_m.19063	PREDICTED: cytochrome P450 704C1 [Theobroma cacao]	57.329	168.42	47.5	22	40	22	1.066	1.008	1.02	0.966	0.967	0.963	0.936	6.11E-02
cds.Camellia_sinensis.comp28305_c0_seq1_m.37395	PREDICTED: ras-related protein RABH1b-like [Nicotiana tabacum]	22.998	38.405	32.4	7	12	4	1.08	1.013	1.027	1.025	0.878	0.971	0.921	1.65E-01

cds.Camellia_sinensis.comp28312_c0_seq2_m.16725	PREDICTED: 40S ribosomal protein S15a [Erythranthe guttata]	14.787	10.525	25.4	4	8	4	0.97	0.953	0.976	1.031	1.086	1.031	1.086	1.18E-02
cds.Camellia_sinensis.comp28313_c0_seq2_m.21555	PREDICTED: DNA polymerase I isoform X1 [Theobroma cacao]	47.596	8.8056	4	1	1	1	1.041	0.921	1.024	1.087	0.969	0.97	1.013	8.18E-01
cds.Camellia_sinensis.comp28314_c0_seq2_m.31619	BAG family molecular chaperone regulator 4 [Asparagus officinalis]	41.296	5.1013	5.4	2	2	2								
cds.Camellia_sinensis.comp28329_c0_seq1_m.34253	PREDICTED: 40S ribosomal protein S3-3-like [Nelumbo nucifera]	26.352	72.336	56.6	13	25	6	0.944	0.937	0.953	1.033	1.005	1.048	1.089	3.00E-03
cds.Camellia_sinensis.comp28333_c0_seq1_m.25292	PREDICTED: alcohol dehydrogenase-like 1 [Sesamum indicum]	50.616	64.179	20.3	7	9	7	0.958	1.007	1.002	1.107	1.009	0.977	1.042	3.76E-01

cds.Camellia_sinensis.comp28338_c0_seq1_m.35010	PREDICTED: protein PALE CRESS, chloroplastic-like isoform X1 [Nicotiana tabacum]	36.376	5.8212	8.4	3	4	3	1.045	0.947	1.006	0.99	0.958	1.002	0.984	6.50E-01
cds.Camellia_sinensis.comp28341_c0_seq2_m.49807	PREDICTED: 40S ribosomal protein S9-2 [Solanum pennellii]	19.591	17.582	25	5	9	5	0.941	0.939	0.973	1.08	1.111	1.057	1.138	2.16E-03
cds.Camellia_sinensis.comp28353_c0_seq2_m.15608	PREDICTED: protein HOTHEAD-like isoform X1 [Nicotiana tabacum]	60.774	1.272	1.6	1	1	1								
cds.Camellia_sinensis.comp28356_c0_seq1_m.13732	PREDICTED: mitochondrial Rho GTPase 1-like isoform X1 [Ipomoea nil]	70.98	1.153	5.5	4	5	2								

cds.Camellia_sinensis.comp28357_c1_seq1_m.51060	PREDICTED: cinnamoyl-CoA reductase 1-like isoform X2 [Populus euphratica]	19.709	3.2853	12.8	3	4	3	0.894	1.041	1.023	1.006	1.033	1.031	1.038	4.65E-01
cds.Camellia_sinensis.comp28358_c0_seq1_m.9158	PREDICTED: subtilisin-like protease SBT1.9 [Vitis vinifera]	84.728	2.3926	4.1	3	3	3	1.102	1.052	1.049	0.92	0.928	0.931	0.868	9.79E-04
cds.Camellia_sinensis.comp28359_c0_seq1_m.6188	PREDICTED: protein PLASTID MOVEMENT IMPAIRED 1 [Theobroma cacao]	95.151	88.673	19.6	16	21	16	1.13	1.122	1.143	0.854	0.868	0.852	0.758	2.04E-06
cds.Camellia_sinensis.comp28362_c0_seq1_m.34005	DUF2358 family protein [Medicago truncatula]	34.304	33.242	7.3	3	4	3	0.937	1	0.933	1.067	1.016	1.038	1.087	3.38E-02
cds.Camellia_sinensis.comp28364_c1_seq2_m.35218	PREDICTED: 40S ribosomal protein S5 [Vitis vinifera]	23.957	57.408	33.5	8	18	8	0.972	0.98	0.977	1.059	1.039	1.037	1.07	6.76E-04

cds.Camellia_sinensis.comp28367_c0_seq1_m.37830	PREDICTED: ras-related protein RABF1 [Vitis vinifera]	21.816	4.6955	17.5	3	4	2								
cds.Camellia_sinensis.comp28371_c0_seq1_m.32315	PREDICTED: (R,S)-reticuline 7-O-methyltransferase [Theobroma cacao]	39.728	2.5522	3.6	1	1	1	0.977	0.838	0.99	1.274	0.941	1.021	1.154	2.61E-01
cds.Camellia_sinensis.comp28373_c0_seq1_m.22341	PREDICTED: aldehyde dehydrogenase family 3 member H1 isoform X1 [Prunus	53.03	86.596	18.6	9	11	9	0.956	0.969	1.01	1.065	1.02	0.987	1.047	1.74E-01
cds.Camellia_sinensis.comp28374_c1_seq1_m.35279	PSII-associated proline-rich protein [Solanum lycopersicum]	23.73	48.953	37.4	5	15	5	1.041	1.019	1.01	0.959	0.985	0.959	0.946	1.15E-02

cds.Camellia_sinensis.comp28406_c0_seq1_m.27823	PREDICTED: ubiquitin fusion degradation protein 1 homolog isoform X2 [Populus euphratica]	35.788	61.348	26.4	8	10	7	1.012	1.062	1.095	0.926	0.928	0.941	0.882	5.98E-03
cds.Camellia_sinensis.comp28414_c0_seq1_m.22936	PREDICTED: PTI1-like tyrosine-protein kinase 1 [Juglans regia]	27.048	14.318	19.3	4	6	3	0.941	0.988	1.007	1.085	1.02	1.005	1.059	1.37E-01
cds.Camellia_sinensis.comp28414_c0_seq4_m.53285	protein kinase Pti1 [Glycine max]	11.218	2.3462	8.1	1	1	1	1.02	0.987	0.954	0.902	1.062	1.079	1.028	7.06E-01
cds.Camellia_sinensis.comp28415_c0_seq1_m.12957	PREDICTED: transmembrane 9 superfamily member 9-like [Ziziphus jujuba]	73.938	11.401	5.8	3	3	3	1.03	1.051	1.075	0.891	0.973	0.972	0.899	2.74E-02

cds.Camellia_sinensis.comp28417_c0_seq1_m.22464	PREDICTED: glyceraldehyde-3-phosphate dehydrogenase, cytosolic [Eucalyptus grandis]	20.841	56.776	60	9	42	3	1.036	1.006	0.954	0.982	1.021	1.02	1.009	7.48E-01
cds.Camellia_sinensis.comp28417_c0_seq1_m.22467	glyceraldehyde-3-phosphate dehydrogenase, cytosolic [Pyrus x bretschneideri]	11.603	156.3	89.5	9	58	8	0.984	0.938	0.951	1.033	1.06	1.043	1.092	5.50E-03
cds.Camellia_sinensis.comp28419_c0_seq1_m.38820	PREDICTED: 1,2-dihydroxy-3-keto-5-methylthiopentene dioxygenase 2 [Nicotiana tabacum]	23.712	14.581	29.4	6	7	5	0.919	0.955	1.043	1.058	1.026	1.026	1.066	1.68E-01
cds.Camellia_sinensis.comp28425_c0_seq1_m.10099	PREDICTED: heat shock cognate protein 80 [Ziziphus jujuba]	62.23	267.5	45.3	27	68	14	1.112	1.084	1.104	0.873	0.881	0.899	0.804	4.29E-05

cds.Camellia_sinensis.comp28425_c0_seq1_m.10101	PREDICTED: heat shock cognate protein 80 [Ziziphus jujuba]	19.329	55.962	33.5	6	15	6	1.058	1.149	1.06	0.898	0.9	0.897	0.825	1.96E-02
cds.Camellia_sinensis.comp28430_c0_seq1_m.26340	PREDICTED: 12-oxophytodienoate reductase 3-like [Jatropha curcas]	43.773	41.732	22.6	9	12	9	0.908	0.997	0.99	1.012	1.041	1.068	1.078	8.86E-02
cds.Camellia_sinensis.comp28431_c0_seq1_m.36056	PREDICTED: 60S ribosomal protein L10 [Vitis vinifera]	27.358	17.045	21.2	5	6	5	1.151	1.104	1.095	0.84	0.859	0.853	0.762	8.26E-05
cds.Camellia_sinensis.comp28435_c0_seq2_m.34929	PREDICTED: NAP1-related protein 2 [Vitis vinifera]	29.633	23.108	15.1	4	5	4	0.943	0.949	1.011	1.057	1.014	1.03	1.068	5.88E-02
cds.Camellia_sinensis.comp28437_c0_seq1_m.30989	PREDICTED: putative deoxyribonuclease TATDN1 [Juglans regia]	36.31	1.4454	3.4	1	1	1								

cds.Camellia_sinensis.comp28438_c0_seq1_m.38905	60S acidic ribosomal protein family [Arabidopsis thaliana]	11.5	31.842	36.3	2	6	2	1.037	0.913	0.878	1.024	1.096	1.069	1.128	8.63E-02
cds.Camellia_sinensis.comp28445_c0_seq1_m.18163	PREDICTED: bifunctional 3-dehydroquinate dehydratase/shikimate dehydrogenase, chloroplastic [Vitis vinifera]	56.806	13.472	8.6	5	7	4	1.421	1.362	1.341	0.547	0.603	0.591	0.422	1.75E-05
cds.Camellia_sinensis.comp28451_c0_seq4_m.34490	PREDICTED: U1 small nuclear ribonucleoprotein A isoform X1 [Vitis vinifera]	23.206	5.1279	10.6	2	3	2	1.063	1.017	1.115	1.014	0.919	0.868	0.877	6.48E-02

cds.Camellia_sinensis.comp28458_c0_seq2_m.12232	PREDICTED: CBS domain-containing protein CBSCBSPB3-like isoform X1 [Nicotiana tabacum]	58.901	64.476	29.4	11	12	11	0.988	1.043	0.99	0.989	0.963	0.995	0.976	2.94E-01
cds.Camellia_sinensis.comp28473_c0_seq1_m.35295	PREDICTED: probable 2-carboxy-D-arabinitol-1-phosphatase [Juglans regia]	21.804	24.328	25.5	4	8	4	1.056	1.053	1.086	0.963	0.928	0.899	0.873	3.60E-03
cds.Camellia_sinensis.comp28473_c0_seq1_m.35296	PREDICTED: probable 2-carboxy-D-arabinitol-1-phosphatase isoform X2 [Ricinus communis]	13.993	18.762	29.2	3	3	3	1.08	1.032	1.052	0.918	0.97	0.944	0.895	5.80E-03

cds.Camellia_sinensis.comp28481_c0_seq2_m.48310	PREDICTED: cell division cycle protein 48 homolog [Arachis ipaensis]	23.78	1.1683	23	5	11	1	1.08	1.03	1.084	0.9	0.936	0.955	0.874	4.90E-03
cds.Camellia_sinensis.comp28483_c0_seq1_m.24621	PREDICTED: protein TRANSPARENT TESTA GLABRA 1-like [Ziziphus jujuba]	38.348	5.152	6.7	2	2	2	0.993	1.081	1.065	1.08	0.875	0.913	0.914	2.48E-01
cds.Camellia_sinensis.comp28498_c0_seq1_m.36521	PREDICTED: VAMP-like protein YKT61 [Ricinus communis]	22.666	9.3791	29.1	6	6	6	0.949	0.98	1.017	1.037	1.069	0.985	1.049	1.99E-01
cds.Camellia_sinensis.comp28499_c2_seq1_m.45054	PREDICTED: thioredoxin-like 3-1, chloroplastic [Theobroma cacao]	22.141	6.5676	21.7	5	9	4	0.989	1.013	0.957	1.005	1.016	1.052	1.039	1.53E-01

cds.Camellia_sinensis.comp28509_c0_seq1_m.24190	PREDICTED: methionyl-tRNA formyltransferase [Citrus sinensis]	32.367	3.3052	7	2	2	2								
cds.Camellia_sinensis.comp28510_c0_seq1_m.34868	PREDICTED: (DL)-glycerol-3-phosphatase 2 [Sesamum indicum]	30.507	21.586	15.9	4	4	4	0.998	1.001	0.966	1.005	0.999	1.03	1.023	1.93E-01
cds.Camellia_sinensis.comp28514_c0_seq1_m.2113	PREDICTED: topless-related protein 1-like isoform X1 [Nicotiana sylvestris]	122.45	20.443	10.6	11	13	7	0.993	0.987	1.054	1.025	0.963	0.985	0.98	5.07E-01
cds.Camellia_sinensis.comp28516_c0_seq1_m.3672	PREDICTED: importin-11 isoform X1 [Vitis vinifera]	113.24	2.8156	1.5	1	1	1	1.022	0.93	0.976	1.152	0.926	1.014	1.056	4.99E-01

cds.Camellia_sinensis.comp28520_c0_seq1_m.30390	PREDICTED: probable voltage-gated potassium channel subunit beta [Ricinus communis]	38.515	28.049	25.9	12	22	12	1.014	1.027	1.021	1.001	0.962	0.971	0.958	2.70E-02
cds.Camellia_sinensis.comp28522_c0_seq1_m.15851	PREDICTED: protein DETOXIFICATION 41-like [Juglans regia]	55.266	4.2103	2.2	1	1	1								
cds.Camellia_sinensis.comp28524_c0_seq1_m.40659	PREDICTED: thylakoid lumenal 15 kDa protein 1, chloroplastic-like isoform X2 [Populus euphratica]	24.028	204.83	46.2	8	25	8	0.934	0.966	0.928	1.091	1.072	1.044	1.134	2.14E-03
cds.Camellia_sinensis.comp28534_c0_seq1_m.8188	PREDICTED: sucrose synthase 7-like [Nicotiana attenuata]	98.673	71.657	24.1	21	28	19	0.933	0.922	0.99	1.059	1.052	1.063	1.116	3.85E-02

cds.Camellia_sinensis.comp28537_c0_seq1_m.11963	PREDICTED: protein arginine N-methyltransferase 1.5 isoform X1 [Vitis vinifera]	72.557	10.893	1.7	1	1	1	0.923	0.998	0.95	1.004	1.025	1.123	1.098	8.81E-02
cds.Camellia_sinensis.comp28540_c0_seq1_m.17423	PREDICTED: ATP-dependent 6-phosphofructokinase 5, chloroplastic-like isoform X1 [Juglans regia]	60.305	8.6349	7.1	4	5	3	0.978	0.953	1.029	0.983	1.028	1.013	1.022	4.52E-01
cds.Camellia_sinensis.comp28540_c0_seq1_m.17425		12.923	-2	11.4	1	1	1	1.036	1.02	0.998	0.965	1.04	0.942	0.965	3.14E-01
cds.Camellia_sinensis.comp28542_c0_seq1_m.42075	PREDICTED: ras-related protein Rab7 [Capsicum annuum]	23.056	15.717	36.1	7	11	5	0.914	1.015	0.952	1	1.043	1.01	1.06	1.50E-01

cds.Camellia_sinensis.comp28543_c0_seq2_m.4228	PREDICTED: putative acyl-activating enzyme 19 isoform X4 [Pyrus x bretschneideri]	105.76	-2	1.3	1	1	1	0.974	1.117	0.933	0.862	1.01	1.101	0.983	8.42E-01
cds.Camellia_sinensis.comp28544_c0_seq2_m.45493	PREDICTED: histone H1 [Vitis vinifera]	25.458	13.92	18.1	4	6	4	1.18	1.147	1.172	0.82	0.863	0.805	0.711	1.05E-04
cds.Camellia_sinensis.comp28544_c2_seq1_m.42824	PREDICTED: histone H1 [Vitis vinifera]	16.262	36.331	32.9	7	9	7	1.099	1.122	1.151	0.81	0.863	0.921	0.769	2.60E-03
cds.Camellia_sinensis.comp28548_c0_seq2_m.54773	PREDICTED: probable NAD(P)H dehydrogenase (quinone) FQR1-like 1 [Eucalyptus grandis]	15.039	3.3033	27.9	5	8	1	1.024	0.974	0.934	1.048	1.038	0.996	1.051	1.78E-01
cds.Camellia_sinensis.comp28551_c0_seq1_m.18183	PREDICTED: protoporphyrinogen oxidase 1, chloroplastic [Vitis vinifera]	62.148	32.276	16.5	9	13	9	1.027	1.068	1.033	0.931	0.958	0.935	0.903	2.48E-03

cds.Camellia_sinensis.comp28555_c0_seq5_m.45688	PREDICTED: 60S ribosomal protein L34-like [Ziziphus jujuba]	13.737	11.453	16.7	2	4	2	1.03	1.027	1.063	0.958	1.006	0.916	0.923	5.09E-02
cds.Camellia_sinensis.comp28560_c0_seq1_m.32994	PREDICTED: probable caffeoyl-CoA O-methyltransferase At4g26220 [Jatropha curcas]	26.165	37.445	25.1	4	6	4	1.015	1.036	0.99	0.997	1.019	0.986	0.987	4.75E-01
cds.Camellia_sinensis.comp28566_c0_seq1_m.16933	Allene oxide synthase [Morus notabilis]	59.022	201.04	34.5	15	27	15	0.98	0.983	1.013	1.019	1.073	1.004	1.04	1.60E-01
cds.Camellia_sinensis.comp28567_c0_seq1_m.19821	PREDICTED: zinc-binding alcohol dehydrogenase domain-containing protein 2 [Vitis vinifera]	50.739	9.2833	12.8	5	6	5	1.009	0.969	1.009	0.997	1.005	1.027	1.014	4.32E-01
cds.Camellia_sinensis.comp28570_c0_seq2_m.15458	MLO-like protein 1 [Cucumis melo]	55.148	2.5016	1.8	1	1	1								

cds.Camellia_sinensis.comp28571_c0_seq9_m.4622	PREDICTED: leucine--tRNA ligase, chloroplastic/mitochondrial isoform X1 [Theobroma cacao]	114.99	79.461	16.1	14	23	14	1.018	1.043	1.013	0.981	0.963	0.943	0.939	1.25E-02
cds.Camellia_sinensis.comp28572_c0_seq1_m.34652	6,7-dimethyl-8-ribityllumazine synthase [Arabidopsis thaliana]	28.521	32.367	5	1	1	1	0.985	1.09	1.112	0.99	0.904	0.92	0.883	5.70E-02
cds.Camellia_sinensis.comp28574_c0_seq3_m.25434	PREDICTED: syntaxin-22-like isoform X1 [Jatropha curcas]	30.301	6.8029	7	2	4	2	1.069	0.94	0.951	1.063	1.001	0.985	1.03	5.49E-01
cds.Camellia_sinensis.comp28594_c0_seq1_m.35130	PREDICTED: probable inactive receptor kinase At2g26730 [Vitis vinifera]	33.135	11.061	9.3	3	4	3	0.976	0.974	0.988	1.015	1.043	0.958	1.027	3.72E-01

cds.Camellia_sinensis.comp28599_c0_seq1_m.9441	PREDICTED: fimbrin-1-like [Ipomoea nil]	82.374	1.8909	1.4	1	1	1								
cds.Camellia_sinensis.comp28601_c0_seq1_m.16117	PREDICTED: alpha,alpha-trehalose-phosphate synthase [UDP-forming] 6 [Vitis vinifera]	70.966	2.2724	2.4	1	1	1								
cds.Camellia_sinensis.comp28607_c0_seq1_m.20197	PREDICTED: dolichyl-diphosphooligosaccharide--protein glycosyltransferase 48 kDa subunit isoform X2 [Vitis vinifera]	48.836	10.237	6.4	3	3	3	0.964	0.974	0.972	1.071	1.022	1.04	1.077	6.28E-03

cds.Camellia_sinensis.comp28614_c0_seq1_m.4055	PREDICTED: cytochrome b6-f complex iron-sulfur subunit 1, chloroplastic [Vitis vinifera]	19.905	209.27	64.3	9	42	9	0.99	0.97	0.994	1.014	1.019	1.01	1.03	2.04E-02
cds.Camellia_sinensis.comp28615_c0_seq1_m.28385	PREDICTED: COP9 signalosome complex subunit 6a [Vitis	34.23	2.5372	3.9	1	1	1								
cds.Camellia_sinensis.comp28616_c0_seq1_m.32795	PREDICTED: nuclear transcription factor Y subunit C-3-like [Cicer arietinum]	28.057	8.0652	16.9	4	4	3	0.926	1.111	0.959	1.014	0.968	1.037	1.008	8.69E-01
cds.Camellia_sinensis.comp28618_c0_seq1_m.22058	PREDICTED: thiamine thiazole synthase 2, chloroplastic [Nelumbo nucifera]	38.079	87.48	24.3	7	27	2	1.005	0.933	0.968	1.029	1.082	1.037	1.083	3.91E-02

cds.Camellia_sinensis.comp28618_c0_seq2_m.24583	PREDICTED: thiamine thiazole synthase 2, chloroplastic [Nelumbo nucifera]	37.555	6.6661	23.8	6	26	1	1.02	0.979	0.992	1.043	1.043	0.946	1.014	7.33E-01
cds.Camellia_sinensis.comp28624_c0_seq1_m.45429	PREDICTED: cytochrome b5 [Vitis vinifera]	18.273	18.801	16.2	2	2	2	1.217	1.16	1.125	0.743	0.876	0.823	0.697	2.42E-03
cds.Camellia_sinensis.comp28631_c0_seq1_m.3052	PREDICTED: structural maintenance of chromosomes protein 1 [Vitis vinifera]	140.04	1.511	0.9	1	1	1								
cds.Camellia_sinensis.comp28632_c0_seq1_m.24756	PREDICTED: cytochrome P450 94A1 [Sesamum indicum]	46.848	2.4849	4.7	2	2	2								
cds.Camellia_sinensis.comp28635_c0_seq1_m.26795	PREDICTED: serpin-ZX [Theobroma cacao]	42.504	98.058	27.5	10	15	10	0.952	1.012	0.989	1.01	1.002	1.014	1.025	2.45E-01

cds.Camellia_sinensis.comp28640_c0_seq80_m.15653	PREDICTED: serine/threonine-protein kinase STN7, chloroplastic [Juglans regia]	65.012	106.95	23.1	13	21	13	0.957	0.938	0.958	1.083	1.055	1.055	1.119	5.37E-04
cds.Camellia_sinensis.comp28647_c1_seq1_m.46830	PREDICTED: serine carboxypeptidase-like [Juglans regia]	14.359	2.8562	6.4	1	2	1	1.034	0.942	1.046	1.013	0.958	1.012	0.987	7.63E-01
cds.Camellia_sinensis.comp28649_c0_seq1_m.14486	PREDICTED: la-related protein 6B [Juglans regia]	56.159	4.4973	2	1	1	1	1.185	1.089	1.089	0.8	0.87	0.923	0.771	6.50E-03
cds.Camellia_sinensis.comp28650_c1_seq2_m.19059	PREDICTED: probable acyl-activating enzyme 5, peroxisomal isoform X2 [Vitis vinifera]	61.164	6.2333	3.9	2	3	2	0.838	0.928	1.006	1.173	1.046	1.059	1.183	5.78E-02

cds.Camellia_sinensis.comp28651_c0_seq1_m.27956	PREDICTED: peptidyl-prolyl cis-trans isomerase FKBP42 [Theobroma cacao]	40.853	11.602	4.8	2	2	2	1.015	1.035	1.006	0.971	0.997	0.978	0.964	3.35E-02
cds.Camellia_sinensis.comp28652_c0_seq1_m.27184	PREDICTED: probable protein phosphatase 2C 9 isoform X1 [Vitis vinifera]	31.107	1.8449	4.2	1	1	1	1.02	0.958	1.08	0.928	1.029	0.987	0.963	4.57E-01
cds.Camellia_sinensis.comp28654_c0_seq1_m.17144	PREDICTED: acetate/butyrate--CoA ligase AAE7, peroxisomal [Vitis vinifera]	62.339	18.957	14.5	10	15	10	0.964	0.933	0.945	1.061	1.062	1.06	1.12	6.98E-03
cds.Camellia_sinensis.comp28660_c0_seq1_m.35924	PREDICTED: 60S ribosomal protein L8-1-like [Erythranthe guttata]	29.08	36.374	23.2	6	10	6	1.131	1.136	1.179	0.876	0.838	0.865	0.748	1.00E-04

cds.Camellia_sinensis.comp28662_c0_seq1_m.25855	PREDICTED: 26S protease regulatory subunit 6B homolog [Juglans regia]	48.401	85.481	29.8	11	18	4	1.018	0.977	0.988	0.847	1.063	1.025	0.984	7.84E-01
cds.Camellia_sinensis.comp28672_c1_seq1_m.6252	PREDICTED: probable glucan 1,3-alpha-glucosidase [Pyrus x bretschneideri]	104.89	93.033	20.5	19	29	19	0.997	1.001	1.03	0.975	1.033	0.987	0.989	6.13E-01
cds.Camellia_sinensis.comp28680_c0_seq1_m.34192	plant/F14G9-20 protein [Medicago truncatula]	42.54	12.387	7.8	3	3	3	1.012	1.08	0.976	0.961	0.997	0.975	0.956	2.33E-01
cds.Camellia_sinensis.comp28686_c0_seq1_m.17887	PREDICTED: mitochondrial phosphate carrier protein 3, mitochondrial [Jatropha curcas]	46.134	2.1466	18.9	8	15	1	1.045	1.137	1.022	0.898	0.966	0.919	0.869	2.28E-02

cds.Camellia_sinensis.comp28686_c0_seq2_m.19806	PREDICTED: mitochondrial phosphate carrier protein 3, mitochondrial [Jatropha curcas]	45.833	23.38	20.8	9	17	2	1.049	0.971	1.037	0.973	0.991	0.98	0.963	2.07E-01
cds.Camellia_sinensis.comp28689_c0_seq3_m.12759	PREDICTED: sodium/pyruvate cotransporter BASS2, chloroplastic [Vitis vinifera]	28.424	2.7041	5.2	1	2	1	0.976	0.858	0.916	1.07	1.151	1.062	1.194	1.70E-02
cds.Camellia_sinensis.comp28691_c0_seq1_m.31137	PREDICTED: serine/threonine-protein kinase SAPK2-like isoform X1 [Gossypium raimondii]	20.204	2.1693	8.5	2	4	1	1.013	1.06	0.949	1.001	1.033	0.951	0.988	7.82E-01
cds.Camellia_sinensis.comp28692_c0_seq1_m.16119	PREDICTED: 2-hydroxyacyl-CoA lyase [Ipomoea nil]	61.941	151.18	44.6	22	53	22	0.976	0.956	0.978	1.031	1.034	1.018	1.059	2.68E-03

cds.Camellia_sinensis.comp28693_c0_seq2_m.28000	PREDICTED: golgin subfamily A member 2-like [Nicotiana sylvestris]	29.593	67.315	29.2	9	17	9	1.04	1.031	1.048	0.96	0.964	0.939	0.918	8.16E-04
cds.Camellia_sinensis.comp28699_c0_seq1_m.21392	PREDICTED: fasciclin-like arabinogalactan protein 10 [Vitis vinifera]	43.524	13.772	7.6	3	3	3	1.063	1.018	1.029	0.953	0.996	0.935	0.927	2.94E-02
cds.Camellia_sinensis.comp28701_c0_seq1_m.31292	PREDICTED: protein-ribulosamine 3-kinase, chloroplastic [Jatropha curcas]	39.908	7.8604	10.7	4	6	4	0.948	1.009	0.955	1.01	0.979	1.066	1.049	2.07E-01
cds.Camellia_sinensis.comp28704_c0_seq1_m.23471	PREDICTED: BAHD acyltransferase DCR [Nicotiana attenuata]	49.995	46.095	13.4	5	6	5	1.335	1.368	1.341	0.636	0.605	0.629	0.462	6.90E-07

cds.Camellia_sinensis.comp28706_c0_seq1_m.43479	PREDICTED: 40S ribosomal protein S20-2-like isoform X2 [Gossypium hirsutum]	21.472	45.646	20.5	5	10	5	1.024	1.031	1.077	0.94	0.989	0.97	0.926	2.34E-02
cds.Camellia_sinensis.comp28708_c1_seq1_m.31563	PREDICTED: probable UDP-arabinopyranose mutase 5 isoform X1 [Pyrus x bretschneideri]	27.822	19.632	30.2	7	9	6	0.894	1.036	0.978	1.139	1.043	0.946	1.076	3.54E-01
cds.Camellia_sinensis.comp28708_c1_seq1_m.31564	PREDICTED: probable UDP-arabinopyranose mutase 5 isoform X1 [Vitis vinifera]	15.651	4.2423	9.4	1	1	1								

cds.Camellia_sinensis.comp28711_c0_seq1_m.30494	PREDICTED: bifunctional UDP-glucose 4-epimerase and UDP-xylose 4-epimerase 1 [Juglans regia]	38.909	11.005	24.3	7	11	6	1.012	0.986	0.983	1.034	1.015	0.963	1.01	6.89E-01
cds.Camellia_sinensis.comp28715_c0_seq1_m.16881	PREDICTED: WD-40 repeat-containing protein MSI4-like [Populus euphratica]	56.68	111.44	22.5	8	13	3	1.078	1.073	1.142	0.913	0.892	0.883	0.816	8.20E-04
cds.Camellia_sinensis.comp28715_c0_seq2_m.17799	PREDICTED: WD-40 repeat-containing protein MSI4-like [Populus euphratica]	56.594	4.3342	18.4	6	12	1								
cds.Camellia_sinensis.comp28717_c0_seq2_m.31648	PREDICTED: tryptophan synthase alpha chain-like [Citrus sinensis]	33.301	53.794	36.9	8	11	8	0.935	0.935	0.944	1.13	1.076	1.039	1.153	2.87E-02

cds.Camellia_sinensis.comp28719_c0_seq1_m.15381	PREDICTED: suppressor of mec-8 and unc-52 protein homolog 1 isoform X1 [Vitis vinifera]	60.164	10.895	10	5	6	5	0.954	0.918	0.929	1.016	1.09	1.125	1.154	1.16E-02
cds.Camellia_sinensis.comp28721_c0_seq1_m.33393	PREDICTED: DNA-directed RNA polymerases IV and V subunit 4 [Malus domestica]	23.06	3.4038	11.7	3	3	3	1.186	1.157	1.205	0.885	0.762	0.766	0.68	1.54E-03
cds.Camellia_sinensis.comp28723_c0_seq1_m.811	PREDICTED: eukaryotic translation initiation factor 4G isoform X2 [Vitis vinifera]	207.74	18.041	4.5	7	9	7	1.012	0.982	1.054	0.931	1.024	0.999	0.969	4.18E-01
cds.Camellia_sinensis.comp28724_c0_seq1_m.17777	PREDICTED: probable nucleoredoxin 1 isoform X1 [Prunus	66.378	151.68	41.5	23	56	22	0.892	0.899	0.881	1.113	1.114	1.112	1.25	7.01E-04

cds.Camellia_sinensis.comp28724_c0_seq2_m.18204	PREDICTED: probable nucleoredoxin 1 [Jatropha curcas]	66.017	13.087	10.9	6	13	5	1.049	1.02	1.043	0.98	0.923	0.973	0.924	1.88E-02
cds.Camellia_sinensis.comp28728_c0_seq1_m.39416	PREDICTED: probable aminopyrimidine aminohydrolase, mitochondrial isoform X2 [Pyrus x bretschnei	16.042	1.3906	11.3	2	3	1	0.982	0.875	0.974	0.944	1.123	1.121	1.126	1.63E-01
cds.Camellia_sinensis.comp28732_c0_seq1_m.10852	PREDICTED: acidic repeat-containing protein isoform X1 [Populus euphratica]	43.497	-2	2.3	1	1	1	0.896	0.886	0.934	1.053	1.147	1.125	1.224	2.68E-03
cds.Camellia_sinensis.comp28733_c0_seq1_m.10252	PREDICTED: threonine--tRNA ligase, mitochondrial 1 [Vitis	84.386	60.04	27.2	22	34	22	0.974	0.944	0.95	1.06	1.056	1.026	1.096	2.90E-03

cds.Camellia_sinensis.comp28738_c0_seq1_m.3615	PREDICTED: ATP-dependent zinc metalloprotease FTSH 12, chloroplastic [Prunus mume]	68.108	2.4193	3.6	2	2	2	0.971	1.018	1.059	0.972	1.007	0.982	0.971	3.55E-01
cds.Camellia_sinensis.comp28738_c0_seq1_m.3616	PREDICTED: ATP-dependent zinc metalloprotease FTSH 12, chloroplastic [Gossypium raimondii]	44.86	24.226	16.2	5	5	5	0.925	1.015	1.028	1.033	1.04	0.985	1.03	4.58E-01
cds.Camellia_sinensis.comp28739_c0_seq1_m.32735	PREDICTED: glutelin type-A 3 isoform X1 [Vitis vinifera]	37.496	38.573	19.7	6	11	6	0.982	0.923	0.963	1.053	1.074	1.01	1.094	2.53E-02
cds.Camellia_sinensis.comp28745_c0_seq2_m.27151	PREDICTED: mevalonate kinase [Solanum lycopersicum]	41.914	2.2238	2.3	1	1	1								

cds.Camellia_sinensis.comp28748_c0_seq2_m.19166	PREDICTED: putative G3BP-like protein isoform X4 [Juglans regia]	46.869	7.2712	8.4	4	5	4	1.013	1.046	0.996	0.946	1.023	0.974	0.963	2.35E-01
cds.Camellia_sinensis.comp28749_c0_seq2_m.10694	PREDICTED: ABC transporter B family member 26, chloroplastic isoform X1 [Vitis vinifera]	76.502	41.596	10.1	7	7	7	0.965	1.036	1.011	1.031	0.947	1.023	0.996	9.13E-01
cds.Camellia_sinensis.comp28751_c0_seq11_m.36414	PREDICTED: non-specific lipid transfer protein GPI-anchored 1 [Vitis vinifera]	20.567	6.1567	5.2	1	1	1	1.458	1.459	1.338	0.517	0.547	0.543	0.378	4.31E-06
cds.Camellia_sinensis.comp28751_c1_seq11_m.26359	PREDICTED: metacaspase-4 [Theobroma cacao]	46.206	33.742	21.6	9	13	9	1.017	1.038	1.044	0.944	0.993	0.987	0.944	3.07E-02

cds.Camellia_sinensis.comp28753_c0_seq1_m.10763	PREDICTED: probable pectinesterase/pectinesterase inhibitor 61 [Sesamum indicum]	65.748	23.809	7.5	5	9	5	1.059	1.018	1.062	0.936	0.934	0.943	0.896	1.42E-03
cds.Camellia_sinensis.comp28757_c0_seq1_m.27387	PREDICTED: aldose 1-epimerase [Juglans regia]	37.045	14.618	16.8	5	9	5	0.949	0.96	0.988	1.036	1.057	0.989	1.064	5.55E-02
cds.Camellia_sinensis.comp28762_c0_seq1_m.34011	PREDICTED: nitrile-specifier protein 5 [Daucus carota subsp. sativus]	39.845	29.873	34.7	9	11	9	1.038	1.038	0.982	0.976	0.998	0.927	0.949	1.39E-01
cds.Camellia_sinensis.comp28768_c0_seq2_m.13174	plant/F17O14-7 protein [Medicago truncatula]	32.627	3.6145	10.7	3	3	3	1.013	1.008	0.964	0.977	1.012	1.032	1.012	6.22E-01
cds.Camellia_sinensis.comp28770_c0_seq2_m.5558	PREDICTED: probable endo-1,3(4)-beta-glucanase ARB_01444 [Citrus sinensis]	17.185	17.11	23.8	3	7	3	0.984	0.915	0.921	1.102	1.02	1.078	1.135	1.80E-02

cds.Camellia_sinensis.comp28780_c0_seq1_m.33527	PREDICTED: copper chaperone for superoxide dismutase, chloroplastic/cytosolic [Jatropha curcas]	35.732	95.431	41.5	12	21	12	0.937	1.013	0.999	1.025	1.012	1.016	1.035	2.84E-01
cds.Camellia_sinensis.comp28786_c0_seq1_m.32971	PREDICTED: aspartic proteinase Asp1-like isoform X1 [Citrus sinensis]	40.28	15.436	10.9	4	6	4	0.943	1.008	1.006	1.043	1.016	1.005	1.036	2.17E-01
cds.Camellia_sinensis.comp28791_c0_seq1_m.20293	PREDICTED: cytochrome P450 704C1 isoform X2 [Populus euphratica]	60.199	5.3799	5.2	2	2	2								
cds.Camellia_sinensis.comp28793_c0_seq1_m.14957	PREDICTED: synaptotagmin-5 [Malus domestica]	55.874	59.912	15.3	7	12	7	0.944	0.946	0.979	1.051	1.075	1.067	1.113	1.38E-03

cds.Camellia_sinensis.comp28794_c0_seq1_m.19751	PREDICTED: glutamine synthetase leaf isozyme, chloroplastic [Juglans regia]	47.936	121.11	42.4	16	43	16	1.067	1.037	1.03	0.946	0.954	0.95	0.909	1.02E-03
cds.Camellia_sinensis.comp28797_c0_seq1_m.33679	PREDICTED: non-specific phospholipase C2 isoform X2 [Citrus sinensis]	40.953	2.1263	8.2	3	4	2	1.009	1.013	1.027	0.932	1.021	1	0.969	3.09E-01
cds.Camellia_sinensis.comp28803_c0_seq1_m.13853	PREDICTED: xylulose kinase [Vitis vinifera]	61.118	52.011	19.4	9	16	9	0.961	0.979	0.963	1.067	1.035	1.017	1.074	9.24E-03
cds.Camellia_sinensis.comp288099_c0_seq1_m.58829	PREDICTED: universal stress protein A-like protein isoform X2 [Cucumis sativus]	11.806	1.4107	7.7	1	2	1	0.991	1.044	0.926	0.975	1.043	1.03	1.029	5.03E-01

cds.Camellia_sinensis.comp28813_c1_seq1_m.29578	PREDICTED: protein ASPARTIC PROTEASE IN GUARD CELL 2-like [Sesamum indicum]	40.944	4.1554	4.7	1	1	1								
cds.Camellia_sinensis.comp28814_c0_seq1_m.25455	PREDICTED: probable 3-hydroxyisobutyrate dehydrogenase, mitochondrial isoform X1 [Vitis	39.572	1.3916	2.2	1	1	1	1.097	1.028	1.088	0.864	0.998	0.908	0.862	3.33E-02
cds.Camellia_sinensis.comp28818_c0_seq1_m.28081	PREDICTED: protein HGH1 homolog [Gossypium hirsutum]	38.528	2.491	2.6	1	1	1	0.998	0.941	0.98	0.922	1.057	1.11	1.058	4.07E-01

cds.Camellia_sinensis.comp28827_c0_seq7_m.56542	PREDICTED: probable polygalacturonase isoform X1 [Nicotiana sylvestris]	13.465	3.3225	12.2	1	1	1	1.043	1.039	1.043	0.966	1.068	0.841	0.92	3.31E-01
cds.Camellia_sinensis.comp28828_c0_seq5_m.27283	photosystem I chlorophyll a/b-binding protein 5, chloroplastic [Cajanus cajan]	12.144	2.6082	8.9	1	2	1	0.92	1.032	1.017	0.964	1.059	1.025	1.027	5.87E-01
cds.Camellia_sinensis.comp28830_c0_seq1_m.25500	PREDICTED: lys-63-specific deubiquitinase BRCC36-like [Jatropha curcas]	50.006	14.061	17.6	7	7	7	1.114	1.097	1.013	0.955	0.925	0.891	0.859	1.33E-02
cds.Camellia_sinensis.comp28835_c0_seq1_m.35332	PREDICTED: 14-3-3-like protein A [Theobroma cacao]	29.421	45.168	44.8	14	51	9	0.951	0.969	0.937	1.019	1.058	1.037	1.09	4.10E-03

cds.Camellia_sinensis.comp28836_c0_seq1_m.11655	PREDICTED: formate--tetrahydrofolate ligase [Populus euphratica]	67.527	227.29	42.5	23	55	23	1.008	0.974	0.987	1.017	1.031	1.017	1.032	4.39E-02
cds.Camellia_sinensis.comp28844_c0_seq1_m.6125	PREDICTED: endo-1,4-beta-xylanase A-like [Ziziphus jujuba]	104.79	11.525	2.8	2	2	2	1.066	1.213	1.185	0.819	0.75	0.929	0.721	1.11E-02
cds.Camellia_sinensis.comp28845_c0_seq1_m.28073	PREDICTED: ubiquinol oxidase, mitochondrial-like [Prunus mume]	44.246	16.262	15.4	6	7	6	1.004	0.952	0.995	0.997	1.013	1.008	1.023	2.55E-01
cds.Camellia_sinensis.comp28846_c0_seq1_m.27141	PREDICTED: hydroxyphenylpyruvate reductase-like [Nicotiana attenuata]	34.145	17.397	20.1	6	10	6	1.003	0.988	1.002	1.027	1.011	1.034	1.026	3.43E-02

cds.Camellia_sinensis.comp28853_c0_seq4_m.31738	PREDICTED: kinesin-like protein KIN-7M, chloroplastic [Camelina sativa]	31.984	15.435	20.1	6	14	6	1.037	1.028	1.056	0.971	0.982	0.966	0.935	1.96E-03
cds.Camellia_sinensis.comp28856_c0_seq1_m.1934	PREDICTED: transformation/transcription domain-associated protein isoform X4 [Capsicum annuum]	156.62	5.6	2.3	3	3	3								
cds.Camellia_sinensis.comp28856_c1_seq1_m.467	PREDICTED: transcription-associated protein 1 isoform X1 [Vitis vinifera]	263.9	15.47	0.9	2	2	2	0.988	1.002	0.973	1.011	1.015	1.025	1.03	3.57E-02

cds.Camellia_sinensis.comp28859_c0_seq6_m.54527	PREDICTED: NEDD8-conjugating enzyme Ubc12-like isoform X2 [Ziziphus jujuba]	11.238	4.1712	17.2	3	3	3	0.985	0.96	0.956	1.07	0.998	1.05	1.075	3.46E-02
cds.Camellia_sinensis.comp28864_c0_seq1_m.15106	PREDICTED: SNF1-related protein kinase catalytic subunit alpha KIN10 [Cucumis sativus]	24.26	8.7014	13.7	3	3	3	0.987	1.016	0.991	1.064	0.987	1.028	1.028	3.06E-01
cds.Camellia_sinensis.comp28865_c0_seq1_m.27741	PREDICTED: 33 kDa ribonucleoprotein, chloroplastic [Erythranthe guttata]	36.156	83.966	20	4	8	4	1.114	1.162	1.129	0.859	0.883	0.838	0.758	1.42E-04
cds.Camellia_sinensis.comp28869_c0_seq1_m.20929	WSC domain-containing protein ARB_07867 precursor [Ziziphus jujuba]	60.625	4.5543	4.4	2	2	2	1.071	1.133	1	0.995	0.886	0.903	0.869	5.09E-02

cds.Camellia_sinensis.comp28876_c0_seq2_m.40932	dynein beta chain, ciliary protein [Arabidopsis thaliana]	20.927	15.71	38.2	6	6	6	0.979	1.016	0.957	1.078	0.899	1.085	1.037	6.32E-01
cds.Camellia_sinensis.comp28877_c0_seq1_m.15313	PREDICTED: cytosolic enolase 3 [Ipomoea nil]	39.649	5.8113	12.5	3	3	3								
cds.Camellia_sinensis.comp28877_c0_seq1_m.15314	Cytosolic enolase 3 [Morus notabilis]	14.166	2.1048	8.5	1	1	1								
cds.Camellia_sinensis.comp28885_c0_seq1_m.17355	PREDICTED: galactinol--sucrose galactosyltransferase-like [Erythranthe guttata]	69.958	41.945	16.6	12	27	12	1.116	1.108	1.108	0.872	0.874	0.865	0.784	2.06E-07
cds.Camellia_sinensis.comp28890_c0_seq2_m.42508	PREDICTED: zinc finger CCCH domain-containing protein 14-like [Juglans regia]	16.33	16.232	25.2	3	6	1	0.878	1.003	1.088	1.009	0.919	1.124	1.028	7.59E-01

cds.Camellia_sinensis.comp28891_c0_seq1_m.36773	RmlC-like cupins superfamily protein [Arabidopsis thaliana]	19.337	28.901	33	5	12	5	1.031	1.043	0.989	0.968	0.982	0.982	0.957	6.12E-02
cds.Camellia_sinensis.comp28901_c0_seq1_m.42012	potassium/sodium hyperpolarization-activated cyclic nucleotide-gated channel protein [Arabidopsis thaliana]	11.287	6.6911	24.2	3	4	3	0.953	1.04	1.008	0.967	1.04	0.976	0.994	8.72E-01
cds.Camellia_sinensis.comp28911_c0_seq2_m.47475	PREDICTED: 17.3 kDa class I heat shock protein-like [Gossypium raimondii]	17.116	74.141	46	6	16	4	1.108	1.063	1.106	0.864	0.896	0.917	0.817	7.77E-04
cds.Camellia_sinensis.comp28917_c0_seq1_m.11358	PREDICTED: phosphoenolpyruvate carboxykinase [ATP] [Ziziphus jujuba]	73.587	59.256	20.5	11	18	6	0.898	0.958	0.916	1.13	1.042	1.077	1.172	6.38E-03

cds.Camellia_sinensis.comp28917_c0_seq4_m.22810	PREDICTED: phosphoenolpyruvate carboxykinase [ATP]-like [Sesamum indicum]	53.101	2.0371	13.8	5	6	1								
cds.Camellia_sinensis.comp28918_c0_seq1_m.15356	PREDICTED: lysM domain receptor-like kinase 4 [Populus euphratica]	71.122	1.6866	1.3	1	1	1	1.025	1.032	0.947	1.03	0.983	0.989	0.999	9.97E-01
cds.Camellia_sinensis.comp28924_c0_seq8_m.8435	PREDICTED: protein STRUBBELIG-RECEPTOR FAMILY 6 [Vitis vinifera]	68.544	3.0036	1.6	1	1	1	1.175	1.199	1.237	0.738	0.785	0.809	0.646	1.42E-04
cds.Camellia_sinensis.comp28926_c0_seq1_m.15424	PREDICTED: amidophosphoribosyltransferase, chloroplastic [Ricinus communis]	65.44	45.385	9.8	5	8	5	1.006	1.009	1.034	0.986	0.994	1.016	0.983	2.33E-01

cds.Camellia_sinensis.comp28927_c0_seq2_m.8142	PREDICTED: serine/threonine-protein kinase BLUS1 [Theobroma cacao]	75.838	1.8571	1.9	1	1	1								
cds.Camellia_sinensis.comp28928_c0_seq1_m.26817	PREDICTED: aspartic proteinase nepenthesin-1 [Ziziphus jujuba]	43.084	16.425	8.8	4	6	4	1.071	1.033	1.121	0.925	0.96	0.894	0.862	9.00E-03
cds.Camellia_sinensis.comp28935_c0_seq1_m.36443	PREDICTED: proteasome subunit alpha type-3 isoform X1 [Gossypium hirsutum]	28.552	35.829	45.7	11	21	8	0.917	1.014	0.973	1.001	1.071	1.043	1.073	1.15E-01
cds.Camellia_sinensis.comp28935_c0_seq2_m.57987	PREDICTED: proteasome subunit alpha type-3 [Jatropha curcas]	11.827	31.424	40	4	6	4	0.965	0.985	0.962	1.052	1.025	1.046	1.072	2.96E-03

cds.Camellia_sinensis.comp28945_c0_seq1_m.36810	PREDICTED: elongation factor P-like [Nicotiana tabacum]	25.742	1.1763	3.4	1	1	1	1.06	1.012	1.134	0.964	0.871	0.949	0.868	3.67E-02
cds.Camellia_sinensis.comp28948_c0_seq1_m.3320	PREDICTED: protein CROWDED NUCLEI 1 [Cucumis melo]	73.4	13.392	12.9	9	10	8	1.124	1.066	1.07	0.893	0.884	0.886	0.817	3.15E-04
cds.Camellia_sinensis.comp28948_c0_seq1_m.3321	PREDICTED: protein CROWDED NUCLEI 1 isoform X2 [Vitis	58.7	3.0255	3.6	2	2	2	1.003	1.094	1.167	0.913	0.953	0.862	0.836	2.76E-02
cds.Camellia_sinensis.comp28953_c0_seq1_m.27824	PREDICTED: mediator of RNA polymerase II transcription subunit 15a isoform X2 [Fragaria vesca subsp.vesca]	44.129	1.47	2	1	1	1	1.092	1.017	1.035	0.948	0.918	0.978	0.905	2.36E-02

cds.Camellia_sinensis.comp28957_c0_seq1_m.23027	PREDICTED: SUPPRESSOR OF ABI3-5 isoform X1 [Theobroma cacao]	26.673	2.5888	5.3	1	1	1	1.033	1.009	1.116	0.974	0.925	0.939	0.899	3.57E-02
cds.Camellia_sinensis.comp28962_c0_seq1_m.23541	PREDICTED: probable protein disulfide-isomerase A6 [Nicotiana tomentosiformis]	39.858	121.32	42.4	17	38	15	1.032	0.998	1.006	0.99	1.003	1.005	0.987	3.24E-01
cds.Camellia_sinensis.comp28963_c0_seq1_m.29910	PREDICTED: uroporphyrinogen-III synthase, chloroplastic isoform X2 [Vitis vinifera]	35.281	1.1737	4.6	2	2	2	1.054	1.101	0.991	0.963	0.969	0.916	0.905	4.89E-02
cds.Camellia_sinensis.comp28964_c0_seq1_m.33040	PREDICTED: TOM1-like protein 2 [Ipomoea nil]	33.459	19.771	30.8	6	10	6	0.926	1.028	0.989	1.109	1.011	0.994	1.058	2.86E-01

cds.Camellia_sinensis.comp28964_c0_seq3_m.39000	PREDICTED: target of Myb protein 1 [Vitis vinifera]	26.211	3.372	15.6	3	5	3	1.046	0.926	1.007	0.949	0.992	1.013	0.992	8.63E-01
cds.Camellia_sinensis.comp28967_c0_seq1_m.49180	PREDICTED: heat shock cognate 70 kDa protein [Solanum lycopersicum]	22.519	4.4789	43.5	9	24	1	0.977	1.067	0.924	0.972	0.957	1.11	1.024	7.32E-01
cds.Camellia_sinensis.comp28968_c0_seq3_m.7600	PREDICTED: BEL1-like homeodomain protein 1 [Vitis vinifera]	76.287	2.9242	3.2	2	2	1								
cds.Camellia_sinensis.comp28969_c0_seq2_m.31057	PREDICTED: mRNA-decapping enzyme subunit 2 [Daucus carota subsp. sativus]	36.607	6.1684	6.5	2	3	2	0.864	1.052	0.985	1.151	0.964	1.021	1.081	3.71E-01
cds.Camellia_sinensis.comp28971_c0_seq1_m.16528	PREDICTED: cyclase-associated protein 1 [Vitis vinifera]	62.147	27.245	20.5	13	17	13	0.973	0.956	0.998	1.009	1.019	1.024	1.043	3.37E-02

cds.Camellia_sinensis.comp28975_c0_seq1_m.17459	transcriptional repressor [Arabidopsis lyrata subsp. lyrata]	67.725	143.68	29.2	16	37	16	1.015	1.016	1.003	0.986	0.988	0.987	0.976	2.82E-02
cds.Camellia_sinensis.comp28977_c0_seq3_m.38961	PREDICTED: putative WEB family protein At1g65010, chloroplastic [Malus domestica]	23.82	1.2572	6	1	1	1								
cds.Camellia_sinensis.comp28980_c0_seq40_c0_seq4_m.400	photosystem I P700 chlorophyll A apoprotein A2 (chloroplast) [Camellia crapnelliana]	82.369	264.44	20.2	11	59	11	1.013	1.013	1.02	0.97	0.99	0.976	0.964	4.70E-03
cds.Camellia_sinensis.comp28980_c0_seq4_m.401	photosystem I P700 chlorophyll A apoprotein A1 (chloroplast) [Camellia crapnelliana]	56.528	40.312	15.5	9	26	9	1.025	0.989	1.007	0.992	1.019	0.986	0.992	6.11E-01

cds.Camellia_sinensis.comp28980_c0_seq40_m.404	photosystem I P700 chlorophyll A apoprotein A1 (chloroplast) [Camellia crapnelliana]	17.031	23.712	15.9	3	12	3	1.012	1.018	1.054	0.938	0.999	0.954	0.937	4.61E-02
cds.Camellia_sinensis.comp28981_c0_seq2_m.32966	PREDICTED: protein trichome birefringence-like 38 [Sesamum indicum]	44.457	26.795	14.4	4	7	4	1.11	1.146	1.138	0.863	0.872	0.88	0.77	2.01E-05
cds.Camellia_sinensis.comp28983_c1_seq1_m.15218	PREDICTED: selenoprotein O [Jatropha curcas]	73.473	101.58	37.3	23	32	23	1.022	1.006	1.004	0.96	1.014	1.007	0.983	3.95E-01
cds.Camellia_sinensis.comp28984_c0_seq1_m.44429	PREDICTED: cysteine proteinase inhibitor-like [Populus euphratica]	13.513	76.668	33.1	6	19	2	1.116	1.075	1.204	0.821	0.905	0.852	0.759	3.28E-03
cds.Camellia_sinensis.comp28984_c0_seq2_m.46176	PREDICTED: cysteine proteinase inhibitor [Ricinus communis]	11.614	63.439	36.8	5	16	1	0.95	0.895	0.981	1.032	1.12	1.068	1.139	2.19E-02

cds.Camellia_sinensis.comp28990_c0_seq2_m.47115	PREDICTED: protein Dr1 homolog isoform X4 [Theobroma cacao]	16.372	12.943	24.7	3	3	3	0.932	0.896	0.992	1.125	1.009	1.054	1.13	4.75E-02
cds.Camellia_sinensis.comp28996_c0_seq1_m.8832	PREDICTED: protein FAM188A [Theobroma cacao]	67.43	61.132	18.6	9	12	9	0.89	0.973	0.89	1.108	1.026	1.132	1.186	1.54E-02
cds.Camellia_sinensis.comp28997_c0_seq1_m.29127	PREDICTED: eukaryotic translation initiation factor 3 subunit I [Cucumis sativus]	35.757	68.283	35.3	11	20	11	0.995	0.974	0.976	1.034	1.06	1.025	1.059	9.14E-03
cds.Camellia_sinensis.comp29003_c0_seq1_m.3215	PREDICTED: ubiquitin carboxyl-terminal hydrolase 26 [Vitis vinifera]	122.66	1.2168	0.7	1	1	1								

cds.Camellia_sinensis.comp29004_c0_seq1_m.28540	PREDICTED: putative septum site-determining protein minD homolog, chloroplastic [Ziziphus jujuba]	36.21	11.526	10.6	3	3	3	0.936	1.018	1.065	1.016	0.916	0.988	0.967	5.38E-01
cds.Camellia_sinensis.comp29005_c0_seq1_m.10811	PREDICTED: D-3-phosphoglycerate dehydrogenase 2, chloroplastic-like [Nicotiana attenuata]	71.371	74.516	22.4	14	23	12	1.084	1.108	1.082	0.881	0.888	0.901	0.816	3.75E-05
cds.Camellia_sinensis.comp29007_c0_seq1_m.6485	PREDICTED: protein GRIP [Juglans regia]	91.598	2.2951	1.9	1	1	1								
cds.Camellia_sinensis.comp29009_c0_seq1_m.6382	PREDICTED: THO complex subunit 5A isoform X2 [Vitis vinifera]	58.935	1.67	1.9	1	1	1								

cds.Camellia_sinensis.comp29009_c2_seq1_m.33399	PREDICTED: translation machinery-associated protein 22 [Populus euphratica]	22.946	2.5605	11.2	2	2	2	1.113	1.116	0.976	0.946	0.878	0.951	0.866	4.94E-02
cds.Camellia_sinensis.comp29011_c0_seq1_m.33325	PREDICTED: ACT domain-containing protein ACR12 [Juglans regia]	34.168	30.961	18.8	6	11	6	1.015	1.012	0.996	1.004	0.995	0.99	0.989	1.90E-01
cds.Camellia_sinensis.comp29013_c0_seq1_m.15937	PREDICTED: TPR repeat-containing thioredoxin TDX isoform X2 [Malus domestica]	44.496	34.283	15.1	7	9	7	1.135	1.116	1.139	0.854	0.893	0.867	0.771	5.88E-05
cds.Camellia_sinensis.comp29013_c0_seq1_m.15938	PREDICTED: ubiquitin receptor RAD23c-like [Vigna	17.285	55.608	33.7	3	7	3	1.072	0.946	0.963	0.979	1.035	1.003	1.012	7.69E-01

cds.Camellia_sinensis.comp29016_c0_seq1_m.12430	PREDICTED: cysteine--tRNA ligase 2, cytoplasmic [Nelumbo nucifera]	60.574	41.197	12	6	7	6	0.975	1.023	0.944	1.046	0.997	0.98	1.028	4.20E-01
cds.Camellia_sinensis.comp29019_c0_seq1_m.35179	PREDICTED: U1 small nuclear ribonucleoprotein A-like [Ziziphus jujuba]	27.341	10.732	13.9	3	3	3	1.056	1.003	1.117	1	0.901	0.938	0.894	6.13E-02
cds.Camellia_sinensis.comp29020_c0_seq1_m.11266	PREDICTED: threonine--tRNA ligase, chloroplastic/mitochondrial 2 [Vitis vinifera]	77.745	25.814	13.1	8	10	8	0.998	0.973	0.961	1.067	1.042	1.014	1.065	2.69E-02
cds.Camellia_sinensis.comp29023_c0_seq3_m.29152	lipoxygenase [Vitis vinifera]	52.425	1.3242	1.7	1	1	1	1.107	1.124	1.06	0.901	0.935	0.85	0.816	3.44E-03

cds.Camellia_sinensis.comp29026_c0_seq1_m.42170	PREDICTED: probable glutathione S-transferase [Populus euphratica]	25.622	1.2231	5.4	1	1	1								
cds.Camellia_sinensis.comp29028_c0_seq1_m.20116	PREDICTED: ras-related protein Rab11D [Vitis vinifera]	24.928	5.7706	26.7	6	8	5	0.997	1.05	1.05	0.952	0.919	0.933	0.905	7.80E-03
cds.Camellia_sinensis.comp29032_c0_seq1_m.32431	PREDICTED: proteasome subunit beta type-2-A-like [Jatropha curcas]	22.685	55.433	36.6	8	13	3	0.979	1.024	0.987	1.011	1.035	1.032	1.029	1.36E-01
cds.Camellia_sinensis.comp29032_c0_seq2_m.54937	PREDICTED: proteasome subunit beta type-2-B [Ipomoea nil]	15.189	3.3625	42.8	6	10	1	0.94	0.96	0.989	1.01	1.104	1.022	1.085	6.12E-02
cds.Camellia_sinensis.comp29039_c0_seq1_m.39236	PREDICTED: ubiquitin-conjugating enzyme E2 variant 1D [Sesamum indicum]	16.562	77.395	58.9	8	21	8	1.01	0.973	0.989	1.002	1.041	1.017	1.03	1.33E-01

cds.Camellia_sinensis.comp29046_c0_seq1_m.29078	PREDICTED: aldo-keto reductase family 4 member C9-like [Gossypium arboreum]	35.072	8.9395	14.6	6	9	6	1.039	0.958	0.997	1.011	1.032	0.997	1.015	5.71E-01
cds.Camellia_sinensis.comp29048_c0_seq1_m.26319	PREDICTED: myb-related protein Myb4 [Theobroma cacao]	42.111	1.5658	3.1	1	1	1	1.057	0.85	0.991	1.033	1.023	1.058	1.075	3.66E-01
cds.Camellia_sinensis.comp29052_c0_seq1_m.19282	PREDICTED: cell division control protein 7 [Ziziphus jujuba]	31.035	1.7548	4	1	2	1								
cds.Camellia_sinensis.comp29052_c0_seq1_m.19283	PREDICTED: E3 ubiquitin-protein ligase KEG [Sesamum indicum]	28.627	1.5985	5.1	1	1	1								
cds.Camellia_sinensis.comp29064_c0_seq1_m.4735	PREDICTED: protein transport protein SEC23 [Vitis vinifera]	95.394	8.4967	4.3	4	4	4	0.89	1.028	0.976	1.151	0.964	1.037	1.089	2.73E-01

cds.Camellia_sinensis.comp29074_c0_seq1_m.15684	PREDICTED: enolase-phosphatase E1 [Vitis vinifera]	54.971	3.253	2.1	1	2	1	1.079	0.986	0.953	0.943	1.037	0.999	0.987	8.02E-01
cds.Camellia_sinensis.comp29075_c0_seq1_m.18691	PREDICTED: inactive dipeptidyl peptidase 10-like isoform X1 [Daucus carota subsp. sativus]	66.517	135.54	27.8	16	24	16	0.94	0.931	0.936	1.086	1.064	1.074	1.149	2.47E-05
cds.Camellia_sinensis.comp29080_c0_seq1_m.18560	PREDICTED: chorismate synthase 1, chloroplastic [Vitis vinifera]	47.258	9.5773	16.5	8	11	8	1.056	1.116	1.071	0.878	0.891	0.921	0.829	1.02E-03
cds.Camellia_sinensis.comp29085_c0_seq1_m.49093	PREDICTED: 10 kDa chaperonin-like [Sesamum indicum]	13.338	57.867	54.9	7	23	7	1.023	0.996	0.996	0.953	0.998	1.028	0.988	6.29E-01

cds.Camellia_sinensis.comp29098_c0_seq1_m.46314	PREDICTED: 10 kDa chaperonin 1, chloroplastic [Nicotiana attenuata]	15.39	47.222	43.7	6	16	6	1.008	1.03	1.047	0.972	0.982	0.956	0.943	1.24E-02
cds.Camellia_sinensis.comp29099_c0_seq1_m.24039	alcohol dehydrogenase family protein [Populus trichocarpa]	41.861	21.811	10.6	4	5	4	1.024	1.047	1.051	0.929	0.92	0.94	0.893	3.82E-04
cds.Camellia_sinensis.comp29105_c0_seq1_m.19191	PREDICTED: probable nucleoredoxin 2 isoform X1 [Ricinus communis]	34.772	2.0821	3.3	1	1	1	0.962	1.009	1.003	0.955	1.02	1.062	1.021	5.88E-01
cds.Camellia_sinensis.comp29108_c0_seq5_m.42787	PREDICTED: vacuolar protein sorting-associated protein 32 homolog 2 [Cucumis melo]	23.571	20.332	38.5	8	8	2	1.148	1.106	1.084	0.9	0.794	0.935	0.788	9.90E-03

cds.Camellia_sinensis.comp29111_c0_seq2_m.42119	PREDICTED: transmembrane emp24 domain-containing protein p24delta9 [Citrus sinensis]	24.489	1.336	6.5	2	3	2	0.882	0.99	0.926	1.113	0.998	1.127	1.157	4.63E-02
cds.Camellia_sinensis.comp29117_c0_seq1_m.25011	PREDICTED: rhomboid-like protein 15 [Juglans regia]	46.147	5.4179	5.9	2	3	2	1.003	1.025	1.072	1.061	0.947	0.901	0.938	2.77E-01
cds.Camellia_sinensis.comp29118_c0_seq1_m.28793	PREDICTED: caffeoylshikimate esterase-like isoform X1 [Nicotiana tomentosiformis]	43.038	10.777	8.2	4	4	4	0.972	0.988	1.003	1.006	1.045	1.002	1.03	1.39E-01
cds.Camellia_sinensis.comp29121_c0_seq1_m.33698	PREDICTED: thioredoxin-like protein AAED1, chloroplastic isoform X2 [Vitis vinifera]	27.749	66.761	30.8	6	13	6	0.977	0.993	0.992	0.977	1.026	1.014	1.019	3.09E-01

cds.Camellia_sinensis.comp29122_c0_seq1_m.32494	PREDICTED: bark storage protein A-like [Juglans regia]	36.404	3.7657	4.2	1	1	1	0.887	1.31	1.103	0.882	0.898	0.913	0.816	2.34E-01
cds.Camellia_sinensis.comp29124_c0_seq1_m.29557	PREDICTED: PI-PLC X domain-containing protein At5g67130 [Arachis duranensis]	41.926	1.7781	4.4	2	2	2	0.945	1.06	0.979	1.076	0.975	0.984	1.017	7.30E-01
cds.Camellia_sinensis.comp29127_c0_seq2_m.27072	PREDICTED: pyruvate dehydrogenase E1 component subunit beta-2, mitochondrial [Setaria	18.566	34.233	49.4	8	21	5	0.991	1.049	1.002	0.998	0.968	0.999	0.975	2.77E-01
cds.Camellia_sinensis.comp29128_c0_seq1_m.17628	PREDICTED: homeobox protein knotted-1-like 13 isoform X3 [Vitis vinifera]	34.562	3.8194	5.9	2	2	2	1.071	1.169	1.038	1.023	0.853	0.833	0.826	5.90E-02

cds.Camellia_sinensis.comp29131_c0_seq1_m.37347	PREDICTED: photosynthetic NDH subunit of lumenal location 4, chloroplastic [Vitis vinifera]	25.654	33.935	26.7	5	10	5	0.929	1.018	1.048	0.949	0.979	1.073	1.002	9.72E-01
cds.Camellia_sinensis.comp29132_c0_seq2_m.16512	PREDICTED: molybdopterin biosynthesis protein CNX1 [Jatropha curcas]	52.017	41.384	9.2	4	6	4	0.975	0.933	0.92	1.051	1.043	1.1	1.129	7.24E-03
cds.Camellia_sinensis.comp29136_c0_seq1_m.23393	PREDICTED: UDP-glucuronic acid decarboxylase 2 [Theobroma cacao]	48.526	6.7826	13.7	6	9	4	0.921	1.08	1.061	1.062	0.997	0.899	0.966	6.49E-01
cds.Camellia_sinensis.comp29143_c0_seq1_m.17411	Mitochondrial metalloendopeptidase OMA1 [Morus notabilis]	50.212	1.2386	1.8	1	2	1	0.926	1.027	0.984	1.021	1.021	1.041	1.05	1.83E-01

cds.Camellia_sinensis.comp29144_c0_seq1_m.14277	PREDICTED: multicopper oxidase LPR1 [Jatropha curcas]	67.314	25.464	11.1	5	9	5	1	1.048	0.904	1.033	0.998	0.991	1.024	6.09E-01
cds.Camellia_sinensis.comp29145_c0_seq1_m.13882	PREDICTED: L-galactono-1,4-lactone dehydrogenase, mitochondrial [Sesamum indicum]	50.886	100.67	23.7	9	14	9	1.012	1.002	1.006	0.978	1.023	0.955	0.979	3.96E-01
cds.Camellia_sinensis.comp29148_c0_seq1_m.35252	PREDICTED: chlorophyll a-b binding protein of LHCII type 1-like [Ziziphus jujuba]	36.449	17.289	12	4	5	4	0.986	0.977	0.976	1.06	1.04	1.057	1.074	4.56E-04
cds.Camellia_sinensis.comp29152_c0_seq1_m.21822	tetratricopeptide repeat-containing protein isoform 2 [Solanum lycopersicum]	48.627	1.5719	2.1	1	1	1								

cds.Camellia_sinensis.comp29155_c0_seq1_m.33111	transmembrane protein, putative [Medicago truncatula]	32.567	8.9467	5.8	1	2	1	0.909	1.012	0.986	1.008	1.192	0.923	1.074	4.58E-01
cds.Camellia_sinensis.comp29156_c0_seq1_m.32125	ATP-dependent Clp protease proteolytic subunit 5 [Morus notabilis]	38.3	35.914	21.1	6	15	6	0.943	0.979	0.945	1.063	1.05	1.01	1.089	1.21E-02
cds.Camellia_sinensis.comp29158_c0_seq1_m.36360	PREDICTED: stearoyl-[acyl-carrier-protein] 9-desaturase, chloroplastic [Theobroma cacao]	40.653	34.636	16	5	5	5	0.917	0.937	0.971	1.104	1.031	1.056	1.13	9.54E-03
cds.Camellia_sinensis.comp29158_c0_seq2_m.57673	PREDICTED: stearoyl-[acyl-carrier-protein] 9-desaturase, chloroplastic isoform X2 [Nelumbo nucifera]	12.895	15.905	23.9	2	2	2	0.974	1.033	0.91	1.057	1.049	0.996	1.063	2.03E-01

cds.Camellia_sinensis.comp29164_c0_seq1_m.34097	PREDICTED: chlorophyll a-b binding protein CP26, chloroplastic [Jatropha curcas]	30.956	191.41	58.6	14	89	14	0.924	0.91	0.918	1.086	1.1	1.091	1.191	3.62E-06
cds.Camellia_sinensis.comp29166_c0_seq2_m.33330	PREDICTED: probable enoyl-CoA hydratase 2, mitochondrial [Juglans regia]	24.479	18.257	16.2	2	2	2	0.992	1.067	0.952	0.961	0.97	1.061	0.994	8.99E-01
cds.Camellia_sinensis.comp29167_c0_seq1_m.10952	PREDICTED: RNA polymerase II degradation factor 1 [Vitis vinifera]	76.05	43.258	7.2	4	5	4	1.073	1.034	1.047	0.965	0.946	1.04	0.936	9.46E-02
cds.Camellia_sinensis.comp29169_c0_seq1_m.32558	PREDICTED: fatty acid desaturase 4, chloroplastic [Fragaria vesca subsp. vesca]	34.715	2.8916	5.5	1	1	1								

cds.Camellia_sinensis.comp29172_c0_seq2_m.27857	PREDICTED: protein FAR1-RELATED SEQUENCE 9 [Vitis	46.018	9.647	6.3	2	2	2	0.926	0.978	0.965	1.136	0.965	1.061	1.102	1.30E-01
cds.Camellia_sinensis.comp29174_c0_seq1_m.31554	PREDICTED: RNA-binding protein CP29B, chloroplastic [Vitis vinifera]	37.304	54.173	19.5	6	11	6	1.104	1.089	1.115	0.92	0.911	0.865	0.815	5.41E-04
cds.Camellia_sinensis.comp29178_c0_seq1_m.22814	PREDICTED: thiosulfate/3-mercaptopyruvate sulfurtransferase 1, mitochondrial isoform X1 [Jatropha	41.559	95.763	38.3	12	27	12	0.948	0.96	0.974	1.029	1.071	1.031	1.086	5.52E-03
cds.Camellia_sinensis.comp29182_c0_seq1_m.10746	PREDICTED: elongation factor G-1, mitochondrial [Theobroma cacao]	84.612	2.5142	2.2	2	2	2	0.913	1.02	0.928	1.044	1.146	0.979	1.108	1.52E-01

cds.Camellia_sinensis.comp29184_c0_seq2_m.31750	PH-response transcription factor [Arabidopsis thaliana]	38.237	18.251	15.7	5	8	5	0.951	0.991	0.977	0.991	0.997	1.1	1.058	1.99E-01
cds.Camellia_sinensis.comp29186_c0_seq1_m.24270	PREDICTED: AP-4 complex subunit mu isoform X1 [Daucus carota subsp. sativus]	52.844	5.2092	7.6	3	4	3	0.968	1.034	1.01	1.108	0.926	0.973	0.998	9.45E-01
cds.Camellia_sinensis.comp29188_c0_seq1_m.28848	PREDICTED: spermidine synthase 1 [Citrus sinensis]	39.312	25.797	11.5	3	5	2	0.958	1.054	1.062	0.997	0.962	1.108	0.998	9.58E-01
cds.Camellia_sinensis.comp29188_c0_seq2_m.30395	PREDICTED: spermidine synthase [Theobroma cacao]	39.351	4.1685	6.7	2	3	1	1.044	1.095	1.039	0.947	0.991	0.877	0.886	3.61E-02
cds.Camellia_sinensis.comp29189_c0_seq1_m.26715	PREDICTED: superoxide dismutase [Mn], mitochondrial [Nicotiana attenuata]	25.54	66.201	33.9	7	37	7	0.991	0.945	0.942	1.024	1.046	1.041	1.081	1.14E-02

cds.Camellia_sinensis.comp29198_c0_seq1_m.29186	PREDICTED: quinone oxidoreductase-like protein 2 homolog [Solanum pennellii]	36.851	4.3385	8	3	4	3	1.03	1.099	0.984	0.913	0.943	1.022	0.925	1.66E-01
cds.Camellia_sinensis.comp29200_c0_seq2_m.48153	PREDICTED: tubulin-folding cofactor A [Gossypium hirsutum]	12.801	54.936	41.6	5	9	5	0.965	0.981	0.936	1.038	1.054	1.032	1.084	5.92E-03
cds.Camellia_sinensis.comp29207_c0_seq2_m.18646	PREDICTED: protein FREE1 [Vitis vinifera]	59.644	19.187	8.7	3	3	3	0.981	1.031	1.082	0.994	0.917	0.998	0.94	1.94E-01
cds.Camellia_sinensis.comp29208_c0_seq3_m.32955	PREDICTED: plant UBX domain-containing protein 4 [Eucalyptus grandis]	34.989	16.688	17.6	4	4	4	0.982	1.083	0.911	0.994	1.013	1.027	1.019	6.92E-01
cds.Camellia_sinensis.comp29220_c0_seq1_m.35024	PREDICTED: tRNA (guanine-N(7)-)-methyltransferase [Citrus sinensis]	28.754	1.9582	4.4	1	1	1								

cds.Camellia_sinensis.comp29221_c0_seq1_m.35565	PREDICTED: trigger factor-like [Phoenix dactylifera]	24.688	59.672	26.9	6	12	6	1.036	1.028	1.079	0.953	0.94	0.939	0.901	2.72E-03
cds.Camellia_sinensis.comp29223_c0_seq1_m.29121	PREDICTED: UDP-glucuronic acid decarboxylase 6 [Nelumbo nucifera]	38.379	50.808	33.2	11	21	6	1.011	0.964	0.973	1.015	1.074	1.013	1.052	1.03E-01
cds.Camellia_sinensis.comp29223_c0_seq2_m.37257	PREDICTED: UDP-glucuronic acid decarboxylase 6 [Arachis ipaensis]	28.743	1.2219	20.1	6	10	1								
cds.Camellia_sinensis.comp29226_c0_seq2_m.38445	PREDICTED: endo-1,3;1,4-beta-D-glucanase-like, partial [Nicotiana tomentosiformis]	18.239	8.7447	12.2	2	3	2	1.075	0.946	1.011	0.996	0.983	0.989	0.979	6.44E-01

cds.Camellia_sinensis.comp29238_c0_seq1_m.38345	PREDICTED: CBS domain-containing protein CBSX3, mitochondrial [Vitis vinifera]	22.649	35.65	50.7	9	24	9	0.949	0.994	0.993	1.027	1.03	1.02	1.048	3.87E-02
cds.Camellia_sinensis.comp29239_c0_seq1_m.40043	PREDICTED: probable carboxylesterase 2 [Vitis vinifera]	19.798	4.2994	18.5	3	6	0								
cds.Camellia_sinensis.comp29239_c0_seq2_m.40683	PREDICTED: probable carboxylesterase 2 [Vitis vinifera]	20.772	42.738	27	5	10	3	0.987	0.943	0.919	1.063	1.053	1.048	1.111	7.78E-03
cds.Camellia_sinensis.comp29239_c0_seq3_m.41918	PREDICTED: probable carboxylesterase 2 [Vitis vinifera]	28.937	2.1282	14.8	3	5	1								
cds.Camellia_sinensis.comp29242_c0_seq1_m.33354	Thylakoid lumenal 19 kDa protein [Morus notabilis]	27.926	50.819	16.2	6	15	6	1.013	0.992	0.972	1.011	1.027	0.997	1.019	2.58E-01

cds.Camellia_sinensis.comp29247_c0_seq2_m.21215	PREDICTED: alcohol dehydrogenase-like [Nicotiana tabacum]	47.038	152.2	23.6	8	16	8	0.984	0.97	0.984	1.028	1.032	1.019	1.048	1.52E-03
cds.Camellia_sinensis.comp29250_c0_seq1_m.23795	PREDICTED: probable mitochondrial saccharopine dehydrogenase-like oxidoreductase At5g39410 isoform X1[Jatropha curcas]	48.98	12.16	13.4	5	5	5	1.015	1.048	1.039	0.906	0.983	1.006	0.933	1.02E-01
cds.Camellia_sinensis.comp29251_c0_seq1_m.4569	PREDICTED: NAD(P)H-quinone oxidoreductase subunit N, chloroplastic-like [Nicotiana tabacum]	19.834	4.0794	21.5	4	4	4	0.953	1.026	1.036	0.981	1.102	0.918	0.995	9.14E-01
cds.Camellia_sinensis.comp29256_c0_seq1_m.4724	PREDICTED: alanine--tRNA ligase [Vitis vinifera]	112.52	207.71	28.2	28	53	28	0.985	0.982	0.975	1.006	1.014	1.037	1.039	1.62E-02

cds.Camellia_sinensis.comp29258_c0_seq1_m.43491	PREDICTED: universal stress protein PHOS32 [Pyrus x bretschneideri]	18.455	2.5107	18.3	4	6	3	0.969	0.98	0.975	1.043	1.046	1.007	1.059	1.07E-02
cds.Camellia_sinensis.comp29266_c0_seq1_m.27706	Dehydration-responsive protein RD22 precursor [Populus trichocarpa]	12.51	42.2	31.7	5	7	5	1.259	1.173	1.193	0.721	0.8	0.794	0.639	3.44E-04
cds.Camellia_sinensis.comp29266_c0_seq2_m.32997	PREDICTED: BURP domain protein RD22-like, partial [Juglans regia]	22.294	8.7869	16.2	4	4	4	1.34	1.282	1.299	0.638	0.669	0.656	0.501	1.70E-06
cds.Camellia_sinensis.comp29270_c0_seq2_m.14589	PREDICTED: glucose-6-phosphate isomerase 1, chloroplastic [Ziziphus jujuba]	68.855	197.81	49.4	29	45	29	1.003	0.997	1.025	0.991	0.995	1.002	0.988	2.46E-01

cds.Camellia_sinensis.comp29274_c0_seq1_m.16753	PREDICTED: succinate-semialdehyde dehydrogenase, mitochondrial [Prunus	56.659	64.941	28.6	15	32	15	0.945	0.964	0.953	1.072	1.075	1.047	1.116	4.19E-04
cds.Camellia_sinensis.comp29282_c0_seq1_m.45480	PREDICTED: heparanase-like protein 3 [Vitis vinifera]	22.451	4.1598	9	2	3	2	1.016	1.053	1.041	1.041	0.919	0.933	0.93	1.44E-01
cds.Camellia_sinensis.comp29286_c0_seq2_m.25516	alpha/beta fold hydrolase [Medicago truncatula]	41.881	29.689	17.3	6	8	6	1.008	0.977	1.007	0.999	1.025	0.997	1.01	5.16E-01
cds.Camellia_sinensis.comp29287_c0_seq6_m.49241	histone-lysine N-methyltransferase [Arabidopsis thaliana]	14.982	1.9029	7.2	1	1	1								
cds.Camellia_sinensis.comp29290_c0_seq2_m.14601	PREDICTED: serine/threonine-protein kinase STN8, chloroplastic isoform X1 [Vitis vinifera]	57.13	47.976	20.2	10	10	10	0.98	1.004	1.005	1.035	1.006	0.998	1.017	2.96E-01

cds.Camellia_sinensis.comp29292_c0_seq4_m.36482	PREDICTED: TATA-box-binding protein-like [Nicotiana tabacum]	28.745	1.4681	4	1	4	1	1.088	1.087	1.061	0.93	0.914	0.948	0.863	3.82E-04
cds.Camellia_sinensis.comp29295_c0_seq1_m.32773	PREDICTED: phosphoglucan phosphatase DSP4, amyloplastic [Ziziphus jujuba]	22.498	6.0198	19.6	3	3	2								
cds.Camellia_sinensis.comp29297_c0_seq2_m.16897	PREDICTED: NEDD8 ultimate buster 1 [Solanum tuberosum	61.841	12.847	10.9	6	8	6	1.03	0.998	1.02	0.97	1.028	0.997	0.983	4.08E-01
cds.Camellia_sinensis.comp29297_c0_seq3_m.18716	PREDICTED: aspartic proteinase [Vitis vinifera]	58.91	11.975	9.1	5	7	5	0.952	0.965	0.924	1.043	1.054	1.045	1.106	1.58E-03
cds.Camellia_sinensis.comp29299_c0_seq1_m.33168	26S protease regulatory subunit 6B-like protein [Morus notabilis]	38.558	5.1123	22.4	9	14	2	0.906	1.043	1.051	1.017	0.966	1.037	1.007	8.80E-01

cds.Camellia_sinensis.comp29301_c0_seq1_m.28275	RNA binding protein [Arabidopsis thaliana]	45.52	6.8912	6.6	2	2	2								
cds.Camellia_sinensis.comp29307_c0_seq1_m.11343	PREDICTED: protein SIEVE ELEMENT OCCLUSION B [Ricinus communis]	48.293	53.183	32.4	14	20	14	0.944	0.996	0.994	1.049	1.027	1.033	1.06	3.55E-02
cds.Camellia_sinensis.comp29307_c0_seq1_m.11344	PREDICTED: protein SIEVE ELEMENT OCCLUSION B [Ricinus communis]	35.78	34.291	25.2	6	9	6	0.951	0.944	0.977	1.085	1.101	1.024	1.118	1.06E-02
cds.Camellia_sinensis.comp29308_c0_seq2_m.24888	PREDICTED: manganese-dependent ADP-ribose/CDP-alcohol diphosphatase [Vitis vinifera]	35.925	3.4379	9.4	3	3	3	0.896	1.026	0.992	1.033	0.995	1.082	1.067	2.35E-01

cds.Camellia_sinensis.comp29319_c1_seq1_m.24228	PREDICTED: alcohol dehydrogenase [Citrus sinensis]	43.146	10.535	14.4	6	11	5	0.961	0.962	0.977	1.028	1.035	1.06	1.077	2.30E-03
cds.Camellia_sinensis.comp29326_c0_seq1_m.19201	PREDICTED: putative G3BP-like protein [Nelumbo nucifera]	51.175	29.607	22.1	10	13	10	1.056	1.119	1.074	0.908	0.898	0.964	0.853	4.64E-03
cds.Camellia_sinensis.comp29333_c0_seq1_m.15486	PREDICTED: molybdate-anion transporter [Citrus sinensis]	50.72	1.8137	3.5	2	2	2	1.067	1.125	1.108	0.881	0.899	0.898	0.812	2.58E-04
cds.Camellia_sinensis.comp29334_c0_seq1_m.21420	EMB2737 [Arabidopsis lyrata subsp. lyrata]	46.009	1.4159	2	1	1	1	0.997	1.055	1.005	1.064	0.927	0.962	0.966	4.67E-01
cds.Camellia_sinensis.comp29337_c0_seq1_m.17938	Ferredoxin--nitrite reductase [Morus notabilis]	56.402	67.988	20.4	11	17	11	0.966	0.97	0.976	1.065	1.041	1.039	1.08	8.20E-04
cds.Camellia_sinensis.comp29343_c0_seq1_m.39591	TPR domain protein [Medicago truncatula]	23.697	16.116	24.4	5	7	5	0.974	0.951	0.908	1.049	1.033	1.125	1.132	2.07E-02

cds.Camellia_sinensis.comp29357_c0_seq1_m.14976	PREDICTED: asparagine--tRNA ligase, cytoplasmic 1 [Vitis vinifera]	63.182	123.47	24.7	14	31	12	0.943	0.976	0.961	1.08	1.03	1.035	1.092	8.22E-03
cds.Camellia_sinensis.comp29358_c0_seq1_m.36435	PREDICTED: 60S ribosomal protein L9 [Cucumis melo]	23.154	78.166	54.2	10	22	10	1.197	1.165	1.142	0.8	0.856	0.83	0.709	1.37E-04
cds.Camellia_sinensis.comp29362_c0_seq2_m.33302	PREDICTED: bifunctional phosphatase IMPL2, chloroplastic-like isoform X1 [Nicotiana tabacum]	37.645	38.799	12.9	3	5	3	0.922	0.963	1.034	1.094	0.996	0.977	1.051	3.65E-01
cds.Camellia_sinensis.comp29363_c0_seq1_m.13525	PREDICTED: pentatricopeptide repeat-containing protein At2g15690 [Solanum lycopersic	74.623	3.3549	3.6	2	2	1	0.983	1.027	1.037	0.948	0.952	1.056	0.97	4.66E-01

cds.Camellia_sinensis.comp29363_c0_seq2_m.22606	PREDICTED: pentatricopeptide repeat-containing protein At2g15690-like [Vitis	53.849	8.2248	6.5	3	3	2	0.956	1.017	1.102	0.918	0.838	1.167	0.951	6.11E-01
cds.Camellia_sinensis.comp29364_c0_seq1_m.23336	PREDICTED: tyrosine aminotransferase-like [Nicotiana attenuata]	46.995	38.035	17.1	7	9	7	1.101	1.111	1.143	0.87	0.889	0.879	0.786	4.43E-05
cds.Camellia_sinensis.comp29365_c0_seq1_m.8543	PREDICTED: probable beta-D-xylosidase 7 isoform X2 [Theobroma cacao]	85.647	15.054	11	8	9	8	1.008	1.011	1.022	1.036	0.961	0.964	0.974	3.35E-01
cds.Camellia_sinensis.comp29366_c0_seq1_m.9691	PREDICTED: WEB family protein At5g55860 [Vitis vinifera]	71.843	33.42	18.7	12	13	12	0.95	0.994	0.991	0.966	1.055	1.021	1.036	2.98E-01

cds.Camellia_sinensis.comp29370_c0_seq2_m.43077	PREDICTED: probable xyloglucan endotransglucosylase/hydrolase protein 23 [Vitis vinifera]	25.9	23.438	21.6	4	5	3	0.809	0.877	0.874	1.182	1.085	1.234	1.368	2.48E-03
cds.Camellia_sinensis.comp29371_c0_seq1_m.7891	PREDICTED: phospholipid:diacylglycerol acyltransferase 1-like [Jatropha curcas]	74.118	25.569	5.4	4	5	4	0.974	0.907	1.005	0.988	1.062	1.082	1.085	1.17E-01
cds.Camellia_sinensis.comp29373_c0_seq2_m.37564	PREDICTED: vacuolar protein-sorting-associated protein 37 homolog 1-like [Gossypium hirsutum]	29.518	5.9198	12.9	4	8	4	1.028	0.998	0.977	0.995	0.964	1.053	1.003	9.36E-01

cds.Camellia_sinensis.comp29378_c0_seq1_m.36536	PREDICTED: mitochondrial outer membrane import complex protein METAXIN [Vitis vinifera]	38.229	2.5981	5.3	2	2	2	0.982	1.057	1.013	0.999	0.953	1.004	0.969	3.05E-01
cds.Camellia_sinensis.comp29380_c0_seq1_m.39114	PITH domain-containing protein [Morus notabilis]	20.83	18.845	11.2	2	4	2	0.936	0.991	0.978	1.054	1.035	1.073	1.088	1.35E-02
cds.Camellia_sinensis.comp29387_c0_seq1_m.10368	PREDICTED: acyltransferase-like protein At3g26840, chloroplastic [Vitis vinifera]	70.567	2.2824	4	2	2	2								
cds.Camellia_sinensis.comp29390_c0_seq1_m.22240	PREDICTED: linolenate hydroperoxide lyase, chloroplastic [Fragaria vesca subsp. vesca]	54.807	56.694	22.6	11	20	11	1.006	0.977	0.962	1.044	1.03	1.014	1.049	3.81E-02

cds.Camellia_sinensis.comp29392_c1_seq2_m.18187	PREDICTED: dnaJ protein ERDJ3B [Nelumbo nucifera]	38.646	10.358	16.6	4	6	4	1.097	1.028	1.036	0.948	0.953	0.907	0.888	1.02E-02
cds.Camellia_sinensis.comp29394_c0_seq1_m.5515	PREDICTED: protein TIC110, chloroplastic isoform X1 [Vitis vinifera]	69.71	143.18	33.4	18	35	18	0.995	1.017	1.014	0.995	0.992	1.004	0.988	2.09E-01
cds.Camellia_sinensis.comp29394_c0_seq1_m.5516	PREDICTED: protein TIC110, chloroplastic-like, partial [Nicotiana tabacum]	24.176	45.791	38.8	9	17	9	1	1.001	1.013	0.968	1.007	0.966	0.976	1.56E-01
cds.Camellia_sinensis.comp29394_c0_seq1_m.5517	PREDICTED: protein TIC110, chloroplastic, partial [Ziziphus jujuba]	22.297	90.416	42	6	23	6	1.001	1.011	1.008	0.994	0.99	0.991	0.985	9.24E-03

cds.Camellia_sinensis.comp29395_c0_seq1_m.21621	PREDICTED: isocitrate dehydrogenase [NADP], chloroplastic/mitochondrial [Citrus sinensis]	54.187	54.55	27.9	13	16	12	0.997	1.003	0.987	0.999	1.023	0.998	1.011	3.07E-01
cds.Camellia_sinensis.comp29397_c0_seq2_m.33702	Minor allergen Alt a 7 [Morus notabilis]	26.511	21.708	19	4	6	3	1.022	0.966	0.964	0.982	1.097	0.981	1.037	4.50E-01
cds.Camellia_sinensis.comp29397_c0_seq4_m.45655	PREDICTED: probable NAD(P)H dehydrogenase (quinone) FQR1-like 2 [Solanum pennellii]	20.132	3.0136	13.3	2	3	1								
cds.Camellia_sinensis.comp29398_c0_seq1_m.19933	PREDICTED: 26S protease regulatory subunit 6A homolog [Arachis ipaensis]	47.429	269.11	64.1	24	55	23	0.954	0.993	0.993	1.017	1.019	1.035	1.045	3.90E-02

cds.Camellia_sinensis.comp29403_c0_seq1_m.1874	PREDICTED: sucrose synthase [Nicotiana tabacum]	92.452	93.255	23.2	18	25	15	1.152	1.168	1.246	0.763	0.812	0.824	0.673	3.01E-04
cds.Camellia_sinensis.comp29417_c0_seq1_m.27919	PREDICTED: uncharacterized oxidoreductase At4g09670-like [Nicotiana tomentosiformis]	44.582	27.988	19.3	8	16	8	0.996	0.994	0.952	1.026	1.037	1.031	1.052	2.83E-02
cds.Camellia_sinensis.comp29418_c0_seq4_m.30677	PREDICTED: psbP domain-containing protein 3, chloroplastic [Juglans regia]	27.62	56.251	25.5	5	14	5	1.02	0.995	0.968	0.983	1.016	1.029	1.015	5.02E-01
cds.Camellia_sinensis.comp29420_c0_seq1_m.7741	PREDICTED: aminopeptidase M1 [Vitis vinifera]	94.411	200.73	28.1	25	44	24	0.901	0.913	0.924	1.114	1.077	1.099	1.202	1.17E-04
cds.Camellia_sinensis.comp29421_c0_seq1_m.26499	PREDICTED: ornithine carbamoyltransferase, chloroplastic-like [Nicotiana tabacum]	43.985	67.068	25.9	8	19	5	0.992	0.956	1.017	1.03	1.111	0.947	1.041	4.77E-01

cds.Camellia_sinensis.comp29421_c0_seq21_m.27772	PREDICTED: ornithine carbamoyltransferase, chloroplastic [Vitis vinifera]	44.517	13.757	19.4	6	18	3	0.973	1.034	1.015	0.948	1.043	0.995	0.988	7.25E-01
cds.Camellia_sinensis.comp29425_c0_seq1_m.27175	PREDICTED: RNA pseudouridine synthase 7 [Capsicum annuum]	12.561	8.0695	10.5	1	2	1								
cds.Camellia_sinensis.comp29426_c0_seq1_m.33534	PREDICTED: probable protein phosphatase 2C 26 [Juglans regia]	34.317	3.5489	7.3	2	3	2	0.977	1.03	1.093	1.04	0.988	0.885	0.94	3.33E-01
cds.Camellia_sinensis.comp29431_c0_seq1_m.26713	PREDICTED: ribosome maturation protein SBDS [Nelumbo nucifera]	40.957	11.771	5.6	2	2	2								
cds.Camellia_sinensis.comp29432_c0_seq1_m.15335	PREDICTED: eukaryotic translation initiation factor 3 subunit D-like [Vitis vinifera]	64.853	103.17	28.6	13	17	13	1.026	1.02	1.046	0.984	0.977	0.952	0.942	9.06E-03

cds.Camellia_sinensis.comp29433_c0_seq1_m.8748	PREDICTED: aldehyde dehydrogenase family 2 member B4, mitochondrial-like [Ziziphus jujuba]	58.352	289.89	37.4	21	66	11	1.036	1.001	1.027	0.968	0.964	0.969	0.947	3.47E-02
cds.Camellia_sinensis.comp29440_c1_seq1_m.13529	PREDICTED: threonine synthase, chloroplastic [Sesamum indicum]	59.995	113.27	23.2	12	23	8	0.993	0.97	0.996	1.029	1.007	1.038	1.039	3.62E-02
cds.Camellia_sinensis.comp29442_c0_seq1_m.19625	PREDICTED: 1-deoxy-D-xylulose 5-phosphate reductoisomerase, chloroplastic [Vitis vinifera]	51.292	98.423	26.9	11	24	11	1.008	1.022	1.02	0.982	0.976	0.976	0.962	1.24E-03
cds.Camellia_sinensis.comp29445_c0_seq1_m.25904	PREDICTED: E3 ubiquitin protein ligase RIE1 [Theobroma cacao]	39.628	8.9173	8.7	3	4	3	0.943	0.949	1.016	1.066	1.056	0.996	1.072	9.41E-02

cds.Camellia_sinensis.comp29449_c0_seq1_m.26166	PREDICTED: uracil phosphoribosyltransferase [Nelumbo nucifera]	18.45	4.6448	18.9	4	5	4	0.995	1.014	0.99	1.003	0.994	1.017	1.005	6.39E-01
cds.Camellia_sinensis.comp29453_c0_seq1_m.21413	PREDICTED: ankyrin repeat domain-containing protein 2B-like [Juglans regia]	38.004	5.4048	27.8	9	13	3	1.054	0.981	1.013	0.989	0.994	0.982	0.973	2.66E-01
cds.Camellia_sinensis.comp29453_c0_seq3_m.23364	PREDICTED: ankyrin repeat domain-containing protein 2B-like [Juglans regia]	38.658	59.736	30.4	9	14	3	1.119	1.054	0.992	0.913	0.927	0.943	0.879	2.40E-02
cds.Camellia_sinensis.comp29463_c0_seq5_m.41542	PREDICTED: 14 kDa zinc-binding protein isoform X1 [Vitis vinifera]	23.522	9.0624	15	3	3	3	0.973	0.994	1.057	1.037	0.984	0.969	0.989	7.48E-01

cds.Camellia_sinensis.comp29466_c0_seq1_m.19012	PREDICTED: lysosomal Pro-X carboxypeptidase [Jatropha curcas]	50.937	51.538	16.3	7	13	7	1.038	1.026	1.038	0.969	0.938	0.983	0.932	7.88E-03
cds.Camellia_sinensis.comp29469_c0_seq1_m.24379	PREDICTED: D-xylose-proton symporter-like 2 [Jatropha curcas]	53.585	2.0805	2	1	1	1								
cds.Camellia_sinensis.comp29469_c3_seq1_m.33264	integral membrane protein [Medicago truncatula]	31.598	8.1923	4.8	2	2	2	0.906	0.954	1.01	1.051	1.09	1.02	1.101	5.74E-02
cds.Camellia_sinensis.comp29470_c0_seq1_m.43811	PREDICTED: 17.3 kDa class I heat shock protein-like [Nelumbo nucifera]	18.121	7.5093	28.3	5	7	3	1.459	1.406	1.484	0.488	0.5	0.509	0.344	3.76E-07
cds.Camellia_sinensis.comp29472_c0_seq1_m.25608	PREDICTED: cell division protein FtsY homolog, chloroplastic [Daucus carota subsp. sativus]	39.021	46.233	28.3	9	16	9	1.019	1.053	1.025	0.966	0.979	0.956	0.937	5.92E-03

cds.Camellia_sinensis.comp29474_c0_seq1_m.21700	PREDICTED: dihydropyrimidine dehydrogenase (NADP(+)), chloroplastic [Ziziphus jujuba]	46.268	31.135	30.1	12	17	12	0.987	1.003	0.972	1.003	1.013	1.039	1.031	8.99E-02
cds.Camellia_sinensis.comp29477_c0_seq1_m.30956	thiol-disulfide oxidoreductase DCC [Medicago truncatula]	25.573	7.1022	8.9	2	3	2	0.944	0.935	0.963	1.095	1.068	1.042	1.128	2.00E-03
cds.Camellia_sinensis.comp29485_c0_seq1_m.3324	PREDICTED: phosphoglucan, water dikinase, chloroplastic isoform X1 [Vitis vinifera]	132.97	225.44	32.9	33	52	33	1.207	1.184	1.213	0.777	0.775	0.78	0.647	2.80E-07
cds.Camellia_sinensis.comp29491_c0_seq1_m.18947	chloroplast cystathionine beta lyase family protein [Populus trichocarpa]	50.257	25.197	11.4	5	7	5	1.006	1.019	1.026	1.004	0.963	0.975	0.964	5.55E-02

cds.Camellia_sinensis.comp29492_c0_seq1_m.29823	PREDICTED: serine racemase isoform X3 [Fragaria vesca subsp. vesca]	35.804	22.85	14.4	5	5	5	0.99	0.997	0.946	1.04	1.012	1.047	1.057	4.70E-02
cds.Camellia_sinensis.comp29499_c1_seq2_m.32075	survival motor neuron protein [Medicago truncatula]	26.914	2.7838	5.6	1	1	1								
cds.Camellia_sinensis.comp29504_c0_seq1_m.1006	PREDICTED: brefeldin A-inhibited guanine nucleotide-exchange protein 5 [Vitis vinifera]	198.79	86.639	14.9	22	25	22	1.021	1.038	1.022	0.962	0.964	0.971	0.94	5.22E-04
cds.Camellia_sinensis.comp29507_c0_seq1_m.40448	PREDICTED: frataxin, mitochondrial [Solanum tuberosum	22.721	4.2534	12.7	2	3	2	0.919	0.99	1.08	1.101	0.975	0.961	1.016	8.11E-01

cds.Camellia_sinensis.comp29513_c0_seq1_m.28159	PREDICTED: phosphoribosylglycinamide formyltransferase, chloroplastic [Vitis vinifera]	33.875	55.246	9.2	2	3	2	0.894	1.083	0.987	1.044	0.991	1.024	1.032	5.82E-01
cds.Camellia_sinensis.comp29516_c0_seq1_m.16444	PREDICTED: leucine aminopeptidase 1-like [Juglans regia]	60.078	323.31	53.4	23	63	19	0.986	0.997	0.964	1.026	1.029	1.014	1.041	2.02E-02
cds.Camellia_sinensis.comp29521_c0_seq1_m.22699	PREDICTED: COP9 signalosome complex subunit 4 [Ricinus	45.004	32.308	21.7	8	11	8	0.978	0.939	1.005	1.055	1.017	0.965	1.039	3.04E-01
cds.Camellia_sinensis.comp29523_c0_seq1_m.24971	PREDICTED: serine--glyoxylate aminotransferase [Capsicum annuum]	44.033	290.14	47.1	15	52	15	1.03	0.994	1.017	0.988	0.961	0.989	0.966	7.01E-02

cds.Camellia_sinensis.comp29527_c0_seq1_m.32348	PREDICTED: exosome complex component RRP41-like isoform X1 [Nelumbo nucifera]	27.497	2.1137	4.7	1	1	1	0.971	1.026	1.086	1.014	0.975	0.938	0.949	2.62E-01
cds.Camellia_sinensis.comp29532_c0_seq1_m.22729	PREDICTED: 4-hydroxyphenylpyruvate dioxygenase [Cucumis	50.965	11.36	10.2	4	4	4	0.979	1.005	1.014	0.99	0.992	1.022	1.002	8.98E-01
cds.Camellia_sinensis.comp29535_c0_seq1_m.38228	PREDICTED: probable plastid-lipid-associated protein 6, chloroplastic [Theobroma cacao]	29.197	159.72	40.7	10	42	10	0.922	0.91	0.911	1.071	1.119	1.083	1.193	2.18E-04
cds.Camellia_sinensis.comp29535_c1_seq2_m.43556	PREDICTED: nucleoside diphosphate kinase 3-like [Nicotiana tabacum]	25.9	25.72	16	5	11	5	0.96	0.977	0.999	1.012	1.013	1.019	1.037	3.65E-02

cds.Camellia_sinensis.comp29537_c0_seq1_m.2052	PREDICTED: DNA-directed RNA polymerase V subunit 1 [Vitis	102.79	6.1456	3.6	3	3	3	1.066	1.337	1.053	1.011	0.759	0.748	0.729	6.26E-02
cds.Camellia_sinensis.comp29537_c0_seq1_m.2053	PREDICTED: DNA-directed RNA polymerase V subunit 1 isoform X2 [Juglans	56.261	1.3349	1.6	1	1	1	1.054	1.145	1.254	0.826	0.837	0.854	0.729	3.56E-03
cds.Camellia_sinensis.comp29538_c0_seq1_m.25104	PREDICTED: cysteine synthase [Vitis vinifera]	34.269	187.64	50.3	13	55	10	0.962	0.943	0.922	1.091	1.064	1.054	1.135	1.38E-03
cds.Camellia_sinensis.comp29540_c0_seq1_m.38474	PREDICTED: adenylate kinase 4 [Jatropha curcas]	31.07	70.014	43.7	12	31	8	1.008	1	0.967	0.999	1.008	1.021	1.018	2.79E-01
cds.Camellia_sinensis.comp29543_c0_seq1_m.4810	PREDICTED: probable ubiquitin conjugation factor E4 [Nicotiana attenuata]	117.01	25.636	7.8	8	10	8	0.954	0.98	0.999	1.009	1.024	1.014	1.039	5.27E-02

cds.Camellia_sinensis.comp29545_c0_seq1_m.24783	PREDICTED: protein ORANGE, chloroplastic [Vitis vinifera]	28.601	2.7335	3.5	1	1	1	0.986	0.99	1.111	1.007	1.028	0.889	0.947	4.13E-01
cds.Camellia_sinensis.comp29546_c0_seq2_m.46414	PREDICTED: vesicle-associated membrane protein 727-like [Gossypium hirsutum]	19.514	2.0884	9.3	2	2	2	1.062	0.961	0.996	0.965	1.026	0.989	0.987	7.34E-01
cds.Camellia_sinensis.comp29548_c0_seq1_m.22053	PREDICTED: NPL4-like protein 2 [Ipomoea nil]	45.936	75.533	14.4	5	7	5	0.998	1.036	0.957	0.988	1	0.985	0.994	8.24E-01
cds.Camellia_sinensis.comp29554_c0_seq1_m.19460	PREDICTED: DEAD-box ATP-dependent RNA helicase 30 [Jatropha curcas]	16.77	9.1415	21.5	3	3	3	1.024	0.965	1.013	1.022	1.059	1.002	1.027	3.35E-01

cds.Camellia_sinensis.comp29554_c0_seq2_m.22346	PREDICTED: DEAD-box ATP-dependent RNA helicase 30 [Jatropha curcas]	46.651	49.842	34.9	13	16	10	0.946	1.018	1.005	1.016	1.031	1.014	1.031	2.53E-01
cds.Camellia_sinensis.comp29554_c1_seq1_m.24306	PREDICTED: protein ELC-like [Nicotiana tomentosiformis]	47.889	6.3077	4.2	2	3	2	0.881	0.966	0.995	1.129	0.989	1.076	1.124	9.39E-02
cds.Camellia_sinensis.comp29555_c0_seq1_m.4214	PREDICTED: receptor protein kinase TMK1 [Vitis vinifera]	104.07	-2	1.3	1	1	1	1.182	0.906	0.875	1.123	1.007	0.909	1.026	7.98E-01
cds.Camellia_sinensis.comp29562_c0_seq1_m.55670	ubiquitin carboxyl-terminal hydrolase 12-like, partial [Cajanus cajan]	15.392	10.026	8.4	1	1	1								

cds.Camellia_sinensis.comp29569_c1_seq1_m.23928	PREDICTED: aspartic proteinase nepenthesin-2 [Ziziphus jujuba]	50.443	1.3437	2.6	1	1	1								
cds.Camellia_sinensis.comp29572_c0_seq1_m.23092	PREDICTED: bifunctional D-cysteine desulfhydrase/1-aminocyclopropane-1-carboxylate deaminase, mitochondrial[Daucus carota subsp. sativus]	48.828	31.608	17.6	7	13	7	1.016	1.004	0.967	0.996	1.02	0.963	0.997	9.08E-01
cds.Camellia_sinensis.comp29574_c0_seq3_m.14046	PREDICTED: DNA-damage-repair/toleration protein DRT111, chloroplastic [Vitis vinifera]	21.365	4.4728	7.5	1	1	1	0.934	1.01	1.141	1.021	0.895	1.012	0.949	5.22E-01

cds.Camellia_sinensis.comp29577_c0_seq2_m.34497	period circadian protein [Arabidopsis thaliana]	26.274	1.5156	6.8	1	1	1								
cds.Camellia_sinensis.comp29581_c0_seq1_m.33141	PREDICTED: haloacid dehalogenase-like hydrolase domain-containing protein 3 [Vitis vinifera]	28.455	8.9899	15	4	7	4	0.983	1.021	0.995	1	1.012	1.022	1.012	4.14E-01
cds.Camellia_sinensis.comp29582_c0_seq21_m.33695	PREDICTED: dehydrogenase/reductase SDR family member 7 isoform X1 [Nelumbo nucifera]	35.635	13.533	19	5	6	5	0.958	1.025	0.983	1.06	1.011	1	1.035	2.60E-01
cds.Camellia_sinensis.comp29586_c0_seq1_m.33130	PREDICTED: isoeugenol synthase 1-like [Ziziphus jujuba]	35.935	3.5992	5.9	2	2	1	1.015	0.95	1.152	0.919	0.968	0.994	0.924	2.81E-01

cds.Camellia_sinensis.comp29593_c2_seq1_m.41307	small GTPase family RAB protein [Medicago truncatula]	19.166	3.1536	29.3	5	7	1								
cds.Camellia_sinensis.comp29593_c2_seq2_m.46337	PREDICTED: ras-related protein RHN1 isoform X2 [Glycine max]	23.583	40.741	33.3	6	9	2	0.916	0.955	0.989	1.047	1.054	1.069	1.108	1.09E-02
cds.Camellia_sinensis.comp29598_c1_seq2_m.44317	PREDICTED: low molecular weight phosphotyrosine protein phosphatase [Fragaria vesca subsp. vesca]	18.644	21.228	34.3	5	7	5	1.012	1.043	0.991	1.007	0.972	0.986	0.973	2.11E-01
cds.Camellia_sinensis.comp29607_c0_seq1_m.41648	PREDICTED: dihydroneopterin aldolase-like [Populus euphratica]	15.261	10.109	19.4	2	3	2	1.037	0.912	0.986	1.011	0.968	1.095	1.047	4.23E-01

cds.Camellia_sinensis.comp29613_c0_seq1_m.38462	PREDICTED: glyceraldehyde-3-phosphate dehydrogenase, cytosolic [Vitis vinifera]	35.407	79.99	47.7	15	52	6	1.027	0.965	0.965	1.017	1.033	0.997	1.03	2.61E-01
cds.Camellia_sinensis.comp29613_c0_seq2_m.38428	PREDICTED: glyceraldehyde-3-phosphate dehydrogenase, cytosolic [Vitis vinifera]	35.561	246.38	64.4	20	74	11	0.945	0.946	0.946	1.061	1.084	1.063	1.131	3.10E-03
cds.Camellia_sinensis.comp29615_c0_seq1_m.14327	PREDICTED: probable pectinesterase/pectinesterase inhibitor 51 [Vitis vinifera]	64.574	19.167	5.6	3	4	3	0.962	1.037	0.956	1.008	1.021	1.019	1.031	3.54E-01
cds.Camellia_sinensis.comp29617_c0_seq1_m.13279	PREDICTED: L-ascorbate oxidase homolog [Sesamum indicum]	62.602	1.864	2.9	2	2	1								

cds.Camellia_sinensis.comp29625_c0_seq1_m.18960	PREDICTED: transmembrane protein 87B-like [Ipomoea nil]	59.072	3.3679	5	3	3	3	0.99	0.974	1.023	1.033	0.976	1.016	1.013	6.01E-01
cds.Camellia_sinensis.comp29628_c0_seq1_m.32748	PREDICTED: probable ribose-5-phosphate isomerase 3, chloroplastic [Nicotiana tomentosiformis]	32.504	120.75	45.8	11	33	11	1.03	1.007	0.991	0.974	0.987	1.002	0.979	1.94E-01
cds.Camellia_sinensis.comp29631_c0_seq1_m.25884	PREDICTED: putative glucose-6-phosphate 1-epimerase isoform X2 [Ricinus communis]	34.535	43.535	12.8	3	3	3	0.971	0.968	0.995	1.029	0.967	1.085	1.05	2.36E-01
cds.Camellia_sinensis.comp29636_c0_seq1_m.42298	PREDICTED: probable glutathione S-transferase [Populus euphratica]	26.993	32.805	28.9	7	11	7	0.924	0.907	0.92	1.075	1.116	1.043	1.176	1.36E-03

cds.Camellia_sinensis.comp29638_c0_seq3_m.36028	PREDICTED: ATP synthase subunit delta', mitochondrial [Ipomoea nil]	23.797	6.1642	20.9	5	14	0								
cds.Camellia_sinensis.comp29638_c0_seq4_m.37459	PREDICTED: ATP synthase subunit delta', mitochondrial [Ipomoea nil]	23.785	22.784	20.9	5	13	0								
cds.Camellia_sinensis.comp29640_c0_seq1_m.16651	PREDICTED: protein TIC 55, chloroplastic [Theobroma cacao]	65.698	84.884	33.5	19	31	12	0.988	1.011	1	0.994	0.986	1.01	0.997	7.72E-01
cds.Camellia_sinensis.comp29640_c0_seq2_m.26004	PREDICTED: protein TIC 55, chloroplastic [Vitis vinifera]	57.381	7.5249	18.6	9	15	2	0.991	0.915	0.929	1.085	1.076	1.029	1.125	1.60E-02
cds.Camellia_sinensis.comp29649_c0_seq1_m.4774	binding protein [Arabidopsis lyrata subsp. lyrata]	31.408	5.1456	7	2	2	2	1.152	1.122	1.101	0.885	0.848	0.858	0.768	1.37E-04

cds.Camellia_sinensis.comp29649_c0_seq1_m.4775	TPR domain protein [Medicago truncatula]	19.568	2.0443	7.5	1	1	1	1.082	1.037	1.122	0.922	0.849	0.97	0.846	2.00E-02
cds.Camellia_sinensis.comp29652_c0_seq1_m.21483	PREDICTED: putative G3BP-like protein isoform X1 [Theobroma cacao]	52.446	40.274	16.9	7	8	7	1.033	1.05	1.095	0.94	0.948	0.921	0.884	3.12E-03
cds.Camellia_sinensis.comp29657_c0_seq1_m.37205	PREDICTED: eukaryotic translation initiation factor 5A [Eucalyptus grandis]	17.528	43.891	43.8	7	14	1	1.153	1.14	0.9	0.806	0.924	1.041	0.868	2.68E-01
cds.Camellia_sinensis.comp29657_c0_seq2_m.38060	PREDICTED: eukaryotic translation initiation factor 5A-2-like [Prunus mume]	17.532	21.353	31.9	5	8	3	1.002	1.085	1.032	0.955	0.96	0.965	0.923	7.75E-02

cds.Camellia_sinensis.comp29657_c0_seq3_m.44557	eukaryotic translation initiation factor 5A isoform V family protein [Populus trichocarpa]	14.964	14.546	19.7	3	5	2	1.078	1.029	1.031	0.933	0.924	0.992	0.908	2.28E-02
cds.Camellia_sinensis.comp29667_c0_seq1_m.29333	PREDICTED: DNA-damage-repair/toleration protein DRT100 [Solanum lycopersicum]	40.354	1.8888	6	2	2	2	0.946	0.879	0.851	1.074	1.205	1.084	1.257	9.22E-03
cds.Camellia_sinensis.comp29670_c0_seq1_m.28316	PREDICTED: uricase-2 isozyme 2 [Juglans regia]	39.992	33.176	18.6	8	10	8	0.958	0.949	0.991	1.023	1.063	1.041	1.079	1.15E-02
cds.Camellia_sinensis.comp29671_c0_seq2_m.15629	PREDICTED: probable lysine--tRNA ligase, cytoplasmic isoform X2 [Jatropha curcas]	55.368	70.612	21.9	9	11	9	0.999	1.029	0.947	1.006	1.024	1.013	1.023	4.04E-01

cds.Camellia_sinensis.comp29672_c0_seq1_m.33549		34.169	33.488	30.8	9	12	8	1.161	1.081	1.123	0.852	0.855	0.886	0.771	4.19E-04
cds.Camellia_sinensis.comp29672_c0_seq2_m.58600		12.028	1.9488	17	2	3	1	1.154	1.063	1.111	0.875	0.898	0.868	0.794	8.76E-04
cds.Camellia_sinensis.comp29679_c0_seq1_m.5887	PREDICTED: SCY1-like protein 2 [Vitis vinifera]	81.419	4.3922	2.7	2	2	2								
cds.Camellia_sinensis.comp29680_c0_seq1_m.30435	PREDICTED: malate dehydrogenase, cytoplasmic-like isoform X1 [Nicotiana tabacum]	37.838	14.158	16.7	6	8	5	1.057	1.035	0.992	1.017	0.954	0.968	0.953	1.48E-01
cds.Camellia_sinensis.comp29682_c0_seq1_m.10171	PREDICTED: transmembrane 9 superfamily member 11 [Vitis vinifera]	74.186	10.393	6.2	4	6	4	1.05	1.079	1.031	0.962	0.945	0.967	0.909	3.18E-03

cds.Camellia_sinensis.comp29683_c0_seq1_m.10827	PREDICTED: protein root UVB sensitive 1, chloroplastic [Vitis vinifera]	70.893	3.5356	4.9	2	2	2								
cds.Camellia_sinensis.comp29686_c0_seq1_m.12915	PREDICTED: sterol 3-beta-glucosyltransferase UGT80A2-like isoform X1 [Sesamum indicum]	67.241	3.5189	4.4	2	2	2	0.844	1.114	1.099	0.957	1.062	0.946	0.97	8.18E-01
cds.Camellia_sinensis.comp29687_c0_seq1_m.40450	PREDICTED: probable aldo-keto reductase 2 isoform X2 [Arachis duranensis]	22.094	240.61	67.5	11	30	10	0.951	0.913	0.922	1.092	1.091	1.095	1.177	5.70E-03
cds.Camellia_sinensis.comp29696_c0_seq2_m.22622	PREDICTED: histidinol-phosphate aminotransferase, chloroplastic-like [Malus domestica]	47.013	5.8874	11	3	4	3	1.055	1.063	0.844	1.196	1.057	0.805	1.032	8.71E-01

cds.Camellia_sinensis.comp29699_c0_seq1_m.14989	PREDICTED: bifunctional riboflavin biosynthesis protein RIBA 1, chloroplastic [Vitis vinifera]	64.091	1.3947	7.8	3	3	1								
cds.Camellia_sinensis.comp29699_c0_seq2_m.19920	PREDICTED: bifunctional riboflavin biosynthesis protein RIBA 1, chloroplastic-like isoform X1 [Populus euphratica]	61.332	31.521	13.8	6	6	4	0.955	0.935	0.947	1.07	1.026	1.083	1.121	2.90E-03
cds.Camellia_sinensis.comp29700_c0_seq2_m.24847	PREDICTED: histone deacetylase 14 isoform X5 [Juglans regia]	50.919	6.4069	6.7	3	5	3	1.062	1.013	0.959	0.986	0.959	1.019	0.977	5.43E-01

cds.Camellia_sinensis.comp29703_c0_seq1_m.36163	PREDICTED: thymidine kinase a isoform X2 [Vitis vinifera]	31.114	1.7459	5.8	1	1	1								
cds.Camellia_sinensis.comp29711_c0_seq1_m.12092	PREDICTED: uncharacterized aarF domain-containing protein kinase At1g79600, chloroplastic [Solanum tuberosum	75.342	62.14	18.7	11	13	11	0.931	0.942	1.005	1.072	1.073	1.026	1.102	2.55E-02
cds.Camellia_sinensis.comp29713_c0_seq1_m.26476	PREDICTED: probable cinnamyl alcohol dehydrogenase 1 [Ipomoea nil]	38.949	67.689	38.4	10	15	10	1.012	1.005	0.982	0.988	0.998	0.995	0.994	5.68E-01
cds.Camellia_sinensis.comp29714_c0_seq1_m.46746	3-oxoacyl-[acyl-carrier-protein] reductase [Populus trichocarpa]	23.207	1.9009	7.1	2	3	1	1.065	1.04	0.998	0.965	0.971	0.956	0.932	2.29E-02

cds.Camellia_sinensis.comp29714_c0_seq2_m.52145	PREDICTED: 3-oxoacyl-[acyl-carrier-protein] reductase 4 isoform X2 [Vitis vinifera]	17.023	1.4282	9.6	2	3	1	0.95	1.129	0.982	0.962	1.007	0.977	0.962	5.49E-01
cds.Camellia_sinensis.comp29715_c0_seq1_m.34103	PREDICTED: inositol-phosphate phosphatase-like [Ziziphus jujuba]	33.47	27.018	21.4	8	11	8	1.009	1.011	1.038	0.955	0.979	0.983	0.954	2.13E-02
cds.Camellia_sinensis.comp29717_c0_seq1_m.55314	PREDICTED: protein indeterminate-domain 2 isoform X1 [Camelina sativa]	15.382	2.1418	6	1	1	1	1.058	1.049	0.916	0.88	0.955	1.131	0.981	8.10E-01
cds.Camellia_sinensis.comp29718_c0_seq1_m.27494	PREDICTED: caffeic acid 3-O-methyltransferase isoform X3 [Vitis vinifera]	42.462	39.381	28.8	9	14	9	1.008	0.994	1.009	1.007	1.021	0.984	1	9.85E-01

cds.Camellia_sinensis.comp29722_c0_seq1_m.57308	PREDICTED: farnesyl pyrophosphate synthase 1 [Arachis ipaensis]	13.421	10.352	19	2	5	1	1.075	1.062	0.836	1.064	1.005	0.963	1.02	7.85E-01
cds.Camellia_sinensis.comp29727_c0_seq1_m.24095	SNF1-related kinase regulatory subunit gamma 1 [Medicago truncatula]	47.636	46.988	19.6	7	14	7	0.934	0.952	0.996	1.075	1.09	1.023	1.106	2.04E-02
cds.Camellia_sinensis.comp29728_c0_seq1_m.9789	PREDICTED: oligopeptide transporter 3 [Nicotiana tabacum]	83.343	45.247	1.6	1	1	1	1.098	1.073	1.073	0.906	0.899	0.93	0.843	1.82E-04
cds.Camellia_sinensis.comp29739_c0_seq2_m.39439	ATP synthase G subunit family protein [Medicago truncatula]	14.202	2.2604	27	4	5	2	1.064	0.992	0.972	1.004	0.924	1.042	0.981	6.81E-01

cds.Camellia_sinensis.comp29739_c0_seq3_m.40040	mitochondrial ATP synthase g subunit family protein [Arabidopsis lyrata subsp. lyrata]	14.126	22.495	41.8	6	10	3	0.952	1.02	1.007	0.996	1.04	0.992	1.016	5.60E-01
cds.Camellia_sinensis.comp29741_c0_seq1_m.17121	PREDICTED: carbamoyl-phosphate synthase small chain, chloroplastic isoform X2 [Daucus carota subsp. sativus]	46.573	35.812	12.4	4	7	4	1.015	1.043	0.997	0.98	0.994	1.014	0.978	2.50E-01
cds.Camellia_sinensis.comp29742_c0_seq1_m.33840	PREDICTED: kynurenine formamidase isoform X1 [Solanum tuberosum	32.454	94.718	32.2	10	29	10	0.897	0.92	0.935	1.104	1.073	1.078	1.183	3.59E-04
cds.Camellia_sinensis.comp29756_c0_seq1_m.19956	calreticulin family protein [Populus trichocarpa]	47.598	62.757	41.5	15	26	15	0.999	1.028	0.991	0.976	0.981	1.008	0.982	3.03E-01

cds.Camellia_sinensis.comp29771_c0_seq1_m.29964	PREDICTED: probable 6-phosphogluconolactonase 4, chloroplastic [Nicotiana attenuata]	40.03	28.471	23.1	9	17	7	0.985	1	0.973	1.086	1.027	1.038	1.065	2.89E-02
cds.Camellia_sinensis.comp29771_c0_seq2_m.30189	PREDICTED: probable 6-phosphogluconolactonase 4, chloroplastic [Nicotiana attenuata]	28.055	7.5369	19.8	5	11	3	0.921	0.908	0.825	1.089	1.143	1.11	1.259	3.44E-03
cds.Camellia_sinensis.comp29773_c0_seq1_m.1135	PREDICTED: protein NETWORKED 1D [Vitis vinifera]	141.52	3.1113	0.8	1	1	1								
cds.Camellia_sinensis.comp29774_c0_seq2_m.36934	ribulose-phosphate 3-epimerase, chloroplastic [Solanum lycopersicum]	30.733	119.56	26.1	5	20	5	1.052	1.035	1.014	0.955	0.97	0.943	0.925	4.34E-03

cds.Camellia_sinensis.comp29780_c0_seq1_m.42303	Thioredoxin family protein [Arabidopsis thaliana]	31.601	21.099	17.1	3	5	3	1.043	0.965	0.937	1.091	0.926	1	1.024	7.09E-01
cds.Camellia_sinensis.comp29787_c0_seq2_m.12219	PREDICTED: probable polyol transporter 6 [Eucalyptus grandis]	54.799	12.065	5.9	3	6	3	0.921	0.922	0.893	1.137	1.048	1.13	1.212	2.46E-03
cds.Camellia_sinensis.comp29789_c0_seq1_m.6782	PREDICTED: scarecrow-like protein 14 [Nicotiana tomentosiformis]	86.597	24.436	9.7	7	9	7	1.003	1.058	1.084	1.066	0.9	0.918	0.917	2.01E-01
cds.Camellia_sinensis.comp29794_c0_seq1_m.35453	PREDICTED: peroxiredoxin-2F, mitochondrial [Daucus carota subsp. sativus]	14.118	20.617	31.5	3	9	3	1.005	0.975	0.97	0.979	1.038	1.009	1.026	2.79E-01

cds.Camellia_sinensis.comp29804_c0_seq1_m.27521	PREDICTED: dnaJ homolog subfamily C member 17 [Nicotiana tomentosiformis]	32.649	6.2867	15.1	5	5	5	1.093	0.981	1.043	0.925	0.988	0.947	0.918	8.08E-02
cds.Camellia_sinensis.comp29810_c0_seq1_m.32455	PREDICTED: dnaJ homolog subfamily B member 13-like [Nelumbo nucifera]	35.917	1.4394	3	1	1	1								
cds.Camellia_sinensis.comp29812_c0_seq1_m.18555	PREDICTED: 4-coumarate--CoA ligase 2-like [Jatropha curcas]	60.204	4.0311	7.8	5	5	2	1.142	1.254	1.239	0.822	0.723	0.767	0.636	6.62E-04
cds.Camellia_sinensis.comp29815_c0_seq1_m.38712	PREDICTED: fasciclin-like arabinogalactan protein 7 [Vitis vinifera]	27.979	1.3848	3.4	1	1	1								

cds.Camellia_sinensis.comp29820_c0_seq1_m.12786	PREDICTED: pentatricopeptide repeat-containing protein At1g10270 [Vitis vinifera]	49.45	1.1883	3.9	2	2	2								
cds.Camellia_sinensis.comp29828_c0_seq1_m.14841	PREDICTED: ATP-dependent DNA helicase 2 subunit KU70 [Populus euphratica]	71.173	2.9452	3.2	2	2	2								
cds.Camellia_sinensis.comp29836_c0_seq1_m.28714	PREDICTED: glyoxylate/succinic semialdehyde reductase 2, chloroplastic isoform X2 [Vitis vinifera]	21.748	39.622	19	3	4	3	1.066	1.042	1.054	0.973	0.933	1.05	0.935	1.23E-01
cds.Camellia_sinensis.comp29844_c0_seq3_m.47883	PREDICTED: ras-related protein RABD1 [Jatropha curcas]	18.559	12.196	38.6	6	7	5	0.98	0.985	0.97	1.028	1.027	1.08	1.068	1.89E-02

cds.Camellia_sinensis.comp29847_c0_seq1_m.38197	PREDICTED: cyclic phosphodiesterase-like [Ziziphus jujuba]	30.663	14.943	13	3	4	3	0.948	0.971	0.992	1.065	1.059	0.991	1.07	6.43E-02
cds.Camellia_sinensis.comp29851_c0_seq1_m.42357	PREDICTED: protochlorophyllide reductase, chloroplastic-like [Malus domestica]	25.76	17.318	25.5	5	10	4	0.993	0.95	0.972	1.051	1.068	1.021	1.077	1.55E-02
cds.Camellia_sinensis.comp29854_c0_seq1_m.26534	PREDICTED: mitogen-activated protein kinase 3 [Eucalyptus grandis]	42.755	5.427	5.1	2	2	2	0.948	0.961	0.989	1.044	1.078	1.006	1.079	3.19E-02
cds.Camellia_sinensis.comp29855_c0_seq1_m.35040	PREDICTED: UMP-CMP kinase isoform X1 [Vitis vinifera]	29.208	2.4452	10.2	3	4	2	1.036	0.927	0.978	1.005	1.029	1.034	1.043	2.64E-01
cds.Camellia_sinensis.comp29859_c0_seq1_m.23082	PREDICTED: COBW domain-containing protein 1-like [Nicotiana tabacum]	23.858	4.4182	6.4	1	1	1								

cds.Camellia_sinensis.comp29859_c0_seq1_m.23083	PREDICTED: uncharacterized GTP-binding protein YjiA [Ricinus communis]	20.676	31.415	32.8	5	6	5	1.118	1.151	1.063	0.861	0.882	0.87	0.784	5.61E-04
cds.Camellia_sinensis.comp29873_c0_seq13_m.9189	PREDICTED: dolichyl-diphosphooligosaccharide--protein glycosyltransferase subunit STT3B [Vitis vinifera]	83.31	18.558	12	10	14	10	0.954	0.966	0.989	0.991	1.009	1.063	1.053	9.50E-02
cds.Camellia_sinensis.comp29874_c0_seq1_m.17960	AP-2 complex subunit mu [Morus notabilis]	49.362	12.564	14.2	6	8	6	0.979	1.007	0.991	1.008	1.007	1.012	1.017	1.14E-01
cds.Camellia_sinensis.comp29888_c0_seq2_m.28666	PREDICTED: anthocyanidin reductase ((2S)-flavan-3-ol-forming)-like isoform X2 [Vitis vinifera]	38.354	62.24	25.1	10	15	10	0.984	0.97	0.967	0.999	1.048	1.032	1.054	2.54E-02

cds.Camellia_sinensis.comp29891_c0_seq1_m.28084	PREDICTED: lanC-like protein GCR2 [Vitis vinifera]	46.079	19.22	14.3	6	8	6	0.987	1.014	0.949	0.979	1.014	1.03	1.025	3.71E-01
cds.Camellia_sinensis.comp29898_c0_seq1_m.36003	PREDICTED: tropinone reductase homolog [Sesamum indicum]	28.72	29.677	21.3	6	14	4	0.958	1.031	1.041	0.998	1.024	0.962	0.985	6.64E-01
cds.Camellia_sinensis.comp29901_c0_seq1_m.36720	PREDICTED: 101 kDa malaria antigen [Phoenix dactylifera]	19.319	34.271	31.2	6	13	6	1.015	0.997	0.979	0.99	0.99	1.008	0.999	9.42E-01
cds.Camellia_sinensis.comp29903_c0_seq1_m.17024	PREDICTED: 6-phosphogluconate dehydrogenase, decarboxylating 1, chloroplastic [Vitis vinifera]	54.431	26.46	29.2	12	20	4	0.983	0.958	0.96	1.042	1.006	1.056	1.07	1.55E-02

cds.Camellia_sinensis.comp29903_c0_seq2_m.17109	PREDICTED: 6-phosphogluconate dehydrogenase, decarboxylating 1, chloroplastic [Vitis vinifera]	54.139	120.3	37.3	16	25	8	0.949	0.956	0.994	1.103	1.071	1.015	1.1	2.83E-02
cds.Camellia_sinensis.comp29905_c0_seq1_m.16721	PREDICTED: probable zinc metallopeptidase EGY3, chloroplastic isoform X1 [Juglans regia]	67.992	28.865	11.9	8	15	8	1.007	0.977	1.025	0.991	0.987	1.027	0.999	9.49E-01
cds.Camellia_sinensis.comp29910_c0_seq1_m.4399	PREDICTED: probable prolyl 4-hydroxylase 10 [Gossypium raimondii]	32.219	2.5556	9.3	3	3	3								
cds.Camellia_sinensis.comp29915_c0_seq3_m.16355	PREDICTED: exportin-7 isoform X1 [Nelumbo nucifera]	62.303	1.5927	3.7	2	2	2	1.019	1.074	0.928	0.971	0.995	1.015	0.987	8.06E-01

cds.Camellia_sinensis.comp29917_c0_seq1_m.18255	PREDICTED: 4-coumarate--CoA ligase 1 [Nicotiana attenuata]	58.683	20.605	9.8	5	5	3	1.067	1.257	1.129	0.902	0.798	0.824	0.731	6.66E-03
cds.Camellia_sinensis.comp29920_c0_seq1_m.35534	PREDICTED: uric acid degradation bifunctional protein TTL isoform X1 [Vitis vinifera]	36.406	11.004	17.1	4	5	4	1.036	0.956	1.024	1.019	1.078	0.997	1.026	4.96E-01
cds.Camellia_sinensis.comp29933_c0_seq2_m.34592	haloacid dehalogenase-like hydrolase family protein [Populus trichocarpa]	27.33	34.643	21.8	5	7	5	1.12	1.088	1.097	0.883	0.875	0.859	0.792	3.95E-05
cds.Camellia_sinensis.comp29934_c0_seq1_m.38024	alpha/beta hydrolase family protein [Medicago truncatula]	21.958	3.1567	6.8	1	1	1	1.207	0.954	1.052	0.922	0.916	0.922	0.859	1.64E-01

cds.Camellia_sinensis.comp29934_c1_seq1_m.31287	PREDICTED: protein CONTINUOUS VASCULAR RING 1 [Vitis vinifera]	33.118	1.744	3.3	1	1	1								
cds.Camellia_sinensis.comp29935_c0_seq1_m.33952	PREDICTED: PRA1 family protein A1-like [Populus euphratica]	30.2	2.6212	6.5	1	1	1								
cds.Camellia_sinensis.comp29937_c0_seq7_m.13219	PREDICTED: prolycopene isomerase, chloroplastic [Prunus mume]	33.43	3.4696	6.3	2	3	2	1.016	1.012	0.962	0.941	1.039	1.033	1.008	8.59E-01
cds.Camellia_sinensis.comp29939_c0_seq2_m.19375	PREDICTED: UBP1-associated protein 2C [Nelumbo nucifera]	40.514	19.16	12.9	5	9	4	0.941	0.99	0.972	1.025	1.044	0.989	1.053	7.51E-02

cds.Camellia_sinensis.comp29941_c0_seq2_m.29977	PREDICTED: transcription initiation factor IIB-2 [Daucus carota subsp. sativus]	34.279	27.15	9	3	3	3	0.834	1.003	0.986	1.099	1.044	1.093	1.146	8.41E-02
cds.Camellia_sinensis.comp29944_c0_seq2_m.24140	Protease HtpX-2-like protein [Morus notabilis]	27.982	13.874	15	5	5	5	0.934	0.939	1.011	1.065	1.046	1.017	1.085	4.74E-02
cds.Camellia_sinensis.comp29947_c0_seq2_m.16040	PREDICTED: glutamate decarboxylase 1 [Vitis vinifera]	55.542	50.339	21.5	10	20	9	0.889	0.956	0.926	1.116	1.066	1.075	1.175	3.08E-03
cds.Camellia_sinensis.comp29947_c0_seq3_m.35602	transmembrane protein, putative [Medicago truncatula]	29.512	44.383	14.6	4	4	4	1.085	1.007	1.125	0.949	0.915	0.894	0.857	1.43E-02
cds.Camellia_sinensis.comp29949_c0_seq1_m.28537	2-oxoacid dehydrogenases acyltransferase family protein [Arabidopsis thaliana]	32.796	26.088	25.2	6	8	5	1.066	0.939	1	1.005	0.998	0.989	0.996	9.42E-01

cds.Camellia_sinensis.comp29954_c0_seq2_m.42248	PREDICTED: 60S ribosomal protein L23-like [Nicotiana tabacum]	15.061	23.705	39.3	5	9	5	1.179	1.179	1.152	0.831	0.825	0.813	0.703	1.99E-06
cds.Camellia_sinensis.comp29955_c0_seq1_m.35485	PREDICTED: MATH domain and coiled-coil domain-containing protein At2g42460 [Sesamum	36.878	6.313	13.2	4	6	4	0.93	0.967	0.917	1.142	0.981	1.056	1.13	6.15E-02
cds.Camellia_sinensis.comp29957_c0_seq1_m.10883	sodium/calcium exchanger family protein / calcium-binding EF hand family protein [Arabidopsis thaliana]	56.8	1.5248	1.8	1	1	1	1.134	1.03	1.015	0.974	0.997	0.836	0.883	1.25E-01

cds.Camellia_sinensis.comp29958_c0_seq2_m.31744	PREDICTED: cell division protein FtsZ homolog 2-1, chloroplastic [Vitis vinifera]	34.357	85.035	26.5	6	10	5	1.061	1.121	1.04	0.938	0.905	0.932	0.861	3.98E-03
cds.Camellia_sinensis.comp29970_c1_seq5_m.23803	PREDICTED: heterogeneous nuclear ribonucleoprotein 1 [Vitis vinifera]	42.742	48.218	11.2	4	7	4	0.922	1.008	0.931	1.032	1.075	1.036	1.099	3.83E-02
cds.Camellia_sinensis.comp29972_c0_seq2_m.28176	PREDICTED: tobamovirus multiplication protein 1 [Prunus mume]	20.692	1.7538	3.9	1	1	1	0.972	0.929	0.979	1.021	1.066	1.056	1.091	1.41E-02
cds.Camellia_sinensis.comp29976_c1_seq26_m.21205	PREDICTED: protein EARLY-RESPONSIVE TO DEHYDRATION 7, chloroplastic [Vitis vinifera]	48.502	73.778	30.1	12	22	12	0.967	0.961	0.983	1.029	1.041	1.049	1.071	1.40E-03

cds.Camellia_sinensis.comp29978_c0_seq1_m.26173	PREDICTED: annexin D3 [Ricinus	39.548	8.9225	10.7	4	4	4	0.968	0.907	1.057	1.072	1.011	1.008	1.054	3.26E-01
cds.Camellia_sinensis.comp29983_c0_seq2_m.38552	PREDICTED: probable prefoldin subunit 3 isoform X2 [Nelumbo nucifera]	19.069	19.511	7.1	1	1	1	0.795	1.029	1.073	1.145	1.015	0.989	1.087	4.37E-01
cds.Camellia_sinensis.comp29990_c0_seq2_m.8649	ABC transporter family protein [Populus trichocarpa]	81.758	36.259	9.4	6	10	6	1.064	1.048	1.007	0.963	0.951	0.927	0.911	9.40E-03
cds.Camellia_sinensis.comp30008_c0_seq1_m.29105	PREDICTED: translocase of chloroplast 34, chloroplastic isoform X2 [Vitis vinifera]	33.364	65.989	31	9	15	9	0.978	0.975	1.003	1.038	1.019	1	1.034	7.48E-02
cds.Camellia_sinensis.comp30008_c0_seq2_m.32150	PREDICTED: ras-related protein Rab7 [Gossypium arboreum]	23.072	13.996	53.9	9	22	4	0.983	0.97	0.984	1.039	1.029	0.974	1.036	1.68E-01

cds.Camellia_sinensis.comp30011_c0_seq2_m.23817	PREDICTED: DUF21 domain-containing protein At4g14240 [Vitis vinifera]	54.604	42.688	17.9	6	6	5	0.968	1.055	1.067	1.053	0.903	0.964	0.945	3.47E-01
cds.Camellia_sinensis.comp30012_c1_seq3_m.27799	PREDICTED: nuclear pore complex protein NUP88 [Vitis vinifera]	38.651	10.564	14.3	5	6	5	1.012	0.987	1.03	0.985	0.968	0.998	0.974	1.62E-01
cds.Camellia_sinensis.comp30013_c0_seq1_m.29161	domon-like ligand-binding domain-containing protein precursor [Solanum lycopersicum]	39.598	2.7553	9.1	3	3	3	0.913	1.055	1.121	0.945	1.013	0.964	0.946	4.61E-01
cds.Camellia_sinensis.comp30018_c0_seq1_m.25032	PREDICTED: protochlorophyllide reductase, chloroplastic [Citrus sinensis]	43.212	39.92	25.8	10	15	4	0.989	0.928	0.958	1.043	1.048	1.064	1.097	8.50E-03

cds.Camellia_sinensis.comp30018_c0_seq2_m.25602	PREDICTED: protochlorophyllide reductase, chloroplastic [Citrus sinensis]	43.243	3.1027	18.3	7	8	1	0.799	0.901	0.872	1.163	1.188	1.141	1.358	1.24E-03
cds.Camellia_sinensis.comp30019_c0_seq1_m.15873	PREDICTED: blue-light photoreceptor PHR2 [Nicotiana tomentosiformis]	49.656	2.6415	2.6	1	1	1	1.473	1.266	1.338	0.627	0.559	0.615	0.442	1.38E-04
cds.Camellia_sinensis.comp30020_c0_seq10_m.23866	PREDICTED: 40S ribosomal protein SA-like [Ipomoea nil]	32.087	106.39	41.2	11	27	11	0.974	0.974	0.964	1.063	1.028	1.046	1.077	1.92E-03
cds.Camellia_sinensis.comp30027_c0_seq6_m.31289	peptidyl-prolyl cis-trans isomerase FKBP19, chloroplastic isoform X4 [Cajanus cajan]	17.666	11.529	35.7	5	6	5	1.034	1.052	1.032	0.991	0.928	0.992	0.934	3.92E-02

cds.Camellia_sinensis.comp30027_c0_seq8_m.47951	PREDICTED: peptidyl-prolyl cis-trans isomerase FKBP19, chloroplastic [Jatropha curcas]	21.909	1.3105	7.1	1	1	1								
cds.Camellia_sinensis.comp30040_c0_seq1_m.22474	PREDICTED: pyruvate dehydrogenase E1 component subunit alpha-3, chloroplastic [Vitis vinifera]	48.213	10.307	8.5	4	4	2	1.029	1.012	1.017	0.97	0.981	0.992	0.962	9.24E-03
cds.Camellia_sinensis.comp30040_c0_seq2_m.23034	PREDICTED: pyruvate dehydrogenase E1 component subunit alpha-3, chloroplastic [Vitis vinifera]	48.065	9.6164	10.5	4	6	2	0.976	1.007	0.838	1.032	1.025	1.051	1.102	2.23E-01

cds.Camellia_sinensis.comp30046_c1_seq7_m.26133	PREDICTED: bifunctional riboflavin kinase/FMN phosphatase [Daucus carota subsp. sativus]	43.406	9.2158	10.4	4	4	4	1.066	0.99	0.996	0.955	0.981	0.973	0.953	1.30E-01
cds.Camellia_sinensis.comp30049_c0_seq1_m.26651	PREDICTED: methyl-CpG-binding domain-containing protein 13 isoform X4 [Vitis vinifera]	45.456	14.276	10.6	3	3	3	1.13	1.068	1.083	0.838	0.907	0.943	0.819	6.60E-03
cds.Camellia_sinensis.comp30052_c0_seq1_m.27677	PREDICTED: histidine--tRNA ligase, cytoplasmic [Jatropha curcas]	51.362	10.188	16.6	7	8	7	0.977	1.018	1.022	1.009	0.987	1.037	1.005	8.07E-01

cds.Camellia_sinensis.comp30053_c0_seq1_m.25085	PREDICTED: 15-cis-phytoene desaturase, chloroplastic/chromoplastic-like [Populus euphratica]	52.942	3.8256	5.4	3	3	3	0.949	0.961	1.062	1.009	0.982	1.055	1.025	5.71E-01
cds.Camellia_sinensis.comp30057_c0_seq1_m.29301	2-dehydro-3-deoxyphosphoheptonate aldolase family protein [Populus trichocarpa]	43.241	36.368	32.1	13	31	7	1.294	1.258	1.324	0.676	0.671	0.673	0.521	5.23E-04
cds.Camellia_sinensis.comp30057_c1_seq2_m.50785	PREDICTED: phospho-2-dehydro-3-deoxyheptonate aldolase 1, chloroplastic-like [Malus domestica]	19.615	40.349	26.9	4	8	4	1.335	1.244	1.382	0.642	0.668	0.639	0.492	3.57E-05

cds.Camellia_sinensis.comp30063_c0_seq1_m.13090	PREDICTED: glutamyl-tRNA reductase 1, chloroplastic-like [Nicotiana tomentosiformis]	59.838	21.126	12.1	6	6	6	0.994	1.005	1.066	0.966	0.983	0.998	0.962	1.76E-01
cds.Camellia_sinensis.comp30068_c0_seq11_m.36320	PREDICTED: 1,4-dihydroxy-2-naphthoyl-CoA synthase, peroxisomal-like isoform X1 [Nicotiana tabacum]	32.792	6.0648	9.9	3	3	3	0.979	0.927	1.011	1.047	1.027	1.029	1.064	7.47E-02
cds.Camellia_sinensis.comp30070_c0_seq1_m.25428	PREDICTED: acetylornithine deacetylase [Solanum	48.944	114.87	20.3	7	14	7	0.963	0.992	1.006	1.03	1.006	1.008	1.028	1.37E-01
cds.Camellia_sinensis.comp30073_c0_seq2_m.10486	PREDICTED: DEAD-box ATP-dependent RNA helicase 8 [Nelumbo nucifera]	56.587	19.491	17.5	7	10	2	0.866	1.006	0.956	1.06	1.1	1.05	1.135	4.99E-02

cds.Camellia_sinensis.comp30074_c0_seq1_m.11760	PREDICTED: beta-adaptin-like protein A [Nicotiana tabacum]	63.034	3.4447	3	1	1	1	0.911	1.035	0.999	0.94	1.002	1.129	1.043	5.72E-01
cds.Camellia_sinensis.comp30075_c0_seq1_m.24809	PREDICTED: malate dehydrogenase, chloroplastic [Solanum tuberosum	43.67	153.91	36.9	14	40	14	1.01	1.001	1.018	1.001	0.985	0.971	0.976	7.43E-02
cds.Camellia_sinensis.comp30077_c0_seq1_m.25826	PREDICTED: rhomboid-like protein 19 [Juglans regia]	34.729	6.1518	5.4	1	1	1	0.978	0.937	1.088	0.976	1.041	0.992	1.002	9.44E-01
cds.Camellia_sinensis.comp30080_c0_seq1_m.19030	PREDICTED: glutaredoxin domain-containing cysteine-rich protein 1 [Jatropha curcas]	37.479	49.119	27	8	9	8	1.016	1.028	1.015	0.949	1.006	0.979	0.959	7.26E-02
cds.Camellia_sinensis.comp30085_c0_seq1_m.16810	PREDICTED: omega-6 fatty acid desaturase, chloroplastic [Vitis vinifera]	33.054	3.818	3.6	1	1	1	0.967	0.99	1.088	0.952	0.985	1.026	0.973	5.63E-01

cds.Camellia_sinensis.comp30087_c0_seq1_m.47948	PREDICTED: 2S albumin-like [Ricinus communis]	17.357	37.043	33.6	6	10	6	1.119	1.136	1.161	0.826	0.847	0.86	0.742	4.35E-05
cds.Camellia_sinensis.comp30091_c0_seq1_m.32600	PREDICTED: protein PPLZ12 [Sesamum indicum]	32.318	39.018	36.6	10	16	10	0.97	1.028	1.027	0.969	0.991	0.985	0.974	2.63E-01
cds.Camellia_sinensis.comp30093_c0_seq1_m.28754	PREDICTED: polygalacturonase inhibitor-like [Sesamum indicum]	36.854	19.237	22	7	15	7	0.929	0.955	0.983	1.074	1.062	1.051	1.112	3.72E-03
cds.Camellia_sinensis.comp30101_c0_seq1_m.30505	PREDICTED: 60S ribosomal protein L19-1-like [Cucumis melo]	24.756	34.606	20.8	4	8	2	0.99	1.09	1.037	0.894	0.914	0.959	0.888	2.66E-02
cds.Camellia_sinensis.comp30105_c0_seq1_m.26906	PREDICTED: indole-3-glycerol phosphate synthase, chloroplastic [Vitis vinifera]	47.555	185.03	47.6	20	51	20	0.989	0.985	0.978	1.015	1.013	1.017	1.032	8.59E-04

cds.Camellia_sinensis.comp30119_c0_seq2_m.13290	PREDICTED: probable inactive receptor kinase At1g48480 [Jatropha curcas]	68.142	13.523	7	5	6	5	1.01	0.975	0.94	0.979	1.041	1.069	1.056	1.78E-01
cds.Camellia_sinensis.comp30121_c0_seq1_m.23941	PREDICTED: homoserine kinase-like [Nicotiana tabacum]	39.752	17.585	5.5	2	4	2	1.002	1.032	0.984	1.03	1.008	1.011	1.01	5.39E-01
cds.Camellia_sinensis.comp30123_c0_seq1_m.21466	PREDICTED: serine--tRNA ligase, mitochondrial [Jatropha curcas]	58.997	79.008	21.6	10	12	10	0.967	0.971	0.979	1.049	1.047	1.017	1.067	3.66E-03
cds.Camellia_sinensis.comp30125_c1_seq1_m.52105	PREDICTED: mitogen-activated protein kinase homolog NTF6 [Vitis vinifera]	19.24	1.2801	6	1	1	1	0.976	0.982	0.947	1.078	1.04	1	1.073	4.48E-02

cds.Camellia_sinensis.comp30126_c0_seq1_m.7663	PREDICTED: exocyst complex component SEC10 [Ricinus communis]	96.479	63.03	10.3	9	10	9	1.006	0.968	1.025	1.009	1	1.057	1.022	4.11E-01
cds.Camellia_sinensis.comp30127_c0_seq1_m.36678	immunophilin family protein [Populus trichocarpa]	23.23	5.6207	5.3	1	1	1	1.103	1.137	1.175	0.783	0.891	0.874	0.746	2.64E-03
cds.Camellia_sinensis.comp30127_c1_seq1_m.22508	PREDICTED: angiomotin-like [Jatropha curcas]	39.859	2.1643	2.2	1	1	1	0.958	0.88	0.931	1.039	1.126	1.098	1.178	8.86E-03
cds.Camellia_sinensis.comp30143_c0_seq1_m.34785	PREDICTED: secoisolariciresinol dehydrogenase [Vitis vinifera]	35.948	3.5757	5.7	2	3	1								
cds.Camellia_sinensis.comp30143_c0_seq2_m.34793	PREDICTED: secoisolariciresinol dehydrogenase-like [Nicotiana tabacum]	36.795	15.607	13	4	4	3	0.985	0.824	0.93	1.013	1.188	1.089	1.201	5.82E-02

cds.Camellia_sinensis.comp30146_c0_seq1_m.21634	PREDICTED: nuclear pore complex protein NUP50A [Theobroma cacao]	44.255	6.2386	12.8	4	4	4	1.311	1.197	1.167	0.75	0.718	0.78	0.612	3.23E-04
cds.Camellia_sinensis.comp30150_c0_seq1_m.14958	PREDICTED: putative leucine-rich repeat receptor-like serine/threonine-protein kinase At2g14440 [Ipomoea	38.064	1.2569	2	1	1	1	1.035	1.045	1.031	0.994	1.005	0.891	0.929	1.91E-01
cds.Camellia_sinensis.comp30158_c0_seq2_m.35978	PREDICTED: vesicle-associated protein 1-3 isoform X2 [Nicotiana sylvestris]	26.985	4.2751	3.7	1	1	1	0.993	1.012	1.036	0.88	1.059	1.021	0.973	6.32E-01
cds.Camellia_sinensis.comp30162_c0_seq1_m.16949	PREDICTED: cytochrome P450 89A2-like [Nicotiana attenuata]	63.252	96.112	21	11	17	8	1.003	0.985	0.951	1.065	0.996	0.988	1.037	2.70E-01

cds.Camellia_sinensis.comp30163_c0_seq4_m.39095	glycosyltransferase family protein [Populus trichocarpa]	31.332	-2	3.8	1	1	1	0.975	0.976	1.074	1.047	0.906	1.034	0.987	8.20E-01
cds.Camellia_sinensis.comp30177_c0_seq3_m.34348	PREDICTED: ATP-dependent Clp protease ATP-binding subunit CLPT1, chloroplastic-like [Nicotiana tomentosiformis]	25.666	16.62	12.9	3	8	2	0.932	1.023	0.944	1.128	0.979	0.956	1.057	4.21E-01
cds.Camellia_sinensis.comp30186_c1_seq1_m.34935	PREDICTED: ER membrane protein complex subunit 3 [Vitis vinifera]	29.572	13.38	15.1	3	3	3	0.956	0.88	1.062	1.074	0.997	1.025	1.068	3.07E-01
cds.Camellia_sinensis.comp30190_c0_seq1_m.10899	PREDICTED: 30S ribosomal protein 2, chloroplastic [Sesamum indicum]	28.529	50.981	23.6	7	16	7	1.031	1.023	1.031	0.955	0.993	0.947	0.938	1.27E-02

cds.Camellia_sinensis.comp30197_c0_seq1_m.31887	beta-glucosidase 44-like [Cajanus cajan]	45.854	18.358	17.7	8	9	8	1.009	1.024	0.959	0.965	0.98	1.018	0.99	7.25E-01
cds.Camellia_sinensis.comp30200_c1_seq1_m.43417	PREDICTED: 60S ribosomal protein L12-like [Daucus carota subsp. sativus]	20.286	51.749	44.2	8	16	8	1.126	1.074	1.046	0.918	0.898	0.951	0.852	4.26E-03
cds.Camellia_sinensis.comp30206_c0_seq1_m.17707	PREDICTED: UDP-sulfoquinovose synthase, chloroplastic [Vitis vinifera]	54.099	99.483	24.5	10	25	10	1.011	1.001	0.994	1.009	1.007	0.983	0.998	8.19E-01
cds.Camellia_sinensis.comp30210_c0_seq1_m.20748	PREDICTED: aspartate aminotransferase [Theobroma cacao]	43.416	112.79	38.8	14	29	14	0.979	1.012	1.008	1.005	1.017	0.974	0.999	9.52E-01
cds.Camellia_sinensis.comp30210_c1_seq1_m.22782	PREDICTED: la protein 1 [Vitis vinifera]	53.937	15.402	9.2	4	4	4	1.199	1.172	1.103	0.824	0.767	0.883	0.712	2.04E-03

cds.Camellia_sinensis.comp30212_c0_seq1_m.7102	PREDICTED: heat shock protein 90-6, mitochondrial-like [Nicotiana tabacum]	90.064	113.73	24.8	16	31	12	1.062	1.061	1.058	0.96	0.951	0.974	0.907	1.58E-04
cds.Camellia_sinensis.comp30214_c0_seq2_m.11773	PREDICTED: acetyl-coenzyme A synthetase, chloroplastic/glyoxysomal isoform X1 [Juglans regia]	83.117	126.23	21.4	15	24	15	0.965	0.966	0.974	1.028	1.041	1.036	1.069	1.42E-04
cds.Camellia_sinensis.comp30217_c0_seq1_m.14326	PREDICTED: asparagine--tRNA ligase, cytoplasmic 2 [Sesamum indicum]	70.297	25.048	13.6	10	12	9	0.963	0.922	1.002	1.015	1.079	0.997	1.071	1.14E-01
cds.Camellia_sinensis.comp30219_c0_seq1_m.27886	PREDICTED: rho-N domain-containing protein 1, chloroplastic [Vitis vinifera]	40.079	4.7068	5.3	2	2	2	1.265	1.217	1.338	0.742	0.683	0.678	0.551	1.18E-04

cds.Camellia_sinensis.comp30223_c0_seq3_m.43652	PREDICTED: phospholipase D alpha 1 [Daucus carota subsp. sativus]	30.004	16.354	27.7	6	8	2	0.986	0.991	0.989	0.964	1.041	1.04	1.027	4.20E-01
cds.Camellia_sinensis.comp30236_c0_seq1_m.38327	PREDICTED: aspartokinase 2, chloroplastic isoform X2 [Vitis vinifera]	35.829	18.546	13	4	5	2	1.006	0.946	1.003	1.075	0.99	0.997	1.036	3.47E-01
cds.Camellia_sinensis.comp30238_c0_seq1_m.38070	PREDICTED: thylakoid lumenal 17.4 kDa protein, chloroplastic isoform X2 [Populus euphratica]	25.561	53.028	35.2	7	33	7	0.974	0.979	0.972	1.032	1.021	1.023	1.052	2.17E-04
cds.Camellia_sinensis.comp30246_c1_seq1_m.24373	PREDICTED: natterin-1-like [Zea mays]	51.883	1.7697	1.5	1	2	1	1.062	1.013	1.073	0.934	0.982	0.929	0.904	1.55E-02
cds.Camellia_sinensis.comp30254_c0_seq3_m.19705	PREDICTED: serine carboxypeptidase-like 27 [Vitis vinifera]	44.262	1.5842	4.1	2	2	2	1.029	1.018	0.996	0.958	1.048	0.953	0.972	4.22E-01

cds.Camellia_sinensis.comp30266_c0_seq1_m.19323	PREDICTED: MLO-like protein 10 [Theobroma cacao]	64.39	1.6435	1.6	1	1	1								
cds.Camellia_sinensis.comp30269_c0_seq5_m.34252	peptidoglycan-binding domain-containing family protein [Populus trichocarpa]	34.21	2.4845	13.4	4	5	2								
cds.Camellia_sinensis.comp30271_c0_seq4_m.31584	PREDICTED: PI-PLC X domain-containing protein At5g67130 [Juglans regia]	44.351	2.7778	3	1	1	1	0.936	1.003	0.948	1.028	1.039	1.068	1.086	2.64E-02
cds.Camellia_sinensis.comp30275_c0_seq1_m.22683	PREDICTED: UDP-glycosyltransferase 87A1-like [Fragaria vesca subsp. vesca]	54.242	18.609	13.3	6	7	6	1.011	0.996	1.016	1.04	0.947	0.975	0.98	4.97E-01

cds.Camellia_sinensis.comp30285_c0_seq2_m.40271	PREDICTED: glycine-rich RNA-binding protein 2-like [Sesamum indicum]	16.248	83.979	58.5	7	25	0
cds.Camellia_sinensis.comp30285_c0_seq3_m.43101	PREDICTED: glycine-rich RNA-binding protein GRP1A-like [Tarenaya hassleriana]	16.183	26.228	48.2	6	23	0
cds.Camellia_sinensis.comp30285_c0_seq5_m.48679	PREDICTED: glycine-rich RNA-binding protein 2-like [Sesamum indicum]	13.511	10.157	49.6	5	16	0

cds.Camellia_sinensis.comp30287_c0_seq1_m.20691	PREDICTED: probable tRNA N6-adenosine threonylcarbamoyltransferase, mitochondrial isoform X2 [Populus	52.535	7.6102	5.7	2	2	2								
cds.Camellia_sinensis.comp30289_c0_seq2_m.47577	PREDICTED: acyl-coenzyme A thioesterase 13-like [Pyrus x bretschneideri]	17.231	3.9146	6.3	1	1	1	1.04	1.002	0.965	1.023	0.991	0.984	0.997	9.19E-01
cds.Camellia_sinensis.comp30291_c0_seq1_m.11527	PREDICTED: primary amine oxidase-like [Juglans regia]	82.737	135.73	36.9	23	47	23	1.122	1.097	1.104	0.881	0.887	0.87	0.794	1.62E-05
cds.Camellia_sinensis.comp30298_c0_seq1_m.9588	ABC transporter E family member 2 [Morus notabilis]	68.221	32.534	14.9	10	12	10	1.022	1.008	1.032	0.958	0.984	1.028	0.97	2.27E-01

cds.Camellia_sinensis.comp30299_c1_seq1_m.29581	PREDICTED: vesicle-associated protein 4-2-like [Gossypium hirsutum]	29.469	9.2212	11.4	2	2	2	1.005	0.982	1.037	1.053	0.973	0.961	0.988	7.14E-01
cds.Camellia_sinensis.comp30303_c0_seq1_m.33137	PREDICTED: ER membrane protein complex subunit 2 [Ziziphus jujuba]	21.809	5.0825	7.6	1	1	1	0.984	0.979	0.882	0.958	1.118	1.094	1.114	1.49E-01
cds.Camellia_sinensis.comp30312_c0_seq1_m.6452	PREDICTED: non-lysosomal glucosylceramidase-like isoform X2 [Juglans regia]	109.58	78.849	14.8	11	18	11	0.983	0.993	0.985	0.997	1.009	1.031	1.026	7.01E-02
cds.Camellia_sinensis.comp30313_c0_seq1_m.35730	PREDICTED: mitochondrial import inner membrane translocase subunit TIM22 [Ziziphus jujuba]	27.225	88.401	29.7	7	17	7	0.977	0.938	0.961	1.064	1.045	1.011	1.085	1.30E-02

cds.Camellia_sinensis.comp30319_c0_seq2_m.36426	PREDICTED: protein SYM1 [Erythranthe guttata]	25.36	91.413	15.5	2	5	2	0.963	0.982	1.047	1.065	1.05	0.993	1.039	3.12E-01
cds.Camellia_sinensis.comp30320_c0_seq1_m.24022	PREDICTED: double-stranded RNA-binding protein 4 isoform X3 [Vitis vinifera]	39.391	5.0646	9.5	4	4	2	0.987	1.095	1.143	0.917	0.922	0.93	0.859	7.50E-02
cds.Camellia_sinensis.comp30323_c0_seq2_m.19870	PREDICTED: malonyl-CoA decarboxylase, mitochondrial [Vitis vinifera]	55.948	2.2638	1.6	1	1	1	0.93	1.048	0.974	1.168	0.928	0.979	1.042	6.58E-01
cds.Camellia_sinensis.comp30327_c1_seq1_m.25283	PREDICTED: PCI domain-containing protein 2 isoform X1 [Jatropha curcas]	48.184	2.7586	6	2	2	2	0.902	0.946	0.957	1.051	1.103	1.074	1.151	3.56E-03
cds.Camellia_sinensis.comp30328_c0_seq1_m.33208		27.843	16.187	17.3	4	5	4	1.03	1.015	0.994	1.018	0.975	0.99	0.982	3.17E-01

cds.Camellia_sinensis.comp30329_c0_seq1_m.9005	PREDICTED: peroxisome biogenesis protein 5 [Sesamum indicum]	85.257	4.4744	3.7	2	2	2	0.829	1.089	1.008	0.99	1.017	1.095	1.06	5.01E-01
cds.Camellia_sinensis.comp30330_c0_seq1_m.44090	PREDICTED: coiled-coil-helix-coiled-coil-helix domain-containing protein 10, mitochondrial [Sesamum indicum]	17.89	3.54	9.7	2	2	2	0.95	1.061	0.969	1.009	0.963	1.062	1.018	7.00E-01
cds.Camellia_sinensis.comp30332_c0_seq1_m.10145	PREDICTED: acetyl-coenzyme A carboxylase carboxyl transferase subunit alpha, chloroplastic-like [Prunus mume]	82.43	36.28	8	5	6	4	0.986	1.129	1.093	0.898	0.95	0.929	0.866	3.14E-02

cds.Camellia_sinensis.comp30335_c0_seq1_m.49908	UDP-glycosyltransferase 73C3 [Morus notabilis]	19.055	1.5191	4.7	1	2	1	1.077	1.043	1.093	0.939	0.94	0.897	0.864	2.12E-03
cds.Camellia_sinensis.comp30337_c0_seq3_m.29088	PREDICTED: LOW QUALITY PROTEIN: mannosyl-oligosaccharide glucosidase GCS1-like [Malus domestica]	39.557	1.4282	2.7	1	1	1	0.976	1.057	1.04	0.963	0.948	1.019	0.953	2.20E-01
cds.Camellia_sinensis.comp30339_c0_seq1_m.10719	PREDICTED: beta-glucosidase BoGH3B [Solanum pennellii]	91.302	15.231	6	5	7	5	1.04	1.074	1.053	0.956	0.91	0.945	0.888	2.46E-03
cds.Camellia_sinensis.comp30341_c1_seq1_m.22308	PREDICTED: fasciclin-like arabinogalactan protein 17 [Vitis vinifera]	49.243	4.6548	6.9	2	2	2	1.028	1.108	0.952	1.057	1.023	0.839	0.945	5.20E-01

cds.Camellia_sinensis.comp30341_c2_seq9_m.44946	PREDICTED: D-xylose-proton symporter-like 3, chloroplastic isoform X2 [Ipomoea nil]	17.358	20.362	28.9	4	6	4	0.995	0.977	0.985	0.99	1.084	1.013	1.044	2.02E-01
cds.Camellia_sinensis.comp30342_c0_seq1_m.25365	adenosine/AMP deaminase family protein [Populus trichocarpa]	41.05	6.0549	3	1	1	1								
cds.Camellia_sinensis.comp30347_c0_seq3_m.33116	PREDICTED: phosphoglucan phosphatase LSF2, chloroplastic isoform X1 [Gossypium raimondii]	31.254	6.7294	7.7	2	3	2	0.981	1.095	1.117	0.891	0.981	0.929	0.877	5.68E-02

cds.Camellia_sinensis.comp30349_c0_seq1_m.32860	PREDICTED: isopentenyl-diphosphate Delta-isomerase I [Vigna radiata var. radiata]	37.08	66.205	34.5	11	21	11	0.994	0.977	0.993	1.015	1.014	0.994	1.02	8.91E-02
cds.Camellia_sinensis.comp30350_c0_seq1_m.24392	PREDICTED: eukaryotic translation initiation factor 3 subunit M [Vitis vinifera]	46.528	69.886	27.1	8	14	8	0.901	0.94	1.007	1.072	1.028	1.103	1.125	3.69E-02
cds.Camellia_sinensis.comp30353_c0_seq1_m.34475	PREDICTED: chloroplast processing peptidase-like isoform X1 [Nicotiana tabacum]	34.383	30.025	25.9	5	7	5	1.055	0.971	1.009	0.955	1.01	1.004	0.978	5.07E-01
cds.Camellia_sinensis.comp30355_c1_seq2_m.33853	PREDICTED: F-box protein PP2-A15 [Malus domestica]	33.364	1.3189	4	1	1	1								

cds.Camellia_sinensis.comp30356_c0_seq1_m.13185	PREDICTED: vacuolar-sorting receptor 6 [Vitis vinifera]	70.812	19.802	18.6	11	12	11	0.915	0.967	0.935	1.065	1.065	1.105	1.148	2.38E-03
cds.Camellia_sinensis.comp30357_c0_seq1_m.16491	PREDICTED: dihydropyrimidinase [Vitis vinifera]	58.109	56.545	18.2	12	15	12	0.923	0.945	0.93	1.112	1.065	1.074	1.162	5.57E-04
cds.Camellia_sinensis.comp30362_c0_seq1_m.16010	PREDICTED: probable inositol transporter 2 [Nelumbo nucifera]	65.018	15.409	6.5	3	4	3	1.077	0.954	0.899	1.153	0.862	1.065	1.051	6.79E-01
cds.Camellia_sinensis.comp30363_c0_seq1_m.41907	PREDICTED: ferredoxin-1 [Gossypium hirsutum]	22.973	3.622	5.9	1	2	1								
cds.Camellia_sinensis.comp30366_c0_seq1_m.20288	PREDICTED: uridine 5'-monophosphate synthase-like [Ipomoea nil]	57.165	14.43	6.3	3	3	3	1.032	1.019	0.998	0.996	0.985	0.973	0.969	5.62E-02

cds.Camellia_sinensis.comp30367_c0_seq1_m.16928	PREDICTED: histone deacetylase 19 isoform X1 [Eucalyptus grandis]	52.827	3.5092	2.6	1	1	1	1.04	0.988	1.184	0.939	0.986	0.86	0.867	1.03E-01
cds.Camellia_sinensis.comp30373_c0_seq1_m.9012	PREDICTED: subtilisin-like protease SBT1.7 [Ricinus communis]	81.232	18.578	7.5	6	7	6	1.169	1.214	1.187	0.738	0.76	0.79	0.641	3.95E-05
cds.Camellia_sinensis.comp30374_c0_seq1_m.36757	PREDICTED: endo-1,3;1,4-beta-D-glucanase [Ricinus communis]	28.259	9.7754	12.7	3	6	3	0.939	0.945	0.91	1.067	1.115	1.075	1.166	1.02E-03
cds.Camellia_sinensis.comp30380_c0_seq1_m.8250	PREDICTED: CSC1-like protein ERD4 [Jatropha curcas]	81.653	80.882	26.7	23	41	23	1.007	1.018	1.035	0.973	0.992	1.005	0.971	7.25E-02
cds.Camellia_sinensis.comp30381_c0_seq1_m.28307	PREDICTED: protein trichome birefringence-like 41 [Ipomoea nil]	42.034	1.4934	2.2	1	1	1	1.154	1.115	1.229	0.828	0.804	0.826	0.703	3.01E-04

cds.Camellia_sinensis.comp30383_c0_seq1_m.25453	PREDICTED: guanine nucleotide-binding protein alpha-1 subunit [Jatropha curcas]	44.776	4.853	4.9	2	3	2	0.924	1.009	0.938	1.122	0.989	1.048	1.1	1.05E-01
cds.Camellia_sinensis.comp30386_c0_seq1_m.16307	PREDICTED: external alternative NAD(P)H-ubiquinone oxidoreductase B1, mitochondrial-like [Ipomoea	67.612	30.735	19.9	12	13	11	1	0.961	0.963	1.045	0.996	0.991	1.037	1.65E-01
cds.Camellia_sinensis.comp30390_c0_seq1_m.29285	PREDICTED: UPF0160 protein [Ziziphus jujuba]	41.607	21.467	13.6	4	6	4	0.955	1.008	1.036	0.995	1.072	1.002	1.023	5.31E-01
cds.Camellia_sinensis.comp30392_c0_seq1_m.33378	PREDICTED: RNA-binding protein 2-like isoform X2 [Juglans regia]	29.17	1.4747	15.2	5	6	2	1.017	0.967	0.984	1.005	0.999	1.038	1.025	2.63E-01

cds.Camellia_sinensis.comp30392_c0_seq2_m.44216	PREDICTED: RNA-binding protein 2-like isoform X2 [Juglans regia]	25.007	17.497	32.5	7	8	4	1.006	0.987	0.997	1.012	0.951	1.052	1.008	8.14E-01
cds.Camellia_sinensis.comp30396_c0_seq1_m.9383	PREDICTED: probable alpha,alpha-trehalose-phosphate synthase [UDP-forming] 7 isoform X3 [Pyrus x bretschneideri]	64.982	12.424	10.2	4	4	4	0.819	1.012	0.864	1.12	1.017	1.214	1.243	5.47E-02
cds.Camellia_sinensis.comp30396_c0_seq1_m.9384	PREDICTED: probable alpha,alpha-trehalose-phosphate synthase [UDP-forming] 7, partial [Jatropha curcas]	19.457	1.5727	5.2	1	1	1	0.904	0.945	0.933	1.151	1.061	1.047	1.171	8.28E-03

cds.Camellia_sinensis.comp30398_c0_seq1_m.22768	PREDICTED: aldehyde dehydrogenase family 2 member C4 [Ricinus communis]	54.268	11.236	13.2	6	6	5	1.003	0.973	1.021	0.954	1.025	0.984	0.989	6.67E-01
cds.Camellia_sinensis.comp30402_c0_seq2_m.38034	PREDICTED: glycine cleavage system H protein 3, mitochondrial [Juglans regia]	17.511	56.766	26.7	4	22	4	1.024	1.004	1.034	0.987	0.964	0.991	0.961	3.07E-02
cds.Camellia_sinensis.comp30402_c0_seq3_m.46558	PREDICTED: glycine cleavage system H protein 3, mitochondrial [Pyrus x bretschneideri]	16.447	54.235	26.6	4	18	4	0.965	0.971	0.988	1.03	1.019	1.057	1.062	9.62E-03
cds.Camellia_sinensis.comp30405_c0_seq2_m.5927	PREDICTED: ubiquinone biosynthesis protein COQ9-B, mitochondrial [Vitis	35.093	63.47	19.2	6	7	6	0.966	0.989	0.974	1.04	1.012	0.993	1.04	6.23E-02

cds.Camellia_sinensis.comp30409_c1_seq1_m.20071	PREDICTED: 2-methyl-6-phytyl-1,4-hydroquinone methyltransferase, chloroplastic-like [Populus euphratica]	38.711	77.336	21.6	6	17	1	0.986	1.015	1.046	0.989	1.016	0.972	0.977	3.40E-01
cds.Camellia_sinensis.comp30409_c1_seq3_m.41209	PREDICTED: 2-methyl-6-phytyl-1,4-hydroquinone methyltransferase, chloroplastic [Theobroma cacao]	22.705	88.828	38.4	6	11	3	1.017	1.011	1.053	0.949	1.006	1.006	0.961	1.61E-01
cds.Camellia_sinensis.comp30411_c1_seq1_m.31103	PREDICTED: 60S ribosomal protein L5 [Vitis vinifera]	35.38	30.696	16.8	6	12	4	0.974	0.94	0.947	1.074	1.051	1.022	1.1	6.12E-03
cds.Camellia_sinensis.comp30411_c1_seq2_m.34917	PREDICTED: 60S ribosomal protein L5 [Populus euphratica]	34.522	3.8403	14.6	4	8	2	0.95	0.961	0.961	1.139	1.002	1.017	1.1	1.49E-01

cds.Camellia_sinensis.comp30412_c0_seq1_m.51782	PREDICTED: 17.3 kDa class I heat shock protein-like [Nelumbo nucifera]	11.746	3.7243	14.9	1	1	1	1.052	1.083	1.118	0.874	1.036	0.825	0.841	6.53E-02
cds.Camellia_sinensis.comp30417_c0_seq1_m.20260	PREDICTED: aspartyl protease family protein At5g10770 [Vitis vinifera]	50.917	84.664	21.8	9	24	9	1.052	1.071	1.009	0.931	0.959	0.963	0.911	1.07E-02
cds.Camellia_sinensis.comp30418_c0_seq1_m.10541	PREDICTED: ABC transporter F family member 1 [Eucalyptus grandis]	53.778	23.342	17.4	8	9	8	0.914	1.022	1.027	1.028	1.009	0.988	1.021	6.09E-01
cds.Camellia_sinensis.comp30419_c0_seq1_m.29507	PREDICTED: bifunctional monothiol glutaredoxin-S16, chloroplastic [Prunus mume]	37.07	12.515	20.2	6	8	6	1.005	1.019	1.058	0.958	0.974	0.959	0.938	1.72E-02

cds.Camellia_sinensis.comp30422_c0_seq1_m.48267	PREDICTED: dynein light chain 1, cytoplasmic [Jatropha	14.258	3.083	17.3	2	2	2	0.994	0.966	0.987	0.996	1.004	1.064	1.04	1.62E-01
cds.Camellia_sinensis.comp30426_c0_seq1_m.40832	PREDICTED: ras-related protein RABA2a [Prunus mume]	23.839	89.244	49.3	9	18	1	1.034	0.971	1.028	0.99	0.972	1.009	0.98	4.22E-01
cds.Camellia_sinensis.comp30426_c0_seq2_m.45216	PREDICTED: ras-related protein RABA2a-like [Gossypium arboreum]	21.7	13.265	52.8	9	14	2	0.868	0.956	0.987	1.017	1.079	1.128	1.147	4.81E-02
cds.Camellia_sinensis.comp30427_c0_seq1_m.28594	uncharacterized LOC102580903 [Solanum tuberosum	41.216	112.52	43.6	14	32	14	0.985	0.956	0.989	1.014	1.045	1.01	1.047	3.79E-02
cds.Camellia_sinensis.comp30428_c0_seq1_m.32708	PREDICTED: rhodanese-like/PpiC domain-containing protein 12, chloroplastic [Prunus mume]	25.507	28.719	31.7	6	8	6	1.002	1.031	1.042	1.004	0.963	1.003	0.966	1.26E-01

cds.Camellia_sinensis.comp30429_c1_seq1_m.31899	PREDICTED: caffeic acid 3-O-methyltransferase [Vitis vinifera]	38.272	65.214	43.7	13	35	13	1.045	1.036	1.039	0.965	0.956	0.945	0.919	2.03E-04
cds.Camellia_sinensis.comp30431_c0_seq1_m.9052	PREDICTED: DEAD-box ATP-dependent RNA helicase 3, chloroplastic isoform X1 [Nelumbo nucifera]	81.033	54.478	25.5	15	25	10	1.105	1.116	1.145	0.889	0.847	0.886	0.779	1.84E-04
cds.Camellia_sinensis.comp30431_c0_seq2_m.9474	PREDICTED: DEAD-box ATP-dependent RNA helicase 3, chloroplastic isoform X1 [Nelumbo nucifera]	80.434	3.4553	10.4	6	10	1	1.116	1.002	1.105	0.986	0.903	0.875	0.858	3.62E-02
cds.Camellia_sinensis.comp30434_c0_seq1_m.24447	3-oxoacyl-[acyl-carrier-protein] reductase [Morus notabilis]	34.44	40.791	14.8	4	9	3	0.997	0.931	1.03	1.048	1.098	1.041	1.077	9.27E-02

cds.Camellia_sinensis.comp30436_c0_seq15_m.2985	Aquaporin PIP2-7 [Morus notabilis]	31.629	36.815	12.5	3	9	3	1	0.96	0.954	1.003	1.016	1.065	1.058	7.34E-02
cds.Camellia_sinensis.comp30436_c0_seq16_m.31026	PREDICTED: eukaryotic translation initiation factor 3 subunit F [Daucus carota subsp. sativus]	31.338	12.763	25.3	8	14	8	0.966	1.003	0.951	1.023	1.029	1.033	1.057	2.63E-02
cds.Camellia_sinensis.comp30436_c0_seq28_m.56874	Histone superfamily protein [Arabidopsis thaliana]	13.537	10.603	27.5	5	14	3	1.189	1.173	1.1	0.883	0.792	0.779	0.709	1.70E-03
cds.Camellia_sinensis.comp30436_c0_seq9_m.19783	PREDICTED: histone H3.2-like [Nicotiana tabacum]	15.302	1.8199	16.9	3	7	1	1.087	1.076	1.122	0.816	0.96	0.914	0.819	1.54E-02
cds.Camellia_sinensis.comp30436_c2_seq1_m.16118	PREDICTED: proline iminopeptidase isoform X1 [Prunus	60.755	2.663	2	1	1	1	1.053	0.969	1.008	1.01	0.935	1.026	0.981	6.23E-01

cds.Camellia_sinensis.comp30439_c0_seq1_m.25477	PREDICTED: mannose-1-phosphate guanylyltransferase 1 [Daucus carota subsp. sativus]	39.599	17.748	13.6	4	6	4	1	1.008	1.066	0.949	1.004	0.95	0.944	1.05E-01
cds.Camellia_sinensis.comp30443_c0_seq1_m.32367	PREDICTED: proteasome subunit beta type-3-A [Sesamum indicum]	22.767	5.083	6.9	1	2	1	0.9	0.956	0.952	1.038	1.042	1.143	1.148	2.07E-02
cds.Camellia_sinensis.comp30443_c0_seq2_m.35514	PREDICTED: proteasome subunit beta type-3-A [Sesamum indicum]	22.81	5.7541	6.9	1	1	1	0.711	1.289	0.839	1.115	0.815	1.269	1.127	5.74E-01
cds.Camellia_sinensis.comp30445_c0_seq1_m.27397	PREDICTED: splicing factor 3B subunit 4 [Nelumbo nucifera]	40.257	47.242	13.9	4	5	4	0.957	1.011	1.042	0.967	1.046	1.06	1.021	6.18E-01

cds.Camellia_sinensis.comp30448_c0_seq1_m.13595	PREDICTED: bifunctional purine biosynthesis protein PurH-like isoform X2 [Gossypium arboreum]	65.947	268.92	45.2	25	67	25	0.982	0.997	0.975	1.007	1.023	1.035	1.038	2.33E-02
cds.Camellia_sinensis.comp30451_c0_seq1_m.44919	PREDICTED: glyceraldehyde-3-phosphate dehydrogenase, cytosolic [Capsicum annuum]	14.387	2.1733	26.7	4	7	2	1.06	1.052	1.072	1.028	0.891	0.892	0.883	1.17E-01
cds.Camellia_sinensis.comp30452_c0_seq1_m.32345	PREDICTED: methyl-CpG-binding domain-containing protein 11 [Vitis vinifera]	34.5	18.045	16.8	5	10	2	1.24	1.175	1.188	0.747	0.82	0.788	0.654	1.78E-04

cds.Camellia_sinensis.comp30452_c0_seq2_m.40812	PREDICTED: methyl-CpG-binding domain-containing protein 11 [Vitis vinifera]	29.818	34.764	28.8	6	8	5	1.237	1.268	1.251	0.782	0.683	0.745	0.588	1.85E-04
cds.Camellia_sinensis.comp30453_c0_seq1_m.32376	PREDICTED: bifunctional epoxide hydrolase 2-like [Capsicum annuum]	37.333	3.0489	5.2	1	1	1	1.016	1.276	0.888	1.011	0.692	1.102	0.882	4.94E-01
cds.Camellia_sinensis.comp30453_c1_seq1_m.29710	PREDICTED: probable 2-oxoglutarate-dependent dioxygenase AOP1 [Vitis vinifera]	22.474	10.463	18.7	4	4	4	1.142	1.146	1.222	0.804	0.807	0.834	0.697	1.36E-04
cds.Camellia_sinensis.comp30454_c1_seq1_m.18257	PREDICTED: dihydrolipoyl dehydrogenase 1, mitochondrial [Ricinus communis]	53.812	201.3	55	20	60	20	1.045	1.002	1.02	0.979	0.979	0.975	0.956	6.81E-02

cds.Camellia_sinensis.comp30456_c0_seq1_m.758	PREDICTED: callose synthase 3-like [Sesamum indicum]	174.78	3.4894	1.2	2	2	2	0.977	1.022	0.983	1.002	1.012	1.015	1.016	3.40E-01
cds.Camellia_sinensis.comp30457_c0_seq1_m.29771	PREDICTED: syntaxin-71-like [Pyrus x bretschneideri]	29.915	48.741	39.1	9	11	9	0.998	1.02	1.065	0.971	0.981	0.966	0.946	4.95E-02
cds.Camellia_sinensis.comp30462_c1_seq2_m.51610	PREDICTED: protein FATTY ACID EXPORT 1, chloroplastic [Vitis vinifera]	17.104	36.46	17.2	2	2	2	0.995	1.034	0.966	0.979	1.077	0.96	1.007	8.88E-01
cds.Camellia_sinensis.comp30465_c2_seq2_m.32745	PREDICTED: enolase [Prunus mume]	41.332	323.31	70	24	68	18	1.022	1.011	0.997	0.966	0.973	0.989	0.966	2.66E-02
cds.Camellia_sinensis.comp30466_c0_seq1_m.19041	PREDICTED: purple acid phosphatase 18 [Prunus mume]	48.915	16.123	8.1	3	5	3	0.958	1.002	0.952	1.069	0.977	1.043	1.061	1.35E-01

cds.Camellia_sinensis.comp30467_c1_seq1_m.34326	PREDICTED: homeobox protein BEL1 homolog [Juglans regia]	44.679	2.0357	2.3	1	1	1	0.998	1.06	1.036	0.963	0.962	0.983	0.94	3.10E-02
cds.Camellia_sinensis.comp30467_c2_seq1_m.40185	PREDICTED: ATP synthase subunit d, mitochondrial-like [Gossypium hirsutum]	24.9	117.83	63.8	17	42	15	1.049	1.01	1.039	0.974	0.98	0.966	0.943	8.18E-03
cds.Camellia_sinensis.comp30468_c0_seq2_m.34663	chalcone--flavonone isomerase 2 [Vitis vinifera]	24.735	189.7	55.2	10	24	10	1.205	1.153	1.183	0.791	0.814	0.811	0.682	1.83E-05
cds.Camellia_sinensis.comp30469_c0_seq1_m.14434	PREDICTED: probable mitochondrial-processing peptidase subunit beta, mitochondrial [Vitis vinifera]	63.829	180.92	31.8	19	46	17	0.995	1.037	1.007	1.003	0.986	0.984	0.978	1.86E-01
cds.Camellia_sinensis.comp30471_c0_seq1_m.40186	40S ribosomal protein S19-3 [Cajanus cajan]	16.159	88.633	65.5	12	22	12	0.93	0.957	0.91	1.074	1.071	1.064	1.147	7.56E-04

cds.Camellia_sinensis.comp30472_c0_seq1_m.38586	PREDICTED: glutathione S-transferase zeta class-like isoform X1 [Sesamum indicum]	25.363	2.3747	4.4	1	1	1								
cds.Camellia_sinensis.comp30472_c3_seq2_m.23353	PREDICTED: pectin acetylesterase 8 [Vitis vinifera]	43.665	91.807	38.3	12	19	12	1.154	1.166	1.154	0.829	0.845	0.813	0.716	4.41E-06
cds.Camellia_sinensis.comp30478_c0_seq1_m.35357	PREDICTED: ribonuclease 2 [Populus euphratica]	32.03	7.2942	7.3	2	3	1	1.044	1.007	1.012	0.962	1.019	0.956	0.959	1.43E-01
cds.Camellia_sinensis.comp30478_c0_seq2_m.35405	PREDICTED: ribonuclease 2 isoform X1 [Daucus carota subsp. sativus]	29.591	2.0584	9	2	3	1								

cds.Camellia_sinensis.comp30479_c0_seq3_m.39564	PREDICTED: translation initiation factor eIF-2B subunit beta-like, partial [Prunus mume]	22.195	3.7682	10.6	2	2	1								
cds.Camellia_sinensis.comp30492_c0_seq4_m.27379	PREDICTED: photosynthetic NDH subunit of lumenal location 3, chloroplastic [Nelumbo nucifera]	17.619	31.666	34.9	5	6	5	0.97	0.976	1.001	0.972	1.046	1.096	1.057	2.11E-01
cds.Camellia_sinensis.comp30506_c0_seq2_m.33369	PREDICTED: glucose-induced degradation protein 8 homolog isoform X1 [Citrus sinensis]	27.493	17.758	23.6	4	4	4	1.032	1	0.988	1.004	0.989	0.991	0.988	4.40E-01

cds.Camellia_sinensis.comp30508_c0_seq2_m.12649	PREDICTED: methylcrotonoyl-CoA carboxylase beta chain, mitochondrial [Jatropha curcas]	63.401	70.189	25.3	12	14	12	0.987	1.021	0.953	1.021	0.997	0.999	1.019	4.21E-01
cds.Camellia_sinensis.comp30509_c0_seq1_m.20039	peptidase M50B-like protein [Medicago truncatula]	25.747	9.1807	5.2	1	1	1	1.114	0.956	0.87	1.027	1.086	0.952	1.043	6.07E-01
cds.Camellia_sinensis.comp30517_c0_seq1_m.10671	PREDICTED: zinc finger BED domain-containing protein DAYSLEEPER-like [Vitis vinifera]	76.909	1.7207	6.4	4	6	2	1.036	0.974	1.012	0.973	0.968	0.996	0.972	2.30E-01

cds.Camellia_sinensis.comp30525_c0_seq1_m.4935	PREDICTED: probable inactive ATP-dependent zinc metalloprotease FTSHI 1, chloroplastic [Nicotiana attenuata]	107.86	17.409	6.8	6	7	6	1.05	0.998	1.041	0.972	0.954	0.936	0.927	1.63E-02
cds.Camellia_sinensis.comp30526_c0_seq1_m.17626	PREDICTED: CDK5RAP3-like protein [Theobroma cacao]	64.311	9.7735	9.1	6	7	6	0.969	0.98	0.963	1.041	1.046	0.996	1.059	2.57E-02
cds.Camellia_sinensis.comp30528_c0_seq1_m.29937	PREDICTED: E3 ubiquitin-protein ligase At1g63170-like [Ipomoea nil]	37.631	4.0865	10.6	2	2	2								
cds.Camellia_sinensis.comp30532_c0_seq1_m.22428	PREDICTED: calcium-transporting ATPase 1, chloroplastic [Solanum pennellii]	47.557	4.5251	6.3	3	4	3	0.928	0.925	0.969	1.038	1.086	1.084	1.137	3.66E-03

cds.Camellia_sinensis.comp30548_c0_seq1_m.6858	PREDICTED: death-inducer obliterator 1 [Theobroma cacao]	98.869	3.2742	1.4	1	1	1	0.734	1.097	0.944	0.92	1.228	1.121	1.178	3.05E-01
cds.Camellia_sinensis.comp30551_c0_seq1_m.6176	PREDICTED: protein KAKU4 isoform X4 [Citrus sinensis]	38.078	1.4118	3.2	1	1	1								
cds.Camellia_sinensis.comp30554_c0_seq4_m.29696	PREDICTED: protein TIC 22, chloroplastic isoform X1 [Vitis vinifera]	33.394	25.864	18.9	5	10	5	0.933	1	0.92	1.008	1.021	0.995	1.06	9.34E-02
cds.Camellia_sinensis.comp30561_c0_seq1_m.15465	PREDICTED: alanine aminotransferase 2-like [Nicotiana tabacum]	56.024	236.88	30.6	12	26	12	0.966	0.964	0.933	1.049	1.049	1.011	1.086	7.80E-03
cds.Camellia_sinensis.comp30562_c0_seq1_m.16496	PREDICTED: ultraviolet-B receptor UVR8 isoform X1 [Ziziphus jujuba]	65.869	4.5542	3.6	2	2	2	0.974	0.974	1.01	1.031	0.974	1.053	1.034	2.79E-01

cds.Camellia_sinensis.comp30571_c0_seq1_m.31708	PREDICTED: 5'-methylthioadenosine/S-adenosylhomocysteine nucleosidase 1-like isoform X1 [Juglans	32.258	15.685	19.6	4	7	4	0.939	0.963	0.979	1.056	1.039	1.076	1.101	3.70E-03
cds.Camellia_sinensis.comp30573_c0_seq2_m.39452	PREDICTED: 30S ribosomal protein S10, chloroplastic [Juglans regia]	21.737	2.0996	8.5	2	5	2	1.019	1.101	1.025	0.901	0.975	0.979	0.908	5.78E-02
cds.Camellia_sinensis.comp30577_c0_seq1_m.21840	PREDICTED: phenylalanine--tRNA ligase, chloroplastic/mitochondrial [Vitis vinifera]	52.454	61.358	32	12	16	12	1.008	0.998	0.985	0.986	1.021	1.003	1.006	6.31E-01
cds.Camellia_sinensis.comp30579_c0_seq4_m.29219	Nuclear transport factor 2 (NTF2) family protein [Arabidopsis thaliana]	32.174	11.875	11.6	3	4	3	1.033	1.125	0.967	0.968	0.933	1.01	0.932	2.32E-01

cds.Camellia_sinensis.comp30587_c0_seq1_m.21326	PREDICTED: aspartic proteinase PCS1-like [Populus euphratica]	49.902	5.6992	5.4	2	4	2	0.928	0.999	0.957	1.058	1.007	1.037	1.076	4.69E-02
cds.Camellia_sinensis.comp30589_c0_seq1_m.21761	PREDICTED: diacylglycerol kinase 5-like [Nicotiana tabacum]	54.002	1.2213	3.1	2	2	2	1.079	1.129	1.099	0.867	0.915	0.888	0.807	4.63E-04
cds.Camellia_sinensis.comp30593_c0_seq1_m.41199	PREDICTED: crocetin glucosyltransferase, chloroplastic-like [Juglans regia]	26.552	3.7718	11.3	3	4	3	0.928	0.956	0.981	1.061	1.04	1.072	1.108	5.08E-03
cds.Camellia_sinensis.comp30601_c0_seq1_m.4143	PREDICTED: zinc protease PQQL-like isoform X3 [Nicotiana tomentosiformis]	51.213	3.0835	3.7	2	2	2	0.929	0.961	1.014	0.998	1.083	1.039	1.074	1.07E-01

cds.Camellia_sinensis.comp30604_c1_seq3_m.17421	PREDICTED: polyadenylate-binding protein RBP45-like [Ipomoea	39.706	17.958	7.9	3	8	3	0.963	0.967	1.014	1.045	1.085	0.997	1.062	1.12E-01
cds.Camellia_sinensis.comp30604_c1_seq5_m.19357	PREDICTED: polyadenylate-binding protein RBP45 [Daucus carota subsp.	49.307	47.177	13.4	5	10	5	1.009	0.971	0.969	0.996	1.01	1.083	1.047	1.89E-01
cds.Camellia_sinensis.comp30614_c0_seq1_m.48786		13.261	8.15	23.8	3	3	3	1.088	1.026	1.057	0.908	0.918	0.987	0.887	1.82E-02
cds.Camellia_sinensis.comp30614_c0_seq2_m.53959	PREDICTED: inactive protein RESTRICTED TEV MOVEMENT 2 [Solanum tuberosum	12.797	5.5342	18.8	3	3	3	1.025	1.142	1.068	0.893	0.925	0.901	0.84	6.50E-03

cds.Camellia_sinensis.comp30618_c0_seq3_m.37107	PREDICTED: 5-formyltetrahydrofolate cyclo-ligase, mitochondrial-like [Nicotiana tabacum]	26.275	4.752	11.9	2	3	2	0.872	0.983	0.987	1.272	0.946	0.988	1.128	3.24E-01
cds.Camellia_sinensis.comp30619_c0_seq1_m.21275	PREDICTED: pentatricopeptide repeat-containing protein At4g35850, mitochondrial-like [Nicotiana tabacum]	53.506	6.6988	8	4	5	4	0.95	0.981	1.036	1.033	1.011	1.009	1.029	3.31E-01
cds.Camellia_sinensis.comp30621_c0_seq1_m.31503	zinc finger family protein [Populus trichocarpa]	34.394	1.8312	2.4	1	1	1	1.091	1.105	1.078	0.962	0.868	0.876	0.827	4.68E-03
cds.Camellia_sinensis.comp30622_c0_seq1_m.27600	PREDICTED: phosphoglycerate mutase-like protein 1 isoform X1 [Sesamum indicum]	31.245	27.202	33.3	7	11	7	1.02	1.006	0.963	0.979	0.953	1.062	1.002	9.82E-01

cds.Camellia_sinensis.comp30625_c0_seq1_m.28951	PREDICTED: uncharacterized methyltransferase At2g41040, chloroplastic [Sesamum indicum]	38.227	22.256	16	5	5	5	0.938	0.905	1.009	1.1	1.037	1.047	1.116	3.97E-02
cds.Camellia_sinensis.comp30629_c0_seq1_m.29610	Protein phosphatase 1 regulatory subunit pprA [Morus notabilis]	40.768	1.5357	2.2	1	1	1	0.951	0.878	0.966	1.148	1.066	1.028	1.16	2.80E-02
cds.Camellia_sinensis.comp30630_c0_seq1_m.10372	PREDICTED: nuclear pore complex protein NUP1 isoform X1 [Vitis vinifera]	83.744	4.1297	4.4	3	3	3	0.997	1.023	1.003	0.978	1.023	0.984	0.987	4.71E-01
cds.Camellia_sinensis.comp30631_c0_seq1_m.33662	PREDICTED: 2-oxoglutarate-dependent dioxygenase DAO [Vitis vinifera]	33.894	2.4382	3.9	1	1	1	0.88	1.056	1.003	1.102	1.011	0.979	1.052	4.64E-01

cds.Camellia_sinensis.comp30637_c0_seq6_m.11237	PREDICTED: 65-kDa microtubule-associated protein 3 [Nelumbo nucifera]	46.281	1.3484	1.7	1	1	1	1.05	1.051	0.993	0.924	0.986	0.989	0.937	8.80E-02
cds.Camellia_sinensis.comp30641_c0_seq2_m.27057	alanine-tRNA ligase [Arabidopsis thaliana]	29.894	43.331	19.8	6	7	6	1.043	1.031	1.012	0.982	0.951	0.962	0.938	7.64E-03
cds.Camellia_sinensis.comp30651_c0_seq1_m.16356	PREDICTED: tetratricopeptide repeat protein 7A-like [Juglans regia]	63.721	1.8398	1.2	1	4	1	0.963	0.974	0.923	1.015	1.045	1.077	1.097	1.75E-02
cds.Camellia_sinensis.comp30665_c0_seq1_m.37214	PREDICTED: nifU-like protein 1, chloroplastic [Juglans regia]	22.681	8.9056	18.2	3	3	3	0.975	0.98	1.001	1.102	1.003	0.961	1.037	4.44E-01

cds.Camellia_sinensis.comp30675_c0_seq1_m.8779	PREDICTED: 1,4-alpha-glucan-branching enzyme 1, chloroplastic/amyloplastic isoform X1 [Vitis vinifera]	96.568	48.838	20.9	18	20	18	1.139	1.137	1.156	0.85	0.833	0.833	0.733	1.71E-06
cds.Camellia_sinensis.comp30678_c0_seq2_m.27253	PREDICTED: maltose excess protein 1, chloroplastic isoform X1 [Ricinus communis]	30.645	4.1173	4.6	1	1	1	1.029	1.064	1.021	0.975	0.95	0.959	0.926	6.54E-03
cds.Camellia_sinensis.comp30683_c0_seq1_m.31687	PREDICTED: peroxidase 72-like [Nelumbo nucifera]	40.265	117.69	36.5	11	24	11	0.95	0.936	0.952	1.068	1.054	1.064	1.123	6.15E-05
cds.Camellia_sinensis.comp30689_c0_seq1_m.27969	nucleotidyltransferase [Medicago truncatula]	44.514	44.555	24.8	10	14	10	0.953	0.977	0.998	1.062	1.043	1.032	1.071	1.18E-02
cds.Camellia_sinensis.comp30697_c0_seq1_m.25600	PREDICTED: SEC12-like protein 2 [Nelumbo nucifera]	31.402	4.2576	7.3	2	2	2	1.072	0.954	1.009	0.943	0.956	1.06	0.975	6.35E-01

cds.Camellia_sinensis.comp30699_c1_seq1_m.13420	PREDICTED: glutamyl-tRNA(Gln) amidotransferase subunit B, chloroplastic/mitochondrial [Theobrom	60.706	60.371	24.5	13	25	13	1.018	1.011	1.024	0.965	0.989	0.981	0.961	8.26E-03
cds.Camellia_sinensis.comp30702_c0_seq1_m.38848	PREDICTED: DNA repair RAD52-like protein 1, mitochondrial [Vitis vinifera]	22.419	12.705	17.5	4	6	4	1.03	1.02	1.041	0.977	0.958	0.95	0.933	2.48E-03
cds.Camellia_sinensis.comp30722_c0_seq1_m.25310	PREDICTED: lecithin-cholesterol acyltransferase-like 1 [Juglans regia]	33.047	3.6446	6	2	3	2	1.025	1.035	1.014	1.005	0.972	0.953	0.953	4.40E-02
cds.Camellia_sinensis.comp30722_c0_seq1_m.25311	PREDICTED: lecithin-cholesterol acyltransferase-like 1 [Juglans regia]	18.598	5.0967	11.8	2	2	2	1.057	1.122	1.026	0.973	0.915	0.896	0.869	1.76E-02

cds.Camellia_sinensis.comp30729_c0_seq15_m.47517	PREDICTED: callose synthase 2-like [Camelina sativa]	13.925	5.806	9.1	1	1	1	1.057	0.946	0.948	1.035	0.961	1.06	1.036	4.93E-01
cds.Camellia_sinensis.comp30732_c0_seq1_m.1325	PREDICTED: auxin transport protein BIG [Vitis vinifera]	186.72	3.7505	1.4	2	2	2								
cds.Camellia_sinensis.comp30736_c0_seq1_m.27581	PREDICTED: protein transport protein SEC13 homolog B-like [Malus domestica]	32.894	2.8494	12	3	9	1	0.938	0.92	0.977	1.051	1.127	1.018	1.127	2.68E-02
cds.Camellia_sinensis.comp30740_c0_seq1_m.25588	PREDICTED: grpE protein homolog, mitochondrial isoform X2 [Sesamum	26.957	25.219	9.1	2	3	2	1.025	1.07	1.015	0.948	0.911	0.992	0.917	4.14E-02
cds.Camellia_sinensis.comp30740_c0_seq1_m.25589	PREDICTED: protein GrpE-like isoform X2 [Cucumis melo]	17.072	34.481	36.4	7	10	7	1.034	1.038	1.083	0.966	0.925	0.953	0.901	6.12E-03

cds.Camellia_sinensis.comp30742_c0_seq1_m.24774	PREDICTED: isovaleryl-CoA dehydrogenase, mitochondrial [Ipomoea nil]	44.591	14.053	13.2	5	5	5	1.01	1.055	0.99	0.955	1.002	0.992	0.965	2.14E-01
cds.Camellia_sinensis.comp30748_c0_seq1_m.15876	PREDICTED: DEAD-box ATP-dependent RNA helicase 47A [Vitis vinifera]	32.902	2.8592	4	1	1	1								
cds.Camellia_sinensis.comp30770_c0_seq2_m.37962	PREDICTED: signal peptide peptidase [Ziziphus jujuba]	30.063	6.6714	6.7	2	2	2	1.005	0.902	0.941	1.146	1.03	1.004	1.117	9.94E-02
cds.Camellia_sinensis.comp30776_c0_seq8_m.41618	PREDICTED: protein RER1B [Theobroma cacao]	13.437	1.2283	8.6	1	1	1								
cds.Camellia_sinensis.comp30783_c0_seq1_m.21832	PREDICTED: tafazzin isoform X2 [Vitis vinifera]	42.042	2.0388	2.4	1	1	1								

cds.Camellia_sinensis.comp30788_c0_seq1_m.9388	PREDICTED: uncharacterized aarF domain-containing protein kinase At4g31390, chloroplastic [Vitis vinifera]	77.309	22.392	17	11	13	11	0.947	0.987	0.966	1.019	1.044	1.051	1.074	9.38E-03
cds.Camellia_sinensis.comp30792_c0_seq1_m.34107	PREDICTED: alkane hydroxylase MAH1-like [Nicotiana tabacum]	31.537	3.2288	7	2	2	2	0.863	0.931	0.917	1.208	1.004	1.127	1.232	2.47E-02
cds.Camellia_sinensis.comp30800_c0_seq1_m.32560	PREDICTED: phytanoyl-CoA dioxygenase [Prunus mume]	19.952	5.5707	11.3	3	3	3	0.902	0.991	1.027	1.086	1.018	1.006	1.065	2.32E-01
cds.Camellia_sinensis.comp30804_c0_seq1_m.38835	transcription termination factor family protein [Medicago truncatula]	24.086	21.122	18.3	5	5	5	1.032	1.037	1.024	0.997	0.966	0.975	0.95	6.92E-03

cds.Camellia_sinensis.comp30816_c0_seq1_m.18539	PREDICTED: probable sodium/metabolite cotransporter BASS3, chloroplastic [Sesamum indicum]	45.167	3.1264	3.7	1	2	1	0.994	1.088	0.887	0.884	1.09	1.058	1.021	8.29E-01
cds.Camellia_sinensis.comp30825_c0_seq1_m.25138	PREDICTED: protein MULTIPLE CHLOROPLAST DIVISION SITE 1-like isoform X2 [Nicotiana tabacum]	16.29	5.6382	6.9	1	1	1	0.936	1.197	0.989	0.886	0.978	1.012	0.921	4.08E-01
cds.Camellia_sinensis.comp30830_c0_seq3_m.33258	PREDICTED: calcineurin B-like protein 3 [Daucus carota subsp. sativus]	26.259	10.221	17.3	5	5	5	0.972	0.958	0.981	1.032	1.041	1.043	1.07	8.77E-04

cds.Camellia_sinensis.comp30833_c0_seq2_m.38525	PREDICTED: trafficking protein particle complex subunit 3 [Theobroma cacao]	20.817	11.371	40.3	7	8	7	0.984	0.965	0.98	1.017	0.984	1.022	1.032	7.70E-02
cds.Camellia_sinensis.comp30836_c0_seq2_m.13616	PREDICTED: calcium-dependent protein kinase 2-like [Nelumbo nucifera]	59.549	7.6811	12.7	5	6	4	0.953	0.929	0.95	1.043	1.09	1.076	1.133	1.24E-03
cds.Camellia_sinensis.comp30836_c0_seq3_m.13814	PREDICTED: calcium-dependent protein kinase 2-like [Nelumbo nucifera]	59.134	40.29	21.1	10	13	4	0.995	0.985	0.986	1.001	1.047	1.004	1.029	1.29E-01
cds.Camellia_sinensis.comp30836_c0_seq8_m.22506	PREDICTED: calcium-dependent protein kinase 2-like [Nelumbo nucifera]	55.148	23.117	18.4	9	10	4	0.99	0.991	1.023	1.008	0.98	1.003	0.996	7.72E-01

cds.Camellia_sinensis.comp30836_c1_seq1_m.24886	PREDICTED: post-GPI attachment to proteins factor 3 [Jatropha curcas]	39.38	24.268	8.5	2	3	2	0.93	1.096	0.927	0.997	1.045	1.022	1.038	5.23E-01
cds.Camellia_sinensis.comp30842_c0_seq1_m.30149	PREDICTED: peroxidase N1 [Solanum tuberosum	38.299	17.217	14.1	4	4	4	0.854	0.979	0.991	1.019	1.102	1.091	1.137	7.20E-02
cds.Camellia_sinensis.comp30843_c0_seq1_m.19911	PREDICTED: COP9 signalosome complex subunit 1 [Vitis	50.277	21.537	11.8	5	5	5	0.918	0.971	0.973	1.051	1.065	1.045	1.104	7.40E-03
cds.Camellia_sinensis.comp30846_c0_seq1_m.36865	PREDICTED: psbP domain-containing protein 7, chloroplastic [Vitis vinifera]	30.996	41.726	37.1	9	11	9	0.997	1.012	0.961	0.999	0.997	1.012	1.013	4.66E-01

cds.Camellia_sinensis.comp30851_c0_seq2_m.31785	PREDICTED: ubiquitin carboxyl-terminal hydrolase 3-like isoform X2 [Gossypium arboreum]	29.536	6.4599	13.8	4	4	4	0.989	0.958	0.989	1.016	0.996	1.028	1.035	6.85E-02
cds.Camellia_sinensis.comp30852_c0_seq2_m.34274	PREDICTED: protein TONNEAU 1a-like [Juglans regia]	29.734	3.5112	5.6	1	1	1								
cds.Camellia_sinensis.comp30857_c0_seq1_m.28428	PREDICTED: glutamyl-tRNA reductase-binding protein, chloroplastic [Vitis vinifera]	41.468	19.28	9.4	2	2	2	1.138	1.01	1.041	0.891	0.956	0.942	0.875	3.32E-02

cds.Camellia_sinensis.comp30876_c0_seq1_m.20229	PREDICTED: protein PLASTID TRANSCRIPTIONALLY ACTIVE 14 isoform X1 [Ziziphus jujuba]	38.586	1.2437	2.1	1	1	1	1.046	0.969	1.023	0.957	0.968	1.037	0.975	4.94E-01
cds.Camellia_sinensis.comp30878_c1_seq5_m.46877	PREDICTED: probable histone H2B.1 [Juglans regia]	16.206	31.564	48	8	37	8	1.153	1.132	1.096	0.844	0.846	0.882	0.761	1.83E-04
cds.Camellia_sinensis.comp30879_c0_seq1_m.36130	PREDICTED: probable carboxylesterase 12 [Populus euphratica]	14.235	7.1361	19.5	2	3	2								
cds.Camellia_sinensis.comp30894_c0_seq1_m.34389	PREDICTED: HD domain-containing protein 2 [Jatropha curcas]	28.86	9.9453	12.9	3	4	3	1.103	1.058	1.004	0.964	0.959	0.941	0.905	2.44E-02

cds.Camellia_sinensis.comp30897_c0_seq1_m.21800	PREDICTED: phosphoinositide phospholipase C 2 [Vitis vinifera]	52.253	1.1818	2	1	1	1								
cds.Camellia_sinensis.comp30901_c0_seq13_m.23260	PREDICTED: leucine-rich repeat extensin-like protein 3 [Populus euphratica]	42.521	1.5575	2.4	1	1	1	1.1	1.021	1.041	0.955	0.924	0.946	0.893	1.00E-02
cds.Camellia_sinensis.comp30902_c0_seq1_m.794	PREDICTED: callose synthase 10 [Nicotiana attenuata]	218.2	12.501	4.2	7	8	6	0.999	1.005	1.06	0.975	0.936	1.065	0.971	5.16E-01
cds.Camellia_sinensis.comp30907_c0_seq1_m.4244	PREDICTED: NHL repeat-containing protein 2 [Ziziphus jujuba]	118.82	213.3	23.4	22	38	22	0.978	0.99	0.989	1	1.01	1	1.018	2.57E-02

cds.Camellia_sinensis.comp30914_c0_seq1_m.13785	PREDICTED: LOW QUALITY PROTEIN: 65-kDa microtubule-associated protein 1-like [Vitis vinifera]	65.614	19.226	10.4	5	5	5	1.139	1.076	1.067	0.998	0.851	0.847	0.821	2.60E-02
cds.Camellia_sinensis.comp30931_c0_seq5_m.14018	PREDICTED: protoporphyrinogen oxidase, mitochondrial isoform X1 [Vitis	62.668	34.237	14.3	7	10	7	1.003	0.953	0.973	1.013	1.025	1.049	1.054	4.33E-02
cds.Camellia_sinensis.comp30934_c0_seq1_m.39216	PREDICTED: late embryogenesis abundant protein D-29-like [Nicotiana tabacum]	27.878	46.336	23.3	6	12	6	0.961	0.896	0.888	1.058	1.099	1.129	1.197	4.50E-03
cds.Camellia_sinensis.comp30939_c0_seq1_m.5098	PREDICTED: vacuolar protein 8 [Theobroma cacao]	69.57	30.843	17	10	12	10	0.977	0.991	0.999	0.992	1.072	1.008	1.035	2.35E-01

cds.Camellia_sinensis.comp30939_c0_seq1_m.5099	PREDICTED: vacuolar protein 8 [Theobroma cacao]	23.045	32.641	16.7	3	4	3	0.996	1.004	1.053	1.032	0.965	0.958	0.968	3.26E-01
cds.Camellia_sinensis.comp30941_c0_seq1_m.40634	PREDICTED: germin-like protein subfamily 1 member 13 [Theobroma cacao]	24.269	89.566	33.9	6	24	6	1.022	0.994	0.984	0.989	1.022	1.003	1.005	7.67E-01
cds.Camellia_sinensis.comp30943_c0_seq1_m.12978	PREDICTED: conserved oligomeric Golgi complex subunit 8 isoform X1 [Vitis vinifera]	63.983	1.4449	1.6	1	1	1	1	0.993	1.025	0.961	1.064	0.965	0.991	7.80E-01
cds.Camellia_sinensis.comp30946_c0_seq1_m.27209	PREDICTED: ribokinase-like [Malus domestica]	40.011	77.722	34.7	11	18	11	0.974	1.029	1.012	1.051	0.985	1.003	1.008	7.72E-01

cds.Camellia_sinensis.comp30953_c0_seq1_m.24684	Serine/Threonine-kinase STN7, related protein, putative [Medicago truncatula]	31.109	30.704	24.1	7	11	7	0.956	0.985	1.021	1.022	1.031	1	1.031	2.21E-01
cds.Camellia_sinensis.comp30953_c0_seq1_m.24686	Protein kinase superfamily protein [Arabidopsis thaliana]	14.099	4.4485	7.8	1	4	1	1.003	1.002	0.988	0.964	1.049	1.042	1.021	5.12E-01
cds.Camellia_sinensis.comp30962_c0_seq4_m.19169	lipase class 3 family protein [Populus trichocarpa]	51.044	1.8008	5.3	3	3	3	1.014	0.942	1.023	0.961	1.041	1.028	1.017	6.61E-01
cds.Camellia_sinensis.comp30974_c0_seq1_m.26333	PREDICTED: uncharacterized oxidoreductase At1g06690, chloroplastic [Vitis vinifera]	26.481	135.54	31.1	6	12	6	1.037	1.052	1.031	0.952	0.984	0.916	0.914	1.38E-02

cds.Camellia_sinensis.comp30974_c0_seq1_m.26334	uncharacterized oxidoreductase At1g06690, chloroplastic [Ananas comosus]	18.519	51.341	40.7	7	11	7	1.021	1.019	1.044	0.991	0.966	0.956	0.945	1.24E-02
cds.Camellia_sinensis.comp30975_c1_seq2_m.19937	PREDICTED: flavin-dependent oxidoreductase FOX1-like [Nicotiana tomentosiformis]	62.388	28.149	17.7	9	11	9	1.033	1.058	1.102	0.94	0.921	0.89	0.862	3.82E-03
cds.Camellia_sinensis.comp30979_c1_seq1_m.25752	PREDICTED: GDP-mannose 3,5-epimerase 2 [Juglans regia]	42.404	114.48	42.8	16	32	16	1.051	1.039	1.041	0.939	0.952	0.949	0.907	5.90E-05
cds.Camellia_sinensis.comp30980_c0_seq2_m.28639	PREDICTED: 50S ribosomal protein L24, chloroplastic [Sesamum indicum]	19.973	7.8095	12.4	2	3	2	1.189	1.116	1.18	0.773	0.857	0.836	0.708	7.21E-04

cds.Camellia_sinensis.comp30983_c0_seq1_m.12171	PREDICTED: outer envelope protein 64, mitochondrial [Jatropha curcas]	66.511	3.3038	2.5	1	1	1								
cds.Camellia_sinensis.comp30991_c1_seq3_m.31644	PREDICTED: HMG-Y-related protein A-like [Raphanus sativus]	20.266	9.7131	34.7	6	13	1								
cds.Camellia_sinensis.comp30991_c1_seq6_m.41940	PREDICTED: HMG-Y-related protein A-like [Raphanus sativus]	19.574	49.648	32.3	6	14	1	1.251	1.197	1.148	0.771	0.811	0.758	0.651	1.80E-04
cds.Camellia_sinensis.comp30992_c0_seq1_m.38770	PREDICTED: AP-1 complex subunit sigma-1 [Sesamum indicum]	18.659	20.684	13.7	2	3	2	1.007	0.945	1.055	1.006	1.033	1.046	1.026	4.78E-01
cds.Camellia_sinensis.comp31000_c0_seq2_m.33049	PREDICTED: peptide deformylase 1B, chloroplastic [Vitis vinifera]	30.703	17.829	30.4	7	7	7	0.99	1.057	1.01	1.054	1.01	0.926	0.978	6.16E-01

cds.Camellia_sinensis.comp31006_c0_seq1_m.35933	PREDICTED: ras-related protein RABA1d [Vitis vinifera]	24.186	13.307	36.2	7	14	2	1.068	0.952	0.925	1.016	0.998	1.045	1.039	4.35E-01
cds.Camellia_sinensis.comp31013_c0_seq1_m.4933	PREDICTED: eukaryotic translation initiation factor 3 subunit C [Vitis vinifera]	107.04	35.83	10.4	10	10	10	1.015	1.042	0.993	0.979	0.974	1.038	0.981	4.69E-01
cds.Camellia_sinensis.comp31014_c0_seq1_m.2071	cytochrome f (chloroplast) [Camellia crapnelliana]	35.219	224.34	55.9	14	74	14	0.996	0.969	0.977	0.987	1.01	1.023	1.027	1.20E-01
cds.Camellia_sinensis.comp31022_c0_seq1_m.26968	peroxisomal (S)-2-hydroxy-acid oxidase GLO1 isoform X1 [Cajanus cajan]	40.676	271.35	60.7	18	107	3	0.992	0.955	0.943	1.027	1.028	1.048	1.074	1.27E-02

cds.Camellia_sinensis.comp31022_c0_seq2_m.27008	peroxisomal (S)-2-hydroxy-acid oxidase GLO1 isoform X1 [Cajanus cajan]	40.384	126.99	60.5	17	103	2	0.996	0.981	1.011	1.02	1.043	1.007	1.027	1.15E-01
cds.Camellia_sinensis.comp31027_c0_seq1_m.11356	PREDICTED: SNARE-interacting protein KEULE-like isoform X2 [Nicotiana tabacum]	42.91	1.1947	1.9	1	1	1	1.036	1.013	0.948	0.979	1.038	0.99	1.003	9.14E-01
cds.Camellia_sinensis.comp31031_c0_seq1_m.9294	PREDICTED: alpha-N-acetylglucosaminidase isoform X1 [Vitis vinifera]	92.124	39.215	12.5	9	9	9	1.024	0.952	1.009	0.974	0.998	0.991	0.993	7.81E-01
cds.Camellia_sinensis.comp31035_c0_seq1_m.19017	PREDICTED: probable lactoylglutathione lyase, chloroplastic [Nelumbo nucifera]	41.181	32.549	30.7	11	48	2	1.006	0.905	0.946	1.075	1.092	1.05	1.126	2.17E-02

cds.Camellia_sinensis.comp31035_c0_seq2_m.20044	PREDICTED: probable lactoylglutathione lyase, chloroplastic [Prunus mume]	40.353	11.947	31.3	11	45	2	1.025	0.971	0.919	0.972	1.032	1.089	1.061	2.63E-01
cds.Camellia_sinensis.comp31050_c0_seq20_m.27451	PREDICTED: probable isoaspartyl peptidase/L-asparaginase 3 isoform X2 [Vitis vinifera]	27.187	6.2571	4.8	1	1	1	1.092	1.078	1.017	0.899	0.988	0.91	0.878	2.37E-02
cds.Camellia_sinensis.comp31052_c0_seq1_m.33192	trigger factor [Arabidopsis thaliana]	23.356	7.5921	9.9	2	4	2	0.865	0.911	0.937	1.09	1.216	1.03	1.23	1.93E-02
cds.Camellia_sinensis.comp31054_c0_seq1_m.44381	PREDICTED: glutathione S-transferase L3 isoform X1 [Vitis vinifera]	18.406	10.927	22.6	5	8	5	1.12	1.064	1.083	0.907	0.894	0.899	0.826	2.60E-04
cds.Camellia_sinensis.comp31058_c0_seq1_m.3937	PREDICTED: aconitate hydratase, cytoplasmic [Citrus sinensis]	108.76	145.83	32.1	30	67	15	0.971	0.989	0.963	1.013	1.024	1.054	1.057	1.72E-02

cds.Camellia_sinensis.comp31058_c0_seq2_m.10105	PREDICTED: aconitate hydratase, cytoplasmic [Nelumbo nucifera]	73.651	146.52	36.5	21	58	14	0.978	0.999	0.946	1.015	1.034	1.039	1.056	3.39E-02
cds.Camellia_sinensis.comp31060_c0_seq1_m.3230	PREDICTED: pentatricopeptide repeat-containing protein MRL1, chloroplastic isoform X1 [Vitis vinifera]	126.72	4.6739	2.4	3	3	3	1.072	1.052	1.033	1.009	0.888	0.939	0.898	4.79E-02
cds.Camellia_sinensis.comp31061_c0_seq1_m.10833	PREDICTED: serine/threonine protein phosphatase 2A 57 kDa regulatory subunit B' theta isoform isoform X1 [Vitis vinifera]	58.796	2.6477	3.5	2	2	2	0.975	0.946	0.938	1.009	1.079	1.072	1.105	1.49E-02

cds.Camellia_sinensis.comp31062_c0_seq1_m.26787	PREDICTED: 3-dehydroquinate synthase homolog isoform X4 [Ziziphus jujuba]	39.34	12.504	14.3	5	5	5	0.971	1.007	1.007	0.984	1.01	0.954	0.988	5.72E-01
cds.Camellia_sinensis.comp31066_c0_seq1_m.29752	PREDICTED: alanine--tRNA ligase isoform X1 [Citrus sinensis]	29.293	1.5211	3.8	1	1	1								
cds.Camellia_sinensis.comp31068_c0_seq1_m.13277	DCD (development and cell death) domain protein [Medicago truncatula]	44.829	3.814	2.5	1	1	1	1.041	1.134	1.183	0.824	0.838	0.951	0.778	1.29E-02
cds.Camellia_sinensis.comp31069_c0_seq1_m.10638	PREDICTED: protein S-acyltransferase 8 [Ricinus communis]	48.481	7.3907	7.8	3	4	3	1.141	1.07	1.152	0.877	0.871	0.876	0.78	8.72E-03
cds.Camellia_sinensis.comp31074_c0_seq1_m.17913	PREDICTED: diacylglycerol kinase 7-like [Citrus sinensis]	53.405	14.843	10.4	4	4	2	0.903	1.018	0.989	0.948	1.053	1.108	1.068	3.26E-01

cds.Camellia_sinensis.comp31074_c0_seq2_m.26304	PREDICTED: diacylglycerol kinase 7-like [Citrus sinensis]	54.448	13.571	11.6	5	6	3	0.94	0.996	0.976	1.01	1.036	1.07	1.07	4.66E-02
cds.Camellia_sinensis.comp31076_c0_seq16_m.8629	Delta(24)-sterol reductase [Morus notabilis]	72.586	13.681	6.2	4	4	4	1.123	1.1	1.117	0.892	0.895	0.852	0.79	1.59E-04
cds.Camellia_sinensis.comp31084_c0_seq84_m.39802	universal stress family protein [Medicago truncatula]	25.319	17.318	12.6	3	8	3	0.965	1.014	0.921	1.048	1.016	1.03	1.067	8.84E-02
cds.Camellia_sinensis.comp31085_c0_seq15_m.15362	PREDICTED: D-3-phosphoglycerate dehydrogenase 1, chloroplastic-like [Daucus carota subsp. sativus]	63.01	123.93	19.9	11	24	8	0.966	0.976	0.992	1.018	0.999	1.028	1.038	3.13E-02
cds.Camellia_sinensis.comp31085_c0_seq3_m.50077	PREDICTED: keratin, type I cytoskeletal 9 isoform X1 [Arachis ipaensis]	21.85	3.3421	13.2	3	4	3	0.969	1.037	0.978	1.035	0.964	1.049	1.021	5.68E-01

cds.Camellia_sinensis.comp31090_c0_seq1_m.34311	catalytic/coenzyme binding protein [Arabidopsis lyrata subsp. lyrata]	30.637	14.714	6.4	2	3	2	0.938	1.047	1.023	1.031	1.009	0.97	1.001	9.71E-01
cds.Camellia_sinensis.comp31091_c0_seq2_m.31417	hydroxyproline-rich glycoprotein [Populus trichocarpa]	15.83	1.7763	7.5	1	1	1	0.924	1.084	0.997	0.967	0.89	1.147	1	9.67E-01
cds.Camellia_sinensis.comp31095_c0_seq1_m.26137	WRKY transcription factor 11-2, putative [Medicago truncatula]	45.815	9.9485	10.2	4	7	4	1.004	0.972	1.034	1.028	1.039	1.012	1.023	3.03E-01
cds.Camellia_sinensis.comp31096_c0_seq2_m.15188		14.195	-2	6.6	1	1	1	0.984	0.925	0.958	1.083	0.988	1.084	1.1	5.57E-02
cds.Camellia_sinensis.comp31098_c1_seq1_m.15559	PREDICTED: violaxanthin de-epoxidase, chloroplastic-like [Populus euphratica]	61.45	9.9885	3.7	2	2	2	0.996	1.079	1.107	0.959	0.967	0.892	0.886	4.14E-02

cds.Camellia_sinensis.comp31113_c0_seq1_m.22495	PREDICTED: ureidoglycolate hydrolase [Juglans regia]	53.52	66.838	17.6	7	19	7	0.949	0.934	0.92	1.065	1.08	1.052	1.141	3.61E-04
cds.Camellia_sinensis.comp31119_c0_seq1_m.23072	PREDICTED: UDP-glycosyltransferase 74E2-like isoform X1 [Juglans regia]	54.517	2.6303	3.7	2	2	2	1.212	1.119	1.041	0.838	0.855	0.89	0.766	5.00E-03
cds.Camellia_sinensis.comp31127_c0_seq1_m.6174	PREDICTED: chaperone protein ClpB1 [Nicotiana tabacum]	101.46	225.87	36.4	30	39	28	0.994	1.028	1.024	0.973	0.984	0.988	0.967	4.38E-02
cds.Camellia_sinensis.comp31129_c0_seq1_m.4353	PREDICTED: intracellular protein transport protein USO1 [Nicotiana attenuata]	75.838	43.291	20.8	15	17	15	0.999	1.031	1.047	0.988	0.962	0.979	0.952	3.63E-02
cds.Camellia_sinensis.comp31133_c0_seq1_m.12936	PREDICTED: pyruvate decarboxylase 1 [Gossypium arboreum]	63.495	25.612	13.4	7	10	5	0.861	0.93	0.89	1.157	1.064	1.105	1.241	2.82E-03

cds.Camellia_sinensis.comp31135_c1_seq1_m.30434	PREDICTED: methyl-CpG-binding domain-containing protein 11 isoform X1 [Vitis vinifera]	37.597	159.77	62.2	20	32	18	1.134	1.121	1.096	0.874	0.866	0.889	0.785	4.15E-05
cds.Camellia_sinensis.comp31135_c2_seq2_m.13406	PREDICTED: long chain acyl-CoA synthetase 1 [Sesamum indicum]	74.769	7.8079	7.4	5	5	5	1.103	1.245	1.211	0.907	0.831	0.668	0.676	1.53E-02
cds.Camellia_sinensis.comp31136_c0_seq1_m.35724	PREDICTED: 30S ribosomal protein S6 alpha, chloroplastic [Vitis vinifera]	28.105	9.1059	10.6	2	7	2	1.021	1.028	1.015	0.974	0.953	0.984	0.95	7.16E-03
cds.Camellia_sinensis.comp31139_c0_seq6_m.36886	PREDICTED: putative RNA-binding protein YlmH [Theobroma cacao]	17.281	2.0955	4.3	1	1	1	0.978	1.015	0.99	1.004	1.037	0.989	1.016	4.30E-01

cds.Camellia_sinensis.comp31140_c0_seq1_m.7027	PREDICTED: zinc finger protein VAR3, chloroplastic [Vitis vinifera]	100.83	106.3	17.1	12	14	12	0.98	1.041	1.017	1.023	0.963	1.013	0.987	6.40E-01
cds.Camellia_sinensis.comp31147_c0_seq4_m.40318	PREDICTED: ras-related protein RABA5b-like [Nicotiana tabacum]	25.402	7.0492	15.4	4	5	3	1.007	1.069	1.028	0.924	1.015	0.955	0.932	9.67E-02
cds.Camellia_sinensis.comp31148_c0_seq2_m.31105	PREDICTED: protein trichome birefringence-like 39 [Ziziphus jujuba]	43.425	12.607	18	6	6	6	1.058	1.061	1.011	0.888	0.98	0.973	0.908	5.09E-02
cds.Camellia_sinensis.comp31154_c0_seq1_m.28476	PREDICTED: (S)-ureidoglycine aminohydrolase [Solanum lycopersicum]	34.597	1.5063	4.5	1	1	1								

cds.Camellia_sinensis.comp31170_c0_seq2_m.13451	PREDICTED: D-2-hydroxyglutarate dehydrogenase, mitochondrial [Vitis vinifera]	65.442	13.703	10.3	7	11	7	1.014	1.014	1.069	0.983	0.986	0.959	0.945	4.69E-02
cds.Camellia_sinensis.comp31171_c0_seq1_m.26823	PREDICTED: IST1 homolog [Solanum pennellii]	36.274	44.124	27.6	8	12	8	0.96	0.977	1.004	1.063	0.971	1.047	1.048	2.11E-01
cds.Camellia_sinensis.comp31172_c0_seq1_m.3334	PREDICTED: exocyst complex component SEC8 [Vitis vinifera]	119.3	55.8	12.2	10	10	10	1.028	0.971	0.988	1.004	1.03	0.96	1.002	9.38E-01
cds.Camellia_sinensis.comp31173_c0_seq2_m.31664	PREDICTED: sanguinarine reductase isoform X1 [Citrus sinensis]	36.914	92.17	32.4	11	17	11	1.033	0.987	1.018	1.022	0.977	0.966	0.976	3.26E-01

cds.Camellia_sinensis.comp31176_c0_seq1_m.25007	PREDICTED: probable dual-specificity RNA methyltransferase RlmN isoform X1 [Daucus carota subsp. sativus]	42.547	1.5601	2.6	1	1	1								
cds.Camellia_sinensis.comp31178_c0_seq1_m.31701	PREDICTED: protein WHI4 isoform X2 [Malus domestica]	35.668	2.185	2.1	1	1	1	1.06	1	1.08	0.98	0.933	0.941	0.909	2.64E-02
cds.Camellia_sinensis.comp31183_c0_seq1_m.26990	Putative arsenical pump-driving ATPase [Morus notabilis]	45.86	22.449	16.5	6	9	6	1.054	1.092	1.023	0.969	0.904	0.93	0.885	1.13E-02

cds.Camellia_sinensis.comp31186_c0_seq1_m.26570	PREDICTED: magnesium-protoporphyrin IX monomethyl ester [oxidative] cyclase, chloroplastic isoform X2[Ziziphus jujuba]	48.864	61.529	33.6	15	25	15	1.044	1.044	1.018	0.971	0.951	0.937	0.92	3.40E-03
cds.Camellia_sinensis.comp31186_c1_seq1_m.18514	PREDICTED: UDP-glucose 6-dehydrogenase 1 [Vitis vinifera]	52.995	134.97	41	16	30	16	1.022	1.017	0.996	0.99	0.985	0.991	0.977	4.76E-02
cds.Camellia_sinensis.comp31187_c0_seq1_m.29494	PREDICTED: vesicle-associated protein 1-2 [Theobroma cacao]	35.307	21.629	14.9	5	9	4	1.03	0.993	1.02	1.071	0.976	0.968	0.991	7.81E-01
cds.Camellia_sinensis.comp31187_c0_seq2_m.31271	PREDICTED: vesicle-associated protein 1-2 [Theobroma cacao]	35.21	26.434	15.2	6	10	5	0.963	1.094	0.937	0.963	1.004	1.025	0.999	9.81E-01

cds.Camellia_sinensis.comp31188_c0_seq2_m.28517	PREDICTED: pyruvate kinase isozyme A, chloroplastic [Theobrom	41.4	21.026	21.5	7	10	7	0.956	1.014	1.011	1.006	0.999	1.039	1.021	4.04E-01
cds.Camellia_sinensis.comp31188_c1_seq1_m.39043	PREDICTED: pyruvate kinase isozyme A, chloroplastic [Vitis	35.505	35.286	3.7	1	3	1	0.98	1.029	1.028	1.033	0.986	0.956	0.98	4.93E-01
cds.Camellia_sinensis.comp31191_c0_seq3_m.14179	PREDICTED: electron transfer flavoprotein-ubiquinone oxidoreductase, mitochondrial isoform	68.67	5.0429	5.2	3	3	3	0.891	0.913	1.024	1.01	1.097	1.097	1.133	6.89E-02
cds.Camellia_sinensis.comp31194_c0_seq1_m.23449	PREDICTED: probable protein phosphatase 2C 76 [Ziziphus jujuba]	43.058	3.2678	4.9	2	2	2	0.969	1.052	1.101	0.949	0.875	1.054	0.922	2.75E-01

cds.Camellia_sinensis.comp31198_c0_seq1_m.17489	PREDICTED: UPF0051 protein ABCI8, chloroplastic [Vitis vinifera]	62.134	74.416	31	14	19	14	0.995	0.998	1.041	0.986	1.013	0.993	0.986	4.55E-01
cds.Camellia_sinensis.comp31199_c0_seq1_m.41911	short-chain dehydrogenase/reductase family protein [Populus trichocarpa]	30.046	9.8716	14.4	5	7	5	0.986	0.961	0.919	1.029	1.064	1.048	1.096	1.55E-02
cds.Camellia_sinensis.comp31205_c0_seq1_m.15992	PREDICTED: glutathione reductase, chloroplastic [Vitis vinifera]	60.57	129.02	35.5	16	36	16	0.986	0.994	0.959	1.01	1.033	1.037	1.048	2.62E-02
cds.Camellia_sinensis.comp31205_c1_seq1_m.18720	PREDICTED: pectinesterase-like [Populus euphratica]	31.464	43.178	17.8	6	14	6	0.879	0.869	0.912	1.126	1.143	1.141	1.282	8.22E-05
cds.Camellia_sinensis.comp31207_c0_seq1_m.21467	PREDICTED: actin-depolymerizing factor 2 [Ricinus	16.07	11.403	41.7	4	9	3	1.038	1.01	1.004	0.974	0.955	1.008	0.962	1.10E-01

cds.Camellia_sinensis.comp31210_c0_seq1_m.18091	PREDICTED: elongation factor 1-gamma [Vitis vinifera]	48.228	64.061	30.3	14	41	14	1.068	1.034	1.035	0.942	0.956	0.954	0.909	1.20E-03
cds.Camellia_sinensis.comp31217_c0_seq1_m.33570	PREDICTED: protein CDI-like [Nicotiana attenuata]	32.291	5.705	3.9	1	1	1								
cds.Camellia_sinensis.comp31218_c1_seq1_m.20508	PREDICTED: tryptophan--tRNA ligase, chloroplastic/mitochondrial isoform X1 [Theobroma cacao]	46.381	34.901	15.7	6	7	6	0.913	1.069	1.047	1.008	1.007	0.986	0.991	9.02E-01
cds.Camellia_sinensis.comp31222_c1_seq1_m.35525	PREDICTED: ran-binding protein 1 homolog c-like [Ipomoea nil]	24.654	58.526	29.2	5	7	4	1.088	1.07	1.083	0.873	0.903	0.922	0.832	3.83E-04

cds.Camellia_sinensis.comp31222_c2_seq1_m.42949	PREDICTED: nucleoside diphosphate kinase B-like [Nelumbo nucifera]	16.432	46.385	28.4	5	10	5	0.944	0.98	0.95	1.056	1.043	1.058	1.098	1.62E-03
cds.Camellia_sinensis.comp31222_c4_seq1_m.33714	14-3-3 protein [Vitis vinifera]	29.408	102.49	54	14	51	8	1.049	0.983	0.995	0.977	1.014	0.999	0.988	6.24E-01
cds.Camellia_sinensis.comp31226_c0_seq1_m.36088	plant/F21F14-40 protein [Medicago truncatula]	30.12	41.57	15	4	11	4	0.896	0.887	0.927	1.101	1.103	1.084	1.213	1.80E-04
cds.Camellia_sinensis.comp31226_c1_seq1_m.33340	PREDICTED: farnesyl pyrophosphate synthase 1 [Arachis duranensis]	40.387	17.961	15.4	7	13	6	0.991	0.971	1.042	1.014	0.984	0.997	0.997	9.13E-01
cds.Camellia_sinensis.comp31236_c0_seq1_m.32178	PREDICTED: xanthoxin dehydrogenase-like isoform X1 [Juglans regia]	30.617	120.71	24	6	17	6	0.978	0.95	0.979	1.058	1.059	1.016	1.078	1.13E-02

cds.Camellia_sinensis.comp31237_c0_seq1_m.31685	mitochondrial outer membrane protein porin of 36 kDa [Solanum tuberosum	29.437	102.2	46.7	11	23	8	1.02	1.019	1.048	0.985	0.95	0.958	0.937	1.05E-02
cds.Camellia_sinensis.comp31237_c0_seq2_m.40792	PREDICTED: mitochondrial outer membrane protein porin of 36 kDa [Eucalyptus grandis]	22.999	3.1935	16.4	4	10	1								
cds.Camellia_sinensis.comp31239_c0_seq1_m.8873	PREDICTED: eukaryotic translation initiation factor 3 subunit B [Vitis vinifera]	85.203	15.558	7	5	8	5	0.984	1.141	1.168	0.946	0.856	0.904	0.822	3.33E-02
cds.Camellia_sinensis.comp31241_c0_seq2_m.19977	PREDICTED: mitochondrial import inner membrane translocase subunit TIM44-2 [Theobroma cacao]	61.556	3.8747	4.3	3	4	3	1.075	1.121	1.119	0.872	0.902	0.916	0.811	4.75E-04

cds.Camellia_sinensis.comp31244_c0_seq1_m.20382	PREDICTED: biotin carboxylase 1, chloroplastic [Theobroma cacao]	58.6	56.384	32.6	16	32	16	1.005	1.029	1.034	0.963	0.96	0.984	0.948	1.01E-02
cds.Camellia_sinensis.comp31250_c0_seq1_m.34626	PREDICTED: proteasome subunit beta type-5 [Gossypium hirsutum]	29.132	41.095	19.4	6	17	6	0.956	0.957	0.928	1.067	1.02	1.046	1.103	4.04E-03
cds.Camellia_sinensis.comp31251_c0_seq1_m.15866	PREDICTED: alternative NAD(P)H-ubiquinone oxidoreductase C1, chloroplastic/mitochondrial [Vitis	21.814	19.284	15.5	2	3	2	0.946	0.915	0.965	1.103	1.091	1.01	1.134	1.70E-02
cds.Camellia_sinensis.comp31251_c0_seq2_m.17301	PREDICTED: alternative NAD(P)H-ubiquinone oxidoreductase C1, chloroplastic/mitochondrial [Vitis	24.758	13.339	16.7	3	4	3	0.94	0.911	1.003	1.125	1.072	1.087	1.151	1.13E-02

cds.Camellia_sinensis.comp31251_c0_seq3_m.45793	NADH dehydrogenase [Medicago truncatula]	12.58	35.996	37.7	3	5	3	0.921	0.981	0.958	1.061	1.109	1.021	1.116	2.23E-02
cds.Camellia_sinensis.comp31254_c0_seq1_m.27350	PREDICTED: geraniol 8-hydroxylase-like [Sesamum indicum]	49.485	16.739	23	10	14	8	0.937	0.948	0.904	1.079	1.04	1.08	1.147	1.92E-03
cds.Camellia_sinensis.comp31257_c1_seq1_m.25709	PREDICTED: malate dehydrogenase [Sesamum indicum]	36.913	98.661	34.6	11	33	5	0.964	0.965	0.954	1.047	1.067	1.046	1.096	2.44E-04
cds.Camellia_sinensis.comp31257_c1_seq2_m.37787	PREDICTED: malate dehydrogenase [Sesamum indicum]	28.109	53.868	34.6	10	27	4	1.023	0.978	0.974	0.987	1.047	1.013	1.024	3.62E-01
cds.Camellia_sinensis.comp31261_c0_seq1_m.13115	PREDICTED: signal recognition particle 54 kDa protein, chloroplastic [Solanum pennellii]	63.501	151.5	41.2	23	45	23	1.042	1.049	1.021	0.95	0.952	0.963	0.921	8.39E-04

cds.Camellia_sinensis.comp31263_c3_seq1_m.49060	PREDICTED: ubiquitin-related modifier 1 homolog 2 [Ricinus communis]	13.822	1.8869	12	1	1	1								
cds.Camellia_sinensis.comp31269_c0_seq1_m.26183	plant/T7N9-9 protein [Medicago truncatula]	41.753	37.484	23.9	8	9	8	1.011	0.978	0.977	0.968	1.033	1.019	1.018	4.78E-01
cds.Camellia_sinensis.comp31270_c0_seq20_m.16661	PREDICTED: folylpolyglutamate synthase isoform X2 [Vitis vinifera]	28.549	5.1992	6.7	2	2	2	0.987	0.988	1.025	1.007	1.003	1.002	1.004	7.72E-01
cds.Camellia_sinensis.comp31270_c0_seq20_m.16662	PREDICTED: folylpolyglutamate synthase-like isoform X1 [Capsicum annuum]	15.334	1.3912	7.5	1	2	1	0.952	1.044	1.103	1.065	0.84	1.005	0.939	4.72E-01

cds.Camellia_sinensis.comp31273_c0_seq1_m.15035	PREDICTED: 15-cis-phytoene desaturase, chloroplastic/chromoplastic isoform X1 [Vitis vinifera]	64.9	45.321	23	11	14	11	1.035	1.039	1.065	0.956	0.925	0.965	0.907	3.30E-03
cds.Camellia_sinensis.comp31275_c0_seq1_m.19312	PREDICTED: beta-galactosidase-like [Sesamum indicum]	68.686	54.92	18.7	10	21	8	0.842	0.827	0.809	1.198	1.198	1.19	1.447	3.07E-06
cds.Camellia_sinensis.comp31276_c0_seq1_m.31390	PREDICTED: 2-Cys peroxiredoxin BAS1, chloroplastic [Juglans regia]	30.692	96.646	45.7	11	43	3	0.918	0.934	0.963	1.084	1.069	1.023	1.128	5.74E-03
cds.Camellia_sinensis.comp31276_c0_seq2_m.31885	PREDICTED: 2-Cys peroxiredoxin BAS1, chloroplastic [Juglans regia]	29.991	20.276	31.4	9	40	1	1.007	0.976	1.091	0.988	0.954	0.935	0.936	1.51E-01
cds.Camellia_sinensis.comp31277_c0_seq2_m.35600	PREDICTED: mitochondrial import receptor subunit TOM20 [Vitis vinifera]	21.758	12.61	24.7	4	4	4	0.984	0.994	0.996	1.028	1.061	0.953	1.023	5.70E-01

cds.Camellia_sinensis.comp31278_c0_seq3_m.47850	PREDICTED: probable NAD(P)H dehydrogenase subunit CRR3, chloroplastic [Capsicum annuum]	20.739	7.3951	17.4	2	2	2								
cds.Camellia_sinensis.comp31279_c0_seq2_m.10195	PREDICTED: IAA-amino acid hydrolase ILR1-like isoform X2 [Ziziphus jujuba]	46.935	1.2403	3.7	2	2	1	1.018	0.947	1.026	0.972	0.969	1.073	1.008	8.73E-01
cds.Camellia_sinensis.comp31280_c0_seq1_m.12848	dihydroxy-acid dehydratase-like [Solanum lycopersicum]	65.943	41.388	23.3	13	21	13	0.943	0.96	0.949	1.064	1.065	1.035	1.109	6.40E-04
cds.Camellia_sinensis.comp31287_c0_seq12_m.42513	PREDICTED: IST1-like protein isoform X1 [Jatropha curcas]	27.878	20.126	7.7	2	2	2	0.91	1.275	1.033	0.94	0.858	0.98	0.863	2.60E-01

cds.Camellia_sinensis.comp31288_c0_seq1_m.17314	PREDICTED: polypyrimidine tract-binding protein homolog 1 isoform X1 [Vitis vinifera]	36.182	1.5279	4.3	2	2	2								
cds.Camellia_sinensis.comp31289_c0_seq1_m.5138	PREDICTED: elongation factor 2 [Juglans regia]	93.649	323.31	36.7	33	99	33	0.976	0.975	0.976	1.011	1.039	1.014	1.047	3.41E-02
cds.Camellia_sinensis.comp31295_c0_seq1_m.4827	PREDICTED: 4-coumarate--CoA ligase 2 [Theobroma cacao]	61.529	11.529	4.7	3	3	2	1.387	1.367	1.352	0.531	0.651	0.592	0.432	4.92E-03
cds.Camellia_sinensis.comp31296_c0_seq1_m.16184	PREDICTED: pyrophosphate--fructose 6-phosphate 1-phosphotransferase subunit beta-like [Daucus carota subsp. sativus]	61.663	171.18	37.8	18	38	18	1.006	1.001	0.995	0.983	0.999	1.002	0.994	4.21E-01

cds.Camellia_sinensis.comp31298_c0_seq3_m.21813	PREDICTED: SH3 domain-containing protein 2-like [Gossypium hirsutum]	41.041	1.7475	2.4	1	1	1	1.104	0.991	0.948	0.998	0.978	0.975	0.97	5.65E-01
cds.Camellia_sinensis.comp31298_c0_seq6_m.27802	PREDICTED: SH3 domain-containing protein 2-like [Ipomoea nil]	27.96	20.749	12.4	3	3	3	0.998	0.954	1.006	1.123	0.948	1.002	1.039	5.32E-01
cds.Camellia_sinensis.comp31305_c0_seq1_m.16175	PREDICTED: synaptotagmin-5-like [Ziziphus jujuba]	26.531	2.5374	4.2	1	2	1	0.933	0.946	1	1.045	1.057	1.046	1.093	1.40E-02
cds.Camellia_sinensis.comp31306_c0_seq1_m.29350	PREDICTED: ferritin isoform X1 [Solanum tuberosum	28.521	42.039	24.7	4	10	3	1.078	1.054	1.083	0.869	0.916	0.939	0.847	2.40E-03
cds.Camellia_sinensis.comp31308_c0_seq2_m.19470	PREDICTED: protein HASTY 1 isoform X4 [Nelumbo nucifera]	18.847	1.2204	12.1	2	2	2	1.046	0.964	1.067	0.964	1.006	0.954	0.95	2.24E-01

cds.Camellia_sinensis.comp31310_c0_seq1_m.36302	PREDICTED: carbonic anhydrase 2-like [Citrus sinensis]	26.853	54.121	33.1	7	14	7	0.915	0.928	0.912	1.128	1.092	1.135	1.218	1.17E-04
cds.Camellia_sinensis.comp31313_c0_seq13_m.16545	PREDICTED: alpha-1,4 glucan phosphorylase L-2 isozyme, chloroplastic/amyloplastic [Vitis vinifera]	68.013	63.367	17.4	10	17	8	1.002	1.002	0.999	0.975	0.976	0.987	0.978	5.64E-03
cds.Camellia_sinensis.comp31313_c0_seq4_m.26743	PREDICTED: alpha-1,4 glucan phosphorylase L-1 isozyme, chloroplastic/amyloplastic isoform X2 [Nicotiana tabacum]	56.271	2.0705	5.1	2	2	1								
cds.Camellia_sinensis.comp31317_c0_seq3_m.29906	PREDICTED: sorbitol dehydrogenase [Juglans regia]	39.421	127.55	52.3	13	23	12	0.927	0.972	0.953	1.046	1.044	1.03	1.094	3.50E-03

cds.Camellia_sinensis.comp31322_c0_seq2_m.13542	PREDICTED: glucose-6-phosphate 1-dehydrogenase 4, chloroplastic isoform X1 [Vitis vinifera]	79.095	9.3664	6.6	5	5	5	0.939	0.962	0.945	1.03	1.087	1.064	1.118	3.00E-03
cds.Camellia_sinensis.comp31330_c0_seq1_m.4335	Chaperone protein [Morus notabilis]	55.673	8.39	12.7	6	10	5	0.979	1.002	0.985	1.012	1.032	1.005	1.028	5.91E-02
cds.Camellia_sinensis.comp31342_c0_seq2_m.33632	PREDICTED: probable plastid-lipid-associated protein 7, chloroplastic [Vitis vinifera]	24.522	12.484	21.6	4	5	4	0.864	0.967	1.212	1.118	0.916	0.953	0.982	9.21E-01
cds.Camellia_sinensis.comp31345_c0_seq1_m.17042	PREDICTED: protein NLRC3 isoform X2 [Vitis vinifera]	66.19	39.41	10.7	6	7	6	0.981	1.005	1.05	1	0.991	1.016	0.99	6.85E-01
cds.Camellia_sinensis.comp31351_c0_seq1_m.37134	PREDICTED: ras-related protein RABH1e [Ipomoea nil]	23.131	1.6546	19.8	4	8	0								

cds.Camellia_sinensis.comp31351_c0_seq2_m.50012	PREDICTED: ras-related protein RABH1e [Ipomoea nil]	11.804	1.9129	26.6	3	3	1	1.025	1.049	1.066	0.957	0.941	0.958	0.91	1.76E-03
cds.Camellia_sinensis.comp31353_c0_seq4_m.6311	PREDICTED: structural maintenance of chromosomes protein 2-1-like [Ziziphus jujuba]	33.556	18.796	19.6	7	9	7	1.084	1.044	1.017	0.937	0.925	0.957	0.896	6.52E-03
cds.Camellia_sinensis.comp31353_c0_seq4_m.6313	DNA repair ATPase-related family protein [Populus trichocarpa]	19.049	4.5979	17.8	2	2	2	1.032	1.089	1.029	1.023	0.886	0.939	0.904	8.87E-02
cds.Camellia_sinensis.comp31354_c0_seq1_m.36920	DUF2996 family protein [Medicago truncatula]	22.698	7.1573	22.7	4	5	4	1.04	1.03	0.988	0.951	1.008	1.024	0.975	4.12E-01

cds.Camellia_sinensis.comp31357_c0_seq1_m.18229	PREDICTED: arginine biosynthesis bifunctional protein ArgJ, chloroplastic isoform X1 [Solanum tuberosum	24.199	11.721	12.4	3	4	3	0.99	0.974	0.967	1.018	1.028	1.044	1.054	6.40E-03
cds.Camellia_sinensis.comp31357_c0_seq1_m.18230	PREDICTED: arginine biosynthesis bifunctional protein ArgJ, chloroplastic [Ricinus communis]	18.399	2.6	11.1	2	3	2	1.003	1.043	1.032	0.965	0.97	0.99	0.95	2.23E-02
cds.Camellia_sinensis.comp31361_c0_seq4_m.23466	PREDICTED: pyruvate dehydrogenase E1 component subunit beta-1, mitochondrial [Nelumbo nucifera]	40.379	73.312	29.4	10	26	7	1.013	0.981	0.981	1	1.006	0.966	0.999	9.52E-01

cds.Camellia_sinensis.comp31366_c0_seq2_m.41934	PREDICTED: isocitrate dehydrogenase [NADP] isoform X2 [Sesamum indicum]	17.381	3.7374	29.2	4	10	2	1.005	1.039	1.077	0.963	1.018	0.901	0.923	1.18E-01
cds.Camellia_sinensis.comp31374_c0_seq6_m.6902	PREDICTED: clathrin interactor EPSIN 2 [Sesamum indicum]	94.053	7.0501	1.8	1	1	1								
cds.Camellia_sinensis.comp31378_c0_seq1_m.15382	PREDICTED: LIMR family protein At5g01460 [Citrus sinensis]	56.249	2.9756	2	1	2	1	0.979	1.284	1.059	1.016	0.766	0.885	0.803	1.28E-01
cds.Camellia_sinensis.comp31393_c0_seq1_m.18843	PREDICTED: uncharacterized membrane protein At1g16860-like [Solanum tuberosum	50.551	2.071	3.8	2	2	2	1.107	1.091	1.101	0.915	0.837	0.924	0.811	2.20E-02

cds.Camellia_sinensis.comp31395_c0_seq4_m.41708	PREDICTED: oxysterol-binding protein-related protein 1C-like isoform X1 [Gossypium arboreum]	29.284	2.2174	2.8	1	2	1	0.97	0.989	1.017	0.991	1.026	1.021	1.021	3.03E-01
cds.Camellia_sinensis.comp31397_c0_seq1_m.2604	PREDICTED: probable manganese-transporting ATPase PDR2 [Vitis vinifera]	134.73	32.351	8.3	10	10	10	1.025	1.043	1.108	0.899	0.942	0.953	0.88	1.24E-02
cds.Camellia_sinensis.comp31398_c0_seq4_m.4052	PREDICTED: peroxisome biogenesis protein 1 isoform X3 [Vitis vinifera]	34.006	-2	2.3	1	2	1	1.02	1.007	1.085	0.973	0.972	0.945	0.929	4.20E-02
cds.Camellia_sinensis.comp31401_c0_seq1_m.25465	isopentenyl-diphosphate delta-isomerase [Arabidopsis thaliana]	36.582	14.071	7.8	2	3	2	0.95	0.941	0.961	1.065	1.064	1.048	1.114	1.78E-04

cds.Camellia_sinensis.comp31402_c0_seq1_m.31439	PREDICTED: syntaxin-61 [Vitis vinifera]	28.189	2.1049	4	1	1	1								
cds.Camellia_sinensis.comp31409_c0_seq1_m.27497	PREDICTED: GDP-mannose 4,6 dehydratase 1 [Ipomoea nil]	41.322	5.6067	11.1	5	7	5	1.032	1.015	1.027	0.945	0.987	0.994	0.952	4.02E-02
cds.Camellia_sinensis.comp31413_c1_seq2_m.24829	PREDICTED: macrophage erythroblast attacher [Nelumbo nucifera]	27.524	1.8941	3.8	1	1	1	0.96	0.956	0.927	1.057	1.079	1.048	1.12	1.28E-03
cds.Camellia_sinensis.comp31413_c1_seq2_m.24830	PREDICTED: macrophage erythroblast attacher [Glycine max]	23.826	6.2066	15	4	5	4	0.955	0.987	0.987	1.026	0.989	1.055	1.048	9.73E-02
cds.Camellia_sinensis.comp31417_c0_seq1_m.11793	PREDICTED: acyl-CoA-binding domain-containing protein 4 isoform X1 [Vitis vinifera]	73.396	23.176	13.7	9	9	8	1.014	1.056	1.016	0.981	0.913	0.984	0.933	6.51E-02

cds.Camellia_sinensis.comp31420_c0_seq2_m.37281	PREDICTED: UMP-CMP kinase 3-like [Sesamum indicum]	23.194	6.134	12.4	3	5	2	1.037	1.039	1.016	1.021	0.889	0.996	0.94	2.14E-01
cds.Camellia_sinensis.comp31421_c0_seq2_m.20836	PREDICTED: ACT domain-containing protein ACR10 [Prunus mume]	46.488	2.2599	3.2	1	1	1								
cds.Camellia_sinensis.comp31423_c0_seq1_m.8048	PREDICTED: eukaryotic translation initiation factor 3 subunit L-like [Juglans regia]	60.856	45.063	18	8	11	8	0.966	0.974	0.973	1.034	1.066	1.073	1.089	1.88E-03
cds.Camellia_sinensis.comp31428_c0_seq1_m.16859	PREDICTED: gamma aminobutyrate transaminase 3, chloroplastic-like [Gossypium hirsutum]	57.364	112.21	29.7	11	26	11	0.966	0.963	0.987	0.993	1.035	1.032	1.049	3.57E-02

cds.Camellia_sinensis.comp31428_c1_seq1_m.4646	NADH-plastoquinone oxidoreductase chain 4L (chloroplast) [Camellia crapnelliana]	11.258	2.2505	8.9	1	2	1	0.863	0.912	0.946	1.126	1.077	1.123	1.222	2.68E-03
cds.Camellia_sinensis.comp31430_c0_seq1_m.17258	PREDICTED: glutamate--glyoxylate aminotransferase 2 [Jatropha curcas]	53.544	251.89	47	21	58	15	1.039	1.015	1.007	0.962	1.001	0.954	0.953	5.13E-02
cds.Camellia_sinensis.comp31430_c0_seq2_m.40957	PREDICTED: glutamate--glyoxylate aminotransferase 2-like [Juglans regia]	18.454	39.85	53	6	16	4	1.097	1.002	1.001	0.977	0.984	0.96	0.942	1.34E-01
cds.Camellia_sinensis.comp31435_c0_seq1_m.13980	transmembrane protein [Arabidopsis thaliana]	73.9	6.3432	5	3	3	3	1.055	1.041	0.982	0.971	0.931	1.016	0.948	1.85E-01

cds.Camellia_sinensis.comp31436_c0_seq1_m.14855	PREDICTED: germinal center kinase 1 isoform X3 [Vitis vinifera]	72.963	1.2564	1.8	1	1	1								
cds.Camellia_sinensis.comp31439_c0_seq1_m.17754	PREDICTED: uncharacterized aarF domain-containing protein kinase At5g05200, chloroplastic [Vitis vinifera]	59.251	71.329	25.7	12	19	12	1.008	1.021	1.012	0.998	0.983	1.004	0.982	6.43E-02
cds.Camellia_sinensis.comp31443_c0_seq1_m.4815	PREDICTED: phototropin-2 [Vitis vinifera]	31.198	7.1811	13.1	3	3	2								
cds.Camellia_sinensis.comp31443_c0_seq2_m.5583	PREDICTED: phototropin-2 [Vitis vinifera]	31.112	7.2338	8.2	2	2	1								
cds.Camellia_sinensis.comp31443_c0_seq2_m.5584	PREDICTED: phototropin-2-like [Malus domestica]	22.588	31.686	28.9	5	5	4	1.233	1.126	1.186	0.789	0.788	0.877	0.692	1.12E-03

cds.Camellia_sinensis.comp31443_c0_seq2_m.5585	PREDICTED: phototropin-2 [Nelumbo nucifera]	11.501	1.6787	14.6	1	1	1									
cds.Camellia_sinensis.comp31446_c1_seq1_m.31776	PREDICTED: COP9 signalosome complex subunit 7-like isoform X2 [Nicotiana	28.105	2.1067	7.7	2	2	2									
cds.Camellia_sinensis.comp31451_c0_seq1_m.18500	PREDICTED: cytochrome P450 86B1-like [Sesamum indicum]	57.848	5.5293	6.8	3	3	3	1.404	1.26	1.295	0.609	0.584	0.735	0.487	7.42E-04
cds.Camellia_sinensis.comp31454_c0_seq1_m.4100	PREDICTED: vam6/Vps39-like protein [Vitis vinifera]	112.57	4.5719	2.1	2	2	2	1.039	1.009	0.96	0.918	1.023	1.05	0.994	8.92E-01
cds.Camellia_sinensis.comp31457_c0_seq1_m.2138	ATPase family AAA domain-containing protein 1 [Morus notabilis]	137.34	12.061	2.6	2	2	1	1.045	1.157	1.127	0.934	0.79	0.926	0.796	2.11E-02

cds.Camellia_sinensis.comp31459_c0_seq2_m.9376	PREDICTED: acyltransferase-like protein At1g54570, chloroplastic isoform X1 [Vitis vinifera]	63.915	5.6143	5.1	3	3	3	1.04	0.937	1.084	1.027	1.017	0.903	0.963	5.63E-01
cds.Camellia_sinensis.comp31464_c0_seq1_m.19989	PREDICTED: leucine-rich repeat extensin-like protein 6 [Daucus carota subsp. sativus]	50.99	50.07	21.6	10	21	10	1.003	1.008	0.977	1.014	0.981	1.011	1.006	6.98E-01
cds.Camellia_sinensis.comp31465_c0_seq3_m.12731	PREDICTED: fasciclin-like arabinogalactan protein 1 [Sesamum indicum]	43.379	8.6034	7.6	2	3	2	1.057	1.047	0.934	0.88	1.178	0.902	0.974	7.58E-01
cds.Camellia_sinensis.comp31465_c0_seq5_m.13544	PREDICTED: fasciclin-like arabinogalactan protein 2 [Theobroma cacao]	20.53	1.8199	4.6	1	1	1								

cds.Camellia_sinensis.comp31474_c0_seq3_m.1857	PREDICTED: stromal processing peptidase, chloroplastic [Vitis vinifera]	143.42	164.03	19	21	30	21	0.993	1.008	1.033	0.98	0.98	0.987	0.971	6.93E-02
cds.Camellia_sinensis.comp31475_c0_seq1_m.12049	PREDICTED: glucan endo-1,3-beta-glucosidase 6 [Juglans	40.907	3.3748	9	3	3	2								
cds.Camellia_sinensis.comp31476_c0_seq1_m.29022	PREDICTED: COP9 signalosome complex subunit 8 [Vitis	21.739	14.877	10.6	2	2	2	0.948	0.976	1.114	0.943	1.053	0.979	0.979	7.59E-01
cds.Camellia_sinensis.comp31481_c1_seq1_m.17674	PREDICTED: glucose-6-phosphate/phosphate translocator 1, chloroplastic isoform X2 [Populus euphratica]	43.511	7.5853	4.8	2	2	2	1.056	1.015	1.02	0.946	0.982	0.976	0.94	2.14E-02

cds.Camellia_sinensis.comp31483_c0_seq1_m.35630	PREDICTED: 17.8 kDa class II heat shock protein [Theobroma cacao]	30.257	43.625	19.8	4	9	4	1.002	0.953	1.007	1.053	1.028	0.954	1.025	5.29E-01
cds.Camellia_sinensis.comp31489_c0_seq1_m.25731	PREDICTED: probable indole-3-pyruvate monooxygenase YUCCA10 isoform X1 [Nelumbo nucifera]	42.664	29.008	31.4	10	15	10	0.989	1.075	1.016	0.965	0.962	0.928	0.927	5.22E-02
cds.Camellia_sinensis.comp31491_c0_seq1_m.14592	PREDICTED: vacuolar protein-sorting-associated protein 33 homolog isoform X2 [Solanum tuberosum	67.853	7.7812	4.2	3	3	3	0.949	0.927	0.974	1.001	1.038	1.016	1.072	1.73E-02

cds.Camellia_sinensis.comp31494_c0_seq1_m.11585	PREDICTED: phosphatidylserine decarboxylase proenzyme 3-like [Malus domestica]	73.368	38.291	22	14	19	11	1.014	1.032	1.039	0.968	0.978	0.981	0.949	3.20E-03
cds.Camellia_sinensis.comp31494_c0_seq2_m.28102	PREDICTED: phosphatidylserine decarboxylase proenzyme 2 [Vitis vinifera]	42.343	4.6582	16.1	7	8	4	1.131	1.097	1.212	0.871	0.84	0.815	0.734	9.38E-04
cds.Camellia_sinensis.comp31503_c0_seq1_m.23563	SOUL heme-binding family protein [Medicago truncatula]	44.862	6.5765	8.4	4	6	4	1.042	1.039	0.987	0.944	0.98	0.985	0.948	7.45E-02
cds.Camellia_sinensis.comp31504_c0_seq1_m.40828	PREDICTED: glutathione S-transferase zeta class-like [Fragaria vesca subsp. vesca]	26.492	9.6859	27.5	5	7	5	0.973	0.965	0.967	1.062	1.034	1.045	1.081	6.63E-04

cds.Camellia_sinensis.comp31513_c0_seq2_m.27050	PREDICTED: beta-ureidopropionase [Ipomoea nil]	48.626	11.128	9.9	3	4	3	1.018	1.153	0.937	1.132	0.891	0.875	0.932	5.14E-01
cds.Camellia_sinensis.comp31528_c0_seq1_m.9279	PREDICTED: probable methionine--tRNA ligase [Daucus carota subsp. sativus]	90.978	147.32	36.3	25	35	23	1.045	1.031	1.051	0.942	0.982	0.95	0.919	3.70E-03
cds.Camellia_sinensis.comp31534_c0_seq1_m.24580	PREDICTED: UDP-glycosyltransferase 73C2 [Vitis vinifera]	55.675	39.703	16.2	7	12	7	0.979	0.955	0.958	1.056	1.107	0.975	1.085	1.01E-01
cds.Camellia_sinensis.comp31534_c0_seq2_m.50624	PREDICTED: UDP-glycosyltransferase 73C2 [Vitis vinifera]	21.076	13.456	10.9	1	1	1	0.813	0.988	1.096	1.098	0.989	1.058	1.086	3.93E-01
cds.Camellia_sinensis.comp31546_c0_seq2_m.19660	GYF domain-containing family protein [Populus trichocarpa]	23.868	9.4244	10.1	1	1	1	1.225	1.025	1.072	0.856	0.832	0.947	0.793	2.56E-02

cds.Camellia_sinensis.comp31546_c0_seq2_m.19661	GYF domain-containing protein [Arabidopsis thaliana]	21.29	1.659	4.7	1	1	1								
cds.Camellia_sinensis.comp31549_c0_seq5_m.7432	PREDICTED: dipeptidyl aminopeptidase 4 [Malus domestica]	87.835	10.998	3.7	3	3	3	0.921	0.972	1.032	0.983	1.053	1.059	1.058	2.34E-01
cds.Camellia_sinensis.comp31550_c0_seq1_m.16059	PREDICTED: probably inactive leucine-rich repeat receptor-like protein kinase IMK2 [Citrus sinensis]	65.384	2.0964	2	1	1	1								
cds.Camellia_sinensis.comp31551_c0_seq7_m.41104	PREDICTED: tobamovirus multiplication protein 2A-like [Gossypium hirsutum]	17.528	4.4109	8.4	1	1	1	1.077	1.024	1.104	0.891	0.753	1.127	0.865	2.53E-01

cds.Camellia_sinensis.comp31558_c0_seq1_m.19170	PREDICTED: squalene synthase [Vitis vinifera]	47.269	5.8641	6	2	2	2	1.199	0.979	0.679	1.216	1.272	0.674	1.107	7.51E-01
cds.Camellia_sinensis.comp31559_c0_seq3_m.32145	PREDICTED: vesicle-associated membrane protein 722-like [Vigna radiata var. radiata]	24.815	-2	10.9	4	11	1	1.011	1.104	1.103	0.939	0.901	0.934	0.862	9.82E-03
cds.Camellia_sinensis.comp31559_c0_seq5_m.33305	Putative vesicle-associated membrane protein 726 [Morus notabilis]	24.779	59.748	29.5	8	19	5	0.972	0.961	1.008	1.051	1.015	1.001	1.043	1.09E-01
cds.Camellia_sinensis.comp31560_c0_seq1_m.23697	PREDICTED: PGR5-like protein 1A, chloroplastic [Ziziphus jujuba]	35.181	28.184	19.4	7	10	7	1.04	1.004	1.015	0.992	0.978	0.976	0.963	3.18E-02

cds.Camellia_sinensis.comp31572_c0_seq2_m.39642	PREDICTED: protein LURP-one-related 12-like [Nicotiana tabacum]	23.361	3.1983	9	2	2	2	0.993	0.962	1.039	1.004	1.009	1.006	1.008	7.32E-01
cds.Camellia_sinensis.comp31577_c0_seq1_m.18209	iron-sulfur cluster biosynthesis family protein [Medicago truncatula]	59.811	108.51	16.2	8	13	8	0.98	0.981	1.035	0.99	1.02	0.991	1.002	9.31E-01
cds.Camellia_sinensis.comp31578_c0_seq2_m.37745	SNF7 family protein [Populus trichocarpa]	27.134	10.817	15.2	4	4	4	1.216	1.204	1.226	0.72	0.775	0.776	0.623	4.39E-05
cds.Camellia_sinensis.comp31580_c0_seq1_m.19204	BSD domain-containing family protein [Populus trichocarpa]	53.407	2.1591	2.1	1	1	1	1.177	1.197	1.132	0.935	0.786	0.732	0.7	8.28E-03
cds.Camellia_sinensis.comp31581_c0_seq1_m.13417	Dynamin-related protein 1E [Morus notabilis]	68.627	15.202	27.3	15	19	4	1.056	1.049	1.081	0.876	0.931	0.933	0.86	2.60E-03
cds.Camellia_sinensis.comp31581_c0_seq2_m.13831	Dynamin-related protein 1E [Morus notabilis]	69.077	83.96	30.4	16	21	4	0.916	0.945	0.933	1.093	1.074	1.104	1.171	2.00E-04

cds.Camellia_sinensis.comp31585_c0_seq1_m.34323	PREDICTED: serine/threonine-protein phosphatase 4 regulatory subunit 2 isoform X1 [Ziziphus jujuba]	27.935	5.7844	13.5	4	5	4	0.935	1.07	1.075	1.019	0.945	0.969	0.952	4.05E-01
cds.Camellia_sinensis.comp31586_c1_seq5_m.40052	PREDICTED: probable prefoldin subunit 2 [Juglans regia]	16.257	21.523	54.4	9	11	9	0.962	0.971	0.954	1.056	1.026	1.051	1.085	1.36E-03
cds.Camellia_sinensis.comp31587_c0_seq1_m.32497	PREDICTED: HD domain-containing protein 2 [Vitis vinifera]	30.043	1.5811	7.1	2	2	2	0.997	1.031	1.09	0.98	0.957	0.948	0.925	5.03E-02
cds.Camellia_sinensis.comp31592_c0_seq1_m.13329	propionyl-CoA carboxylase, putative [Medicago truncatula]	74.98	2.3327	1.6	1	1	1								
cds.Camellia_sinensis.comp31599_c0_seq1_m.8884	40S ribosomal protein S14-3 [Morus notabilis]	16.325	78.275	46	6	12	6	1.159	1.168	1.142	0.814	0.835	0.827	0.714	2.25E-06

cds.Camellia_sinensis.comp31609_c0_seq1_m.1993	PREDICTED: magnesium-chelatase subunit ChlH, chloroplastic [Gossypium m	156.47	42.581	7.5	9	9	9	0.978	1.019	1.104	0.953	0.984	1.009	0.95	2.69E-01
cds.Camellia_sinensis.comp31610_c0_seq1_m.25962	PREDICTED: insulin-degrading enzyme-like 1, peroxisomal [Theobroma cacao]	46.134	12.081	8.2	3	4	3								
cds.Camellia_sinensis.comp31614_c1_seq1_m.20028	PREDICTED: vacuolar-processing enzyme-like [Jatropha curcas]	44.625	3.3268	4	2	4	2	1.088	1.068	1.084	0.886	0.934	0.932	0.849	8.40E-04
cds.Camellia_sinensis.comp31617_c0_seq1_m.20299	PREDICTED: cytochrome P450 98A2 [Citrus sinensis]	59.852	9.0653	5.9	4	4	4	1.173	1.061	1.04	0.875	0.884	0.883	0.807	2.94E-02

cds.Camellia_sinensis.comp31628_c0_seq1_m.23367	PREDICTED: ubiquitin-activating enzyme E1 1-like [Ziziphus jujuba]	47.768	13.454	14.7	8	15	3	0.979	0.953	0.989	1.028	1.022	0.977	1.036	1.43E-01
cds.Camellia_sinensis.comp31628_c1_seq1_m.44607	PREDICTED: ubiquitin-activating enzyme E1 1-like [Ziziphus jujuba]	28.047	4.959	8.7	2	2	2								
cds.Camellia_sinensis.comp31629_c2_seq1_m.24972	PREDICTED: ABC transporter G family member 11 [Vitis vinifera]	47.276	9.734	2.4	1	1	1	1.233	1.228	1.006	0.775	0.805	0.893	0.713	1.34E-02
cds.Camellia_sinensis.comp31633_c0_seq1_m.23576	PREDICTED: crocetin glucosyltransferase, chloroplastic-like [Nicotiana sylvestris]	51.15	35.256	10	4	5	2	0.953	0.953	0.981	1.011	1.053	1.071	1.086	1.39E-02

cds.Camellia_sinensis.comp31633_c0_seq2_m.35544	PREDICTED: crocetin glucosyltransferase, chloroplastic-like, partial [Sesamum indicum]	35.64	15.347	10.8	3	3	1								
cds.Camellia_sinensis.comp31634_c0_seq3_m.15811	PREDICTED: protein MON2 homolog isoform X2 [Juglans regia]	24.093	1.4044	4.7	1	1	1								
cds.Camellia_sinensis.comp31636_c0_seq2_m.39904	PREDICTED: ADP-ribosylation factor 1 [Prunus mume]	20.231	15.529	14.8	2	4	2	1.059	1.031	1.004	1.073	0.97	0.999	0.983	6.29E-01
cds.Camellia_sinensis.comp31638_c0_seq1_m.17824	PREDICTED: protein TRIGALACTOSYLDIACYLGLYCEROL 3, chloroplastic isoform X1 [Sesamum indicum]	37.986	2.9666	5.1	2	3	2	1.023	1.055	1.013	0.916	0.989	1.001	0.94	1.09E-01

cds.Camellia_sinensis.comp31639_c0_seq2_m.22265	PREDICTED: tubulin alpha-3 chain [Gossypium raimondii]	49.711	18.069	24.7	10	19	0								
cds.Camellia_sinensis.comp31642_c0_seq2_m.42606	PREDICTED: probable mediator of RNA polymerase II transcription subunit 37c, partial [Eucalyptus grandis]	22.778	7.1212	15.1	3	5	1	0.995	1.033	1.054	0.869	1.08	0.97	0.947	4.23E-01
cds.Camellia_sinensis.comp31646_c0_seq1_m.28973	alpha/beta-Hydrolases superfamily protein [Arabidopsis thaliana]	36.259	2.725	4.8	2	2	2	1.048	1.074	1.042	0.816	0.997	1.008	0.892	2.27E-01
cds.Camellia_sinensis.comp31652_c0_seq1_m.6916	PREDICTED: protein EXORDIUM-like 2 [Theobroma cacao]	36.343	21.274	14	4	6	4	1.017	1.015	1.047	0.973	0.951	0.964	0.938	6.02E-03

cds.Camellia_sinensis.comp31652_c0_seq1_m.6917	PREDICTED: fumarate hydratase 1, mitochondrial isoform X2 [Gossypium	29.957	99.173	43.3	10	20	10	1.016	1.063	1.049	0.916	0.958	0.973	0.91	1.39E-02
cds.Camellia_sinensis.comp31652_c0_seq1_m.6918	Fumarate hydratase 1 family protein [Populus trichocarpa]	18.7	11.413	16.5	3	3	3	1.045	1.051	1.043	0.934	0.95	0.953	0.904	1.05E-04
cds.Camellia_sinensis.comp31653_c0_seq1_m.14478	PREDICTED: CLP protease regulatory subunit CLPX1, mitochondrial-like [Solanum pennellii]	68.927	4.6876	4.7	3	4	1								
cds.Camellia_sinensis.comp31654_c0_seq2_m.30801	PREDICTED: fatty-acid-binding protein 3, chloroplastic [Vitis vinifera]	30.251	18.353	9.8	2	4	2	0.996	1.063	1.06	0.958	0.945	0.969	0.921	2.16E-02

cds.Camellia_sinensis.comp31662_c0_seq1_m.30500	PREDICTED: putative glucose-6-phosphate 1-epimerase [Nelumbo nucifera]	35.501	10.983	29.6	7	13	2	1.013	0.912	1.038	0.918	1.066	1.06	1.027	6.99E-01
cds.Camellia_sinensis.comp31676_c0_seq1_m.23298	PREDICTED: OTU domain-containing protein 6B [Cucumis melo]	27.75	1.7361	4.8	1	1	1								
cds.Camellia_sinensis.comp31678_c1_seq1_m.16965	zinc ion-binding protein, putative [Medicago truncatula]	40.757	5.25	8.9	4	4	4	0.936	0.951	0.945	1.04	1.091	1.055	1.125	1.40E-03
cds.Camellia_sinensis.comp31687_c0_seq2_m.32126	PREDICTED: proteasome subunit beta type-7-A [Sesamum indicum]	29.52	11.415	9.2	3	10	3	1.02	1.003	1.023	1.01	0.983	0.976	0.975	1.01E-01
cds.Camellia_sinensis.comp31690_c0_seq1_m.13753	PREDICTED: plant UBX domain-containing protein 11 isoform X1 [Vitis vinifera]	62.219	4.5695	6.2	4	4	4	0.986	0.992	1.043	0.955	0.949	1.028	0.971	3.88E-01

cds.Camellia_sinensis.comp31693_c0_seq1_m.36890	PREDICTED: vesicle-associated membrane protein 714 [Prunus mume]	26.479	2.1048	3.9	1	1	1	0.992	0.992	0.959	1.058	1.034	0.983	1.045	1.50E-01
cds.Camellia_sinensis.comp31694_c0_seq1_m.25248	PREDICTED: 3-oxo-Delta(4,5)-steroid 5-beta-reductase-like [Nicotiana attenuata]	30.526	9.7601	21.9	6	9	6	0.933	1.028	1.023	0.994	1.011	0.984	1.002	9.40E-01
cds.Camellia_sinensis.comp31694_c0_seq6_m.38692	PREDICTED: universal stress protein A-like protein [Ziziphus jujuba]	18.521	7.9883	16	4	6	4	1.011	1.011	0.968	0.979	0.995	1.027	1.004	8.64E-01
cds.Camellia_sinensis.comp31696_c0_seq1_m.8121	aleurone layer morphogenesis protein [Medicago truncatula]	85.151	5.347	4.5	4	4	4	1.041	1.02	0.991	0.958	0.969	1.02	0.966	2.17E-01

cds.Camellia_sinensis.comp31697_c0_seq1_m.20650	PREDICTED: NADH dehydrogenase [ubiquinone] 1 alpha subcomplex subunit 9, mitochondrial [Ziziphus jujuba]	38.682	67.922	32.1	10	14	10	1.011	1.023	0.975	1.006	0.982	1.017	0.999	9.48E-01
cds.Camellia_sinensis.comp31714_c0_seq2_m.17445	PREDICTED: renalase isoform X1 [Citrus sinensis]	27.415	7.0843	12	2	2	2								
cds.Camellia_sinensis.comp31715_c0_seq1_m.21131	PREDICTED: 2-methylbutanal oxime monooxygenase [Theobroma cacao]	58.791	22.417	12.4	7	9	7	0.901	0.932	0.946	1.118	1.094	1.082	1.185	6.02E-04
cds.Camellia_sinensis.comp31721_c0_seq1_m.24824	PREDICTED: mitogen-activated protein kinase homolog MMK1 [Vigna angularis]	45.454	11.166	15.8	6	7	5	0.977	0.971	1.026	1.006	1.024	1.056	1.038	1.74E-01

cds.Camellia_sinensis.comp31721_c0_seq2_m.41791	PREDICTED: mitogen-activated protein kinase homolog MMK2 isoform X2 [Glycine max]	20.964	1.6928	9.8	2	3	1	0.989	1.038	0.905	1.015	1.021	1.044	1.05	2.84E-01
cds.Camellia_sinensis.comp31721_c0_seq31_m.44147	PREDICTED: mitogen-activated protein kinase homolog MMK2 [Pyrus x bretschneideri]	29.242	11.143	14.3	4	6	3	0.976	1.093	1.055	0.986	0.979	0.917	0.923	1.20E-01
cds.Camellia_sinensis.comp31721_c1_seq1_m.40504	PREDICTED: mitogen-activated protein kinase homolog MMK2 [Vitis vinifera]	22.169	86.288	28.1	4	6	3	1.037	0.962	0.93	1.104	0.964	1.007	1.05	4.02E-01
cds.Camellia_sinensis.comp31723_c0_seq1_m.25436	PREDICTED: trithorax group protein osa [Ziziphus jujuba]	54.091	127.87	33.2	12	24	11	1.007	1.02	0.958	0.998	1.003	0.994	1.003	8.66E-01

cds.Camellia_sinensis.comp31731_c0_seq1_m.2028	PREDICTED: xanthine dehydrogenase 1 [Vitis vinifera]	91.284	3.3731	2.3	2	3	2	0.989	0.931	1.086	1.014	0.971	1.022	1	9.67E-01
cds.Camellia_sinensis.comp31764_c0_seq2_m.44977	PREDICTED: NADH dehydrogenase [ubiquinone] iron-sulfur protein 6, mitochondrial [Ipomoea nil]	14.225	2.4462	17.7	2	3	2	1.091	0.917	1.018	0.871	0.808	1.277	0.977	7.95E-01
cds.Camellia_sinensis.comp31766_c0_seq1_m.39689	PREDICTED: dihydrolipoyllysine-residue succinyltransferase component of 2-oxoglutarate dehydrogenase complex 2, mitochondrial-like [Juglans regia]	30.695	2.9499	3.5	1	3	1	1.095	0.941	1.002	0.947	0.974	1.034	0.973	6.32E-01

cds.Camellia_sinensis.comp31767_c1_seq1_m.31627	PREDICTED: 60S ribosomal protein L14-2-like [Nelumbo nucifera]	15.416	10.763	35.8	5	14	2	1.11	1.113	1.124	0.877	0.831	0.87	0.77	1.05E-04
cds.Camellia_sinensis.comp31769_c0_seq1_m.36107	PREDICTED: protein N-terminal glutamine amidohydrolase [Citrus sinensis]	25.432	5.083	6.2	1	1	1	0.93	0.916	1.072	1.067	0.995	1.047	1.065	2.94E-01
cds.Camellia_sinensis.comp31776_c0_seq1_m.23439	PREDICTED: dolichyl-phosphate beta-glucosyltransferase [Ricinus communis]	20.68	5.9004	14.3	2	2	2	0.986	0.956	0.995	1.052	1.019	1.01	1.049	5.06E-02
cds.Camellia_sinensis.comp31779_c0_seq1_m.1001	PREDICTED: proteasome-associated protein ECM29 homolog [Vitis vinifera]	187.43	7.8071	2.5	4	4	4	0.972	1.023	0.996	1.007	1.026	0.99	1.011	5.83E-01

cds.Camellia_sinensis.comp31781_c0_seq1_m.40000	PREDICTED: 39S ribosomal protein L47, mitochondrial [Cucumis sativus]	16.537	7.9898	28.7	3	3	3	0.927	1.03	0.905	1.07	0.989	1.103	1.105	1.23E-01
cds.Camellia_sinensis.comp31787_c0_seq1_m.11219	PREDICTED: YTH domain-containing family protein 1-like [Sesamum indicum]	79.591	31.73	13.4	11	15	11	0.928	0.975	1	1.028	1.071	1.04	1.081	3.53E-02
cds.Camellia_sinensis.comp31788_c0_seq1_m.5572	PREDICTED: probable receptor-like protein kinase At5g47070 isoform X2 [Nelumbo nucifera]	46.778	22.622	8.6	4	5	3	1.011	1.069	1.01	0.982	0.962	0.975	0.945	4.53E-02
cds.Camellia_sinensis.comp31789_c0_seq1_m.20372	PREDICTED: cytochrome P450 76C2-like [Vitis vinifera]	63.468	14.003	13.3	7	9	6	0.999	1.05	1.025	0.975	0.969	0.986	0.953	3.50E-02

cds.Camellia_sinensis.comp31792_c2_seq1_m.12491	PREDICTED: uncharacterized membrane protein At3g27390 [Sesamum	28.132	4.0855	4	1	1	1	1.181	1.031	1.169	0.807	0.868	0.903	0.762	7.80E-03
cds.Camellia_sinensis.comp31806_c0_seq1_m.4333	PREDICTED: protein argonaute 4-like isoform X2 [Juglans regia]	102.18	81.51	23.8	20	28	11	1.142	1.186	1.174	0.82	0.825	0.795	0.697	2.17E-05
cds.Camellia_sinensis.comp31809_c0_seq1_m.5522	PREDICTED: pyruvate, phosphate dikinase, chloroplastic [Malus domestica]	107	165.23	25.8	26	55	26	0.974	1.006	0.998	1.019	1	0.987	1.009	5.24E-01
cds.Camellia_sinensis.comp31810_c0_seq2_m.6849	PREDICTED: probable linoleate 9S-lipoxygenase 5 [Juglans regia]	86.42	141.24	37.1	30	59	27	0.991	0.966	0.985	1.018	1.019	1.024	1.04	7.42E-03

cds.Camellia_sinensis.comp31810_c0_seq2_m.6850	PREDICTED: probable linoleate 9S-lipoxygenase 5 [Eucalyptus grandis]	21.445	48.311	30.2	6	11	6	0.992	1.009	0.953	1.049	1.03	1.003	1.043	1.17E-01
cds.Camellia_sinensis.comp31816_c1_seq3_m.30019	PREDICTED: acyl-CoA-binding domain-containing protein 1 [Vitis vinifera]	40.747	3.3527	3.2	1	1	1								
cds.Camellia_sinensis.comp31817_c0_seq1_m.7018	PREDICTED: polyadenylate-binding protein 8-like isoform X2 [Nicotiana	44.482	2.2016	18.1	8	13	1	1	1.024	0.914	1.019	1.022	1.034	1.047	3.12E-01
cds.Camellia_sinensis.comp31817_c0_seq2_m.7904	PREDICTED: polyadenylate-binding protein 8-like isoform X2 [Nicotiana	44.471	57.432	23.6	9	15	2	0.926	0.951	0.998	1.05	0.977	0.973	1.043	2.69E-01

cds.Camellia_sinensis.comp31817_c0_seq2_m.7905	polyadenylate-binding family protein [Populus trichocarpa	29.29	66.505	41.5	10	12	10	0.976	0.982	1	1.03	0.991	1.019	1.028	1.16E-01
cds.Camellia_sinensis.comp31819_c0_seq1_m.19399	PREDICTED: sphingosine-1-phosphate lyase [Jatropha curcas]	62.296	7.6452	8.9	5	5	5	0.961	1.051	1.025	1.055	0.95	1.056	1.008	8.76E-01
cds.Camellia_sinensis.comp31821_c0_seq13_m.22182	PREDICTED: protein indeterminate-domain 5, chloroplastic isoform X1 [Theobroma cacao]	20.883	1.7351	4.3	1	1	1								
cds.Camellia_sinensis.comp31826_c0_seq1_m.17816	PREDICTED: nicotinate phosphoribosyltransferase 2 [Daucus carota subsp. sativus]	62.797	7.4605	6.4	3	4	3	1.008	1.019	1.024	0.964	1.002	0.982	0.966	4.65E-02

cds.Camellia_sinensis.comp31827_c0_seq1_m.11455	calcium ion-binding protein [Medicago truncatula]	64.054	1.5508	1.6	1	1	1	0.992	0.961	1.005	1.115	0.969	0.979	1.035	5.22E-01
cds.Camellia_sinensis.comp31830_c0_seq1_m.18863	PREDICTED: serine carboxypeptidase-like [Eucalyptus grandis]	25.392	34.376	34.1	6	17	6	1.069	1.043	0.985	0.949	0.986	0.975	0.94	8.21E-02
cds.Camellia_sinensis.comp31831_c0_seq2_m.20143	PREDICTED: plasminogen activator inhibitor 1 RNA-binding protein [Vitis vinifera]	40.744	35.755	13.6	4	5	3	1.107	1.144	1.206	0.882	0.789	0.839	0.726	1.42E-03
cds.Camellia_sinensis.comp31831_c0_seq3_m.20572	PREDICTED: U3 small nucleolar RNA-associated protein 25 [Jatropha curcas]	40.236	48.52	19.8	6	13	4	1.308	1.202	1.239	0.708	0.658	0.781	0.573	5.42E-04

cds.Camellia_sinensis.comp31831_c0_seq7_m.23621	PREDICTED: plasminogen activator inhibitor 1 RNA-binding protein [Vitis vinifera]	38.619	114.14	26	7	15	6	1.16	1.134	1.089	0.834	0.844	0.838	0.744	9.76E-05
cds.Camellia_sinensis.comp31838_c0_seq18_m.38193	PREDICTED: probable glutathione peroxidase 2 [Citrus sinensis]	22.177	11.021	34.7	8	15	8	0.942	0.961	0.922	1.092	1.091	1.02	1.134	8.04E-03
cds.Camellia_sinensis.comp31839_c1_seq12_m.43599	PREDICTED: 40S ribosomal protein S26-1 [Musa acuminata subsp. malaccensis]	17.653	21.35	33.5	5	9	1	1.042	1.055	1.118	0.901	0.977	0.897	0.863	1.39E-02
cds.Camellia_sinensis.comp31839_c1_seq13_m.43609	PREDICTED: 40S ribosomal protein S26-3 [Vitis vinifera]	17.755	3.7895	33.3	5	10	1	1.119	1.083	1.08	0.801	0.958	0.929	0.819	2.30E-02

cds.Camellia_sinensis.comp31839_c1_seq9_m.41622	PREDICTED: mavicyanin [Theobroma cacao]	24.512	15.908	10.1	2	3	2	1.019	1.029	1.017	0.986	1.005	0.97	0.966	3.34E-02
cds.Camellia_sinensis.comp31852_c0_seq1_m.12704	PREDICTED: probable pre-mRNA-splicing factor ATP-dependent RNA helicase DEAH9 isoform X1 [Theobroma cacao]	77.817	2.6793	1.7	1	1	1	0.879	1.08	0.907	1.091	1.075	1.003	1.106	2.07E-01
cds.Camellia_sinensis.comp31855_c0_seq25_c0_seq2_m.30542	PREDICTED: putative hydrolase C777.06c [Citrus sinensis]	36.191	6.6618	4	1	1	1	1.089	0.959	1.129	0.927	0.979	0.906	0.885	9.25E-02

cds.Camellia_sinensis.comp31857_c0_seq5_m.17877	PREDICTED: putative E3 ubiquitin-protein ligase XBAT35 isoform X2 [Nicotiana sylvestris]	58.081	6.101	3	1	1	1	1.047	1.043	0.986	0.787	1.044	1.078	0.946	5.57E-01
cds.Camellia_sinensis.comp31860_c0_seq10_c0_seq1_m.5968	PREDICTED: ubiquitin-conjugating enzyme E2 36 [Theobroma cacao]	22.288	59.801	34.8	7	21	7	0.998	1.024	0.979	0.998	1.018	1.008	1.008	6.14E-01
cds.Camellia_sinensis.comp31866_c0_seq1_m.8702	PREDICTED: pentatricopeptide repeat-containing protein At1g74850, chloroplastic [Vitis vinifera]	91.798	5.3669	3.7	3	3	3	0.975	1.001	1.035	0.994	0.977	1.03	0.997	8.95E-01

cds.Camellia_sinensis.comp31868_c0_seq1_m.13263	PREDICTED: soluble starch synthase 1, chloroplastic/amyloplastic [Ricinus communis]	71.693	31.484	15.4	7	7	7	0.937	0.982	0.963	0.985	1.056	1.098	1.089	7.06E-02
cds.Camellia_sinensis.comp31870_c0_seq1_m.10965	PREDICTED: probable cadmium/zinc-transporting ATPase HMA1, chloroplastic [Sesamum indicum]	44.186	2.7391	3.8	2	2	2	1.133	1.079	1.109	0.849	0.934	0.867	0.798	2.16E-03
cds.Camellia_sinensis.comp31872_c0_seq1_m.4416	PREDICTED: 4-alpha-glucanotransferase DPE2-like [Populus euphratica]	54.165	26.595	19.2	7	9	7	1	1.006	1.047	0.957	0.966	0.988	0.953	5.11E-02
cds.Camellia_sinensis.comp31872_c0_seq1_m.4417	PREDICTED: 4-alpha-glucanotransferase DPE2 [Prunus mume]	45.813	31.861	23.6	8	15	8	1.068	1.019	1.001	0.939	0.975	0.989	0.94	6.75E-02

cds.Camellia_sinensis.comp31876_c1_seq9_m.17023	PREDICTED: protein TRANSPARENT TESTA 12 [Jatropha curcas]	52.1	3.0324	5.4	3	3	3	0.986	0.974	1.029	0.976	1.015	1.03	1.011	6.69E-01
cds.Camellia_sinensis.comp31879_c0_seq1_m.17822	PREDICTED: transcription factor HBP-1b(c38)-like [Sesamum indicum]	20.744	2.0083	7.3	1	1	1								
cds.Camellia_sinensis.comp31879_c0_seq3_m.23557	PREDICTED: transcription factor HBP-1b(c1)-like isoform X1 [Sesamum indicum]	36.731	2.0037	2.1	1	1	1								

cds.Camellia_sinensis.comp31884_c0_seq1_m.15173	PREDICTED: mitochondrial-processing peptidase subunit alpha [Musa acuminata subsp. malaccensis]	54.485	182.83	44	17	41	17	1.019	0.985	0.996	0.991	1.019	0.994	1.001	9.24E-01
cds.Camellia_sinensis.comp31888_c0_seq4_m.43428	Metal-dependent phosphohydrolase [Arabidopsis thaliana]	25.147	12.217	16.6	3	3	3	1.163	1.175	1.164	0.81	0.803	0.837	0.7	1.53E-05
cds.Camellia_sinensis.comp31892_c0_seq1_m.18682	PREDICTED: 3-oxoacyl-[acyl-carrier-protein] synthase, mitochondrial isoform X1 [Vitis	50.769	27.358	7.2	2	2	2	0.822	1.18	1.104	1.109	0.873	0.938	0.94	6.98E-01

cds.Camellia_sinensis.comp31896_c0_seq2_m.899	PREDICTED: nuclear pore complex protein NUP205 isoform X1 [Vitis vinifera]	210.18	8.8411	2.8	6	6	6	0.925	1.003	0.967	1.055	0.962	0.985	1.037	3.76E-01
cds.Camellia_sinensis.comp31897_c0_seq5_m.20303	PREDICTED: adenylate kinase 5, chloroplastic isoform X9 [Eucalyptus grandis]	37.035	116.82	49.5	15	35	14	0.979	0.964	0.933	1.077	1.011	1.036	1.086	2.37E-02
cds.Camellia_sinensis.comp31897_c0_seq5_m.20304	PREDICTED: adenylate kinase 5, chloroplastic [Prunus mume]	24.94	63.793	35.3	6	15	6	0.943	0.978	0.944	1.045	1.045	1.03	1.089	2.70E-03
cds.Camellia_sinensis.comp31900_c0_seq10_c0_seq1_m.9317	PREDICTED: UPF0261 protein mll9388 [Daucus carota subsp. sativus]	81.029	18.749	7.8	6	6	6	0.964	0.974	0.964	1.041	1.062	0.999	1.069	2.24E-02

cds.Camellia_sinensis.comp31905_c0_seq4_m.29858	PREDICTED: gamma-soluble NSF attachment protein [Citrus sinensis]	25.122	34.24	21.8	5	5	5	0.96	1.033	1.027	1.025	1.017	1.003	1.008	7.38E-01
cds.Camellia_sinensis.comp31906_c0_seq7_m.42862	PREDICTED: probable protein phosphatase 2C 11 [Nicotiana tabacum]	11.134	16.463	19	1	1	1	1.142	1.056	0.829	1.074	0.885	1.006	0.98	9.01E-01
cds.Camellia_sinensis.comp31907_c0_seq4_m.34738	PREDICTED: protein STRICTOSIDINE SYNTHASE-LIKE 10 [Prunus mume]	31.691	5.4191	8.3	2	3	2	0.89	1.042	0.947	1.012	0.997	1.135	1.092	2.24E-01
cds.Camellia_sinensis.comp31908_c0_seq1_m.34676	PREDICTED: UDP-glycosyltransferase 88A1 [Vitis vinifera]	41.835	5.5749	8.2	3	3	3	0.9	1.003	0.938	1.047	1.021	1.05	1.098	4.57E-02
cds.Camellia_sinensis.comp31908_c1_seq1_m.55064	PREDICTED: UDP-glycosyltransferase 88A1-like [Vitis vinifera]	12.391	18.292	26.1	2	4	2	0.962	1.028	0.935	1.014	1.097	0.982	1.057	2.68E-01

cds.Camellia_sinensis.comp31909_c0_seq1_m.17647	PREDICTED: 26S proteasome regulatory subunit 4 homolog A [Sesamum	49.555	81.331	42.7	19	25	19	1.173	1.171	1.16	0.817	0.807	0.794	0.69	1.02E-06
cds.Camellia_sinensis.comp31910_c0_seq2_m.21802	PREDICTED: guanosine nucleotide diphosphate dissociation inhibitor 2 isoform X2 [Ziziphus jujuba]	49.602	192.48	39.2	16	42	16	0.99	0.963	0.968	0.998	1.038	1.035	1.051	3.04E-02
cds.Camellia_sinensis.comp31911_c0_seq1_m.1537	PREDICTED: clustered mitochondria protein [Vitis vinifera]	155.82	19.682	4.4	5	5	5	1.089	1.12	1.113	0.854	0.846	0.942	0.795	2.88E-03
cds.Camellia_sinensis.comp31913_c0_seq1_m.21576	PREDICTED: peroxiredoxin Q, chloroplastic [Ricinus communis]	24.214	134.98	46.8	9	34	9	1.061	1.015	1.024	0.961	0.953	0.963	0.928	5.88E-03

cds.Camellia_sinensis.comp31916_c0_seq1_m.9389	PREDICTED: calcium-dependent protein kinase 1 [Theobroma cacao]	64.326	3.4849	3.3	2	4	2	0.96	0.964	0.955	1.057	1.049	1.056	1.098	1.66E-05
cds.Camellia_sinensis.comp31922_c0_seq2_m.26835	PREDICTED: ATP-citrate synthase alpha chain protein 2 [Nicotiana attenuata]	48.023	1.21	10.3	5	9	2	1.348	1.238	1.215	0.647	0.688	0.769	0.554	5.81E-04
cds.Camellia_sinensis.comp31929_c0_seq3_m.23533	PREDICTED: lysophospholipid acyltransferase LPEAT1 isoform X1 [Nelumbo nucifera]	43.816	1.6855	2.1	1	1	1	1.046	1.001	0.87	0.987	1.048	1.054	1.059	3.70E-01
cds.Camellia_sinensis.comp31929_c0_seq8_m.42956	PREDICTED: probable protein phosphatase 2C 62 isoform X1 [Sesamum indicum]	18.358	1.8239	7.1	1	1	1								

cds.Camellia_sinensis.comp31931_c0_seq1_m.7532	PREDICTED: pyrophosphate-energized vacuolar membrane proton pump-like [Ipomoea nil]	80.572	120.24	18.5	13	34	7	1.026	0.969	0.994	0.979	1.019	0.993	1.001	9.70E-01
cds.Camellia_sinensis.comp31936_c0_seq1_m.9484	PREDICTED: calcium-transporting ATPase, endoplasmic reticulum-type [Vitis vinifera]	85.11	1.8695	3.4	3	6	1	1.056	1.036	1.008	0.968	1.01	0.92	0.935	8.75E-02
cds.Camellia_sinensis.comp31946_c0_seq1_m.19079	PREDICTED: DEAD-box ATP-dependent RNA helicase 38 [Ipomoea nil]	54.44	94.859	29.7	13	22	13	0.954	0.977	0.968	1.009	1.061	1.053	1.077	1.23E-02
cds.Camellia_sinensis.comp31950_c0_seq1_m.31380	PREDICTED: probable proteasome inhibitor [Ziziphus jujuba]	31.92	19.493	14.5	4	7	4	1.01	0.957	0.935	1.046	0.995	1.094	1.08	9.71E-02

cds.Camellia_sinensis.comp31952_c0_seq2_m.12569	PREDICTED: pentatricopeptide repeat-containing protein At1g26460, mitochondrial [Nelumbo nucifera]	69.016	6.5756	2.7	2	2	2	1.055	0.92	1.033	0.969	1.006	1.02	0.996	9.54E-01
cds.Camellia_sinensis.comp31955_c0_seq7_m.21954	PREDICTED: nicotinate-nucleotide pyrophosphorylase [carboxylating], chloroplastic [Jatropha curcas]	35.621	37.042	17.9	6	7	6	0.985	1.022	0.98	1.009	0.994	1.04	1.019	3.78E-01
cds.Camellia_sinensis.comp31956_c0_seq1_m.37546	PREDICTED: granule-bound starch synthase 1, chloroplastic/amyloplastic-like [Ziziphus jujuba]	27.177	33.057	17.9	4	6	3	0.901	0.945	0.903	1.053	1.082	1.13	1.188	2.62E-03

cds.Camellia_sinensis.comp31956_c1_seq1_m.27245	PREDICTED: granule-bound starch synthase 1, chloroplastic/amyloplastic-like isoform X1 [Juglans regia]	41.147	12.727	14.3	5	6	5	0.908	0.914	0.968	1.063	1.099	1.083	1.163	2.54E-03
cds.Camellia_sinensis.comp31957_c0_seq1_m.21585	PREDICTED: ATP sulfurylase 1, chloroplastic [Vitis vinifera]	52.087	2.2585	3.9	2	3	1	1.021	1.154	1.015	0.905	0.935	0.957	0.877	4.43E-02
cds.Camellia_sinensis.comp31970_c0_seq1_m.27609	PREDICTED: GDSL esterase/lipase At5g55050 [Vitis vinifera]	40.373	3.1195	5.6	2	3	2	0.959	1.106	1.098	0.953	0.948	0.937	0.897	1.50E-01
cds.Camellia_sinensis.comp31972_c0_seq3_m.32434	PREDICTED: LOW QUALITY PROTEIN: ammonium transporter 1 member 2 [Citrus	28.097	58.859	18.9	4	8	4	1.054	0.997	0.98	0.995	1.003	1.001	0.989	6.92E-01

cds.Camellia_sinensis.comp31973_c2_seq4_m.32928	PREDICTED: protein-lysine N-methyltransferase Mettl10-like [Daucus carota subsp. sativus]	37.398	3.0913	20.8	6	6	1								
cds.Camellia_sinensis.comp31973_c2_seq5_m.36078	PREDICTED: protein-lysine N-methyltransferase Mettl10 [Vitis vinifera]	37.447	24.782	25.8	7	7	2	0.927	1.001	1.04	1.037	0.881	1.131	1.027	7.92E-01
cds.Camellia_sinensis.comp31974_c0_seq2_m.16924	PREDICTED: T-complex protein 1 subunit delta [Cucumis melo]	57.783	97.147	30.5	18	31	18	0.918	0.958	0.941	1.069	1.049	1.063	1.129	8.59E-04
cds.Camellia_sinensis.comp31976_c1_seq1_m.38493	PREDICTED: ferritin-3, chloroplastic [Theobroma cacao]	28.646	153.31	42.4	10	88	10	0.948	0.949	0.974	1.061	1.06	1.043	1.102	7.41E-04

cds.Camellia_sinensis.comp31977_c0_seq1_m.18004	PREDICTED: serine/threonine-protein kinase STY46-like [Gossypium arboreum]	48.339	12.914	18.6	9	10	8	0.954	0.977	1.045	1.007	1.025	1.02	1.026	4.03E-01
cds.Camellia_sinensis.comp31981_c0_seq1_m.2451	PREDICTED: structural maintenance of chromosomes protein 3 [Daucus carota subsp. sativus]	76.744	7.1283	5.5	4	4	4	1.098	1.138	1.051	0.902	0.943	0.927	0.843	2.98E-03
cds.Camellia_sinensis.comp31981_c0_seq1_m.2452	PREDICTED: structural maintenance of chromosomes protein 3 [Vitis vinifera]	63.56	8.4056	5.8	4	5	4	1.059	1.108	1.146	0.873	0.884	0.886	0.798	6.41E-04
cds.Camellia_sinensis.comp31984_c0_seq1_m.21525	PREDICTED: arabinosyltransferase RRA3-like [Nelumbo nucifera]	48.963	2.9882	2.3	1	1	1	1.008	1.028	0.895	1.044	1.011	1.027	1.052	3.05E-01

cds.Camellia_sinensis.comp31984_c1_seq4_m.33674	adaptin ear-binding coat-associated protein 1 [Medicago truncatula]	30.531	24.374	13.4	3	3	3	1.054	0.976	1.056	0.934	0.981	0.956	0.93	7.23E-02
cds.Camellia_sinensis.comp31984_c2_seq1_m.13214	peptidyl-prolyl cis-trans isomerase CYP71 [Cajanus cajan]	37.666	3.5294	9.6	3	3	3	0.934	1.01	0.976	1.054	1.005	1.044	1.063	8.57E-02
cds.Camellia_sinensis.comp31984_c2_seq1_m.13215	PREDICTED: peptidyl-prolyl cis-trans isomerase CYP71-like [Gossypium hirsutum]	17.258	2.6346	11.8	2	2	2								
cds.Camellia_sinensis.comp31990_c0_seq1_m.12729	PREDICTED: CSC1-like protein HYP1 isoform X2 [Vitis vinifera]	57.491	7.3314	8.3	4	4	4	1.006	0.99	0.945	0.949	1.049	1.068	1.043	3.80E-01

cds.Camellia_sinensis.comp32000_c0_seq1_m.11354	PREDICTED: FRIGIDA-like protein 3 [Vitis vinifera]	42.698	42.802	25.6	9	12	9	1.019	1.051	1.03	0.958	1.015	0.938	0.939	6.61E-02
cds.Camellia_sinensis.comp32006_c0_seq1_m.11509	PREDICTED: thylakoid lumenal 17.9 kDa protein, chloroplastic [Nelumbo nucifera]	25.818	42.195	33.6	6	10	6	0.963	0.909	0.946	1.04	1.013	1.094	1.117	1.76E-02
cds.Camellia_sinensis.comp32007_c0_seq2_m.24766	PREDICTED: 1-acyl-sn-glycerol-3-phosphate acyltransferase 2 [Vigna angularis]	28.159	10.727	11.2	3	3	3	0.97	1.011	1.117	0.977	0.966	0.966	0.939	2.83E-01
cds.Camellia_sinensis.comp32009_c0_seq1_m.9085	PREDICTED: ABC transporter D family member 2, chloroplastic [Nelumbo nucifera]	20.86	1.9763	5.3	1	1	1	0.958	0.969	1.042	0.934	1.027	1.08	1.024	6.72E-01

cds.Camellia_sinensis.comp32011_c0_seq1_m.24958	PREDICTED: mitochondrial import inner membrane translocase subunit TIM17-2-like [Daucus carota subsp.sativus]	24.116	1.8303	3.8	1	1	1	0.979	1.004	0.995	1.009	0.993	1.031	1.018	2.38E-01
cds.Camellia_sinensis.comp32013_c0_seq2_m.11407	PREDICTED: pyrophosphate--fructose 6-phosphate 1-phosphotransferase subunit alpha [Sesamum indicum]	67.174	6.2866	23.2	11	15	2	1.004	1.058	0.981	0.907	0.936	0.948	0.917	2.93E-02
cds.Camellia_sinensis.comp32013_c0_seq3_m.12719	PREDICTED: pyrophosphate--fructose 6-phosphate 1-phosphotransferase subunit alpha [Sesamum indicum]	67.297	59.581	27.7	13	17	4	0.945	0.966	0.949	1.029	1.048	1.087	1.106	4.48E-03

cds.Camellia_sinensis.comp32014_c0_seq2_m.20692	PREDICTED: methylthioribose kinase [Vitis vinifera]	47.734	74.525	32.5	13	21	13	0.935	0.963	0.936	1.055	1.064	1.059	1.121	3.23E-04
cds.Camellia_sinensis.comp32016_c1_seq1_m.16328	PREDICTED: probable leucine-rich repeat receptor-like protein kinase At5g49770 [Capsicum	74.639	3.6044	3.5	3	3	2	0.934	0.952	0.973	1.086	1.025	1.058	1.108	7.38E-03
cds.Camellia_sinensis.comp32023_c0_seq13_m.7956	PREDICTED: conserved oligomeric Golgi complex subunit 4 [Erythranthe guttata]	65.246	1.7826	2	1	1	1	0.951	1.026	1.126	1.021	0.96	0.929	0.938	3.27E-01
cds.Camellia_sinensis.comp32026_c0_seq1_m.14730	PREDICTED: glucosidase 2 subunit beta isoform X1 [Vitis vinifera]	69.482	2.4058	3.4	2	2	2								

cds.Camellia_sinensis.comp32027_c0_seq1_m.3932	PREDICTED: ATPase 10, plasma membrane-type [Eucalyptus grandis]	104.46	196.46	38.8	32	80	22	0.998	0.988	1.01	1	1.012	1.004	1.007	4.10E-01
cds.Camellia_sinensis.comp32027_c0_seq4_m.14972	transmembrane protein, putative [Medicago truncatula]	26.309	8.107	15.8	3	3	3								
cds.Camellia_sinensis.comp32029_c0_seq1_m.29689	PREDICTED: photosynthetic NDH subunit of subcomplex B 2, chloroplastic [Juglans regia]	40.599	58.33	20.1	6	7	6	0.994	0.986	1.012	1.022	1.015	0.985	1.01	5.09E-01
cds.Camellia_sinensis.comp32030_c0_seq3_m.42437	PREDICTED: thymidylate kinase isoform X2 [Sesamum indicum]	15.97	2.9187	7.6	1	1	1	0.96	1.085	0.955	1	0.97	1.04	1.003	9.24E-01
cds.Camellia_sinensis.comp32031_c0_seq1_m.16650	PREDICTED: citrate synthase, glyoxysomal [Sesamum indicum]	56.278	49.115	21.5	10	16	10	0.965	0.967	0.984	1.059	1.029	1.02	1.066	7.86E-03

cds.Camellia_sinensis.comp32035_c0_seq3_m.19627	PREDICTED: dolichyl-diphosphooligosaccharide--protein glycosyltransferase 48 kDa subunit isoform X3 [Vitis vinifera]	48.95	14.938	13.5	6	11	6	1.002	0.987	0.955	1.01	1.043	1.016	1.042	7.34E-02
cds.Camellia_sinensis.comp32035_c0_seq4_m.21981	PREDICTED: scopoletin glucosyltransferase [Theobroma cacao]	52.919	24.768	18.4	9	15	9	0.93	0.978	0.96	1.067	1.072	1.033	1.106	5.78E-03
cds.Camellia_sinensis.comp32036_c0_seq1_m.17656	PREDICTED: probable solanesyl-diphosphate synthase 3, chloroplastic [Ricinus communis]	46.323	4.6719	6.7	3	4	3	0.974	0.973	1.02	0.968	0.957	1.118	1.026	6.83E-01

cds.Camellia_sinensis.comp32037_c2_seq4_m.5633	PREDICTED: KH domain-containing protein At4g18375 isoform X2 [Eucalyptus grandis]	58.48	97.132	40.2	19	24	17	1.022	1.04	1.04	0.996	0.956	0.946	0.934	1.52E-02
cds.Camellia_sinensis.comp32037_c2_seq5_m.11381	PREDICTED: KH domain-containing protein At4g18375-like isoform X1 [Nicotiana tabacum]	57.174	128.33	26.6	14	18	13	1.042	1.059	1.049	0.933	0.963	0.957	0.906	7.76E-04
cds.Camellia_sinensis.comp32038_c0_seq1_m.13087	PREDICTED: auxilin-related protein 2 [Vitis vinifera]	84.421	4.8131	2.6	2	3	2	1.144	1.224	1.125	0.693	0.791	0.966	0.701	2.20E-02
cds.Camellia_sinensis.comp32040_c0_seq90_m.9512	PREDICTED: probable lactoylglutathione lyase, chloroplastic [Citrus sinensis]	38.267	65.407	36.8	12	46	6	0.962	0.95	0.983	1.04	1.031	1.032	1.072	2.56E-03

cds.Camellia_sinensis.comp32041_c0_seq1_m.33904	PREDICTED: haloacid dehalogenase-like hydrolase domain-containing protein 3 [Vitis vinifera]	32.839	6.4579	16.1	4	4	4	0.873	0.955	0.922	1.136	1.104	1.056	1.199	5.74E-03
cds.Camellia_sinensis.comp32054_c0_seq1_m.4386	PREDICTED: exportin-2 [Daucus carota subsp. sativus]	81.504	18.972	4.5	4	4	4	0.947	0.99	0.937	1.035	1.026	1.053	1.084	1.22E-02
cds.Camellia_sinensis.comp32054_c0_seq1_m.4387	PREDICTED: exportin-2 [Vitis vinifera]	27.506	24.096	16.3	3	5	3	0.942	0.992	0.977	1.06	1.05	1.003	1.069	4.32E-02
cds.Camellia_sinensis.comp32055_c0_seq13_m.33899	PREDICTED: inositol-pentakisphosphate 2-kinase [Vitis vinifera]	26.37	1.2853	3.8	1	1	1	0.99	0.996	1.065	0.997	0.987	0.973	0.969	2.76E-01
cds.Camellia_sinensis.comp32062_c0_seq4_m.10927	PREDICTED: putative clathrin assembly protein At2g25430 [Sesamum	71.016	22.404	9.9	5	7	5	1.228	1.063	0.947	0.863	0.938	0.969	0.855	1.41E-01

cds.Camellia_sinensis.comp32072_c0_seq1_m.9129	PREDICTED: zinc finger BED domain-containing protein DAYSLEEPER-like [Nicotiana tabacum]	77.118	22.41	9.1	5	6	2	0.963	0.992	0.979	1.004	1.009	1.069	1.05	8.93E-02
cds.Camellia_sinensis.comp32072_c0_seq2_m.9179	PREDICTED: zinc finger BED domain-containing protein DAYSLEEPER-like [Nicotiana tabacum]	77.498	1.802	6.5	4	5	1	0.955	1.09	0.989	0.98	0.971	1.023	0.98	6.85E-01
cds.Camellia_sinensis.comp32081_c0_seq1_m.30822	PREDICTED: omega-amidase, chloroplastic [Nicotiana attenuata]	40.89	120.55	28.6	9	25	9	0.927	0.948	0.965	1.075	1.034	1.05	1.112	2.78E-03

cds.Camellia_sinensis.comp32088_c0_seq1_m.18859	PREDICTED: 2-hydroxy-6-oxononadienedioate/2-hydroxy-6-oxononatrienedioate hydrolase isoform X1 [Vigna radiata var. radiata]	54.271	2.0886	2.2	1	1	1	1.099	0.987	0.957	0.952	0.924	1.071	0.968	6.27E-01
cds.Camellia_sinensis.comp32093_c0_seq1_m.17441	PREDICTED: pentatricopeptide repeat-containing protein At5g13770, chloroplastic [Nicotiana tomentosiformis]	72.428	1.2708	1.4	1	1	1								

cds.Camellia_sinensis.comp32094_c0_seq1_m.5391	PREDICTED: LOW QUALITY PROTEIN: calcium-transporting ATPase 4, plasma membrane-type-like [Ziziphus jujuba]	116.18	23.398	7.9	8	12	6	0.962	0.94	0.921	1.069	1.051	1.076	1.132	9.97E-04
cds.Camellia_sinensis.comp32095_c0_seq1_m.21396	PREDICTED: fructose-1,6-bisphosphatase, cytosolic [Jatropha curcas]	51.329	79.64	28.3	10	11	10	0.93	0.945	0.944	1.054	1.088	1.084	1.144	2.80E-04
cds.Camellia_sinensis.comp32098_c0_seq3_m.35699	PREDICTED: glycine-rich RNA-binding protein 2, mitochondrial-like isoform X2 [Nicotiana tomentosiformis]	28.961	134.76	21.5	4	8	1	1.077	0.956	0.998	1.046	0.977	0.95	0.981	6.96E-01

cds.Camellia_sinensis.comp32098_c0_seq8_m.39893	PREDICTED: glycine-rich RNA-binding protein blt801 [Vitis vinifera]	16.541	1.8011	37.3	4	9	1	0.976	1.014	0.973	0.989	1.062	1	1.03	3.25E-01
cds.Camellia_sinensis.comp32098_c1_seq1_m.2920	PREDICTED: RNA-binding protein Musashi homolog 2 [Vitis vinifera]	40.367	43.45	22.1	7	9	2								
cds.Camellia_sinensis.comp32098_c1_seq4_m.23978	PREDICTED: heterogeneous nuclear ribonucleoprotein A/B [Ziziphus jujuba]	39.804	2.5328	19.4	6	9	1	0.941	0.985	0.967	1.033	1.057	1.041	1.082	6.02E-03
cds.Camellia_sinensis.comp32098_c3_seq1_m.36836	PREDICTED: calmodulin-7 isoform X1 [Vitis vinifera]	16.848	125.03	55	11	46	11	1.001	0.967	0.97	1.004	1.046	1.034	1.05	4.21E-02

cds.Camellia_sinensis.comp32107_c0_seq1_m.31113	PREDICTED: probable inactive purple acid phosphatase 27 [Juglans regia]	48.189	9.3346	9.5	4	4	3	0.796	0.991	0.916	1.18	1.131	1.045	1.242	4.03E-02
cds.Camellia_sinensis.comp32112_c0_seq2_m.32185	PREDICTED: N-(5'-phosphoribosyl)anthranilate isomerase 1, chloroplastic isoform X2 [Ricinus communis]	29.997	8.5974	13.8	3	3	3	1.093	1.044	1.048	0.919	0.943	0.937	0.879	1.48E-03
cds.Camellia_sinensis.comp32115_c0_seq1_m.13747	PREDICTED: transcription initiation factor IIF subunit alpha-like isoform X1 [Juglans regia]	59.741	1.9279	3.3	1	1	1								

cds.Camellia_sinensis.comp32116_c0_seq3_m.15155	PREDICTED: probable protein phosphatase 2C 60 isoform X1 [Nelumbo nucifera]	25.977	6.0133	15.8	5	7	4	0.942	0.946	0.993	1.05	1.035	1.039	1.084	9.62E-03
cds.Camellia_sinensis.comp32116_c0_seq6_m.22215	PREDICTED: probable protein phosphatase 2C 60 [Pyrus x bretschneideri]	10.92	1.3693	8.1	1	1	1								
cds.Camellia_sinensis.comp32119_c0_seq1_m.26230	PREDICTED: protein FLX-like 1 [Nelumbo nucifera]	33.426	4.9706	9.1	3	3	2	1.008	0.99	0.988	1.048	0.932	1.044	1.013	7.88E-01
cds.Camellia_sinensis.comp32127_c0_seq1_m.30393	PREDICTED: short-chain dehydrogenase TIC 32, chloroplastic [Vitis vinifera]	34.14	63.954	16.8	3	4	3	0.975	1.035	0.968	0.963	1.018	1.009	1.004	8.86E-01
cds.Camellia_sinensis.comp32128_c0_seq1_m.17622	PREDICTED: fumarylacetoacetase [Jatropha curcas]	42.157	52.045	16.7	6	8	6	1.05	1.03	1.052	0.945	0.963	0.949	0.912	4.82E-04

cds.Camellia_sinensis.comp32129_c0_seq4_m.28333	PREDICTED: sugar transporter ERD6-like 5 isoform X1 [Daucus carota subsp. sativus]	47.497	8.9092	5.9	4	5	4	0.946	0.972	0.988	1.05	1.076	1.023	1.084	1.42E-02
cds.Camellia_sinensis.comp32129_c0_seq5_m.28757	PREDICTED: syntaxin-121-like [Juglans regia]	22.938	17.293	15.2	3	4	3	0.917	0.925	0.976	1.062	1.087	1.093	1.15	2.78E-03
cds.Camellia_sinensis.comp32133_c0_seq2_m.52215	PREDICTED: purple acid phosphatase 2-like [Nicotiana tabacum]	15.569	1.7413	5	1	3	1	0.961	0.997	1.046	0.994	1.051	0.967	1.003	9.43E-01
cds.Camellia_sinensis.comp32134_c0_seq1_m.14166	PREDICTED: sorcin [Eucalyptus grandis]	19.439	1.9488	5.9	1	3	1								
cds.Camellia_sinensis.comp32141_c1_seq1_m.28717	PREDICTED: phosphoglucan phosphatase DSP4, amyloplastic-like isoform X2 [Juglans regia]	44.121	17.365	10.4	4	4	3	0.996	0.986	1.045	1.004	1.028	0.952	0.986	6.43E-01

cds.Camellia_sinensis.comp32142_c0_seq1_m.26960	oxidoreductase family protein [Populus trichocarpa]	34.503	52.481	31.7	10	22	10	0.977	0.967	0.991	1.015	1.03	1.025	1.046	5.64E-03
cds.Camellia_sinensis.comp32145_c0_seq15_m.12715	PREDICTED: UDP-sugar pyrophosphorylase [Ziziphus jujuba]	68.995	43.801	24.8	17	23	17	1.005	1.001	1.021	0.995	0.975	1	0.981	1.25E-01
cds.Camellia_sinensis.comp32151_c0_seq1_m.30264	PREDICTED: anamorsin homolog isoform X3 [Vitis vinifera]	29.862	15.633	20.7	4	5	4	0.942	1.075	1.001	0.989	1.053	0.946	0.99	8.56E-01
cds.Camellia_sinensis.comp32152_c0_seq1_m.21546	PREDICTED: D-glycerate 3-kinase, chloroplastic [Juglans regia]	51.891	37.865	20.8	8	16	8	1.01	1.004	0.986	0.992	0.999	0.972	0.988	3.20E-01
cds.Camellia_sinensis.comp32154_c0_seq34_m.14388	lycopene beta/epsilon cyclase [Medicago truncatula]	24.114	7.3996	11.3	2	2	2	1.033	0.972	1.046	1.046	0.927	0.981	0.968	4.72E-01

cds.Camellia_sinensis.comp32155_c0_seq1_m.31991	PREDICTED: succinate dehydrogenase subunit 5, mitochondrial [Vitis vinifera]	29.706	125.41	42.7	9	19	9	0.999	1.017	0.985	1.024	0.981	0.982	0.995	7.91E-01
cds.Camellia_sinensis.comp32158_c0_seq2_m.17123	PREDICTED: protein ASPARTIC PROTEASE IN GUARD CELL 1-like [Nicotiana tabacum]	49.376	62.233	20.3	6	6	6	1.008	1.064	1.095	0.967	0.916	0.89	0.876	1.80E-02
cds.Camellia_sinensis.comp32158_c1_seq1_m.14727	PREDICTED: patellin-3 [Populus euphratica]	57.719	176.63	40.6	19	45	18	0.947	0.92	0.95	1.073	1.078	1.059	1.14	3.42E-04
cds.Camellia_sinensis.comp32162_c0_seq1_m.28224	PREDICTED: 3-hydroxyisobutyryl-CoA hydrolase 1-like [Juglans regia]	43.603	12.381	15	5	7	3	0.961	1.047	1.022	1.007	0.951	1.023	0.984	6.58E-01

cds.Camellia_sinensis.comp32162_c0_seq3_m.43558	PREDICTED: 3-hydroxyisobutyryl-CoA hydrolase 1 isoform X1 [Vitis vinifera]	28.973	48.369	24.2	5	8	3	0.887	1.06	0.997	1.046	0.959	1.075	1.046	4.97E-01
cds.Camellia_sinensis.comp32168_c0_seq1_m.16101	PREDICTED: regulation of nuclear pre-mRNA domain-containing protein 2 isoform X2 [Theobroma cacao]	55.617	1.8075	2	1	1	1								

cds.Camellia_sinensis.comp32175_c0_seq1_m.9849	PREDICTED: trifunctional UDP-glucose 4,6-dehydratase/UDP-4-keto-6-deoxy-D-glucose 3,5-epimerase/UDP-4-keto-L-rhamnose-reductase RHM1 [Vitis vinifera]	75.906	22.214	13.8	10	10	9	1.053	1.043	1.016	0.959	0.985	0.962	0.934	7.36E-03
cds.Camellia_sinensis.comp32183_c0_seq1_m.8166	PREDICTED: UDP-N-acetylmuramoyl-L-alanyl-D-glutamate--2,6-diaminopimelate ligase-like [Nicotiana tabacum]	86.924	2.953	2	2	2	2	1.063	1.098	1.123	0.872	0.914	0.91	0.821	8.40E-04

cds.Camellia_sinensis.comp32188_c0_seq1_m.41118	PREDICTED: mitochondrial outer membrane protein porin 2-like [Nicotiana attenuata]	29.782	17.572	37.4	11	26	5	1.023	1.018	0.985	0.97	1.018	1.027	0.996	8.67E-01
cds.Camellia_sinensis.comp32188_c0_seq2_m.46109	PREDICTED: mitochondrial outer membrane protein porin 2-like [Nicotiana attenuata]	24.947	54.364	41.7	11	29	5	0.971	0.999	0.988	1.003	1.022	1.023	1.03	4.57E-02
cds.Camellia_sinensis.comp32189_c0_seq1_m.16263	PREDICTED: inosine-5'-monophosphate dehydrogenase 2-like isoform X1 [Nelumbo nucifera]	53.517	95.67	30.2	12	22	12	0.948	0.921	0.941	1.064	1.07	1.062	1.137	1.38E-04
cds.Camellia_sinensis.comp32202_c0_seq1_m.40220	PREDICTED: 40S ribosomal protein S24-1 [Sesamum indicum]	15.8	2.504	11.7	2	5	2	1.108	1.129	1.076	0.859	0.894	0.928	0.809	1.30E-03

cds.Camellia_sinensis.comp32203_c0_seq1_m.3830	PREDICTED: presequence protease 1, chloroplastic/mitochondrial-like isoform X1 [Ipomoea nil]	134.51	323.31	47.1	54	133	54	0.998	0.993	0.99	1.008	1.01	1.013	1.017	3.78E-03
cds.Camellia_sinensis.comp32205_c0_seq1_m.13071	PREDICTED: outer envelope protein 64, chloroplastic-like [Nelumbo nucifera]	64.388	4.7647	10.5	7	7	4	1.043	1.014	0.95	0.946	0.967	1.078	0.995	9.05E-01
cds.Camellia_sinensis.comp32205_c0_seq2_m.13339	PREDICTED: outer envelope protein 64, chloroplastic-like [Nelumbo nucifera]	64.334	48.641	24.2	13	16	10	0.953	0.969	0.959	1.043	1.078	1.052	1.101	9.38E-04
cds.Camellia_sinensis.comp32207_c0_seq1_m.13675	PREDICTED: phosphomannomutase/phosphoglucomutase isoform X1 [Prunus	69.24	40.24	20.3	11	18	11	0.976	0.954	0.971	1.026	1.018	1.048	1.066	4.54E-03

cds.Camellia_sinensis.comp32209_c0_seq1_m.29095	arginase 1 [Solanum lycopersicum]	41.886	86.806	30.4	10	21	9	1.041	1.117	1.08	0.938	0.922	0.914	0.857	2.08E-03
cds.Camellia_sinensis.comp32212_c1_seq1_m.2978	PREDICTED: pumilio homolog 2 [Theobroma cacao]	113.63	30.888	5.8	4	4	4	1.041	1.014	1.017	1.067	0.957	1.009	0.987	6.95E-01
cds.Camellia_sinensis.comp32212_c2_seq1_m.2678	PREDICTED: ABC transporter B family member 1 [Ipomoea nil]	147.41	7.0531	5.1	5	6	4	1.104	0.98	1.126	0.895	0.952	0.927	0.864	3.70E-02
cds.Camellia_sinensis.comp32214_c0_seq1_m.34384	PREDICTED: 14-3-3-like protein GF14 kappa [Daucus carota subsp. sativus]	28.611	80.223	53.8	13	33	6	0.951	0.953	0.881	1.108	1.104	1.051	1.172	6.84E-03
cds.Camellia_sinensis.comp32214_c0_seq3_m.43656	PREDICTED: 14-3-3-like protein GF14 kappa [Daucus carota subsp. sativus]	20.201	56.274	47.5	6	20	3	1.017	1.01	0.953	0.984	1.083	0.993	1.027	5.20E-01

cds.Camellia_sinensis.comp32220_c0_seq1_m.15769	PREDICTED: trigger factor-like protein TIG, Chloroplastic [Vitis vinifera]	61.406	133.2	35.8	18	30	18	1.011	0.999	0.997	1.006	1.009	0.993	1	9.63E-01
cds.Camellia_sinensis.comp32222_c0_seq1_m.16474	PREDICTED: BTB/POZ domain-containing protein At5g48800 isoform X1 [Vitis vinifera]	69.478	1.2464	3.6	3	3	2	0.997	0.975	1.051	0.99	1.012	0.985	0.988	6.52E-01
cds.Camellia_sinensis.comp32225_c0_seq2_m.10673	PREDICTED: sulfite reductase [ferredoxin], chloroplastic [Citrus sinensis]	72.426	78.367	30.8	19	39	19	0.989	0.978	0.971	1.021	1.028	1.014	1.043	3.30E-03
cds.Camellia_sinensis.comp32228_c0_seq1_m.50575	PREDICTED: putative lipid-binding protein At4g00165 [Juglans regia]	12.894	1.3554	6.5	1	1	1								

cds.Camellia_sinensis.comp32229_c0_seq1_m.3901	PREDICTED: phosphoenolpyruvate carboxylase 4 [Eucalyptus grandis]	119.31	126.11	26.4	27	37	27	1.02	1.004	1.029	0.978	0.983	0.978	0.963	6.80E-03
cds.Camellia_sinensis.comp32232_c0_seq1_m.2058	PREDICTED: ABC transporter C family member 10-like [Populus euphratica]	147.83	11.382	2.9	3	5	1	1.162	0.973	0.83	1.012	0.99	1.025	1.021	7.88E-01
cds.Camellia_sinensis.comp32233_c0_seq3_m.16588	PREDICTED: serine/threonine-protein phosphatase 5 isoform X2 [Jatropha curcas]	54.998	3.4471	6.2	3	4	3	0.982	0.994	0.988	1.076	1.031	0.949	1.031	5.13E-01
cds.Camellia_sinensis.comp32237_c0_seq1_m.38904		13.007	32.337	42.6	6	14	6	1.102	0.986	0.994	0.902	0.995	1.025	0.948	3.67E-01

cds.Camellia_sinensis.comp32238_c0_seq2_m.1501	PREDICTED: nuclear pore complex protein NUP160 [Vitis vinifera]	168.45	2.9997	1.5	2	2	2								
cds.Camellia_sinensis.comp32239_c0_seq19_m.9961	PREDICTED: prolyl endopeptidase [Jatropha curcas]	88.772	55.459	10.9	10	23	8	1.001	1.003	0.995	0.988	1.024	1.02	1.011	4.03E-01
cds.Camellia_sinensis.comp32240_c0_seq10_m.17125	PREDICTED: pre-mRNA-processing factor 19 [Vitis vinifera]	56.825	13.909	13.5	7	9	6	0.929	0.973	1.006	1.044	0.963	1.003	1.035	3.53E-01
cds.Camellia_sinensis.comp32240_c0_seq30_m.46763	PREDICTED: pre-mRNA-processing factor 19 [Vitis vinifera]	13.225	4.1548	30.8	4	6	3	0.879	0.953	1.001	0.981	1.141	1.077	1.129	1.06E-01
cds.Camellia_sinensis.comp32243_c0_seq1_m.3211	PREDICTED: paladin [Vitis vinifera]	142.95	1.5251	0.8	1	1	1								

cds.Camellia_sinensis.comp32245_c0_seq1_m.4096	PREDICTED: probable 26S protease regulatory subunit 10B isoform X2 [Juglans regia]	50.495	1.3175	2.4	1	1	1	0.943	1.114	0.957	0.963	0.997	1.034	0.993	9.45E-01
cds.Camellia_sinensis.comp32245_c0_seq1_m.4098	PREDICTED: probable 26S protease regulatory subunit 10B isoform X1 [Juglans regia]	19.299	2.4147	13.5	2	2	2								
cds.Camellia_sinensis.comp32254_c0_seq1_m.30872	PREDICTED: strigolactone esterase D14 [Juglans regia]	32.449	36.651	21.9	6	12	6	1.024	1.049	1.055	0.945	0.961	0.941	0.91	1.08E-03
cds.Camellia_sinensis.comp32262_c0_seq1_m.19868	PREDICTED: actin-7 [Prunus mume]	41.695	253.54	47.5	17	120	2	0.991	1.021	1.004	1.015	0.98	0.988	0.989	4.65E-01

cds.Camellia_sinensis.comp32262_c1_seq1_m.35881	PREDICTED: 60S ribosomal protein L10a [Phoenix dactylifera]	24.516	24.037	32.4	7	17	7	1.039	1.037	1.029	0.962	1	0.974	0.946	8.94E-03
cds.Camellia_sinensis.comp32271_c0_seq1_m.19075	PREDICTED: glutathione synthetase, chloroplastic isoform X1 [Vitis vinifera]	62.209	15.9	11.7	7	9	6	0.895	0.94	0.939	1.102	1.042	1.085	1.164	2.84E-03
cds.Camellia_sinensis.comp32271_c0_seq2_m.46601	PREDICTED: glutathione synthetase, chloroplastic isoform X3 [Vitis vinifera]	25.792	8.9201	13.5	3	4	2	0.995	1.015	0.993	1.079	1.005	0.929	1.003	9.74E-01
cds.Camellia_sinensis.comp32272_c0_seq3_m.2750	PREDICTED: protein MODIFIER OF SNC1 1 [Vitis vinifera]	99.007	2.2957	2.2	2	2	2								

cds.Camellia_sinensis.comp32275_c0_seq1_m.40576	PREDICTED: germin-like protein subfamily T member 1 [Juglans regia]	22.619	29.867	27.2	5	13	5	0.898	0.901	0.932	1.133	1.14	1.091	1.232	3.24E-04
cds.Camellia_sinensis.comp32284_c0_seq1_m.945	PREDICTED: protein SHOOT GRAVITROPISM 6 isoform X2 [Vitis vinifera]	62.846	5.9657	2.1	1	1	1	0.959	0.981	1.025	1.146	0.988	0.928	1.033	6.85E-01
cds.Camellia_sinensis.comp32284_c0_seq3_m.1022	PREDICTED: protein SHOOT GRAVITROPISM 6 isoform X4 [Vitis vinifera]	68.995	5.3556	5.5	3	3	3	0.979	0.993	0.957	1.167	0.976	0.952	1.057	4.76E-01
cds.Camellia_sinensis.comp32285_c1_seq7_m.22791	PREDICTED: serine/threonine-protein kinase HT1-like [Sesamum indicum]	41.091	8.8736	6.5	2	3	2	0.88	0.876	0.964	1.122	1.048	1.162	1.225	9.54E-03

cds.Camellia_sinensis.comp32288_c0_seq1_m.43494	peroxidase P7 precursor [Capsicum annuum]	25.098	45.347	31.4	7	15	5	0.933	0.878	0.927	1.108	1.105	1.064	1.197	1.52E-03
cds.Camellia_sinensis.comp32293_c1_seq1_m.24238	thioredoxin family protein [Populus trichocarpa]	47.782	6.649	10.6	4	5	2	1.013	1.113	1.093	0.949	0.916	0.909	0.862	9.54E-03
cds.Camellia_sinensis.comp32293_c1_seq2_m.25320	PREDICTED: protein disulfide isomerase-like 2-3 [Juglans regia]	48.101	12.993	10.2	4	5	2	1.065	1.008	1.05	1.006	0.907	0.988	0.929	1.07E-01
cds.Camellia_sinensis.comp32304_c0_seq1_m.3254	phenylalanine ammonia-lyase family protein [Populus trichocarpa]	77.185	5.9738	28.2	19	41	1								
cds.Camellia_sinensis.comp32304_c0_seq3_m.11225	PREDICTED: phenylalanine ammonia-lyase [Vitis vinifera]	77.077	118.08	37.8	25	54	6	1.375	1.378	1.392	0.561	0.579	0.587	0.417	1.92E-07

cds.Camellia_sinensis.comp32304_c0_seq5_m.38144	PREDICTED: phenylalanine ammonia-lyase [Vitis vinifera]	30.151	2.475	15.7	5	10	0								
cds.Camellia_sinensis.comp32311_c0_seq1_m.42109	NADPH:QUINONE OXIDOREDUCTASE family protein [Populus trichocarpa]	22.987	38.035	31.2	4	4	4	0.963	1.054	1.012	1.034	0.953	0.968	0.976	5.32E-01
cds.Camellia_sinensis.comp32313_c0_seq1_m.11206	PREDICTED: protein BONZAI 1-like [Juglans regia]	65.25	1.3509	3	2	2	2	0.897	1.011	0.889	1.173	1.085	0.988	1.161	8.37E-02
cds.Camellia_sinensis.comp32314_c0_seq2_m.7150	PREDICTED: guanine nucleotide-binding protein subunit beta-2 [Citrus sinensis]	41.112	10.711	8	3	4	3	0.98	0.951	0.963	1.03	1.067	1.045	1.086	3.62E-03

cds.Camellia_sinensis.comp32315_c0_seq4_m.34834	PREDICTED: protein LUTEIN DEFICIENT 5, chloroplastic [Ipomoea nil]	34.82	1.8687	2.9	1	1	1								
cds.Camellia_sinensis.comp32321_c0_seq1_m.22581	PREDICTED: V-type proton ATPase subunit H [Theobrom	55.987	59.034	15.4	7	11	7	0.967	0.959	0.974	1.044	1.077	1.006	1.078	2.07E-02
cds.Camellia_sinensis.comp32323_c0_seq1_m.26511	PREDICTED: pyruvate dehydrogenase (acetyl-transferring) kinase, mitochondrial-like [Nicotiana attenuata]	41.962	1.5816	4.3	1	1	1								
cds.Camellia_sinensis.comp32324_c0_seq8_m.30857	PREDICTED: probable polyamine aminopropyl transferase [Malus domestica]	18.543	3.8695	5.9	1	1	1	1.069	1.053	0.972	0.993	0.927	0.979	0.937	1.49E-01

cds.Camellia_sinensis.comp32335_c0_seq1_m.14824	PREDICTED: putative 3,4-dihydroxy-2-butanone kinase [Malus	53.565	109.98	36.3	15	25	15	0.959	0.946	0.938	1.088	1.029	1.044	1.112	4.10E-03
cds.Camellia_sinensis.comp32336_c2_seq3_m.47894	PREDICTED: transmembrane protein 33 homolog [Juglans regia]	17.468	42.018	19.9	2	3	2	0.934	0.938	0.954	1.032	1.099	1.026	1.117	8.58E-03
cds.Camellia_sinensis.comp32341_c1_seq1_m.31959	PREDICTED: glutamate dehydrogenase 2 [Gossypium raimondii]	32.818	18.896	24.3	6	9	4	0.973	0.965	0.942	1.122	1.015	1.075	1.115	2.40E-02
cds.Camellia_sinensis.comp32343_c1_seq1_m.13967	PREDICTED: fatty acid amide hydrolase-like [Sesamum indicum]	66.642	2.9938	3.3	2	2	2	1.191	1.182	1.254	0.774	0.74	0.799	0.638	1.00E-04

cds.Camellia_sinensis.comp32345_c0_seq1_m.37112	PREDICTED: proteasome subunit alpha type-2-A [Daucus carota subsp. sativus]	25.65	87.343	46	9	18	9	1.002	0.975	0.992	1.01	1.021	1.002	1.022	9.14E-02
cds.Camellia_sinensis.comp32347_c1_seq1_m.26020	PREDICTED: phosphoribulokinase, chloroplastic-like [Populus	46.592	236.37	47.6	18	105	17	1.02	1.02	1	0.979	1	0.978	0.973	4.76E-02
cds.Camellia_sinensis.comp32348_c0_seq1_m.12333	PREDICTED: flowering time control protein FCA isoform X3 [Vitis vinifera]	65.426	6.3868	3.1	2	2	2	1.011	1	0.995	0.996	1.037	0.969	0.999	9.37E-01
cds.Camellia_sinensis.comp32356_c0_seq5_m.37866	PREDICTED: 50S ribosomal protein HLP, mitochondrial isoform X3 [Nelumbo	13.333	1.8762	8.1	1	1	1	0.887	1.035	1.109	1.084	0.889	1.019	0.987	8.98E-01

cds.Camellia_sinensis.comp32360_c0_seq1_m.22186	PREDICTED: 30S ribosomal protein S1-like [Nicotiana tabacum]	46.85	41.834	15.2	5	7	5	0.992	1.057	0.939	1.001	1.055	0.968	1.012	7.82E-01
cds.Camellia_sinensis.comp32362_c0_seq1_m.5696	PREDICTED: alpha-xylosidase 1-like [Juglans regia]	102.92	96.953	21	18	27	16	0.97	0.92	0.93	1.066	1.08	1.046	1.132	2.64E-03
cds.Camellia_sinensis.comp32362_c0_seq2_m.6011	PREDICTED: alpha-xylosidase 1-like [Juglans regia]	103	20.166	10.2	9	13	7	1.017	1.009	1.028	1.01	0.951	1.002	0.97	1.94E-01
cds.Camellia_sinensis.comp32367_c0_seq1_m.5385	PREDICTED: protein translocase subunit SecA, chloroplastic [Vitis vinifera]	107.75	232.29	41.8	35	48	35	1.002	1.017	1.01	0.994	0.977	0.992	0.978	3.36E-02
cds.Camellia_sinensis.comp32368_c0_seq1_m.6891	PREDICTED: linoleate 13S-lipoxygenase 3-1, chloroplastic [Nicotiana tabacum]	102.78	21.33	9.8	8	9	8	0.952	0.997	1.062	1.009	0.98	1.023	1	9.74E-01

cds.Camellia_sinensis.comp32370_c0_seq3_m.44534	PREDICTED: superoxide dismutase [Fe] 3, chloroplastic [Juglans regia]	27.666	1.2639	3.3	1	1	1	0.897	0.99	0.959	1.069	1.065	1.053	1.12	6.06E-02
cds.Camellia_sinensis.comp32371_c0_seq1_m.8400	PREDICTED: nucleolin 2 [Prunus mume]	79.316	3.9865	2	1	1	1	1.251	1.248	1.228	0.744	0.722	0.735	0.591	4.70E-07
cds.Camellia_sinensis.comp32375_c0_seq2_m.21212	PREDICTED: oxysterol-binding protein-related protein 3C-like [Nelumbo nucifera]	51.934	11.64	9.9	4	4	3	1.034	1.021	1.122	1.041	0.847	0.933	0.888	1.42E-01
cds.Camellia_sinensis.comp32378_c0_seq1_m.4555	PREDICTED: phosphatidylinositol 4-phosphate 5-kinase 9-like [Sesamum indicum]	92.934	1.9421	1	1	1	1	1.22	1.184	1.215	0.769	0.804	0.747	0.641	4.23E-05
cds.Camellia_sinensis.comp32382_c0_seq1_m.25543	PREDICTED: 60S ribosomal protein L7-2-like [Ipomoea nil]	28.589	51.596	44.7	10	14	10	1.139	1.172	1.166	0.796	0.863	0.82	0.713	1.78E-04

cds.Camellia_sinensis.comp32384_c0_seq1_m.22206	PREDICTED: sucrose-phosphatase 2-like [Sesamum indicum]	48.112	162.33	31.6	12	24	12	1.037	1.053	0.985	0.955	0.963	0.961	0.936	8.45E-02
cds.Camellia_sinensis.comp32386_c0_seq1_m.26881	PREDICTED: translation initiation factor eIF-2B subunit beta-like [Nicotiana tabacum]	44.049	6.7722	6.7	3	3	2	0.934	1.001	1.034	0.992	1.041	1.016	1.027	4.56E-01
cds.Camellia_sinensis.comp32387_c0_seq1_m.7300	PREDICTED: eukaryotic translation initiation factor [Vitis vinifera]	87.118	109.52	29.4	23	32	23	0.961	0.96	0.949	1.038	1.055	1.073	1.103	7.01E-04
cds.Camellia_sinensis.comp32388_c0_seq1_m.31024	PREDICTED: perakine reductase-like [Populus euphratica]	30.224	5.9833	7.4	2	3	1								

cds.Camellia_sinensis.comp32393_c0_seq1_m.22641	PREDICTED: probable N-acetyl-gamma-glutamyl-phosphate reductase, chloroplastic [Vitis vinifera]	44.074	49.966	23.1	9	11	9	0.979	1.021	0.954	0.971	0.998	1.054	1.023	5.03E-01
cds.Camellia_sinensis.comp32394_c0_seq74_m.10219	PREDICTED: probable acyl-activating enzyme 18, peroxisomal [Ziziphus	81.183	52.494	14.9	9	10	9	1.289	1.404	1.323	0.588	0.58	0.607	0.442	4.53E-06
cds.Camellia_sinensis.comp32394_c0_seq94_m.12860	PREDICTED: 15.7 kDa heat shock protein, peroxisomal [Solanum tuberosum	18.83	5.4027	22.5	4	4	4	1.029	1.041	1.035	0.945	0.941	1.004	0.931	7.65E-02
cds.Camellia_sinensis.comp32397_c0_seq17_m.21879	PREDICTED: V-type proton ATPase subunit d2-like [Daucus carota subsp.	40.616	10.037	15.1	6	8	6	0.987	1.007	0.965	0.985	1.045	1.018	1.03	2.34E-01

cds.Camellia_sinensis.comp32401_c0_seq1_m.44336	PREDICTED: uroporphyrinogen decarboxylase, chloroplastic [Ipomoea nil]	21.735	14.659	23.2	3	6	3	1.009	0.978	0.893	1.062	1.023	1.042	1.086	9.41E-02
cds.Camellia_sinensis.comp32402_c0_seq1_m.10103	PREDICTED: zeaxanthin epoxidase, chloroplastic [Juglans regia]	73.508	66.786	27.4	18	32	18	1.015	0.997	1.005	0.979	1	1.004	0.989	2.92E-01
cds.Camellia_sinensis.comp32410_c0_seq1_m.12637	PREDICTED: protein TOPLESS-like, partial [Juglans regia]	48.81	27.386	15	6	7	5	0.903	0.905	0.938	1.074	1.108	1.074	1.186	4.56E-04
cds.Camellia_sinensis.comp32413_c0_seq1_m.18086	P-loop nucleoside triphosphate hydrolase superfamily protein [Medicago truncatula]	57.784	12.969	10.1	5	6	5	1.01	1.027	1.059	0.983	0.948	0.97	0.937	2.04E-02

cds.Camellia_sinensis.comp32419_c0_seq1_m.20477	PREDICTED: serine/threonine-protein kinase SRK2A [Vitis vinifera]	41.231	4.688	14	5	6	3	1.09	1.077	1.077	0.917	0.909	0.911	0.844	1.88E-06
cds.Camellia_sinensis.comp32429_c0_seq3_m.4590	PREDICTED: putative late blight resistance protein homolog R1B-14 [Solanum pennellii]	96.321	7.4915	5.7	5	6	5	0.957	0.96	1.03	1.042	1.049	0.973	1.04	3.15E-01
cds.Camellia_sinensis.comp32432_c0_seq1_m.18622	PREDICTED: calcium-dependent protein kinase 1-like [Nelumbo nucifera]	57.26	45.757	23.2	10	12	6	1.045	0.991	0.994	0.949	1.067	1.02	1.002	9.78E-01

cds.Camellia_sinensis.comp32434_c0_seq1_m.25833	PREDICTED: 4-diphosphocytidyl-2-C-methyl-D-erythritol kinase, chloroplastic/chromoplastic [Nicotiana attenuata]	45.215	21.832	11.5	4	6	4	1.005	1.017	1.029	1.034	0.972	1.019	0.991	6.79E-01
cds.Camellia_sinensis.comp32434_c0_seq3_m.38079	PREDICTED: 4-diphosphocytidyl-2-C-methyl-D-erythritol kinase, chloroplastic/chromoplastic isoform X2[Vitis vinifera]	28.668	3.2328	8.4	2	4	2	1.011	0.979	1.027	1.09	0.986	0.923	0.994	8.80E-01
cds.Camellia_sinensis.comp32437_c0_seq1_m.8865	PREDICTED: acyl-CoA dehydrogenase family member 10 [Jatropha	92.395	40.355	15.3	12	19	12	0.955	1.01	0.96	1.005	1.036	1.01	1.043	1.04E-01

cds.Camellia_sinensis.comp32440_c0_seq1_m.1590	PREDICTED: cleavage and polyadenylation specificity factor subunit 1 isoform X1 [Theobroma cacao]	164.58	12.974	4.5	6	6	6	0.954	0.963	0.953	1.087	1.035	1.035	1.1	4.68E-03
cds.Camellia_sinensis.comp32443_c0_seq1_m.34125	PREDICTED: peptide methionine sulfoxide reductase A1 [Ricinus communis]	29.217	39.908	21.9	7	22	7	1.027	1.02	0.984	0.963	0.986	1.012	0.977	2.97E-01
cds.Camellia_sinensis.comp32444_c0_seq1_m.4295	PREDICTED: multiple C2 and transmembrane domain-containing protein 1-like [Jatropha curcas]	116.26	70.831	16.4	15	23	15	0.957	0.971	0.982	1.042	1.039	1.028	1.068	1.48E-03

cds.Camellia_sinensis.comp32448_c0_seq2_m.29251	PREDICTED: 3-hydroxyisobutyryl-CoA hydrolase-like protein 3, mitochondrial isoform X1 [Juglans	32.919	23.432	24.5	7	12	7	1.008	0.958	1.016	1.01	0.955	1.026	1.003	9.24E-01
cds.Camellia_sinensis.comp32451_c0_seq1_m.37243	phosphoglycerate mutase family protein [Medicago truncatula]	30.907	12.848	13.6	3	3	3	1.068	0.956	1	1.01	1.01	0.959	0.985	7.16E-01
cds.Camellia_sinensis.comp32453_c0_seq1_m.12949	PREDICTED: lysine--tRNA ligase, cytoplasmic-like [Gossypium hirsutum]	69.931	94.876	37.2	25	36	25	0.965	0.953	0.947	1.038	1.037	1.042	1.088	1.22E-04
cds.Camellia_sinensis.comp32458_c0_seq1_m.14581	PREDICTED: probable galactinol--sucrose galactosyltransferase 2 [Eucalyptus grandis]	64.87	86.998	19.5	12	20	12	1.139	1.189	1.21	0.835	0.79	0.795	0.684	1.15E-04

cds.Camellia_sinensis.comp32458_c1_seq1_m.45032	PREDICTED: probable galactinol--sucrose galactosyltransferase 2 [Citrus sinensis]	18.413	46.075	30.8	3	6	2	1.227	1.145	1.162	0.755	0.835	0.805	0.678	4.21E-04
cds.Camellia_sinensis.comp32458_c1_seq2_m.46697	PREDICTED: probable galactinol--sucrose galactosyltransferase 2 [Sesamum indicum]	18.472	1.2074	20.1	2	4	1	1.028	1.013	0.985	1.112	0.727	1.138	0.984	8.27E-01
cds.Camellia_sinensis.comp32459_c1_seq1_m.24665	PREDICTED: serine carboxypeptidase-like 18 [Jatropha curcas]	52.937	11.819	17.6	6	9	4	0.94	0.965	0.954	1.05	1.027	1.088	1.107	5.32E-03
cds.Camellia_sinensis.comp32460_c0_seq1_m.14509	PREDICTED: reticuline oxidase-like protein [Eucalyptus grandis]	46.143	17.56	16.8	8	12	7	1.006	1.009	1.015	0.989	0.941	1.05	0.983	6.35E-01

cds.Camellia_sinensis.comp32467_c0_seq1_m.27579	PREDICTED: 2-methylene-furan-3-one reductase [Theobroma cacao]	42.717	279.91	52.5	19	66	19	1.057	1.017	1.004	0.964	0.964	0.978	0.944	2.40E-02
cds.Camellia_sinensis.comp32471_c0_seq4_m.12264	PREDICTED: NEDD8-activating enzyme E1 regulatory subunit AXR1 isoform X1 [Vitis vinifera]	64.292	42.122	16	8	10	0								
cds.Camellia_sinensis.comp32475_c0_seq1_m.12477	PREDICTED: vacuolar-sorting receptor 3 [Theobroma cacao]	69.884	4.3057	14.4	7	8	1	1.015	0.979	1.027	1.031	1.013	0.947	0.99	7.42E-01
cds.Camellia_sinensis.comp32475_c0_seq2_m.12762	PREDICTED: vacuolar-sorting receptor 3-like [Juglans regia]	69.568	58.183	18	9	10	3	1.016	1.044	1.106	0.998	0.971	0.867	0.896	8.92E-02

cds.Camellia_sinensis.comp32485_c0_seq3_m.14868	PREDICTED: 70 kDa peptidyl-prolyl isomerase-like [Prunus mume]	64.493	1.6608	4.5	3	6	1	1.094	1.105	1.146	0.914	0.829	0.887	0.786	1.68E-03
cds.Camellia_sinensis.comp32494_c0_seq2_m.1383	PREDICTED: protein transport protein SEC16B homolog [Prunus mume]	114.8	3.7918	0.9	1	1	1								
cds.Camellia_sinensis.comp32494_c0_seq2_m.1384	PREDICTED: protein transport protein SEC16B homolog [Solanum tuberosum	29.447	2.1614	3.8	1	1	1	1.053	1.15	1.037	0.957	0.78	1.004	0.846	1.09E-01
cds.Camellia_sinensis.comp32495_c1_seq8_m.32998	PREDICTED: derlin-2.2 [Ipomoea nil]	28.292	4.2017	13.5	3	3	2	0.948	0.95	1.086	1.016	1.099	0.924	1.018	8.05E-01
cds.Camellia_sinensis.comp32495_c1_seq9_m.33127	PREDICTED: derlin-2.2 [Ipomoea nil]	28.151	8.5652	8.2	2	2	1	1.003	0.875	0.927	1.032	1.107	1.079	1.147	3.59E-02

cds.Camellia_sinensis.comp32501_c0_seq3_m.20794	PREDICTED: kelch domain-containing protein 3 isoform X3 [Vitis vinifera]	57.433	3.2848	1.9	1	1	1	1.087	0.824	1.134	0.947	1.06	0.948	0.97	8.49E-01
cds.Camellia_sinensis.comp32502_c1_seq1_m.40785	PREDICTED: glutamyl-tRNA(Gln) amidotransferase subunit C, chloroplastic/mitochondrial-like [Vitis	17.402	11.111	9.2	1	3	1	1.059	1.001	1.004	0.992	0.994	0.95	0.958	1.45E-01
cds.Camellia_sinensis.comp32506_c0_seq2_m.22896	PREDICTED: peptide chain release factor PrfB1, chloroplastic [Sesamum indicum]	27.069	11.607	22.2	6	9	6	0.987	1.028	1.067	1.025	0.99	0.95	0.962	2.86E-01

cds.Camellia_sinensis.comp32506_c0_seq2_m.22897	PREDICTED: peptide chain release factor PrfB1, chloroplastic [Vitis vinifera]	17.521	14.814	26.8	3	4	3	0.873	1.122	0.938	1.061	1.084	0.952	1.056	5.28E-01
cds.Camellia_sinensis.comp32513_c0_seq4_m.40033	PREDICTED: putative F-box protein At3g23950 [Daucus carota subsp. sativus]	30.028	2.1194	3.4	1	1	1	0.895	1.058	1.032	1.031	1.004	1.003	1.018	7.22E-01
cds.Camellia_sinensis.comp32522_c2_seq1_m.14114	PREDICTED: peptide-N4-(N-acetyl-beta-glucosaminyl)asparagine amidase A-like [Nicotiana	71.676	20.114	6.4	4	9	3	0.964	1.002	1.043	1.006	1.041	0.979	1.006	8.50E-01

cds.Camellia_sinensis.comp32522_c2_seq3_m.15766	PREDICTED: peptide-N4-(N-acetyl-beta-glucosaminyl)asparagine amidase A [Prunus	65.729	17.41	6.6	4	5	1	1.027	1.06	1.009	1.061	0.896	0.95	0.939	2.75E-01
cds.Camellia_sinensis.comp32523_c0_seq2_m.19658	PREDICTED: tubulin beta-1 chain [Ipomoea nil]	50.754	48.789	30.2	9	19	2	1.252	1.157	1.144	0.734	0.777	0.869	0.67	2.14E-03
cds.Camellia_sinensis.comp32523_c0_seq3_m.20537	PREDICTED: tubulin beta-1 chain [Oryza brachyantha]	54.973	54.033	29.5	10	23	3	1.169	1.162	1.091	0.838	0.866	0.867	0.751	3.21E-04
cds.Camellia_sinensis.comp32523_c0_seq4_m.21036	PREDICTED: tubulin beta-2 chain [Nelumbo nucifera]	53.579	82.705	36.1	12	24	3								

cds.Camellia_sinensis.comp32523_c1_seq1_m.11627	PREDICTED: WPP domain-interacting tail-anchored protein 1 [Vitis vinifera]	77.162	1.5994	3.6	3	3	3	0.867	1.042	1.107	1.051	0.962	0.998	0.998	9.71E-01
cds.Camellia_sinensis.comp32526_c0_seq1_m.7405	PREDICTED: nuclear pore complex protein NUP85 [Vitis vinifera]	80.37	27.331	8.4	4	5	4	0.952	1.008	1.105	1.148	0.877	0.965	0.976	7.62E-01
cds.Camellia_sinensis.comp32528_c0_seq1_m.20398	PREDICTED: hydroxymethylglutaryl-CoA synthase-like [Gossypium arboreum]	51.699	6.5554	8.9	4	5	3	1.009	1.014	0.913	1.081	1.04	1.026	1.072	1.35E-01
cds.Camellia_sinensis.comp32528_c0_seq2_m.21516	PREDICTED: hydroxymethylglutaryl-CoA synthase [Theobroma cacao]	51.561	21.405	8.1	4	5	3	0.949	0.98	0.939	1.052	1.022	1.121	1.114	2.36E-02

cds.Camellia_sinensis.comp32536_c0_seq3_m.1041	Heterogeneous nuclear ribonucleoprotein Q [Morus notabilis]	90.479	19.33	10.4	8	8	8	0.994	1.047	0.999	0.953	0.95	1.022	0.962	2.52E-01
cds.Camellia_sinensis.comp32537_c0_seq1_m.2620	tolB protein-like protein [Arabidopsis thaliana]	73.827	107.67	25.8	13	18	11	0.93	0.914	0.902	1.133	1.074	1.06	1.19	1.46E-03
cds.Camellia_sinensis.comp32537_c0_seq3_m.13950	tolB protein-like protein [Arabidopsis thaliana]	77.657	55.919	22.6	12	18	10	0.902	0.933	0.928	1.114	1.1	1.066	1.187	5.18E-04
cds.Camellia_sinensis.comp32537_c0_seq5_m.16986	PREDICTED: topless-related protein 1 isoform X1 [Vigna radiata var. radiata]	62.321	20.338	15	8	11	4	0.965	0.974	1.044	0.983	0.957	1.089	1.015	7.73E-01

cds.Camellia_sinensis.comp32538_c0_seq1_m.33174	PREDICTED: probable 2-oxoglutarate-dependent dioxygenase At3g49630 isoform X1 [Theobroma cacao]	37.605	3.4332	10.4	3	3	3	0.984	0.909	0.962	1.131	1.029	1.013	1.111	6.56E-02
cds.Camellia_sinensis.comp32542_c0_seq2_m.8482	PREDICTED: vacuolar protein sorting-associated protein 51 homolog [Vitis vinifera]	89.96	10.538	4	3	3	3								
cds.Camellia_sinensis.comp32546_c0_seq1_m.9615	PREDICTED: heat shock 70 kDa protein, mitochondrial-like [Sesamum indicum]	72.905	306.64	41.2	29	84	23	0.993	0.97	0.969	1.025	1.015	1.017	1.043	8.04E-03

cds.Camellia_sinensis.comp32554_c0_seq1_m.10016	PREDICTED: nuclear pore complex protein NUP62 [Vitis vinifera]	74.947	13.645	7.3	6	6	6	0.929	1.02	1.003	1.014	1.004	0.99	1.019	5.45E-01
cds.Camellia_sinensis.comp32556_c0_seq1_m.17665	PREDICTED: pyridoxine/pyridoxamine 5'-phosphate oxidase 1, chloroplastic [Vitis vinifera]	53.413	25.09	16.8	6	7	6	0.951	1.04	0.976	1.155	0.932	0.969	1.03	7.35E-01
cds.Camellia_sinensis.comp32557_c2_seq3_m.24353	type 2A protein phosphatase-3 [Populus trichocarpa]	35.665	67.883	26.8	6	8	4	0.986	0.972	0.973	1.119	1.126	0.852	1.057	6.61E-01

cds.Camellia_sinensis.comp32557_c2_seq6_m.32119	PREDICTED: probable bifunctional methylthioribulose-1-phosphate dehydratase/enolase-phosphatase E1 1[Nicotiana attenuata]	45.532	33.579	19.6	9	12	9	0.991	0.994	0.989	1.084	0.983	1.027	1.04	3.05E-01
cds.Camellia_sinensis.comp32559_c0_seq1_m.23940	PREDICTED: 30S ribosomal protein S1 isoform X1 [Theobroma cacao]	43.965	7.4792	5.8	2	4	2	0.951	0.99	1.079	0.985	1.059	0.958	0.994	9.14E-01
cds.Camellia_sinensis.comp32561_c0_seq9_m.47671	39S ribosomal protein L53/MRP-L53 [Medicago truncatula]	18.602	-2	6.7	1	1	1								
cds.Camellia_sinensis.comp32565_c0_seq1_m.19595	PREDICTED: chaperone protein dnaJ GFA2, mitochondrial [Vitis vinifera]	44.78	3.3611	4.9	2	2	2	0.988	0.973	0.894	1.045	1.031	1.088	1.108	4.19E-02

cds.Camellia_sinensis.comp32566_c0_seq2_m.24088	PREDICTED: ERBB-3 BINDING PROTEIN 1 [Theobroma cacao]	43.52	52.548	30.6	11	13	8	1.199	1.148	1.055	0.862	0.882	0.855	0.764	2.28E-03
cds.Camellia_sinensis.comp32566_c0_seq3_m.24185	PREDICTED: EBP1 isoform X1 [Solanum tuberosum	19.199	12.455	29.7	5	6	2	1.039	1.412	0.889	0.911	0.806	0.914	0.788	1.94E-01
cds.Camellia_sinensis.comp32567_c0_seq1_m.20559	PREDICTED: casein kinase I isoform delta-like [Ziziphus jujuba]	53.237	3.3561	5.5	3	4	3	0.998	1.074	1.095	0.936	0.901	0.964	0.884	2.35E-02
cds.Camellia_sinensis.comp32575_c0_seq1_m.12281	PREDICTED: 2-isopropyl malate synthase A [Vitis vinifera]	66.102	92.444	29.2	15	23	11	0.99	0.962	0.961	1.035	1.012	0.991	1.043	5.75E-02
cds.Camellia_sinensis.comp32575_c0_seq3_m.14832	PREDICTED: 2-isopropyl malate synthase A [Vitis vinifera]	65.888	6.956	15.3	9	11	5	0.923	1.057	0.958	1.022	1.02	1.041	1.049	2.91E-01

cds.Camellia_sinensis.comp32577_c0_seq1_m.48543	PREDICTED: pentatricopeptide repeat-containing protein At4g38150-like [Malus domestica]	22.736	8.6434	22.2	5	5	5	0.996	1.065	1.074	0.95	0.94	0.973	0.913	2.53E-02
cds.Camellia_sinensis.comp32577_c1_seq1_m.18933	PREDICTED: argininosuccinate lyase, chloroplastic [Vitis vinifera]	59.026	22.933	14.3	7	8	7	1.013	0.98	0.94	1.024	1.053	0.994	1.047	1.66E-01
cds.Camellia_sinensis.comp32578_c0_seq1_m.37624	PREDICTED: vacuolar protein sorting-associated protein 26A [Ziziphus jujuba]	25.996	27.362	13.1	3	3	2	0.913	0.948	1.042	1.026	1.048	1.049	1.076	1.36E-01
cds.Camellia_sinensis.comp32581_c1_seq3_m.881	PREDICTED: heat shock protein 90-5, chloroplastic [Ziziphus jujuba]	91.123	323.31	41.9	26	55	22	1.194	1.155	1.172	0.807	0.787	0.827	0.688	2.33E-05

cds.Camellia_sinensis.comp32581_c1_seq51_m.2176	PREDICTED: trafficking protein particle complex II-specific subunit 130 homolog [Vitis vinifera]	140.11	8.0215	4.3	5	5	5	0.959	1.078	0.976	1.009	0.967	1.023	0.995	9.33E-01
cds.Camellia_sinensis.comp32581_c1_seq91_m.24298	PREDICTED: probable protein phosphatase 2C 60 [Nelumbo nucifera]	49.565	3.2527	5.1	2	2	2	0.96	1.001	1.034	0.985	1.031	1.004	1.008	7.51E-01
cds.Camellia_sinensis.comp32585_c0_seq1_m.4306	PREDICTED: 5-methyltetrahydropteroyltriglutamate--homocysteine methyltransferase [Ziziphus jujuba]	71.439	46.35	26.5	17	46	11	0.985	0.973	0.987	1.022	1.027	1.017	1.041	1.58E-03

cds.Camellia_sinensis.comp32586_c0_seq1_m.27262	PREDICTED: triosephosphate isomerase, chloroplastic [Solanum pennellii]	34.474	155.35	37.8	11	40	11	1.054	0.998	1.036	0.948	0.971	0.969	0.935	2.01E-02
cds.Camellia_sinensis.comp325875_c0_seq1_m.52540	PREDICTED: trans-resveratrol di-O-methyltransferase-like [Capsicum annuum]	18.308	3.3564	6.7	1	1	1	0.827	0.94	0.8	1.074	1.207	1.205	1.358	8.00E-03
cds.Camellia_sinensis.comp32591_c0_seq1_m.18528	PREDICTED: anthranilate phosphoribosyltransferase, chloroplastic [Jatropha curcas]	44.465	35.223	17	6	11	6	1.007	0.99	1.044	0.94	1.022	1.044	0.988	7.47E-01
cds.Camellia_sinensis.comp32592_c3_seq4_m.29222	PREDICTED: arogenate dehydratase 3, chloroplastic [Vitis vinifera]	45.948	3.0291	4.3	2	2	1	1.331	1.137	1.271	0.663	0.842	0.675	0.583	3.78E-03

cds.Camellia_sinensis.comp32594_c0_seq1_m.2005	PREDICTED: translocase of chloroplast 159, chloroplastic [Nicotiana attenuata]	145.19	169.24	17.4	19	26	19	1.093	1.091	1.089	0.9	0.863	0.894	0.812	3.90E-03
cds.Camellia_sinensis.comp32595_c0_seq1_m.5552	PREDICTED: tubulin--tyrosine ligase-like protein 12 isoform X2 [Vitis vinifera]	102.33	1.3778	0.9	1	1	1	1.037	0.888	1.004	0.848	1.055	1.167	1.048	7.20E-01
cds.Camellia_sinensis.comp32596_c0_seq2_m.1710	PREDICTED: CLIP-associated protein isoform X1 [Vitis vinifera]	161.78	3.2754	1.6	2	2	2	0.835	1.039	1.221	1.007	1.037	0.888	0.947	7.30E-01
cds.Camellia_sinensis.comp32598_c0_seq1_m.32571	PREDICTED: nifU-like protein 4, mitochondrial [Citrus sinensis]	20.076	20.747	38.9	6	9	6	0.963	1.038	0.964	0.988	1.013	0.964	1	9.89E-01

cds.Camellia_sinensis.comp32599_c0_seq1_m.31879	PREDICTED: gamma carbonic anhydrase 1, mitochondrial [Ziziphus jujuba]	29.459	79.989	38.4	10	17	4	1.018	1.026	0.977	0.992	0.976	0.986	0.978	2.35E-01
cds.Camellia_sinensis.comp32599_c0_seq3_m.34004	PREDICTED: gamma carbonic anhydrase 1, mitochondrial-like [Gossypium hirsutum]	29.493	23.55	32.8	9	16	3	0.987	0.959	0.931	1.048	1.042	1.008	1.077	2.34E-02
cds.Camellia_sinensis.comp32604_c0_seq2_m.38380	PREDICTED: ER membrane protein complex subunit 4-like [Gossypium raimondii]	19.751	3.5857	17.8	3	4	3	0.998	0.908	1.104	0.93	1.013	1.053	0.995	9.69E-01
cds.Camellia_sinensis.comp32608_c0_seq2_m.39213	PREDICTED: proteasome subunit alpha type-7 [Vitis vinifera]	27.457	64.161	48.8	13	26	13	1.003	0.967	0.963	1.011	1.025	1.043	1.05	3.67E-02

cds.Camellia_sinensis.comp32611_c0_seq2_m.7606	PREDICTED: 26S proteasome non-ATPase regulatory subunit 8 homolog A [Sesamum	26.899	75.183	45.3	9	14	9	1.012	0.992	0.996	0.954	0.984	1.015	0.984	4.43E-01
cds.Camellia_sinensis.comp32611_c0_seq5_m.9822	PREDICTED: eukaryotic translation initiation factor 5B isoform X2 [Prunus	49.752	2.1091	4.1	1	1	1	0.883	0.94	1.151	1.074	1.077	0.91	1.029	7.57E-01
cds.Camellia_sinensis.comp32612_c0_seq1_m.44258	PREDICTED: small ubiquitin-related modifier 1 [Eucalyptus grandis]	11.644	63.404	33.3	4	8	1	1.095	1.016	1.17	0.962	0.784	0.954	0.823	6.51E-02
cds.Camellia_sinensis.comp32612_c1_seq1_m.15867	transaldolase family protein [Populus trichocarpa]	48.347	172.31	32.3	13	33	13	1.061	1.018	1.054	0.946	0.961	0.966	0.917	3.76E-03

cds.Camellia_sinensis.comp32613_c0_seq1_m.19293	PREDICTED: phenylalanine--tRNA ligase alpha subunit, cytoplasmic [Vitis vinifera]	59.46	7.2012	8.2	5	6	5	0.961	0.985	0.953	1.038	1.038	1.011	1.065	9.00E-03
cds.Camellia_sinensis.comp32614_c0_seq24_m.32059	plastid transcriptionally active 6 [Arabidopsis thaliana]	39.691	1.67	4.9	2	2	2								
cds.Camellia_sinensis.comp32619_c1_seq1_m.30469	Tetratricopeptide repeat (TPR)-like superfamily protein [Arabidopsis thaliana]	21.115	2.1993	6.2	1	1	1								
cds.Camellia_sinensis.comp32624_c0_seq18_m.48943	PREDICTED: organellar oligopeptidase A, chloroplastic/mitochondrial [Vitis vinifera]	20.667	1.6392	11.2	2	2	2								

cds.Camellia_sinensis.comp32624_c0_seq1_m.4750	PREDICTED: probable ADP-ribosylation factor GTPase-activating protein AGD14 isoform X2 [Theobroma cacao]	34.441	2.1163	2.9	1	1	1	1.061	1.021	1.007	1.042	0.96	0.91	0.943	2.28E-01
cds.Camellia_sinensis.comp32625_c0_seq1_m.1156	ribulose-1 (chloroplast) [Carpinus putoensis]	53.444	323.31	55.6	31	631	31	1	1.022	1.005	0.992	0.971	0.971	0.969	3.24E-02
cds.Camellia_sinensis.comp32625_c0_seq1_m.1157	ATP synthase CF1 beta subunit (chloroplast) [Camellia leptophylla]	49.355	323.31	72.1	22	234	21	1.005	1.007	0.995	1.001	1.012	0.988	0.998	8.09E-01
cds.Camellia_sinensis.comp32625_c0_seq1_m.1158	ATP synthase CF1 epsilon subunit (chloroplast) [Camellia leptophylla]	14.685	149.91	66.9	7	28	7	0.992	0.98	0.977	1.056	0.995	1.015	1.04	9.97E-02

cds.Camellia_sinensis.comp32626_c0_seq3_m.34857	Thioredoxin-like protein [Morus notabilis]	28.957	87.929	42.5	11	20	11	1.013	1.035	0.985	0.992	0.988	0.985	0.978	2.61E-01
cds.Camellia_sinensis.comp32626_c2_seq1_m.40398	TATA BINDING protein ASSOCIATED FACTOR 21KDA SUBUNIT [Populus trichocarpa]	23.413	1.9244	4.8	1	2	1	1.021	0.954	1.054	0.961	0.98	1.034	0.982	6.56E-01
cds.Camellia_sinensis.comp32626_c3_seq1_m.19050	PREDICTED: FRIGIDA-like protein 4a [Nicotiana tabacum]	63.348	24.606	14.5	8	13	8	1.011	1.075	1.095	0.942	0.92	0.906	0.87	6.52E-03
cds.Camellia_sinensis.comp32628_c0_seq6_m.1529	PREDICTED: chromatin modification-related protein eaf-1 [Vitis vinifera]	102.17	17.366	9.1	8	8	8	1.033	1.073	1.076	0.932	0.897	0.962	0.877	5.52E-03
cds.Camellia_sinensis.comp32629_c0_seq1_m.13549	PREDICTED: T-complex protein 1 subunit alpha [Jatropha curcas]	41.847	70.735	33.3	11	18	11	0.955	0.977	0.935	1.051	1.054	1.035	1.095	2.80E-03

cds.Camellia_sinensis.comp32629_c0_seq1_m.13550	PREDICTED: T-complex protein 1 subunit alpha [Jatropha curcas]	17.331	9.7863	16.2	3	6	3	0.938	1.002	1.025	1.035	1.017	1.043	1.044	1.90E-01
cds.Camellia_sinensis.comp32632_c0_seq1_m.2349	SH3 domain protein [Medicago truncatula]	116.34	3.2732	2.1	3	3	3	1.072	1.098	1.151	0.881	0.887	0.889	0.8	8.84E-03
cds.Camellia_sinensis.comp32633_c0_seq1_m.37141	PREDICTED: secoisolariciresinol dehydrogenase [Malus domestica]	32.062	11.239	15.1	5	7	5	0.965	0.962	0.996	1.059	1.015	1.017	1.057	3.46E-02
cds.Camellia_sinensis.comp32635_c0_seq1_m.17640	PREDICTED: phospholipase SGR2 isoform X4 [Ziziphus jujuba]	53.979	12.173	8.2	3	3	3	0.926	1.056	1.078	0.999	1.007	0.938	0.962	5.22E-01
cds.Camellia_sinensis.comp32637_c0_seq1_m.12436	PREDICTED: protein MLP1 isoform X2 [Vitis vinifera]	76.674	11.268	7.2	5	6	4	1.065	1.144	1.081	0.882	0.892	0.929	0.822	1.88E-03

cds.Camellia_sinensis.comp32637_c0_seq6_m.41238	PREDICTED: protein MLP1 isoform X2 [Vitis vinifera]	31.347	1.7717	3.9	1	1	1	0.959	0.948	1.147	1.045	0.978	0.94	0.97	7.12E-01
cds.Camellia_sinensis.comp32640_c0_seq1_m.17669	PREDICTED: haloalkane dehalogenase [Theobroma cacao]	44.878	124.04	39.3	12	30	12	1.066	1.059	1.003	0.937	0.945	0.943	0.903	3.49E-02
cds.Camellia_sinensis.comp32640_c0_seq10_c0_seq1_m.17671	PREDICTED: U6 snRNA-associated Sm-like protein LSm3 [Populus euphratica]	16.597	2.7429	12.9	2	3	2	0.871	1.01	0.981	1.179	1.031	0.97	1.111	2.29E-01
cds.Camellia_sinensis.comp32641_c0_seq1_m.1177	PREDICTED: transcription elongation factor SPT6 homolog [Vitis vinifera]	189.38	8.921	0.9	2	2	2	1.068	1.138	1.129	0.893	0.843	0.905	0.792	1.38E-03

cds.Camellia_sinensis.comp32645_c0_seq1_m.1618	PREDICTED: enoyl-[acyl-carrier-protein] reductase [NADH], chloroplastic [Vitis vinifera]	41.544	94.606	27	9	25	3	0.993	0.945	0.972	1.035	1.052	1.015	1.066	2.24E-02
cds.Camellia_sinensis.comp32654_c0_seq1_m.12782	PREDICTED: peptidyl-prolyl cis-trans isomerase PASTICCINO1 isoform X1 [Vitis vinifera]	70.005	10.659	6.1	3	3	3	1.04	1.116	1.044	0.911	0.851	0.864	0.821	3.04E-03
cds.Camellia_sinensis.comp32659_c0_seq6_m.10945	PREDICTED: probable glutamate carboxypeptidase 2 isoform X1 [Sesamum indicum]	39.017	27.07	19	6	9	6	0.946	0.987	0.949	1.033	1.045	1.092	1.1	1.19E-02
cds.Camellia_sinensis.comp32659_c0_seq6_m.10946	PREDICTED: probable glutamate carboxypeptidase 2 [Vitis vinifera]	22.959	16.231	18.2	4	5	4	0.942	0.956	0.966	1.069	1.068	1.029	1.105	2.32E-03

cds.Camellia_sinensis.comp32660_c0_seq1_m.8080	PREDICTED: pyrophosphate-energized vacuolar membrane proton pump [Theobroma cacao]	40.823	29.981	12.9	4	13	2	0.96	1.022	1.056	0.987	1.058	1.032	1.013	7.24E-01
cds.Camellia_sinensis.comp32660_c0_seq1_m.8081	PREDICTED: pyrophosphate-energized vacuolar membrane proton pump 1-like isoform X2 [Nicotiana tomentosiformis]	22.99	3.1325	30.8	6	15	1								
cds.Camellia_sinensis.comp32669_c0_seq1_m.22867	PREDICTED: stomatin-like protein 2, mitochondrial [Capsicum annuum]	44.407	84.708	43.7	16	26	16	0.994	1.043	0.988	0.995	0.991	1.012	0.991	6.59E-01

cds.Camellia_sinensis.comp32675_c0_seq1_m.32022	PREDICTED: DCN1-like protein 1 [Theobroma cacao]	30.045	2.5435	7.4	2	2	2								
cds.Camellia_sinensis.comp32685_c0_seq1_m.26863	PREDICTED: acidic mammalian chitinase [Vitis vinifera]	47.12	24.795	5.4	2	2	2	1.015	1.091	1.018	1.056	0.949	0.875	0.922	2.31E-01
cds.Camellia_sinensis.comp32690_c0_seq1_m.15967	PREDICTED: ectonucleotide pyrophosphatase/phosphodiesterase family member 3-like [Nicotiana tomentosiformis]	55.646	14.376	11	6	7	5	1.039	1.054	1.042	0.974	0.931	0.943	0.908	2.44E-03

cds.Camellia_sinensis.comp32691_c0_seq2_m.17320	PREDICTED: lipoamide acyltransferase component of branched-chain alpha-keto acid dehydrogenase complex,mitochondrial [Vitis vinifera]	62.031	24.979	5.9	2	2	2	0.976	0.967	1.171	0.967	0.964	0.962	0.929	3.80E-01
cds.Camellia_sinensis.comp32694_c0_seq3_m.27655	alpha/beta-hydrolases superfamily protein precursor [Solanum lycopersicum]	22.834	1.7437	4	1	2	1	1.019	1.017	1.02	0.972	0.99	0.984	0.964	2.56E-03
cds.Camellia_sinensis.comp32696_c0_seq3_m.41222	PREDICTED: aladin isoform X1 [Vitis vinifera]	27.84	5.1003	14.9	4	4	4	0.992	0.924	0.958	1.055	1.023	1.086	1.101	2.29E-02
cds.Camellia_sinensis.comp32699_c0_seq1_m.25218	PREDICTED: 26S proteasome non-ATPase regulatory subunit 13 homolog A [Vitis vinifera]	47.41	46.294	22.8	9	16	9	0.961	0.964	0.993	1.031	1.033	0.99	1.047	5.88E-02

cds.Camellia_sinensis.comp32701_c0_seq1_m.13827	PREDICTED: ATPase family AAA domain-containing protein 3-B [Nelumbo nucifera]	74.452	28.859	11.9	9	12	8	0.923	0.975	0.939	1.035	1.042	1.027	1.094	5.72E-03
cds.Camellia_sinensis.comp32702_c0_seq14_m.38973	PREDICTED: profilin-2 [Gossypium hirsutum]	14.14	34.761	38.9	5	19	4	1.041	0.993	0.974	0.99	1.006	1.022	1.003	8.76E-01
cds.Camellia_sinensis.comp32707_c0_seq1_m.33071	PREDICTED: probable diphthine methyl ester synthase [Vitis vinifera]	30.465	6.7062	6.9	2	2	2	0.917	0.959	0.997	1.095	1.017	1.045	1.099	4.35E-02
cds.Camellia_sinensis.comp32709_c0_seq2_m.50782	PREDICTED: (+)-neomenthol dehydrogenase [Vitis vinifera]	17.282	59.453	40.4	7	17	7	0.983	1.005	0.997	1.013	1.008	1.024	1.02	6.68E-02

cds.Camellia_sinensis.comp32709_c3_seq1_m.49004	PREDICTED: (+)-neomenthol dehydrogenase [Theobroma cacao]	17.639	47.85	48.1	7	15	7	0.997	0.979	1	1.011	0.995	0.985	1.005	6.45E-01
cds.Camellia_sinensis.comp32710_c0_seq5_m.19068	PREDICTED: endoplasmic reticulum-Golgi intermediate compartment protein 3 isoform X1 [Juglans regia]	40.331	1.9355	2.3	1	1	1	1.016	0.985	1.082	0.972	1.012	0.936	0.947	2.04E-01
cds.Camellia_sinensis.comp32716_c0_seq1_m.13905	PREDICTED: phosphoglucomutase, chloroplastic-like [Populus	68.529	116.97	29	15	37	15	0.968	0.976	0.97	1.055	1.05	1.022	1.073	2.30E-03
cds.Camellia_sinensis.comp32716_c0_seq2_m.17812	PREDICTED: phosphoglucomutase, cytoplasmic [Daucus carota subsp.	63.336	295.75	42.7	23	62	12	0.957	0.956	0.966	1.033	1.061	1.042	1.089	5.61E-04

cds.Camellia_sinensis.comp32716_c0_seq3_m.17919	PREDICTED: phosphoglucomutase, cytoplasmic [Daucus carota subsp.	63.559	70.773	34.3	18	50	7	1.038	1.026	1.01	0.945	0.993	0.969	0.946	2.67E-02
cds.Camellia_sinensis.comp32716_c3_seq1_m.24652	PREDICTED: desiccation-related protein At2g46140 [Ziziphus	34.969	57.56	30.8	11	18	11	1.162	1.127	1.143	0.854	0.848	0.842	0.741	3.33E-06
cds.Camellia_sinensis.comp32717_c0_seq1_m.34599	PREDICTED: glutathione S-transferase U17-like [Malus domestica]	25.892	29.821	32.2	7	15	7	1.337	1.307	1.346	0.645	0.642	0.635	0.482	1.07E-07
cds.Camellia_sinensis.comp32721_c2_seq17_m.22610	PREDICTED: multiple inositol polyphosphate phosphatase 1 [Vitis vinifera]	57.393	5.2379	8.9	5	6	2	1.016	0.9	0.964	1.147	0.985	1.011	1.091	2.15E-01

cds.Camellia_sinensis.comp32721_c3_seq1_m.7620	PREDICTED: DNA-binding protein SMUBP-2 [Theobroma cacao]	97.559	4.3756	3.9	4	4	4	1.046	1.138	1.098	0.916	0.933	0.853	0.823	6.06E-03
cds.Camellia_sinensis.comp32724_c0_seq9_m.38007	PREDICTED: PHD finger-like domain-containing protein 5B [Brassica oleracea var. oleracea]	12.414	2.0466	8.2	1	1	1								
cds.Camellia_sinensis.comp32724_c1_seq1_m.8232	PREDICTED: importin subunit beta-1 [Vitis vinifera]	91.958	9.8643	3.4	2	3	2								
cds.Camellia_sinensis.comp32730_c0_seq1_m.23155	PREDICTED: ADP,ATP carrier protein, mitochondrial-like [Ipomoea nil]	49.552	68.861	20.8	11	52	6	1.023	0.997	1.007	1.002	1	1.018	0.998	8.19E-01

cds.Camellia_sinensis.comp32730_c0_seq2_m.54520	PREDICTED: ADP,ATP carrier protein 1, mitochondrial, partial [Cucumis melo]	15.292	28.758	28.9	4	30	2	1.006	0.999	0.985	1.042	0.998	0.966	1.005	8.41E-01
cds.Camellia_sinensis.comp32732_c0_seq5_m.39027	PREDICTED: thiosulfate sulfurtransferase 16, chloroplastic-like [Nicotiana tabacum]	20.187	20.809	13.9	2	4	2	0.708	0.694	0.783	1.411	1.215	1.441	1.861	6.96E-04
cds.Camellia_sinensis.comp32733_c0_seq2_m.7870	PREDICTED: nucleolin 2 isoform X2 [Populus euphratica]	43.038	2.5127	4.2	2	2	2								
cds.Camellia_sinensis.comp32733_c0_seq2_m.7871	PREDICTED: nucleolin 2 isoform X5 [Theobroma cacao]	37.571	11.486	14.8	5	5	5	1.064	1.012	1.042	0.982	0.947	0.949	0.923	1.28E-02
cds.Camellia_sinensis.comp32734_c0_seq1_m.19033	PREDICTED: serine/threonine-protein kinase rio1 [Theobroma cacao]	55.265	-2	1.8	1	1	1	1.053	1.091	1.133	0.923	0.905	0.88	0.826	1.66E-03

cds.Camellia_sinensis.comp32736_c2_seq9_m.26887	PREDICTED: ATP-dependent Clp protease proteolytic subunit-related protein 3, chloroplastic [Daucus carota subsp. sativus]	36.507	21.394	18.6	6	11	6	0.995	1.011	1.016	0.987	0.968	0.965	0.966	2.21E-02
cds.Camellia_sinensis.comp32737_c1_seq27_m.36809	PREDICTED: putative pterin-4-alpha-carbinolamine dehydratase [Theobroma cacao]	21.022	2.0391	8.6	2	2	2	0.984	0.976	1.038	0.926	1.052	1.031	1.004	9.58E-01
cds.Camellia_sinensis.comp32739_c0_seq3_m.16599	PREDICTED: protein disulfide-isomerase [Nelumbo nucifera]	59.466	136.03	39.8	21	52	18	1.023	0.994	1.017	0.972	0.985	0.993	0.972	5.97E-02
cds.Camellia_sinensis.comp32739_c0_seq5_m.46232	PREDICTED: protein disulfide-isomerase [Nelumbo nucifera]	11.563	58.865	46.1	5	10	4	0.939	0.987	0.981	1.022	1.079	1.039	1.08	2.65E-02

cds.Camellia_sinensis.comp32742_c1_seq1_m.45933	PREDICTED: MLP-like protein 329 [Malus domestica]	15.84	14.108	29.2	6	19	6	1.096	1.026	1.02	0.923	0.96	0.956	0.904	1.82E-02
cds.Camellia_sinensis.comp32743_c0_seq1_m.18298	PREDICTED: pheophorbide a oxygenase, chloroplastic [Citrus sinensis]	64.511	31.615	17	10	16	9	0.959	1.004	0.983	1.025	1.03	1.004	1.038	7.03E-02
cds.Camellia_sinensis.comp32746_c0_seq4_m.1832	PREDICTED: cell division cycle and apoptosis regulator protein 1 [Citrus sinensis]	91.223	21.677	3.3	2	2	2	1.115	1.082	0.999	0.971	0.852	0.963	0.872	6.13E-02
cds.Camellia_sinensis.comp32746_c0_seq4_m.1833	PREDICTED: cell division cycle and apoptosis regulator protein 1-like [Juglans regia]	73.066	17.17	6.1	3	3	3	0.897	0.978	0.977	1.069	1.05	1.061	1.115	1.97E-02

cds.Camellia_sinensis.comp32750_c0_seq1_m.2788	PREDICTED: heat shock 70 kDa protein 14-like [Sesamum indicum]	93.302	139	33	29	58	19	1.054	1.044	1.031	0.947	0.968	0.976	0.924	2.00E-03
cds.Camellia_sinensis.comp32750_c0_seq4_m.29864	PREDICTED: heat shock 70 kDa protein 14-like [Sesamum indicum]	39.488	36.472	29.4	11	23	6	1.041	1.012	1.026	1.021	0.958	0.935	0.946	1.13E-01
cds.Camellia_sinensis.comp32759_c0_seq3_m.33575	PREDICTED: erlin-2-B [Juglans regia]	26.825	10.511	22.8	6	7	6	0.98	0.992	0.996	0.983	1.023	1.02	1.02	2.34E-01
cds.Camellia_sinensis.comp32762_c0_seq1_m.10285	PREDICTED: ABC transporter G family member 11 [Ricinus	79.525	7.4286	2.7	2	2	2	1.174	1.224	1.243	0.792	0.761	0.749	0.632	4.14E-05
cds.Camellia_sinensis.comp32762_c0_seq5_m.23908	carboxyl-terminal peptidase, putative (DUF239) [Arabidopsis thaliana]	46.737	12.397	7.1	2	2	2	0.969	1.075	0.98	0.999	1.041	0.948	0.988	7.99E-01

cds.Camellia_sinensis.comp32762_c1_seq3_m.42855	polyketide cyclase/dehydrase and lipid transporter [Medicago truncatula]	20.26	3.243	9.5	2	5	2	1.011	1.035	1.009	1.045	0.944	0.964	0.967	3.38E-01
cds.Camellia_sinensis.comp32765_c1_seq2_m.35449	RAP release 2, galactose-binding-like domain protein, putative (DUF1997) [Arabidopsis thaliana]	30.745	19.69	6.9	2	2	2	0.997	0.985	0.947	1.05	0.973	1.063	1.054	1.79E-01
cds.Camellia_sinensis.comp32769_c0_seq1_m.42882	PREDICTED: microtubule-associated protein 70-2 [Vitis vinifera]	24.68	3.264	4.9	1	1	1	0.977	1.006	1.07	1	1.003	0.955	0.969	3.74E-01
cds.Camellia_sinensis.comp32769_c1_seq2_m.20869	PREDICTED: microtubule-associated protein 70-2-like isoform X1 [Jatropha curcas]	45.149	3.4536	6.7	2	2	2	1.013	0.982	1.004	1.019	1	0.992	1.004	7.58E-01

cds.Camellia_sinensis.comp32771_c0_seq3_m.18310	PREDICTED: protein NRT1/ PTR FAMILY 8.3-like [Nicotiana tabacum]	64.9	2.7966	3.1	2	2	2	1.13	1.079	1.049	0.929	0.84	0.948	0.834	1.35E-02
cds.Camellia_sinensis.comp32800_c0_seq1_m.3897	PREDICTED: phospholipase A-2-activating protein [Prunus mume]	84.033	86.378	22.6	14	19	14	0.982	0.973	0.977	1.022	1.016	1.007	1.039	1.72E-03
cds.Camellia_sinensis.comp32800_c0_seq1_m.3898	PREDICTED: succinate dehydrogenase [ubiquinone] flavoprotein subunit 1, mitochondrial [Juglans regia]	41.711	150.34	27.6	9	18	9	1.011	0.992	1.011	1.021	1.001	0.992	1	9.98E-01

cds.Camellia_sinensis.comp32800_c0_seq3_m.35099	PREDICTED: LOW QUALITY PROTEIN: succinate dehydrogenase [ubiquinone] flavoprotein subunit 1, mitochondrial-like[Sesamum indicum]	28.478	7.2209	25.7	6	14	3	1.026	0.927	0.997	0.967	1.032	1.06	1.037	4.21E-01
cds.Camellia_sinensis.comp32800_c0_seq5_m.39195	PREDICTED: LOW QUALITY PROTEIN: succinate dehydrogenase [ubiquinone] flavoprotein subunit 1, mitochondrial-like[Sesamum indicum]	28.503	82.298	34	7	20	4	1.021	1.039	1.024	0.954	1.007	0.982	0.954	4.67E-02

cds.Camellia_sinensis.comp32801_c0_seq3_m.30883	PREDICTED: V-type proton ATPase subunit D [Theobrom	29.117	57.583	48.7	14	21	14	0.955	0.986	0.972	1.06	1.05	1.033	1.079	3.10E-03
cds.Camellia_sinensis.comp32802_c0_seq1_m.10798	PREDICTED: endoglucanase 25-like [Solanum	68.121	1.4024	1.1	1	1	1								
cds.Camellia_sinensis.comp32806_c0_seq1_m.28386	PREDICTED: beta-glucosidase BoGH3B [Theobroma cacao]	46.413	22.959	8.8	3	4	3	0.903	0.981	0.907	1.103	1.071	1.039	1.151	1.15E-02
cds.Camellia_sinensis.comp32806_c0_seq3_m.55090	PREDICTED: beta-glucosidase BoGH3B [Theobroma cacao]	10.718	3.3033	11.1	1	3	1	0.868	0.925	0.97	1.043	1.103	1.13	1.186	1.29E-02
cds.Camellia_sinensis.comp32809_c0_seq9_m.17983	PREDICTED: eukaryotic translation initiation factor 6-2 [Juglans regia]	26.483	24.118	18	4	6	4	0.967	0.914	0.958	1.065	1.032	1.114	1.131	1.21E-02

cds.Camellia_sinensis.comp32812_c0_seq1_m.9893	PREDICTED: acylamino-acid-releasing enzyme isoform X2 [Juglans regia]	90.165	101.05	20.3	13	27	13	0.951	0.959	0.952	1.065	1.038	1.077	1.111	7.21E-04
cds.Camellia_sinensis.comp32814_c0_seq17_m.3754	RanBP1 domain protein [Medicago truncatula]	29.525	4.9083	12.4	3	3	3	1.06	1.048	0.954	0.964	1.019	0.953	0.959	3.52E-01
cds.Camellia_sinensis.comp32818_c0_seq3_m.31143	PREDICTED: short-chain type dehydrogenase/reductase isoform X2 [Jatropha curcas]	27.946	6.6083	9	2	3	2	0.92	0.974	0.922	1.123	1.068	1.03	1.144	1.27E-02
cds.Camellia_sinensis.comp32818_c0_seq3_m.31144	transmembrane protein [Arabidopsis thaliana]	14.878	6.5071	9.6	1	2	1	0.95	1.016	1.006	0.988	0.973	1.08	1.023	5.95E-01
cds.Camellia_sinensis.comp32820_c0_seq1_m.30501	S-adenosyl-L-methionine-dependent methyltransferase [Medicago truncatula]	17.397	21.181	19.4	3	4	3	0.967	1.062	0.984	1.076	0.977	0.996	1.012	7.88E-01

cds.Camellia_sinensis.comp32820_c0_seq4_m.31281	S-adenosyl-L-methionine-dependent methyltransferase [Medicago truncatula]	11.507	36.026	52.9	4	9	4	0.991	0.996	1.038	1.018	0.983	0.966	0.981	4.14E-01
cds.Camellia_sinensis.comp32825_c0_seq1_m.3789	PREDICTED: nuclear pore complex protein GP210 [Vitis vinifera]	89.918	11.104	1.5	1	1	1	0.991	1.061	1.044	1.063	0.924	0.926	0.941	2.84E-01
cds.Camellia_sinensis.comp32825_c0_seq3_m.9245	PREDICTED: nuclear pore complex protein GP210 [Vitis vinifera]	92.267	11.033	3.6	3	3	3	0.961	1.018	1.062	1.009	0.967	0.995	0.977	5.13E-01
cds.Camellia_sinensis.comp32827_c0_seq1_m.7451	PREDICTED: granule-bound starch synthase 2, chloroplastic/amyloplastic isoform X2 [Vitis vinifera]	83.244	17.404	6	5	6	5	0.986	1.003	1.04	0.965	1.035	1.009	0.993	8.05E-01

cds.Camellia_sinensis.comp32828_c0_seq1_m.1768	PREDICTED: enhancer of mRNA-decapping protein 4-like [Sesamum indicum]	110.09	107.56	13.2	13	16	13	0.966	0.997	0.996	1.006	1.02	1.037	1.035	6.36E-02
cds.Camellia_sinensis.comp32828_c0_seq1_m.1769	PREDICTED: enhancer of mRNA-decapping protein 4 [Vitis vinifera]	29.879	2.5966	5.8	1	2	1								
cds.Camellia_sinensis.comp32831_c0_seq1_m.10962	PREDICTED: long chain acyl-CoA synthetase 2 [Ipomoea nil]	49.022	1.6066	1.6	1	2	1								
cds.Camellia_sinensis.comp32836_c0_seq2_m.19349	PREDICTED: protein SSUH2 homolog [Ricinus communis]	48.317	72.251	40.8	16	28	16	0.947	0.95	0.941	1.088	1.057	1.054	1.127	3.41E-04
cds.Camellia_sinensis.comp32846_c0_seq1_m.32656	PREDICTED: cinnamoyl-CoA reductase 2-like [Nicotiana attenuata]	35.4	73.272	36.4	12	18	12	1.001	0.981	1.008	1.008	0.986	1.031	1.012	4.91E-01

cds.Camellia_sinensis.comp32847_c0_seq1_m.24332	PREDICTED: RNA-binding KH domain-containing protein PEPPER [Vitis vinifera]	49.479	37.144	23.6	11	15	6	0.972	0.927	0.928	1.025	1.082	1.017	1.105	1.62E-02
cds.Camellia_sinensis.comp32847_c0_seq2_m.26434	RNA-binding KH domain-containing protein PEPPER-like [Cajanus cajan]	48.055	79.058	26.5	12	16	7	0.971	0.968	0.965	1.025	1.035	1.009	1.057	1.94E-03
cds.Camellia_sinensis.comp32850_c0_seq2_m.6603	PREDICTED: alpha-glucosidase-like [Vitis vinifera]	102.68	24.61	10.1	9	16	7	0.927	0.924	0.919	1.098	1.069	1.111	1.183	1.37E-04
cds.Camellia_sinensis.comp32850_c0_seq3_m.7253	PREDICTED: alpha-glucosidase [Juglans regia]	103.12	9.0077	5.3	5	10	3	0.914	0.921	0.901	1.118	1.092	1.103	1.211	3.70E-05
cds.Camellia_sinensis.comp32851_c0_seq1_m.13429	PREDICTED: peroxisomal membrane protein PEX14 [Juglans regia]	37.63	18.71	11.5	5	5	5	0.983	0.989	0.992	0.994	0.986	1.043	1.02	3.88E-01

cds.Camellia_sinensis.comp32851_c0_seq1_m.13430	PREDICTED: peroxisomal membrane protein PEX14 [Jatropha curcas]	31.968	14.919	18.5	4	5	4	1.081	0.977	0.977	0.992	1.032	0.936	0.975	6.05E-01		
cds.Camellia_sinensis.comp32857_c0_seq2_m.10287	PREDICTED: somatic embryogenesis receptor kinase 2 [Vitis vinifera]	68.949	1.5451	2.7	2	3	2										
cds.Camellia_sinensis.comp32863_c1_seq1_m.3632	PREDICTED: phototropin-1 isoform X2 [Vitis vinifera]	114.58	1.435	2.5	3	3	0										
cds.Camellia_sinensis.comp32865_c0_seq1_m.13403	PREDICTED: probable aspartyl aminopeptidase [Juglans regia]	53.372	60.294	23.3	10	14	10	0.937	0.953	0.975	1.042	1.076	1.055	1.108	2.34E-03		

cds.Camellia_sinensis.comp32867_c1_seq2_m.38138	PREDICTED: ubiquitin-conjugating enzyme E2 2 isoform X1 [Gossypium hirsutum]	17.334	1.7217	15.8	2	2	1	0.995	1.102	0.964	0.943	0.956	1.039	0.96	4.69E-01
cds.Camellia_sinensis.comp32867_c2_seq1_m.1279	PREDICTED: nucleolin 2-like isoform X1 [Juglans regia]	101.17	1.6245	1.5	1	1	1								
cds.Camellia_sinensis.comp32867_c2_seq2_m.8329	PREDICTED: exocyst complex component EXO70A1 [Vitis vinifera]	74.002	-2	1.5	1	1	1	1.068	1.022	1.13	0.954	0.917	0.899	0.86	1.15E-02
cds.Camellia_sinensis.comp32869_c0_seq59_m.46979	PREDICTED: V-type proton ATPase subunit F [Sesamum	14.523	15.556	21.5	3	4	3	1.01	0.97	0.926	1.066	1.053	0.993	1.071	1.08E-01

cds.Camellia_sinensis.comp32871_c0_seq1_m.5864	PREDICTED: exocyst complex component SEC3A [Theobroma cacao]	100.12	11.192	3.3	3	3	3	0.99	0.984	1.068	0.981	0.953	1.048	0.98	6.31E-01
cds.Camellia_sinensis.comp32872_c0_seq11_m.40246	PREDICTED: photosynthetic NDH subunit of lumenal location 1, chloroplastic isoform X1 [Nelumbo nucifera]	23.951	35.807	43.3	9	17	9	0.97	0.988	1.014	0.976	1.025	0.998	1.009	6.62E-01
cds.Camellia_sinensis.comp32872_c0_seq5_m.2326	PREDICTED: dolichyl-diphosphooligosaccharide--protein glycosyltransferase subunit 1B [Jatropha curcas]	52.023	18.148	14.7	8	9	8	1.006	0.972	0.992	1.007	0.992	1.047	1.026	2.55E-01
cds.Camellia_sinensis.comp32875_c0_seq1_m.22914	PREDICTED: elongation factor Ts, mitochondrial [Ricinus communis]	21.138	2.5364	13.3	3	3	3	1.024	0.921	1.041	1.022	1.003	1	1.013	7.31E-01

cds.Camellia_sinensis.comp32876_c0_seq9_m.20024	histidine phosphatase family (branch 1) protein [Medicago truncatula]	26.609	11.541	15.8	4	6	4	0.972	0.981	0.94	1.01	1.056	1.062	1.081	1.90E-02
cds.Camellia_sinensis.comp32880_c0_seq2_m.10056	PREDICTED: topless-related protein 3-like [Sesamum indicum]	82.193	10.865	7.5	6	7	6	0.917	0.938	0.951	1.073	1.118	1.053	1.156	2.16E-03
cds.Camellia_sinensis.comp32891_c1_seq3_m.39628	PREDICTED: protein CREG1 isoform X1 [Sesamum indicum]	22.324	5.7675	10	2	3	2	1.033	1.019	0.927	0.949	1.024	1.05	1.015	7.61E-01
cds.Camellia_sinensis.comp32896_c0_seq2_m.3153	PREDICTED: protein transport protein Sec24-like At4g32640 isoform X1 [Vitis vinifera]	101.38	14.385	5.5	5	5	5	0.988	0.984	1.019	0.983	1.032	1.004	1.009	6.33E-01

cds.Camellia_sinensis.comp32898_c0_seq1_m.8011	PREDICTED: NADPH--cytochrome P450 reductase [Theobroma cacao]	76.867	30.958	13.4	10	13	10	1.009	1.127	1.058	0.968	0.919	0.92	0.879	2.41E-02
cds.Camellia_sinensis.comp32899_c0_seq1_m.5712	PREDICTED: polyribonucleotide nucleotidyltransferase 2, mitochondrial [Vitis vinifera]	103.65	4.9102	4.3	5	5	5	0.922	1.009	1.024	1.056	1.02	0.995	1.039	3.49E-01
cds.Camellia_sinensis.comp32900_c0_seq2_m.8385	PREDICTED: exocyst complex component EXO84B [Vitis vinifera]	85.674	10.938	2.7	2	3	2	1.068	1.074	0.915	1.052	0.914	0.976	0.962	6.04E-01
cds.Camellia_sinensis.comp32901_c0_seq1_m.18562	PREDICTED: low-temperature-induced cysteine proteinase [Vitis vinifera]	55.425	53.097	11	5	16	4	1.038	1.007	0.973	0.984	0.982	0.967	0.972	2.20E-01

cds.Camellia_sinensis.comp32902_c0_seq1_m.22717	PREDICTED: cytochrome P450 81E8-like [Fragaria vesca subsp. vesca]	56.736	6.5762	6.4	3	3	1	1.005	1.139	1.048	0.969	0.936	0.898	0.878	3.95E-02
cds.Camellia_sinensis.comp32903_c0_seq1_m.28618	PREDICTED: short-chain dehydrogenase TIC 32, chloroplastic [Eucalyptus grandis]	35.246	1.6557	2.8	1	1	1								
cds.Camellia_sinensis.comp32905_c0_seq2_m.23124	PREDICTED: rab escort protein 1 [Vitis vinifera]	21.926	1.1978	3.5	1	1	1	0.936	0.937	1.07	1	1.009	1.069	1.046	3.98E-01
cds.Camellia_sinensis.comp32908_c0_seq1_m.21148	PREDICTED: beta-glucosidase-like SFR2, chloroplastic isoform X2 [Vitis vinifera]	66.04	32.834	13.5	8	11	8	0.982	1.025	1.031	0.989	0.991	1.016	0.986	4.79E-01
cds.Camellia_sinensis.comp32914_c0_seq1_m.2641	PREDICTED: myosin-9-like [Populus euphratica]	139.41	177.1	31.8	36	48	36	1.02	1.015	1.016	0.966	0.962	0.99	0.956	7.94E-03

cds.Camellia_sinensis.comp32917_c0_seq4_m.31727	PREDICTED: chorismate mutase 1, chloroplastic-like isoform X1 [Nelumbo nucifera]	36.759	20.297	23.6	7	7	5	1.255	0.89	1.197	0.779	0.974	0.861	0.782	1.30E-01
cds.Camellia_sinensis.comp32917_c0_seq5_m.34127	PREDICTED: chorismate mutase 3, chloroplastic [Prunus mume]	37.031	2.032	12.7	4	4	2	0.983	1.096	0.644	0.883	1.121	1.283	1.207	3.61E-01
cds.Camellia_sinensis.comp32919_c0_seq1_m.35142	PREDICTED: shootin-1-like [Juglans regia]	30.589	1.7725	4	1	1	1	0.956	1.057	1.04	0.983	1.033	0.944	0.97	4.92E-01
cds.Camellia_sinensis.comp32921_c0_seq3_m.29082	PREDICTED: ATP-dependent (S)-NAD(P)H-hydrate dehydratase isoform X2 [Juglans regia]	39.62	24.919	31.9	9	11	7	0.939	0.969	0.979	1.046	1.083	1.011	1.088	2.35E-02

cds.Camellia_sinensis.comp32921_c0_seq51_m.30515	PREDICTED: ATP-dependent (S)-NAD(P)H-hydrate dehydratase isoform X2 [Juglans regia]	39.752	5.2957	11.2	4	5	2	0.906	0.925	0.962	1.063	1.14	1.041	1.161	1.02E-02
cds.Camellia_sinensis.comp32922_c0_seq8_m.5477	COP1-interacting-like protein [Medicago truncatula]	91.503	-2	1.2	1	1	1	0.896	0.955	1.058	1.035	1.033	1.051	1.072	2.77E-01
cds.Camellia_sinensis.comp32929_c0_seq1_m.26024	PREDICTED: IAA-amino acid hydrolase ILR1-like 4 [Vitis vinifera]	46.508	43.968	30.8	11	20	11	0.977	0.977	0.971	1.025	1.044	1.009	1.052	7.20E-03
cds.Camellia_sinensis.comp32936_c1_seq16_c1_seq1_m.15922	PREDICTED: methyl-CpG-binding domain-containing protein 11-like [Nelumbo nucifera]	17.779	1.9602	7.5	1	1	1	1.15	1.065	1.133	0.835	0.955	0.83	0.783	8.68E-03
cds.Camellia_sinensis.comp32936_c1_seq1_m.15923		17.216	2.7012	12.8	2	2	2	1.235	1.101	1.145	0.778	0.783	0.902	0.708	4.14E-03

cds.Camellia_sinensis.comp32938_c0_seq1_m.2560	PREDICTED: ABC transporter B family member 27-like [Juglans regia]	69.361	9.9672	8.1	6	8	6	0.991	1.023	1.068	1.025	0.953	1.032	0.977	5.16E-01
cds.Camellia_sinensis.comp32939_c0_seq1_m.21635	PREDICTED: probable protein phosphatase 2C 59 isoform X3 [Ziziphus jujuba]	33.229	16.795	20.6	6	7	4	1.019	0.94	0.931	1.031	1.035	1.031	1.072	1.35E-01
cds.Camellia_sinensis.comp32940_c0_seq1_m.6630	PREDICTED: receptor-like protein kinase FERONIA [Eucalyptus grandis]	97.286	3.5028	2	2	2	1	1.034	0.994	0.975	1.016	0.975	1.011	1	9.93E-01
cds.Camellia_sinensis.comp32944_c0_seq2_m.12177	PREDICTED: phosphoribosylaminoimidazole carboxylase, chloroplastic isoform X1 [Vitis vinifera]	63.85	67.56	21.8	10	12	10	0.963	0.957	0.979	1.063	0.97	1.037	1.059	1.15E-01

cds.Camellia_sinensis.comp32948_c0_seq1_m.27404	PREDICTED: early nodulin-93-like isoform X1 [Cucumis sativus]	12.508	1.3107	8.6	1	3	1	0.938	1.017	0.848	1.08	0.968	1.175	1.15	1.44E-01
cds.Camellia_sinensis.comp32951_c0_seq41_m.5915	PREDICTED: probable fructokinase-6, chloroplastic [Prunus mume]	42.152	140.47	35.2	11	26	10	1.039	1.025	1.034	0.958	0.961	1.011	0.946	3.51E-02
cds.Camellia_sinensis.comp32954_c0_seq1_m.19253	PREDICTED: tocopherol cyclase, chloroplastic-like [Juglans regia]	57.86	69.003	30.9	14	22	14	0.996	1.002	0.997	0.983	0.986	1.035	1.003	8.86E-01
cds.Camellia_sinensis.comp32955_c1_seq25_m.24660	spermine synthase-like [Pyrus x bretschneideri]	17.418	2.7036	9	1	2	1								
cds.Camellia_sinensis.comp32956_c0_seq16_m.16454	PREDICTED: proline--tRNA ligase, chloroplastic/mitochondrial [Vitis vinifera]	41.292	38.907	32.7	11	13	11	0.982	0.983	0.998	1.002	0.996	1.009	1.015	8.34E-02

cds.Camellia_sinensis.comp32956_c0_seq1_m.16455	PREDICTED: proline--tRNA ligase, chloroplastic/mitochondrial-like [Ziziphus jujuba]	21.203	19.885	6.8	1	2	1	1.017	0.983	1.017	0.892	1.013	1.076	0.988	8.05E-01
cds.Camellia_sinensis.comp32958_c0_seq1_m.15709	PREDICTED: protein disulfide isomerase-like 1-6 [Juglans regia]	69.483	34.066	17.8	10	15	10	0.946	0.985	0.997	1.04	1.068	1.033	1.073	2.03E-02
cds.Camellia_sinensis.comp32960_c0_seq25_m.26509	PREDICTED: ubiquitin thioesterase otubain-like [Prunus mume]	34.039	10.643	8.9	3	4	3	0.966	0.948	1.032	0.979	0.98	1.018	1.011	7.22E-01
cds.Camellia_sinensis.comp32962_c0_seq4_m.49070	PREDICTED: probable complex I intermediate-associated protein 30 [Eucalyptus grandis]	15.347	12.445	11.1	1	2	1	0.956	1.038	0.977	0.961	1.041	1.04	1.024	5.51E-01

cds.Camellia_sinensis.comp32965_c0_seq10_m.29424	PREDICTED: 15-cis-phytoene desaturase-like [Citrus sinensis]	25.462	10.155	10.1	2	2	2	0.848	0.931	0.922	1.172	1.143	1.038	1.241	1.04E-02
cds.Camellia_sinensis.comp32965_c0_seq6_m.16402	Phytoene dehydrogenase [Morus notabilis]	14.666	17.376	38.9	4	4	4	0.87	0.907	0.997	1.173	0.931	1.081	1.148	1.61E-01
cds.Camellia_sinensis.comp32966_c0_seq4_m.46913	Uncharacterized RNA-binding protein [Morus notabilis]	21.176	28.295	34.6	5	13	4	0.971	0.973	0.966	1.049	1.028	1.051	1.075	6.19E-04
cds.Camellia_sinensis.comp32966_c1_seq1_m.3138	PREDICTED: beta-xylosidase/alpha-L-arabinofuranosidase 2 [Theobroma cacao]	85.23	89.468	21.5	14	39	13	0.986	0.96	0.945	1.025	1.033	1.043	1.073	6.30E-03
cds.Camellia_sinensis.comp32970_c0_seq1_m.25443	PREDICTED: 26S protease regulatory subunit 8 homolog A-like [Nelumbo nucifera]	47.343	16.414	43.8	16	36	1	1.079	0.963	1.092	0.988	0.91	0.963	0.913	1.27E-01

cds.Camellia_sinensis.comp32970_c0_seq2_m.28918	PREDICTED: 26S protease regulatory subunit 8 homolog A [Elaeis guineensis	47.36	113.48	48.3	18	38	3	0.964	0.962	1.009	1	1.06	0.988	1.039	2.33E-01
cds.Camellia_sinensis.comp32974_c0_seq1_m.6813	PREDICTED: protein VACUOLELESS1 [Ziziphus jujuba]	29.532	2.8655	3.8	1	1	1	1.05	1.13	0.945	0.921	0.866	1.073	0.915	3.35E-01
cds.Camellia_sinensis.comp32978_c1_seq1_m.3957	DUF1680 domain protein [Medicago truncatula]	96.576	123.37	22.1	17	35	17	1.033	1.019	1.025	0.962	0.959	0.958	0.936	8.42E-05
cds.Camellia_sinensis.comp32978_c2_seq3_m.35595	PREDICTED: thioredoxin-like 2, chloroplastic [Juglans regia]	24.845	2.7549	5.3	1	2	1	1.085	0.933	0.968	1.065	1.012	0.944	1.012	8.38E-01
cds.Camellia_sinensis.comp32979_c0_seq7_m.19787	PREDICTED: starch synthase VI isoform X1 [Vitis vinifera]	67.189	36.163	14.8	7	8	7	0.965	0.963	0.97	0.995	1.086	1.054	1.082	9.41E-02

cds.Camellia_sinensis.comp32982_c0_seq1_m.22597	PREDICTED: tryptophan synthase beta chain 1 [Cucumis melo]	52.249	66.155	27.4	12	16	12	0.997	1.003	0.961	1.006	1.046	0.993	1.028	2.46E-01
cds.Camellia_sinensis.comp32990_c0_seq1_m.16756	PREDICTED: beta-hexosaminidase 3 [Juglans regia]	63.604	144.08	31.3	17	44	16	1.013	1.011	1.014	0.993	1.012	0.968	0.979	2.33E-01
cds.Camellia_sinensis.comp32992_c0_seq3_m.44235	PREDICTED: 7-hydroxymethyl chlorophyll a reductase, chloroplastic [Daucus carota subsp. sativus]	21.122	3.9041	9.7	2	3	2	1.033	1.027	0.997	1.034	0.94	0.972	0.964	2.78E-01
cds.Camellia_sinensis.comp32994_c0_seq5_m.31489	PREDICTED: beta-glucosidase 11-like [Vitis vinifera]	15.516	4.7417	9.2	1	1	1	0.96	1.008	1.005	1.136	1.064	0.854	1.027	8.32E-01

cds.Camellia_sinensis.comp33001_c0_seq1_m.24338	PREDICTED: probable envelope ADP,ATP carrier protein, chloroplastic [Sesamum indicum]	46.369	1.7697	2.6	1	1	1								
cds.Camellia_sinensis.comp33002_c0_seq2_m.11764	PREDICTED: cleavage and polyadenylation specificity factor subunit 2 isoform X1 [Vitis vinifera]	55.953	1.9264	2	1	2	1	1.014	0.927	0.992	0.975	1.068	1.036	1.05	2.70E-01
cds.Camellia_sinensis.comp33002_c1_seq1_m.14584	PREDICTED: TBCC domain-containing protein 1 [Vitis vinifera]	62.496	25.606	9.9	5	9	5	0.983	1.014	0.915	1.035	1.079	1.057	1.089	5.83E-02

cds.Camellia_sinensis.comp33003_c0_seq1_m.29734	PREDICTED: mitochondrial dicarboxylate/tricarboxylate transporter DTC [Sesamum indicum]	31.998	72.629	28.2	7	16	6	1.008	0.982	0.987	1.007	1.001	1.026	1.019	1.57E-01
cds.Camellia_sinensis.comp33003_c0_seq2_m.47039	PREDICTED: mitochondrial dicarboxylate/tricarboxylate transporter DTC [Pyrus x bretschneideri]	12.97	-2	12.6	2	4	1	0.981	1.095	1.069	0.989	0.891	0.976	0.908	1.09E-01
cds.Camellia_sinensis.comp33006_c0_seq1_m.2880	PREDICTED: protein CROWDED NUCLEI 1 [Vitis	125.75	8.027	4	5	6	4								

cds.Camellia_sinensis.comp33007_c0_seq1_m.17728	PREDICTED: sorting and assembly machinery component 50 homolog B-like [Solanum tuberosum	18.616	4.8031	9.7	1	1	1								
cds.Camellia_sinensis.comp33008_c0_seq1_m.20904	PREDICTED: AT-hook motif nuclear-localized protein 1-like [Ipomoea nil]	34.445	13.888	11.1	3	3	3	0.96	0.877	0.987	1.013	1.086	1.087	1.128	4.66E-02
cds.Camellia_sinensis.comp33009_c0_seq1_m.13504	PREDICTED: transmembrane 9 superfamily member 3 [Ipomoea nil]	67.928	6.5648	7.8	5	6	4	0.968	0.989	1.114	1.033	0.954	0.954	0.958	4.57E-01
cds.Camellia_sinensis.comp33009_c0_seq2_m.44801	PREDICTED: transmembrane 9 superfamily member 3 [Nelumbo nucifera]	24.91	3.5015	17.4	4	5	3	0.992	1.024	1.056	0.997	0.959	0.978	0.955	9.76E-02

cds.Camellia_sinensis.comp33013_c0_seq1_m.25050	PREDICTED: protein TRIGALACTOSYLDIACYLGLYCEROL 4, chloroplastic [Vitis vinifera]	52.738	5.8688	5.7	3	4	3	1.004	0.953	0.984	0.997	1.005	0.996	1.019	2.77E-01
cds.Camellia_sinensis.comp33013_c0_seq2_m.25698	PREDICTED: protein TRIGALACTOSYLDIACYLGLYCEROL 4, chloroplastic [Prunus mume]	52.588	5.4637	6.5	3	5	3	0.96	1.018	1.1	1.062	0.918	0.956	0.954	4.63E-01
cds.Camellia_sinensis.comp33014_c0_seq5_m.19830	PREDICTED: zinc finger CCCH domain-containing protein 49 [Vitis vinifera]	55.008	9.6304	13.8	6	6	6	1.018	1.006	1.07	0.958	0.948	1	0.939	6.63E-02
cds.Camellia_sinensis.comp33017_c1_seq47_m.330	NADH dehydrogenase subunit 7 (chloroplast) [Camellia sinensis]	45.653	55.381	23.2	8	13	8	0.997	0.984	0.974	1.013	1.014	0.997	1.023	5.66E-02

cds.Camellia_sinensis.comp33017_c1_seq64_m.1059	photosystem II p680 chlorophyll A apoprotein (chloroplast) [Camellia crapnelliana]	39.493	212.71	27.2	9	64	9	0.965	1.013	0.978	1.034	1.007	1.018	1.035	1.04E-01
cds.Camellia_sinensis.comp33017_c1_seq64_m.1060	ATP-dependent Clp protease proteolytic subunit (chloroplast) [Diplopanax stachyanthus]	21.995	46.072	18.4	4	11	4	0.992	1.014	1.016	0.998	1.009	0.985	0.99	3.90E-01
cds.Camellia_sinensis.comp33017_c1_seq66_m.1341	NADH dehydrogenase subunit 2 (chloroplast) [Ardisia polysticta]	58.583	3.0367	3.1	2	2	2	1.036	1.034	1.023	0.97	0.902	1.03	0.938	2.33E-01
cds.Camellia_sinensis.comp33017_c1_seq67_m.1798	photosystem II protein D1 (chloroplast) [Scopolia parviflora]	38.95	127.22	22.9	8	59	8	0.966	0.987	0.987	1.021	1.022	1.027	1.044	4.24E-03

cds.Camellia_sinensis.comp33017_c1_seq9_m.116	Ycf2 (chloroplast) [Camellia sinensis]	56.595	2.2689	2.7	1	1	1								
cds.Camellia_sinensis.comp33017_c1_seq9_m.118	DNA-directed RNA polymerase alpha chain (chloroplast) [Camellia crapnelliana]	39.538	19.373	18.7	6	8	6	0.951	0.919	0.982	1.032	0.995	1.071	1.086	4.46E-02
cds.Camellia_sinensis.comp33017_c1_seq9_m.123	ribosomal protein L2 (chloroplast) [Nicotiana otophora]	15.145	9.8927	28.9	3	8	3	1.132	1.08	1.058	0.874	0.858	0.899	0.805	8.45E-04
cds.Camellia_sinensis.comp33017_c1_seq9_m.125	ribosomal protein S11 (plastid) [Lysimachia coreana]	15.028	46.804	26.8	3	7	3	1.011	1.069	1.093	0.899	0.932	0.955	0.878	1.11E-02
cds.Camellia_sinensis.comp33017_c1_seq9_m.131	50S ribosomal protein L14 (chloroplast) [Camellia crapnelliana]	13.535	23.786	51.6	6	12	6	0.971	0.971	1.029	1.01	1.016	0.992	1.016	4.81E-01

cds.Camellia_sinensis.comp33017_c1_seq9_m.134	30S ribosomal protein S7 (chloroplast) [Camellia crapnelliana]	12.03	7.9022	26.6	3	4	3	1.105	1.034	1.135	0.882	0.931	0.924	0.836	5.30E-03
cds.Camellia_sinensis.comp33018_c1_seq1_m.33727	PREDICTED: glutathione S-transferase T1 [Ziziphus jujuba]	28.014	32.788	37.2	9	26	4	0.914	0.944	0.973	1.088	1.045	1.081	1.135	4.44E-03
cds.Camellia_sinensis.comp33018_c1_seq2_m.49711	PREDICTED: glutathione S-transferase T1 [Ricinus communis]	21.296	100.49	58.8	11	27	6	0.867	0.869	0.879	1.174	1.091	1.129	1.298	2.76E-04
cds.Camellia_sinensis.comp33019_c0_seq5_m.5844	PREDICTED: ABC transporter A family member 7 [Vitis vinifera]	106	5.1401	2.4	2	2	2								
cds.Camellia_sinensis.comp33028_c0_seq1_m.3160	PREDICTED: protein transport protein Sec24-like At3g07100 [Vitis vinifera]	115.42	5.728	4.7	5	5	5	1.027	0.988	0.999	1	1.058	1.005	1.016	4.98E-01

cds.Camellia_sinensis.comp33032_c0_seq1_m.4992	PREDICTED: chaperone protein ClpB3, chloroplastic isoform X2 [Sesamum indicum]	110.2	296.03	42.7	39	73	36	1.02	1.023	1.026	0.984	0.981	0.976	0.958	1.24E-04
cds.Camellia_sinensis.comp33033_c0_seq4_m.6390	RNA ligase, putative [Medicago truncatula]	29.422	6.8522	6.5	1	1	1								
cds.Camellia_sinensis.comp33043_c1_seq1_m.8511	PREDICTED: isoamylase 3, chloroplastic-like [Malus domestica]	93.557	76.25	17.3	13	15	13	1.051	1.081	1.018	0.93	0.95	0.936	0.894	3.74E-03
cds.Camellia_sinensis.comp33050_c1_seq1_m.7876	PREDICTED: sucrose synthase 2 [Ziziphus jujuba]	101.16	323.31	36.2	32	76	29	0.905	0.925	0.905	1.103	1.109	1.095	1.209	2.01E-05
cds.Camellia_sinensis.comp33050_c2_seq5_m.26955	PREDICTED: epimerase family protein SDR39U1 homolog, chloroplastic [Juglans regia]	41.043	69.33	32.8	11	24	11	0.958	0.96	0.98	1.009	1.078	1.014	1.07	4.02E-02

cds.Camellia_sinensis.comp33055_c0_seq1_m.12922	PREDICTED: ubiquitin domain-containing protein DSK2a-like [Nelumbo nucifera]	59.193	46.334	16.5	6	11	5	1.034	1.039	1.024	1.035	0.97	0.969	0.96	1.39E-01
cds.Camellia_sinensis.comp33067_c0_seq2_m.36662	PREDICTED: 60S ribosomal protein L23a [Cucumis melo]	17.502	31.985	26.5	4	9	4	1.15	1.116	1.084	0.831	0.917	0.881	0.785	1.88E-03
cds.Camellia_sinensis.comp33067_c1_seq1_m.17116	PREDICTED: calcium-dependent protein kinase 26-like [Gossypium hirsutum]	60.422	17.947	16.4	7	8	7	0.989	1.028	1.02	1.028	0.966	0.964	0.974	3.32E-01
cds.Camellia_sinensis.comp33070_c0_seq1_m.2815	PREDICTED: fructokinase-like 2, chloroplastic isoform X2 [Vitis vinifera]	64.81	1.8299	2.3	1	1	1								

cds.Camellia_sinensis.comp33070_c0_seq3_m.5410	PREDICTED: folate synthesis bifunctional protein, mitochondrial [Vitis vinifera]	55.462	8.9523	7.7	4	6	4	0.946	1.095	1.055	0.914	0.973	1.02	0.939	3.16E-01
cds.Camellia_sinensis.comp33070_c1_seq1_m.4292	PREDICTED: AP-4 complex subunit epsilon [Jatropha curcas]	112.72	27.531	9.4	9	14	9	0.991	0.979	1	1.011	0.97	0.982	0.998	8.66E-01
cds.Camellia_sinensis.comp33072_c0_seq1_m.25537	PREDICTED: protein TIC 40, chloroplastic isoform X1 [Vitis vinifera]	49.159	65.856	33	13	29	13	0.967	1.023	1.02	0.997	1.024	0.983	0.998	9.38E-01
cds.Camellia_sinensis.comp33078_c0_seq1_m.10577	PREDICTED: ABC transporter B family member 28 [Prunus mume]	78.06	37.098	18.2	11	12	10	1.003	0.961	0.944	1.03	1.013	1.023	1.054	4.61E-02

cds.Camellia_sinensis.comp33084_c4_seq1_m.45006	PREDICTED: nascent polypeptide-associated complex subunit alpha-like protein 1 [Elaeis guineensis	21.74	25.533	22.1	3	9	3	1.01	1.026	1.023	0.978	1.004	0.922	0.949	1.10E-01
cds.Camellia_sinensis.comp33084_c5_seq1_m.15203	PREDICTED: ATP synthase subunit beta, mitochondrial-like [Solanum pennellii]	59.463	323.31	53.2	22	91	19	1.034	1.003	1.02	0.991	0.971	0.981	0.963	2.31E-02
cds.Camellia_sinensis.comp33084_c5_seq2_m.50711	PREDICTED: ATP synthase subunit beta, mitochondrial-like [Nicotiana attenuata]	17.821	6.558	22.5	3	14	1	0.98	0.985	0.959	1.022	1.016	1.055	1.058	1.71E-02
cds.Camellia_sinensis.comp33087_c0_seq1_m.7166	PREDICTED: phospholipase D delta isoform X3 [Vitis vinifera]	97.908	13.033	10.2	8	9	8	0.907	0.899	0.961	1.077	1.069	1.012	1.141	9.94E-03

cds.Camellia_sinensis.comp33092_c0_seq1_m.9775	PREDICTED: BEL1-like homeodomain protein 1 [Sesamum indicum]	72.048	11.451	4.8	3	4	2	0.962	0.952	0.93	1.091	1.047	1.042	1.118	3.22E-03
cds.Camellia_sinensis.comp33094_c1_seq3_m.7239		18.899	-2	4.1	1	2	1	0.986	0.93	1.021	0.972	1.037	1.068	1.048	2.98E-01
cds.Camellia_sinensis.comp33096_c0_seq1_m.17329	PREDICTED: dnaJ protein P58IPK homolog [Prunus mume]	53.241	8.4665	13.6	6	6	6	0.954	0.982	0.999	1.089	0.98	1.02	1.052	2.08E-01
cds.Camellia_sinensis.comp33098_c0_seq1_m.2140	PREDICTED: ABC transporter C family member 14 [Juglans	164.88	28.019	6.4	8	9	7	1.115	1.088	1.181	0.829	0.87	0.89	0.765	1.12E-03
cds.Camellia_sinensis.comp33104_c0_seq1_m.8567	PREDICTED: protein CHUP1, chloroplastic isoform X1 [Gossypium raimondii]	33.717	12.278	24.8	7	9	7	1.083	1.064	1.072	0.913	0.911	0.943	0.86	2.44E-04

cds.Camellia_sinensis.comp33104_c0_seq1_m.8568	P-loop nucleoside triphosphate hydrolase superfamily protein [Medicago truncatula]	32.664	3.7664	11.2	3	3	3	0.973	0.994	0.954	1.123	0.971	1.007	1.062	2.69E-01
cds.Camellia_sinensis.comp33104_c1_seq1_m.10864	PREDICTED: protein CHUP1, chloroplastic [Vitis vinifera]	76.402	28.278	14.6	10	10	10	1.098	1.072	1.096	0.899	0.883	0.924	0.829	2.45E-04
cds.Camellia_sinensis.comp33106_c0_seq2_m.24466	PREDICTED: proline iminopeptidase [Juglans regia]	44.324	109.41	41.3	15	30	15	0.957	0.968	0.953	1.047	1.039	1.028	1.082	3.64E-04
cds.Camellia_sinensis.comp33108_c0_seq2_m.12576	PREDICTED: zinc finger CCCH domain-containing protein 64 isoform X2 [Vitis vinifera]	40.297	4.0797	7.1	2	2	2	1.157	0.971	0.635	1.186	0.995	1.067	1.176	3.65E-01

cds.Camellia_sinensis.comp33109_c0_seq13_m.29073	PREDICTED: CBS domain-containing protein CBSX2, chloroplastic [Eucalyptus grandis]	27.432	61.034	26.6	8	12	7	1.04	1.022	1.044	0.973	0.959	0.987	0.94	4.20E-03
cds.Camellia_sinensis.comp33115_c0_seq1_m.14614	PREDICTED: monodehydroascorbate reductase 5, mitochondrial [Nelumbo nucifera]	53.595	268.25	49.6	21	54	20	0.956	0.938	0.943	1.052	1.066	1.04	1.113	2.99E-04
cds.Camellia_sinensis.comp33120_c0_seq2_m.23566	PREDICTED: FKBP12-interacting protein of 37 kDa [Theobroma cacao]	38.799	5.7355	10.1	4	4	4	0.889	1.027	1.055	1.1	1.015	0.945	1.03	6.82E-01
cds.Camellia_sinensis.comp33121_c0_seq1_m.12663	PREDICTED: acyl-CoA-binding domain-containing protein 4 [Vitis vinifera]	55.599	46.401	15	7	10	7	1.071	1.086	1.042	0.971	0.896	0.928	0.874	6.70E-03

cds.Camellia_sinensis.comp33126_c0_seq1_m.36777	PREDICTED: monothiol glutaredoxin-S15, mitochondrial [Juglans regia]	20.607	3.227	11.3	2	2	2	1.035	0.97	0.994	0.982	1.007	1.018	1.003	9.00E-01
cds.Camellia_sinensis.comp33128_c0_seq5_m.17158	PREDICTED: probable sphingolipid transporter spinster homolog 2 isoform X2 [Theobroma cacao]	54.879	2.9318	3.3	2	2	2	1.019	1.135	0.965	0.91	0.98	0.984	0.921	2.12E-01
cds.Camellia_sinensis.comp33133_c0_seq1_m.7963	PREDICTED: probable ADP-ribosylation factor GTPase-activating protein AGD8 [Ricinus communis]	43.269	28.301	18.6	6	9	6	1.079	1.07	1.05	1.01	0.902	0.928	0.888	2.63E-02

cds.Camellia_sinensis.comp33133_c0_seq1_m.7964	PREDICTED: 50S ribosomal protein L17, chloroplastic-like [Nicotiana tabacum]	32.902	3.3185	7.1	2	2	2	1.034	1.208	1.094	0.858	0.932	0.853	0.792	1.25E-02
cds.Camellia_sinensis.comp33134_c0_seq1_m.9785	PREDICTED: YTH domain-containing family protein 3-like isoform X1 [Juglans regia]	76.212	16.126	8.4	5	5	3	0.993	1.063	1.045	0.973	0.983	0.946	0.936	4.81E-02
cds.Camellia_sinensis.comp33135_c1_seq15_m.24170	Hop-interacting protein THI002 [Medicago truncatula]	41.36	1.4159	2.2	1	1	1	1.1	1.003	1.039	0.938	0.946	0.961	0.905	2.39E-02
cds.Camellia_sinensis.comp33135_c1_seq6_m.3926	PREDICTED: DNA damage-binding protein 1-like [Ipomoea nil]	47.553	29.162	19.4	8	11	8	0.976	1.029	0.953	1.016	0.99	1.039	1.029	3.33E-01

cds.Camellia_sinensis.comp33135_c1_seq8_m.4460	PREDICTED: vacuolar-sorting receptor 1-like isoform X1 [Nelumbo nucifera]	56.842	1.5406	2.8	1	1	1								
cds.Camellia_sinensis.comp33135_c1_seq9_m.5256	PREDICTED: DNA damage-binding protein 1 isoform X1 [Vitis vinifera]	70.415	18.987	11.1	7	8	7	0.93	0.95	0.962	1.09	1.049	1.022	1.112	7.40E-03
cds.Camellia_sinensis.comp33135_c1_seq9_m.5257	PREDICTED: ankyrin repeat and KH domain-containing protein R11A8.7 [Jatropha curcas]	41.956	6.5535	5.3	1	1	1								
cds.Camellia_sinensis.comp33136_c0_seq1_m.635	PREDICTED: E3 ubiquitin-protein ligase PRT6 [Vitis vinifera]	231.72	2.1827	0.6	1	1	1	1.061	1.085	1.05	1.031	0.81	0.953	0.874	1.91E-01

cds.Camellia_sinensis.comp33139_c0_seq1_m.9505	PREDICTED: calcium sensing receptor, chloroplastic isoform X1 [Citrus sinensis]	38.973	59.77	20.1	7	17	7	1.022	1.006	1.016	0.997	0.98	0.997	0.977	3.40E-02
cds.Camellia_sinensis.comp33139_c0_seq1_m.9506	PREDICTED: calcium sensing receptor, chloroplastic isoform X2 [Citrus sinensis]	34.998	73.64	33.1	12	24	12	1.049	0.977	1.021	0.985	0.987	0.998	0.975	2.99E-01
cds.Camellia_sinensis.comp33147_c0_seq4_m.18745	PREDICTED: mediator of RNA polymerase II transcription subunit 25 [Vitis vinifera]	30.668	3.4694	6.7	2	2	2	0.981	0.959	1	1.032	0.987	1.057	1.046	1.28E-01
cds.Camellia_sinensis.comp33149_c0_seq1_m.7064	PREDICTED: calmodulin-binding receptor-like cytoplasmic kinase 3 isoform X1 [Vitis vinifera]	51.687	2.0378	3.1	2	2	2	1.034	0.993	0.95	1.079	0.962	0.992	1.019	6.90E-01

cds.Camellia_sinensis.comp33150_c0_seq10_m.1236	PREDICTED: UTP--glucose-1-phosphate uridylyltransferase 3, chloroplastic isoform X4 [Vitis vinifera]	91.036	3.0028	2.6	1	1	1	0.867	0.984	0.983	0.912	1.14	1.142	1.127	2.48E-01
cds.Camellia_sinensis.comp33153_c0_seq1_m.22690	PREDICTED: phosphoribosylformylglycinamidine cyclo-ligase, chloroplastic/mitochondrial [Theobrom	49.847	73.465	12.3	6	9	6	1.019	0.993	1.014	1.003	0.997	1.005	0.993	4.51E-01
cds.Camellia_sinensis.comp33156_c0_seq26_m.19418	PREDICTED: probable ATP-dependent RNA helicase ddx5 [Vitis	48.589	-2	2.3	1	1	1	1.045	0.968	0.966	1.012	1.011	1.005	1.016	5.80E-01
cds.Camellia_sinensis.comp33158_c0_seq1_m.20871	PREDICTED: selenium-binding protein 2 [Pyrus x bretschneideri]	54.503	185.29	30.7	13	40	13	0.878	0.871	0.844	1.131	1.16	1.124	1.317	5.90E-05

cds.Camellia_sinensis.comp33158_c0_seq3_m.30038	PREDICTED: probable acyl-activating enzyme 16, chloroplastic [Vitis	51.082	14.194	14.8	6	6	5	0.971	0.968	1.028	1.129	0.991	1.038	1.064	2.26E-01
cds.Camellia_sinensis.comp33158_c0_seq4_m.30027	PREDICTED: probable acyl-activating enzyme 16, chloroplastic isoform X2	38.012	25.331	15.4	5	6	5	0.956	1.002	0.916	1.05	1.082	1.045	1.105	2.31E-02
cds.Camellia_sinensis.comp33160_c0_seq1_m.1554	PREDICTED: symplekin isoform X1 [Nicotiana attenuata]	147.23	1.4087	1	1	1	1								
cds.Camellia_sinensis.comp33160_c1_seq1_m.11786	PREDICTED: plant UBX domain-containing protein 8 [Vitis vinifera]	66.641	3.9894	15.8	9	12	2	0.986	1.08	1.096	0.897	0.964	0.973	0.896	5.98E-02

cds.Camellia_sinensis.comp33160_c1_seq20_m.16209	PREDICTED: plant UBX domain-containing protein 8 [Vitis vinifera]	68.037	95.282	33.1	18	22	11	1.015	1.037	1.047	0.965	0.935	1.012	0.94	6.46E-02
cds.Camellia_sinensis.comp33163_c0_seq1_m.21007	PREDICTED: geraniol 8-hydroxylase-like [Nicotiana attenuata]	58.702	1.5689	2.1	1	1	1	0.862	0.991	0.88	1.171	1.199	0.95	1.215	8.98E-02
cds.Camellia_sinensis.comp33165_c0_seq3_m.11874	PREDICTED: protease Do-like 2, chloroplastic [Theobroma cacao]	69.038	38.773	19.5	10	12	10	1.063	1.067	1.041	0.963	0.953	0.938	0.9	6.23E-04
cds.Camellia_sinensis.comp33167_c0_seq27_m.12525	PREDICTED: malonate--CoA ligase isoform X2 [Vitis vinifera]	54.797	28.27	19.8	8	11	8	1.002	1.008	1.009	0.98	0.97	0.992	0.974	1.94E-02
cds.Camellia_sinensis.comp33170_c0_seq11_m.16683	PREDICTED: GDHB glutamate dehydrogenase isoform X1 [Vitis vinifera]	43.292	154.4	50.6	17	34	13	0.978	0.959	0.993	1.033	1.029	1.043	1.06	5.88E-03

cds.Camellia_sinensis.comp33170_c0_seq5_m.11453	PREDICTED: TOM1-like protein 2 [Ricinus communis]	71.744	2.8349	2.6	2	2	2	1.059	0.913	1.053	1.006	0.997	0.977	0.985	8.09E-01
cds.Camellia_sinensis.comp33170_c0_seq60_m.11481	PREDICTED: protein XRI1 isoform X1 [Ziziphus jujuba]	26.516	-2	4.2	1	1	1	1.054	0.964	1.113	0.932	0.969	0.963	0.915	1.15E-01
cds.Camellia_sinensis.comp33174_c2_seq17_m.44746	PREDICTED: protein phosphatase inhibitor 2 isoform X2 [Juglans regia]	20.138	2.397	8.7	1	1	1	1.051	1.109	0.949	1.001	0.912	0.972	0.928	2.40E-01
cds.Camellia_sinensis.comp33176_c0_seq1_m.23980	PREDICTED: probable mitochondrial import inner membrane translocase subunit TIM21 [Vitis vinifera]	34.672	7.7594	10.3	3	3	3	0.862	1.062	1.05	0.993	1.01	1.011	1.013	8.18E-01

cds.Camellia_sinensis.comp33177_c0_seq1_m.33010	isoflavone reductase-like protein [Cajanus cajan]	37.403	158.3	40.5	11	31	10	1.187	1.168	1.174	0.79	0.824	0.804	0.685	4.30E-06
cds.Camellia_sinensis.comp33181_c0_seq1_m.4653	PREDICTED: long chain acyl-CoA synthetase 6, peroxisomal-like isoform X1 [Nelumbo nucifera]	19.538	1.4836	5.2	1	1	1	0.969	0.964	0.921	1.127	1.078	0.971	1.113	8.88E-02
cds.Camellia_sinensis.comp33184_c0_seq4_m.35050	PREDICTED: GTP-binding protein SAR1A-like [Juglans regia]	13.203	52.141	60	5	11	2	0.924	0.987	1.009	1.033	1.022	1.024	1.054	1.81E-01
cds.Camellia_sinensis.comp33187_c0_seq2_m.48458	PREDICTED: vicianin hydrolase-like [Populus euphratica]	18.361	3.7707	14	2	2	2	1.122	1.085	0.995	0.972	0.883	0.923	0.868	3.53E-02

cds.Camellia_sinensis.comp33194_c0_seq5_m.22855	PREDICTED: mannose-1-phosphate guanyltransferase alpha [Capsicum annuum]	45.754	12.245	10.1	4	4	3	1.039	1.066	0.956	1.016	0.863	1.057	0.959	5.62E-01
cds.Camellia_sinensis.comp33196_c0_seq2_m.28361	PREDICTED: 26S proteasome non-ATPase regulatory subunit 14 homolog [Juglans regia]	36.592	34.887	24.4	8	12	8	0.978	1.024	0.975	1.046	1.023	1.029	1.041	8.04E-02
cds.Camellia_sinensis.comp33200_c0_seq1_m.32195	PREDICTED: protein FLX-like 3 [Vitis vinifera]	30.858	1.5811	16.8	4	5	2	0.891	0.983	1.099	0.981	1.084	0.987	1.027	6.96E-01
cds.Camellia_sinensis.comp33200_c0_seq2_m.34220	PREDICTED: protein FLX-like 3 isoform X1 [Nicotiana tabacum]	31.569	26.988	33.3	7	12	6	1.009	0.997	1.013	0.981	1.018	0.988	0.989	4.31E-01

cds.Camellia_sinensis.comp33210_c0_seq10_c0_seq1_m.934	PREDICTED: protein argonaute 1-like [Solanum tuberosum	111.95	108.01	20.5	19	25	19	1.05	1.065	1.048	0.947	0.929	0.95	0.893	1.99E-04
cds.Camellia_sinensis.comp33211_c0_seq4_m.6750	NADH dehydrogenase subunit 5 (mitochondrion) [Nelumbo nucifera]	65.746	2.6632	1.7	1	1	1	0.986	0.979	1.041	1.067	0.906	1.034	1	9.75E-01
cds.Camellia_sinensis.comp33215_c0_seq1_m.532	PREDICTED: serine-rich adhesin for platelets isoform X2 [Prunus	232.61	4.0934	1.5	3	3	1								
cds.Camellia_sinensis.comp33215_c0_seq4_m.3227	PREDICTED: serine-rich adhesin for platelets isoform X2 [Theobroma cacao]	123.77	14.409	3.2	3	3	1								
cds.Camellia_sinensis.comp33217_c0_seq2_m.9233	PREDICTED: U-box domain-containing protein 44 [Jatropha curcas]	71.843	13.476	5.9	4	4	4	1.039	0.989	1.005	0.979	1.016	0.975	0.979	3.44E-01

cds.Camellia_sinensis.comp33218_c0_seq22_m.55156	PREDICTED: yrdC domain-containing protein, mitochondrial [Jatropha curcas]	13.465	1.5347	9	1	1	1								
cds.Camellia_sinensis.comp33218_c0_seq8_m.28613	PREDICTED: phospholipase D beta 1-like [Juglans regia]	30.457	3.0651	3	1	2	1	0.858	1.031	0.904	1.065	1.064	1.114	1.161	5.50E-02
cds.Camellia_sinensis.comp33218_c1_seq13_m.38803	PREDICTED: alpha-galactosidase 3 [Malus domestica]	24.141	2.5046	4.2	1	1	1	0.897	0.913	0.886	1.139	1.099	1.111	1.242	9.66E-05
cds.Camellia_sinensis.comp33218_c1_seq6_m.23129	PREDICTED: alpha-galactosidase 3 isoform X1 [Juglans regia]	46.578	69.842	33.6	12	27	6	0.981	0.967	0.981	1.063	1.021	1.064	1.075	7.54E-03
cds.Camellia_sinensis.comp33220_c1_seq3_m.37071	transmembrane protein, putative [Medicago truncatula]	32.171	3.3328	4.2	1	1	1								

cds.Camellia_sinensis.comp33221_c2_seq1_m.12402	PREDICTED: protein IQ-DOMAIN 14 [Daucus carota subsp. sativus]	61.594	4.0248	1.8	1	1	1	1.202	1.077	0.998	0.853	0.849	0.98	0.818	4.94E-02
cds.Camellia_sinensis.comp33223_c0_seq10_m.48390	PREDICTED: glycerol kinase [Juglans regia]	21.665	23.946	14.9	3	5	3	0.899	0.974	0.952	1.094	1.061	1.098	1.152	5.68E-03
cds.Camellia_sinensis.comp33224_c0_seq1_m.18045	catalase isozyme 3 [Nicotiana tabacum]	62.315	236.63	42	21	77	14	0.994	0.976	1.006	0.997	1.032	0.999	1.017	2.92E-01
cds.Camellia_sinensis.comp33224_c0_seq3_m.44552	catalase family protein [Populus trichocarpa]	22.173	4.6852	25.5	4	15	1								
cds.Camellia_sinensis.comp33227_c0_seq34_m.36211	PREDICTED: peptidyl-tRNA hydrolase ICT1, mitochondrial isoform X1 [Nelumbo	26.743	3.3688	4.6	1	1	1	1.139	1.068	1.172	0.845	0.851	0.89	0.765	1.12E-03

cds.Camellia_sinensis.comp33228_c2_seq1_m.6837	PREDICTED: mechanosensitive ion channel protein 1, mitochondrial [Vitis vinifera]	59.262	2.6231	3	1	1	1								
cds.Camellia_sinensis.comp33229_c0_seq3_m.31359	PREDICTED: trithorax group protein osa [Ziziphus jujuba]	41.817	22.751	13.6	4	4	4	0.951	0.976	1.036	1.105	0.899	1.053	1.032	6.98E-01
cds.Camellia_sinensis.comp33229_c0_seq4_m.43060	PREDICTED: exocyst complex component SEC15B [Nelumbo nucifera]	26.615	5.6746	10.1	2	2	2	0.97	0.956	1.003	1.027	1.101	0.964	1.056	2.66E-01
cds.Camellia_sinensis.comp33232_c0_seq6_m.52447	PREDICTED: 5'-nucleotidase domain-containing protein 4 isoform X4 [Vitis vinifera]	13.525	1.8736	6.2	1	1	1	0.904	1.249	0.895	0.952	0.962	1.044	0.97	8.79E-01

cds.Camellia_sinensis.comp33237_c0_seq3_m.6037	PREDICTED: endoplasmic reticulum metallopeptidase 1 [Vitis vinifera]	101.09	17.359	7.6	7	9	7	0.969	0.966	0.955	1.053	1.056	0.991	1.073	3.02E-02
cds.Camellia_sinensis.comp33238_c0_seq1_m.13409	PREDICTED: glucose-6-phosphate 1-dehydrogenase, chloroplastic isoform X1 [Vitis vinifera]	67.627	7.1256	14.9	9	15	4	0.952	0.979	1.004	1.031	1.015	1.038	1.051	4.11E-02
cds.Camellia_sinensis.comp33239_c0_seq2_m.22246	PREDICTED: mitochondrial fission protein ELM1 [Sesamum indicum]	47.382	1.8547	3.5	2	2	2								
cds.Camellia_sinensis.comp33240_c0_seq1_m.6214	PREDICTED: transcriptional corepressor LEUNIG_HOMOLOG-like [Nicotiana tabacum]	84.145	6.6767	3.4	2	2	2	0.911	1.051	1.024	1.052	0.994	0.991	1.017	7.20E-01

cds.Camellia_sinensis.comp33243_c0_seq5_m.32025	PREDICTED: lactoylglutathione lyase-like [Glycine max]	27.943	49.157	40.4	10	22	10	0.961	0.972	0.968	1.053	1.035	1.055	1.083	3.21E-04
cds.Camellia_sinensis.comp33245_c0_seq1_m.13680	PREDICTED: laccase-14 [Vitis vinifera]	63.033	27.115	6.2	3	5	3	1.089	1.004	1.079	0.894	0.932	0.935	0.87	9.36E-03
cds.Camellia_sinensis.comp33247_c0_seq3_m.214	DNA-directed RNA polymerase beta chain (chloroplast) [Camellia crapnelliana]	121.03	24.446	7.6	6	6	6	0.959	0.979	0.989	1.04	1.042	1.013	1.057	1.22E-02
cds.Camellia_sinensis.comp33247_c0_seq3_m.215	DNA-directed RNA polymerase beta" subunit (chloroplast) [Camellia impressinervis]	103.93	6.7058	4.4	4	4	4	1.047	1.01	1.048	0.942	0.96	0.979	0.928	1.05E-02

cds.Camellia_sinensis.comp33247_c0_seq3_m.216	RNA polymerase beta' subunit (chloroplast) [Camellia sinensis]	79.614	10.2	8.1	5	5	5	1.003	1.115	1.049	0.918	0.926	0.981	0.892	3.74E-02
cds.Camellia_sinensis.comp33247_c0_seq3_m.217	RNA polymerase beta' subunit (chloroplast) [Camellia leptophylla]	54.904	1.6292	1.4	1	1	1	1.041	0.988	0.938	0.968	1.109	0.964	1.025	6.92E-01
cds.Camellia_sinensis.comp33247_c0_seq3_m.219	ribosomal protein S2 (chloroplast) [Camellia pubicosta]	26.894	25.399	22.9	5	8	5	1.027	1.086	1.153	0.913	0.913	0.908	0.837	3.40E-02
cds.Camellia_sinensis.comp33248_c0_seq1_m.4358	PREDICTED: glycine dehydrogenase (decarboxylating), mitochondrial [Nelumbo nucifera]	114.39	323.31	40.3	36	108	36	1.046	1.034	1.037	0.96	0.978	0.964	0.931	4.17E-04

cds.Camellia_sinensis.comp33249_c0_seq1_m.20076	PREDICTED: LON peptidase N-terminal domain and RING finger protein 1 isoform X1 [Sesamum indicum]	61.012	-2	1.5	1	1	1	1.168	1.091	1.121	0.815	0.843	0.92	0.763	2.74E-03
cds.Camellia_sinensis.comp33251_c0_seq1_m.12241	PREDICTED: inorganic pyrophosphatase 3-like isoform X1 [Nicotiana attenuata]	28.55	6.0627	12.4	3	3	3								
cds.Camellia_sinensis.comp33251_c0_seq9_m.52859	long chain base biosynthesis protein 1 [Cajanus cajan]	12.25	2.9165	17.7	2	2	2	1.001	0.879	0.986	1.016	1.012	1.122	1.099	1.48E-01
cds.Camellia_sinensis.comp33252_c0_seq1_m.35074	PREDICTED: (+)-neomenthol dehydrogenase-like, partial [Juglans regia]	29.037	31.462	24	6	7	6	0.963	0.937	0.952	1.049	1.038	1.071	1.107	1.10E-03

cds.Camellia_sinensis.comp33254_c0_seq4_m.22604	PREDICTED: putative clathrin assembly protein At5g35200 [Ziziphus	48.43	2.0754	4.2	2	2	1								
cds.Camellia_sinensis.comp33258_c0_seq1_m.11478	probable starch synthase 4, chloroplastic/amyloplastic [Vitis vinifera]	77.21	9.532	6.1	4	4	4	0.94	0.918	0.941	1.052	1.045	1.132	1.154	6.10E-03
cds.Camellia_sinensis.comp33258_c0_seq5_m.27121	PREDICTED: small nuclear ribonucleoprotein-associated protein B' [Jatropha curcas]	28.564	17.233	14.4	3	3	3	0.854	0.964	0.956	1.075	1.027	1.162	1.177	3.75E-02
cds.Camellia_sinensis.comp33260_c0_seq1_m.19690	PREDICTED: protein RNA-directed DNA methylation 3 [Vitis vinifera]	54.421	4.3133	3.3	1	1	1								

cds.Camellia_sinensis.comp33260_c0_seq3_m.36353	PREDICTED: protein RNA-directed DNA methylation 3 [Vitis vinifera]	36.461	3.0432	3.4	1	2	1	1.109	1.035	0.889	1.112	0.957	0.901	0.979	8.30E-01
cds.Camellia_sinensis.comp33261_c2_seq1_m.16769	PREDICTED: metal transporter Nramp3-like [Nicotiana tomentosiformis]	56.252	1.4928	3.1	1	1	1								
cds.Camellia_sinensis.comp33262_c0_seq1_m.1290	PREDICTED: ABC transporter C family member 10-like [Nicotiana attenuata]	164.51	18.033	6.3	8	10	6	1.063	1.064	1.105	0.907	0.936	0.896	0.847	8.18E-04
cds.Camellia_sinensis.comp33263_c0_seq1_m.1372	PREDICTED: AT-hook motif nuclear-localized protein 10-like [Nelumbo nucifera]	34.272	31.241	10.4	3	4	3	0.964	1.018	0.951	1.019	0.987	1.073	1.05	2.05E-01

cds.Camellia_sinensis.comp33267_c1_seq4_m.44623	PREDICTED: ribosome biogenesis protein erb1 isoform X2 [Citrus sinensis]	12.982	4.0755	27.4	2	2	2	1.24	1.164	1.115	0.763	0.827	0.831	0.688	8.39E-04
cds.Camellia_sinensis.comp33269_c0_seq1_m.34486	PREDICTED: caffeoylshikimate esterase [Vitis vinifera]	36.689	6.186	6.6	2	3	2	1.085	1.01	1.148	0.887	0.899	0.952	0.844	1.72E-02
cds.Camellia_sinensis.comp33272_c0_seq1_m.18645	PREDICTED: V-type proton ATPase subunit B 2	57.87	155.76	38.1	17	30	16	0.99	0.973	0.97	1.018	1.021	1.021	1.043	2.22E-02
cds.Camellia_sinensis.comp33275_c0_seq1_m.14084	PREDICTED: serine/threonine protein phosphatase 2A 55 kDa regulatory subunit B beta isoform-like isoform X2 [Cicer arietinum]	60.795	13.436	9.2	5	6	5	0.974	1.03	1.003	0.946	1.017	0.977	0.978	4.39E-01

cds.Camellia_sinensis.comp33278_c0_seq3_m.39726	PREDICTED: coatomer subunit delta [Vitis vinifera]	12.178	3.5319	20.2	2	4	1	0.865	0.906	1.357	1.144	0.812	0.946	0.928	7.33E-01
cds.Camellia_sinensis.comp33280_c0_seq1_m.9895	PREDICTED: nucleobase-ascorbate transporter 6 [Sesamum indicum]	57.668	3.98	2.5	1	4	1								
cds.Camellia_sinensis.comp33280_c0_seq2_m.11931	PREDICTED: nucleobase-ascorbate transporter 6 [Sesamum indicum]	57.58	18.925	7.7	4	5	4	0.988	0.969	0.967	1.004	1.045	1	1.043	5.59E-02
cds.Camellia_sinensis.comp33289_c0_seq3_m.41555	thioredoxin protein [Medicago truncatula]	27.874	6.0223	16	5	8	5	0.955	0.978	0.963	0.995	1.076	1.046	1.076	3.79E-02
cds.Camellia_sinensis.comp33294_c0_seq29_m.26235	PREDICTED: dynamin-like protein ARC5 [Daucus carota subsp. sativus]	49.426	3.0027	6.9	3	3	3	0.987	1.04	1.124	0.899	0.938	0.998	0.9	9.57E-02

cds.Camellia_sinensis.comp33294_c0_seq38_m.31539	PREDICTED: SNF1-related protein kinase regulatory subunit gamma-1-like [Nicotiana tabacum]	32.685	10.479	15.2	4	5	4	0.824	0.853	0.962	1.111	1.141	1.11	1.274	6.92E-03
cds.Camellia_sinensis.comp33295_c0_seq2_m.24156	PREDICTED: pyruvate dehydrogenase E1 component subunit beta-3, chloroplastic [Juglans regia]	44.743	20.217	5.2	2	3	2	1.053	1.098	0.971	0.878	0.983	1.034	0.927	2.73E-01
cds.Camellia_sinensis.comp33297_c0_seq1_m.7766	PREDICTED: topless-related protein 4-like isoform X2 [Daucus carota subsp. sativus]	99.254	6.082	9.1	8	9	3								

cds.Camellia_sinensis.comp33298_c0_seq14_m.32209	PREDICTED: ubiquitin carboxyl-terminal hydrolase isozyme L3 [Jatropha curcas]	25.461	2.0136	7.4	2	2	2	0.963	0.968	1.004	1.03	0.988	1.064	1.05	1.25E-01
cds.Camellia_sinensis.comp33299_c0_seq1_m.7996	PREDICTED: pentatricopeptide repeat-containing protein At2g37230 [Vitis vinifera]	53.217	3.6998	5.8	3	3	3	1.007	0.998	0.96	0.963	1.075	1.007	1.027	5.03E-01
cds.Camellia_sinensis.comp33300_c0_seq1_m.24462	PREDICTED: UDP-glycosyltransferase 89A2-like isoform X2 [Juglans regia]	53.695	11.497	9.6	4	4	4	0.985	0.99	0.998	0.965	0.988	1.007	0.996	7.44E-01
cds.Camellia_sinensis.comp33300_c0_seq3_m.39256	PREDICTED: DNA-binding protein DDB_G0278111 [Vitis vinifera]	14.856	37.721	18.8	2	3	2	0.929	0.91	1.008	1.037	1.079	1.066	1.118	2.83E-02

cds.Camellia_sinensis.comp33302_c1_seq1_m.8446	PREDICTED: protein transport protein Sec24-like At3g07100 [Vitis vinifera]	83.814	6.0344	4	2	2	2	0.975	0.976	1.031	1.077	0.967	0.992	1.018	6.69E-01
cds.Camellia_sinensis.comp33304_c0_seq2_m.12419	PREDICTED: synaptotagmin-2 [Vitis vinifera]	34.703	34.572	38.6	11	17	8	0.902	1.004	1.016	1.063	1.016	1.042	1.068	1.69E-01
cds.Camellia_sinensis.comp33304_c0_seq2_m.12420	PREDICTED: synaptotagmin-2 [Vitis vinifera]	22.693	13.069	21.2	5	12	3	0.938	0.959	0.996	1.05	1.044	0.99	1.066	6.74E-02
cds.Camellia_sinensis.comp33306_c0_seq15_m.34501	transmembrane protein [Arabidopsis thaliana]	36.872	1.8118	3.6	1	1	1								
cds.Camellia_sinensis.comp33310_c0_seq1_m.2699	PREDICTED: protein CROWDED NUCLEI 4-like [Nicotiana	109.18	19.228	5.2	5	7	5	1.002	0.998	1.014	0.955	1	1.011	0.984	4.18E-01

cds.Camellia_sinensis.comp33310_c0_seq40_m.30096	PREDICTED: protein CROWDED NUCLEI 4-like [Daucus carota subsp.	39.101	4.0506	8.9	2	2	2	1.055	1.03	0.95	0.946	1.019	0.996	0.976	5.65E-01
cds.Camellia_sinensis.comp33312_c0_seq23_m.20101	PREDICTED: ATP-dependent 6-phosphofructokinase 3-like isoform X1 [Juglans regia]	54.198	49.435	30.7	15	21	10	1.032	1.055	1.019	0.979	0.999	0.951	0.943	2.85E-02
cds.Camellia_sinensis.comp33312_c0_seq3_m.6920	PREDICTED: putative ion channel POLLUX-like 2 isoform X2 [Sesamum indicum]	56.638	3.8582	4	2	2	2	1.027	0.935	1.158	0.891	1.05	0.937	0.922	3.71E-01

cds.Camellia_sinensis.comp33312_c0_seq3_m.6921	PREDICTED: putative ion channel POLLUX-like 2 [Ziziphus jujuba]	38.942	25.843	28.8	10	10	10	1.084	1.035	1.058	0.94	0.953	0.964	0.899	2.22E-03
cds.Camellia_sinensis.comp33313_c0_seq9_m.5082	PREDICTED: conserved oligomeric Golgi complex subunit 6 [Vitis vinifera]	56.639	6.6096	6.5	3	3	3	1.123	0.951	1.034	1.006	0.915	0.962	0.928	2.54E-01
cds.Camellia_sinensis.comp33315_c0_seq1_m.7168	PREDICTED: probable ADP-ribosylation factor GTPase-activating protein AGD14 isoform X1 [Juglans regia]	81.628	9.284	5.2	4	4	4	1.161	1.14	1.079	0.85	0.867	0.933	0.784	2.46E-03

cds.Camellia_sinensis.comp33318_c0_seq1_m.2110	PREDICTED: calcium-transporting ATPase 10, plasma membrane-type isoform X1 [Sesamum indicum]	118.25	4.4966	5.6	4	4	4								
cds.Camellia_sinensis.comp33320_c1_seq1_m.27826	PREDICTED: methionine adenosyltransferase 2 subunit beta isoform X1 [Solanum pennellii]	35.571	9.3727	7.4	2	2	2	0.872	1.085	1.041	1.067	1.008	0.955	1.011	8.60E-01
cds.Camellia_sinensis.comp33325_c0_seq1_m.21011	PREDICTED: phospholipase A(1) LCAT3 isoform X2 [Theobroma cacao]	50.15	14.466	6.1	3	3	3	0.981	0.993	1.031	0.991	1.003	1.012	1	9.76E-01

cds.Camellia_sinensis.comp33327_c0_seq1_m.19277	protein FAR1-RELATED SEQUENCE 4-like isoform X2 [Cajanus cajan]	57.422	1.2308	3.5	2	2	2								
cds.Camellia_sinensis.comp33332_c0_seq1_m.3881	PREDICTED: protein STABILIZED1 [Nelumbo nucifera]	73.663	5.7523	6.1	4	4	4	1.039	1.06	1.029	0.952	0.974	0.928	0.912	5.10E-03
cds.Camellia_sinensis.comp33337_c0_seq1_m.13812	PREDICTED: 4-hydroxy-tetrahydrodipicolinate reductase 1, chloroplastic-like isoform X1 [Nelumbo nucifera]	36.246	13.711	14.4	5	6	5	0.999	0.963	0.966	1.074	1.03	1.015	1.065	3.77E-02
cds.Camellia_sinensis.comp33340_c0_seq1_m.3039	PREDICTED: beta-galactosidase-like [Sesamum indicum]	24.029	1.3916	7.5	2	2	1	0.752	0.801	0.884	1.236	1.23	1.179	1.496	1.20E-03

cds.Camellia_sinensis.comp33340_c0_seq2_m.5151	PREDICTED: beta-galactosidase-like [Sesamum indicum]	93.865	9.1505	5.9	5	6	3	0.827	0.932	0.833	1.135	1.213	1.17	1.357	2.08E-03
cds.Camellia_sinensis.comp33345_c1_seq2_m.46861	PREDICTED: hydroxymethylglutaryl-CoA lyase, mitochondrial isoform X3 [Vitis	20.699	1.5141	11.3	2	2	2	1.027	1.042	1.073	1	0.934	0.924	0.91	2.70E-02
cds.Camellia_sinensis.comp33346_c0_seq2_m.36031	PREDICTED: 60 kDa jasmonate-induced protein [Elaeis guineensis	30.07	24.115	25	6	7	6	0.962	0.983	1.011	1.036	0.992	1.018	1.03	1.91E-01
cds.Camellia_sinensis.comp33348_c2_seq1_m.10350	PREDICTED: calmodulin-binding protein 60 B isoform X1 [Nicotiana attenuata]	72.24	24.092	13.1	9	11	7	0.99	1.051	1.114	0.992	0.862	0.968	0.894	1.11E-01

cds.Camellia_sinensis.comp33352_c0_seq1_m.32981	PREDICTED: probable CDP-diacylglycerol--inositol 3-phosphatidyltransferase 2 [Ricinus communis]	30.807	1.3491	3	1	1	1	1.035	0.997	1.035	1.007	0.947	0.981	0.957	1.12E-01
cds.Camellia_sinensis.comp33354_c0_seq1_m.13563	PREDICTED: LOW QUALITY PROTEIN: alkaline/neutral invertase CINV2 [Sesamum indicum]	63.531	41.672	15.5	9	11	8	0.937	0.991	0.988	1.095	1.002	1.06	1.083	6.70E-02
cds.Camellia_sinensis.comp33354_c0_seq2_m.25461	PREDICTED: PTI1-like tyrosine-protein kinase At3g15890 [Prunus mume]	41.299	11.588	14.4	5	5	5	1.018	0.988	1.095	0.979	1.02	0.904	0.936	2.29E-01
cds.Camellia_sinensis.comp33358_c0_seq1_m.2298	PREDICTED: K(+) efflux antiporter 3, chloroplastic [Vitis vinifera]	54.334	21.849	14.8	6	6	6	1.013	1.05	1.025	0.999	1.008	0.91	0.945	1.67E-01

cds.Camellia_sinensis.comp33360_c0_seq1_m.13258	PREDICTED: glucose-1-phosphate adenylyltransferase large subunit 1-like isoform X1 [Juglans regia]	58.663	33.295	13.7	6	9	5	1.193	1.167	1.164	0.899	0.868	0.796	0.727	9.83E-04
cds.Camellia_sinensis.comp33365_c2_seq3_m.23252	PREDICTED: 4-hydroxy-tetrahydrodipicolinate synthase, chloroplastic isoform X2 [Theobroma cacao]	40.84	93.394	19.1	9	15	9	0.996	0.98	0.982	1.01	0.981	1.027	1.02	2.37E-01
cds.Camellia_sinensis.comp33365_c2_seq6_m.29298	PREDICTED: pectinesterase [Vitis vinifera]	50.684	17.205	9.4	4	8	4	1.017	1.04	1.043	0.959	0.992	0.949	0.935	1.27E-02
cds.Camellia_sinensis.comp33370_c0_seq1_m.9312	PREDICTED: protein TolB [Oryza sativa Japonica Group]	79.118	41.568	9.2	5	5	5	0.986	0.971	0.988	1.051	1.039	0.941	1.029	5.19E-01

cds.Camellia_sinensis.comp33371_c0_seq5_m.12685	PREDICTED: chaperonin 60 subunit beta 4, chloroplastic [Vitis vinifera]	55.331	18.182	14.1	7	8	7	1.06	1.054	1.027	0.943	0.949	0.987	0.917	7.22E-03
cds.Camellia_sinensis.comp33373_c0_seq1_m.2756	PREDICTED: guanylate-binding protein 2 [Vitis vinifera]	120.52	101.33	16.7	18	23	14	0.975	1.009	0.985	1.001	0.985	1.024	1.014	4.18E-01
cds.Camellia_sinensis.comp33379_c1_seq5_m.17768	PREDICTED: zinc finger CCCH domain-containing protein ZFN-like [Vitis vinifera]	47.127	7.1706	6.2	3	4	3	0.993	1.039	0.977	1.018	0.978	1.004	0.997	9.04E-01
cds.Camellia_sinensis.comp33379_c1_seq6_m.18136	PREDICTED: argininosuccinate synthase, chloroplastic [Ricinus communis]	28.882	1.8687	33.2	9	12	1	0.861	1.005	1.061	1.073	0.997	1.036	1.061	3.96E-01

cds.Camellia_sinensis.comp33379_c1_seq7_m.18340	PREDICTED: argininosuccinate synthase, chloroplastic isoform X1 [Theobroma cacao]	57.7	162.29	37.3	21	35	6	0.969	1.002	0.958	1.046	0.989	0.987	1.032	2.53E-01
cds.Camellia_sinensis.comp33379_c1_seq9_m.20280	PREDICTED: argininosuccinate synthase, chloroplastic [Nelumbo nucifera]	22.693	-2	31.2	8	12	1								
cds.Camellia_sinensis.comp33379_c3_seq1_m.20752	PREDICTED: 4-coumarate--CoA ligase-like 9 isoform X1 [Lupinus angustifolius]	60.818	11.402	6.2	3	3	3	1.056	1.025	1.01	1.032	0.965	0.915	0.942	1.78E-01
cds.Camellia_sinensis.comp33380_c0_seq5_m.5253	PREDICTED: eukaryotic translation initiation factor 4B1 [Solanum lycopersicum]	24.027	7.8008	3.9	1	1	1	1.257	1.248	1.287	0.725	0.714	0.692	0.562	2.03E-06

cds.Camellia_sinensis.comp33383_c1_seq1_m.7617	PREDICTED: receptor-like protein kinase HERK 1 [Fragaria vesca subsp. vesca]	72.257	1.4273	2.3	2	5	1	0.999	0.949	1.031	0.996	1.053	0.982	1.017	6.17E-01
cds.Camellia_sinensis.comp33383_c2_seq1_m.43851	NAD(P)-binding Rossmann-fold superfamily protein [Arabidopsis thaliana]	26.339	4.4534	11.8	3	4	3	0.971	1.05	1.029	1.033	0.952	0.975	0.97	4.24E-01
cds.Camellia_sinensis.comp33384_c0_seq1_m.27191	PREDICTED: probable uridine nucleosidase 2 [Prunus mume]	34.706	7.0946	7.7	2	2	2								
cds.Camellia_sinensis.comp33389_c0_seq1_m.11693	PREDICTED: RNA-binding protein 39-like [Daucus carota subsp. sativus]	67.271	1.5299	2.6	1	1	1								

cds.Camellia_sinensis.comp33396_c0_seq2_m.31354	PREDICTED: conserved oligomeric Golgi complex subunit 5 [Nelumbo nucifera]	32.801	1.923	7	2	2	2	0.98	1.034	1.115	0.915	0.929	1.025	0.917	1.69E-01
cds.Camellia_sinensis.comp33399_c0_seq6_m.16738	PREDICTED: CTP synthase [Prunus mume]	53.507	1.7602	3.1	2	2	2	0.937	0.958	0.907	1.112	1.129	0.992	1.154	3.19E-02
cds.Camellia_sinensis.comp33403_c4_seq3_m.15130	kinase family protein [Populus trichocarpa]	56.939	31.333	15.3	6	8	4	0.945	0.996	0.954	1.049	1.051	0.998	1.07	4.44E-02
cds.Camellia_sinensis.comp33403_c4_seq5_m.18825	PREDICTED: probable serine/threonine-protein kinase At4g35230 [Ziziphus	56.334	10.209	14.6	6	7	3	0.918	0.941	0.895	1.144	1.08	1.062	1.193	2.76E-03

cds.Camellia_sinensis.comp33403_c4_seq7_m.38162	PREDICTED: probable serine/threonine-protein kinase At5g41260, partial [Sesamum indicum]	20.055	1.2302	9.4	2	2	1								
cds.Camellia_sinensis.comp33404_c0_seq8_m.8014	PREDICTED: protein TIC 62, chloroplastic isoform X1 [Vitis vinifera]	50.288	134.51	33.5	15	34	15	1.028	1.008	0.982	0.966	0.989	1.008	0.982	3.67E-01
cds.Camellia_sinensis.comp33409_c0_seq4_m.12014	PREDICTED: far upstream element-binding protein 1 [Theobroma cacao]	70.69	59.644	23.7	12	24	6	0.986	0.991	0.982	1	1.036	1.051	1.043	4.85E-02
cds.Camellia_sinensis.comp33409_c0_seq7_m.12810	PREDICTED: far upstream element-binding protein 1 [Theobroma cacao]	71.501	171.76	22.8	12	23	6	1.079	0.959	0.942	0.982	1.02	0.994	1.005	8.80E-01

cds.Camellia_sinensis.comp33410_c0_seq3_m.12744	PREDICTED: prohibitin-3, mitochondrial [Vitis vinifera]	30.749	53.744	21.7	6	18	6	0.946	0.933	0.947	1.036	1.055	1.065	1.117	3.01E-04
cds.Camellia_sinensis.comp33412_c0_seq2_m.15285	PREDICTED: pyruvate kinase 1, cytosolic isoform X2 [Juglans regia]	57.48	121.28	36.2	18	35	1								
cds.Camellia_sinensis.comp33412_c0_seq3_m.16128	PREDICTED: pyruvate kinase 1, cytosolic isoform X1 [Ipomoea nil]	57.338	33.495	32.3	15	28	2	0.852	0.987	1.06	1.214	0.948	0.983	1.085	4.73E-01
cds.Camellia_sinensis.comp33412_c0_seq6_m.16895	PREDICTED: pyruvate kinase 1, cytosolic-like [Daucus carota subsp. sativus]	57.701	-2	38	17	34	0								

cds.Camellia_sinensis.comp33412_c0_seq7_m.17502	PREDICTED: pyruvate kinase 1, cytosolic isoform X2 [Juglans regia]	57.52	4.4895	34.7	17	33	0								
cds.Camellia_sinensis.comp33412_c0_seq9_m.18397	PREDICTED: pyruvate kinase 1, cytosolic-like [Daucus carota subsp. sativus]	57.716	70.266	36.4	17	35	0								
cds.Camellia_sinensis.comp33424_c0_seq1_m.14287	PREDICTED: beta-amylase 1, chloroplastic [Sesamum indicum]	64.58	39.285	12.7	6	6	6	0.968	0.96	1.014	1.039	1.039	1.01	1.05	6.69E-02
cds.Camellia_sinensis.comp33437_c0_seq17_c0_seq1_m.14754	PREDICTED: imidazole glycerol phosphate synthase hisHF, chloroplastic [Sesamum indicum]	63.537	88.875	27.1	13	15	13	0.988	1.014	0.973	1.01	0.981	1.014	1.01	5.62E-01

cds.Camellia_sinensis.comp33441_c0_seq4_m.29150	PREDICTED: photosynthetic NDH subunit of subcomplex B 1, chloroplastic [Juglans regia]	44.901	49.762	26.5	9	12	9	1.077	0.982	1.019	1.023	0.975	0.924	0.949	2.60E-01
cds.Camellia_sinensis.comp33447_c0_seq1_m.22646	PREDICTED: protein TRIGALACTOSYLDIACYLGLYCEROL 4, chloroplastic [Vitis vinifera]	51.929	5.0868	3.6	2	2	2								
cds.Camellia_sinensis.comp33448_c0_seq1_m.9535	PREDICTED: NADH dehydrogenase [ubiquinone] flavoprotein 1, mitochondrial [Jatropha curcas]	53.334	113.48	38.9	15	27	15	1.021	1.047	0.988	0.964	0.96	0.981	0.951	4.91E-02

cds.Camellia_sinensis.comp33448_c0_seq2_m.21505	PREDICTED: phosphoglycerate kinase, cytosolic [Vitis vinifera]	42.607	114.92	58.7	24	94	17	0.975	0.94	0.946	1.055	1.063	1.054	1.109	8.78E-04
cds.Camellia_sinensis.comp33451_c0_seq2_m.27864	PREDICTED: tryptophan--tRNA ligase, cytoplasmic [Ipomoea	50.612	60.487	21.5	8	12	8	0.971	0.937	0.972	1.04	1.041	1.007	1.072	1.27E-02
cds.Camellia_sinensis.comp33453_c0_seq1_m.2831	PREDICTED: importin-5-like [Juglans regia]	123.52	106.96	12.4	12	17	9	1.066	1.021	1.024	0.962	0.969	0.965	0.931	3.67E-02
cds.Camellia_sinensis.comp33453_c0_seq2_m.2999	PREDICTED: importin-5 [Beta vulgaris subsp. vulgaris]	123.51	29.723	6.3	7	9	4	1.03	0.865	1.154	0.823	0.996	1.126	0.966	7.86E-01
cds.Camellia_sinensis.comp33459_c0_seq2_m.19681	PREDICTED: uncharacterized membrane protein At1g75140 [Vitis vinifera]	63.594	1.2739	1.2	1	1	1	1.069	1.019	0.905	0.974	0.938	1.091	1.003	9.59E-01

cds.Camellia_sinensis.comp33460_c0_seq1_m.36440	PREDICTED: nicotinamidase 1-like [Juglans regia]	27.077	24.661	18.4	4	7	4	0.956	1.043	1.025	0.968	1.011	1.044	1	9.98E-01
cds.Camellia_sinensis.comp33465_c0_seq1_m.11018	PREDICTED: serine/threonine-protein kinase STY46-like [Nicotiana tabacum]	17.096	2.4822	16.9	2	2	2								
cds.Camellia_sinensis.comp33465_c0_seq3_m.12575	PREDICTED: serine/threonine-protein kinase STY46 isoform X1 [Vitis vinifera]	62.76	1.7899	3.1	1	1	1								
cds.Camellia_sinensis.comp33467_c1_seq1_m.38838	PREDICTED: isoflavone reductase homolog [Arachis ipaensis]	23.598	5.9644	9	2	3	1	0.947	0.993	1.052	0.967	1.068	0.989	1.011	8.16E-01
cds.Camellia_sinensis.comp33469_c0_seq1_m.8798	PREDICTED: nudix hydrolase 3 [Vitis vinifera]	94.186	85.31	23.6	20	37	20	0.966	0.955	0.95	1.04	1.061	1.047	1.096	2.78E-04

cds.Camellia_sinensis.comp33469_c0_seq3_m.35195	PREDICTED: NECAP-like protein CG9132 [Nicotiana tabacum]	33.611	2.7696	8.7	3	4	3	0.904	0.991	0.989	1.05	1.067	1.06	1.102	8.60E-02
cds.Camellia_sinensis.comp33472_c0_seq1_m.4299	PREDICTED: ubiquitin fusion degradation protein 1 homolog isoform X2 [Juglans regia]	35.518	1.618	5.3	2	3	1	1.107	1.086	1.038	0.922	0.896	0.93	0.851	1.88E-03
cds.Camellia_sinensis.comp33473_c0_seq1_m.28691	PREDICTED: probable cinnamyl alcohol dehydrogenase 1 [Arachis duranensis]	39.779	4.3057	39.9	12	33	1	0.972	0.938	0.92	1.019	1.05	1.121	1.127	2.13E-02
cds.Camellia_sinensis.comp33473_c0_seq2_m.31095	PREDICTED: probable cinnamyl alcohol dehydrogenase 1 [Arachis duranensis]	39.763	125.72	39.9	12	32	1	0.847	0.803	0.831	1.175	1.22	1.19	1.445	4.14E-05

cds.Camellia_sinensis.comp33473_c0_seq3_m.41971	PREDICTED: probable cinnamyl alcohol dehydrogenase 1 [Arachis duranensis]	29.866	22.196	17.7	5	12	5	0.929	0.856	0.919	1.096	1.104	1.072	1.21	2.14E-03
cds.Camellia_sinensis.comp33473_c1_seq1_m.11925	PREDICTED: actin-interacting protein 1-2-like [Nicotiana attenuata]	66.021	51.651	23.8	13	17	13	0.977	0.949	0.964	1.051	1.036	1.017	1.074	4.92E-03
cds.Camellia_sinensis.comp33475_c0_seq1_m.5850	PREDICTED: proteinaceous RNase P 1, chloroplastic/mitochondrial [Vitis vinifera]	98.504	-2	1	1	1	1	0.921	0.871	0.929	1.094	1.13	1.096	1.22	9.40E-04
cds.Camellia_sinensis.comp33476_c0_seq3_m.1571	Kinesin-like protein KIF15 [Morus notabilis]	134.78	1.4591	0.9	1	1	1	1.093	1.009	1.145	0.906	0.947	0.884	0.843	1.56E-02
cds.Camellia_sinensis.comp33477_c1_seq1_m.14299	RasGAP-activating-like protein 1 [Morus notabilis]	71.92	1.4804	2.3	2	2	2	1.007	1.097	0.998	0.964	1.031	0.905	0.935	2.32E-01

cds.Camellia_sinensis.comp33484_c0_seq1_m.22585	PREDICTED: golgin subfamily A member 6-like protein 6 isoform X2 [Citrus sinensis]	43.579	17.026	15.9	5	6	3	0.972	1.245	0.96	0.907	1.04	0.873	0.888	3.15E-01
cds.Camellia_sinensis.comp33490_c0_seq2_m.13230	PREDICTED: ubiquitin domain-containing protein DSK2a-like [Ziziphus jujuba]	58.553	72.574	20.4	8	15	7	0.959	0.926	0.993	1.071	1.004	1.071	1.093	3.93E-02
cds.Camellia_sinensis.comp33490_c0_seq6_m.32432	PREDICTED: ubiquitin domain-containing protein DSK2a-like [Juglans regia]	27.796	9.8541	10.2	2	3	2	0.96	1.029	0.916	1.118	0.979	1.022	1.074	2.43E-01
cds.Camellia_sinensis.comp33491_c0_seq1_m.10369	PREDICTED: ATP-dependent zinc metalloprotease FTSH 2, chloroplastic [Vitis vinifera]	74.429	323.31	39.3	26	100	25	1.01	0.984	1.015	1.012	1	0.997	1	9.95E-01

cds.Camellia_sinensis.comp33495_c0_seq1_m.32618	PREDICTED: riboflavin biosynthesis protein PYRD, chloroplastic [Vitis vinifera]	27.785	2.3948	4	1	1	1	1.006	0.992	1.043	0.963	0.893	1.103	0.973	6.52E-01
cds.Camellia_sinensis.comp33495_c0_seq1_m.32619	PREDICTED: riboflavin biosynthesis protein PYRD, chloroplastic [Juglans regia]	13.994	2.8471	16.2	2	2	2								
cds.Camellia_sinensis.comp33498_c0_seq10_m.49841	PREDICTED: pleiotropic drug resistance protein 2-like [Gossypium hirsutum]	21.25	2.4554	21.2	4	4	2	1.085	0.984	0.989	0.946	1.034	0.958	0.961	3.99E-01
cds.Camellia_sinensis.comp33502_c0_seq10_m.7346	PREDICTED: protein ROOT HAIR DEFECTIVE 3 homolog 2 [Jatropha curcas]	92.627	28.688	12.3	10	11	9	0.987	1	1.058	0.999	0.93	0.987	0.958	2.31E-01

cds.Camellia_sinensis.comp33502_c0_seq18_m.39296	PREDICTED: sugar carrier protein C-like [Ipomoea nil]	29.706	1.1983	5.3	2	3	1	0.964	0.961	0.975	1.074	0.983	1.065	1.077	1.25E-01
cds.Camellia_sinensis.comp33502_c0_seq4_m.3342	PREDICTED: sugar carrier protein C-like [Ipomoea nil]	57.517	9.2049	7.7	4	5	3	1.039	0.992	1.006	0.91	1.005	1.046	0.975	5.71E-01
cds.Camellia_sinensis.comp33502_c0_seq4_m.3343	PREDICTED: fructokinase-like 1, chloroplastic [Gossypium raimondii]	56.656	4.6011	9.7	4	4	4								
cds.Camellia_sinensis.comp33502_c0_seq8_m.7124	protein ROOT HAIR DEFECTIVE 3-like [Cajanus cajan]	57.917	2.7256	3.6	2	3	1	0.992	1.005	1.009	1.044	0.937	1.024	1	9.71E-01
cds.Camellia_sinensis.comp33505_c0_seq15_m.522	PREDICTED: trafficking protein particle complex subunit 13 isoform X1 [Vitis vinifera]	49.836	1.814	3.8	2	2	2	1.018	1.017	1.083	0.94	0.911	1.026	0.923	1.20E-01

cds.Camellia_sinensis.comp33506_c1_seq1_m.16384	PREDICTED: dihydrolipoyl dehydrogenase 2, chloroplastic-like isoform X2 [Nicotiana tabacum]	59.902	10.661	10.4	6	7	1								
cds.Camellia_sinensis.comp33506_c1_seq2_m.23251	PREDICTED: 3-isopropylmalate dehydrogenase, chloroplastic isoform X2 [Sesamum indicum]	41.541	100.08	27.2	9	20	5	1.001	0.976	0.97	1.043	0.981	1.049	1.043	1.52E-01
cds.Camellia_sinensis.comp33506_c1_seq5_m.38168	PREDICTED: isocitrate dehydrogenase [NAD] catalytic subunit 5, mitochondrial [Jatropha curcas]	16.264	41.319	19.1	3	5	2	0.962	0.937	0.943	1.038	1.049	1.077	1.113	1.38E-03

cds.Camellia_sinensis.comp33506_c1_seq8_m.53720	PREDICTED: 3-isopropylmalate dehydrogenase, chloroplastic-like [Erythranthe guttata]	16.733	13.354	40.7	4	9	1	0.937	0.834	0.931	1.038	1.055	1.237	1.232	3.73E-02
cds.Camellia_sinensis.comp33515_c0_seq15_m.9639	PREDICTED: protein QUIRKY-like [Nicotiana tabacum]	88.969	22.857	6.2	5	6	5	0.958	1.026	1.043	1.072	0.931	0.986	0.987	7.93E-01
cds.Camellia_sinensis.comp33515_c1_seq13_m.30685	PREDICTED: cytochrome P450 CYP72A219-like isoform X2 [Juglans regia]	45.166	7.1653	2.8	1	1	1	0.858	0.865	0.967	1.145	1.058	1.157	1.249	9.54E-03
cds.Camellia_sinensis.comp33516_c0_seq1_m.500	vacuolar protein sorting protein [Medicago truncatula]	277.54	37.559	5.6	12	12	12	0.9	0.996	0.951	1.011	1.068	1.032	1.093	5.55E-02
cds.Camellia_sinensis.comp33516_c0_seq2_m.904	vacuolar protein sorting protein [Medicago truncatula]	209.64	4.0715	2.2	4	6	4	0.977	1.004	1.046	1.041	0.995	1.011	1.007	7.90E-01

cds.Camellia_sinensis.comp33518_c0_seq1_m.16947	PREDICTED: ribulose bisphosphate carboxylase/oxygenase activase, chloroplastic isoform X1 [Vitis vinifera]	52.007	323.31	64.6	28	229	5	1.055	1.015	1.014	0.99	1.008	0.951	0.956	1.06E-01
cds.Camellia_sinensis.comp33518_c0_seq2_m.16961	PREDICTED: ribulose bisphosphate carboxylase/oxygenase activase, chloroplastic isoform X1 [Vitis vinifera]	52.182	104.4	62.7	27	222	0								
cds.Camellia_sinensis.comp33518_c0_seq3_m.18225	PREDICTED: ribulose bisphosphate carboxylase/oxygenase activase, chloroplastic [Vitis vinifera]	52.204	193.73	56.1	25	170	11	1.039	1.012	1.023	0.967	0.99	0.973	0.953	9.90E-03

cds.Camellia_sinensis.comp33518_c0_seq4_m.18496	PREDICTED: ribulose bisphosphate carboxylase/oxygenase activase, chloroplastic isoform X2 [Vitis vinifera]	48.053	80.308	62.3	25	220	1	1.028	1.079	1.041	0.949	0.98	0.928	0.908	1.07E-02
cds.Camellia_sinensis.comp33519_c0_seq1_m.26361	L-ascorbate peroxidase 3, peroxisomal [Jatropha curcas]	31.925	68.585	38.5	11	27	9	0.991	0.988	0.986	0.984	1.066	1	1.029	3.76E-01
cds.Camellia_sinensis.comp33519_c0_seq2_m.53802	PREDICTED: L-ascorbate peroxidase 3, peroxisomal [Citrus sinensis]	13.35	1.651	19.8	2	7	1								
cds.Camellia_sinensis.comp33521_c0_seq2_m.6031	PREDICTED: U-box domain-containing protein 4 [Theobroma cacao]	73.804	1.263	2	1	1	1								

cds.Camellia_sinensis.comp33522_c1_seq1_m.3043	PREDICTED: beta-fructofuranosidase, soluble isoenzyme I-like isoform X1 [Nelumbo nucifera]	21.882	4.3444	14.1	2	2	2	0.896	0.956	0.981	1.094	1.02	1.088	1.13	2.50E-02
cds.Camellia_sinensis.comp33524_c0_seq8_m.15295	PREDICTED: DNA ligase 1-like [Sesamum indicum]	71.98	1.815	1.5	1	1	1								
cds.Camellia_sinensis.comp33531_c0_seq1_m.1329	PREDICTED: methionine S-methyltransferase isoform X1 [Vitis vinifera]	121.24	4.4648	2.2	3	4	3	1.012	1.028	1.064	0.959	0.99	0.949	0.934	2.45E-02
cds.Camellia_sinensis.comp33545_c2_seq7_m.24297	PREDICTED: long chain acyl-CoA synthetase 4-like [Juglans regia]	15.039	1.2136	9.9	1	1	1								

cds.Camellia_sinensis.comp33546_c0_seq21_m.10170	NADH dehydrogenase subunit 7 (mitochondrion) [Citrullus lanatus]	33.092	124.95	32.1	8	17	8	0.98	0.982	0.975	1.051	1.018	0.99	1.042	1.46E-01
cds.Camellia_sinensis.comp33546_c0_seq33_m.34860	cytochrome c oxidase subunit 2 (mitochondrion) [Beta macrocarp	29.284	11.783	14.8	4	6	4	1.006	0.957	1.013	1.018	1.035	1.002	1.027	2.61E-01
cds.Camellia_sinensis.comp33546_c0_seq8_m.3383	NADH dehydrogenase subunit 2 (mitochondrion) [Nelumbo nucifera]	16.559	13.47	7.4	1	1	1	0.944	0.952	0.977	1.061	1.056	1.036	1.097	1.82E-03
cds.Camellia_sinensis.comp33549_c0_seq17_m.17070	PREDICTED: splicing factor U2af large subunit B isoform X4 [Daucus carota subsp. sativus]	33.167	2.2872	3.3	1	1	1								

cds.Camellia_sinensis.comp33553_c0_seq10_m.22919	PREDICTED: gluconokinase isoform X2 [Citrus sinensis]	42.295	33.831	19.1	6	7	6	0.92	1.018	0.94	1.085	1.043	1.037	1.1	4.76E-02
cds.Camellia_sinensis.comp33554_c0_seq2_m.16296	PREDICTED: FGGY carbohydrate kinase domain-containing protein [Vitis vinifera]	66.651	30.546	13.6	8	13	8	0.935	0.978	0.967	1.033	1.025	0.996	1.06	2.83E-02
cds.Camellia_sinensis.comp33556_c0_seq1_m.6069	PREDICTED: lon protease homolog 2, peroxisomal [Vitis	98.253	3.0766	2.6	2	2	2								
cds.Camellia_sinensis.comp33558_c0_seq5_m.35964	PREDICTED: syntaxin-51-like [Sesamum indicum]	25.715	30.448	29.2	8	14	8	0.961	1.005	0.974	1.027	1.027	1.003	1.04	6.42E-02
cds.Camellia_sinensis.comp33558_c3_seq1_m.40063	PREDICTED: thioredoxin O2, mitochondrial [Sesamum indicum]	16.298	15.794	22.6	3	6	3	1.037	1.037	1.118	0.927	0.887	0.984	0.877	2.80E-02

cds.Camellia_sinensis.comp33561_c1_seq1_m.5928	PREDICTED: nuclear pore complex protein NUP1 [Vitis vinifera]	70.092	1.4038	2.6	2	2	2	1.106	1.026	1.008	0.998	0.895	0.956	0.907	8.40E-02
cds.Camellia_sinensis.comp33562_c0_seq8_m.5046	PREDICTED: riboflavin synthase [Vitis vinifera]	30.636	63.606	39.1	8	13	8	1.056	0.971	0.984	0.938	0.998	0.997	0.974	4.77E-01
cds.Camellia_sinensis.comp33567_c0_seq4_m.25188	transmembrane protein, putative [Medicago truncatula]	35.594	10.409	6.9	2	2	2	1.019	1.188	1.001	0.867	0.953	0.957	0.866	8.75E-02
cds.Camellia_sinensis.comp33568_c0_seq10_m.48518	PREDICTED: cysteine synthase isoform X1 [Glycine max]	15.483	6.5529	13	2	4	1	1.075	1.277	1.072	0.881	0.816	0.846	0.743	8.94E-03
cds.Camellia_sinensis.comp33568_c0_seq4_m.28856	PREDICTED: cysteine synthase isoform X1 [Glycine max]	40.437	74.078	38.2	12	28	10	1.009	1.007	1	0.983	1.025	0.997	0.996	7.78E-01

cds.Camellia_sinensis.comp33570_c0_seq13_m.43530	PREDICTED: adenylosuccinate lyase [Theobroma cacao]	14.693	15.167	31.3	4	6	2	1.002	1.008	1.049	0.932	1	1.012	0.962	2.58E-01
cds.Camellia_sinensis.comp33570_c0_seq3_m.15855	PREDICTED: adenylosuccinate lyase [Theobroma cacao]	61.638	43.604	22.3	12	17	10	0.992	0.964	1.022	1.034	1.003	1.032	1.031	1.96E-01
cds.Camellia_sinensis.comp33576_c0_seq4_m.32648	PREDICTED: chlorophyll(ide) b reductase NOL, chloroplastic isoform X1 [Populus euphratica]	36.34	10.424	19.5	6	7	6	0.98	0.983	0.97	0.999	1.052	1.031	1.051	3.39E-02
cds.Camellia_sinensis.comp33577_c0_seq17_m.10507	PREDICTED: serine/threonine protein phosphatase 2A regulatory subunit B"beta-like [Sesamum indicum]	61.941	46.756	14.9	6	8	6	1.03	0.977	0.93	1.024	1.031	1.018	1.046	2.61E-01

cds.Camellia_sinensis.comp33579_c0_seq9_m.18067	PREDICTED: phosphoinositide phospholipase C 2 [Vitis vinifera]	68.055	54.331	26.3	16	29	16	0.953	0.97	0.959	1.033	1.04	1.022	1.074	6.00E-04
cds.Camellia_sinensis.comp33579_c1_seq1_m.50294	PREDICTED: 14-3-3-like protein GF14 kappa [Nelumbo nucifera]	21.135	90.547	54.8	11	31	8	0.964	0.974	0.908	1.056	1.07	1.087	1.129	6.54E-03
cds.Camellia_sinensis.comp33582_c0_seq6_m.35475	PREDICTED: putative 4-hydroxy-tetrahydrodipicolinate reductase 3, chloroplastic [Populus euphratica]	17.41	12.795	36.2	4	4	4	0.94	1.024	1.031	1.016	0.954	1.05	1.008	8.47E-01
cds.Camellia_sinensis.comp33583_c0_seq17_m.28135	PREDICTED: splicing factor 3A subunit 2-like [Nicotiana sylvestris]	38.298	47.905	16	5	8	5	1.004	1.023	0.994	0.982	0.998	1.01	0.99	4.29E-01

cds.Camellia_sinensis.comp33583_c0_seq1_m.1570	PREDICTED: ABC transporter D family member 1 isoform X2 [Daucus carota subsp. sativus]	151.04	16.629	5.3	8	9	8	1.025	1.068	1.08	0.952	0.935	0.935	0.889	2.32E-03
cds.Camellia_sinensis.comp33588_c0_seq1_m.8929	PREDICTED: gamma-glutamyltranspeptidase 3-like [Juglans regia]	68.035	6.4163	4.3	3	4	3	1.072	1.096	1.088	0.894	0.99	0.944	0.869	9.32E-03
cds.Camellia_sinensis.comp33592_c0_seq1_m.12654	PREDICTED: dolichyl-diphosphooligosaccharide--protein glycosyltransferase subunit 2-like [Juglans regia]	83.854	125.68	29.2	17	31	17	0.994	0.988	0.99	1.041	1.042	1.001	1.038	1.10E-01

cds.Camellia_sinensis.comp33596_c1_seq16_m.36604	PREDICTED: epidermal growth factor receptor substrate 15-like isoform X2 [Nicotiana tomentosiformis]	27.017	5.7515	12.2	2	2	2	1.068	1.119	0.983	0.874	1.017	0.924	0.888	1.10E-01
cds.Camellia_sinensis.comp33596_c1_seq1_m.3182	PREDICTED: epidermal growth factor receptor substrate 15-like 1 [Solanum tuberosum	113.82	6.2309	8.2	8	8	6	1.038	1.119	1.092	0.939	0.936	0.864	0.843	8.20E-03
cds.Camellia_sinensis.comp33596_c1_seq2_m.6425	PREDICTED: epidermal growth factor receptor substrate 15-like [Nelumbo nucifera]	90.117	12.65	9.7	10	10	8	1.069	1.08	1.092	0.864	0.969	0.912	0.847	7.70E-03

cds.Camellia_sinensis.comp33599_c0_seq1_m.28484	PREDICTED: mitochondrial import receptor subunit TOM40-1-like [Nicotiana tabacum]	40.638	5.4752	12	4	4	4	1.134	0.879	0.979	1.153	1.002	0.863	1.009	9.52E-01
cds.Camellia_sinensis.comp33599_c1_seq11_m.43029	PREDICTED: histone H2AX [Citrus sinensis]	14.658	6.2367	25	5	15	2	1.193	1.245	1.316	0.749	0.739	0.704	0.584	9.72E-05
cds.Camellia_sinensis.comp33604_c0_seq4_m.28246	PREDICTED: ubiquitin carboxyl-terminal hydrolase 6 [Vitis vinifera]	45.026	26.945	13.6	5	6	5	0.969	1.075	1.013	1.044	0.85	1.056	0.965	6.33E-01
cds.Camellia_sinensis.comp33605_c0_seq11_m.35919	PREDICTED: glutathione S-transferase L3-like isoform X1 [Nicotiana tabacum]	31.234	56.243	33.8	8	20	5	0.934	0.895	0.869	1.095	1.117	1.122	1.236	6.78E-04

cds.Camellia_sinensis.comp33605_c0_seq4_m.13858	PREDICTED: eukaryotic translation initiation factor 3 subunit H [Vitis vinifera]	38.617	60.538	21.1	6	6	6	0.974	0.962	0.986	1.045	1.036	1.018	1.061	4.96E-03
cds.Camellia_sinensis.comp33605_c0_seq6_m.24251	PREDICTED: glutathione S-transferase L3 [Vitis vinifera]	27.424	182.85	56.1	14	46	12	0.902	0.887	0.904	1.117	1.145	1.125	1.258	1.87E-05
cds.Camellia_sinensis.comp33606_c0_seq1_m.24840	PREDICTED: probable indole-3-acetic acid-amido synthetase GH3.6 [Juglans regia]	58.518	6.0999	7.1	3	3	3	0.841	0.973	0.996	1.005	1.129	1.093	1.148	9.14E-02
cds.Camellia_sinensis.comp33611_c0_seq1_m.11426	PREDICTED: glycerol-3-phosphate dehydrogenase SDP6, mitochondrial-like [Sesamum indicum]	71.729	96.499	20.3	12	16	12	0.987	0.986	0.986	1.047	1.006	1.008	1.034	1.22E-01

cds.Camellia_sinensis.comp33613_c0_seq1_m.2133	PREDICTED: protein TSS [Vitis vinifera]	160.29	3.4852	2.6	4	4	0								
cds.Camellia_sinensis.comp33613_c0_seq3_m.4842	PREDICTED: protein TSS [Vitis vinifera]	101.64	20.313	6.1	6	7	4	1.187	1.114	1.098	0.867	0.789	0.876	0.745	1.98E-03
cds.Camellia_sinensis.comp33613_c0_seq8_m.14007	PREDICTED: protein TSS [Vitis vinifera]	11.895	1.7316	10.1	1	1	1								
cds.Camellia_sinensis.comp33614_c1_seq1_m.4575	PREDICTED: dynamin-2A-like [Nicotiana tabacum]	63.783	77	18.1	10	11	10	1.142	1.167	1.152	0.833	0.78	0.849	0.711	2.04E-04
cds.Camellia_sinensis.comp33614_c1_seq4_m.11953	PREDICTED: LOW QUALITY PROTEIN: dynamin-2A-like [Ziziphus jujuba]	25.277	3.5301	15	3	3	3	1.22	1.199	1.178	0.78	0.797	0.766	0.651	4.81E-06
cds.Camellia_sinensis.comp33616_c0_seq9_m.1487	PREDICTED: ABC transporter C family member 14 [Juglans	38.954	11.198	7.2	2	2	2	1.047	1.09	0.998	0.99	0.901	0.967	0.912	7.18E-02

cds.Camellia_sinensis.comp33617_c0_seq3_m.36051	PREDICTED: hippocampus abundant transcript-like protein 1 isoform X3 [Nicotiana tabacum]	17.546	5.5301	10.2	1	1	1								
cds.Camellia_sinensis.comp33622_c1_seq1_m.39647	esterase/lipase/thioesterase family protein [Populus trichocarpa]	24.368	16.259	32.4	7	10	7	0.973	1.004	1.003	1.029	1.025	0.956	1.01	7.32E-01
cds.Camellia_sinensis.comp33623_c0_seq1_m.17480	PREDICTED: RNA-binding protein 1 isoform X1 [Vitis vinifera]	24.198	43.399	21.6	4	9	4	1.001	1.031	1.027	0.938	0.94	0.944	0.923	1.04E-03
cds.Camellia_sinensis.comp33624_c0_seq11_m.49384	PREDICTED: NAD-dependent malic enzyme 62 kDa isoform, mitochondrial [Jatropha curcas]	15.224	1.5362	49.6	6	17	1	0.957	0.958	0.995	1.037	1.069	1.007	1.07	3.51E-02

cds.Camellia_sinensis.comp33624_c0_seq1_m.8477	PREDICTED: ubiquitin receptor RAD23c [Vitis vinifera]	40.93	14.004	24.6	7	15	3	1	1.031	0.987	1.029	0.987	0.957	0.985	5.69E-01
cds.Camellia_sinensis.comp33624_c0_seq2_m.10448	PREDICTED: NAD-dependent malic enzyme 62 kDa isoform, mitochondrial [Jatropha curcas]	69.135	63.189	26.8	15	28	10	1.002	0.973	1.035	1.001	0.963	0.989	0.981	4.22E-01
cds.Camellia_sinensis.comp33625_c1_seq15_m.31691	PREDICTED: NADH-cytochrome b5 reductase-like protein [Nelumbo nucifera]	36.397	38.217	23.4	9	17	9	1.033	1.002	0.979	0.998	1.001	0.996	0.994	7.34E-01
cds.Camellia_sinensis.comp33625_c2_seq45_m.19700	PREDICTED: protein LST8 homolog isoform X1 [Sesamum indicum]	19.682	3.066	10.9	2	2	2	0.98	0.992	0.937	1.127	0.968	1.018	1.07	2.41E-01

cds.Camellia_sinensis.comp33629_c0_seq1_m.23642	probable E3 ubiquitin-protein ligase HERC1-like [Vitis vinifera]	31.952	82.445	50.3	11	16	11	0.892	0.94	0.985	1.044	1.081	1.078	1.137	1.37E-02
cds.Camellia_sinensis.comp33629_c1_seq1_m.6279	PREDICTED: alpha-glucan phosphorylase, H isozyme [Ricinus communis]	73.512	122.77	24.8	13	32	12	0.957	0.975	0.99	1.017	1.067	1.023	1.063	2.73E-02
cds.Camellia_sinensis.comp33629_c1_seq1_m.6280	PREDICTED: alpha-glucan phosphorylase, H isozyme [Gossypium arboreum]	25.645	73.265	48.9	8	10	8	1.007	0.956	0.95	0.991	1.064	1.017	1.055	1.29E-01
cds.Camellia_sinensis.comp33638_c0_seq2_m.512	PREDICTED: CCR4-NOT transcription complex subunit 1 [Nicotiana attenuata]	262.71	22.537	2.9	7	8	7	0.886	0.937	0.994	1.091	1.049	1.051	1.133	2.42E-02

cds.Camellia_sinensis.comp33644_c0_seq1_m.1783	PREDICTED: ethylene-insensitive protein 2 [Vitis vinifera]	141.7	2.5473	1.5	2	2	2	1.005	1.157	0.98	0.94	0.986	0.928	0.908	1.63E-01
cds.Camellia_sinensis.comp33650_c1_seq2_m.10063	PREDICTED: protein LYK5 [Vitis vinifera]	75.823	3.4138	3	2	2	2	1.046	1.084	0.978	0.892	0.988	1.001	0.927	1.82E-01
cds.Camellia_sinensis.comp33650_c1_seq7_m.35695	PREDICTED: ferritin-3, chloroplastic [Theobroma cacao]	30.802	78.547	34.3	9	24	8	0.851	0.853	0.872	1.126	1.196	1.144	1.345	1.16E-04
cds.Camellia_sinensis.comp33653_c0_seq15_m.32110	PREDICTED: probable protein phosphatase 2C 11 isoform X2 [Nicotiana tabacum]	31.382	51.334	29.1	7	8	5	0.961	1.051	0.955	1.07	0.937	1.068	1.036	5.52E-01
cds.Camellia_sinensis.comp33654_c0_seq3_m.37515	PREDICTED: glycylpeptide N-tetradecanoyltransferase 1-like [Jatropha curcas]	33.858	14.953	10.1	3	5	3	0.934	1.002	0.941	1.038	1.072	1.056	1.1	1.64E-02

cds.Camellia_sinensis.comp33656_c0_seq1_m.9924	PREDICTED: hydroquinone glucosyltransferase [Jatropha curcas]	55.279	10.737	6.8	4	6	1	1.097	1.011	1.011	0.951	0.962	0.958	0.92	9.58E-02
cds.Camellia_sinensis.comp33656_c0_seq6_m.24112	PREDICTED: hydroquinone glucosyltransferase [Jatropha curcas]	55.305	7.9968	10.3	6	8	2	0.948	0.961	0.979	1.016	0.992	1.123	1.084	1.12E-01
cds.Camellia_sinensis.comp33659_c0_seq2_m.20927	glutamine synthetase cytosolic isozyme 1 [Gossypium hirsutum]	41.319	156.74	53	16	76	8	1.061	1.015	0.977	0.983	0.954	0.986	0.957	1.74E-01
cds.Camellia_sinensis.comp33659_c1_seq3_m.35435	PREDICTED: acylpyruvase FAHD1, mitochondrial [Ricinus communis]	29.712	11.224	15.8	5	10	5	0.996	0.985	0.979	1.003	1.004	1.042	1.03	9.47E-02
cds.Camellia_sinensis.comp33665_c2_seq27_m.12080	PREDICTED: monosaccharide-sensing protein 2 [Ricinus communis]	80.555	6.5353	3.5	2	2	2	0.969	1.148	1.149	0.857	0.868	0.995	0.833	6.99E-02

cds.Camellia_sinensis.comp33666_c0_seq1_m.13386	PREDICTED: serine/threonine-protein phosphatase BSL1 [Vitis vinifera]	71.364	13.607	11.1	7	7	1	0.961	1.007	1.021	1.035	0.988	1.004	1.013	6.06E-01
cds.Camellia_sinensis.comp33666_c0_seq2_m.13614	PREDICTED: serine/threonine-protein phosphatase BSL1 [Vitis vinifera]	71.134	33.019	12.8	8	8	2	1.024	0.979	0.963	1.002	1.026	1.015	1.026	2.56E-01
cds.Camellia_sinensis.comp33668_c0_seq2_m.14633	PREDICTED: T-complex protein 1 subunit gamma [Nelumbo nucifera]	60.595	85.649	41.7	23	36	23	0.917	0.935	0.95	1.042	1.083	1.053	1.134	1.22E-03
cds.Camellia_sinensis.comp33672_c0_seq1_m.4477	PREDICTED: puromycin-sensitive aminopeptidase isoform X1 [Vitis vinifera]	111.03	323.31	42.5	38	71	33	0.996	0.999	1.014	1.006	1.017	1.005	1.006	4.01E-01

cds.Camellia_sinensis.comp33672_c0_seq2_m.32085	PREDICTED: puromycin-sensitive aminopeptidase [Theobroma cacao]	35.749	107.86	48.9	13	28	9	0.943	1.002	0.947	1.077	1.004	1.014	1.07	8.25E-02
cds.Camellia_sinensis.comp33673_c0_seq2_m.27338	PREDICTED: probable inactive purple acid phosphatase 29 [Jatropha curcas]	42.338	11.937	14.7	6	14	6	1.039	1.034	0.993	0.978	0.982	1.006	0.967	1.21E-01
cds.Camellia_sinensis.comp33674_c0_seq1_m.21430	PREDICTED: serine hydroxymethyltransferase 4 [Sesamum indicum]	53.634	287.31	48.1	23	56	23	1.063	1.042	1.029	0.96	0.963	0.946	0.915	1.28E-03
cds.Camellia_sinensis.comp33674_c1_seq1_m.33814	PREDICTED: annexin D5-like isoform X1 [Gossypium raimondii]	36.828	8.9928	13.6	5	6	5	0.911	0.933	0.926	1.089	1.13	1.091	1.195	2.18E-04

cds.Camellia_sinensis.comp33677_c0_seq1_m.35450	PREDICTED: probable glutathione S-transferase [Gossypium raimondii]	24.87	48.903	36.5	10	21	5	0.984	0.948	0.942	1.047	1.071	1.049	1.102	3.24E-03
cds.Camellia_sinensis.comp33677_c0_seq3_m.39927	PREDICTED: probable glutathione S-transferase [Gossypium raimondii]	24.968	2.0858	7.2	2	3	1	0.95	0.865	0.984	1.095	1.21	0.936	1.158	1.65E-01
cds.Camellia_sinensis.comp33679_c0_seq1_m.30047	PREDICTED: protein transport protein SEC13 homolog B [Vitis vinifera]	32.584	48.755	25.6	6	10	4	0.906	1.013	0.99	1.023	1.055	1.025	1.067	1.38E-01
cds.Camellia_sinensis.comp33680_c1_seq10_c1_seq1_m.21491	PREDICTED: DEAD-box ATP-dependent RNA helicase 8 [Vitis vinifera]	51.714	22.726	19.5	8	11	3	0.984	0.985	0.97	1	1.014	1.031	1.036	2.50E-02

cds.Camellia_sinensis.comp33680_c2_seq3_m.23462	PREDICTED: 26S proteasome non-ATPase regulatory subunit 4 homolog [Vitis vinifera]	44.876	70.859	27.2	8	15	8	1.022	1.022	0.992	1.007	0.947	1.035	0.985	5.95E-01
cds.Camellia_sinensis.comp33685_c0_seq1_m.434	PREDICTED: MAG2-interacting protein 2 isoform X1 [Vitis vinifera]	274.48	1.1904	0.4	1	1	1								
cds.Camellia_sinensis.comp33690_c0_seq1_m.25763	PREDICTED: 40S ribosomal protein S4-like [Ziziphus jujuba]	29.877	119.54	53	14	30	10	1.147	1.13	1.143	0.84	0.875	0.846	0.749	2.35E-05
cds.Camellia_sinensis.comp33691_c0_seq1_m.1922	PREDICTED: cullin-associated NEDD8-dissociated protein 1 [Vitis vinifera]	134.6	7.3362	4.4	5	5	5	0.976	1.015	1.053	0.968	0.939	1.055	0.973	5.33E-01

cds.Camellia_sinensis.comp33691_c1_seq3_m.8486	PREDICTED: leucine-rich repeat extensin-like protein 2 [Theobroma cacao]	78.473	1.1971	1.9	1	1	1	0.837	1.024	0.881	1.156	1.063	1.088	1.206	4.37E-02
cds.Camellia_sinensis.comp33693_c0_seq1_m.8033	PREDICTED: sucrose synthase 7 isoform X3 [Vitis vinifera]	69.517	7.497	8.3	5	8	4	0.844	0.917	0.887	1.189	1.072	1.099	1.269	3.82E-03
cds.Camellia_sinensis.comp33693_c0_seq1_m.8034	PREDICTED: sucrose synthase 7-like [Jatropha curcas]	35.542	2.9563	10.9	4	4	3	0.965	0.9	0.976	1.003	1.088	1.083	1.117	3.88E-02
cds.Camellia_sinensis.comp33694_c0_seq1_m.21873	plant/F20D21-34 protein [Medicago truncatula]	42.817	26.193	13.7	6	9	6	0.925	0.904	0.952	1.084	1.062	1.109	1.17	1.28E-03
cds.Camellia_sinensis.comp33697_c0_seq2_m.4997	PREDICTED: mannose-1-phosphate guanyltransferase alpha-like [Juglans regia]	45.559	7.0289	8	3	4	2	0.884	0.854	0.966	1.068	1.074	1.072	1.189	4.17E-02

cds.Camellia_sinensis.comp33702_c0_seq2_m.7924	PREDICTED: uncharacterized TPR repeat-containing protein At1g05150-like [Ipomoea nil]	90.324	22.367	9.3	7	9	7	0.988	1.029	1.051	0.953	0.963	0.975	0.942	3.75E-02
cds.Camellia_sinensis.comp33702_c0_seq3_m.51726	PREDICTED: cyprosin-like [Nicotiana attenuata]	12.418	1.8133	8	1	2	1	1.071	1.147	1.079	0.926	0.882	0.875	0.814	1.82E-03
cds.Camellia_sinensis.comp33705_c2_seq1_m.6268	PREDICTED: L-arabinokinase-like [Gossypium arboreum]	110.46	68.209	11.8	11	11	11	0.921	0.961	0.914	1.093	1.077	1.015	1.139	9.10E-03
cds.Camellia_sinensis.comp33707_c0_seq1_m.23833	DNA-directed RNA polymerase subunit alpha [Arabidopsis thaliana]	46.089	55.689	20.5	7	8	7	0.991	1.031	1.008	1.002	0.988	0.993	0.984	2.72E-01

cds.Camellia_sinensis.comp33711_c1_seq11_m.40423	PREDICTED: DNA-directed RNA polymerase V subunit 7 [Vitis	20.286	3.8428	14	2	2	2	0.941	0.966	0.98	1.052	1.054	1.033	1.087	3.44E-03
cds.Camellia_sinensis.comp33711_c1_seq2_m.23785	PREDICTED: methionine aminopeptidase 2B isoform X2 [Vitis vinifera]	32.046	1.9604	6.2	2	2	2	1.076	1.059	1.101	0.919	0.938	0.893	0.85	8.61E-04
cds.Camellia_sinensis.comp33714_c0_seq4_m.5479	PREDICTED: probable NOT transcription complex subunit VIP2 isoform X1 [Theobroma cacao]	70.871	3.2544	3.3	2	2	2	0.947	0.966	0.963	1.059	1.02	1.069	1.095	4.44E-03
cds.Camellia_sinensis.comp33717_c0_seq1_m.15910	PREDICTED: 3-ketoacyl-CoA thiolase 2, peroxisomal isoform X1 [Erythranthe guttata]	54.836	135.52	32.2	14	29	12	1.011	0.972	1.003	1.017	1.006	1.032	1.023	1.79E-01

cds.Camellia_sinensis.comp33718_c0_seq2_m.8004	PREDICTED: protein transport protein SEC23 [Vitis vinifera]	84.491	43.856	12.6	8	16	7	0.969	0.951	0.981	1.013	1.031	1.042	1.064	7.18E-03
cds.Camellia_sinensis.comp33727_c0_seq39_m.19053	aminopeptidase (DUF3754) [Arabidopsis thaliana]	41.023	8.1704	7.3	3	3	3	1.03	1.007	1.078	0.993	0.972	0.922	0.927	6.30E-02
cds.Camellia_sinensis.comp33729_c0_seq2_m.7772	PREDICTED: uncharacterized aarF domain-containing protein kinase At1g79600, chloroplastic isoform X2[Gossypium raimondii]	64.802	1.2861	1.7	1	1	1	1.045	1.042	0.965	1.074	0.945	0.936	0.968	5.49E-01
cds.Camellia_sinensis.comp33730_c0_seq16_m.33689	PREDICTED: acetyl-CoA acetyltransferase, cytosolic 1-like [Nicotiana attenuata]	30.582	23.631	23.4	5	8	5	0.984	0.983	1.072	0.986	1.006	1.074	1.009	8.27E-01

cds.Camellia_sinensis.comp33730_c0_seq3_m.22338	PREDICTED: uroporphyrinogen decarboxylase 1, chloroplastic [Vitis vinifera]	46.487	8.6837	9.5	4	8	4	0.998	0.98	1.023	1.041	0.968	0.959	0.989	7.08E-01
cds.Camellia_sinensis.comp33730_c0_seq7_m.25290	PREDICTED: acetyl-CoA acetyltransferase, cytosolic 1-like isoform X2 [Nelumbo nucifera]	23.859	24.556	30.5	5	9	5	0.987	0.934	0.978	0.993	1.056	1.032	1.063	6.99E-02
cds.Camellia_sinensis.comp33730_c1_seq1_m.45250	PREDICTED: acetyl-CoA acetyltransferase, cytosolic 1 isoform X1 [Pyrus x bretschneideri]	18.638	52.128	32.4	5	7	1	0.99	1.047	1.031	0.962	1.007	0.968	0.957	1.19E-01

cds.Camellia_sinensis.comp33730_c1_seq2_m.46525	PREDICTED: acetyl-CoA acetyltransferase, cytosolic 1 [Sesamum indicum]	16.163	2.0639	15.3	3	3	2	0.968	1.068	0.982	1.041	0.968	0.986	0.992	8.58E-01
cds.Camellia_sinensis.comp33730_c1_seq3_m.47110	PREDICTED: acetyl-CoA acetyltransferase, cytosolic 1 isoform X1 [Pyrus x bretschneideri]	18.589	6.5008	32.4	5	7	1	0.95	1.057	0.993	0.984	1.044	0.986	1.005	8.93E-01
cds.Camellia_sinensis.comp33735_c0_seq6_m.39680	PREDICTED: short-chain dehydrogenase TIC 32, chloroplastic isoform X2 [Vitis vinifera]	29.874	2.9154	4.8	1	1	1	0.954	0.943	1.07	1.132	0.986	0.942	1.031	6.89E-01

cds.Camellia_sinensis.comp33736_c0_seq1_m.12198	PREDICTED: ATP-citrate synthase beta chain protein 2-like [Gossypium hirsutum]	65.798	162.57	41.3	21	39	21	1.071	1.085	1.08	0.948	0.937	0.919	0.867	1.24E-04
cds.Camellia_sinensis.comp33739_c0_seq1_m.15479	PREDICTED: protein PHOSPHATE STARVATION RESPONSE 1 isoform X2 [Nelumbo nucifera]	54.187	3.5745	6.5	3	3	3	1.038	1.024	0.838	0.965	1.071	1.071	1.071	4.03E-01
cds.Camellia_sinensis.comp33740_c0_seq1_m.40906	PREDICTED: putative quinone-oxidoreductase homolog, chloroplastic [Prunus mume]	25.49	12.034	21.7	5	10	5	0.922	0.895	0.9	1.147	1.088	1.11	1.231	3.17E-04

cds.Camellia_sinensis.comp33740_c0_seq4_m.44344	PREDICTED: putative quinone-oxidoreductase homolog, chloroplastic [Vitis vinifera]	26.873	48.773	37.5	8	16	8	0.76	0.787	0.842	1.169	1.224	1.244	1.522	2.83E-04
cds.Camellia_sinensis.comp33751_c0_seq1_m.26198	PREDICTED: protein AUXIN RESPONSE 4-like [Nicotiana tabacum]	49.845	10.701	11.4	4	6	4	0.984	0.998	1.024	1.015	0.897	0.987	0.964	3.93E-01
cds.Camellia_sinensis.comp33753_c0_seq3_m.13069	PREDICTED: transmembrane 9 superfamily member 1 [Daucus carota subsp. sativus]	73.379	28.64	8.7	5	8	5	1.023	1.014	1.108	0.931	0.961	0.935	0.899	2.40E-02
cds.Camellia_sinensis.comp33754_c0_seq1_m.2989	Calcium-transporting ATPase 2, plasma membrane-type [Morus notabilis]	110.65	21.117	7.9	9	11	7	0.972	0.969	1.014	1.019	1.061	0.989	1.039	2.08E-01

cds.Camellia_sinensis.comp33758_c0_seq3_m.2481	PREDICTED: protein lava lamp isoform X5 [Vitis vinifera]	67.343	23.233	7.1	4	5	4	1.002	1.079	0.98	0.94	1.023	1.008	0.971	4.90E-01
cds.Camellia_sinensis.comp33758_c0_seq6_m.11488	PREDICTED: golgin subfamily B member 1 isoform X3 [Vitis vinifera]	90.128	1.2589	1.2	1	1	1								
cds.Camellia_sinensis.comp33758_c0_seq7_m.35167	PREDICTED: protein lava lamp isoform X5 [Vitis vinifera]	40.89	2.5239	3.2	1	1	1								
cds.Camellia_sinensis.comp33759_c0_seq1_m.17652	PREDICTED: glycerophosphodiester phosphodiesterase GDPD6 [Prunus mume]	46.132	48.826	22.7	7	13	7	1.023	0.985	1.047	1.006	0.977	0.97	0.967	1.83E-01
cds.Camellia_sinensis.comp33763_c1_seq1_m.2508	PREDICTED: abscisic-aldehyde oxidase isoform X1 [Vitis vinifera]	149.07	3.1233	2.3	3	3	3								

cds.Camellia_sinensis.comp33765_c1_seq1_m.22427	PREDICTED: metal tolerance protein 4-like [Daucus carota subsp. sativus]	46.275	9.8506	9.2	3	3	3	1.007	0.957	0.989	1.022	0.991	1.047	1.036	1.78E-01
cds.Camellia_sinensis.comp33768_c0_seq1_m.12313	PREDICTED: probable methylenetetrahydrofolate reductase [Sesamum indicum]	67.159	77.244	29.8	14	24	14	1.058	1.062	1.063	0.906	0.939	0.922	0.869	5.50E-03
cds.Camellia_sinensis.comp33771_c0_seq10_m.21552	PREDICTED: developmentally-regulated G-protein 2 [Jatropha curcas]	44.539	8.8989	16.8	6	6	6	1.013	0.967	1.106	0.97	0.916	0.89	0.9	8.84E-02
cds.Camellia_sinensis.comp33771_c0_seq4_m.2898	PREDICTED: protein MLP1 [Prunus mume]	89.027	37.497	13.4	10	13	10	1.077	1.111	1.101	0.902	0.865	0.905	0.812	2.75E-04

cds.Camellia_sinensis.comp33774_c0_seq1_m.647	PREDICTED: DExH-box ATP-dependent RNA helicase DExH14 [Juglans regia]	236.14	3.3879	0.8	2	2	2	1.028	1.059	1.045	0.916	0.942	1.003	0.913	3.18E-02
cds.Camellia_sinensis.comp33775_c0_seq12_m.43731	PREDICTED: phenazine biosynthesis-like domain-containing protein 1 [Sesamum indicum]	28.619	9.3833	16.7	3	4	3	1.021	0.996	0.866	1.029	1.084	1.018	1.086	1.97E-01
cds.Camellia_sinensis.comp33776_c0_seq1_m.19252	PREDICTED: (-)-alpha-terpineol synthase [Vitis vinifera]	70.02	47.141	12.2	7	10	7	1.351	1.265	1.304	0.639	0.691	0.651	0.505	2.15E-05
cds.Camellia_sinensis.comp33777_c0_seq27_m.11553	PREDICTED: NADP-dependent malic enzyme [Jatropha curcas]	63.849	124.66	33.2	18	49	11	0.902	0.908	0.899	1.109	1.12	1.11	1.233	5.36E-07

cds.Camellia_sinensis.comp33777_c0_seq5_m.13395	PREDICTED: NADP-dependent malic enzyme isoform X1 [Elaeis guineensis	63.776	-2	15.9	8	21	0								
cds.Camellia_sinensis.comp33779_c0_seq1_m.5726	AMP deaminase [Morus notabilis]	97.692	5.8963	2.9	2	2	2	1.123	1.099	0.996	0.916	0.915	0.929	0.858	5.50E-02
cds.Camellia_sinensis.comp33780_c0_seq6_m.35358	NF-kappa-B inhibitor-like protein [Arabidopsis thaliana]	20.06	8.9984	11.8	2	3	2	0.957	1.037	0.984	1.002	0.966	1.066	1.019	6.47E-01
cds.Camellia_sinensis.comp33784_c0_seq4_m.22630	Bifunctional aspartokinase/homoserine dehydrogenase [Morus notabilis]	53.058	1.4244	32.2	11	21	1	0.984	0.999	1.068	0.94	1.017	0.998	0.969	4.09E-01
cds.Camellia_sinensis.comp33785_c0_seq2_m.24838	PREDICTED: caffeine synthase 1 [Theobroma cacao]	41.41	21.465	5.1	2	6	2	1.076	1.107	1.075	0.914	0.928	0.879	0.835	6.61E-04

cds.Camellia_sinensis.comp33788_c0_seq2_m.6647	PREDICTED: linoleate 13S-lipoxygenase 2-1, chloroplastic-like [Ziziphus jujuba]	103	252.63	42.9	41	115	0								
cds.Camellia_sinensis.comp33788_c0_seq3_m.6645	PREDICTED: linoleate 13S-lipoxygenase 2-1, chloroplastic [Citrus sinensis]	103.18	323.31	56.1	52	187	37	0.999	0.978	0.983	1.012	1.028	1.012	1.031	2.08E-02
cds.Camellia_sinensis.comp33790_c0_seq27_m.37002	PREDICTED: 6-phosphofructo-2-kinase/fructose-2,6-bisphosphatase [Vitis	30.61	1.3112	12.4	3	3	1								
cds.Camellia_sinensis.comp33792_c0_seq12_m.25225	PREDICTED: centromere protein O isoform X2 [Gossypium raimondii]	15.471	1.2867	5.2	1	1	1	0.889	0.931	1.019	0.919	1.153	1.116	1.123	2.38E-01

cds.Camellia_sinensis.comp33793_c0_seq9_m.50887	PREDICTED: ATPase 11, plasma membrane-type-like, partial [Ziziphus jujuba]	19.483	-2	26.1	4	7	1	1.16	1.111	1.11	0.872	0.906	0.806	0.764	1.96E-03
cds.Camellia_sinensis.comp33794_c0_seq1_m.11067	PREDICTED: GDP-L-galactose phosphorylase 2-like [Juglans regia]	50.598	1.471	2	1	1	1	1.016	0.987	1.034	0.955	1.017	0.996	0.977	3.70E-01
cds.Camellia_sinensis.comp33805_c0_seq1_m.3088	PREDICTED: peptidyl-prolyl cis-trans isomerase CYP26-2, chloroplastic [Daucus carota subsp. sativus]	42.714	30.237	17.9	7	14	7	0.957	0.963	0.986	1.032	1.006	1.007	1.048	1.93E-02

cds.Camellia_sinensis.comp33810_c0_seq12_m.16459	PREDICTED: peptide-N(4)-(N-acetyl-beta-glucosaminyl)asparagine amidase-like isoform X3 [Nicotiana tabacum]	47.329	34.231	12	5	8	5	0.982	0.967	1.035	0.961	1.046	0.992	1.005	8.88E-01
cds.Camellia_sinensis.comp33810_c0_seq16_m.21013	PREDICTED: elongation factor Tu, mitochondrial [Ziziphus jujuba]	49.151	4.0318	26	10	34	1		0.969						
cds.Camellia_sinensis.comp33810_c0_seq17_m.21163	PREDICTED: elongation factor Tu, mitochondrial [Ziziphus jujuba]	49.41	183.58	58.3	23	59	13	1.002	0.997	0.971	1.008	0.98	1.036	1.018	3.95E-01
cds.Camellia_sinensis.comp33816_c0_seq1_m.11403	PREDICTED: probable methyltransferase PMT3 [Sesamum indicum]	70.795	11.198	10.5	7	7	7	1.136	1.095	1.13	0.916	0.917	0.858	0.801	8.56E-04

cds.Camellia_sinensis.comp33821_c0_seq1_m.3784	PREDICTED: calmodulin-binding transcription activator 4 isoform X4 [Vitis vinifera]	102.32	1.9756	0.9	1	1	1	1.144	0.939	1.252	0.854	0.78	0.998	0.789	1.03E-01
cds.Camellia_sinensis.comp33824_c0_seq2_m.36478	PREDICTED: uncharacterized mitochondrial protein AtMg00820-like [Solanum pennellii]	13.265	1.1996	7.8	1	1	1	0.38	1.355	1.338	1.39	0.9	0.736	0.985	8.44E-01
cds.Camellia_sinensis.comp33827_c0_seq17_m.7425	triacylglycerol lipase, putative [Medicago truncatula]	97.312	35.235	11.9	10	18	10	0.997	0.99	0.975	1.011	0.989	0.993	1.01	3.32E-01
cds.Camellia_sinensis.comp33830_c0_seq40_m.37216	PREDICTED: soluble inorganic pyrophosphatase 2 isoform X1 [Ziziphus jujuba]	24.22	36.447	27.8	6	14	4	1.052	1.013	0.966	0.931	1.033	0.987	0.974	5.25E-01

cds.Camellia_sinensis.comp33832_c0_seq3_m.4772	PREDICTED: vacuolar protein sorting-associated protein 18 homolog [Citrus sinensis]	40.004	4.7417	6.9	3	4	3	0.958	0.988	0.999	1.018	1.001	1.032	1.036	8.19E-02
cds.Camellia_sinensis.comp33832_c0_seq7_m.15327	PREDICTED: vacuolar protein sorting-associated protein 18 homolog [Vitis vinifera]	24.52	1.9945	4	1	1	1	1.06	1.028	0.926	1.028	1.009	0.952	0.992	8.86E-01
cds.Camellia_sinensis.comp33833_c0_seq1_m.1745	PREDICTED: phytochrome B [Nicotiana attenuata]	92.778	2.3612	2	1	1	1	0.828	1.039	1.07	1.005	1.032	1.057	1.053	5.18E-01
cds.Camellia_sinensis.comp33835_c0_seq1_m.2917	PREDICTED: calmodulin-binding transcription activator 3 isoform X2 [Vitis vinifera]	101.35	3.8512	3.1	2	3	2	1.042	1.343	1.269	0.742	0.729	0.825	0.628	5.88E-03

cds.Camellia_sinensis.comp33837_c0_seq1_m.1657	PREDICTED: ABC transporter C family member 3 [Juglans regia]	162.49	2.4264	1.7	2	3	1								
cds.Camellia_sinensis.comp33844_c0_seq1_m.7097	PREDICTED: urease isoform X1 [Vitis vinifera]	89.823	12.235	6.2	6	6	6	0.925	0.972	1.021	1.076	1.008	1.043	1.072	1.11E-01
cds.Camellia_sinensis.comp33848_c0_seq22_m.32635	PREDICTED: serine carboxypeptidase-like 34 [Nicotiana attenuata]	43.487	9.0222	6.8	2	4	2								
cds.Camellia_sinensis.comp33848_c0_seq32_m.51761	PREDICTED: serine carboxypeptidase-like 34 [Gossypium hirsutum]	11.371	6.7125	18	2	3	2	0.999	0.996	1.035	0.983	1.026	0.971	0.983	4.65E-01
cds.Camellia_sinensis.comp33852_c0_seq1_m.4083	PREDICTED: probable LRR receptor-like serine/threonine-protein kinase At1g06840 [Sesamum	10.719	5.4683	26.3	2	2	2	0.968	1.019	1.148	1.084	0.875	0.917	0.917	3.46E-01

cds.Camellia_sinensis.comp33856_c0_seq1_m.4923	PREDICTED: U4/U6 small nuclear ribonucleoprotein PRP4-like protein [Capsicum	65.943	2.96	3.2	2	3	2	0.968	1.05	1.07	0.994	0.93	0.987	0.943	1.92E-01
cds.Camellia_sinensis.comp33856_c0_seq2_m.19953	PREDICTED: L-idonate 5-dehydrogenase isoform X1 [Vitis vinifera]	40.424	1.3814	4.8	2	2	1	0.891	0.879	0.896	1.144	1.103	1.134	1.268	4.18E-05
cds.Camellia_sinensis.comp33862_c0_seq1_m.24624	PREDICTED: aspartate carbamoyltransferase, chloroplastic [Vitis vinifera]	20.835	2.2978	16.8	3	4	1	1.121	1.094	0.895	0.985	0.935	0.957	0.925	3.73E-01
cds.Camellia_sinensis.comp33862_c0_seq2_m.25605	PREDICTED: aspartate carbamoyltransferase, chloroplastic [Vitis vinifera]	48.528	80.381	20.4	9	12	7	0.965	0.985	1.001	1.095	1.028	1.028	1.068	5.04E-02

cds.Camellia_sinensis.comp33864_c0_seq1_m.18334	PREDICTED: phosphoacetylglucosamine mutase [Sesamum indicum]	21.564	20.752	19.5	3	3	3	0.985	1.027	0.968	1.006	0.989	1.069	1.028	4.02E-01
cds.Camellia_sinensis.comp33864_c0_seq4_m.31714	phosphoacetylglucosamine mutase family protein [Populus trichocarpa]	25.32	3.0009	6.5	1	1	1								
cds.Camellia_sinensis.comp33865_c0_seq1_m.12153	PREDICTED: alpha-L-arabinofuranosidase 1 [Theobroma cacao]	73.682	145.76	26.5	14	29	14	1.068	1.001	1.028	0.965	0.956	0.949	0.927	1.75E-02
cds.Camellia_sinensis.comp33869_c0_seq9_m.15654	PREDICTED: dihydroorotate dehydrogenase (quinone), mitochondrial [Nicotiana tomentosiformis]	48.933	21.725	16.2	5	8	5	0.973	1.001	0.991	1.041	1.021	0.987	1.028	1.91E-01

cds.Camellia_sinensis.comp33870_c0_seq1_m.320	PREDICTED: BEACH domain-containing protein C2 isoform X1 [Vitis vinifera]	239.69	1.1736	0.4	1	1	1								
cds.Camellia_sinensis.comp33870_c0_seq1_m.321	PREDICTED: BEACH domain-containing protein C2 isoform X1 [Vitis vinifera]	81.947	7.2691	4.9	3	3	3								
cds.Camellia_sinensis.comp33871_c0_seq3_m.5629	PREDICTED: signal recognition particle subunit SRP72 [Solanum lycopersicum]	73.239	3.9676	2.9	2	2	2	1.1	1.084	1.017	0.91	0.937	0.934	0.869	5.58E-03
cds.Camellia_sinensis.comp33871_c0_seq50_m.42386	PREDICTED: haloacid dehalogenase-like hydrolase domain-containing protein 3 isoform X5 [Juglans regia]	28.024	50.901	36.8	8	13	8	0.996	0.99	0.974	1.002	1.01	1.021	1.025	4.73E-02

cds.Camellia_sinensis.comp33886_c0_seq1_m.19146	PREDICTED: 26S proteasome non-ATPase regulatory subunit 12 homolog A [Juglans regia]	50.601	34.445	30.1	13	19	5	0.972	0.995	0.985	1.031	1.012	1.011	1.035	2.17E-02
cds.Camellia_sinensis.comp33886_c0_seq4_m.36703	PREDICTED: 26S proteasome non-ATPase regulatory subunit 12 homolog A-like [Malus domestica]	37.632	1.3554	27.4	9	15	0								
cds.Camellia_sinensis.comp33887_c0_seq1_m.271	PREDICTED: auxin transport protein BIG [Vitis vinifera]	333.18	32.131	4.4	13	13	13	1.116	1.114	1.142	0.861	0.89	0.953	0.802	2.02E-03
cds.Camellia_sinensis.comp33887_c0_seq4_m.373	PREDICTED: auxin transport protein BIG [Fragaria vesca subsp. vesca]	19.515	3.717	5.7	1	1	1								

cds.Camellia_sinensis.comp33888_c0_seq1_m.2131	PREDICTED: myosin-1 [Vitis vinifera]	132.59	3.8075	1.6	2	2	2	0.999	1.095	1.014	1.057	0.888	0.952	0.932	2.82E-01
cds.Camellia_sinensis.comp33889_c0_seq28_m.25885	PREDICTED: structural maintenance of chromosomes domain-containing protein isoform X1 [Citrus sinensis]	34.54	4.4585	13.8	5	6	2	0.958	1.016	1.01	0.986	1.063	0.982	1.016	6.56E-01
cds.Camellia_sinensis.comp33889_c0_seq30_m.29004	PREDICTED: structural maintenance of chromosomes domain-containing protein isoform X1 [Citrus sinensis]	34.724	27.86	28.8	8	11	5	0.984	1.008	0.931	1.011	1.046	1.097	1.079	8.49E-02
cds.Camellia_sinensis.comp33896_c0_seq1_m.3923	PREDICTED: phospholipase D beta 2-like [Juglans regia]	123.44	5.5244	1.1	1	1	1								

cds.Camellia_sinensis.comp33898_c0_seq3_m.18677	PREDICTED: chaperone protein ClpD, chloroplastic-like isoform X1 [Nicotiana tabacum]	64.975	1.4974	2	1	1	1								
cds.Camellia_sinensis.comp33900_c0_seq8_m.36305	PREDICTED: structural maintenance of chromosomes protein 2-1-like [Jatropha curcas]	35.458	1.7112	2.8	1	1	1								
cds.Camellia_sinensis.comp33901_c0_seq11_m.12359	PREDICTED: ruBisCO large subunit-binding protein subunit beta, chloroplastic [Prunus mume]	64.509	323.31	68.3	39	143	39	1.026	0.983	1	0.982	1.014	0.994	0.994	7.08E-01

cds.Camellia_sinensis.comp33906_c0_seq2_m.25830	PREDICTED: cinnamoyl-CoA reductase 1-like [Juglans regia]	35.449	48.443	29	8	16	8	0.964	0.981	0.994	1.039	1.006	1.017	1.042	3.43E-02
cds.Camellia_sinensis.comp33906_c0_seq3_m.27977	PREDICTED: cinnamoyl-CoA reductase 1-like [Populus euphratica]	37.368	25.105	23.7	8	18	8	1.004	1.022	1.038	0.98	1.025	0.987	0.977	2.31E-01
cds.Camellia_sinensis.comp33908_c0_seq1_m.29164	PREDICTED: probable 2-oxoglutarate-dependent dioxygenase At3g49630 isoform X2 [Gossypium hirsutum]	38.545	60.667	32.3	9	20	9	0.972	0.947	0.971	1.047	1.045	1.049	1.087	1.05E-02
cds.Camellia_sinensis.comp33909_c1_seq24_m.41178	PREDICTED: prefoldin subunit 6 [Eucalyptus grandis]	14.698	86.539	46.9	8	14	8	0.984	0.961	0.941	1.024	1.058	1.033	1.079	9.16E-03

cds.Camellia_sinensis.comp33910_c0_seq20_m.15403	PREDICTED: polypyrimidine tract-binding protein homolog 3 isoform X1 [Vitis vinifera]	49.338	13.329	4.7	2	5	2	0.959	1.005	0.952	1.074	1.059	1.015	1.08	3.33E-02
cds.Camellia_sinensis.comp33911_c0_seq11_m.15177	PREDICTED: eukaryotic peptide chain release factor subunit 1-3 [Vitis vinifera]	49.119	23.01	12.6	5	7	5	1.045	1.09	1.115	0.96	0.874	0.9	0.841	6.72E-03
cds.Camellia_sinensis.comp33912_c0_seq1_m.4257	PREDICTED: ribonuclease TUDOR 1 [Vitis vinifera]	108.05	173.05	39.1	34	55	32	1.073	1.059	1.055	0.941	0.944	0.929	0.883	6.09E-05
cds.Camellia_sinensis.comp33912_c0_seq3_m.52756	PREDICTED: ribonuclease TUDOR 1 [Vitis vinifera]	17.419	40.241	26.9	3	7	1	1.058	0.937	1.126	0.873	0.989	1.007	0.919	3.01E-01

cds.Camellia_sinensis.comp33913_c0_seq3_m.10650	PREDICTED: chitin elicitor receptor kinase 1 isoform X2 [Ricinus communis]	30.026	1.2562	7.8	2	2	2	0.965	0.983	1.021	0.94	1.014	1.087	1.024	6.45E-01
cds.Camellia_sinensis.comp33915_c0_seq13_m.14647	PREDICTED: SWI/SNF complex component SNF12 homolog [Prunus mume]	60.533	3.1169	4.6	2	2	2	0.899	0.922	1.031	0.997	1.102	1.08	1.115	1.03E-01
cds.Camellia_sinensis.comp33917_c0_seq5_m.17790	CBL-interacting protein kinase 12 [Vitis vinifera]	23.858	1.2942	5.2	1	1	1								
cds.Camellia_sinensis.comp33918_c4_seq1_m.22509	PREDICTED: 26S proteasome non-ATPase regulatory subunit 11 homolog [Juglans regia]	49.861	85.341	31.5	15	25	15	0.96	0.972	0.98	1.053	1.033	1.019	1.066	4.68E-03
cds.Camellia_sinensis.comp33922_c0_seq2_m.15111	transmembrane protein [Arabidopsis thaliana]	54.251	11.879	12.6	7	9	7	1.074	1.065	1.071	0.884	0.976	0.936	0.871	4.04E-02

cds.Camellia_sinensis.comp33925_c0_seq16_m.56713	PREDICTED: 2-oxoglutarate-Fe(II) type oxidoreductase [Nicotiana tomentosiformis]	12.585	1.5248	8.1	1	1	1	0.864	1.012	0.958	1.086	1.085	1.033	1.131	6.46E-02
cds.Camellia_sinensis.comp33925_c0_seq2_m.3411	PREDICTED: eukaryotic translation initiation factor 3 subunit A [Vitis vinifera]	55.415	41.034	19.5	10	16	10	1.054	1.054	1.027	0.935	0.944	0.961	0.906	1.12E-03
cds.Camellia_sinensis.comp33925_c0_seq2_m.3412	PREDICTED: eukaryotic translation initiation factor 3 subunit A [Vitis vinifera]	47.53	16.462	19	8	13	8	1.03	1.053	1.055	0.975	0.917	0.944	0.904	6.28E-03
cds.Camellia_sinensis.comp33930_c0_seq10_m.8955	PREDICTED: methylcrotonoyl-CoA carboxylase subunit alpha, mitochondrial isoform X2 [Vitis	70.867	56.37	20.7	10	17	10	0.978	1.04	0.97	1.013	0.983	0.958	0.989	7.02E-01

cds.Camellia_sinensis.comp33932_c0_seq1_m.28186	PREDICTED: cytosolic sulfotransferase 15-like [Pyrus x bretschneideri]	43.31	43.773	17.6	6	16	5	0.958	0.999	0.998	1.017	0.978	1.037	1.026	3.10E-01
cds.Camellia_sinensis.comp33933_c0_seq1_m.2733	PREDICTED: protein transport protein SEC31 homolog B isoform X2 [Vitis vinifera]	100.81	93.229	23.7	17	23	13	0.959	1.004	1.011	1.031	1.008	1.006	1.024	2.64E-01
cds.Camellia_sinensis.comp33933_c0_seq3_m.14914	PREDICTED: protein transport protein SEC31 homolog B isoform X2 [Vitis vinifera]	66.449	31.616	17.9	11	13	7	0.957	0.96	0.978	1.025	1.043	1.048	1.076	1.54E-03

cds.Camellia_sinensis.comp33935_c0_seq3_m.47980	PREDICTED: probable sodium/metabolite cotransporter BASS2, chloroplastic isoform X1 [Nelumbo nucifera]	20.286	6.6018	6.9	1	1	1	1.089	1.057	1.128	0.848	0.923	0.932	0.826	5.58E-03
cds.Camellia_sinensis.comp33941_c0_seq17_m.22888	PREDICTED: putative transferase At4g12130, mitochondrial [Juglans regia]	45.722	2.5172	2.7	1	1	1								
cds.Camellia_sinensis.comp33941_c0_seq30_m.30670	PREDICTED: carbon catabolite repressor protein 4 homolog 5 isoform X2 [Vitis vinifera]	19.971	-2	4.1	1	1	1	0.932	0.975	0.979	1.078	1.029	1.033	1.088	1.77E-02

cds.Camellia_sinensis.comp33943_c0_seq1_m.12954	PREDICTED: probable inactive purple acid phosphatase 1 isoform X1 [Citrus sinensis]	68.668	203.44	36.1	16	26	10	0.942	0.918	0.935	1.105	1.1	1.049	1.164	1.16E-03
cds.Camellia_sinensis.comp33943_c0_seq4_m.15739	PREDICTED: probable inactive purple acid phosphatase 1 [Malus domestica]	40.079	7.7103	23.3	7	9	3	1.027	1.011	0.935	1.088	0.915	1.034	1.022	7.57E-01
cds.Camellia_sinensis.comp33944_c1_seq11_m.40983	PREDICTED: acyl-CoA-binding domain-containing protein 4 [Juglans regia]	21.779	13.464	20.1	4	6	3	1.042	1.054	1.094	0.932	0.944	0.965	0.891	2.92E-03
cds.Camellia_sinensis.comp33944_c1_seq4_m.13093	PREDICTED: probable calcium-binding protein CML43 [Theobroma cacao]	28.13	7.8116	22.5	7	8	7	1.036	1.038	1.036	0.999	0.971	0.965	0.944	3.22E-02

cds.Camellia_sinensis.comp33944_c2_seq1_m.32174	PREDICTED: clathrin light chain 1-like [Nicotiana attenuata]	36.048	8.4553	5.6	2	2	2								
cds.Camellia_sinensis.comp33947_c1_seq37_m.2586	PREDICTED: UPF0603 protein At1g54780, chloroplastic [Cucumis melo]	32.325	97.449	23.4	6	27	6	1.01	0.985	1.006	0.967	1.027	1.031	1.008	7.49E-01
cds.Camellia_sinensis.comp33947_c1_seq9_m.13318	PREDICTED: acetolactate synthase 1, chloroplastic [Sesamum indicum]	73.223	41.869	17.1	9	13	9	0.944	0.999	1.014	1.027	1.01	1.009	1.03	2.53E-01
cds.Camellia_sinensis.comp33955_c0_seq1_m.3353	PREDICTED: villin-4 [Vitis vinifera]	83.45	126.54	24.1	15	21	15	1.028	1.069	1.077	0.95	0.941	0.924	0.887	1.94E-03
cds.Camellia_sinensis.comp33955_c0_seq1_m.3354	PREDICTED: villin-4-like [Juglans regia]	20.984	12.487	28.3	4	5	4	0.987	0.976	1.113	1	1.016	0.918	0.954	4.26E-01

cds.Camellia_sinensis.comp33956_c0_seq1_m.27445	PREDICTED: zeaxanthin epoxidase, chloroplastic isoform X1 [Nelumbo nucifera]	24.213	49.34	23.1	5	14	5	0.984	1.026	1.001	0.987	0.997	0.997	0.99	4.77E-01
cds.Camellia_sinensis.comp33959_c1_seq2_m.24595	PREDICTED: eukaryotic translation initiation factor 5B-like isoform X2 [Capsicum annuum]	29.222	1.3872	3.7	1	1	1								
cds.Camellia_sinensis.comp33965_c0_seq1_m.33803	PREDICTED: probable glutathione S-transferase parC [Arachis ipaensis]	25.816	46.468	34.5	10	27	8	1.04	0.973	1.015	1.006	0.984	0.985	0.982	4.50E-01
cds.Camellia_sinensis.comp33966_c0_seq2_m.7726	PREDICTED: VHS domain-containing protein At3g16270 [Vitis vinifera]	77.822	3.2652	2.8	2	2	2	1.022	1.052	1.042	0.912	0.95	1.016	0.924	6.93E-02

cds.Camellia_sinensis.comp33972_c1_seq3_m.2695	PREDICTED: probable isoleucine--tRNA ligase, cytoplasmic [Sesamum indicum]	132.26	55.389	15.1	18	20	18	1.008	1.012	0.992	1.015	0.991	1.012	1.002	8.49E-01
cds.Camellia_sinensis.comp33978_c1_seq3_m.5793	PREDICTED: dynamin-related protein 3A-like [Nicotiana tabacum]	72.769	31.305	17.9	12	16	10	0.994	1.011	0.987	1.022	1.006	1.01	1.015	1.49E-01
cds.Camellia_sinensis.comp33978_c1_seq3_m.5794	PREDICTED: dynamin-related protein 3A-like, partial [Nicotiana tabacum]	18.096	1.9426	14.8	2	2	1	0.985	1.037	1.091	1.012	1.011	0.874	0.931	2.67E-01
cds.Camellia_sinensis.comp33978_c1_seq5_m.27523	PREDICTED: dynamin-related protein 3A [Sesamum indicum]	36.661	8.9523	14.4	4	7	2	0.942	1.045	0.969	0.969	1.055	1.035	1.035	4.39E-01

cds.Camellia_sinensis.comp33980_c1_seq1_m.8778	PREDICTED: vacuolar protein sorting-associated protein 52 A [Jatropha curcas]	80.486	3.0987	1.7	1	1	1								
cds.Camellia_sinensis.comp33981_c0_seq2_m.6161	PREDICTED: dentin sialophosphoprotein [Pyrus x bretschneideri]	105.54	9.7128	3.8	3	3	3	1.008	1.038	1.069	0.985	0.947	1.005	0.943	7.29E-02
cds.Camellia_sinensis.comp33982_c1_seq3_m.7198	cyclopropane-fatty-acyl-phospholipid synthase family protein [Populus trichocarpa]	66.255	1.6065	1.5	1	1	1								
cds.Camellia_sinensis.comp33983_c0_seq2_m.40866	Glucan endo-1,3-beta-glucosidase, basic isoform [Morus notabilis]	30.21	58.25	33.8	6	10	6	0.981	1.01	1.1	0.988	0.973	0.957	0.944	1.87E-01

cds.Camellia_sinensis.comp33986_c1_seq3_m.35934	PREDICTED: chlorophyll a-b binding protein of LHCII type 1 [Vitis	30.915	102.14	41.2	7	61	3	0.928	0.906	1.014	1.074	1.042	1.019	1.101	6.09E-02
cds.Camellia_sinensis.comp33988_c1_seq2_m.15117	PREDICTED: signal recognition particle subunit SRP68 [Ipomoea nil]	59.751	3.9188	3.4	2	2	2	0.916	1.171	1.112	1.004	0.873	0.929	0.877	2.09E-01
cds.Camellia_sinensis.comp33988_c1_seq6_m.31549	PREDICTED: nudix hydrolase 14, chloroplastic isoform X2 [Citrus sinensis]	17.716	10.655	22.9	3	5	3	0.982	0.899	0.934	1.12	1.083	1.046	1.154	1.13E-02
cds.Camellia_sinensis.comp33988_c1_seq6_m.31550	PREDICTED: nudix hydrolase 14, chloroplastic isoform X2 [Daucus carota subsp. sativus]	11.266	1.9009	8.7	1	2	1								

cds.Camellia_sinensis.comp33989_c0_seq4_m.26489	PREDICTED: src substrate protein p85 [Sesamum indicum]	38.921	19.452	22.4	7	8	7	0.979	0.981	0.977	0.987	0.956	1.065	1.024	5.50E-01
cds.Camellia_sinensis.comp33994_c0_seq2_m.42269	PREDICTED: protein FLUORESCENT IN BLUE LIGHT, chloroplastic isoform X2 [Juglans regia]	23.548	16.725	28.9	6	10	6	0.996	1.053	1.153	0.918	0.939	0.945	0.875	3.90E-02
cds.Camellia_sinensis.comp33995_c0_seq2_m.2476	PREDICTED: trafficking protein particle complex subunit 8 [Vitis vinifera]	125.51	3.7889	2	3	4	3	1.007	1.032	0.998	1.008	1.024	0.941	0.979	4.76E-01
cds.Camellia_sinensis.comp33998_c0_seq1_m.9870	PREDICTED: vesicle-fusing ATPase-like [Nicotiana tabacum]	81.052	70.707	32.8	22	24	22	0.954	0.999	0.994	1.009	0.995	1.03	1.03	1.75E-01

cds.Camellia_sinensis.comp34003_c0_seq9_m.5917	PREDICTED: probable transmembrane GTPase FZO-like, chloroplastic isoform X2 [Vitis vinifera]	55.531	31.512	15.1	6	11	6	0.973	1.058	0.999	0.988	0.979	0.95	0.963	2.43E-01
cds.Camellia_sinensis.comp34003_c0_seq9_m.5918	PREDICTED: probable transmembrane GTPase FZO-like, chloroplastic isoform X1 [Vitis vinifera]	36.545	13.777	19.8	6	6	6	1.017	1.022	1.039	0.957	0.945	0.986	0.938	1.08E-02
cds.Camellia_sinensis.comp34005_c2_seq9_m.33257	PREDICTED: UPF0047 protein C4A8.02c [Sesamum indicum]	21.143	4.8625	9.3	2	3	2	0.995	1.001	1.033	1.012	0.99	0.98	0.984	3.57E-01
cds.Camellia_sinensis.comp34008_c0_seq3_m.6454	PREDICTED: protein SMG7 [Ipomoea nil]	103.43	-2	1.5	1	1	1	1.025	1.051	0.994	1.009	0.963	0.96	0.955	1.14E-01

cds.Camellia_sinensis.comp34012_c0_seq1_m.9574	PREDICTED: histone-lysine N-methyltransferase, H3 lysine-9 specific SUVH1 [Vitis vinifera]	62.024	18.599	12	6	6	6	1.133	1.139	1.052	0.858	0.918	0.868	0.795	2.30E-03
cds.Camellia_sinensis.comp34012_c0_seq9_m.30706	PREDICTED: carboxymethylenebutenolidase homolog [Citrus sinensis]	34.366	141.17	30.8	7	20	7	0.948	0.982	0.934	1.014	1.028	1.045	1.078	1.20E-02
cds.Camellia_sinensis.comp34014_c0_seq3_m.37894	PREDICTED: triacylglycerol lipase SDP1 [Vitis vinifera]	37.195	-2	3	1	2	1	1.032	0.985	1.076	0.977	0.961	0.97	0.94	7.84E-02
cds.Camellia_sinensis.comp34017_c0_seq1_m.26436	PREDICTED: DEAD-box ATP-dependent RNA helicase 50 isoform X2 [Nelumbo nucifera]	51.415	3.1721	2.8	1	1	1								

cds.Camellia_sinensis.comp34018_c0_seq2_m.20619	PREDICTED: protein KINESIN LIGHT CHAIN-RELATED 2 [Vitis vinifera]	58.212	4.9927	7.3	3	3	3	0.932	0.909	0.868	1.065	1.116	1.145	1.228	2.32E-03
cds.Camellia_sinensis.comp34025_c0_seq20_m.22499	PREDICTED: protein disulfide isomerase-like 5-4 [Sesamum indicum]	53.83	3.7179	2.1	1	2	1								
cds.Camellia_sinensis.comp34025_c0_seq9_m.18171	PREDICTED: YTH domain-containing family protein 2-like isoform X5 [Gossypium raimondii]	24.909	2.5779	4.1	1	1	1								
cds.Camellia_sinensis.comp34027_c0_seq1_m.4704	PREDICTED: valine--tRNA ligase, mitochondrial isoform X1 [Sesamum	62.244	22.744	14.3	8	12	7	1.016	1.021	1.018	0.975	0.985	0.978	0.962	3.03E-04

cds.Camellia_sinensis.comp34027_c0_seq1_m.4705	PREDICTED: valine--tRNA ligase, chloroplastic/mitochondrial 2 isoform X2 [Vitis vinifera]	47.632	11.537	16.3	7	7	7	1.01	0.993	1.035	1.045	0.977	0.969	0.985	5.82E-01
cds.Camellia_sinensis.comp34029_c0_seq1_m.14296	PREDICTED: protein GDAP2 homolog isoform X1 [Sesamum indicum]	62.986	3.4381	2.1	1	1	1								
cds.Camellia_sinensis.comp34030_c0_seq1_m.10720	PREDICTED: dynamin-related protein 3A isoform X2 [Ricinus communis]	90.887	25.712	2.8	2	3	1	0.931	0.984	1.065	1.098	0.989	0.958	1.022	7.25E-01
cds.Camellia_sinensis.comp34031_c0_seq2_m.14854	PREDICTED: carboxyl-terminal-processing peptidase 2, chloroplastic isoform X1 [Vitis vinifera]	27.679	2.6932	10.5	3	3	2	0.961	1.026	1.063	1.021	0.961	0.979	0.971	4.48E-01

cds.Camellia_sinensis.comp34037_c0_seq1_m.20889	PREDICTED: sorting nexin 1 [Nicotiana sylvestris]	45.871	13.756	18.3	8	9	8	0.965	0.968	0.982	1.002	0.998	1.042	1.044	4.52E-02
cds.Camellia_sinensis.comp34040_c0_seq1_m.20513	PREDICTED: 3-oxoacyl-[acyl-carrier-protein] synthase I, chloroplastic-like [Solanum pennellii]	54.079	49.836	13.1	8	15	8	0.973	1.064	1.05	0.996	0.972	0.954	0.947	1.50E-01
cds.Camellia_sinensis.comp34044_c2_seq11_m.28589	PREDICTED: syntaxin-22-like [Ipomoea nil]	29.996	4.0373	8.4	3	3	2	0.983	0.988	0.998	0.996	1.027	1.021	1.025	7.49E-02
cds.Camellia_sinensis.comp34044_c2_seq21_m.42698	PREDICTED: probable receptor-like protein kinase At5g47070 [Ricinus communis]	30.79	1.4183	6.5	2	2	1	0.908	0.884	0.89	1.226	1.154	0.992	1.257	7.12E-02
cds.Camellia_sinensis.comp34044_c2_seq7_m.27172	PREDICTED: syntaxin-22 [Vitis vinifera]	30.388	2.2144	7.3	2	2	1								

cds.Camellia_sinensis.comp34048_c0_seq1_m.4458	PREDICTED: ATPase 11, plasma membrane-type [Theobroma cacao]	105.09	85.251	33.6	29	61	10	0.973	0.972	0.945	1.027	1.044	1.059	1.083	3.62E-03
cds.Camellia_sinensis.comp34051_c2_seq2_m.45356	ureidoglycolate hydrolase, putative [Medicago truncatula]	21.358	10.422	26.6	5	5	5	0.867	0.908	0.955	1.231	1.029	1.064	1.218	3.44E-02
cds.Camellia_sinensis.comp34052_c0_seq1_m.14055	PREDICTED: ERAD-associated E3 ubiquitin-protein ligase HRD1B [Theobroma cacao]	62.437	13.215	3.1	1	1	1	0.914	0.927	1.12	1.075	0.938	1.051	1.035	6.61E-01
cds.Camellia_sinensis.comp34053_c0_seq1_m.3953	PREDICTED: alpha-mannosidase isoform X2 [Populus euphratica]	22.514	15.463	24.8	4	4	4	0.994	1.08	1.082	1.014	0.88	0.981	0.911	1.39E-01

cds.Camellia_sinensis.comp34054_c0_seq1_m.7832	PREDICTED: cell division cycle protein 48 homolog [Gossypium hirsutum]	89.762	8.7774	40.7	28	67	1	0.996	1.166	0.961	0.988	0.98	0.91	0.922	2.92E-01
cds.Camellia_sinensis.comp34055_c0_seq1_m.3127	plant/MOJ9-14 protein [Medicago truncatula]	88.701	2.7142	1.2	1	1	1	1.091	1.096	0.959	0.913	0.906	1.016	0.901	1.45E-01
cds.Camellia_sinensis.comp34056_c1_seq2_m.9658	PREDICTED: cullin-3A-like [Nicotiana tabacum]	85.255	4.0169	3.3	2	2	2	0.993	0.995	0.999	1.088	0.965	0.977	1.014	7.69E-01
cds.Camellia_sinensis.comp34065_c3_seq1_m.1918	Elongation factor Ts [Morus notabilis]	135.44	261.06	34.4	40	91	40	1.095	1.081	1.08	0.902	0.9	0.907	0.832	1.65E-06
cds.Camellia_sinensis.comp34068_c2_seq1_m.15283	PREDICTED: eukaryotic translation initiation factor 2A isoform X1 [Nelumbo nucifera]	56.865	21.105	12.6	6	7	6	0.987	1.036	1.009	0.982	1.011	0.986	0.983	3.53E-01

cds.Camellia_sinensis.comp34072_c0_seq2_m.25478	PREDICTED: probable L-type lectin-domain containing receptor kinase II.1 [Juglans regia]	57.269	5.7128	2.1	1	1	1								
cds.Camellia_sinensis.comp340748_c0_seq1_m.58494	PREDICTED: stachyose synthase [Jatropha curcas]	11.651	33.483	19.6	1	2	1	1.086	1.04	0.928	1.038	0.985	0.923	0.965	5.78E-01
cds.Camellia_sinensis.comp34075_c0_seq3_m.11466	PREDICTED: AP-2 complex subunit alpha-1-like isoform X2 [Nicotiana tomentosiformis]	69.07	16.721	10.1	6	6	6	0.929	1.004	1.003	0.981	1.086	1.019	1.051	2.75E-01
cds.Camellia_sinensis.comp34075_c0_seq3_m.11467	PREDICTED: AP-2 complex subunit alpha-1 [Vitis vinifera]	12.898	1.4788	9.2	1	1	1								

cds.Camellia_sinensis.comp34075_c0_seq7_m.34932	PREDICTED: AP-2 complex subunit alpha-1 [Vitis vinifera]	31.815	4.9637	7.9	2	2	2	1.045	0.952	0.983	1.033	1.033	0.965	1.017	6.54E-01
cds.Camellia_sinensis.comp34083_c0_seq2_m.8412	PREDICTED: tyrosine--tRNA ligase, cytoplasmic-like [Jatropha curcas]	43.794	8.7453	8.6	2	2	2	0.949	0.92	1.089	0.977	1.11	0.976	1.035	6.23E-01
cds.Camellia_sinensis.comp34083_c0_seq2_m.8413		35.482	1.7408	3.1	1	1	1								
cds.Camellia_sinensis.comp34084_c0_seq10_m.46247	PREDICTED: histidinol dehydrogenase, chloroplastic isoform X7 [Vitis vinifera]	24.454	25.154	17.7	3	4	3	0.938	0.978	0.957	1.051	1.083	1.02	1.098	1.15E-02
cds.Camellia_sinensis.comp34085_c1_seq1_m.20728	glycine-rich family protein [Populus trichocarpa]	49.596	35.995	14.8	6	8	6	1.114	1.101	1.093	0.889	0.905	0.881	0.809	2.26E-05

cds.Camellia_sinensis.comp34086_c0_seq36_m.34160	PREDICTED: chaperonin CPN60-like 2, mitochondrial isoform X2 [Vitis	30.119	2.5101	3.5	1	1	1								
cds.Camellia_sinensis.comp34086_c1_seq1_m.19789	PREDICTED: probable ADP-ribosylation factor GTPase-activating protein AGD6 [Nelumbo nucifera]	50.492	2.1232	4	2	2	2	1.107	1.06	1.121	0.888	0.832	0.965	0.817	1.15E-02
cds.Camellia_sinensis.comp34089_c0_seq1_m.14562	PREDICTED: nicalin-1 [Vitis vinifera]	62.37	22.888	10.5	5	5	5	0.992	1.004	1.027	1.028	0.981	0.991	0.992	6.81E-01
cds.Camellia_sinensis.comp34092_c0_seq1_m.28868	PREDICTED: 3-hydroxyisobutyryl-CoA hydrolase-like protein 1, mitochondrial [Prunus	46.256	3.8994	3.6	1	1	1								

cds.Camellia_sinensis.comp34094_c1_seq22_m.42788	PREDICTED: proline-, glutamic acid- and leucine-rich protein 1 [Theobroma cacao]	18.942	2.4182	5.7	1	1	1								
cds.Camellia_sinensis.comp34095_c2_seq5_m.9186	PREDICTED: probable serine/threonine-protein kinase DDB_G0276461 [Solanum tuberosum	50.746	6.7516	6	2	2	2	1.031	1.142	1.047	0.963	0.901	0.906	0.86	1.73E-02
cds.Camellia_sinensis.comp34096_c0_seq1_m.9195	PREDICTED: CAAX prenyl protease 1 homolog [Ipomoea nil]	48.48	12.5	12.7	5	8	5	0.927	0.936	0.982	0.996	1.138	1.049	1.119	5.99E-02
cds.Camellia_sinensis.comp34099_c1_seq1_m.12104	PREDICTED: cytosolic endo-beta-N-acetylglucosaminidase 1 isoform X3 [Vitis vinifera]	81.311	64.775	15.7	9	14	9	0.937	0.986	0.986	1.041	1.027	1.045	1.07	1.87E-02

cds.Camellia_sinensis.comp34100_c0_seq1_m.24455	putative pyruvate, phosphate dikinase regulatory protein [Morus notabilis]	44.46	15.26	16.8	6	6	6	0.871	0.959	0.882	1.062	1.027	1.199	1.212	2.67E-02
cds.Camellia_sinensis.comp34105_c0_seq4_m.5315	PREDICTED: large proline-rich protein BAG6-like isoform X1 [Juglans regia]	97.531	28.617	14.2	10	13	10	0.986	1.021	1.022	1.013	0.982	0.995	0.987	4.34E-01
cds.Camellia_sinensis.comp34105_c0_seq5_m.6640	PREDICTED: large proline-rich protein bag6-A isoform X3 [Vitis vinifera]	94.314	7.3546	2.4	2	3	2	1.143	0.924	1.138	0.902	0.826	1.043	0.865	2.11E-01
cds.Camellia_sinensis.comp34108_c0_seq3_m.10334	PREDICTED: pyridine nucleotide-disulfide oxidoreductase domain-containing protein 2 [Vitis vinifera]	65.568	1.3772	2.3	1	1	1								

cds.Camellia_sinensis.comp34111_c0_seq1_m.15589	PREDICTED: carotene epsilon-monooxygenase, chloroplastic isoform X1 [Vitis vinifera]	62.123	71.213	16.2	8	12	8	1.056	1.026	1.007	0.951	0.976	0.979	0.941	2.14E-02
cds.Camellia_sinensis.comp34112_c0_seq12_m.1183	PREDICTED: UDP-glucose:glycoprotein glucosyltransferase isoform X2 [Vitis vinifera]	126.55	47.391	9.3	9	10	9	1.088	1.063	1.082	0.951	0.916	0.902	0.856	8.39E-04
cds.Camellia_sinensis.comp34112_c0_seq12_m.1184	PREDICTED: UDP-glucose:glycoprotein glucosyltransferase isoform X1 [Solanum tuberosum	61.979	20.19	10.9	6	6	6	1.086	1.095	1.135	0.946	0.858	0.9	0.815	2.82E-03
cds.Camellia_sinensis.comp34114_c0_seq13_m.35078	PREDICTED: polyol transporter 5 [Theobroma cacao]	37.876	4.2453	9	3	4	3	0.973	1.039	1.075	0.927	1.012	0.978	0.945	2.20E-01

cds.Camellia_sinensis.comp34116_c0_seq2_m.4556	PREDICTED: coatomer subunit beta'-2 isoform X1 [Ricinus communis]	104.45	20.525	8.1	6	6	6	1.066	1.107	1.038	0.965	0.874	0.99	0.881	3.93E-02
cds.Camellia_sinensis.comp34117_c0_seq1_m.2876	PREDICTED: extensin isoform X1 [Vitis vinifera]	21.63	2.8677	7.1	1	1	1	1.11	1.095	1.006	0.89	0.901	0.975	0.861	2.38E-02
cds.Camellia_sinensis.comp34120_c0_seq1_m.4606	PREDICTED: glucose-1-phosphate adenylyltransferase small subunit, chloroplastic/amyloplastic [Nicotiana sylvestris]	56.727	230.67	53.6	21	67	21	1.083	1.044	1.046	0.926	0.938	0.944	0.885	7.59E-04
cds.Camellia_sinensis.comp34120_c1_seq1_m.21354	PREDICTED: serine carboxypeptidase-like 17 [Juglans regia]	59.467	70.042	18.7	9	17	7	1.066	0.997	1.088	0.957	0.923	0.972	0.905	3.13E-02

cds.Camellia_sinensis.comp34121_c0_seq1_m.11319	cystathionine gamma synthase [Solanum lycopersicum]	61.571	42.812	13.6	7	9	4	0.962	0.854	0.97	1.105	1.071	1.095	1.174	1.84E-02
cds.Camellia_sinensis.comp34121_c0_seq2_m.17596	cystathionine gamma synthase [Solanum lycopersicum]	61.6	33.408	12	7	8	4	0.948	0.963	0.908	1.045	1.089	1.059	1.133	4.30E-03
cds.Camellia_sinensis.comp34123_c0_seq1_m.23759	PREDICTED: bifunctional nitrilase/nitrile hydratase NIT4A [Citrus sinensis]	41.769	40.315	18.1	6	7	6	1.017	1.02	0.951	1.033	0.953	0.987	0.995	8.84E-01
cds.Camellia_sinensis.comp34123_c1_seq1_m.6016	PREDICTED: heat shock protein 83 [Populus euphratica]	81.075	57.041	22	17	33	9	1.011	1.064	1.039	0.998	0.926	0.956	0.925	4.05E-02
cds.Camellia_sinensis.comp34123_c1_seq4_m.18635	PREDICTED: eukaryotic initiation factor 4A-14 [Sesamum indicum]	22.008	49.11	33.5	7	25	2	0.94	0.873	0.9	1.063	1.098	1.119	1.209	1.92E-03

cds.Camellia_sinensis.comp34123_c1_seq6_m.19320	PREDICTED: eukaryotic initiation factor 4A-6-like [Nicotiana tabacum]	25.518	8.2718	41	10	26	3	0.928	0.985	0.958	1.082	0.959	1.079	1.087	1.32E-01
cds.Camellia_sinensis.comp34128_c0_seq3_m.18780	PREDICTED: prolycopene isomerase, chloroplastic isoform X1 [Juglans regia]	33.855	16.813	13.2	3	5	3	1.011	1.031	1.112	1.009	0.92	0.918	0.903	7.37E-02
cds.Camellia_sinensis.comp34128_c0_seq3_m.18781	PREDICTED: prolycopene isomerase, chloroplastic isoform X1 [Jatropha curcas]	32.556	8.6008	9.1	3	3	3	0.979	1.059	1.157	0.918	1.041	0.848	0.879	1.64E-01
cds.Camellia_sinensis.comp34132_c1_seq1_m.26547	PREDICTED: ruvB-like protein 1 [Glycine max]	50.618	55.934	29.3	11	15	11	0.978	1.023	0.971	1.01	0.995	1.028	1.021	3.38E-01

cds.Camellia_sinensis.comp34132_c2_seq2_m.479	PREDICTED: acetyl-CoA carboxylase 1 [Theobroma cacao]	252.94	89.799	14.8	30	35	30	1.296	1.316	1.3	0.677	0.649	0.673	0.511	5.66E-07
cds.Camellia_sinensis.comp34135_c0_seq1_m.8698	PREDICTED: isoamylase 1, chloroplastic [Vitis vinifera]	73.898	8.0902	3.8	2	2	2	1.041	1.056	1.09	0.912	0.987	0.904	0.88	1.42E-02
cds.Camellia_sinensis.comp34135_c0_seq2_m.9029	PREDICTED: inter alpha-trypsin inhibitor, heavy chain 4 [Vitis vinifera]	83.599	3.5325	2.2	1	1	1	0.918	0.791	0.81	1.242	1.18	1.121	1.407	3.36E-03
cds.Camellia_sinensis.comp34137_c0_seq1_m.31036	PREDICTED: enoyl-CoA hydratase 2, peroxisomal-like [Nicotiana tabacum]	34.204	13.839	19.1	5	8	5	0.974	0.961	0.981	1.021	1.028	1.029	1.056	1.16E-03

cds.Camellia_sinensis.comp34138_c0_seq1_m.417	PREDICTED: eIF-2-alpha kinase activator GCN1 isoform X2 [Vitis vinifera]	178.54	10.868	4.1	6	6	6	1.023	1.151	1.013	1.04	0.906	0.866	0.882	1.39E-01
cds.Camellia_sinensis.comp34138_c0_seq1_m.418	PREDICTED: eIF-2-alpha kinase activator GCN1 isoform X1 [Vitis vinifera]	105.96	9.1554	5.7	5	5	5	1.028	1.1	1.058	0.992	0.91	0.907	0.882	2.31E-02
cds.Camellia_sinensis.comp34146_c1_seq2_m.12594	PREDICTED: cysteine-rich receptor-like protein kinase 10 isoform X1 [Vitis vinifera]	74.868	33.5	15.3	9	11	9	0.968	0.958	0.984	1.102	0.971	1.067	1.079	1.26E-01
cds.Camellia_sinensis.comp34154_c0_seq1_m.1213	PREDICTED: nuclear pore complex protein NUP155 isoform X1 [Vitis vinifera]	162.98	97.943	13.8	17	18	17	0.938	1.009	1	1.028	1.002	1.006	1.03	2.82E-01

cds.Camellia_sinensis.comp34158_c0_seq1_m.6372	colon cancer-associated Mic1-like protein [Medicago truncatula]	83.17	63.592	17.7	12	17	12	0.93	0.947	0.939	1.051	1.026	1.095	1.126	3.82E-03
cds.Camellia_sinensis.comp34159_c0_seq1_m.20017	PREDICTED: probable protein phosphatase 2C BIPP2C1 isoform X1 [Vitis vinifera]	51.124	15.429	7.7	3	4	3	0.95	0.983	0.99	0.978	1.11	1.009	1.06	2.32E-01
cds.Camellia_sinensis.comp34162_c0_seq2_m.20178	PREDICTED: glycerol-3-phosphate acyltransferase, chloroplastic [Vitis vinifera]	50.505	2.8519	5	3	3	3	0.973	0.98	0.998	1.035	0.989	1.04	1.038	1.02E-01
cds.Camellia_sinensis.comp34164_c0_seq1_m.29465	PREDICTED: probable aldo-keto reductase 2 [Arachis ipaensis]	37.729	161.63	47.8	18	48	17	0.955	0.948	0.968	1.05	1.076	1.044	1.104	8.76E-04

cds.Camellia_sinensis.comp34165_c0_seq4_m.51327	PREDICTED: solanesyl diphosphate synthase 3, chloroplastic/mitochondrial-like, partial [Ziziphus jujuba]	13.092	2.3417	6.7	1	1	1	0.925	0.887	1.083	1.085	0.986	1.062	1.082	2.91E-01
cds.Camellia_sinensis.comp34165_c1_seq1_m.2418	PREDICTED: malate dehydrogenase [NADP], chloroplastic-like [Juglans regia]	39.089	115.22	36.9	13	41	13	1.011	0.978	1.014	0.977	1.006	0.991	0.99	5.38E-01
cds.Camellia_sinensis.comp34166_c1_seq1_m.6035	PREDICTED: 1,4-alpha-glucan-branching enzyme 2-2, chloroplastic/amyloplastic [Jatropha curcas]	77.526	27.744	11.6	9	13	9	1.026	1.061	1.058	0.945	0.952	0.948	0.905	7.97E-04

cds.Camellia_sinensis.comp34166_c1_seq5_m.48364	PREDICTED: 1,4-alpha-glucan-branching enzyme 2-2, chloroplastic/amyloplastic isoform X2 [Ipomoea nil]	21.48	5.4569	18.2	3	5	3	0.941	1.049	1.071	0.893	1.004	1.047	0.962	5.59E-01
cds.Camellia_sinensis.comp34167_c0_seq10_m.30539	PREDICTED: probable inactive shikimate kinase like 2, chloroplastic isoform X1 [Gossypium raimondii]	38.936	18.861	13.6	3	3	3	0.993	1.002	0.951	0.986	0.98	1.098	1.04	3.97E-01
cds.Camellia_sinensis.comp34171_c0_seq3_m.31358	PREDICTED: ATP-dependent zinc metalloprotease FTSH 10, mitochondrial [Theobroma cacao]	19.986	1.8133	5	1	1	1								

cds.Camellia_sinensis.comp34177_c0_seq1_m.15509	PREDICTED: chaperonin CPN60-2, mitochondrial-like [Nelumbo nucifera]	63.01	299.88	56	32	89	23	1	0.973	0.986	1.033	1.007	1.022	1.035	3.41E-02
cds.Camellia_sinensis.comp34179_c0_seq13_m.38394	PREDICTED: transmembrane protein 120 homolog [Solanum lycopersicum]	29.474	1.7629	7.4	2	3	1								
cds.Camellia_sinensis.comp34181_c0_seq1_m.5894	PREDICTED: beta-galactosidase 8 [Vitis vinifera]	91.583	88.547	14.2	11	20	8	1.05	1.063	1.074	0.934	0.929	0.925	0.875	4.35E-05
cds.Camellia_sinensis.comp34189_c0_seq1_m.9539	PREDICTED: protein NUCLEAR FUSION DEFECTIVE 5, mitochondrial [Vitis vinifera]	87.668	6.402	5.8	5	5	5	1.012	1	0.999	0.915	1.017	1.057	0.993	8.52E-01

cds.Camellia_sinensis.comp34190_c0_seq1_m.8571	PREDICTED: nuclear pore complex protein NUP93A [Vitis vinifera]	78.532	12.872	10.1	7	8	7	0.997	1.008	0.948	1.041	0.959	1.06	1.036	3.88E-01
cds.Camellia_sinensis.comp34190_c0_seq6_m.52408	PREDICTED: nuclear pore complex protein NUP93A-like [Camelina sativa]	18.523	12.529	13.4	2	3	2	0.963	0.988	0.998	1.046	0.996	1.058	1.051	8.05E-02
cds.Camellia_sinensis.comp34192_c0_seq1_m.6581	PREDICTED: protein pleiotropic regulatory locus 1 [Vitis vinifera]	50.665	3.2312	2.9	1	1	1	0.886	1.052	0.957	1.077	0.984	1.072	1.082	2.35E-01
cds.Camellia_sinensis.comp34195_c1_seq15_m.37697	PREDICTED: nucleosome assembly protein 1;4 isoform X3 [Nelumbo nucifera]	34.725	6.474	3.6	1	1	1	0.872	1.109	0.915	0.93	1.145	1.052	1.08	4.55E-01

cds.Camellia_sinensis.comp34197_c0_seq1_m.11738	PREDICTED: serine hydroxymethyltransferase 3, chloroplastic-like [Juglans regia]	56.738	171.64	40	20	43	20	1.007	1.004	0.999	0.983	0.983	1.012	0.989	3.40E-01
cds.Camellia_sinensis.comp34199_c1_seq1_m.13823	PREDICTED: serine/threonine-protein phosphatase 2A 65 kDa regulatory subunit A beta isoform [Jatropha curcas]	65.686	30.549	16.2	9	14	2								
cds.Camellia_sinensis.comp34199_c1_seq2_m.14936	PREDICTED: serine/threonine-protein phosphatase 2A 65 kDa regulatory subunit A beta isoform [Cucumis melo]	65.673	7.1609	13.5	8	13	1								

cds.Camellia_sinensis.comp34203_c0_seq1_m.10841	PREDICTED: protein SIEVE ELEMENT OCCLUSION B-like [Juglans regia]	86.102	169.64	29.8	23	107	22	0.94	0.94	0.93	1.069	1.084	1.083	1.152	1.81E-05
cds.Camellia_sinensis.comp34203_c0_seq2_m.52934	PREDICTED: protein SIEVE ELEMENT OCCLUSION B [Vitis vinifera]	18.907	3.1796	13.2	2	5	1	0.879	0.995	0.922	1.085	1.156	1.002	1.16	5.44E-02
cds.Camellia_sinensis.comp34205_c0_seq7_m.3600	PREDICTED: protein translocase subunit SECA2, chloroplastic isoform X2 [Cucumis sativus]	30.71	2.5828	3.7	1	1	1	0.974	0.944	1.077	1.065	0.974	0.985	1.01	8.41E-01
cds.Camellia_sinensis.comp34215_c0_seq4_m.366	PREDICTED: proteasome activator subunit 4 [Vitis vinifera]	189.89	42.092	7.2	11	11	11	0.975	1.012	1.017	0.99	1.018	0.992	0.999	9.42E-01

cds.Camellia_sinensis.comp34215_c0_seq58_m.42722	PREDICTED: proteasome activator subunit 4 [Solanum tuberosum	13.701	3.7334	13.6	2	2	2	0.925	1.074	1.057	0.887	1.031	1.033	0.966	6.37E-01
cds.Camellia_sinensis.comp34219_c0_seq6_m.53809	PREDICTED: D-aminoacyl-tRNA deacylase-like isoform X1 [Sesamum indicum]	12.778	1.4169	12.5	1	1	1								
cds.Camellia_sinensis.comp34220_c1_seq1_m.11670	PREDICTED: GTP-binding protein TypA/BipA homolog [Citrus	76.152	87.425	14.1	8	11	8	0.994	0.99	0.97	1.028	1.025	0.997	1.032	6.08E-02
cds.Camellia_sinensis.comp34223_c0_seq13_c0_seq1_m.7172	PREDICTED: WD repeat-containing protein 48 isoform X2 [Vitis vinifera]	83.43	3.5801	3.1	3	3	3	0.996	1.033	0.94	0.945	1.053	1.039	1.023	6.38E-01

cds.Camellia_sinensis.comp34227_c0_seq1_m.5314	PREDICTED: vacuolar protein sorting-associated protein 53 A isoform X1 [Vitis vinifera]	91.929	9.1242	4.4	3	3	3	1.076	1.012	1.009	0.898	0.919	1.073	0.933	2.95E-01
cds.Camellia_sinensis.comp34230_c0_seq14_m.26616	PREDICTED: ubiquitin-like modifier-activating enzyme 5 isoform X1 [Theobroma cacao]	49.031	10.752	10.1	4	7	4	0.958	0.987	1.007	1.055	1.011	1.034	1.05	6.16E-02
cds.Camellia_sinensis.comp34231_c0_seq1_m.1045	Inactive ubiquitin carboxyl-terminal hydrolase 54 [Morus notabilis]	147.88	2.0751	0.8	1	1	1								
cds.Camellia_sinensis.comp34232_c0_seq1_m.11671	PREDICTED: D-lactate dehydrogenase [cytochrome], mitochondrial [Sesamum indicum]	62.078	27.377	17.7	10	12	10	1.016	0.985	0.999	0.939	1.025	1.012	0.992	7.78E-01

cds.Camellia_sinensis.comp34233_c0_seq8_m.18519	PREDICTED: insulin-degrading enzyme-like 1, peroxisomal [Vitis vinifera]	63.407	14.863	9.4	6	8	6	0.934	0.961	0.981	1.064	1.042	1.045	1.096	4.28E-03
cds.Camellia_sinensis.comp34234_c0_seq1_m.9086	PREDICTED: long chain acyl-CoA synthetase 6, peroxisomal [Vitis vinifera]	76.776	52.236	17.5	10	12	8	0.927	0.937	0.912	1.092	1.058	1.052	1.153	5.22E-04
cds.Camellia_sinensis.comp34234_c0_seq2_m.11139	PREDICTED: long chain acyl-CoA synthetase 4 [Sesamum indicum]	47.856	17.676	16	8	11	8	0.957	0.945	1.024	1.028	1.02	1.066	1.064	9.17E-02
cds.Camellia_sinensis.comp34234_c0_seq4_m.33542	PREDICTED: long chain acyl-CoA synthetase 6, peroxisomal [Ipomoea nil]	42.515	3.6646	9.1	3	4	1	1.017	1	0.951	1.061	0.946	1.036	1.025	5.82E-01

cds.Camellia_sinensis.comp34234_c0_seq5_m.34016	PREDICTED: long chain acyl-CoA synthetase 4 [Camelina sativa]	18.244	19.357	28.2	5	8	5	0.978	0.991	0.955	1.047	1.025	1.017	1.056	1.67E-02
cds.Camellia_sinensis.comp34234_c1_seq1_m.26920	PREDICTED: protein DJ-1 homolog B-like [Gossypium raimondii]	28.292	38.337	46	9	21	9	0.989	0.981	0.942	1.048	1.048	1.04	1.077	8.24E-03
cds.Camellia_sinensis.comp34234_c1_seq1_m.26921	PREDICTED: protein DJ-1 homolog B-like [Gossypium hirsutum]	20.874	20.78	10.7	3	6	3	1.007	0.969	1.005	1.006	0.972	1.044	1.014	6.08E-01
cds.Camellia_sinensis.comp34235_c0_seq12_m.36539	PREDICTED: nuclear transcription factor Y subunit C-9-like [Juglans regia]	27.116	4.6883	12	3	4	2	0.824	0.979	0.938	1.111	1.066	1.13	1.206	2.54E-02

cds.Camellia_sinensis.comp34237_c2_seq1_m.4193	PREDICTED: beta-galactosidase [Theobroma cacao]	124.74	97.904	19.1	20	28	20	0.953	0.915	0.943	1.064	1.08	1.07	1.143	4.95E-04
cds.Camellia_sinensis.comp34240_c0_seq7_m.27449	PREDICTED: ATPase 5, plasma membrane-type-like [Pyrus x bretschneideri]	40.722	5.5014	21.9	8	13	3	0.926	0.881	1.028	1.083	1.031	1.084	1.128	6.49E-02
cds.Camellia_sinensis.comp34240_c1_seq10_m.17883	PREDICTED: plasma membrane ATPase 4-like [Pyrus x bretschneideri]	64.498	69.466	38.7	21	40	8	0.963	0.943	0.994	1.074	1.026	1.039	1.082	1.81E-02
cds.Camellia_sinensis.comp34240_c1_seq2_m.39347	PREDICTED: ATPase 11, plasma membrane-type [Theobroma cacao]	25.215	6.8186	25.3	5	12	2	0.995	0.983	1.126	1.042	0.908	0.955	0.936	3.27E-01
cds.Camellia_sinensis.comp34248_c0_seq7_m.20367	PREDICTED: polyadenylate-binding protein RBP47B' [Vitis	50.238	6.7628	6.9	3	4	3	0.964	0.954	0.961	1.035	1.044	1.064	1.092	5.56E-04

cds.Camellia_sinensis.comp34249_c0_seq1_m.1198	PREDICTED: ALG-2 interacting protein X [Vitis vinifera]	77.238	32.773	14.7	10	13	8	0.988	1.023	0.988	1.062	1.074	0.957	1.031	4.84E-01
cds.Camellia_sinensis.comp34249_c0_seq6_m.27601	PREDICTED: ALG-2 interacting protein X [Vitis vinifera]	33.68	2.3031	8.4	3	4	1	1.06	0.98	0.898	1.08	1.047	0.947	1.046	5.01E-01
cds.Camellia_sinensis.comp34252_c0_seq1_m.14690	serine carboxypeptidase S10 family protein [Populus trichocarpa]	54.234	12.881	5.8	2	3	2	1.024	1.075	1.024	0.969	0.961	0.945	0.921	9.76E-03
cds.Camellia_sinensis.comp34253_c0_seq1_m.1260	transducin family protein [Populus trichocarpa]	164.82	36.503	7.7	11	13	11	0.973	1.05	0.962	1.028	1.012	1.006	1.02	4.99E-01
cds.Camellia_sinensis.comp34253_c1_seq2_m.45510	PREDICTED: actin-depolymerizing factor 2 [Nelumbo nucifera]	15.575	7.7002	17.3	2	3	1	1.202	1.063	0.878	0.889	0.922	1.015	0.899	3.74E-01

cds.Camellia_sinensis.comp34256_c0_seq2_m.4266	PREDICTED: DExH-box ATP-dependent RNA helicase DExH1 isoform X3 [Nelumbo nucifera]	52.217	3.0576	3.7	1	1	1								
cds.Camellia_sinensis.comp34256_c0_seq6_m.34640	PREDICTED: proteasome assembly chaperone 2 [Juglans regia]	31.034	4.0489	7.1	2	3	2	0.957	0.94	1.033	1.006	1.006	1.076	1.054	2.22E-01
cds.Camellia_sinensis.comp34263_c0_seq2_m.23362	PREDICTED: heterogeneous nuclear ribonucleoprotein 1-like [Ziziphus jujuba]	36.715	150.85	33	11	27	10	1.006	0.977	0.94	1.012	1.038	1.053	1.062	5.82E-02
cds.Camellia_sinensis.comp34268_c0_seq11_m.13029	PREDICTED: calcium-dependent protein kinase 13 isoform X1 [Vitis vinifera]	38.172	2.3621	3.5	1	1	1								

cds.Camellia_sinensis.comp34268_c0_seq14_m.18047	PREDICTED: cleft lip and palate transmembrane protein 1 homolog [Populus euphratica]	70.701	4.2399	3.9	2	3	2	1.011	1.212	1.057	1.226	0.716	0.782	0.83	2.94E-01
cds.Camellia_sinensis.comp34274_c0_seq5_m.7141	PREDICTED: far upstream element-binding protein 1 isoform X2 [Vitis vinifera]	43.512	50.677	29.9	8	13	2	0.984	1.067	0.943	0.896	1.004	1.107	1.004	9.80E-01
cds.Camellia_sinensis.comp34274_c0_seq6_m.7377	PREDICTED: far upstream element-binding protein 1 isoform X2 [Vitis vinifera]	43.918	11.689	30.2	7	12	1								
cds.Camellia_sinensis.comp34275_c0_seq5_m.32368	PREDICTED: N-carbamoyl putrescine amidase-like [Juglans regia]	33.158	35.213	21.5	5	9	5	0.962	0.924	0.948	1.081	1.057	1.069	1.132	7.77E-04

cds.Camellia_sinensis.comp34277_c0_seq1_m.15838	PREDICTED: chitinase-like protein PB1E7.04c [Nelumbo nucifera]	64.873	1.8879	1.5	1	1	1	1.033	1.074	1.015	0.974	0.926	0.973	0.92	2.44E-02
cds.Camellia_sinensis.comp34279_c0_seq20_m.34635	PREDICTED: HVA22-like protein a isoform X1 [Sesamum indicum]	13.946	3.6905	23.3	2	3	2								
cds.Camellia_sinensis.comp34283_c0_seq1_m.6318	PREDICTED: nuclear cap-binding protein subunit 1 isoform X2 [Vitis vinifera]	98.126	40.113	11	7	9	7	0.947	1.003	1.005	1.024	0.998	1.053	1.041	1.84E-01
cds.Camellia_sinensis.comp34284_c0_seq1_m.5548	PREDICTED: DEAD-box ATP-dependent RNA helicase 46 [Ziziphus jujuba]	30.838	1.3184	7.9	2	3	1								

cds.Camellia_sinensis.comp34285_c0_seq1_m.3525	PREDICTED: cullin-4-like [Solanum pennellii]	86.326	11.251	7.2	6	7	6	1.006	1.007	0.998	0.991	1.028	1.01	1.006	6.21E-01
cds.Camellia_sinensis.comp34287_c0_seq1_m.12262	DPP6 N-terminal domain-like protein [Arabidopsis thaliana]	77.632	137.24	30	17	31	17	1.057	1.018	0.998	0.973	0.979	0.974	0.952	1.03E-01
cds.Camellia_sinensis.comp34292_c0_seq9_m.10938	PREDICTED: 1,2-dihydroxy-3-keto-5-methylthiopentene dioxygenase 1 [Vitis vinifera]	24.088	9.6973	20.9	5	5	5	1.064	1.179	1.016	0.847	0.908	0.961	0.833	3.36E-02
cds.Camellia_sinensis.comp34294_c1_seq1_m.16054	PREDICTED: protein SUPPRESSOR OF GENE SILENCING 3 homolog [Vigna angularis]	52.393	16.872	12.8	6	6	6	1.189	1.137	1.201	0.812	0.834	0.805	0.695	5.64E-05

cds.Camellia_sinensis.comp34294_c4_seq1_m.1774	PREDICTED: alpha-glucan water dikinase, chloroplastic isoform X1 [Vitis vinifera]	158.71	135.59	21.4	28	36	28	1.047	1.032	1.064	0.955	0.964	0.928	0.906	2.38E-03
cds.Camellia_sinensis.comp34294_c4_seq7_m.53283	PREDICTED: plant UBX domain-containing protein 8-like isoform X1 [Nicotiana attenuata]	13.459	5.8208	27.4	3	3	3	1.015	1.083	1.084	0.911	0.937	0.961	0.883	9.56E-03
cds.Camellia_sinensis.comp34295_c0_seq1_m.18899	PREDICTED: serine carboxypeptidase-like 40 [Citrus sinensis]	56.083	11.229	10.8	4	4	4	0.963	0.99	1.103	1.011	0.957	0.989	0.968	5.18E-01
cds.Camellia_sinensis.comp34297_c0_seq1_m.1942	PREDICTED: Niemann-Pick C1 protein [Vitis vinifera]	142.73	7.8943	3.2	4	4	4	1.02	1.006	1.083	1.033	0.955	0.908	0.931	1.76E-01

cds.Camellia_sinensis.comp34297_c0_seq7_m.2291	PREDICTED: Niemann-Pick C1 protein isoform X2 [Vitis vinifera]	86.649	5.5979	1.4	1	1	1	1.062	1.111	0.997	0.933	0.965	0.921	0.889	2.74E-02
cds.Camellia_sinensis.comp34297_c0_seq7_m.2292	PREDICTED: Niemann-Pick C1 protein isoform X2 [Vitis vinifera]	35.217	2.4454	6.2	1	1	1	1.107	0.924	0.922	0.96	1.044	1.042	1.031	6.37E-01
cds.Camellia_sinensis.comp34298_c0_seq3_m.11240	PREDICTED: exocyst complex component SEC6 [Vitis vinifera]	88.748	9.3636	5.2	4	5	4	0.984	1.044	1.047	0.989	0.97	0.971	0.953	8.64E-02
cds.Camellia_sinensis.comp34299_c2_seq4_m.53960	PREDICTED: photosynthetic NDH subunit of lumenal location 5, chloroplastic [Ziziphus jujuba]	15.568	5.7357	19.7	2	5	1	1.055	1.139	1.009	0.944	0.892	0.947	0.869	2.58E-02

cds.Camellia_sinensis.comp34299_c3_seq1_m.27800	PREDICTED: mitochondrial outer membrane protein porin of 34 kDa [Nicotiana attenuata]	29.486	89.402	33	10	25	6	1.023	0.979	0.96	0.978	1.022	1.018	1.019	4.66E-01
cds.Camellia_sinensis.comp34299_c3_seq2_m.31009	PREDICTED: mitochondrial outer membrane protein porin of 34 kDa [Nicotiana attenuata]	31.604	127.01	43.4	12	28	8	1.003	0.963	0.988	0.994	1.019	1.011	1.024	1.67E-01
cds.Camellia_sinensis.comp34303_c0_seq1_m.1171	Serine/threonine-protein kinase [Morus notabilis]	128.74	11.375	4.8	6	6	4	0.943	1.094	0.977	1.01	0.952	1	0.983	7.62E-01
cds.Camellia_sinensis.comp34303_c0_seq1_m.1172	PREDICTED: MLP-like protein 423 isoform X2 [Ziziphus jujuba]	17.014	106.91	51.7	9	20	9	1.255	1.332	1.276	0.671	0.675	0.674	0.523	7.58E-04

cds.Camellia_sinensis.comp34303_c0_seq2_m.1564	Serine/threonine-protein kinase [Morus notabilis]	150.83	1.8473	2.2	3	3	2	0.969	0.997	1.002	0.975	1.064	1.007	1.026	4.09E-01
cds.Camellia_sinensis.comp34308_c0_seq1_m.15055	PREDICTED: T-complex protein 1 subunit theta [Ziziphus jujuba]	66.985	174.46	40.7	21	37	21	0.956	0.951	0.945	1.079	1.064	1.057	1.122	7.86E-05
cds.Camellia_sinensis.comp34318_c0_seq2_m.34825	PREDICTED: probable zinc metalloprotease EGY1, chloroplastic [Cucumis sativus]	33.297	5.5672	7	2	3	2	1.057	1.012	0.998	1.004	0.944	0.984	0.956	1.47E-01
cds.Camellia_sinensis.comp34320_c0_seq5_m.4979	PREDICTED: putative GTP diphosphokinase RSH1, chloroplastic isoform X2 [Citrus sinensis]	61.549	2.0247	1.5	1	1	1	1.12	1.116	1.086	0.869	0.896	0.884	0.797	6.35E-05

cds.Camellia_sinensis.comp34323_c2_seq1_m.31968	PREDICTED: poly(rC)-binding protein 4-like [Nicotiana tomentosiformis]	29.299	1.2756	3.6	1	1	1	0.933	1.062	1.03	1.061	0.957	0.975	0.989	8.50E-01
cds.Camellia_sinensis.comp34328_c0_seq20_m.15224	PREDICTED: DNA polymerase delta catalytic subunit [Sesamum indicum]	70.096	1.3498	1.3	1	1	1	1.015	0.983	0.985	0.987	1.023	1.014	1.014	4.13E-01
cds.Camellia_sinensis.comp34331_c1_seq1_m.11534	PREDICTED: transmembrane 9 superfamily member 7 [Glycine max]	57.195	8.8688	4.8	2	2	1	1.071	1.092	0.972	1.058	0.895	0.909	0.913	2.23E-01
cds.Camellia_sinensis.comp34331_c1_seq2_m.12395	PREDICTED: transmembrane 9 superfamily member 7 [Gossypium hirsutum]	13.744	27.575	18.2	2	3	1	0.987	1.023	1.033	1.055	1.003	0.912	0.976	5.95E-01

cds.Camellia_sinensis.comp34335_c1_seq1_m.24901	PREDICTED: beta-hexosaminidase 2 [Vitis vinifera]	55.016	3.6431	4.2	2	2	2	1.039	1.144	1.103	1.021	0.787	0.893	0.822	6.65E-02
cds.Camellia_sinensis.comp34337_c0_seq1_m.6146	PREDICTED: helicase-like transcription factor CHR28 isoform X1 [Vitis vinifera]	98.952	11.483	1.6	1	1	1	0.951	0.914	0.922	1.12	1.117	1.013	1.166	1.22E-02
cds.Camellia_sinensis.comp34341_c0_seq3_m.4779	PREDICTED: importin beta-like SAD2 [Ziziphus jujuba]	52.991	5.4683	5	2	2	2								
cds.Camellia_sinensis.comp34342_c0_seq1_m.3848	PREDICTED: probable starch synthase 4, chloroplastic/amyloplastic [Vitis vinifera]	114.51	24.798	9.2	7	9	7	0.995	1.166	1.047	0.95	0.916	0.948	0.877	5.55E-02
cds.Camellia_sinensis.comp34343_c0_seq1_m.1828	dentin sialophosphoprotein [Populus trichocarpa]	164.05	35.27	8.7	12	13	12	1.025	1.062	1.039	1.022	0.962	0.952	0.939	6.10E-02

cds.Camellia_sinensis.comp34350_c0_seq10_m.46921	PREDICTED: probable alpha-mannosidase At5g13980 [Vitis vinifera]	24.634	9.9395	21.7	4	12	2	0.986	1.021	0.891	1.012	1.003	1.1	1.075	2.21E-01
cds.Camellia_sinensis.comp34350_c0_seq6_m.4895	PREDICTED: alpha-mannosidase isoform X2 [Gossypium raimondii]	115.64	36.673	12	13	19	13	0.998	1.022	1.033	0.962	0.986	0.988	0.962	4.27E-02
cds.Camellia_sinensis.comp34352_c0_seq2_m.1656	PREDICTED: MAP3K epsilon protein kinase 1 isoform X1 [Vitis vinifera]	152.21	1.8503	0.7	1	1	1	1.027	1.107	0.934	0.918	0.979	1.029	0.954	4.80E-01
cds.Camellia_sinensis.comp34354_c0_seq1_m.18787	PREDICTED: 7-deoxyloganetin glucosyltransferase-like isoform X4 [Prunus	55.626	2.6867	2.8	1	1	1								

cds.Camellia_sinensis.comp34360_c0_seq26_m.5184	K(+) efflux antiporter 2 [Morus notabilis]	12.95	4.0614	14.6	1	1	1								
cds.Camellia_sinensis.comp34364_c0_seq4_m.4091	PREDICTED: villin-3-like [Citrus sinensis]	104.08	185	32.7	26	38	26	0.975	0.96	0.958	1.03	1.023	1.057	1.075	3.18E-03
cds.Camellia_sinensis.comp34368_c0_seq1_m.18665	PREDICTED: farnesylcysteine lyase [Theobroma cacao]	60.529	1.563	3.5	2	2	2								
cds.Camellia_sinensis.comp34371_c3_seq6_m.43394	PREDICTED: expansin-A10 [Theobroma cacao]	14.598	4.6309	7.5	1	2	1	0.996	1.028	0.931	1.034	0.999	1.025	1.035	3.22E-01
cds.Camellia_sinensis.comp34374_c0_seq14_m.23217	PREDICTED: cytochrome c1-2, heme protein, mitochondrial [Citrus sinensis]	39.622	27.58	20.8	5	14	3	0.962	1.063	0.954	1.033	0.994	1.009	1.019	6.12E-01

cds.Camellia_sinensis.comp34374_c0_seq2_m.23931	PREDICTED: cytochrome c1-2, heme protein, mitochondrial [Nicotiana attenuata]	39.537	96.985	25.6	6	19	4	0.936	1.047	0.957	1.073	0.998	1.035	1.056	2.38E-01
cds.Camellia_sinensis.comp34376_c0_seq16_c0_seq1_m.7966	PREDICTED: beta-adaptin-like protein C [Solanum pennellii]	87.647	78.468	17.5	12	24	12	0.966	0.984	1.019	1.052	1.027	0.995	1.035	1.97E-01
cds.Camellia_sinensis.comp34376_c0_seq8_m.44486	PREDICTED: DNA-directed RNA polymerase II subunit 4 [Jatropha	13.233	2.8975	14.9	2	3	2	0.983	0.949	0.941	1.033	1.02	1.092	1.095	2.22E-02
cds.Camellia_sinensis.comp34380_c0_seq3_m.15281	PREDICTED: serine/threonine-protein phosphatase 6 regulatory subunit 3 isoform X4 [Vitis vinifera]	74.971	2.2651	3.2	2	2	2	1.193	1.072	1.09	0.746	0.859	0.992	0.774	4.20E-02

cds.Camellia_sinensis.comp34384_c0_seq3_m.25787	PREDICTED: glycine--tRNA ligase 1, mitochondrial-like [Nicotiana sylvestris]	51.633	17.204	31.7	13	22	4	0.95	0.931	0.938	1.057	1.075	1.052	1.129	1.60E-04
cds.Camellia_sinensis.comp34384_c1_seq74_m.22974	PREDICTED: conserved oligomeric Golgi complex subunit 1 [Vitis vinifera]	46.186	14.842	4.4	2	2	2	1.06	1.019	1.081	0.89	1	0.939	0.895	4.23E-02
cds.Camellia_sinensis.comp34386_c0_seq1_m.3887	PREDICTED: 26S proteasome non-ATPase regulatory subunit 1 homolog A [Vitis vinifera]	103.45	123.65	16.2	13	24	11	0.983	0.977	1.003	1.041	1.004	1.016	1.033	7.07E-02
cds.Camellia_sinensis.comp34386_c0_seq2_m.36640	PREDICTED: 26S proteasome non-ATPase regulatory subunit 1 homolog A-like [Prunus mume]	24.693	32.551	32.9	7	12	5	0.99	1.012	0.96	1.018	0.979	1.02	1.019	4.15E-01

cds.Camellia_sinensis.comp34387_c0_seq3_m.4957	PREDICTED: ATP-dependent zinc metalloprotease FTSH 9, chloroplastic isoform X2 [Ziziphus jujuba]	90.027	3.2078	4.1	3	3	3	0.947	1.076	1.062	1.081	0.948	0.902	0.95	4.80E-01
cds.Camellia_sinensis.comp34391_c0_seq31_m.16332	PREDICTED: probable serine/threonine-protein phosphatase 2A regulatory subunit B" subunit TON2 [Phoenix dactylifera]	54.772	3.6363	4.6	3	3	3	1.03	1.113	1.086	0.934	0.911	0.902	0.851	3.02E-03
cds.Camellia_sinensis.comp34393_c0_seq93_m.28437	PREDICTED: phosphatase IMPL1, chloroplastic [Daucus carota subsp. sativus]	41.26	41.241	14	4	4	4	1.021	0.975	1.01	0.935	0.992	1.05	0.99	7.84E-01

cds.Camellia_sinensis.comp34394_c0_seq8_m.5071	PREDICTED: protein MEI2-like 5 [Vitis vinifera]	71.595	2.5959	3.3	2	2	2	1.061	1.128	1.014	0.991	0.859	0.935	0.869	5.31E-02
cds.Camellia_sinensis.comp34396_c0_seq2_m.38667	PREDICTED: polyadenylate-binding protein 2 isoform X1	24.789	24.247	19.9	4	5	3	1.019	1.052	0.981	1.018	0.993	0.943	0.968	3.40E-01
cds.Camellia_sinensis.comp34396_c2_seq6_m.44576	PREDICTED: subtilisin-like protease [Sesamum indicum]	15.093	1.458	11.6	1	1	1								
cds.Camellia_sinensis.comp34398_c0_seq18_m.2676	PREDICTED: carbamoyl-phosphate synthase large chain, chloroplastic-like [Ipomoea nil]	104.3	124.56	19.2	17	30	17	0.982	0.984	0.969	1.022	1.033	1.018	1.047	2.12E-03

cds.Camellia_sinensis.comp34398_c0_seq1_m.2677	PREDICTED: carbamoyl-phosphate synthase large chain, chloroplastic [Vitis vinifera]	28.476	6.8659	13.3	4	7	4	0.992	0.989	0.993	0.989	1.04	1.038	1.031	2.05E-01
cds.Camellia_sinensis.comp34399_c1_seq4_m.6514	PREDICTED: importin subunit alpha-2 [Vitis vinifera]	58.401	13.971	20.2	10	12	2	1.066	1.004	1.018	0.929	1.02	0.956	0.941	1.36E-01
cds.Camellia_sinensis.comp34399_c1_seq4_m.6515	RAB GTPase-like protein A5B [Medicago truncatula]	23.924	13.406	40.3	9	20	2	0.916	0.962	0.966	1.072	1.043	1.01	1.099	1.77E-02
cds.Camellia_sinensis.comp34399_c1_seq9_m.15209	PREDICTED: importin subunit alpha-2 [Jatropha curcas]	58.693	46.464	24.1	11	13	3	1.001	1.057	0.963	0.987	0.931	1.065	0.987	7.91E-01
cds.Camellia_sinensis.comp34400_c0_seq12_m.17770	PREDICTED: cullin-1-like isoform X2 [Nicotiana tabacum]	63.353	4.209	9.2	5	5	1	1.032	1.067	1.085	0.953	0.916	0.941	0.883	2.66E-03

cds.Camellia_sinensis.comp34400_c0_seq4_m.8026	PREDICTED: cullin-1 isoform X1 [Vitis vinifera]	86.372	16.424	7.8	6	6	2								
cds.Camellia_sinensis.comp34400_c0_seq9_m.12194	PREDICTED: granule-bound starch synthase 1, chloroplastic/amyloplastic isoform X2 [Theobroma cacao]	67.174	39.882	21.9	12	21	11	1.242	1.24	1.25	0.718	0.734	0.744	0.588	4.87E-07
cds.Camellia_sinensis.comp34400_c1_seq1_m.48780	thionin-like protein [Arabidopsis thaliana]	22.293	6.1601	14.3	2	2	2	0.947	1.095	1.066	0.911	0.891	1.088	0.93	3.88E-01
cds.Camellia_sinensis.comp34401_c1_seq1_m.16024	PREDICTED: NAD-dependent malic enzyme 59 kDa isoform, mitochondrial [Sesamum indicum]	66.219	78.478	27.3	15	22	11	1.002	0.991	0.977	0.999	1.018	1.001	1.016	1.64E-01

cds.Camellia_sinensis.comp34401_c1_seq3_m.37084	PREDICTED: NAD-dependent malic enzyme 59 kDa isoform, mitochondrial [Juglans regia]	27.044	40.641	30.7	7	14	4	0.981	0.96	0.944	1.027	1.063	1.055	1.09	4.82E-03
cds.Camellia_sinensis.comp34401_c2_seq1_m.38626	PREDICTED: chorismate mutase 2 [Nelumbo nucifera]	29.023	15.511	33.2	7	9	7	0.988	0.991	1.008	1.041	0.99	1.031	1.025	2.13E-01
cds.Camellia_sinensis.comp34403_c0_seq2_m.16300	PREDICTED: protein decapping 5 isoform X1 [Vitis vinifera]	63.679	15.289	11.4	6	6	6	1.022	0.988	1.061	0.978	0.982	0.962	0.951	8.36E-02
cds.Camellia_sinensis.comp34406_c0_seq1_m.24451	PREDICTED: UDP-glucose flavonoid 3-O-glucosyltransferase 6-like [Fragaria vesca subsp. vesca]	41.357	6.3523	2.9	1	2	1								

cds.Camellia_sinensis.comp34406_c0_seq6_m.55028	PREDICTED: anthocyanidin 3-O-glucosyltransferase 2-like [Daucus carota subsp. sativus]	13.482	1.8133	7.4	1	1	1	1.036	0.972	1.003	1.003	1.029	0.963	0.995	8.51E-01
cds.Camellia_sinensis.comp34407_c0_seq17_m.15211	PREDICTED: diaminopimelate decarboxylase 2, chloroplastic [Vitis vinifera]	52.851	117.76	30.4	13	25	13	1.016	1.021	0.998	0.985	1.002	0.986	0.98	8.07E-02
cds.Camellia_sinensis.comp34409_c0_seq2_m.1102	sulfate adenylyltransferase subunit [Arabidopsis thaliana]	23.254	48.53	12.9	2	3	2	0.914	0.928	1.01	1.007	1.048	1.12	1.113	7.09E-02
cds.Camellia_sinensis.comp34413_c0_seq23_m.26389	PREDICTED: ribulose bisphosphate carboxylase/oxygenase activase, chloroplastic [Cicer arietinum]	49.902	28.752	13.8	6	8	6	1.024	1.058	1.047	0.894	0.979	0.951	0.903	2.23E-02

cds.Camellia_sinensis.comp34414_c0_seq2_m.24049	PREDICTED: probable inactive shikimate kinase like 1, chloroplastic isoform X4 [Vitis vinifera]	33.47	22.122	14.6	4	6	4	1.052	0.962	0.951	1.003	0.984	1.009	1.01	7.46E-01
cds.Camellia_sinensis.comp34415_c1_seq1_m.31227	chaperonin-like RbcX protein 2, chloroplastic [Cajanus cajan]	25.662	2.7286	4.9	1	1	1	1.024	1.053	1.062	1.01	0.956	0.898	0.912	6.02E-02
cds.Camellia_sinensis.comp34415_c2_seq1_m.57003	PREDICTED: cysteine proteinase inhibitor [Juglans regia]	13.541	2.0271	14.3	2	2	2	0.874	0.95	0.968	1.089	1.061	1.097	1.163	1.00E-02
cds.Camellia_sinensis.comp34416_c0_seq1_m.6374	putative cytoskeletal protein mRNA [Populus trichocarpa]	85.626	10.155	7.9	7	8	7	1.168	1.074	1.135	0.845	0.812	0.882	0.752	1.14E-03

cds.Camellia_sinensis.comp34418_c1_seq1_m.3485	PREDICTED: valine--tRNA ligase, mitochondrial 1 isoform X2 [Vitis vinifera]	121.15	58.702	11.3	12	13	11	1.054	1.113	1.106	0.947	0.883	0.902	0.835	2.56E-03
cds.Camellia_sinensis.comp34418_c2_seq1_m.25656	PREDICTED: alpha-galactosidase 1-like [Nelumbo nucifera]	45.832	155.78	40.1	15	33	13	0.923	0.92	0.912	1.1	1.068	1.088	1.182	5.86E-05
cds.Camellia_sinensis.comp34423_c0_seq1_m.1154	PREDICTED: phosphatidylinositol 4-kinase alpha 1 isoform X1 [Theobroma cacao]	196.84	3.1816	1.7	3	3	3	1.075	1.075	0.999	0.948	0.945	0.947	0.902	5.23E-02
cds.Camellia_sinensis.comp34432_c0_seq6_m.4366	PREDICTED: homeobox-leucine zipper protein ATHB-15 [Vitis vinifera]	93.697	1.3829	1.1	1	1	1								

cds.Camellia_sinensis.comp34434_c1_seq2_m.33206	mitochondrial glycoprotein [Populus trichocarpa]	27.672	11.641	15.2	3	3	3	0.898	1.071	0.951	1.118	1.024	0.969	1.065	3.88E-01
cds.Camellia_sinensis.comp34436_c0_seq1_m.23561	PREDICTED: putative GPI-anchor transamidase [Vitis vinifera]	47.14	4.5401	4.1	1	1	1								
cds.Camellia_sinensis.comp34438_c1_seq5_m.21556	PREDICTED: peptidyl-prolyl cis-trans isomerase CYP37, chloroplastic [Jatropha curcas]	34.068	61.016	27.7	8	18	8	1.013	0.954	0.96	1.041	1.044	1.033	1.065	3.01E-02
cds.Camellia_sinensis.comp34438_c1_seq5_m.21557	PREDICTED: peptidyl-prolyl cis-trans isomerase CYP37, chloroplastic [Vigna angularis]	12.34	3.6704	18.8	3	5	3	0.986	0.939	1.018	1.065	1.033	1.032	1.064	7.29E-02

cds.Camellia_sinensis.comp34439_c0_seq1_m.36377	PREDICTED: ATP-dependent zinc metalloprotease FTSH 11, chloroplastic/mitochondrial-like [Vigna angularis]	29.058	12.512	16.1	5	5	5	0.981	0.972	1	1.036	0.938	1.011	1.011	7.64E-01
cds.Camellia_sinensis.comp34443_c0_seq13_m.11218	PREDICTED: V-type proton ATPase catalytic subunit A [Nelumbo	68.663	323.31	53.9	34	78	34	0.97	0.976	0.972	1.025	1.042	1.035	1.063	2.80E-04
cds.Camellia_sinensis.comp34444_c0_seq6_m.37666	K(+) efflux antiporter 2, chloroplastic-like [Cajanus	28.159	36.533	25	5	9	5	1.037	1.062	1.086	0.976	0.917	0.964	0.897	9.44E-03
cds.Camellia_sinensis.comp34446_c0_seq1_m.10981	PREDICTED: proton pump-interactor 1 [Vitis vinifera]	72.596	30.611	14.3	8	9	8	1.081	1.137	1.164	0.882	0.896	0.85	0.777	7.18E-04

cds.Camellia_sinensis.comp34446_c2_seq1_m.19110	PREDICTED: monogalactosyldiacylglycerol synthase, chloroplastic [Theobroma cacao]	35.826	2.8526	5.2	2	2	2	0.927	0.969	1.023	1.054	1.036	1.017	1.064	1.06E-01
cds.Camellia_sinensis.comp34447_c0_seq1_m.1754	Transducin/WD40 repeat-like superfamily protein [Arabidopsis thaliana]	143.41	32.448	5.3	6	8	6	0.942	0.987	0.987	0.977	1.009	1.058	1.044	2.01E-01
cds.Camellia_sinensis.comp34451_c0_seq1_m.3457	PREDICTED: 110 kDa U5 small nuclear ribonucleoprotein component CLO [Vitis	110.5	7.4798	4.4	3	3	3								
cds.Camellia_sinensis.comp34451_c1_seq1_m.17892	sodium/calcium exchanger family protein [Populus trichocarpa]	64.451	5.3938	6.4	3	4	3	1.099	1.137	1.083	0.846	0.914	0.892	0.799	1.14E-03

cds.Camellia_sinensis.comp34451_c2_seq2_m.41716	PREDICTED: 40S ribosomal protein S13-like [Erythranthe guttata]	17.136	12.895	24.5	4	8	4	1.107	1.092	1.073	0.894	0.925	0.87	0.822	5.95E-04
cds.Camellia_sinensis.comp34452_c0_seq1_m.3869	PREDICTED: probable phosphoinositide phosphatase SAC9 [Vitis vinifera]	120.62	3.2276	1.6	2	2	2	1.031	1.003	1.01	0.956	0.972	1.028	0.971	2.74E-01
cds.Camellia_sinensis.comp34455_c0_seq3_m.6116	PREDICTED: protein FAR1-RELATED SEQUENCE 3 [Vitis	44.559	3.4653	9.3	3	4	1	0.897	1.023	0.906	1.04	1.154	1.014	1.135	9.33E-02
cds.Camellia_sinensis.comp34457_c0_seq1_m.13702	PREDICTED: probable acyl-activating enzyme 17, peroxisomal isoform X2 [Ziziphus	80.7	26.051	10.5	7	12	7	0.962	1.018	1.009	1	1.015	1.046	1.024	3.38E-01

cds.Camellia_sinensis.comp34458_c0_seq1_m.4994	PREDICTED: pentatricopeptide repeat-containing protein At5g46580, chloroplastic [Nicotiana tomentosiformis]	78.976	3.1051	6.2	5	5	2	0.911	1.005	1.019	1.099	0.963	1.031	1.054	3.72E-01
cds.Camellia_sinensis.comp34458_c0_seq5_m.26948	PREDICTED: pentatricopeptide repeat-containing protein At5g46580, chloroplastic [Sesamum indicum]	51.333	11.137	15.9	7	8	4	0.951	0.997	0.93	1.079	1.08	0.993	1.095	5.88E-02
cds.Camellia_sinensis.comp34460_c2_seq11_m.10323	PREDICTED: neutral alpha-glucosidase C [Nicotiana sylvestris]	51.942	105.06	27.8	12	18	12	0.988	0.998	1.033	0.975	0.978	0.993	0.976	1.72E-01

cds.Camellia_sinensis.comp34460_c2_seq19_m.31055	PREDICTED: alpha-glucosidase 2-like [Citrus sinensis]	30.786	9.0573	13.8	4	8	4	0.952	1.029	1.031	0.99	1.037	1.051	1.022	5.28E-01
cds.Camellia_sinensis.comp34460_c2_seq26_m.42758	transmembrane protein, putative [Medicago truncatula]	25.22	5.4741	7.7	2	2	2	0.873	0.873	0.917	1.172	1.113	1.106	1.273	6.02E-04
cds.Camellia_sinensis.comp34466_c0_seq1_m.25934	PREDICTED: DNA-directed RNA polymerases II, IV and V subunit 3 [Theobroma cacao]	35.758	79.419	20.7	5	5	5	0.957	0.922	1.028	1.021	1.039	1.033	1.064	1.23E-01
cds.Camellia_sinensis.comp34468_c1_seq2_m.18467	PREDICTED: ornithine aminotransferase, mitochondrial-like [Juglans regia]	52.303	72.236	33.2	12	26	12	0.943	0.959	0.979	1.04	1.067	1.031	1.089	4.64E-03
cds.Camellia_sinensis.comp34468_c1_seq3_m.20334	PREDICTED: KH domain-containing protein At4g18375-like [Malus domestica]	41.231	1.5079	2.4	1	1	1	1.211	1.186	1.27	0.841	0.722	0.713	0.621	1.08E-03

cds.Camellia_sinensis.comp34468_c1_seq6_m.20890	PREDICTED: KH domain-containing protein At4g18375-like [Prunus mume]	26.268	12.034	12.8	3	4	3	1.156	1.142	1.255	0.789	0.797	0.811	0.675	2.18E-04
cds.Camellia_sinensis.comp34470_c0_seq10_m.5228	PREDICTED: golgin candidate 5 [Jatropha curcas]	109.71	4.4135	2.8	3	3	3	0.948	1.008	1.039	1.034	0.975	1.013	1.009	7.83E-01
cds.Camellia_sinensis.comp34474_c0_seq1_m.4870	PREDICTED: heat shock 70 kDa protein 17 [Nelumbo nucifera]	51.457	7.1507	7.3	3	4	3	0.941	1.099	1.035	1.088	0.954	0.999	0.989	8.67E-01
cds.Camellia_sinensis.comp34474_c0_seq1_m.4871	PREDICTED: heat shock 70 kDa protein 17-like [Populus euphratica]	33.998	4.8345	12.8	3	3	3	0.94	1.058	0.943	1.004	1.068	1.006	1.047	3.48E-01
cds.Camellia_sinensis.comp34480_c0_seq10_m.23118	PREDICTED: glucan endo-1,3-beta-glucosidase 8 [Vitis	52.902	14.094	12.4	5	5	5	0.909	0.95	1.017	1.051	0.938	1.034	1.051	3.61E-01

cds.Camellia_sinensis.comp34481_c0_seq1_m.8834	PREDICTED: protein TOC75-3, chloroplastic-like [Sesamum indicum]	89.873	123.2	27.1	19	32	19	0.98	0.985	0.986	0.998	1.007	1.01	1.022	6.16E-03
cds.Camellia_sinensis.comp34481_c0_seq3_m.20244	PREDICTED: leucine-rich repeat extensin-like protein 4 [Citrus sinensis]	52.66	23.249	7.8	3	5	3	0.918	0.989	1.017	1.006	1.002	1.017	1.035	3.76E-01
cds.Camellia_sinensis.comp34487_c0_seq1_m.16775	PREDICTED: eukaryotic translation initiation factor 2 subunit gamma-like [Jatropha curcas]	50.748	88.553	24.6	10	14	10	0.961	0.95	0.95	1.031	1.058	1.046	1.096	4.03E-04
cds.Camellia_sinensis.comp34487_c0_seq7_m.35493	PREDICTED: MICOS complex subunit mic25a-like isoform X2 [Nelumbo nucifera]	21.575	2.2728	20.5	4	4	4	1.141	1.133	1.106	0.827	0.832	0.92	0.763	1.62E-03

cds.Camellia_sinensis.comp34488_c1_seq1_m.29564	PREDICTED: AB hydrolase superfamily protein YfhM-like [Prunus mume]	43.349	10.761	12.9	6	8	4	0.988	1.005	0.97	1.065	0.987	0.989	1.026	4.00E-01
cds.Camellia_sinensis.comp34488_c1_seq2_m.29851	PREDICTED: bifunctional epoxide hydrolase 2 [Citrus sinensis]	35.255	4.0585	17.7	6	10	4	0.991	0.955	1.033	1.019	1.037	0.983	1.02	5.05E-01
cds.Camellia_sinensis.comp34490_c0_seq1_m.3865	PREDICTED: isoleucine--tRNA ligase, chloroplastic/mitochondrial isoform X2 [Vitis vinifera]	110.79	38.967	13.2	13	14	13	0.993	1.025	1.039	0.982	0.986	0.967	0.96	5.05E-02
cds.Camellia_sinensis.comp34490_c1_seq2_m.18307	At2g47010/F14M4.16 [Arabidopsis lyrata subsp.	56.641	68.133	19.4	9	19	9	0.958	0.978	0.955	1.042	1.035	1.053	1.083	9.03E-04
cds.Camellia_sinensis.comp34493_c0_seq21_m.38449	PREDICTED: probable cytosolic oligopeptidase A [Sesamum indicum]	28.832	1.2475	6.6	2	4	2	0.904	1.018	1.097	0.981	0.996	1.022	0.993	9.53E-01

cds.Camellia_sinensis.comp34493_c0_seq8_m.11683	PREDICTED: probable cytosolic oligopeptidase A [Theobroma cacao]	18.812	2.1577	9.2	1	1	1	0.802	0.915	1.052	1.089	1.149	1.047	1.186	1.00E-01
cds.Camellia_sinensis.comp34499_c1_seq2_m.5013	PREDICTED: pantothenate kinase 2 [Prunus mume]	101.98	32.834	10.3	9	12	9	0.943	0.944	0.944	0.977	1.032	1.022	1.071	5.64E-02
cds.Camellia_sinensis.comp34500_c0_seq1_m.1120	PREDICTED: prolyl endopeptidase-like [Sesamum indicum]	80.844	52.152	20.2	12	15	12	0.978	0.956	0.97	1.04	1.06	1.041	1.082	9.95E-04
cds.Camellia_sinensis.comp34502_c0_seq2_m.29373	PREDICTED: alcohol dehydrogenase-like 7 [Eucalyptus grandis]	39.085	6.3619	9.2	3	4	3	0.983	0.959	0.859	1.038	1.115	1.07	1.151	3.78E-02
cds.Camellia_sinensis.comp34502_c1_seq5_m.34199	PREDICTED: peptidyl-prolyl cis-trans isomerase FKBP16-3, chloroplastic-like [Camelina sativa]	13.437	11.072	22.2	4	12	4	0.975	0.975	0.988	1.04	1.026	1.02	1.05	2.48E-03

cds.Camellia_sinensis.comp34506_c0_seq19_m.24942	PREDICTED: agmatine deiminase [Ziziphus jujuba]	42.533	16.916	18.4	8	9	8	0.993	1.007	1.024	1.024	0.982	1.023	1.002	9.30E-01
cds.Camellia_sinensis.comp34507_c0_seq23_m.34462	PREDICTED: ribonuclease J-like isoform X2 [Arachis duranensis]	30.201	1.8403	6.2	2	2	1	1.031	1.105	1.105	0.953	0.839	0.955	0.848	2.67E-02
cds.Camellia_sinensis.comp34508_c2_seq1_m.27588	PREDICTED: protein STRICTOSIDINE SYNTHASE-LIKE 5-like [Fragaria vesca subsp. vesca]	46.318	26.613	16.3	7	14	7	0.961	0.971	0.975	1.035	1.072	1.029	1.079	4.96E-03
cds.Camellia_sinensis.comp34513_c0_seq1_m.8059	PREDICTED: conserved oligomeric Golgi complex subunit 3 isoform X1 [Nelumbo nucifera]	66.093	4.7409	6.5	4	4	4	1.034	1.014	1.005	0.983	1.007	1.018	0.985	3.26E-01

cds.Camellia_sinensis.comp34513_c1_seq1_m.16451	PREDICTED: beta-(1,2)-xylosyltransferase [Nicotiana attenuata]	57.07	23.518	8.1	3	3	3	0.919	0.999	1.046	1.007	1.014	0.975	1.011	7.78E-01
cds.Camellia_sinensis.comp34514_c0_seq9_m.24763	PREDICTED: dihydroorotase, mitochondrial isoform X2 [Vitis	26.318	2.1729	7.6	2	2	2	1.11	0.966	0.916	1.079	0.883	1.045	1.005	9.60E-01
cds.Camellia_sinensis.comp34516_c0_seq2_m.44947	PREDICTED: glutamate synthase 1 [NADH], chloroplastic isoform X2 [Nicotiana attenuata]	27.441	10.571	8.4	2	5	2	0.973	0.941	0.963	1.086	1.112	1.034	1.123	7.94E-03
cds.Camellia_sinensis.comp34516_c1_seq1_m.14271	PREDICTED: glutamate synthase 1 [NADH], chloroplastic isoform X1 [Vitis vinifera]	66.623	21.001	11.4	6	7	4	1.034	1.033	1.016	0.992	0.99	0.91	0.938	8.94E-02

cds.Camellia_sinensis.comp34516_c1_seq2_m.46745	PREDICTED: glutamate synthase [NADH], amyloplastic-like, partial [Nicotiana tabacum]	25.891	24.9	26.8	5	6	3	0.993	0.929	1.024	0.98	1.016	1.071	1.041	3.54E-01
cds.Camellia_sinensis.comp34518_c1_seq1_m.25912	PREDICTED: novel plant SNARE 13 [Theobrom	29.468	4.8579	14.2	4	4	4	0.921	1.01	0.993	0.912	1.011	0.97	0.989	8.07E-01
cds.Camellia_sinensis.comp34525_c2_seq1_m.3453	PREDICTED: protein EXPORTIN 1A [Vitis vinifera]	92.645	17.826	13.7	10	14	10	0.983	0.918	0.938	1.038	1.065	1.075	1.119	7.64E-03
cds.Camellia_sinensis.comp34525_c2_seq1_m.3454	PREDICTED: exportin-1-like isoform X3 [Nicotiana sylvestris]	29.726	36.271	21.2	6	9	6	0.968	0.937	0.974	1.074	1.066	1.061	1.112	1.06E-03
cds.Camellia_sinensis.comp34526_c0_seq2_m.10529	PREDICTED: luminal-binding protein 5 [Theobroma cacao]	73.573	240.54	44	30	69	23	1.135	1.17	1.147	0.844	0.839	0.841	0.731	2.10E-06

cds.Camellia_sinensis.comp34526_c1_seq1_m.8583	PREDICTED: glycerophosphodiester phosphodiesterase GDPDL3-like [Juglans regia]	86.642	67.282	10.1	6	7	6	0.946	0.921	0.922	1.066	1.094	1.056	1.153	4.83E-04
cds.Camellia_sinensis.comp34526_c2_seq2_m.42838	PREDICTED: RNA-binding protein 8A-B [Nelumbo nucifera]	22.988	11.346	22.1	4	4	4	0.977	1.087	1.041	0.928	0.912	0.958	0.901	3.90E-02
cds.Camellia_sinensis.comp34532_c1_seq1_m.1859	Mitogen-activated protein kinase kinase kinase 13-A [Morus notabilis]	59.684	6.4866	3.1	2	2	1	0.962	0.972	1.055	1.006	1.015	1.005	1.012	6.98E-01
cds.Camellia_sinensis.comp34532_c1_seq3_m.3564	Mitogen-activated protein kinase kinase kinase 13-A [Morus notabilis]	123.02	10.571	3.2	4	4	2	0.911	1.011	1.094	0.876	1.157	0.967	0.995	9.29E-01

cds.Camellia_sinensis.comp34533_c0_seq11_m.1139	PREDICTED: glutamate synthase 1 [NADH], chloroplastic isoform X1 [Gossypium raimondii]	135.57	2.3808	11.2	12	34	0								
cds.Camellia_sinensis.comp34533_c0_seq12_m.1258	PREDICTED: ferredoxin-dependent glutamate synthase, chloroplastic [Vitis vinifera]	172.94	323.31	38.5	52	154	43	0.997	0.986	0.994	1.019	1.02	1.006	1.023	1.52E-02
cds.Camellia_sinensis.comp34538_c0_seq17_m.16360	PREDICTED: uncharacterized hydrolase YNR064C-like isoform X2 [Vigna angularis]	12.823	5.5762	25.4	2	2	2	0.962	1.064	1.054	1.022	0.935	0.973	0.951	2.95E-01

cds.Camellia_sinensis.comp34541_c0_seq1_m.9840	PREDICTED: ATP-dependent zinc metalloprotease FTSH, chloroplastic [Jatropha curcas]	76.797	323.31	51.9	30	101	24	0.974	0.984	0.985	1.006	1.032	1.023	1.04	9.10E-03
cds.Camellia_sinensis.comp34544_c0_seq2_m.18151	PREDICTED: alpha-1,3-mannosyl-glycoprotein 2-beta-N-acetylglucosaminyltransferase isoform X1 [Vitis vinifera]	38.735	7.7274	12.7	4	4	4								
cds.Camellia_sinensis.comp34545_c0_seq1_m.1794	PREDICTED: probable inactive ATP-dependent zinc metalloprotease FTSHI 5, chloroplastic [Vitis vinifera]	152.43	1.2732	0.5	1	1	1	1.164	1.155	1.165	0.796	0.886	0.79	0.71	1.14E-02

cds.Camellia_sinensis.comp34548_c0_seq1_m.8240	PREDICTED: programmed cell death protein 4 [Vitis vinifera]	79.412	37.641	18.4	11	18	8	0.971	1.012	1.007	0.998	1.006	1.002	1.005	6.99E-01
cds.Camellia_sinensis.comp34548_c0_seq2_m.34446	PREDICTED: programmed cell death protein 4 [Vitis vinifera]	28.431	9.0893	16.9	4	5	1	0.979	1.042	0.975	1.035	0.929	1.052	1.007	9.06E-01
cds.Camellia_sinensis.comp34549_c1_seq1_m.3197	PREDICTED: protein CLP1 homolog [Sesamum indicum]	46.684	8.7579	8.2	3	3	3	0.932	1.044	0.976	1.049	0.909	1.105	1.038	6.33E-01
cds.Camellia_sinensis.comp34553_c0_seq10_m.35414	PREDICTED: glutathionyl-hydroquinone reductase YqjG-like [Nicotiana tabacum]	31.986	7.0224	7.4	2	2	2	1.017	0.977	0.979	1.08	0.973	0.988	1.023	5.69E-01
cds.Camellia_sinensis.comp34553_c0_seq11_m.37196	PREDICTED: protein VAC14 homolog [Sesamum indicum]	25.829	1.9708	4.3	1	1	1								

cds.Camellia_sinensis.comp34553_c0_seq19_m.46997	PREDICTED: protein VAC14 homolog [Ricinus communis]	24.907	1.2425	4.9	1	1	1								
cds.Camellia_sinensis.comp34553_c0_seq4_m.21377	PREDICTED: protein VAC14 homolog [Jatropha curcas]	57.909	10.571	8.8	4	5	4	0.948	0.931	0.96	1.075	1.05	1.065	1.124	4.83E-04
cds.Camellia_sinensis.comp34553_c0_seq5_m.24176	PREDICTED: dynamin-related protein 1C [Juglans regia]	51.436	12.331	13.5	5	7	2	0.974	0.945	0.991	1.009	1.041	1.005	1.05	5.21E-02
cds.Camellia_sinensis.comp34557_c0_seq17_m.14225	PREDICTED: ABC transporter C family member 2 [Vitis vinifera]	81.457	96.453	22.1	18	26	18	0.995	1.001	1.02	0.998	0.982	0.971	0.978	1.17E-01
cds.Camellia_sinensis.comp34560_c1_seq1_m.10301	PREDICTED: wall-associated receptor kinase-like 1 [Vitis vinifera]	81.16	8.2556	4.4	3	3	3	1.09	0.948	1.142	0.848	0.936	1.018	0.881	1.76E-01

cds.Camellia_sinensis.comp34561_c0_seq51_m.12030	PREDICTED: LOW QUALITY PROTEIN: protein furry homolog-like [Sesamum indicum]	89.56	1.1866	1.3	1	1	1	0.869	0.976	1.037	0.975	1.091	1.081	1.092	2.30E-01
cds.Camellia_sinensis.comp34564_c0_seq5_m.14216	PREDICTED: dynamin-related protein 4C-like [Erythranthe guttata]	77.371	20.686	11.9	8	10	8	1.022	1.053	1.018	0.981	0.975	0.965	0.944	8.16E-03
cds.Camellia_sinensis.comp34568_c0_seq4_m.3640	PREDICTED: ubiquitin-activating enzyme E1 1 [Vitis vinifera]	121.65	154.31	28.3	26	38	20	0.975	0.982	1	1.013	1.006	0.998	1.02	8.13E-02
cds.Camellia_sinensis.comp34568_c0_seq7_m.14820	PREDICTED: glucose-6-phosphate isomerase, cytosolic [Sesamum indicum]	62.775	99.277	25.2	15	35	15	1.001	0.964	0.958	1.024	1.038	1.034	1.059	1.57E-02
cds.Camellia_sinensis.comp34570_c0_seq2_m.2150	PREDICTED: transportin-1 isoform X1 [Vitis vinifera]	100.93	52.604	12.9	10	11	10	0.934	0.971	1.012	1.032	1.001	1.074	1.065	1.10E-01

cds.Camellia_sinensis.comp34575_c0_seq1_m.23342	PREDICTED: bifunctional aspartate aminotransferase and glutamate/aspartate-prephenate aminotransferase isoform X2 [Vitis	52.631	69.467	23.8	9	11	9	1.061	1.113	1.066	0.881	0.876	0.956	0.837	5.14E-03
cds.Camellia_sinensis.comp34575_c1_seq35_m.27645	PREDICTED: ranBP2-type zinc finger protein At1g67325 isoform X1 [Vitis vinifera]	30.904	3.5299	6.4	1	2	1								
cds.Camellia_sinensis.comp34576_c0_seq2_m.20834	PREDICTED: ATP-dependent 6-phosphofructokinase 2 [Theobrom	56.344	4.6632	8.2	4	5	3	1.021	1.055	0.99	0.971	0.993	0.971	0.957	9.38E-02
cds.Camellia_sinensis.comp34580_c0_seq5_m.8252	PREDICTED: protein fluG [Vitis vinifera]	94.155	101.14	18.5	12	16	12	0.928	0.955	0.98	1.026	1.041	1.056	1.091	8.10E-03

cds.Camellia_sinensis.comp34583_c0_seq1_m.9794	PREDICTED: glutamine--fructose-6-phosphate aminotransferase [isomerizing] 2 [Vitis vinifera]	74.672	17.668	7.9	5	5	5	0.936	0.931	0.864	1.076	1.086	1.113	1.199	2.78E-03
cds.Camellia_sinensis.comp34585_c0_seq8_m.15571	PREDICTED: CBL-interacting protein kinase 04 isoform X1 [Vitis vinifera]	41.985	2.53	3.2	1	2	1								
cds.Camellia_sinensis.comp34586_c0_seq4_m.32396	PREDICTED: endochitinase PR4-like [Nelumbo nucifera]	24.711	10.503	11.5	2	2	2	0.855	0.774	0.763	1.226	1.205	1.246	1.537	3.16E-04
cds.Camellia_sinensis.comp34588_c0_seq1_m.16583	PREDICTED: adenylosuccinate synthetase 2, chloroplastic-like [Nelumbo nucifera]	54.314	118.54	27.5	13	26	13	0.984	1.004	1.003	1.021	1.013	1.008	1.017	8.79E-02

cds.Camellia_sinensis.comp34590_c1_seq4_m.24841	PREDICTED: L-2-hydroxyglutarate dehydrogenase, mitochondrial isoform X1 [Vitis	46.031	2.037	4.7	2	2	2								
cds.Camellia_sinensis.comp34593_c0_seq13_m.30739	PREDICTED: arginine--tRNA ligase, cytoplasmic-like isoform X2 [Sesamum indicum]	38.734	49.981	47.5	15	21	15	0.976	0.963	0.964	1.034	1.056	1	1.064	1.94E-02
cds.Camellia_sinensis.comp34593_c1_seq8_m.38584	PREDICTED: arginine--tRNA ligase, cytoplasmic-like isoform X2 [Sesamum indicum]	28.805	73.985	40.5	9	12	9	0.974	0.99	1.004	1.039	1.035	0.991	1.033	1.42E-01
cds.Camellia_sinensis.comp34595_c0_seq15_m.9001	UDP-glycosyltransferase 73C2 [Morus notabilis]	56.321	83.053	20.5	11	26	11	0.993	0.998	1.001	0.998	1.036	0.992	1.011	4.65E-01

cds.Camellia_sinensis.comp34599_c0_seq1_m.633	PREDICTED: protein lingerer isoform X1 [Vitis vinifera]	93.084	4.1394	1.7	1	2	1								
cds.Camellia_sinensis.comp34604_c1_seq6_m.34115	PREDICTED: 50S ribosomal protein L1, chloroplastic [Sesamum indicum]	34.331	83.202	40.3	14	28	14	1.075	1.035	1.033	0.953	0.947	0.946	0.906	1.68E-03
cds.Camellia_sinensis.comp34606_c0_seq6_m.25994	PREDICTED: 2-alkenal reductase (NADP(+)-dependent)-like [Sesamum indicum]	38.633	64.812	37.3	13	34	12	0.948	0.936	0.933	1.06	1.072	1.053	1.131	6.35E-05
cds.Camellia_sinensis.comp34608_c0_seq8_m.29269	PREDICTED: replication factor C subunit 5 [Theobroma cacao]	39.34	8.6074	13.7	5	5	5	0.886	0.961	0.905	1.062	1.082	1.139	1.193	5.32E-03

cds.Camellia_sinensis.comp34614_c0_seq18_m.43313	putative ADP-ribosylation factor GTPase-activating protein AGD5 [Morus notabilis]	29.563	1.8704	5.2	2	2	2	1.079	1.029	0.957	0.95	0.953	1.024	0.955	3.46E-01
cds.Camellia_sinensis.comp34614_c0_seq20_m.44756	PREDICTED: probable ADP-ribosylation factor GTPase-activating protein AGD5 [Capsicum annuum]	21.264	1.9202	4.5	1	1	1								
cds.Camellia_sinensis.comp34619_c0_seq3_m.17747	PREDICTED: phosphomevalonate kinase isoform X1 [Vitis vinifera]	54.117	1.4173	2.8	1	1	1								
cds.Camellia_sinensis.comp34627_c0_seq4_m.31090	PREDICTED: clavaminate synthase-like protein At3g21360 [Vitis vinifera]	36.8	12.654	16.1	5	5	5	0.951	0.93	0.989	1.059	1.03	1.066	1.099	1.04E-02

cds.Camellia_sinensis.comp34633_c0_seq5_m.39298	PREDICTED: NADH--cytochrome b5 reductase 1 isoform X2 [Ricinus communis]	30.415	1.4866	5.9	2	2	1								
cds.Camellia_sinensis.comp34641_c0_seq5_m.25316	PREDICTED: leucine--tRNA ligase, cytoplasmic-like [Nelumbo nucifera]	50.012	20.908	31.8	12	15	7	0.965	1.02	0.93	1.051	1.029	1.032	1.068	7.52E-02
cds.Camellia_sinensis.comp34641_c0_seq9_m.35789	PREDICTED: leucine--tRNA ligase, cytoplasmic-like [Nelumbo nucifera]	41.856	20.258	23.3	7	14	7	0.968	0.964	0.969	1.062	1.016	1.037	1.074	3.14E-02
cds.Camellia_sinensis.comp34643_c0_seq2_m.5535	PREDICTED: formin-like protein 3 isoform X1 [Fragaria vesca subsp.	57.687	3.8452	2.8	1	1	1								

cds.Camellia_sinensis.comp34643_c0_seq3_m.10744	PREDICTED: beta-galactosidase 9 isoform X1 [Citrus sinensis]	81.319	23.389	12.3	9	17	9	0.994	0.98	0.99	1.007	1	0.981	1.008	4.18E-01
cds.Camellia_sinensis.comp34643_c0_seq7_m.44936	PREDICTED: beta-galactosidase 9 [Ziziphus jujuba]	18.296	12.838	25.2	4	8	4	0.938	0.988	1.023	1.048	1.041	1.01	1.051	1.44E-01
cds.Camellia_sinensis.comp34645_c2_seq10_m.12522	PREDICTED: TBC1 domain family member 17 isoform X3 [Vitis vinifera]	80.592	18.723	12.3	7	8	7	0.951	0.963	0.999	1.057	0.994	1.008	1.05	1.09E-01
cds.Camellia_sinensis.comp34645_c2_seq6_m.4156	PREDICTED: probable sucrose-phosphate synthase isoform X3 [Nicotiana tabacum]	118.13	15.906	9.5	11	13	7	1.089	1.163	1.099	0.87	0.874	0.877	0.782	7.04E-03
cds.Camellia_sinensis.comp34645_c2_seq7_m.4543	PREDICTED: probable sucrose-phosphate synthase 3 [Prunus mume]	119.66	40.225	12.3	15	18	11	1.092	1.022	1.092	0.883	0.926	0.937	0.857	5.64E-03

cds.Camellia_sinensis.comp34650_c0_seq1_m.31180	PREDICTED: probable S-adenosylmethionine-dependent methyltransferase At5g37990 [Vitis vinifera]	40.715	11.405	15.5	6	8	6	0.977	0.942	1.042	0.987	1.03	1.021	1.026	4.59E-01
cds.Camellia_sinensis.comp34652_c0_seq1_m.14108	PREDICTED: patatin-like phospholipase domain-containing protein 2 isoform X1 [Nicotiana tomentosiformis]	47.37	1.883	2.1	1	1	1								
cds.Camellia_sinensis.comp34662_c1_seq5_m.34836	PREDICTED: nucleoid-associated protein At2g24020, chloroplastic-like [Nelumbo nucifera]	21.74	75.859	55.2	9	23	9	0.972	0.991	0.983	1.041	1.01	1.031	1.046	1.29E-02

cds.Camellia_sinensis.comp34663_c0_seq10_m.34729	PREDICTED: 50S ribosomal protein L15, chloroplastic [Vitis vinifera]	29.408	47.897	29.7	7	10	7	1.179	1.227	1.204	0.766	0.789	0.787	0.649	4.20E-06
cds.Camellia_sinensis.comp34663_c0_seq4_m.5288	ATP-NAD kinase family protein [Populus trichocarpa]	104.87	7.2113	4.9	4	5	4	0.957	0.889	0.861	1.09	1.075	1.159	1.228	6.14E-03
cds.Camellia_sinensis.comp34664_c1_seq3_m.34598	PREDICTED: peroxisomal (S)-2-hydroxy-acid oxidase GLO4 [Solanum lycopersicum]	37.428	27.271	19.2	6	9	6	0.956	1.015	1.013	1.004	1.002	1.045	1.022	4.02E-01
cds.Camellia_sinensis.comp34665_c2_seq9_m.7129	PREDICTED: ABC transporter C family member 2 [Theobroma cacao]	102.12	78.415	10.2	9	15	9	1.01	0.996	1.041	0.964	0.962	0.995	0.959	6.82E-02

cds.Camellia_sinensis.comp34674_c0_seq1_m.19224	PREDICTED: phosphoribosylamine--glycine ligase [Daucus carota subsp. sativus]	55.786	100.69	34.3	13	13	13	1.012	0.947	1.055	1.035	0.961	1.039	1.007	8.66E-01
cds.Camellia_sinensis.comp34674_c1_seq1_m.45225	PREDICTED: protein CutA, chloroplastic [Citrus sinensis]	20.119	31.688	24.1	5	9	5	0.989	0.88	0.912	1.09	1.118	1.046	1.17	1.63E-02
cds.Camellia_sinensis.comp34685_c0_seq1_m.17248	group 1 family glycosyltransferase [Medicago truncatula]	56.927	23.804	9.8	5	9	5	0.985	1.028	1.023	0.992	0.991	0.957	0.968	1.48E-01
cds.Camellia_sinensis.comp34691_c0_seq2_m.30056	PREDICTED: stromal 70 kDa heat shock-related protein, chloroplastic-like [Lupinus angustifolius]	37.758	124.04	44.3	16	45	9	0.94	0.925	0.905	1.084	1.093	1.082	1.177	1.41E-04

cds.Camellia_sinensis.comp34695_c0_seq5_m.19447	PREDICTED: DEAD-box ATP-dependent RNA helicase 35 [Gossypium raimondii]	60.629	1.3572	1.3	1	1	1	1.025	1.054	0.976	1.003	0.962	0.981	0.964	2.31E-01
cds.Camellia_sinensis.comp34697_c1_seq1_m.2737	PREDICTED: 5'-3' exoribonuclease 3 [Vitis vinifera]	126.6	2.0083	1.4	2	2	2	1.027	1.058	1.03	0.949	0.977	0.955	0.925	3.80E-03
cds.Camellia_sinensis.comp34698_c0_seq1_m.3023	PREDICTED: DNA-directed RNA polymerases IV and V subunit 2 [Solanum lycopersic	137.92	3.0848	1.6	2	2	2	1.063	1.027	1.069	0.946	0.919	0.966	0.896	4.58E-03
cds.Camellia_sinensis.comp34700_c0_seq1_m.18868	UDP-glycosyltransferase 71C3 [Morus notabilis]	56.597	37.281	12.1	6	15	6	1.086	1.021	1.053	0.909	0.923	0.983	0.891	1.78E-02

cds.Camellia_sinensis.comp34706_c0_seq9_m.31711	PREDICTED: glutamyl-tRNA reductase-binding protein, chloroplastic isoform X1 [Nelumbo nucifera]	37.928	15.911	15.3	5	6	5	1.034	1.126	1.07	0.932	0.936	0.858	0.844	1.07E-02
cds.Camellia_sinensis.comp34712_c0_seq4_m.22172	PREDICTED: importin subunit beta-1 [Vitis vinifera]	57.069	8.9628	16.7	6	9	3	1.038	1.042	0.984	0.94	0.929	0.952	0.921	1.41E-02
cds.Camellia_sinensis.comp34717_c0_seq1_m.15886	PREDICTED: probable splicing factor 3A subunit 1 [Beta vulgaris subsp. vulgaris]	44.567	23.655	6.4	2	2	2								
cds.Camellia_sinensis.comp34717_c0_seq1_m.15888	PREDICTED: probable splicing factor 3A subunit 1 [Vitis vinifera]	23.346	46.449	47.6	8	11	8	1.018	0.975	1.021	1.01	0.972	1.031	1	9.87E-01

cds.Camellia_sinensis.comp34717_c1_seq3_m.37342	PREDICTED: probable splicing factor 3A subunit 1 [Vitis vinifera]	25.445	14.626	10.5	2	2	2	0.962	0.923	0.99	1.059	0.996	1.094	1.095	5.69E-02
cds.Camellia_sinensis.comp34722_c0_seq1_m.7470	PREDICTED: alpha-L-fucosidase 2-like isoform X1 [Populus euphratica]	45.366	9.3131	6.2	2	2	2	0.986	0.979	0.935	0.921	1.072	1.116	1.072	3.35E-01
cds.Camellia_sinensis.comp34722_c0_seq2_m.21225	PREDICTED: alpha-L-fucosidase 2-like isoform X2 [Juglans regia]	46.676	14.981	6.4	2	3	2	0.983	1.002	0.917	1.039	1.07	1.008	1.074	8.78E-02
cds.Camellia_sinensis.comp34728_c0_seq1_m.3101	PREDICTED: serine/threonine-protein phosphatase BSL3 [Gossypium arboreum]	106.39	26.306	8.7	8	9	8	0.97	0.99	0.989	0.999	0.987	1.032	1.023	1.97E-01

cds.Camellia_sinensis.comp34735_c0_seq10_m.10269	PREDICTED: DNA gyrase subunit B, chloroplastic/mitochondrial [Vitis vinifera]	84.962	2.7479	7.1	4	5	2	0.968	1.069	1.054	0.957	0.954	0.996	0.94	1.49E-01
cds.Camellia_sinensis.comp34735_c0_seq6_m.9683	PREDICTED: DNA gyrase subunit B, chloroplastic/mitochondrial [Nicotiana tabacum]	69.374	15.312	9.7	5	5	3								
cds.Camellia_sinensis.comp34739_c0_seq2_m.24439	PREDICTED: ribosome-binding ATPase YchF [Ricinus communis]	46.296	48.637	31.3	11	17	11	1.033	1.07	1.037	0.953	0.969	0.945	0.913	2.44E-03
cds.Camellia_sinensis.comp34739_c0_seq4_m.27322	PREDICTED: transcription factor Pur-alpha 1 [Vitis vinifera]	32.266	48.111	40.4	12	17	12	0.983	0.976	0.979	1.045	0.99	1.009	1.036	1.58E-01
cds.Camellia_sinensis.comp34743_c1_seq4_m.16761	PREDICTED: chaperone protein dnaJ 13 [Vitis vinifera]	60.258	11.678	5.4	3	3	3	1.005	1.015	1.022	1.042	0.922	1.002	0.975	5.41E-01

cds.Camellia_sinensis.comp34744_c0_seq7_m.6769	PREDICTED: probable alanine--tRNA ligase, chloroplastic isoform X2 [Sesamum indicum]	43.522	70.825	31.2	10	14	8	1.051	0.983	1.028	0.968	0.953	0.987	0.95	8.13E-02
cds.Camellia_sinensis.comp34745_c0_seq3_m.30675	PREDICTED: 3-methyl-2-oxobutanoate hydroxymethyltransferase 1, mitochondrial [Juglans regia]	38.47	12.005	7.9	3	4	3	1.094	1.034	1.153	1.014	0.862	0.831	0.825	4.70E-02
cds.Camellia_sinensis.comp34747_c0_seq1_m.25473	PREDICTED: protein ECERIFERUM 1 [Theobroma cacao]	38.368	1.8741	4.7	2	2	2								
cds.Camellia_sinensis.comp34748_c1_seq9_m.32783	PREDICTED: tetratricopeptide repeat protein 1 [Theobroma cacao]	31.663	10.31	13.1	3	3	3	1.073	1.045	0.987	1.052	0.892	0.948	0.931	2.50E-01

cds.Camellia_sinensis.comp34751_c1_seq2_m.18448	PREDICTED: trafficking protein particle complex II-specific subunit 120 homolog isoform X1 [Vitis vinifera]	49.679	3.8305	5	2	3	2								
cds.Camellia_sinensis.comp34751_c2_seq1_m.4380	PREDICTED: probable polyribonucleotide nucleotidyltransferase 1, chloroplastic [Vitis vinifera]	69.87	29.843	12.9	7	8	7	1.138	1.204	1.142	0.772	0.88	0.9	0.732	3.62E-03
cds.Camellia_sinensis.comp34751_c2_seq1_m.4381	PREDICTED: polyribonucleotide nucleotidyltransferase 1, chloroplastic-like [Nicotiana tabacum]	33.056	2.9873	9.3	3	3	3	1.075	1.083	1.152	0.889	0.875	0.904	0.806	8.39E-04

cds.Camellia_sinensis.comp34753_c0_seq2_m.17644	PREDICTED: nuclear pore complex protein NUP98A [Vitis vinifera]	69.604	7.6347	8.6	6	6	5	0.941	1.025	1.005	1.06	0.991	0.998	1.026	4.80E-01
cds.Camellia_sinensis.comp34756_c1_seq2_m.1669	PREDICTED: sister chromatid cohesion protein PDS5 homolog A isoform X2 [Vitis vinifera]	95.499	1.421	1.2	1	1	1	0.977	0.948	0.938	1.141	1.014	1.009	1.105	7.89E-02
cds.Camellia_sinensis.comp34763_c0_seq1_m.14738	glutamate--cysteine ligase, chloroplastic [Nicotiana tabacum]	59.457	98.571	26.4	13	26	13	0.996	1.004	0.973	0.986	1.005	0.995	1.004	7.06E-01
cds.Camellia_sinensis.comp34765_c0_seq33_m.14153	PREDICTED: thioredoxin reductase NTRC [Ipomoea	36.436	52.225	30	8	16	8	0.98	0.978	1.026	1.019	0.983	1.014	1.011	6.06E-01

cds.Camellia_sinensis.comp34765_c0_seq72_m.31574	PREDICTED: U2 small nuclear ribonucleoprotein A' [Ipomoea nil]	33.296	56.787	29.8	7	9	7	1.143	1.172	1.146	0.895	0.872	0.816	0.746	5.16E-04
cds.Camellia_sinensis.comp34765_c0_seq9_m.9901	PREDICTED: thioredoxin reductase NTRC isoform X1 [Theobrom	25.838	52.381	23.8	4	7	4	1.029	0.973	1.009	1.033	1.022	1	1.015	4.82E-01
cds.Camellia_sinensis.comp34770_c0_seq1_m.4049	PREDICTED: exocyst complex component SEC5A [Vitis vinifera]	123.42	6.4798	1.5	1	1	1	1.005	1.129	1.021	1.008	0.919	0.918	0.902	9.75E-02
cds.Camellia_sinensis.comp34770_c1_seq1_m.5193	PREDICTED: L-ascorbate oxidase homolog [Juglans regia]	59.889	18.432	7	4	4	3	1.009	1.057	1.038	0.942	0.943	1.009	0.932	5.63E-02

cds.Camellia_sinensis.comp34780_c0_seq2_m.3092	PREDICTED: ubiquitin carboxyl-terminal hydrolase 12 isoform X1 [Vitis vinifera]	128.19	51.784	13.4	18	22	12	0.975	1.016	1.01	0.988	0.995	1.023	1.002	9.23E-01
cds.Camellia_sinensis.comp34783_c0_seq6_m.39458	PREDICTED: transmembrane emp24 domain-containing protein p24beta2-like [Prunus mume]	24.746	17.025	14.5	3	5	3	0.933	0.997	1.006	1.006	1.037	1.065	1.059	1.19E-01
cds.Camellia_sinensis.comp34786_c0_seq1_m.1054	PREDICTED: brefeldin A-inhibited guanine nucleotide-exchange protein 2 [Vitis vinifera]	135.25	7.9774	1.6	2	2	0								

cds.Camellia_sinensis.comp34786_c0_seq1_m.1055	PREDICTED: brefeldin A-inhibited guanine nucleotide-exchange protein 2 [Citrus sinensis]	69.982	1.6028	3.8	3	3	1								
cds.Camellia_sinensis.comp34786_c0_seq3_m.6334	PREDICTED: brefeldin A-inhibited guanine nucleotide-exchange protein 2-like [Jatropha curcas]	111.33	8.0893	5	5	5	2								
cds.Camellia_sinensis.comp34788_c0_seq15_m.2755	DUF1350 family protein [Medicago truncatula]	42.48	14.013	9.2	3	4	1	0.93	0.888	0.965	1.082	1.086	1.084	1.169	2.29E-02

cds.Camellia_sinensis.comp34789_c1_seq1_m.8540	PREDICTED: uncharacterized aarF domain-containing protein kinase At1g79600, chloroplastic [Sesamum indicum]	59.937	7.2469	3.7	2	2	2	0.98	0.886	1.009	1.122	1.032	0.999	1.097	1.52E-01
cds.Camellia_sinensis.comp34789_c1_seq2_m.9243	PREDICTED: uncharacterized aarF domain-containing protein kinase At1g79600, chloroplastic isoform X1[Gossypium raimondii]	21.324	2.93	6.6	1	1	1	0.658	1.549	0.858	0.961	1.044	0.96	0.967	9.11E-01
cds.Camellia_sinensis.comp34793_c0_seq4_m.2146	PREDICTED: tripeptidyl-peptidase 2 isoform X1 [Vitis vinifera]	109.19	130.06	34.9	30	49	30	1.029	1.023	1.039	0.989	0.977	0.985	0.955	1.30E-03

cds.Camellia_sinensis.comp34793_c0_seq4_m.2147	PREDICTED: tripeptidyl-peptidase 2 [Nelumbo nucifera]	15.278	5.4707	17.3	2	3	2	1.121	0.917	1.036	0.97	1.041	0.911	0.951	5.24E-01
cds.Camellia_sinensis.comp34798_c0_seq1_m.436	PREDICTED: serine/threonine-protein kinase TOR [Theobroma cacao]	277.53	14.952	3	7	7	7	0.804	1.867	0.819	0.714	0.785	0.977	0.709	4.09E-01
cds.Camellia_sinensis.comp34802_c1_seq1_m.780	PREDICTED: chromatin structure-remodeling complex protein SYD isoform X1 [Ziziphus jujuba]	88.729	3.0965	3.4	3	3	3	1.074	1.42	1.076	0.746	0.925	0.711	0.667	3.08E-02

cds.Camellia_sinensis.comp34805_c0_seq5_m.5158	PREDICTED: calcium-transporting ATPase 3, endoplasmic reticulum-type isoform X4 [Vitis vinifera]	60.775	1.4735	2.1	1	1	1	0.896	1.019	1.125	1.032	0.935	1.014	0.981	8.34E-01
cds.Camellia_sinensis.comp34807_c0_seq18_m.28811	PREDICTED: adenylate cyclase, terminal-differentiation specific [Vitis vinifera]	40.865	2.0589	3.3	1	1	1								
cds.Camellia_sinensis.comp34809_c0_seq1_m.4649	PREDICTED: exosome complex exonuclease RRP44 homolog A [Ziziphus	98.005	4.6841	2.6	2	2	2	0.945	0.924	0.943	1.064	1.083	1.071	1.144	1.04E-04
cds.Camellia_sinensis.comp34815_c1_seq34_m.13298	PREDICTED: zinc finger protein ZPR1 [Vitis vinifera]	55.299	8.5994	3.2	1	1	1	0.84	1.038	1.022	0.891	1.07	1.161	1.077	5.24E-01

cds.Camellia_sinensis.comp34815_c1_seq62_m.37747	PREDICTED: bifunctional aspartokinase/homoserine dehydrogenase isoform X5 [Solanum tuberosum	33.059	5.768	6.5	2	2	2	0.891	0.969	1.01	1.081	1.024	1.058	1.102	7.03E-02
cds.Camellia_sinensis.comp34817_c0_seq1_m.16288	PREDICTED: SEC1 family transport protein SLY1-like [Populus euphratica]	61.494	19.022	10.6	5	5	5	0.968	1.046	1.018	1.004	0.93	1.034	0.979	6.05E-01
cds.Camellia_sinensis.comp34819_c0_seq1_m.987	PREDICTED: clathrin heavy chain 1 [Vigna radiata var. radiata]	192.51	323.31	33.8	56	98	10	0.993	0.984	0.972	1.026	0.999	1.031	1.036	3.73E-02
cds.Camellia_sinensis.comp34819_c0_seq4_m.1484	PREDICTED: clathrin heavy chain 1-like [Nelumbo nucifera]	183.31	15.903	33.9	52	93	6	1.009	1.028	0.981	0.976	0.998	1.057	1.004	8.93E-01

cds.Camellia_sinensis.comp34821_c0_seq1_m.3529	PREDICTED: protease Do-like 7 isoform X1 [Vitis vinifera]	122.44	79.554	17	18	20	12	1.004	1.018	1.014	1.006	0.981	0.968	0.973	8.67E-02
cds.Camellia_sinensis.comp34821_c0_seq3_m.44578	PREDICTED: protease Do-like 7 isoform X3 [Lupinus angustifolius]	27.768	36.057	33.5	8	9	3	0.96	0.994	1.073	1.047	0.98	0.986	0.995	9.23E-01
cds.Camellia_sinensis.comp34822_c0_seq70_m.49511	PREDICTED: allantoinase isoform X2 [Daucus carota subsp. sativus]	16.693	2.6798	21.6	2	2	2								
cds.Camellia_sinensis.comp34824_c0_seq1_m.11780	PREDICTED: cellulose synthase-like protein G3 isoform X2 [Ziziphus jujuba]	48.382	1.5015	3.7	2	2	2	0.977	1.088	0.996	0.951	0.978	1.012	0.961	3.57E-01

cds.Camellia_sinensis.comp34826_c2_seq3_m.16973	PREDICTED: eukaryotic translation initiation factor 3 subunit E [Arachis ipaensis]	51.27	110.27	28.1	12	16	12	0.949	0.985	0.959	1.046	0.993	1.013	1.055	4.66E-02
cds.Camellia_sinensis.comp34838_c1_seq1_m.2286	PREDICTED: coatomer subunit alpha-1 [Ricinus communis]	136.7	124.24	17.8	22	32	22	1.119	1.106	1.094	0.91	0.89	0.879	0.807	5.94E-05
cds.Camellia_sinensis.comp34844_c0_seq1_m.2284	PREDICTED: ABC transporter B family member 11 [Vitis vinifera]	114.31	14.011	6.5	5	6	3	1.023	0.997	1.013	1.047	0.978	0.929	0.974	4.80E-01
cds.Camellia_sinensis.comp34851_c2_seq1_m.3514	PREDICTED: cell division cycle 5-like protein [Vitis vinifera]	113.21	2.3616	1.9	2	2	2								
cds.Camellia_sinensis.comp34851_c2_seq4_m.8374	PREDICTED: pre-mRNA-processing protein 40A [Nelumbo nucifera]	57.399	2.4999	4.5	2	2	2								

cds.Camellia_sinensis.comp34851_c2_seq4_m.8375	PREDICTED: pre-mRNA-processing protein 40A isoform X2 [Vitis vinifera]	44.437	4.0584	5.7	2	2	2	0.839	1.253	0.834	1.133	0.982	0.99	1.061	6.22E-01
cds.Camellia_sinensis.comp34867_c0_seq1_m.407	DUF2921 family protein [Medicago truncatula]	85.938	1.3491	1.1	1	1	1								
cds.Camellia_sinensis.comp34869_c0_seq1_m.249	PREDICTED: E3 ubiquitin-protein ligase UPL1 isoform X2 [Vitis vinifera]	386.46	17.637	2.8	10	10	7	0.977	1.134	1.069	0.946	0.917	0.933	0.879	4.56E-02
cds.Camellia_sinensis.comp34870_c0_seq1_m.6900	PREDICTED: subtilisin-like protease SBT1.5 [Vitis vinifera]	82.486	28.675	10	6	8	6	0.868	0.89	0.923	1.12	1.119	1.104	1.247	2.79E-04

cds.Camellia_sinensis.comp34876_c1_seq2_m.32424	PREDICTED: geranylgeranyl transferase type-2 subunit alpha 1 [Vitis vinifera]	32.118	8.5522	7.7	2	2	2	0.818	0.963	0.674	1.281	0.953	1.373	1.469	6.55E-02
cds.Camellia_sinensis.comp34880_c1_seq5_m.13829	PREDICTED: E3 ubiquitin-protein ligase UPL2 [Vitis vinifera]	81.209	3.3157	6.6	4	5	3	0.93	1.076	1.02	0.981	1.078	0.931	0.988	8.52E-01
cds.Camellia_sinensis.comp34896_c1_seq4_m.388	PREDICTED: probable phosphoribosylformylglycinamidine synthase, chloroplastic/mitochondrial [Vitis vinifera]	154.82	113.16	22.6	29	43	29	0.97	1.018	0.99	1.004	1.003	0.994	1.008	6.12E-01
cds.Camellia_sinensis.comp34896_c1_seq4_m.390	PREDICTED: topless-related protein 3-like [Sesamum indicum]	30.977	13.056	25.2	7	10	4	0.892	0.96	0.847	0.924	1.016	1.098	1.126	1.30E-01

cds.Camellia_sinensis.comp34897_c0_seq1_m.551	PREDICTED: DExH-box ATP-dependent RNA helicase DExH12-like [Nelumbo nucifera]	204.09	4.9096	0.9	2	2	2								
cds.Camellia_sinensis.comp34899_c0_seq2_m.2747	PREDICTED: beta-glucosidase 44-like [Citrus sinensis]	14.95	1.4665	5.6	1	2	1	0.999	0.985	0.991	0.997	1.058	0.981	1.021	4.44E-01
cds.Camellia_sinensis.comp34899_c1_seq1_m.31033	PREDICTED: probable ATP synthase 24 kDa subunit, mitochondrial [Vitis vinifera]	28.086	121.01	54.8	17	51	17	1.028	1.028	1.029	0.976	0.979	0.964	0.946	7.22E-03
cds.Camellia_sinensis.comp34899_c2_seq1_m.7036	PREDICTED: glutamine--tRNA ligase, cytoplasmic [Vitis vinifera]	90.976	54.287	23	18	28	18	0.999	0.974	0.984	1.015	0.965	1.041	1.022	4.22E-01

cds.Camellia_sinensis.comp34914_c0_seq1_m.885	PREDICTED: probable tRNA N6-adenosine threonylcarbamoyltransferase [Theobroma cacao]	38.531	14.1	14.5	4	5	4	0.887	0.993	1.063	1.062	1.003	0.96	1.028	6.52E-01
cds.Camellia_sinensis.comp34919_c0_seq1_m.38285	PREDICTED: early light-induced protein, chloroplastic [Arachis ipaensis]	24.048	31.945	19.2	4	6	4	0.91	0.941	0.964	1.001	1.06	1.114	1.128	2.71E-02
cds.Camellia_sinensis.comp34921_c0_seq1_m.57219	PREDICTED: chlorophyll a-b binding protein 151, chloroplastic [Ricinus communis]	12.857	43.093	12	2	19	2	0.939	0.937	0.937	1.068	1.065	1.056	1.134	1.75E-06
cds.Camellia_sinensis.comp34927_c0_seq1_m.40973	PREDICTED: plastocyanin B'/B" [Ricinus communis]	17.157	87.079	38.2	3	42	3	0.945	0.96	0.924	1.099	1.024	1.067	1.128	6.78E-03

cds.Camellia_sinensis.comp34929_c0_seq1_m.29202	PREDICTED: chlorophyll a-b binding protein CP24 10A, chloroplastic [Gossypium hirsutum]	27.483	50.377	23.6	5	20	5	0.921	0.888	0.888	1.052	1.163	1.122	1.237	2.60E-03
cds.Camellia_sinensis.comp34935_c0_seq1_m.42009	PREDICTED: ferredoxin [Ziziphus jujuba]	15.691	2.9641	6.8	1	3	1	1.181	1.077	1.01	0.821	0.84	1.031	0.824	8.23E-02
cds.Camellia_sinensis.comp34946_c0_seq1_m.36496	PREDICTED: photosystem I reaction center subunit XI, chloroplastic [Theobroma cacao]	22.982	84.35	17.5	5	13	5	0.869	0.963	0.929	1.103	1.088	1.113	1.197	4.24E-03
cds.Camellia_sinensis.comp34950_c0_seq1_m.37352	PREDICTED: glutathione S-transferase-like [Sesamum indicum]	23.86	65.342	56.6	9	36	9	0.99	0.996	0.937	1.056	1.01	1.026	1.058	7.35E-02

cds.Camellia_sinensis.comp34958_c0_seq1_m.22394	PREDICTED: carbonic anhydrase 2 isoform X1 [Vitis vinifera]	35.791	244.11	66.1	18	90	16	1.094	1.066	1.096	0.93	0.936	0.901	0.85	3.84E-04
cds.Camellia_sinensis.comp34971_c0_seq1_m.24340	PREDICTED: 60S ribosomal protein L3 [Vitis vinifera]	44.428	36.622	27.2	13	26	9	1.095	1.107	1.126	0.905	0.858	0.869	0.791	2.00E-04
cds.Camellia_sinensis.comp34975_c0_seq1_m.47652	thylakoid soluble phosphoprotein TSP9 protein [Medicago truncatula]	11.379	18.233	44.8	6	28	6	1.06	1.051	1.045	0.943	0.941	0.951	0.898	3.70E-05
cds.Camellia_sinensis.comp34977_c0_seq1_m.38399	PREDICTED: 60S ribosomal protein L18-2 [Eucalyptus grandis]	20.934	55.785	28.3	5	9	5	1.09	1.074	1.055	0.895	0.954	0.906	0.856	2.00E-03
cds.Camellia_sinensis.comp34979_c0_seq1_m.37351	plant/F25P12-18 protein [Medicago truncatula]	18.363	77.213	47.3	6	14	6	1.332	1.28	1.31	0.71	0.626	0.669	0.511	6.05E-05

cds.Camellia_sinensis.comp34981_c0_seq1_m.15901	PREDICTED: fructose-bisphosphate aldolase cytoplasmic isozyme [Sesamum	39.634	313.78	58.1	17	55	17	0.978	0.976	0.97	1.022	1.027	1.042	1.057	9.24E-04
cds.Camellia_sinensis.comp34991_c0_seq1_m.15071	PREDICTED: phosphoglycerate kinase, chloroplastic [Nicotiana sylvestris]	53.529	323.31	61.5	29	145	22	1.048	1.007	1.023	0.976	0.999	0.98	0.96	4.06E-02
cds.Camellia_sinensis.comp34996_c0_seq1_m.19097	flavonoid 3' hydroxylase [Vitis vinifera]	57.059	17.251	19.1	9	14	9	1.248	1.29	1.269	0.668	0.678	0.703	0.538	2.09E-06
cds.Camellia_sinensis.comp35001_c0_seq1_m.38543	PREDICTED: glyceraldehyde-3-phosphate dehydrogenase B, chloroplastic [Ziziphus jujuba]	32.204	64.996	21.5	7	29	7	1.031	1.002	1.004	0.988	0.997	0.995	0.981	1.21E-01

cds.Camellia_sinensis.comp35002_c0_seq1_m.43995	PREDICTED: 60S ribosomal protein L32-1-like [Cucumis melo]	15.662	11.261	26.3	4	8	4	1.107	1.185	1.187	0.802	0.822	0.831	0.706	1.60E-04
cds.Camellia_sinensis.comp35021_c0_seq1_m.26701	PREDICTED: 14-3-3-like protein D isoform X1 [Ricinus communis]	29.45	136.13	54.8	15	54	11	1.002	0.984	0.959	1.012	1.023	1.02	1.037	4.86E-02
cds.Camellia_sinensis.comp35026_c0_seq1_m.46440	photosystem II 5 kD family protein [Populus trichocarpa]	11.685	12.832	11.7	2	14	2	0.987	0.988	0.929	1.045	1.066	1.042	1.086	1.86E-02
cds.Camellia_sinensis.comp35031_c0_seq1_m.8910	PREDICTED: 5-methyltetrahydropteroyltriglutamate--homocysteine methyltransferase-like [Nicotiana tabacum]	84.844	187.45	35.6	27	57	20	1.032	1.039	1.078	0.952	0.935	0.957	0.903	2.64E-03

cds.Camellia_sinensis.comp35037_c0_seq1_m.45235	macrophage migration inhibition factor-like protein [Medicago	13.043	15.924	50	4	9	4	1.005	0.975	0.973	1.024	1.002	1.016	1.03	7.18E-02
cds.Camellia_sinensis.comp35043_c0_seq1_m.40382	PREDICTED: 50S ribosomal protein L12, chloroplastic [Solanum lycopersicum]	19.234	79.068	41.8	11	32	11	0.989	0.962	0.977	1.009	1.044	1.03	1.053	1.55E-02
cds.Camellia_sinensis.comp35044_c0_seq1_m.44426	PREDICTED: cytochrome b5 [Vitis vinifera]	17.783	29.766	39.6	4	8	4	1.317	1.204	1.242	0.773	0.657	0.768	0.584	8.04E-04
cds.Camellia_sinensis.comp35045_c0_seq1_m.29822	CS domain protein [Medicago truncatula]	23.006	46.815	32.7	8	19	8	1.184	1.134	1.152	0.794	0.808	0.858	0.709	2.03E-04
cds.Camellia_sinensis.comp35046_c0_seq1_m.27092	PREDICTED: glutamine synthetase nodule isozyme [Ziziphus jujuba]	39.204	24.323	36.2	12	50	4	1.158	1.121	1.104	0.808	0.888	0.873	0.759	1.02E-03

cds.Camellia_sinensis.comp35051_c0_seq1_m.22526	PREDICTED: geranylgeranyl diphosphate reductase, chloroplastic [Vitis vinifera]	54.645	67.049	23	10	20	10	1.046	1.025	1.084	0.94	0.95	0.95	0.9	3.30E-03
cds.Camellia_sinensis.comp35057_c0_seq1_m.37888	PREDICTED: thioredoxin F1, chloroplastic-like [Sesamum indicum]	19.7	17.969	19.3	4	12	4	1.037	1.019	1.05	0.983	0.981	0.932	0.932	2.26E-02
cds.Camellia_sinensis.comp35063_c0_seq1_m.37893	PREDICTED: peptidyl-prolyl cis-trans isomerase CYP19-4 [Malus domestica]	22.45	48.611	36.5	7	23	5	1.107	1.116	1.074	0.896	0.882	0.901	0.813	9.94E-05
cds.Camellia_sinensis.comp35073_c0_seq1_m.47663	PREDICTED: 40S ribosomal protein S25-2 [Citrus sinensis]	14.911	4.9295	19	3	3	3	1.013	1.142	1.126	0.9	0.923	0.883	0.825	8.70E-03

cds.Camellia_sinensis.comp35081_c0_seq1_m.58830	PREDICTED: elongation factor 1-alpha-like, partial [Gossypium arboreum]	11.563	3.3328	63.5	7	29	1	0.963	1.092	0.998	1.059	0.748	1.145	0.967	7.39E-01
cds.Camellia_sinensis.comp35106_c0_seq1_m.6667	PREDICTED: inositol-3-phosphate synthase [Nicotiana tomentosiformis]	56.19	186.79	29	16	49	16	0.901	0.884	0.874	1.103	1.142	1.123	1.267	6.03E-05
cds.Camellia_sinensis.comp35107_c0_seq1_m.44017	PREDICTED: 60S ribosomal protein L30-like [Juglans regia]	12.241	7.8302	16.1	2	5	2	1.018	1.038	1.011	0.963	0.994	0.931	0.942	4.20E-02
cds.Camellia_sinensis.comp35113_c0_seq1_m.25625	PREDICTED: porphobilinogen deaminase, chloroplastic [Vitis vinifera]	41.897	22.951	17.7	7	11	7	1.047	1.068	1.072	0.916	0.94	0.946	0.879	4.63E-04

cds.Camellia_sinensis.comp35127_c0_seq1_m.49698	PREDICTED: phosphoprotein ECPP44-like [Gossypium arboreum]	13.331	96.74	73.1	7	12	7	0.943	0.957	0.98	1.02	1.065	1.068	1.095	8.30E-03
cds.Camellia_sinensis.comp35145_c0_seq1_m.54819	PREDICTED: stromal 70 kDa heat shock-related protein, chloroplastic-like [Nelumbo nucifera]	15.126	6.7673	77	12	42	3	1.061	1.049	1.049	0.97	0.947	0.95	0.908	3.23E-04
cds.Camellia_sinensis.comp35153_c0_seq1_m.36410	PREDICTED: universal stress protein PHOS32-like isoform X2 [Juglans regia]	17.419	67.543	42.1	5	10	5	1	0.983	0.966	1.007	1.065	1.012	1.046	9.55E-02

cds.Camellia_sinensis.comp35156_c0_seq1_m.46856	PREDICTED: succinate dehydrogenase subunit 7B, mitochondrial-like isoform X1 [Jatropha	11.324	4.8037	9.9	1	4	1	0.996	0.916	1.028	0.985	1.029	1.021	1.032	4.25E-01
cds.Camellia_sinensis.comp35173_c0_seq1_m.9433	Elongation factor G [Morus notabilis]	90.514	323.31	43.5	29	78	29	1.01	1	0.981	1.003	1.027	0.997	1.012	3.91E-01
cds.Camellia_sinensis.comp35183_c0_seq1_m.41778	PREDICTED: nucleoside diphosphate kinase 1 [Daucus carota subsp. sativus]	16.414	70.709	33.8	6	15	6	0.941	0.943	0.98	1.051	1.036	1.051	1.096	2.86E-03
cds.Camellia_sinensis.comp35195_c0_seq1_m.31710	PREDICTED: 30S ribosomal protein S5, chloroplastic-like [Gossypium raimondii]	37.255	98.51	32.1	12	29	12	1.087	1.034	1.07	0.914	0.935	0.923	0.869	9.60E-04

cds.Camellia_sinensis.comp35198_c0_seq1_m.15306	PREDICTED: pyruvate kinase, cytosolic isozyme [Jatropha curcas]	55.114	177.63	40	18	34	18	0.99	0.994	0.988	0.981	0.999	1.013	1.007	5.03E-01
cds.Camellia_sinensis.comp35200_c0_seq1_m.30284	PREDICTED: GDSL esterase/lipase 1-like [Fragaria vesca subsp. vesca]	40.596	1.9736	5.2	2	2	2								
cds.Camellia_sinensis.comp35217_c0_seq1_m.27870	PREDICTED: leucoanthocyanidin dioxygenase [Theobroma cacao]	40.057	51.661	24.5	7	9	7	1.343	1.36	1.416	0.567	0.584	0.561	0.416	8.03E-07
cds.Camellia_sinensis.comp35229_c0_seq1_m.39687	PREDICTED: NADH dehydrogenase [ubiquinone] 1 alpha subcomplex subunit 13-B [Daucus carota subsp. sativus]	21.891	23.45	38	7	15	7	1.016	0.995	0.988	0.993	1.004	1.002	1	9.95E-01

cds.Camellia_sinensis.comp35231_c0_seq1_m.48564	PREDICTED: stress-response A/B barrel domain-containing protein HS1 [Eucalyptus grandis]	13.499	40.212	35	3	11	3	0.922	0.881	0.914	1.119	1.072	1.101	1.212	5.15E-04
cds.Camellia_sinensis.comp35236_c0_seq1_m.43555	PREDICTED: small ubiquitin-related modifier 1 [Pyrus x bretschneideri]	13.194	1.4869	30.4	4	8	0								
cds.Camellia_sinensis.comp35241_c0_seq1_m.57775	PREDICTED: ATP synthase subunit d, mitochondrial-like [Gossypium hirsutum]	12.944	55.631	68.8	9	24	7	0.998	0.996	1.018	1.005	1.028	0.971	0.997	8.80E-01
cds.Camellia_sinensis.comp35255_c0_seq1_m.31469	PREDICTED: alcohol dehydrogenase class-3 [Elaeis guineensis	42.624	74.052	22.9	9	31	9	0.954	0.955	0.967	1.04	1.066	1.038	1.093	7.57E-04

cds.Camellia_sinensis.comp35258_c0_seq1_m.47005	PREDICTED: SKP1-like protein 1A isoform X5 [Vitis vinifera]	11.688	52.826	59	8	9	4	0.981	0.927	0.943	1.083	1.063	1.028	1.113	9.06E-03
cds.Camellia_sinensis.comp35270_c0_seq1_m.24855	PREDICTED: glycerate dehydrogenase [Malus domestica]	43.829	144.18	59.2	21	64	21	0.984	0.974	0.978	1.02	1.018	1.005	1.036	2.90E-03
cds.Camellia_sinensis.comp35272_c0_seq1_m.43385	PREDICTED: small nuclear ribonucleoprotein SmD3b-like [Juglans regia]	18.612	30.504	37.5	5	11	5	0.97	0.993	0.991	1.013	1.006	1.019	1.028	2.84E-02
cds.Camellia_sinensis.comp35287_c0_seq1_m.32879	PREDICTED: 50S ribosomal protein L3, chloroplastic [Beta vulgaris subsp. vulgaris]	34.481	38.478	20	6	10	5	1.101	1.217	1.172	0.855	0.827	0.763	0.701	1.34E-03

cds.Camellia_sinensis.comp35291_c0_seq1_m.30274	PREDICTED: 28 kDa ribonucleoprotein, chloroplastic-like [Nelumbo nucifera]	35.919	168.49	30.1	10	38	8	0.997	1.033	1.023	0.987	0.98	0.992	0.969	4.91E-02
cds.Camellia_sinensis.comp35293_c0_seq1_m.28986	PREDICTED: 24-methylenesterol C-methyltransferase 2 [Prunus mume]	40.392	30.98	17.6	5	5	5	0.918	0.998	0.973	1.064	1.053	1.031	1.09	3.08E-02
cds.Camellia_sinensis.comp35310_c0_seq1_m.46730	PREDICTED: endoplasmin homolog [Vitis	22.179	7.0517	28	6	9	4	0.969	0.909	0.949	1.018	1.081	1.084	1.126	1.30E-02
cds.Camellia_sinensis.comp35321_c0_seq1_m.34345	PREDICTED: aquaporin SIP1-1-like [Ziziphus jujuba]	28.011	1.9884	3.4	1	2	1	1.058	0.986	1.021	0.96	0.985	0.987	0.957	1.19E-01
cds.Camellia_sinensis.comp35325_c0_seq1_m.38167	PREDICTED: desiccation protectant protein Lea14 homolog [Juglans regia]	16.566	24.062	43.8	6	13	6	0.929	0.933	0.963	1.017	1.072	1.139	1.143	1.87E-02

cds.Camellia_sinensis.comp35329_c0_seq1_m.34688	PREDICTED: 50S ribosomal protein L5, chloroplastic [Sesamum indicum]	28.944	43.108	34.1	8	14	8	1.076	1.195	1.139	0.834	0.879	0.83	0.746	1.18E-03
cds.Camellia_sinensis.comp35338_c0_seq1_m.58333	PREDICTED: heat shock protein 90-2, partial [Cucumis melo]	12.776	2.1117	34.3	4	10	2	1.158	1.072	1.072	0.874	0.884	0.907	0.807	1.58E-03
cds.Camellia_sinensis.comp35345_c0_seq1_m.29931	Thioredoxin family protein [Arabidopsis thaliana]	39.56	75.683	31.2	9	15	9	1.061	1.033	1.005	0.955	0.954	0.958	0.925	3.86E-02
cds.Camellia_sinensis.comp35351_c0_seq1_m.16248	PREDICTED: cytochrome P450 82C4-like [Nicotiana tabacum]	60.067	1.2892	1.7	1	1	1								
cds.Camellia_sinensis.comp35353_c0_seq1_m.47317	PREDICTED: calvin cycle protein CP12-3, chloroplastic [Theobroma cacao]	21.807	16.26	6.5	1	2	1	0.908	0.956	1.072	0.955	0.996	1.131	1.05	5.31E-01

cds.Camellia_sinensis.comp35355_c0_seq1_m.52503	PREDICTED: 60S ribosomal protein L14-2 [Ipomoea nil]	12.528	24.42	44.4	5	15	2	1.067	1.115	1.136	0.923	0.863	0.872	0.801	1.36E-03
cds.Camellia_sinensis.comp35362_c0_seq1_m.37823	PREDICTED: ubiquitin-conjugating enzyme E2 2 isoform X1 [Gossypium hirsutum]	17.348	6.6963	15.8	2	3	1	1.01	1.009	0.9	0.931	1.011	1.143	1.057	4.95E-01
cds.Camellia_sinensis.comp35365_c0_seq1_m.39330	PREDICTED: probable phospholipid hydroperoxide glutathione peroxidase isoform X1 [Sesamum indicum]	28.822	45.75	28.7	8	18	5	1.025	1.014	0.993	0.995	0.998	0.997	0.986	2.76E-01
cds.Camellia_sinensis.comp35367_c0_seq1_m.32300	PREDICTED: iron-sulfur cluster assembly protein 1-like [Malus domestica]	18.352	16.06	28.3	4	9	4	0.93	0.967	0.948	1.072	1.025	1.094	1.122	6.80E-03

cds.Camellia_sinensis.comp35368_c0_seq1_m.58448	PREDICTED: ribulose bisphosphate carboxylase/oxygenase activase, chloroplastic [Eucalyptus grandis]	11.731	2.5004	43	4	33	1	1.041	1.002	1.078	0.93	1.007	0.938	0.921	6.66E-02
cds.Camellia_sinensis.comp35369_c0_seq1_m.32992	PREDICTED: ATP-dependent zinc metalloprotease FTSH, chloroplastic-like [Nicotiana tabacum]	38.443	100.36	34.8	11	31	5	0.997	0.99	0.97	1.006	1.022	1.004	1.025	6.56E-02
cds.Camellia_sinensis.comp35376_c0_seq1_m.33068	PREDICTED: light-harvesting complex-like protein 3 isotype 1, chloroplastic [Vitis vinifera]	28.668	74.731	24	7	13	7	1.023	1.022	1.051	0.943	1.002	0.929	0.928	3.96E-02

cds.Camellia_sinensis.comp35389_c0_seq1_m.56728	PREDICTED: heat shock protein 90-1-like [Gossypium raimondii]	14.198	5.8399	33.1	3	6	1	1.074	1.207	1.064	0.879	0.839	0.909	0.785	6.77E-03
cds.Camellia_sinensis.comp35390_c0_seq1_m.27691	PREDICTED: thylakoid lumenal 29 kDa protein, chloroplastic [Vitis vinifera]	38.211	190.71	49.7	16	62	16	1.015	0.977	0.993	0.995	1.036	1.016	1.021	2.70E-01
cds.Camellia_sinensis.comp35393_c0_seq1_m.41325	PREDICTED: nascent polypeptide-associated complex subunit alpha-like protein 2 [Vitis vinifera]	26.78	4.474	9.4	2	2	2	1.052	1.038	0.935	0.891	1.057	1.022	0.982	7.75E-01

cds.Camellia_sinensis.comp35402_c0_seq1_m.34998	PREDICTED: ATP-dependent Clp protease proteolytic subunit 6, chloroplastic-like [Gossypium arboreum]	29.068	12.287	21.9	5	10	5	1.011	0.987	0.95	1.026	1.027	1.021	1.043	1.47E-01
cds.Camellia_sinensis.comp35415_c0_seq1_m.34796	PREDICTED: proteasome subunit beta type-6 [Theobroma cacao]	29.448	155.07	40.4	12	31	12	0.985	0.967	0.959	1.039	1.037	1.014	1.061	5.82E-03
cds.Camellia_sinensis.comp35416_c0_seq1_m.4189	PREDICTED: 2-oxoglutarate dehydrogenase, mitochondrial [Vitis vinifera]	115.44	323.31	35.4	30	51	30	0.992	0.993	0.995	1.015	1.006	1.013	1.018	3.22E-03
cds.Camellia_sinensis.comp35420_c0_seq1_m.48498	PREDICTED: 40S ribosomal protein S2-2 [Theobroma cacao]	13.297	30.235	41.3	6	10	2	1.135	1.167	1.142	0.862	0.793	0.863	0.731	4.24E-04
cds.Camellia_sinensis.comp35423_c0_seq1_m.48297	PREDICTED: actin-like [Ziziphus jujuba]	23.241	21.478	35.7	8	22	0								

cds.Camellia_sinensis.comp35424_c0_seq1_m.42554	PREDICTED: calvin cycle protein CP12-1, chloroplastic [Vitis vinifera]	19.146	97.515	30.9	6	15	6	1.074	1.055	1.074	0.909	0.904	0.926	0.855	7.80E-05
cds.Camellia_sinensis.comp35447_c0_seq1_m.38935	PREDICTED: late embryogenesis abundant protein-like [Capsicum annuum]	24.057	49.586	31.7	7	18	7	1.134	1.079	1.144	0.881	0.852	0.87	0.775	2.56E-04
cds.Camellia_sinensis.comp35455_c0_seq1_m.30880	PREDICTED: probable cinnamyl alcohol dehydrogenase 6 isoform X1 [Jatropha curcas]	42.005	19.105	9.4	4	8	4	1.051	0.997	1.009	1.01	0.964	0.974	0.964	1.64E-01
cds.Camellia_sinensis.comp35459_c0_seq1_m.25644	PREDICTED: chloroplast stem-loop binding protein of 41 kDa b, chloroplastic isoform X2 [Juglans regia]	42.393	139.42	52.4	19	69	19	1.073	1.031	1.033	0.955	0.957	0.943	0.91	2.42E-03

cds.Camellia_sinensis.comp35466_c0_seq1_m.31835	PREDICTED: aspartate-semialdehyde dehydrogenase [Arachis duranensis]	42.612	126.71	37.2	11	22	11	0.98	1.007	1.016	0.968	1.009	0.951	0.975	2.84E-01
cds.Camellia_sinensis.comp35493_c0_seq1_m.19331	PREDICTED: aspartic proteinase-like isoform X1 [Nicotiana tabacum]	56.98	17.012	14.3	6	7	6	1.003	1.06	1.002	0.979	0.937	0.993	0.949	1.10E-01
cds.Camellia_sinensis.comp35503_c0_seq1_m.31945	PREDICTED: probable pyridoxal 5'-phosphate synthase subunit PDX1 [Erythranthe guttata]	33.22	77.227	41.6	13	31	3	0.988	0.906	0.915	0.995	1.043	1.171	1.142	7.72E-02
cds.Camellia_sinensis.comp35522_c0_seq1_m.26786	PREDICTED: fructose-1,6-bisphosphatase, cytosolic [Sesamum indicum]	37.136	161.77	52.6	16	44	16	0.967	0.949	0.959	1.033	1.073	1.051	1.098	1.58E-03

cds.Camellia_sinensis.comp35528_c0_seq1_m.44861	protein PHLOEM PROTEIN 2-LIKE A9-like [Malus domestica]	17.764	5.8544	12.5	2	2	2	1.045	0.959	0.947	1.005	1.031	1.021	1.036	3.19E-01
cds.Camellia_sinensis.comp35531_c0_seq1_m.42382	PREDICTED: mitochondrial pyruvate carrier 1 [Eucalyptus grandis]	14.775	3.76	7.6	1	3	1	0.921	0.933	0.926	1.057	1.146	1.053	1.171	3.00E-02
cds.Camellia_sinensis.comp35556_c0_seq1_m.33839	PREDICTED: adenine phosphoribosyltransferase 1-like [Ipomoea	29.058	178.54	32.2	10	21	10	1.008	0.96	1.01	1.012	0.998	1.032	1.021	3.27E-01
cds.Camellia_sinensis.comp35566_c0_seq1_m.52597	dual specificity phosphatase Cdc25 [Cajanus cajan]	14.406	3.3768	10.8	1	1	1								

cds.Camellia_sinensis.comp35567_c0_seq1_m.24399	PREDICTED: basic leucine zipper and W2 domain-containing protein 2-like [Daucus carota subsp. sativus]	47.198	65.478	20.9	7	8	7	1.123	1.1	1.141	0.866	0.849	0.869	0.768	3.66E-05
cds.Camellia_sinensis.comp35568_c0_seq1_m.15312	PREDICTED: dolichyl-diphosphooligosaccharide--protein glycosyltransferase subunit 1A [Vitis vinifera]	70.656	49.356	17.1	11	15	11	0.988	0.977	0.933	1.02	1.057	1.026	1.071	2.97E-02
cds.Camellia_sinensis.comp35571_c0_seq1_m.42364	PREDICTED: huntingtin-interacting protein K [Sesamum indicum]	12.131	33.053	52.7	7	15	7	1.001	0.982	1.016	0.961	1.004	1.027	0.998	9.10E-01
cds.Camellia_sinensis.comp35577_c0_seq1_m.48965	PREDICTED: heat shock 70 kDa protein 15-like [Prunus mume]	22.539	30.279	19.8	4	8	1	1.026	1.01	0.996	0.979	1.058	0.935	0.98	5.98E-01

cds.Camellia_sinensis.comp35581_c0_seq1_m.39136	PREDICTED: probable NADH dehydrogenase [ubiquinone] 1 alpha subcomplex subunit 12 [Nicotiana attenuata]	21.792	17.451	35.8	7	11	7	1.068	1.042	1.054	0.928	0.962	0.945	0.896	9.56E-04
cds.Camellia_sinensis.comp35582_c0_seq1_m.29174	PREDICTED: putative GDP-L-fucose synthase 2 [Eucalyptus grandis]	35.115	11.916	15.7	6	10	6	1.026	1.01	1.01	0.982	0.992	0.982	0.97	8.60E-03
cds.Camellia_sinensis.comp35584_c0_seq1_m.33004	PREDICTED: peptidyl-prolyl cis-trans isomerase FKBP17-2, chloroplastic [Vitis vinifera]	27.691	35.255	21.6	4	7	4	1.001	1.009	0.959	1.004	1.003	1.035	1.025	2.65E-01

cds.Camellia_sinensis.comp35586_c0_seq1_m.34293	PREDICTED: protein HHL1, chloroplastic isoform X1 [Juglans regia]	26.68	24.876	22.9	5	7	5	1.191	1.171	1.167	0.808	0.816	0.803	0.688	5.76E-07
cds.Camellia_sinensis.comp35589_c0_seq1_m.31522	PREDICTED: clathrin light chain 2-like [Nelumbo nucifera]	33.283	8.3437	14.4	4	6	4	1.076	1.024	1.112	0.96	0.963	0.856	0.865	3.30E-02
cds.Camellia_sinensis.comp35593_c0_seq1_m.42827	PREDICTED: FK506-binding protein 2 [Populus euphratica]	19.619	78.26	29.4	5	12	5	1.156	1.099	1.086	0.898	0.842	0.893	0.788	1.06E-03
cds.Camellia_sinensis.comp35597_c0_seq1_m.22562	PREDICTED: protease Do-like 1, chloroplastic [Nelumbo nucifera]	45.86	96.78	22.7	9	20	9	0.992	0.983	0.991	0.982	1.01	1.024	1.017	2.61E-01
cds.Camellia_sinensis.comp35605_c0_seq1_m.40316	PREDICTED: protein P21-like [Juglans regia]	27.051	57.679	28.7	6	13	6	0.938	0.968	0.975	1.027	1.076	1.057	1.097	6.88E-03

cds.Camellia_sinensis.comp35614_c0_seq1_m.19095	regulator of chromosome condensation family protein [Populus trichocarpa]	57.099	14.624	9.2	5	5	5	0.984	1.055	0.976	1.048	0.978	0.943	0.985	7.12E-01
cds.Camellia_sinensis.comp35619_c0_seq1_m.47230	pectinesterase (Protein of unknown function, DUF538) [Arabidopsis thaliana]	18.162	9.4808	11.4	2	6	2	1.034	1.059	1.033	0.894	0.926	0.961	0.89	6.34E-03
cds.Camellia_sinensis.comp35623_c0_seq1_m.29238	PREDICTED: rhodanese-like domain-containing protein 11, chloroplastic isoform X1 [Vitis vinifera]	33.647	54	36	9	11	9	1.043	1.036	1.059	0.984	0.948	0.956	0.92	3.10E-03
cds.Camellia_sinensis.comp35641_c0_seq1_m.17743	PREDICTED: 3-isopropylmalate dehydratase large subunit-like [Nicotiana sylvestris]	55.293	94.438	40.2	17	35	17	0.98	1.01	1.004	1.002	1.034	1.005	1.016	3.17E-01

cds.Camellia_sinensis.comp35658_c0_seq1_m.42642	PREDICTED: cyanate hydratase 2 isoform X1 [Populus euphratica]	21.41	135.07	48.2	8	23	8	0.921	0.945	0.917	1.086	1.084	1.073	1.165	1.15E-04
cds.Camellia_sinensis.comp35664_c0_seq1_m.36076	PREDICTED: heat shock 70 kDa protein 15-like [Gossypium hirsutum]	21.054	11.653	26.6	6	10	4	1.05	1.048	1.015	0.899	0.954	1.041	0.93	1.65E-01
cds.Camellia_sinensis.comp35666_c0_seq1_m.32810	PREDICTED: bifunctional protein FolD 2 [Vitis vinifera]	32.468	102.34	50.8	16	28	16	0.957	0.964	0.961	1.048	1.045	1.038	1.086	1.99E-05
cds.Camellia_sinensis.comp35670_c0_seq1_m.50049	PREDICTED: LOW QUALITY PROTEIN: glutamate--glyoxylate aminotransferase 2-like [Sesamum indicum]	14.554	60.831	79.8	9	34	5	1.053	1.067	1.046	0.897	0.943	0.962	0.885	4.78E-03

cds.Camellia_sinensis.comp35671_c0_seq1_m.27606	PREDICTED: protein-tyrosine-phosphatase PTP1 isoform X4 [Vitis vinifera]	38.978	3.8124	5.5	2	3	2								
cds.Camellia_sinensis.comp35694_c0_seq1_m.52626	PREDICTED: cytochrome b-c1 complex subunit Rieske-4, mitochondrial-like [Sesamum indicum]	13.98	1.3086	7.8	1	2	1	1.021	0.902	1.005	1.018	0.966	1.098	1.053	3.94E-01
cds.Camellia_sinensis.comp35697_c0_seq1_m.49233	zinc finger/BTB domain protein [Arabidopsis thaliana]	17.019	12.721	25.5	4	5	4	1.128	1.096	1.156	0.889	0.893	0.868	0.784	1.62E-04
cds.Camellia_sinensis.comp35698_c0_seq1_m.53260	PREDICTED: dnaJ protein homolog 2 isoform X2 [Solanum pennellii]	16.8	1.2587	32.3	5	9	1								
cds.Camellia_sinensis.comp35708_c0_seq1_m.33676	Cytochrome b5 isoform 1 [Morus notabilis]	18.896	30.788	20.1	2	4	2	0.926	1.054	1.055	0.996	1	0.916	0.959	4.81E-01

cds.Camellia_sinensis.comp35725_c0_seq1_m.40948	PREDICTED: protein SRC2 homolog [Ricinus communis]	26.043	6.7499	5.1	1	1	1								
cds.Camellia_sinensis.comp35727_c0_seq1_m.58807	PREDICTED: phosphoribulokinase, chloroplastic-like [Nelumbo	10.544	11.602	26.2	2	9	1	0.946	0.99	0.976	0.99	1.086	1.032	1.067	9.72E-02
cds.Camellia_sinensis.comp35729_c0_seq1_m.33389	PREDICTED: coiled-coil domain-containing protein 124 [Vitis vinifera]	26.485	1.7892	3.9	1	1	1	1.093	1.101	1.013	0.927	0.903	0.945	0.865	8.34E-03
cds.Camellia_sinensis.comp35730_c0_seq1_m.24381	PREDICTED: thiamine pyrophosphokinase 1 isoform X1 [Juglans regia]	35.054	7.251	14.9	5	5	5	1.071	1.061	0.985	0.935	0.966	0.974	0.922	5.18E-02
cds.Camellia_sinensis.comp35731_c0_seq1_m.25816	PREDICTED: 5'-nucleotidase SurE [Prunus mume]	33.19	10.332	7.1	2	2	2	1.056	1.043	1.035	0.944	0.912	1.001	0.912	2.85E-02

cds.Camellia_sinensis.comp35736_c0_seq1_m.15954	PREDICTED: uncharacterized membrane protein At1g16860-like [Ipomoea nil]	51.735	4.6506	4.3	2	2	2	0.83	1.179	1.029	1.032	1.028	0.93	0.984	9.53E-01
cds.Camellia_sinensis.comp35757_c0_seq1_m.13862	PREDICTED: 3-phosphoshikimate 1-carboxyvinyltransferase 2 [Juglans regia]	55.63	53.83	20.8	9	18	9	1.124	1.158	1.158	0.814	0.822	0.808	0.71	3.33E-06
cds.Camellia_sinensis.comp35765_c0_seq1_m.14995	PREDICTED: nephrocystin-3 [Daucus carota subsp. sativus]	66.62	9.7843	8.6	4	4	4	1.444	1.201	0.695	0.798	0.81	0.974	0.773	3.98E-01
cds.Camellia_sinensis.comp35772_c0_seq1_m.9930	PREDICTED: glyoxysomal fatty acid beta-oxidation multifunctional protein MFP-a [Jatropha curcas]	79.026	73.568	31.3	23	46	23	0.973	0.977	1.004	1.019	1.023	1.012	1.034	3.20E-02

cds.Camellia_sinensis.comp35775_c0_seq1_m.39035	PREDICTED: dnaJ protein homolog [Nicotiana tabacum]	26.466	2.2782	4.7	1	1	1	1.215	1.207	1.163	0.726	0.827	0.8	0.656	5.20E-04
cds.Camellia_sinensis.comp35776_c0_seq1_m.30569	PREDICTED: ER membrane protein complex subunit 10 [Theobroma cacao]	33.904	5.3731	14.9	5	5	5	0.953	0.979	1.037	0.994	1.002	1.084	1.037	3.82E-01
cds.Camellia_sinensis.comp35781_c0_seq1_m.36035	PREDICTED: putative UDP-rhamnose:rhamnosyltransferase 1 [Vitis vinifera]	35.201	3.8539	6	2	3	2	1.048	1.179	1.209	0.864	0.842	0.83	0.738	2.70E-03
cds.Camellia_sinensis.comp35782_c0_seq1_m.21655	PREDICTED: calreticulin-3-like [Populus euphratica]	53.159	24.522	16.7	8	8	6	1.088	1.068	1.125	0.905	0.942	0.868	0.827	2.50E-03

cds.Camellia_sinensis.comp35788_c0_seq1_m.35839	PREDICTED: single-stranded DNA-binding protein WHY1, chloroplastic-like [Nicotiana tomentosiformis]	30.892	78.031	32.7	7	8	7	0.987	1.008	0.987	0.961	0.993	1.023	0.998	9.24E-01
cds.Camellia_sinensis.comp35803_c0_seq1_m.54656	PREDICTED: GTP-binding protein SAR1A-like [Arachis ipaensis]	12.06	8.8721	50.5	4	9	1	1.027	0.972	1.022	0.938	1.053	0.991	0.987	7.33E-01
cds.Camellia_sinensis.comp35812_c0_seq1_m.44084	PREDICTED: leiomodin-2 isoform X1 [Capsicum annuum]	19.714	21.876	17.7	4	9	4	1.02	0.951	1.004	0.995	1.048	1.028	1.032	2.87E-01
cds.Camellia_sinensis.comp35815_c0_seq1_m.45037	PREDICTED: heat shock factor-binding protein 1 [Theobroma cacao]	14.518	21.426	32.6	4	5	4	0.941	0.963	0.972	1.052	1.045	1.031	1.088	1.76E-03

cds.Camellia_sinensis.comp35826_c0_seq1_m.50055	PREDICTED: aldehyde dehydrogenase family 2 member B4, mitochondrial-like, partial [Malus domestica]	11.685	17.758	38.7	6	13	4	1.033	0.997	0.989	0.962	0.995	1.009	0.982	4.14E-01
cds.Camellia_sinensis.comp35836_c0_seq1_m.16144	PREDICTED: 4-coumarate--CoA ligase-like 7 [Nicotiana tabacum]	59.481	116.84	36	18	29	18	0.996	0.981	0.989	1.007	1.003	1.028	1.024	5.29E-02
cds.Camellia_sinensis.comp35854_c0_seq1_m.36569	PREDICTED: apoptotic chromatin condensation inducer in the nucleus-like isoform X2 [Juglans regia]	22.996	1.9246	7.1	2	3	2	1.021	1.101	1.113	0.922	0.885	0.946	0.851	8.48E-03

cds.Camellia_sinensis.comp35858_c0_seq1_m.34257	PREDICTED: gamma carbonic anhydrase-like 2, mitochondrial [Jatropha curcas]	27.529	18.788	17.1	5	9	5	1.019	1.04	1.046	0.949	0.917	1.058	0.942	2.31E-01
cds.Camellia_sinensis.comp35860_c0_seq1_m.31307	PREDICTED: beta-carotene isomerase D27, chloroplastic [Juglans regia]	31.178	4.6969	8.6	2	4	2	1.036	1.125	1.098	0.855	0.918	0.957	0.838	1.23E-02
cds.Camellia_sinensis.comp35861_c0_seq1_m.53807	PREDICTED: reticulon-like protein B1 [Vitis vinifera]	16.668	1.5626	7.4	1	1	1								
cds.Camellia_sinensis.comp35876_c0_seq1_m.40720	quinone reductase family protein [Arabidopsis lyrata subsp. lyrata]	21.74	132.29	53.7	9	16	5	0.973	1.041	1.053	1.039	1.008	0.929	0.97	5.02E-01

cds.Camellia_sinensis.comp35880_c0_seq1_m.17775	PREDICTED: proline--tRNA ligase, cytoplasmic-like [Nelumbo nucifera]	58.213	113.25	35.7	15	24	7	1.025	1.005	1.05	0.924	0.955	0.93	0.912	4.74E-03
cds.Camellia_sinensis.comp35884_c0_seq1_m.33763	PREDICTED: glutathione S-transferase DHAR3, chloroplastic-like [Juglans regia]	30.256	73.884	44.3	12	35	11	0.958	0.965	0.979	1.046	1.061	1.044	1.086	5.41E-04
cds.Camellia_sinensis.comp35887_c0_seq1_m.54467	PREDICTED: glycine dehydrogenase (decarboxylating), mitochondrial [Nelumbo nucifera]	15.759	17.381	14.8	2	7	2	1.006	0.99	1	0.988	0.985	0.998	0.992	2.44E-01
cds.Camellia_sinensis.comp35890_c0_seq1_m.40314	heterogeneous nuclear ribonucleoprotein A1-like 2 [Cajanus cajan]	32.541	3.8679	9.5	3	11	2	1.005	1.014	0.997	0.971	1.071	0.991	1.006	8.83E-01

cds.Camellia_sinensis.comp35892_c0_seq1_m.47220	PREDICTED: putative UDP-rhamnose:rhamnosyltransferase 1 [Populus euphratica]	18.03	2.0242	11.8	2	2	2	1.127	1.079	1.064	0.948	0.871	0.89	0.828	3.58E-03
cds.Camellia_sinensis.comp35902_c0_seq1_m.7329	PREDICTED: V-type proton ATPase subunit a3 [Vitis	92.954	91.381	21.3	14	22	14	1.007	1.016	0.958	1.018	1.031	0.999	1.022	3.34E-01
cds.Camellia_sinensis.comp35952_c0_seq1_m.28377	PREDICTED: calcyclin-binding protein [Vitis vinifera]	25.139	7.4392	14.8	4	4	4	0.99	0.969	0.988	0.988	1.023	1.031	1.032	9.92E-02
cds.Camellia_sinensis.comp35956_c0_seq1_m.38504	PREDICTED: eukaryotic translation initiation factor 3 subunit K-like [Populus euphratica]	26.977	4.5408	14	4	4	4	0.862	0.946	0.943	1.141	1.037	1.092	1.189	1.36E-02

cds.Camellia_sinensis.comp35959_c0_seq1_m.28749	PREDICTED: phosphoenolpyruvate carboxylase [Capsicum annuum]	37.521	78.93	49.2	15	27	10	1.027	1.035	1.021	0.941	0.974	0.954	0.931	2.60E-03
cds.Camellia_sinensis.comp35960_c0_seq1_m.40073	PREDICTED: probable xyloglucan endotransglucosylase/hydrolase protein 21 [Gossypium hirsutum]	25.651	18.331	6.6	1	1	1	1.073	0.99	0.922	0.931	1.013	1.067	1.009	8.86E-01
cds.Camellia_sinensis.comp35961_c0_seq1_m.31683	calcium homeostasis regulator CHoR1 [Solanum tuberosum	36.586	51.282	15.9	4	5	4	1.017	1.011	1.015	1.002	1.019	0.944	0.974	3.73E-01
cds.Camellia_sinensis.comp35964_c0_seq1_m.25987	PREDICTED: photosystem II stability/assembly factor HCF136, chloroplastic [Nelumbo nucifera]	46.271	254.44	39	14	38	14	0.999	0.98	0.999	1.005	0.991	1.008	1.009	3.52E-01

cds.Camellia_sinensis.comp3598_c0_seq1_m.48410	PREDICTED: trans-resveratrol di-O-methyltransferase [Vitis vinifera]	21.142	23.263	28	4	6	4	0.892	0.868	0.888	1.126	1.156	1.145	1.294	2.14E-05
cds.Camellia_sinensis.comp35991_c0_seq1_m.6464	PREDICTED: beta-galactosidase 10 [Vitis vinifera]	103.07	37.854	18.6	17	22	15	1.083	1.062	1.061	0.875	0.941	0.948	0.862	4.88E-03
cds.Camellia_sinensis.comp36001_c0_seq1_m.34430	PREDICTED: vesicle-associated protein 4-1-like isoform X2 [Ipomoea nil]	28.964	7.0504	16.7	4	4	4	0.992	0.97	1.086	0.902	1.003	1.049	0.969	6.00E-01
cds.Camellia_sinensis.comp36014_c0_seq1_m.41139	PREDICTED: RNA polymerase II subunit A C-terminal domain phosphatase SSU72 [Ricinus communis]	22.681	7.3112	6.2	1	1	1	0.956	0.971	0.96	1.146	1.058	0.94	1.089	2.91E-01

cds.Camellia_sinensis.comp36040_c0_seq1_m.13820	PREDICTED: cell division protein FtsZ homolog 2-1, chloroplastic isoform X1 [Citrus sinensis]	51.412	39.442	18.1	7	12	6	0.964	1.063	1.078	0.893	0.969	1	0.922	1.70E-01
cds.Camellia_sinensis.comp36045_c0_seq1_m.37397	PREDICTED: nectarin-1 [Eucalyptus grandis]	23.629	6.1942	11.1	3	6	3	1.015	0.968	1.035	0.994	1.024	1.022	1.007	7.49E-01
cds.Camellia_sinensis.comp36068_c0_seq1_m.43701	PREDICTED: triosephosphate isomerase, cytosolic [Prunus mume]	17.268	119.61	47.5	6	16	6	0.986	0.965	0.964	1.08	1.006	1.028	1.068	4.20E-02
cds.Camellia_sinensis.comp36069_c0_seq1_m.40367	PREDICTED: universal stress protein A-like protein [Nicotiana attenuata]	17.951	19.571	23.9	4	7	4	0.976	1.019	0.955	1.016	1.01	1.022	1.033	1.63E-01

cds.Camellia_sinensis.comp36071_c0_seq1_m.59211	PREDICTED: reticulon-like protein B1 [Daucus carota subsp. sativus]	11.192	6.7362	18.8	2	2	2	1.062	1.019	1.045	0.91	1.002	0.954	0.917	4.47E-02
cds.Camellia_sinensis.comp36078_c0_seq1_m.57530	PREDICTED: elongation factor 1-delta-like [Juglans regia]	12.047	3.4778	32.5	3	9	1	0.917	1.081	1.012	1.108	0.895	1.007	1	9.85E-01
cds.Camellia_sinensis.comp36119_c0_seq1_m.23684	PREDICTED: haloacid dehalogenase-like hydrolase domain-containing protein At3g48420 isoform X1 [Theobroma cacao]	40.503	32.846	18.5	7	14	7	0.956	1.017	0.996	0.973	0.983	1.003	0.997	8.85E-01
cds.Camellia_sinensis.comp36132_c0_seq1_m.37343	PREDICTED: 50S ribosomal protein L11, chloroplastic [Nicotiana tomentosiformis]	26.731	21.071	29.8	7	16	7	1.017	1.015	1.009	0.975	0.981	0.978	0.965	2.64E-04

cds.Camellia_sinensis.comp36154_c0_seq1_m.40268	PREDICTED: 60S ribosome subunit biogenesis protein NIP7 homolog [Vitis vinifera]	20.921	8.9644	9.1	1	1	1	0.855	0.963	1.21	1.027	1	0.974	0.991	9.91E-01
cds.Camellia_sinensis.comp36157_c0_seq1_m.18410	PREDICTED: preprotein translocase subunit SCY1, chloroplastic-like isoform X1 [Populus euphratica]	59.383	14.664	16.2	8	8	8	0.954	0.955	0.958	1.049	1.069	1.012	1.092	3.23E-02
cds.Camellia_sinensis.comp36191_c0_seq1_m.43051	verprolin [Ananas comosus]	14.126	2.0647	6.8	1	1	1	0.992	0.947	1.003	1.003	1.044	1.026	1.045	1.07E-01
cds.Camellia_sinensis.comp36199_c0_seq1_m.25892	PREDICTED: very-long-chain 3-oxoacyl-CoA reductase 1 [Juglans regia]	35.595	5.1761	11.2	4	4	4	1.276	1.221	1.168	0.709	0.7	0.803	0.604	5.62E-04

cds.Camellia_sinensis.comp36262_c0_seq1_m.44784	transmembrane protein [Arabidopsis thaliana]	14.622	38.584	17.3	4	6	4	1.009	1.026	0.981	0.946	1.015	1.037	0.994	8.41E-01
cds.Camellia_sinensis.comp36281_c0_seq1_m.57270	PREDICTED: aspartic proteinase-like isoform X1 [Juglans regia]	12.684	13.27	20.7	2	5	2	0.973	1.028	1.001	0.955	1.022	1.031	1.002	9.57E-01
cds.Camellia_sinensis.comp36290_c0_seq1_m.44094	PREDICTED: protein canopy-1 [Vitis vinifera]	23.03	17.86	23.8	4	7	4	1.118	1.167	1.194	0.83	0.834	0.834	0.718	3.40E-03
cds.Camellia_sinensis.comp36306_c0_seq1_m.27863	PREDICTED: uridylate kinase-like [Nicotiana tabacum]	37.36	6.6769	7.2	2	2	2	0.889	0.919	1.01	1.068	1.096	1.056	1.143	2.76E-02
cds.Camellia_sinensis.comp36311_c0_seq1_m.37088	PREDICTED: cytosolic sulfotransferase 15-like [Populus euphratica]	37.427	3.1918	9	3	5	2	0.922	1.043	0.965	0.962	1.029	1.096	1.054	3.74E-01

cds.Camellia_sinensis.comp36326_c0_seq1_m.11410	PREDICTED: acyl-coenzyme A oxidase 3, peroxisomal [Vitis vinifera]	76.486	33.514	10.2	7	12	7	0.983	1.026	1.029	1.008	0.958	0.97	0.966	1.84E-01
cds.Camellia_sinensis.comp36355_c0_seq1_m.46734	PREDICTED: auxin-repressed 12.5 kDa protein [Nelumbo nucifera]	12.814	5.4048	21.2	2	2	2	0.806	0.936	1.017	1.226	1.073	1.002	1.196	1.16E-01
cds.Camellia_sinensis.comp36361_c0_seq1_m.27775	PREDICTED: protein CIA1 [Ipomoea nil]	44.375	15.979	7.3	3	6	3	1.01	1.04	1.016	1.006	0.979	0.954	0.959	7.49E-02
cds.Camellia_sinensis.comp36377_c0_seq1_m.36798	pyridoxamine 5'-phosphate oxidase family protein [Medicago truncatula]	31.796	48.93	25	6	12	6	0.981	1.079	0.981	1.049	1	0.958	0.989	8.04E-01
cds.Camellia_sinensis.comp36378_c0_seq1_m.22456	PREDICTED: phospholipase A1-IIdelta isoform X2 [Solanum tuberosum	47.548	15.938	10.3	4	8	4	0.969	0.975	0.955	1.013	1.022	0.988	1.043	2.42E-02

cds.Camellia_sinensis.comp36402_c0_seq1_m.27301	PREDICTED: L-lactate dehydrogenase B-like [Gossypium hirsutum]	37.778	18.094	10.3	3	3	3	0.987	0.991	0.997	0.952	0.987	1.094	1.019	7.15E-01
cds.Camellia_sinensis.comp36409_c0_seq1_m.19147	PREDICTED: translation initiation factor IF-2-like [Solanum pennellii]	52.942	1.4165	2.2	1	1	1								
cds.Camellia_sinensis.comp36424_c0_seq1_m.18168	PREDICTED: protein LURP-one-related 10-like [Nicotiana sylvestris]	25.015	1.57	6.3	2	2	2	1.002	0.955	1.003	0.968	1.098	0.987	1.031	5.23E-01
cds.Camellia_sinensis.comp36431_c0_seq1_m.41059	FKBP-type peptidyl-prolyl cis-trans isomerase 3 family protein [Populus trichocarpa]	24.262	9.5421	13	2	4	2	1.033	1.072	0.976	0.953	0.961	0.96	0.933	1.29E-01

cds.Camellia_sinensis.comp36483_c0_seq1_m.36245	PREDICTED: early nodulin-like protein 1 [Nicotiana attenuata]	30.285	2.1488	3.6	1	1	1	0.918	0.881	0.91	1.099	1.14	1.093	1.23	3.40E-04
cds.Camellia_sinensis.comp36485_c0_seq1_m.31618	PREDICTED: protein OS-9 homolog [Daucus carota subsp. sativus]	40.345	5.6964	8.9	3	3	3	0.961	0.919	1.064	1.104	0.987	0.991	1.047	4.60E-01
cds.Camellia_sinensis.comp36514_c0_seq1_m.48039	PREDICTED: glycine-rich RNA-binding protein RZ1B-like [Ziziphus jujuba]	16.847	1.4399	4.5	1	1	1	1.183	1.11	1.186	0.703	0.871	0.893	0.709	1.15E-02
cds.Camellia_sinensis.comp36522_c0_seq1_m.30982	PREDICTED: replication factor C subunit 2 [Eucalyptus grandis]	38.637	4.6018	8.1	2	2	2	0.932	0.997	0.992	1.061	1.04	1.003	1.063	8.86E-02

cds.Camellia_sinensis.comp36530_c0_seq1_m.37183	PREDICTED: LOW QUALITY PROTEIN: tropinone reductase-like 3 [Daucus carota subsp. sativus]	29.649	3.7353	6.9	2	3	2	1.022	1.05	1.012	1.013	0.951	0.954	0.946	7.55E-02
cds.Camellia_sinensis.comp36539_c0_seq1_m.4777	PREDICTED: mannosylglycoprotein endo-beta-mannosidase [Vitis	110.57	21.426	11.1	10	10	10	0.982	0.996	0.994	1.028	1.008	0.982	1.015	3.38E-01
cds.Camellia_sinensis.comp36549_c0_seq1_m.49530	PREDICTED: V-type proton ATPase 16 kDa proteolipid subunit [Fragaria vesca subsp.	14.909	-2	12.1	1	1	1	0.835	0.897	1	1.128	1.229	0.967	1.217	9.10E-02
cds.Camellia_sinensis.comp36555_c0_seq1_m.57037	PREDICTED: polyubiquitin, partial [Raphanus sativus]	13.224	117.67	74.6	6	46	6	1.039	1.011	1.006	0.948	0.974	0.982	0.95	2.52E-02

cds.Camellia_sinensis.comp36564_c0_seq1_m.18010	PREDICTED: protein disulfide isomerase-like 1-4 [Populus euphratica]	67.5	27.807	10.8	7	7	7	1.04	1.014	1.06	0.973	0.957	0.921	0.916	1.30E-02
cds.Camellia_sinensis.comp36568_c0_seq1_m.11790	PREDICTED: protein SIEVE ELEMENT OCCLUSION B [Ricinus communis]	83.418	263.13	38.9	26	50	26	0.934	0.941	0.938	1.051	1.088	1.064	1.139	2.37E-04
cds.Camellia_sinensis.comp36571_c0_seq1_m.32975	PREDICTED: casein kinase 1-like protein 2 [Nicotiana attenuata]	20.561	6.7704	6.2	1	1	1								
cds.Camellia_sinensis.comp36576_c0_seq1_m.20868	PREDICTED: UDP-N-acetylglucosamine diphosphorylase 1 [Citrus sinensis]	55.479	13.619	8.6	3	4	3	1.05	1	0.98	0.959	0.945	1.038	0.971	4.48E-01
cds.Camellia_sinensis.comp36580_c0_seq1_m.17447	PREDICTED: protein FLX-like 2 [Ziziphus jujuba]	50.983	79.218	40.1	19	26	19	1.004	1.029	0.972	1.012	0.974	1.011	0.997	9.08E-01

cds.Camellia_sinensis.comp36602_c0_seq1_m.7660	PREDICTED: carbon catabolite repressor protein 4 homolog 1 [Pyrus x bretschneideri]	66.865	4.0721	1.8	1	1	1								
cds.Camellia_sinensis.comp36606_c0_seq1_m.29147	PREDICTED: phosphoglycerate mutase-like protein 1 [Prunus mume]	39.804	20.594	15.7	5	7	5	1.023	1.074	1.016	0.95	0.981	0.994	0.94	4.79E-02
cds.Camellia_sinensis.comp36612_c0_seq1_m.34117	calcium-dependent phosphotriesterase superfamily protein [Medicago truncatula]	36.199	59.094	37	9	15	9	0.973	0.956	1.003	1.013	1.03	1.018	1.044	4.37E-02
cds.Camellia_sinensis.comp36627_c0_seq1_m.24222	PREDICTED: chlorophyll synthase, chloroplastic [Prunus mume]	40.487	27.013	8.8	2	2	2	0.941	0.949	0.958	1.071	1.07	1.039	1.117	6.02E-04

cds.Camellia_sinensis.comp36670_c0_seq1_m.28249	PREDICTED: replication factor C subunit 3 [Gossypium raimondii]	41.896	6.6658	11.7	5	5	5	0.973	0.972	0.932	1.023	1.053	1.048	1.086	7.94E-03
cds.Camellia_sinensis.comp36696_c0_seq1_m.28755	PREDICTED: probable pyridoxal 5'-phosphate synthase subunit PDX1 [Daucus carota subsp. sativus]	37.041	3.3632	35.2	12	31	2	0.952	0.948	0.955	1.059	1.047	1.061	1.109	2.21E-05
cds.Camellia_sinensis.comp36700_c0_seq1_m.39603	sodium/calcium exchanger family protein / calcium-binding EF hand family protein [Arabidopsis thaliana]	34.335	11.168	9.8	2	2	2	0.883	0.923	1.071	1.15	0.965	1.045	1.098	2.88E-01

cds.Camellia_sinensis.comp36719_c0_seq1_m.52881	PREDICTED: heat shock 70 kDa protein, mitochondrial-like [Nicotiana tabacum]	17.549	12.197	44.7	9	25	3	0.905	1	1.002	1.024	1.035	1.09	1.083	1.06E-01
cds.Camellia_sinensis.comp36733_c0_seq1_m.44838	PREDICTED: UPF0426 protein At1g28150, chloroplastic [Prunus mume]	13.843	19.999	26.8	3	4	3	0.879	0.948	0.888	1.111	1.116	1.117	1.232	1.27E-02
cds.Camellia_sinensis.comp36734_c0_seq1_m.26682	PREDICTED: probable membrane-associated 30 kDa protein, chloroplastic [Nelumbo nucifera]	36.17	255.58	57.3	18	59	18	1.031	1.01	0.976	0.981	0.99	1.007	0.987	5.10E-01
cds.Camellia_sinensis.comp36751_c0_seq1_m.21704	PREDICTED: protein STRICTOSIDINE SYNTHASE-LIKE 3-like [Jatropha curcas]	43.85	64.193	34.1	12	16	12	1.091	1.116	1.058	0.871	0.886	0.92	0.82	8.95E-04

cds.Camellia_sinensis.comp36757_c0_seq1_m.20481	PREDICTED: actin-related protein 4 isoform X1 [Ziziphus jujuba]	54.079	45.258	32.4	11	11	11	0.965	1.095	0.948	1.053	1.003	0.994	1.014	7.65E-01
cds.Camellia_sinensis.comp36769_c0_seq1_m.58934	PREDICTED: organellar oligopeptidase A, chloroplastic/mitochondrial-like, partial [Nicotiana tabacum]	11.713	7.6225	34	4	7	4	0.911	0.965	1.006	1.062	1.034	1.03	1.085	5.39E-02
cds.Camellia_sinensis.comp36801_c0_seq1_m.55689	PREDICTED: aldehyde dehydrogenase family 2 member B7, mitochondrial [Eucalyptus grandis]	14.435	56.327	53.4	5	9	5	0.939	0.924	0.941	1.091	1.048	1.07	1.144	4.81E-04
cds.Camellia_sinensis.comp36804_c0_seq1_m.45298	transmembrane protein, putative [Medicago truncatula]	17.492	24.943	23.1	3	4	3	0.99	0.979	0.987	1.012	1.023	1.003	1.028	1.44E-02

cds.Camellia_sinensis.comp36845_c0_seq1_m.56300	probable mitochondrial-processing peptidase subunit beta [Cucumis melo]	14.372	10.274	24.8	3	5	1	0.958	1.025	1.031	1.045	0.953	1.003	0.996	9.06E-01
cds.Camellia_sinensis.comp36862_c0_seq1_m.44499	PREDICTED: pectin acetylesterase 8-like [Juglans regia]	27.734	8.5182	11.8	3	4	3	1.013	1.032	1.043	0.988	0.966	0.961	0.944	8.74E-03
cds.Camellia_sinensis.comp36945_c0_seq1_m.52410	PREDICTED: probable phospholipid hydroperoxide glutathione peroxidase [Ricinus communis]	11.142	12.953	43	5	12	2	0.949	0.925	0.942	1.096	1.055	1.063	1.141	6.84E-04
cds.Camellia_sinensis.comp37006_c0_seq1_m.34879	PREDICTED: pre-mRNA-splicing factor SPF27 homolog [Vitis vinifera]	30.057	12.138	16.3	5	6	5	0.977	1.007	1.043	0.989	1.021	0.979	0.987	6.13E-01

cds.Camellia_sinensis.comp37024_c0_seq1_m.39511	PREDICTED: small heat shock protein, chloroplastic-like isoform X2 [Nelumbo nucifera]	22.449	19.429	35.2	6	13	6	0.975	0.953	1.013	1.047	1.035	1.02	1.055	5.04E-02
cds.Camellia_sinensis.comp37091_c0_seq1_m.40308	PREDICTED: ATP-dependent zinc metalloprotease FTSH 10, mitochondrial [Ricinus communis]	26.826	2.2185	4.6	1	1	1	1.121	1.153	0.984	0.92	0.902	0.895	0.834	2.23E-02
cds.Camellia_sinensis.comp37118_c0_seq1_m.40237	PREDICTED: eukaryotic translation initiation factor 4B2 [Vitis vinifera]	33.312	2.7258	3.3	1	1	1	1.038	1.117	1.147	0.874	0.969	0.839	0.812	1.61E-02
cds.Camellia_sinensis.comp37124_c0_seq1_m.21044	PREDICTED: chaperone protein dnaJ 10 [Prunus mume]	38.789	18.414	17.5	6	8	6	1.043	0.984	1.023	1.048	0.966	0.98	0.982	5.68E-01

cds.Camellia_sinensis.comp37127_c0_seq1_m.43123	PREDICTED: 30S ribosomal protein 3-1, chloroplastic-like [Populus euphratica]	22.557	4.1082	6	1	3	1								
cds.Camellia_sinensis.comp37161_c0_seq1_m.54381	PREDICTED: dynein light chain LC6, flagellar outer arm [Ziziphus	11.778	3.7654	22.4	2	2	2	0.899	0.999	0.967	1.079	1.018	1.069	1.105	4.93E-02
cds.Camellia_sinensis.comp37196_c0_seq1_m.31940	PREDICTED: cinnamoyl-CoA reductase 2 [Sesamum indicum]	37.462	5.8002	8.7	3	4	3	0.903	0.903	0.997	1.05	1.035	1.144	1.152	3.57E-02
cds.Camellia_sinensis.comp37365_c0_seq1_m.23220	PREDICTED: tyrosine--tRNA ligase, chloroplastic/mitochondrial-like [Gossypium hirsutum]	59.345	1.7901	3.7	2	2	2	0.98	0.978	1.027	1.019	1.026	0.985	1.015	5.00E-01

cds.Camellia_sinensis.comp37458_c0_seq1_m.24872	PREDICTED: sec-independent protein translocase protein TATC, chloroplastic [Ziziphus jujuba]	39.076	19.004	10.4	2	2	2	1.011	1.002	1.026	0.969	0.971	1.024	0.975	2.61E-01
cds.Camellia_sinensis.comp37631_c0_seq1_m.44158	PREDICTED: mavicyanin-like [Gossypium hirsutum]	18.427	2.0941	9.7	2	2	2	1.13	1.157	1.213	0.833	0.807	0.818	0.702	1.01E-04
cds.Camellia_sinensis.comp37633_c0_seq1_m.16602	PREDICTED: sucrose transport protein SUC4 [Theobroma cacao]	54.176	1.9145	1.6	1	1	1	1.047	1.007	1.07	0.957	0.936	0.978	0.919	1.81E-02
cds.Camellia_sinensis.comp37744_c0_seq1_m.41489	PREDICTED: arabinosyltransferase RRA3-like [Nelumbo nucifera]	32.58	6.8766	4.8	1	1	1	1.043	0.934	1.031	0.96	0.992	1.041	0.995	9.23E-01
cds.Camellia_sinensis.comp37907_c0_seq1_m.56509	PREDICTED: fruit protein pKIWI502-like [Nicotiana tabacum]	13.301	20.582	28.7	3	5	2	1.178	0.977	1.112	0.877	0.985	0.844	0.828	6.03E-02

cds.Camellia_sinensis.comp38000_c0_seq1_m.28599	PREDICTED: prostaglandin E synthase 2-like [Jatropha curcas]	43.273	11.411	11	5	7	4	1.014	1.007	1.018	0.986	1.005	0.951	0.968	1.19E-01
cds.Camellia_sinensis.comp38035_c0_seq1_m.22259	PREDICTED: GDP-Man:Man(3)GlcNAc(2)-PP-Dol alpha-1,2-mannosyltransferase [Citrus sinensis]	52.521	11.603	7.5	3	4	3	0.822	0.906	1.033	1.124	1.008	1.056	1.155	1.15E-01
cds.Camellia_sinensis.comp38050_c0_seq1_m.42138	PREDICTED: phenylalanine ammonia-lyase G4 [Ricinus communis]	26.074	3.7057	24.1	7	13	3	1.227	1.231	1.292	0.762	0.75	0.707	0.592	4.47E-05
cds.Camellia_sinensis.comp38095_c0_seq1_m.52871	PREDICTED: transmembrane protein 120 homolog [Solanum tuberosum	14.949	12.507	23.1	3	5	2	0.985	1.116	1.022	0.973	0.95	0.954	0.921	9.94E-02

cds.Camellia_sinensis.comp38139_c0_seq1_m.32128	PREDICTED: D-amino-acid transaminase, chloroplastic [Eucalyptus grandis]	39.473	5.1197	7.5	3	4	3	1.031	1.026	0.996	1	1.006	0.945	0.967	2.05E-01
cds.Camellia_sinensis.comp38204_c0_seq1_m.10632	PREDICTED: flowering time control protein FY [Vitis vinifera]	81.936	5.6821	2.4	2	2	2	0.943	1.029	0.942	1.054	0.964	1.088	1.066	2.45E-01
cds.Camellia_sinensis.comp38251_c0_seq1_m.43401	PREDICTED: glycine-rich RNA-binding protein 6, mitochondrial [Prunus	15.393	3.0006	9.4	1	1	1								
cds.Camellia_sinensis.comp38448_c0_seq1_m.28380	transmembrane protein, putative [Medicago truncatula]	37.351	3.9296	3	1	1	1	0.92	0.977	1.022	1.038	1.067	1.003	1.065	1.47E-01
cds.Camellia_sinensis.comp38515_c0_seq1_m.33013	heat shock protein binding protein [Arabidopsis lyrata subsp. lyrata]	33.03	8.6961	12.8	4	6	4	1.004	1.024	1.059	0.955	0.964	0.994	0.944	4.24E-02

cds.Camellia_sinensis.comp38553_c0_seq1_m.49905	PREDICTED: 10 kDa chaperonin-like [Sesamum indicum]	11.983	3.4024	17.4	2	3	2	0.991	1.005	1.05	1.004	1	0.96	0.973	2.94E-01
cds.Camellia_sinensis.comp38568_c0_seq1_m.40718	PREDICTED: pantothenate kinase 1 isoform X2 [Ziziphus jujuba]	20.76	16.641	24.2	3	3	3	0.958	0.946	1.022	0.995	1.053	1.045	1.057	1.35E-01
cds.Camellia_sinensis.comp38637_c0_seq1_m.34256	PREDICTED: pyridoxal 5'-phosphate synthase-like subunit PDX1.2 [Nicotiana tomentosiformis]	32.845	62.895	12.1	3	4	2	1.025	1.002	1.082	0.953	0.969	0.968	0.93	3.63E-02

cds.Camellia_sinensis.comp38664_c0_seq1_m.43903	PREDICTED: protein HOMOLOG OF MAMMALIAN LYST-INTERACTING PROTEIN 5-like isoform X2 [Ziziphus jujuba]	26.14	2.2908	5.2	1	1	1	0.969	1.068	1.157	0.921	0.903	0.979	0.878	8.62E-02
cds.Camellia_sinensis.comp38927_c0_seq1_m.45078	PREDICTED: dihydrolipoyllysine-residue acetyltransferase component 1 of pyruvate dehydrogenase complex,mitochondrial [Prunus mume]	26.622	1.8582	9.7	2	4	1	1.027	0.988	1.012	1.006	0.946	1.025	0.983	5.57E-01

cds.Camellia_sinensis.comp39032_c0_seq1_m.57934	PREDICTED: 4-hydroxy-3-methylbut-2-en-1-yl diphosphate synthase (ferredoxin), chloroplastic-like [Prunus mume]	12.312	15.127	9.1	1	3	1	1.088	1.094	1.036	0.942	0.914	0.911	0.86	1.76E-03
cds.Camellia_sinensis.comp39049_c0_seq1_m.47896	PREDICTED: acyl-protein thioesterase 2 [Ricinus communis]	12.752	28.227	15	2	2	2	0.943	0.979	1.071	1.011	0.965	1.047	1.01	8.19E-01
cds.Camellia_sinensis.comp39084_c0_seq1_m.17097	dicarboxylate transporter 2.1, chloroplastic-like [Nicotiana tabacum]	65.498	17.499	6.1	3	6	3	1.012	0.951	1.026	0.998	1.05	1.028	1.029	3.53E-01
cds.Camellia_sinensis.comp39106_c0_seq1_m.45247	glycine-rich RNA-binding protein 4, mitochondrial [Asparagus officinalis]	19.515	3.1439	9.8	2	2	2								

cds.Camellia_sinensis.comp39475_c0_seq1_m.51706	receptor-interacting protein, putative [Medicago truncatula]	16.397	19.621	31.2	3	3	3	1.034	1.039	0.988	1.135	0.959	0.858	0.964	6.34E-01
cds.Camellia_sinensis.comp39568_c0_seq1_m.46204	PREDICTED: low-temperature-induced cysteine proteinase-like [Juglans regia]	21.795	2.2803	9.9	3	3	2	1.108	0.971	0.991	0.946	0.998	0.977	0.951	3.33E-01
cds.Camellia_sinensis.comp39622_c0_seq1_m.30228	PREDICTED: electron transfer flavoprotein subunit alpha, mitochondrial [Juglans regia]	37.073	56.795	29.5	8	10	8	1.004	1.044	1.014	0.96	0.974	1.006	0.96	8.81E-02
cds.Camellia_sinensis.comp39943_c0_seq1_m.53264	PREDICTED: sorting nexin 2A [Vitis vinifera]	17.431	1.8136	7.7	1	1	1								

cds.Camellia_sinensis.comp39971_c0_seq1_m.51236	PREDICTED: glycine-rich RNA-binding protein RZ1C isoform X1 [Fragaria vesca subsp. vesca]	12.197	2.0356	7.6	1	1	1	1.315	1.349	1.269	0.589	0.705	0.673	0.5	2.57E-04
cds.Camellia_sinensis.comp401276_c0_seq1_m.59258	PREDICTED: beta-glucosidase 12-like [Jatropha curcas]	10.919	14.908	20	2	3	2	1.056	1.011	1.061	0.966	0.984	0.919	0.917	2.75E-02
cds.Camellia_sinensis.comp40162_c0_seq1_m.14618	PREDICTED: aldehyde dehydrogenase family 3 member H1 isoform X1 [Vitis vinifera]	60.979	38.077	14.3	8	10	7	1.046	1.072	1.033	0.958	0.951	0.932	0.902	1.62E-03
cds.Camellia_sinensis.comp40232_c0_seq1_m.33656	methyltransferase type 11 [Medicago truncatula]	33.591	14.827	18	5	5	5	1	1.021	1.025	0.991	0.968	0.987	0.967	3.40E-02

cds.Camellia_sinensis.comp40300_c0_seq1_m.49352	Tetratricopeptide repeat (TPR)-like superfamily protein [Arabidopsis thaliana]	18.842	-2	4.2	1	2	1	0.752	1.032	1.266	1.178	0.958	0.863	0.983	9.88E-01
cds.Camellia_sinensis.comp40740_c0_seq1_m.48580	PREDICTED: kxDL motif-containing protein 1-like [Juglans regia]	15.205	6.0489	9	1	1	1	0.946	0.876	1.056	1.028	1.084	1.037	1.094	1.77E-01
cds.Camellia_sinensis.comp40827_c0_seq1_m.36639	PREDICTED: thaumatin-like protein [Vitis vinifera]	29.404	8.7214	9	2	2	2	1.067	1.067	1.015	0.971	0.933	0.939	0.903	7.92E-03
cds.Camellia_sinensis.comp41274_c0_seq1_m.27135	PREDICTED: omega-hydroxypalmitate O-feruloyl transferase [Vitis vinifera]	50.195	15.876	13.3	6	10	6	1.296	1.24	1.31	0.678	0.725	0.652	0.534	5.93E-05

cds.Camellia_sinensis.comp41282_c0_seq1_m.25530	PREDICTED: probable acyl-activating enzyme 1, peroxisomal isoform X1 [Vitis vinifera]	52.393	1.1559	1.9	1	1	1	0.945	0.963	0.951	1.076	1.082	1.011	1.108	1.03E-02
cds.Camellia_sinensis.comp41324_c0_seq1_m.36863	Acid phosphatase 1 [Morus notabilis]	29.104	12.726	15.6	3	4	3	0.974	1.012	0.934	1.163	0.997	0.945	1.063	4.31E-01
cds.Camellia_sinensis.comp41457_c0_seq1_m.43683	PREDICTED: germin-like protein 11-1 [Vitis vinifera]	26.058	6.9438	13.7	3	3	3	1.088	1.097	1.005	0.938	0.93	0.927	0.876	4.29E-02
cds.Camellia_sinensis.comp414583_c0_seq1_m.57860	PREDICTED: galactinol synthase 2-like [Citrus sinensis]	13.069	4.5376	19.8	2	3	2	0.952	0.929	0.777	1.122	1.075	1.181	1.271	2.50E-02
cds.Camellia_sinensis.comp42092_c0_seq1_m.36103	transmembrane protein [Arabidopsis thaliana]	33.844	6.4069	9.5	3	4	3	0.915	1.086	0.981	1.051	1.023	0.966	1.019	7.23E-01

cds.Camellia_sinensis.comp421310_c0_seq1_m.58720	PREDICTED: subtilisin-like protease SBT1.7 [Vitis vinifera]	11.108	2.5143	16.3	2	3	2	0.892	0.951	0.96	1.028	1.11	1.093	1.153	1.25E-02
cds.Camellia_sinensis.comp42486_c0_seq1_m.42101	PREDICTED: calponin homology domain-containing protein DDB_G0272472 isoform X2 [Populus euphratica]	28.725	6.6258	16.6	4	4	3								
cds.Camellia_sinensis.comp42499_c0_seq1_m.34886	transmembrane protein, putative [Medicago truncatula]	37.405	4.2894	7.6	3	5	3	1.014	1.002	0.989	1.034	1.009	0.963	1	9.98E-01
cds.Camellia_sinensis.comp42843_c0_seq1_m.45117	PREDICTED: protein RETICULATA-RELATED 1, chloroplastic-like [Nicotiana tabacum]	21.168	21.174	17.5	3	4	3	0.954	1.01	1.015	0.991	0.969	1.046	1.009	7.83E-01

cds.Camellia_sinensis.comp42942_c0_seq1_m.42302	PREDICTED: protein FAM136A [Vitis vinifera]	20.553	10.436	20	3	3	3	1.022	0.956	0.962	1.019	1.089	0.965	1.045	3.48E-01
cds.Camellia_sinensis.comp43060_c0_seq1_m.14223	PREDICTED: ATPase family AAA domain-containing protein 3C-like [Nicotiana tabacum]	69.453	16.508	11.8	9	10	8	0.959	0.962	0.945	1.028	0.994	1.046	1.07	1.31E-02
cds.Camellia_sinensis.comp43546_c0_seq1_m.36228	PREDICTED: BTB/POZ and MATH domain-containing protein 3-like isoform X1 [Vigna radiata var. radiata]	35.6	1.9894	2.9	1	1	1								

cds.Camellia_sinensis.comp4412_c0_seq1_m.47742	PREDICTED: dammarenediol II synthase-like [Daucus carota subsp. sativus]	25.26	-2	6	1	1	1								
cds.Camellia_sinensis.comp44218_c0_seq1_m.46187	SNARE-associated family protein [Populus trichocarpa]	17.607	6.242	9.9	1	1	1		1.046	1.009	1.069		0.953	0.984	7.97E-01
cds.Camellia_sinensis.comp45076_c0_seq1_m.42729	PREDICTED: acetylornithine aminotransferase, mitochondrial [Theobroma cacao]	22.025	1.6269	16.3	3	4	2	0.974	1.12	1.013	0.942	0.993	0.96	0.932	1.94E-01
cds.Camellia_sinensis.comp45314_c0_seq1_m.27017	PREDICTED: DUF21 domain-containing protein At4g14240 [Vitis vinifera]	45.073	4.306	7.2	2	2	1								

cds.Camellia_sinensis.comp45372_c0_seq1_m.42279	PREDICTED: CCG-binding protein 1 [Juglans regia]	24.55	2.6584	10.2	2	2	2	0.865	0.993	0.944	1.041	1.047	1.145	1.154	4.72E-02
cds.Camellia_sinensis.comp4543_c0_seq1_m.35900	PREDICTED: ricin-like [Elaeis guineensis	36.078	50.212	6.5	2	4	2	0.848	0.856	0.848	1.113	1.174	1.204	1.368	5.54E-03
cds.Camellia_sinensis.comp45686_c0_seq1_m.44193	PREDICTED: homeobox protein BEL1 homolog [Juglans regia]	22.13	1.7117	5.3	1	1	1	1.095	1	1.01	0.937	0.954	0.993	0.929	9.37E-02
cds.Camellia_sinensis.comp45943_c0_seq1_m.50866	PREDICTED: endo-1,3;1,4-beta-D-glucanase-like [Juglans regia]	19.354	28.42	22.7	4	4	4	1.014	0.948	1.1	0.973	0.967	0.998	0.96	4.20E-01
cds.Camellia_sinensis.comp46107_c0_seq1_m.13891	PREDICTED: putative BPI/LBP family protein At1g04970 isoform X1 [Nelumbo nucifera]	53.95	40.69	13.5	6	7	6	1.071	1.02	1.033	0.949	0.937	0.963	0.912	5.20E-03

cds.Camellia_sinensis.comp46125_c0_seq1_m.54741		11.837	-2	7.5	1	1	1								
cds.Camellia_sinensis.comp46723_c0_seq1_m.58684	PREDICTED: plasma membrane Ca2+-ATPase isoform X1 [Glycine max]	11.241	-2	18.1	2	3	1	0.937	0.935	0.986	1.044	1.021	1.102	1.108	2.30E-02
cds.Camellia_sinensis.comp47152_c0_seq1_m.29533	alpha/beta hydrolase family protein [Medicago truncatula]	40.689	52.657	35.6	10	15	10	1.017	1.04	1.013	0.967	0.957	0.977	0.945	5.04E-03
cds.Camellia_sinensis.comp47520_c0_seq1_m.36626	PREDICTED: probable sarcosine oxidase [Vitis vinifera]	39.465	3.6348	4	1	1	1	1.137	1.074	1.18	1.099	0.677	0.812	0.763	1.14E-01
cds.Camellia_sinensis.comp47551_c0_seq1_m.50882		14.053	7.1958	19.2	3	4	3	1.016	0.969	1.031	0.96	1.028	0.996	0.989	7.14E-01

cds.Camellia_sinensis.comp47557_c0_seq1_m.46952	PREDICTED: phosphoenolpyruvate carboxylase [Capsicum annuum]	16.262	-2	30.3	4	6	0								
cds.Camellia_sinensis.comp48384_c0_seq1_m.47721	PREDICTED: acetylornithine aminotransferase, mitochondrial [Populus euphratica]	15.282	9.8073	12.8	2	3	2	1.058	1.079	0.991	0.919	0.979	0.928	0.903	3.52E-02
cds.Camellia_sinensis.comp48392_c0_seq1_m.51039	PREDICTED: gibberellin-regulated protein 1-like isoform X2 [Nicotiana sylvestris]	12.635	3.2003	18.1	2	3	2	1.053	1.023	1.001	1.062	0.983	0.993	0.987	6.67E-01
cds.Camellia_sinensis.comp492684_c0_seq1_m.58839	PREDICTED: translationally-controlled tumor protein homolog [Gossypium hirsutum]	11.776	11.42	38.5	6	16	3	1.012	0.95	1.018	0.997	1.03	1.001	1.016	5.39E-01

cds.Camellia_sinensis.comp49969_c0_seq1_m.40407	PREDICTED: heat shock 70 kDa protein [Nelumbo nucifera]	33.991	1.8236	20.1	7	22	1								
cds.Camellia_sinensis.comp51268_c0_seq1_m.48530	PREDICTED: clathrin heavy chain 1-like [Oryza brachyantha]	20.307	16.54	18.9	2	3	1	1.01	0.912	0.919	1.032	1.043	1.103	1.119	4.38E-02
cds.Camellia_sinensis.comp52650_c0_seq1_m.46280	PREDICTED: probable calcium-binding protein CML29 [Theobroma cacao]	17.113	40.119	10.1	1	4	1	0.888	1.064	1.105	1.036	0.941	0.973	0.965	6.85E-01
cds.Camellia_sinensis.comp53351_c0_seq1_m.53378	PREDICTED: serine carboxypeptidase-like [Erythranthe guttata]	17.209	1.7211	5.2	1	1	1	1.168	1.141	1.092	0.89	0.828	0.843	0.753	6.39E-04
cds.Camellia_sinensis.comp53621_c0_seq1_m.37575	PREDICTED: cannabidiolic acid synthase-like 1 [Citrus sinensis]	34.518	5.1177	10.8	3	3	3	0.623	0.613	0.553	1.402	1.527	1.424	2.433	4.04E-05

cds.Camellia_sinensis.comp53961_c0_seq1_m.41267	PREDICTED: protein PLASTID REDOX INSENSITIVE 2 [Nicotiana attenuata]	23.337	4.1696	12.2	2	2	2	0.941	0.941	0.975	1.049	1.019	1.101	1.109	1.55E-02
cds.Camellia_sinensis.comp54424_c0_seq1_m.57834	PREDICTED: nucleoporin NUP152-like [Juglans	11.825	2.1357	8.9	1	1	1								
cds.Camellia_sinensis.comp55332_c0_seq1_m.45185		26.423	4.4742	7	1	1	1								
cds.Camellia_sinensis.comp56153_c0_seq1_m.55980		11.726	16.486	11	2	3	2	1.016	0.932	0.906	1.083	1.095	1.064	1.136	2.22E-02
cds.Camellia_sinensis.comp57239_c0_seq1_m.48520	PREDICTED: shikimate O-hydroxycinnamoyltransferase [Citrus sinensis]	15.275	3.059	8.8	1	1	1	1.064	1.051	1.022	0.953	0.966	0.936	0.91	3.30E-03

cds.Camellia_sinensis.comp57924_c0_seq1_m.44234	Aldolase-type TIM barrel family protein [Arabidopsis thaliana]	22.123	120.89	36.9	6	13	6	0.935	0.984	0.968	1.008	1.065	1.08	1.092	2.74E-02
cds.Camellia_sinensis.comp59003_c0_seq1_m.33995	PREDICTED: protein SCO1 homolog 2, mitochondrial isoform X2	31.166	1.7285	5.8	2	2	2	1.011	1.045	1.013	0.986	1	0.949	0.956	7.75E-02
cds.Camellia_sinensis.comp59169_c0_seq1_m.58685	PREDICTED: cysteine-rich repeat secretory protein 60-like [Populus euphratica]	10.862	3.2851	13.3	1	1	1	0.915	0.945	0.92	1.034	1.094	1.123	1.169	4.06E-03
cds.Camellia_sinensis.comp6175_c0_seq1_m.49015	PREDICTED: protein ASPARTIC PROTEASE IN GUARD CELL 2 [Citrus sinensis]	19.549	5.8724	14	2	3	2	1.061	1.041	1.059	1.077	0.949	0.86	0.913	2.83E-01

cds.Camellia_sinensis.comp63389_c0_seq1_m.43336	putative mannitol dehydrogenase [Morus notabilis]	27.064	47.983	28.6	7	12	7	0.841	0.852	0.832	1.159	1.155	1.19	1.388	4.78E-06
cds.Camellia_sinensis.comp66358_c0_seq1_m.30735	PREDICTED: CLIP-associated protein-like [Nicotiana attenuata]	37.671	1.1765	2.5	1	1	1	0.883	0.842	0.972	1.114	1.112	1.123	1.242	3.56E-02
cds.Camellia_sinensis.comp66891_c0_seq1_m.58005	PREDICTED: BEL1-like homeodomain protein 9 [Vitis vinifera]	12.246	1.5614	7.3	1	1	1	1.034	1.004	1.068	0.974	0.908	1.009	0.931	1.13E-01
cds.Camellia_sinensis.comp6690_c0_seq1_m.53308	PREDICTED: golgin subfamily A member 1 isoform X1 [Theobroma cacao]	18.257	-2	5.8	1	1	1	1.079	0.971	1.026	0.941	1.008	0.97	0.949	2.27E-01
cds.Camellia_sinensis.comp67036_c0_seq1_m.38292	PREDICTED: reticuline oxidase-like protein [Eucalyptus grandis]	36.396	5.7183	6.9	2	6	1	0.921	0.924	0.929	1.074	1.06	1.124	1.174	1.23E-02

cds.Camellia_sinensis.comp67181_c0_seq1_m.57359	PREDICTED: zeatin O-xylosyltransferase-like [Nicotiana attenuata]	11.895	12.544	8.2	1	1	1	1.09	1.072	1.005	0.952	0.947	0.921	0.89	1.25E-02
cds.Camellia_sinensis.comp67295_c0_seq1_m.36148	PREDICTED: peroxisomal 2,4-dienoyl-CoA reductase [Vitis vinifera]	33.097	1.2639	2.6	1	1	1	0.949	0.995	0.957	1.018	1.041	1.06	1.075	1.78E-02
cds.Camellia_sinensis.comp69681_c0_seq1_m.38233	PREDICTED: vestitone reductase-like [Vitis vinifera]	37.026	3.26	8.7	3	3	3	0.884	0.869	0.893	1.15	1.176	1.082	1.288	6.41E-04
cds.Camellia_sinensis.comp7043_c0_seq1_m.43597	PREDICTED: probable bifunctional methylthioribulose-1-phosphate dehydratase/enolase-phosphatase E1 2 isoform X1 [Vitis vinifera]	19.226	1.5844	4.1	1	3	1	0.96	0.909	0.979	1.021	1.045	1.068	1.1	2.02E-02

cds.Camellia_sinensis.comp7156_c0_seq1_m.36164	PREDICTED: tryptophan aminotransferase-related protein 2 [Vitis vinifera]	36.233	1.5395	2.8	1	1	1	0.444	1.522	1.425	1.148	0.853	0.667	0.787	7.78E-01
cds.Camellia_sinensis.comp72738_c0_seq1_m.34175	PREDICTED: hexose carrier protein HEX6-like [Gossypium raimondii]	40.021	2.3306	3.7	1	1	1								
cds.Camellia_sinensis.comp7306_c0_seq1_m.21311	PREDICTED: pectinesterase-like [Gossypium hirsutum]	56.944	99.605	20.4	8	22	8	1.19	1.181	1.217	0.809	0.786	0.78	0.662	4.30E-06
cds.Camellia_sinensis.comp7329_c0_seq1_m.36717	PREDICTED: clathrin heavy chain 1-like, partial [Nicotiana tabacum]	38.859	7.1565	25.2	10	13	2	1.047	0.982	0.918	1.015	0.956	1.086	1.037	5.26E-01

cds.Camellia_sinensis.comp7370_c0_seq1_m.2142	PREDICTED: sister chromatid cohesion 1 protein 4 [Vitis vinifera]	146.01	2.1265	1.3	2	2	2	1.001	1.031	1.138	1.048	0.849	0.935	0.893	1.86E-01
cds.Camellia_sinensis.comp7398_c0_seq1_m.51903	PREDICTED: aldehyde dehydrogenase family 2 member C4 [Ricinus communis]	19.086	6.2598	17.6	3	3	2	1.043	0.977	0.989	0.952	1.035	1.006	0.995	8.70E-01
cds.Camellia_sinensis.comp7414_c0_seq1_m.23149	PREDICTED: endoplasmic reticulum-Golgi intermediate compartment protein 3-like [Nelumbo nucifera]	43.668	3.4285	9.1	5	5	5	0.965	0.964	0.964	1.055	1.019	1.055	1.082	2.12E-02
cds.Camellia_sinensis.comp7442_c0_seq1_m.31191	PREDICTED: protein DETOXIFICATION 27-like [Ziziphus jujuba]	44.466	2.5815	3.2	1	1	1								

cds.Camellia_sinensis.comp7457_c0_seq1_m.29704	PREDICTED: glucan endo-1,3-beta-glucosidase 5 [Sesamum	48.901	3.5188	4.6	2	2	1								
cds.Camellia_sinensis.comp7459_c0_seq1_m.59126	PREDICTED: protein transport protein SEC23-like [Nicotiana tabacum]	10.585	34.392	27.5	3	4	3	0.957	0.956	0.923	1.195	1.005	1	1.128	1.21E-01
cds.Camellia_sinensis.comp74698_c0_seq1_m.45845	PREDICTED: cysteine-rich repeat secretory protein 55-like [Gossypium hirsutum]	26.69	7.2489	12.1	2	2	2	1.047	1.295	1.01	0.964	0.734	0.925	0.783	9.90E-02
cds.Camellia_sinensis.comp7481_c0_seq1_m.25260	PREDICTED: protein disulfide-isomerase [Nelumbo nucifera]	48.994	1.2802	4.1	3	4	1	1.015	0.926	0.952	0.888	1.169	1.06	1.077	4.62E-01

cds.Camellia_sinensis.comp7486_c0_seq1_m.13209	PREDICTED: ferrochelatase-2, chloroplastic [Vitis vinifera]	57.912	13.033	7.5	4	6	4	1.054	1.074	0.994	0.927	0.945	0.942	0.901	1.27E-02
cds.Camellia_sinensis.comp74925_c0_seq1_m.19313	PREDICTED: probable acyl-activating enzyme 2 [Juglans regia]	65.575	12.883	8.1	4	5	4	0.981	1.04	0.985	1.071	0.927	1.014	1.002	9.90E-01
cds.Camellia_sinensis.comp7507_c0_seq1_m.52448	PREDICTED: phospholipase D alpha 1-like [Juglans regia]	18.715	14.699	19.5	3	4	2	0.947	0.884	0.954	1.045	1.095	1.105	1.165	6.80E-03
cds.Camellia_sinensis.comp7540_c0_seq1_m.42081	PREDICTED: acylphosphatase-like isoform X1 [Nicotiana tabacum]	25.436	6.4602	8.8	2	4	2	0.926	0.967	0.951	1.035	1.038	1.047	1.097	2.08E-03
cds.Camellia_sinensis.comp7544_c0_seq2_m.47755	PREDICTED: calcineurin subunit B-like [Nicotiana tabacum]	21.778	6.1985	9.8	2	3	1	0.918	0.949	0.873	1.094	1.142	1.062	1.204	4.48E-03

cds.Camellia_sinensis.comp7575_c0_seq1_m.43805	plant/MOJ 10-14 protein [Medicago truncatula]	21.256	11.112	16.2	3	3	3	0.969	1.05	1.002	1.028	1.008	0.958	0.991	7.91E-01
cds.Camellia_sinensis.comp7627_c0_seq1_m.12093	PREDICTED: root phototropism protein 3 [Citrus sinensis]	73.688	10.098	7.8	4	5	4	1.052	0.898	1.144	1.031	0.947	0.932	0.941	5.01E-01
cds.Camellia_sinensis.comp7655_c0_seq2_m.28124	PREDICTED: 50S ribosomal protein L15 isoform X1 [Daucus carota subsp. sativus]	30.729	5.6863	3.9	1	1	1								
cds.Camellia_sinensis.comp7756_c0_seq1_m.19701	PREDICTED: probable 2-carboxy-D-arabinitol-1-phosphatase [Theobroma cacao]	59.893	191.09	29.8	15	35	15	0.981	0.981	0.999	1.016	1.012	1.007	1.025	1.99E-02
cds.Camellia_sinensis.comp7765_c0_seq1_m.49699	PREDICTED: tubulin alpha-3 chain-like [Malus domestica]	15.45	7.7989	44.9	5	9	2	1.1	1.09	1.325	0.765	0.856	0.823	0.695	7.32E-03

cds.Camellia_sinensis.comp7790_c0_seq1_m.36728	embryo-abundant family protein [Populus trichocarpa]	30.556	89.831	42.2	11	21	11	1.032	0.959	0.985	0.971	1.06	0.996	1.017	6.45E-01
cds.Camellia_sinensis.comp7843_c0_seq1_m.37966	PREDICTED: isoleucine--tRNA ligase, cytoplasmic-like [Nicotiana tabacum]	23.995	2.5024	9.7	1	2	1								
cds.Camellia_sinensis.comp7873_c0_seq1_m.33440	PREDICTED: exosome complex component RRP42 [Ziziphus jujuba]	29.914	3.5615	9.1	2	2	2								
cds.Camellia_sinensis.comp7911_c0_seq1_m.48286	PREDICTED: HVA22-like protein f [Daucus carota subsp. sativus]	18.385	10.847	18.8	3	3	3	0.943	0.935	0.947	1.089	1.064	1.035	1.128	1.36E-03
cds.Camellia_sinensis.comp7916_c0_seq2_m.4146	LETM1-like protein [Medicago truncatula]	103.16	3.8131	2.2	2	2	2	0.992	1.1	1.041	0.962	0.918	0.985	0.914	7.05E-02

cds.Camellia_sinensis.comp7948_c0_seq1_m.50183	PREDICTED: protein SPA, chloroplastic isoform X1 [Citrus sinensis]	12.787	5.1659	12.3	1	2	1	1.006	1.028	1.063	1.03	0.898	0.98	0.939	2.11E-01
cds.Camellia_sinensis.comp7974_c0_seq1_m.52825	PREDICTED: coatomer subunit zeta-1-like [Juglans regia]	18.477	9.455	14.5	3	6	3	1.053	1.014	0.983	0.992	1.023	0.937	0.968	3.69E-01
cds.Camellia_sinensis.comp7976_c0_seq1_m.40429	PREDICTED: thioredoxin M3, chloroplastic-like [Ipomoea nil]	19.762	39.591	28.8	4	5	4	0.98	1.046	1.011	1.005	0.998	0.97	0.979	3.87E-01
cds.Camellia_sinensis.comp7984_c0_seq1_m.25603	PREDICTED: maspardin [Theobroma cacao]	46.59	1.7325	2.9	1	1	1								
cds.Camellia_sinensis.comp8031_c0_seq1_m.49811	PREDICTED: alcohol dehydrogenase class-3-like isoform X2 [Capsicum annuum]	13.113	2.2441	18.5	2	2	2	1.036	0.965	1.075	0.927	0.999	0.996	0.95	2.72E-01

cds.Camellia_sinensis.comp8059_c0_seq1_m.38917		19.822	1.7826	6.5	1	1	1								
cds.Camellia_sinensis.comp8060_c0_seq2_m.27832	PREDICTED: ferredoxin--NADP reductase, root isozyme, chloroplastic [Cicer arietinum]	42.626	44.229	27.2	10	15	10	1.025	1.004	0.981	0.995	0.99	1.004	0.993	6.34E-01
cds.Camellia_sinensis.comp80684_c0_seq1_m.42619	PREDICTED: bifunctional epoxide hydrolase 2-like [Erythranthe guttata]	31.406	4.6848	10.5	2	2	2	0.9	0.998	0.839	1.094	1.016	1.187	1.205	5.06E-02
cds.Camellia_sinensis.comp80707_c0_seq1_m.24397	transferring glycosyl group transferase [Medicago truncatula]	53.175	1.1743	1.9	1	1	1	1.024	1.069	1.004	0.985	0.963	0.955	0.937	3.58E-02
cds.Camellia_sinensis.comp80858_c0_seq1_m.39939	PREDICTED: phosphomannomutase/phosphoglucomutase isoform X2 [Ziziphus jujuba]	34.189	37.016	19	6	7	6	1.017	1.052	1.057	0.973	0.925	0.969	0.917	1.29E-02

cds.Camellia_sinensis.comp8105_c0_seq1_m.8660	PREDICTED: copper amine oxidase 1-like isoform X2 [Populus euphratica]	26.139	2.1693	4.3	1	1	1								
cds.Camellia_sinensis.comp81312_c0_seq1_m.23202	PREDICTED: aspartic proteinase Asp1 isoform X3 [Fragaria vesca subsp. vesca]	51.099	2.1335	2.8	1	1	1	0.957	1.379	0.937	1.033	0.837	0.851	0.831	2.91E-01
cds.Camellia_sinensis.comp81846_c0_seq1_m.59305	PREDICTED: pectinesterase 2.2-like [Juglans regia]	11.322	11.57	27.7	2	2	2	0.954	0.967	0.995	1.119	0.954	1.036	1.066	2.63E-01

cds.Camellia_sinensis.comp8216_c0_seq1_m.31089	PREDICTED: biotin carboxyl carrier protein of acetyl-CoA carboxylase, chloroplastic-like [Gossypium raimondii]	29.807	7.1227	13.1	4	10	4	1.037	1.057	1.008	0.986	0.974	1.005	0.956	5.27E-02
cds.Camellia_sinensis.comp8275_c0_seq1_m.49996	PREDICTED: protein lava lamp isoform X5 [Vitis vinifera]	21.74	2.7562	11	2	2	2	1.087	1.008	1.098	1.018	0.854	0.926	0.876	8.03E-02
cds.Camellia_sinensis.comp8320_c0_seq1_m.49805	PREDICTED: SPX domain-containing protein 1-like [Jatropha curcas]	22.306	1.203	7.3	1	1	1								
cds.Camellia_sinensis.comp8340_c0_seq1_m.48112	PREDICTED: mitochondrial phosphate carrier protein 3, mitochondrial [Vitis vinifera]	14.185	7.2687	16.5	2	4	1	0.982	0.899	0.882	0.979	1.09	1.188	1.179	6.68E-02

cds.Camellia_sinensis.comp8370_c0_seq1_m.39819	PREDICTED: probable glutathione S-transferase [Vitis vinifera]	26.244	45.45	39.2	8	13	8	0.918	0.892	0.888	1.128	1.139	1.046	1.228	2.08E-03
cds.Camellia_sinensis.comp8436_c0_seq1_m.50373	PREDICTED: pectin acetylesterase 10 [Nelumbo nucifera]	20.935	6.1345	6.4	1	1	1	1	1.156	1.139	0.951	0.847	0.897	0.818	2.46E-02
cds.Camellia_sinensis.comp8468_c0_seq1_m.37024	glyoxalase/bleomycin resistance protein/dioxygenase [Medicago truncatula]	22.623	15.368	22.7	4	6	4	1.039	1.063	1.022	0.952	0.953	0.988	0.926	1.00E-02
cds.Camellia_sinensis.comp8505_c0_seq1_m.28563	PREDICTED: putative invertase inhibitor [Ziziphus jujuba]	21.54	2.1267	5.1	1	1	1	0.984	1.102	1.233	0.884	0.953	0.834	0.805	4.72E-02
cds.Camellia_sinensis.comp85250_c0_seq1_m.59355	phosphoenolpyruvate carboxylase-like [Malus domestica]	11.762	-2	24	3	4	1	0.848	0.975	0.967	1.14	1.114	1.004	1.168	5.98E-02
cds.Camellia_sinensis.comp8548_c0_seq1_m.30661	Protein ApaG [Morus notabilis]	21.503	7.6426	10.5	2	2	2	0.902	1.09	1.057	1.056	1.009	0.908	0.975	7.63E-01

cds.Camellia_sinensis.comp85536_c0_seq1_m.58933	stress up-regulated Nod 19 protein [Medicago truncatula]	11.674	3.4634	10.7	1	1	1									
cds.Camellia_sinensis.comp8559_c0_seq1_m.31734	transmembrane protein, putative [Medicago truncatula]	18.39	2.1378	9.6	1	1	1	0.978	0.945	0.932	1.193	0.937	1.044	1.112	2.29E-01
cds.Camellia_sinensis.comp8571_c0_seq1_m.30552	PREDICTED: protein MEMO1 [Vitis vinifera]	32.687	4.0276	6.5	2	2	2	0.928	1.013	1.025	1.022	0.991	1.041	1.03	4.33E-01
cds.Camellia_sinensis.comp8673_c0_seq1_m.10797	26S proteasome non-ATPase regulatory subunit 5 [Morus notabilis]	21.633	31.4	41.7	7	7	7	0.981	1.018	1.082	0.985	0.982	0.962	0.951	1.67E-01
cds.Camellia_sinensis.comp8726_c0_seq1_m.43781	PREDICTED: UPF0160 protein-like [Populus euphratica]	13.611	7.49	11.4	1	2	1	0.999	0.948	0.973	1.026	1.031	1.039	1.06	1.95E-02

cds.Camellia_sinensis.comp8740_c0_seq1_m.36201	SMP-30/gluconolaconase/LRE-like region protein [Medicago truncatula]	39.718	4.3274	5.9	2	2	2	1.035	0.989	0.977	1.029	0.937	1.038	1.001	9.95E-01
cds.Camellia_sinensis.comp8761_c0_seq1_m.42795	PREDICTED: 15 kDa selenoprotein-like isoform X2 [Juglans regia]	20.876	4.5007	9.8	2	2	2	1.008	1.043	0.934	1.092	0.936	0.998	1.014	8.30E-01
cds.Camellia_sinensis.comp8839_c0_seq1_m.30938	PREDICTED: mitochondrial outer membrane protein porin 4 [Juglans regia]	29.662	46.409	31.9	9	15	8	1.08	1.023	1.033	0.939	0.953	1.001	0.923	3.41E-02
cds.Camellia_sinensis.comp8848_c0_seq1_m.38628	latex abundant protein 1 [Populus trichocarpa]	25.275	3.9751	5.8	1	2	1	1.073	0.956	0.938	1.011	0.92	1.102	1.022	7.69E-01
cds.Camellia_sinensis.comp8891_c1_seq1_m.56602	tropinone reductase homolog At5g06060-like [Ananas comosus]	13.052	15.253	23	3	8	2	0.994	0.953	0.98	1.008	0.994	1.037	1.038	9.93E-02

cds.Camellia_sinensis.comp8892_c0_seq1_m.2156	PREDICTED: kinesin-like protein KIN-14B [Vitis vinifera]	142.71	312.9	36.1	41	60	41	1.048	1.038	1.032	0.959	0.961	0.962	0.924	6.36E-05
cds.Camellia_sinensis.comp8914_c0_seq1_m.15133	PREDICTED: probable RNA-binding protein 46 isoform X3 [Vitis vinifera]	62.649	67.809	25.8	13	15	13	0.993	0.964	0.977	1.025	1.002	1.028	1.041	2.66E-02
cds.Camellia_sinensis.comp8916_c0_seq1_m.36632	PREDICTED: putative 4-hydroxy-4-methyl-2-oxoglutarate aldolase 3 isoform X2 [Ricinus communis]	17.69	21.263	30.1	5	6	4	0.98	1.023	0.971	1.05	1.014	1.021	1.037	1.29E-01
cds.Camellia_sinensis.comp8921_c0_seq1_m.29053	PREDICTED: inositol-tetrakisphosphate 1-kinase 1 [Vitis vinifera]	35.903	14.876	14	4	5	4	1.09	1.037	1.02	0.946	1.01	0.897	0.907	6.74E-02

cds.Camellia_sinensis.comp8966_c0_seq1_m.22522	PREDICTED: uncharacterized hydrolase YugF-like [Ziziphus jujuba]	45.77	2.0702	3.4	1	1	1								
cds.Camellia_sinensis.comp8972_c0_seq1_m.42754	PREDICTED: cytochrome c6, chloroplastic [Vitis vinifera]	18.845	5.6472	19	4	5	4	0.915	0.958	0.97	1.06	1.083	1.042	1.12	5.60E-03
cds.Camellia_sinensis.comp90284_c0_seq1_m.38720	PREDICTED: D-3-phosphoglycerate dehydrogenase 3, chloroplastic-like [Jatropha curcas]	34.008	16.57	12	4	5	4	0.918	0.952	1.002	1.012	1.063	1.119	1.112	5.13E-02
cds.Camellia_sinensis.comp9057_c0_seq1_m.42253	PREDICTED: purple acid phosphatase [Eucalyptus grandis]	32.811	4.3972	3.9	1	1	1	1.029	1.138	0.984	0.965	0.966	0.913	0.903	9.74E-02
cds.Camellia_sinensis.comp9149_c0_seq1_m.50925	PREDICTED: basic blue protein [Arachis ipaensis]	12.843	2.3989	10.6	1	1	1								

cds.Camellia_sinensis.comp9166_c0_seq1_m.26212	PREDICTED: protein SRG1 [Theobroma cacao]	43.278	95.806	28.3	10	16	10	0.96	0.965	0.991	1.044	1.09	1.046	1.091	7.26E-03
cds.Camellia_sinensis.comp9179_c0_seq1_m.26764	PREDICTED: protein DJ-1 homolog D [Musa acuminata subsp. malaccensis]	43.787	59.101	16.7	6	17	6	0.925	0.947	0.939	1.084	1.036	1.055	1.129	1.20E-03
cds.Camellia_sinensis.comp9186_c0_seq1_m.31693	PREDICTED: peroxisomal trans-2-enoyl-CoA reductase [Sesamum indicum]	29.245	4.4668	3.8	1	2	1	0.896	1.47	0.922	0.901	0.882	0.918	0.821	4.02E-01
cds.Camellia_sinensis.comp9249_c0_seq1_m.13569	PREDICTED: cysteine--tRNA ligase, chloroplastic/mitochondrial isoform X2 [Vitis vinifera]	53.618	7.5102	5.7	3	5	3	0.966	1.013	1.01	0.984	0.972	1.069	1.012	7.53E-01

cds.Camellia_sinensis.comp9258_c0_seq1_m.13017	PREDICTED: MICOS complex subunit MIC60 [Nelumbo nucifera]	74.337	109.42	30.2	18	25	18	1.017	0.999	0.991	1.026	0.998	0.985	1.001	9.70E-01
cds.Camellia_sinensis.comp9365_c0_seq1_m.15477	PREDICTED: vacuolar protein sorting-associated protein 45 homolog [Theobroma cacao]	64.82	2.6308	2.1	1	1	1								
cds.Camellia_sinensis.comp93901_c0_seq1_m.56272	PREDICTED: 1-aminocyclopropane-1-carboxylate oxidase homolog 1-like [Jatropha curcas]	13.59	4.3057	8.2	1	1	1	1.086	0.986	1.016	0.942	0.907	1.052	0.939	2.95E-01
cds.Camellia_sinensis.comp9404_c0_seq1_m.46471	DUF1680 domain protein [Medicago truncatula]	14.004	9.3763	16.3	1	1	1								

cds.Camellia_sinensis.comp9406_c0_seq1_m.55002	PREDICTED: protein ABCI7, chloroplastic-like isoform X2 [Ipomoea nil]	14.823	13.542	24.1	3	6	3	1.076	1.043	0.992	0.94	0.96	1.009	0.935	1.02E-01
cds.Camellia_sinensis.comp9428_c0_seq1_m.53920	PREDICTED: calcium-transporting ATPase 10, plasma membrane-type [Erythranthe guttata]	16.319	-2	7.4	1	1	1	1.048	1.074	1.049	0.938	0.905	0.975	0.889	6.70E-03
cds.Camellia_sinensis.comp9438_c0_seq1_m.15474	PREDICTED: aldehyde dehydrogenase family 7 member A1 [Nelumbo nucifera]	56.082	32.538	18.3	9	15	9	0.973	0.998	0.993	0.996	1.018	1.027	1.026	9.89E-02
cds.Camellia_sinensis.comp9443_c0_seq1_m.43794	PREDICTED: dnaJ homolog 1, mitochondrial-like [Sesamum	19.311	4.4	28.7	6	14	1	1.034	1.032	1.065	0.91	0.948	1.003	0.914	3.88E-02

cds.Camellia_sinensis.comp9451_c0_seq1_m.34591	PREDICTED: pyrroline-5-carboxylate reductase [Erythranthe guttata]	28.647	1.7665	9.7	3	3	3	0.963	1.054	0.98	1.036	0.986	0.996	1.007	8.23E-01
cds.Camellia_sinensis.comp9469_c0_seq1_m.45143	PREDICTED: protein TIC 55, chloroplastic [Solanum tuberosum	17.791	2.1189	5.8	1	1	1	0.968	1.126	1.06	0.906	1.004	0.935	0.902	1.29E-01
cds.Camellia_sinensis.comp946_c0_seq1_m.50226	PREDICTED: carboxylesterase 1-like [Juglans regia]	20.971	2.2902	6.4	1	1	1	0.982	0.994	0.879	1.008	1.137	1.021	1.109	1.31E-01
cds.Camellia_sinensis.comp9477_c0_seq1_m.27406	PREDICTED: phosphoribosylaminoimidazole-succinocarboxamide synthase, chloroplastic [Ricinus	45.922	33.978	27.1	10	13	10	0.991	0.974	1.005	0.957	1.033	1.012	1.011	6.97E-01

cds.Camellia_sinensis.comp9536_c0_seq2_m.35214	Nuclear transport factor 2 (NTF2) family protein [Arabidopsis thaliana]	29.351	22.84	21.5	5	7	5	1.002	1.033	1.041	0.996	0.999	0.968	0.963	7.20E-02
cds.Camellia_sinensis.comp9540_c0_seq1_m.46800	PREDICTED: small nuclear ribonucleoprotein E-like [Populus euphratica]	14.937	3.0596	9.4	1	3	1								
cds.Camellia_sinensis.comp9560_c0_seq1_m.38151	PREDICTED: UPF0678 fatty acid-binding protein-like protein At1g79260 [Vitis vinifera]	20.389	7.7577	10.3	2	3	2	0.973	0.943	0.942	1.022	1.047	1.093	1.106	1.07E-02
cds.Camellia_sinensis.comp9565_c0_seq1_m.53859	PREDICTED: protein ROOT HAIR DEFECTIVE 3-like [Malus domestica]	16.407	7.8304	20.3	2	2	2								

cds.Camellia_sinensis.comp9596_c0_seq1_m.33351	PREDICTED: probable enoyl-CoA hydratase 1, peroxisomal [Juglans regia]	29.042	25.078	28.6	7	9	7	0.948	0.987	0.973	1.044	1.045	1.076	1.088	5.34E-03
cds.Camellia_sinensis.comp9641_c0_seq1_m.41860	PREDICTED: cellulose synthase-like protein E6, partial [Vitis vinifera]	33.443	2.0846	7.4	2	2	2	1.038	1.123	0.97	0.866	1.003	0.988	0.912	2.18E-01
cds.Camellia_sinensis.comp9643_c0_seq1_m.39066	PREDICTED: protein DCL, chloroplastic [Vitis vinifera]	24.25	4.4331	4.8	1	1	1	1.07	0.992	1.016	0.881	1.041	0.992	0.947	3.56E-01
cds.Camellia_sinensis.comp9669_c0_seq1_m.35880	PREDICTED: PITH domain-containing protein 1 [Juglans regia]	22.44	28.824	17.6	3	4	3	0.941	1.015	1.007	0.997	1.064	0.996	1.032	3.89E-01

cds.Camellia_sinensis.comp9690_c0_seq1_m.30320	PREDICTED: acetolactate synthase small subunit 2, chloroplastic [Vitis vinifera]	35.374	14.053	15.2	5	6	5	0.93	0.971	0.909	1.093	1.052	1.076	1.146	3.58E-03
cds.Camellia_sinensis.comp9719_c0_seq1_m.15955	PREDICTED: translation initiation factor eIF-2B subunit gamma [Nelumbo nucifera]	49.73	13.029	11	4	6	4	0.913	1.029	0.999	1.027	0.987	1.082	1.053	3.10E-01
cds.Camellia_sinensis.comp9790_c0_seq1_m.23760	PREDICTED: acyl-CoA thioesterase 2-like isoform X1 [Nicotiana tabacum]	48.149	9.933	9.8	4	6	4	1.025	1.049	1.019	1.008	0.964	0.992	0.958	5.37E-02
cds.Camellia_sinensis.comp9808_c0_seq2_m.22340	PREDICTED: serine carboxypeptidase-like 45 isoform X1 [Theobroma cacao]	55.567	10.927	9.1	4	5	4	0.922	0.964	0.955	1.051	1.055	1.04	1.107	1.98E-03

cds.Camellia_sinensis.comp9835_c0_seq1_m.44238	PREDICTED: cytochrome P450 CYP72A219-like [Nicotiana tabacum]	29.72	8.2084	24.5	7	8	6	1.066	1.09	1.042	1.019	0.911	0.883	0.88	4.57E-02
cds.Camellia_sinensis.comp9846_c0_seq1_m.9208	PREDICTED: MAR-binding filament-like protein 1-1 isoform X1 [Fragaria vesca subsp. vesca]	66.879	60.388	32	18	20	18	1.057	1.066	1.078	0.9	0.938	0.927	0.864	4.18E-04
cds.Camellia_sinensis.comp98529_c0_seq1_m.53511	PREDICTED: glycerophosphodiester phosphodiesterase GDPD1, chloroplastic [Vitis vinifera]	13.38	2.6605	8.1	1	1	1	1.015	1.2	1.033	0.908	0.901	0.93	0.843	3.60E-02
cds.Camellia_sinensis.comp9879_c0_seq1_m.20633	PREDICTED: monothiol glutaredoxin-S17 isoform X1 [Vitis vinifera]	54.981	48.971	14.6	6	8	6	0.97	1.01	1.035	1.043	0.94	1.008	0.992	8.23E-01

cds.Camellia_sinensis.comp9908_c0_seq1_m.26455	PREDICTED: probable polygalacturonase [Eucalyptus grandis]	26.271	3.3251	12.3	2	2	2								
cds.Camellia_sinensis.comp9913_c0_seq1_m.57107	PREDICTED: pre-mRNA-processing factor 19-like, partial [Nicotiana tomentosiformis]	12.677	2.9441	12.8	1	1	1								
cds.Camellia_sinensis.comp9962_c0_seq1_m.50653	PREDICTED: RNA-binding protein 1-like [Juglans regia]	19.996	106.01	54.6	7	12	7	0.965	1.018	0.993	0.997	0.968	1.011	1	9.97E-01
cds.Camellia_sinensis.comp9991_c0_seq1_m.48761	PREDICTED: 60S acidic ribosomal protein P2B-like [Populus euphratica]	11.319	15.089	22.1	3	7	3	1.036	1.046	1.061	0.974	0.975	0.922	0.913	1.00E-02
cds.Camellia_sinensis.comp9994_c0_seq1_m.38016	PREDICTED: adenylate kinase 4 [Oryza sativa Japonica Group]	28.4	14.865	42.6	10	21	6	1.023	1.071	1.069	0.918	0.917	0.974	0.888	8.84E-03
