# Supplementary material for: Drought stress triggers proteomic changes involving lignin, flavonoids and fatty acids in tea plants
Source: Sci Rep. 2020 Sep 23;10:15504. doi: 10.1038/s41598-020-72596-1 (PMC7511325; doi:10.1038/s41598-020-72596-1)
Supplement: Supplementary file 3 — Supplementary Table S3. [file 41598_2020_72596_MOESM3_ESM.pdf]

Drought Stress Triggers the Proteomic Changes Involved in Lignins, Flavonoids and Fatty Acids in Tea Plants
Honglian Gu[1], Yu Wang[1], Hui Xie[1], Chen Qiu[1], Shuning Zhang[1], Jun Xiao[2], Hongyan Li[3], Liang Chen[4], Xinghui Li[5], Zhaotang Ding[1,*]
1 Tea Research Institute, Qingdao Agricultural University, Qingdao, Shandong266109, China.
2 School of Biological Science and Winery Engineering, Taishan University, Taian, Shandong271000, China.
3 Haiyang fruit technology promotion station, Yantai, Shandong265100, China.
4 Tea Research Institute, Chinese Academy of Agricultural Sciences, Hangzhou, Zhejiang310008, China.
5 Tea Research Institute, Nanjing Agricultural University, Nanjing, Jiangsu210095, China.
guhongliantea@163.com (H.G.); wangyutea@163.com (Y.W.); xiehuitea@163.com (H.X.); qiuchentea@163.com (C.Q.); zhangshuningtea@163.com (S.Z.); xiaojun8299@163.com (J.X.); sunmijia0710@163.com (H.L.); liangchen@tricaas.com (L.C.); lxh@njau.edu.cn (X.L.).
*Correspondence: dzttea@163.com; Tel: +85-15853260396

Supplementary Table S3. GO enrichment

GO Terms Level 1	GO Terms ID	GO Terms Level	GO Terms Description	Fisher's exact test P value	Protein accession	Protein description	DT/CK Ratio	Regulated Type	DT/CK P value	MW [kDa]	Score	Coverage [%]	Peptides	PSMs	Unique peptides	CK1	CK2	CK3	DT1	DT2	DT3
Cellular Component	GO:0043231	7	intracellular membrane-bounded organelle	0.167839	cds.Camellia_sinensis.comp30452_c0_seq2_m.40812	PREDICTED: methyl-CpG-binding domain-containing protein 11 [Vitis vinifera]	0.588	Down	0.000185	29.818	34.764	28.8	6	8	5	1.237	1.268	1.251	0.782	0.683	0.745
Cellular Component	GO:0043231	7	intracellular membrane-bounded organelle	0.167839	cds.Camellia_sinensis.comp33599_c1_seq11_m.43029	PREDICTED: histone H2AX [Citrus sinensis]	0.584	Down	9.72E-05	14.658	6.2367	25	5	15	2	1.193	1.245	1.316	0.749	0.739	0.704
Cellular Component	GO:0043231	7	intracellular membrane-bounded organelle	0.167839	cds.Camellia_sinensis.comp30452_c0_seq1_m.32345	PREDICTED: methyl-CpG-binding domain-containing protein 11 [Vitis vinifera]	0.654	Down	0.000178	34.5	18.045	16.8	5	10	2	1.24	1.175	1.188	0.747	0.82	0.788
Cellular Component	GO:0043231	7	intracellular membrane-bounded organelle	0.167839	cds.Camellia_sinensis.comp30146_c0_seq1_m.21634	PREDICTED: nuclear pore complex protein NUP50A [Theobroma cacao]	0.612	Down	0.000323	44.255	6.2386	12.8	4	4	4	1.311	1.197	1.167	0.75	0.718	0.78
Cellular Component	GO:0043231	7	intracellular membrane-bounded organelle	0.167839	cds.Camellia_sinensis.comp30991_c1_seq6_m.41940	PREDICTED: HMG-Y-related protein A-like [Raphanus sativus]	0.651	Down	0.00018	19.574	49.648	32.3	6	14	1	1.251	1.197	1.148	0.771	0.811	0.758
Cellular Component	GO:0043231	7	intracellular membrane-bounded organelle	0.167839	cds.Camellia_sinensis.comp19929_c0_seq1_m.31347	PREDICTED: clathrin light chain 1-like [Capsicum annuum]	0.595	Down	1.87E-05	37.317	14.334	9.5	3	3	2	1.269	1.241	1.205	0.735	0.758	0.719
Cellular Component	GO:0043228	3	non-membrane-bounded organelle	0.005856	cds.Camellia_sinensis.comp28106_c0_seq1_m.46951	PREDICTED: 60S ribosomal protein L21-1 [Daucus carota subsp. sativus]	0.659	Down	2.54E-06	18.716	16.424	30.5	6	12	6	1.198	1.207	1.205	0.774	0.798	0.807
Cellular Component	GO:0043228	3	non-membrane-bounded organelle	0.005856	cds.Camellia_sinensis.comp33599_c1_seq11_m.43029	PREDICTED: histone H2AX [Citrus sinensis]	0.584	Down	9.72E-05	14.658	6.2367	25	5	15	2	1.193	1.245	1.316	0.749	0.739	0.704
Cellular Component	GO:0043228	3	non-membrane-bounded organelle	0.005856	cds.CL83Contig1_m.15259	PREDICTED: tubulin alpha chain [Ziziphus jujuba]	0.663	Down	0.0002	49.677	103.8	32	12	24	2	1.164	1.213	1.213	0.788	0.835	0.757
Cellular Component	GO:0043228	3	non-membrane-bounded organelle	0.005856	cds.Camellia_sinensis.comp30991_c1_seq6_m.41940	PREDICTED: HMG-Y-related protein A-like [Raphanus sativus]	0.651	Down	0.00018	19.574	49.648	32.3	6	14	1	1.251	1.197	1.148	0.771	0.811	0.758
Cellular Component	GO:0043228	3	non-membrane-bounded organelle	0.005856	cds.Camellia_sinensis.comp34663_c0_seq10_m.34729	"PREDICTED: 50S ribosomal protein L15, chloroplastic [Vitis vinifera]"	0.649	Down	4.2E-06	29.408	47.897	29.7	7	10	7	1.179	1.227	1.204	0.766	0.789	0.787
Cellular Component	GO:0043228	3	non-membrane-bounded organelle	0.005856	cds.Camellia_sinensis.comp11716_c0_seq1_m.37295	PREDICTED: 40S ribosomal protein S7 [Nelumbo nucifera]	0.639	Down	1.55E-05	26.673	10.971	19.4	5	8	5	1.188	1.23	1.177	0.751	0.773	0.773
Cellular Component	GO:0043228	3	non-membrane-bounded organelle	0.005856	cds.Camellia_sinensis.comp24911_c0_seq1_m.38588	PREDICTED: 40S ribosomal protein S11-like [Daucus carota subsp. sativus]	0.639	Down	7.59E-08	17.858	12.377	35.2	7	8	7	1.2	1.215	1.197	0.766	0.767	0.774
Cellular Component	GO:0043228	3	non-membrane-bounded organelle	0.005856	cds.Camellia_sinensis.comp25442_c0_seq1_m.40798	PREDICTED: 40S ribosomal protein S15 [Ricinus communis]	0.649	Down	0.00318	17.202	10.646	19.2	2	5	1	1.215	1.125	1.264	0.756	0.871	0.713
Cellular Component	GO:0043229	6	intracellular organelle	0.01146	cds.Camellia_sinensis.comp30452_c0_seq2_m.40812	PREDICTED: methyl-CpG-binding domain-containing protein 11 [Vitis vinifera]	0.588	Down	0.000185	29.818	34.764	28.8	6	8	5	1.237	1.268	1.251	0.782	0.683	0.745
Cellular Component	GO:0043229	6	intracellular organelle	0.01146	cds.Camellia_sinensis.comp28106_c0_seq1_m.46951	PREDICTED: 60S ribosomal protein L21-1 [Daucus carota subsp. sativus]	0.659	Down	2.54E-06	18.716	16.424	30.5	6	12	6	1.198	1.207	1.205	0.774	0.798	0.807

Cellular Component	GO:0043229	6	intracellular organelle	0.01146	cds.Camellia_sinensis.comp33599_c1_seq11_m.43029	PREDICTED: histone H2AX [Citrus sinensis]	0.584	Down	9.72E-05	14.658	6.2367	25	5	15	2	1.193	1.245	1.316	0.749	0.739	0.704
Cellular Component	GO:0043229	6	intracellular organelle	0.01146	cds.CL83Contig1_m.15259	PREDICTED: tubulin alpha chain [Ziziphus jujuba]	0.663	Down	0.0002	49.677	103.8	32	12	24	2	1.164	1.213	1.213	0.788	0.835	0.757
Cellular Component	GO:0043229	6	intracellular organelle	0.01146	cds.Camellia_sinensis.comp30146_c0_seq1_m.21634	PREDICTED: nuclear pore complex protein NUP50A [Theobroma cacao]	0.612	Down	0.000323	44.255	6.2386	12.8	4	4	4	1.311	1.197	1.167	0.75	0.718	0.78
Cellular Component	GO:0043229	6	intracellular organelle	0.01146	cds.Camellia_sinensis.comp30991_c1_seq6_m.41940	PREDICTED: HMG-Y-related protein A-like [Raphanus sativus]	0.651	Down	0.00018	19.574	49.648	32.3	6	14	1	1.251	1.197	1.148	0.771	0.811	0.758
Cellular Component	GO:0043229	6	intracellular organelle	0.01146	cds.Camellia_sinensis.comp19929_c0_seq1_m.31347	PREDICTED: clathrin light chain 1-like [Capsicum annuum]	0.595	Down	1.87E-05	37.317	14.334	9.5	3	3	2	1.269	1.241	1.205	0.735	0.758	0.719
Cellular Component	GO:0043229	6	intracellular organelle	0.01146	cds.Camellia_sinensis.comp34663_c0_seq10_m.34729	"PREDICTED: 50S ribosomal protein L15, chloroplastic [Vitis vinifera]"	0.649	Down	4.2E-06	29.408	47.897	29.7	7	10	7	1.179	1.227	1.204	0.766	0.789	0.787
Cellular Component	GO:0043229	6	intracellular organelle	0.01146	cds.Camellia_sinensis.comp30452_c0_seq1_m.32345	PREDICTED: methyl-CpG-binding domain-containing protein 11 [Vitis vinifera]	0.654	Down	0.000178	34.5	18.045	16.8	5	10	2	1.24	1.175	1.188	0.747	0.82	0.788
Cellular Component	GO:0043229	6	intracellular organelle	0.01146	cds.Camellia_sinensis.comp11716_c0_seq1_m.37295	PREDICTED: 40S ribosomal protein S7 [Nelumbo nucifera]	0.639	Down	1.55E-05	26.673	10.971	19.4	5	8	5	1.188	1.23	1.177	0.751	0.773	0.773
Cellular Component	GO:0043229	6	intracellular organelle	0.01146	cds.Camellia_sinensis.comp24911_c0_seq1_m.38588	PREDICTED: 40S ribosomal protein S11-like [Daucus carota subsp. sativus]	0.639	Down	7.59E-08	17.858	12.377	35.2	7	8	7	1.2	1.215	1.197	0.766	0.767	0.774
Cellular Component	GO:0043229	6	intracellular organelle	0.01146	cds.Camellia_sinensis.comp25442_c0_seq1_m.40798	PREDICTED: 40S ribosomal protein S15 [Ricinus communis]	0.649	Down	0.00318	17.202	10.646	19.2	2	5	1	1.215	1.125	1.264	0.756	0.871	0.713
Cellular Component	GO:0043226	2	organelle	0.01146	cds.Camellia_sinensis.comp30452_c0_seq2_m.40812	PREDICTED: methyl-CpG-binding domain-containing protein 11 [Vitis vinifera]	0.588	Down	0.000185	29.818	34.764	28.8	6	8	5	1.237	1.268	1.251	0.782	0.683	0.745
Cellular Component	GO:0043226	2	organelle	0.01146	cds.Camellia_sinensis.comp28106_c0_seq1_m.46951	PREDICTED: 60S ribosomal protein L21-1 [Daucus carota subsp. sativus]	0.659	Down	2.54E-06	18.716	16.424	30.5	6	12	6	1.198	1.207	1.205	0.774	0.798	0.807
Cellular Component	GO:0043226	2	organelle	0.01146	cds.Camellia_sinensis.comp33599_c1_seq11_m.43029	PREDICTED: histone H2AX [Citrus sinensis]	0.584	Down	9.72E-05	14.658	6.2367	25	5	15	2	1.193	1.245	1.316	0.749	0.739	0.704
Cellular Component	GO:0043226	2	organelle	0.01146	cds.CL83Contig1_m.15259	PREDICTED: tubulin alpha chain [Ziziphus jujuba]	0.663	Down	0.0002	49.677	103.8	32	12	24	2	1.164	1.213	1.213	0.788	0.835	0.757
Cellular Component	GO:0043226	2	organelle	0.01146	cds.Camellia_sinensis.comp30146_c0_seq1_m.21634	PREDICTED: nuclear pore complex protein NUP50A [Theobroma cacao]	0.612	Down	0.000323	44.255	6.2386	12.8	4	4	4	1.311	1.197	1.167	0.75	0.718	0.78
Cellular Component	GO:0043226	2	organelle	0.01146	cds.Camellia_sinensis.comp30991_c1_seq6_m.41940	PREDICTED: HMG-Y-related protein A-like [Raphanus sativus]	0.651	Down	0.00018	19.574	49.648	32.3	6	14	1	1.251	1.197	1.148	0.771	0.811	0.758
Cellular Component	GO:0043226	2	organelle	0.01146	cds.Camellia_sinensis.comp19929_c0_seq1_m.31347	PREDICTED: clathrin light chain 1-like [Capsicum annuum]	0.595	Down	1.87E-05	37.317	14.334	9.5	3	3	2	1.269	1.241	1.205	0.735	0.758	0.719
Cellular Component	GO:0043226	2	organelle	0.01146	cds.Camellia_sinensis.comp34663_c0_seq10_m.34729	"PREDICTED: 50S ribosomal protein L15, chloroplastic [Vitis vinifera]"	0.649	Down	4.2E-06	29.408	47.897	29.7	7	10	7	1.179	1.227	1.204	0.766	0.789	0.787
Cellular Component	GO:0043226	2	organelle	0.01146	cds.Camellia_sinensis.comp30452_c0_seq1_m.32345	PREDICTED: methyl-CpG-binding domain-containing protein 11 [Vitis vinifera]	0.654	Down	0.000178	34.5	18.045	16.8	5	10	2	1.24	1.175	1.188	0.747	0.82	0.788

Cellular Component	GO:0043226	2	organelle	0.01146	cds.Camellia_sinensis.comp11716_c0_seq1_m.37295	PREDICTED: 40S ribosomal protein S7 [Nelumbo nucifera]	0.639	Down	1.55E-05	26.673	10.971	19.4	5	8	5	1.188	1.23	1.177	0.751	0.773	0.773
Cellular Component	GO:0043226	2	organelle	0.01146	cds.Camellia_sinensis.comp24911_c0_seq1_m.38588	PREDICTED: 40S ribosomal protein S11-like [Daucus carota subsp. sativus]	0.639	Down	7.59E-08	17.858	12.377	35.2	7	8	7	1.2	1.215	1.197	0.766	0.767	0.774
Cellular Component	GO:0043226	2	organelle	0.01146	cds.Camellia_sinensis.comp25442_c0_seq1_m.40798	PREDICTED: 40S ribosomal protein S15 [Ricinus communis]	0.649	Down	0.00318	17.202	10.646	19.2	2	5	1	1.215	1.125	1.264	0.756	0.871	0.713
Cellular Component	GO:0005634	8	nucleus	0.01935	cds.Camellia_sinensis.comp30452_c0_seq2_m.40812	PREDICTED: methyl-CpG-binding domain-containing protein 11 [Vitis vinifera]	0.588	Down	0.000185	29.818	34.764	28.8	6	8	5	1.237	1.268	1.251	0.782	0.683	0.745
Cellular Component	GO:0005634	8	nucleus	0.01935	cds.Camellia_sinensis.comp33599_c1_seq11_m.43029	PREDICTED: histone H2AX [Citrus sinensis]	0.584	Down	9.72E-05	14.658	6.2367	25	5	15	2	1.193	1.245	1.316	0.749	0.739	0.704
Cellular Component	GO:0005634	8	nucleus	0.01935	cds.Camellia_sinensis.comp30452_c0_seq1_m.32345	PREDICTED: methyl-CpG-binding domain-containing protein 11 [Vitis vinifera]	0.654	Down	0.000178	34.5	18.045	16.8	5	10	2	1.24	1.175	1.188	0.747	0.82	0.788
Cellular Component	GO:0005634	8	nucleus	0.01935	cds.Camellia_sinensis.comp30146_c0_seq1_m.21634	PREDICTED: nuclear pore complex protein NUP50A [Theobroma cacao]	0.612	Down	0.000323	44.255	6.2386	12.8	4	4	4	1.311	1.197	1.167	0.75	0.718	0.78
Cellular Component	GO:0005634	8	nucleus	0.01935	cds.Camellia_sinensis.comp30991_c1_seq6_m.41940	PREDICTED: HMG-Y-related protein A-like [Raphanus sativus]	0.651	Down	0.00018	19.574	49.648	32.3	6	14	1	1.251	1.197	1.148	0.771	0.811	0.758
Cellular Component	GO:0044446	7	intracellular organelle part	0.025252	cds.Camellia_sinensis.comp33599_c1_seq11_m.43029	PREDICTED: histone H2AX [Citrus sinensis]	0.584	Down	9.72E-05	14.658	6.2367	25	5	15	2	1.193	1.245	1.316	0.749	0.739	0.704
Cellular Component	GO:0044446	7	intracellular organelle part	0.025252	cds.Camellia_sinensis.comp34663_c0_seq10_m.34729	"PREDICTED: 50S ribosomal protein L15, chloroplastic [Vitis vinifera]"	0.649	Down	4.2E-06	29.408	47.897	29.7	7	10	7	1.179	1.227	1.204	0.766	0.789	0.787
Cellular Component	GO:0044446	7	intracellular organelle part	0.025252	cds.CL83Contig1_m.15259	PREDICTED: tubulin alpha chain [Ziziphus jujuba]	0.663	Down	0.0002	49.677	103.8	32	12	24	2	1.164	1.213	1.213	0.788	0.835	0.757
Cellular Component	GO:0044446	7	intracellular organelle part	0.025252	cds.Camellia_sinensis.comp30146_c0_seq1_m.21634	PREDICTED: nuclear pore complex protein NUP50A [Theobroma cacao]	0.612	Down	0.000323	44.255	6.2386	12.8	4	4	4	1.311	1.197	1.167	0.75	0.718	0.78
Cellular Component	GO:0044446	7	intracellular organelle part	0.025252	cds.Camellia_sinensis.comp30991_c1_seq6_m.41940	PREDICTED: HMG-Y-related protein A-like [Raphanus sativus]	0.651	Down	0.00018	19.574	49.648	32.3	6	14	1	1.251	1.197	1.148	0.771	0.811	0.758
Cellular Component	GO:0044446	7	intracellular organelle part	0.025252	cds.Camellia_sinensis.comp25442_c0_seq1_m.40798	PREDICTED: 40S ribosomal protein S15 [Ricinus communis]	0.649	Down	0.00318	17.202	10.646	19.2	2	5	1	1.215	1.125	1.264	0.756	0.871	0.713
Cellular Component	GO:0044446	7	intracellular organelle part	0.025252	cds.Camellia_sinensis.comp19929_c0_seq1_m.31347	PREDICTED: clathrin light chain 1-like [Capsicum annuum]	0.595	Down	1.87E-05	37.317	14.334	9.5	3	3	2	1.269	1.241	1.205	0.735	0.758	0.719
Cellular Component	GO:0044422	3	organelle part	0.025252	cds.Camellia_sinensis.comp33599_c1_seq11_m.43029	PREDICTED: histone H2AX [Citrus sinensis]	0.584	Down	9.72E-05	14.658	6.2367	25	5	15	2	1.193	1.245	1.316	0.749	0.739	0.704
Cellular Component	GO:0044422	3	organelle part	0.025252	cds.Camellia_sinensis.comp34663_c0_seq10_m.34729	"PREDICTED: 50S ribosomal protein L15, chloroplastic [Vitis vinifera]"	0.649	Down	4.2E-06	29.408	47.897	29.7	7	10	7	1.179	1.227	1.204	0.766	0.789	0.787
Cellular Component	GO:0044422	3	organelle part	0.025252	cds.CL83Contig1_m.15259	PREDICTED: tubulin alpha chain [Ziziphus jujuba]	0.663	Down	0.0002	49.677	103.8	32	12	24	2	1.164	1.213	1.213	0.788	0.835	0.757
Cellular Component	GO:0044422	3	organelle part	0.025252	cds.Camellia_sinensis.comp30146_c0_seq1_m.21634	PREDICTED: nuclear pore complex protein NUP50A [Theobroma cacao]	0.612	Down	0.000323	44.255	6.2386	12.8	4	4	4	1.311	1.197	1.167	0.75	0.718	0.78

Cellular Component	GO:0044422	3	organelle part	0.025252	cds.Camellia_sinensis.comp30991_c1_seq6_m.41940	PREDICTED: HMG-Y-related protein A-like [Raphanus sativus]	0.651	Down	0.00018	19.574	49.648	32.3	6	14	1	1.251	1.197	1.148	0.771	0.811	0.758
Cellular Component	GO:0044422	3	organelle part	0.025252	cds.Camellia_sinensis.comp25442_c0_seq1_m.40798	PREDICTED: 40S ribosomal protein S15 [Ricinus communis]	0.649	Down	0.00318	17.202	10.646	19.2	2	5	1	1.215	1.125	1.264	0.756	0.871	0.713
Cellular Component	GO:0044422	3	organelle part	0.025252	cds.Camellia_sinensis.comp19929_c0_seq1_m.31347	PREDICTED: clathrin light chain 1-like [Capsicum annuum]	0.595	Down	1.87E-05	37.317	14.334	9.5	3	3	2	1.269	1.241	1.205	0.735	0.758	0.719
Cellular Component	GO:0071944	4	cell periphery	0.037305	cds.Camellia_sinensis.comp7306_c0_seq1_m.21311	PREDICTED: pectinesterase-like [Gossypium hirsutum]	0.662	Down	4.3E-06	56.944	99.605	20.4	8	22	8	1.19	1.181	1.217	0.809	0.786	0.78
Cellular Component	GO:0071944	4	cell periphery	0.037305	cds.Camellia_sinensis.comp26419_c0_seq1_m.19908	PREDICTED: probable xyloglucan endotransglucosylase/hydrolase protein 6 [Juglans regia]	0.573	Down	5.66E-05	32.893	26.298	26.8	8	11	6	1.24	1.256	1.279	0.758	0.72	0.685
Cellular Component	GO:0071944	4	cell periphery	0.037305	cds.Camellia_sinensis.comp19929_c0_seq1_m.31347	PREDICTED: clathrin light chain 1-like [Capsicum annuum]	0.595	Down	1.87E-05	37.317	14.334	9.5	3	3	2	1.269	1.241	1.205	0.735	0.758	0.719
Molecular Function	GO:0003855	6	3-dehydroquinate dehydratase activity	0.001751	cds.Camellia_sinensis.comp28445_c0_seq1_m.18163	"PREDICTED: bifunctional 3-dehydroquinate dehydratase/shikimate dehydrogenase, chloroplastic [Vitis vinifera]"	0.422	Down	1.75E-05	56.806	13.472	8.6	5	7	4	1.421	1.362	1.341	0.547	0.603	0.591
Molecular Function	GO:0003855	6	3-dehydroquinate dehydratase activity	0.001751	cds.Camellia_sinensis.comp12441_c0_seq1_m.14154	"PREDICTED: bifunctional 3-dehydroquinate dehydratase/shikimate dehydrogenase, chloroplastic [Vitis vinifera]"	0.557	Down	1.48E-06	58.235	113.88	32.7	17	30	16	1.237	1.275	1.282	0.707	0.716	0.69
Molecular Function	GO:0003849	5	3-deoxy-7-phosphoheptulonate synthase activity	0.001751	cds.Camellia_sinensis.comp30057_c1_seq2_m.50785	"PREDICTED: phospho-2-dehydro-3-deoxyheptonate aldolase 1, chloroplastic-like [Malus domestica]"	0.492	Down	3.57E-05	19.615	40.349	26.9	4	8	4	1.335	1.244	1.382	0.642	0.668	0.639
Molecular Function	GO:0003849	5	3-deoxy-7-phosphoheptulonate synthase activity	0.001751	cds.Camellia_sinensis.comp30057_c0_seq1_m.29301	2-dehydro-3-deoxyphosphoheptonate aldolase family protein [Populus trichocarpa]	0.521	Down	0.000523	43.241	36.368	32.1	13	31	7	1.294	1.258	1.324	0.676	0.671	0.673
Molecular Function	GO:0004764	6	shikimate 3-dehydrogenase (NADP+) activity	0.001751	cds.Camellia_sinensis.comp28445_c0_seq1_m.18163	"PREDICTED: bifunctional 3-dehydroquinate dehydratase/shikimate dehydrogenase, chloroplastic [Vitis vinifera]"	0.422	Down	1.75E-05	56.806	13.472	8.6	5	7	4	1.421	1.362	1.341	0.547	0.603	0.591
Molecular Function	GO:0004764	6	shikimate 3-dehydrogenase (NADP+) activity	0.001751	cds.Camellia_sinensis.comp12441_c0_seq1_m.14154	"PREDICTED: bifunctional 3-dehydroquinate dehydratase/shikimate dehydrogenase, chloroplastic [Vitis vinifera]"	0.557	Down	1.48E-06	58.235	113.88	32.7	17	30	16	1.237	1.275	1.282	0.707	0.716	0.69
Molecular Function	GO:0016746	4	transferase activity, transferring acyl groups	0.003686	cds.Camellia_sinensis.comp28704_c0_seq1_m.23471	PREDICTED: BAHD acyltransferase DCR [Nicotiana attenuata]	0.462	Down	6.9E-07	49.995	46.095	13.4	5	6	5	1.335	1.368	1.341	0.636	0.605	0.629
Molecular Function	GO:0016746	4	transferase activity, transferring acyl groups	0.003686	cds.Camellia_sinensis.comp24164_c0_seq1_m.25907	PREDICTED: protein ECERIFERUM 26 [Vitis vinifera]	0.557	Down	0.001556	49.78	18.322	13.3	5	8	5	1.223	1.255	1.319	0.68	0.807	0.629

Molecular Function	GO:0016746	4	transferase activity, transferring acyl groups	0.003686	cds.Camellia_sinensis.comp16373_c0_seq1_m.51330	PREDICTED: chalcone synthase [Pyrus x bretschneideri]	0.391	Down	6.01E-05	19.938	47.804	46.6	6	27	1	1.467	1.452	1.298	0.561	0.511	0.575
Molecular Function	GO:0016746	4	transferase activity, transferring acyl groups	0.003686	cds.Camellia_sinensis.comp27350_c0_seq1_m.24260	PREDICTED: chalcone synthase 1 [Juglans regia]	0.35	Down	2.17E-05	48.966	67.923	32.2	11	35	2	1.518	1.391	1.424	0.472	0.54	0.506
Molecular Function	GO:0016746	4	transferase activity, transferring acyl groups	0.003686	cds.Camellia_sinensis.comp24298_c0_seq1_m.23229	PREDICTED: 3-ketoacyl-CoA synthase 6 [Sesamum indicum]	0.213	Down	0.006598	56.181	4.7365	4.4	2	3	2	1.558	1.615	1.613	0.265	0.345	0.41
Molecular Function	GO:0016746	4	transferase activity, transferring acyl groups	0.003686	cds.Camellia_sinensis.comp41274_c0_seq1_m.27135	PREDICTED: omega-hydroxypalmitate O-feruloyl transferase [Vitis vinifera]	0.534	Down	5.93E-05	50.195	15.876	13.3	6	10	6	1.296	1.24	1.31	0.678	0.725	0.652
Molecular Function	GO:0016841	5	ammonia-lyase activity	0.008343	cds.Camellia_sinensis.comp24818_c0_seq1_m.28200	PREDICTED: phenylalanine ammonia-lyase 1 [Daucus carota subsp. sativus]	0.48	Down	2.07E-05	45.753	19.992	40.3	16	32	7	1.376	1.315	1.268	0.616	0.661	0.625
Molecular Function	GO:0016841	5	ammonia-lyase activity	0.008343	cds.Camellia_sinensis.comp32304_c0_seq3_m.11225	PREDICTED: phenylalanine ammonia-lyase [Vitis vinifera]	0.417	Down	1.92E-07	77.077	118.08	37.8	25	54	6	1.375	1.378	1.392	0.561	0.579	0.587
Molecular Function	GO:0020037	5	heme binding	0.01063	cds.Camellia_sinensis.comp34996_c0_seq1_m.19097	flavonoid 3' hydroxylase [Vitis vinifera]	0.538	Down	2.09E-06	57.059	17.251	19.1	9	14	9	1.248	1.29	1.269	0.668	0.678	0.703
Molecular Function	GO:0020037	5	heme binding	0.01063	cds.Camellia_sinensis.comp28142_c0_seq1_m.18076	PREDICTED: trans-cinnamate 4-monooxygenase [Jatropha curcas]	0.578	Down	1.1E-06	60.624	61.458	26.1	13	20	13	1.245	1.257	1.245	0.711	0.741	0.714
Molecular Function	GO:0020037	5	heme binding	0.01063	cds.Camellia_sinensis.comp24956_c0_seq1_m.23294	PREDICTED: peroxidase 42 [Eucalyptus grandis]	0.609	Down	2.14E-05	37.589	27.808	24	6	6	6	1.202	1.251	1.235	0.749	0.773	0.725
Molecular Function	GO:0020037	5	heme binding	0.01063	cds.Camellia_sinensis.comp31451_c0_seq1_m.18500	PREDICTED: cytochrome P450 86B1-like [Sesamum indicum]	0.487	Down	0.000742	57.848	5.5293	6.8	3	3	3	1.404	1.26	1.295	0.609	0.584	0.735
Molecular Function	GO:0020037	5	heme binding	0.01063	cds.Camellia_sinensis.comp35044_c0_seq1_m.44426	PREDICTED: cytochrome b5 [Vitis vinifera]	0.584	Down	0.000804	17.783	29.766	39.6	4	8	4	1.317	1.204	1.242	0.773	0.657	0.768
Molecular Function	GO:0020037	5	heme binding	0.01063	cds.Camellia_sinensis.comp26137_c0_seq1_m.14029	"PREDICTED: flavonoid 3',5'-hydroxylase 2-like [Jatropha curcas]"	0.479	Down	0.000165	57.074	16.113	17.6	8	10	8	1.242	1.363	1.379	0.639	0.682	0.588
Molecular Function	GO:0016835	4	carbon-oxygen lyase activity	0.013102	cds.Camellia_sinensis.comp28445_c0_seq1_m.18163	"PREDICTED: bifunctional 3-dehydroquinate dehydratase/shikimate dehydrogenase, chloroplastic [Vitis vinifera]"	0.422	Down	1.75E-05	56.806	13.472	8.6	5	7	4	1.421	1.362	1.341	0.547	0.603	0.591
Molecular Function	GO:0016835	4	carbon-oxygen lyase activity	0.013102	cds.Camellia_sinensis.comp32592_c3_seq4_m.29222	"PREDICTED: arogenate dehydratase 3, chloroplastic [Vitis vinifera]"	0.583	Down	0.003784	45.948	3.0291	4.3	2	2	1	1.331	1.137	1.271	0.663	0.842	0.675
Molecular Function	GO:0016835	4	carbon-oxygen lyase activity	0.013102	cds.Camellia_sinensis.comp12441_c0_seq1_m.14154	"PREDICTED: bifunctional 3-dehydroquinate dehydratase/shikimate dehydrogenase, chloroplastic [Vitis vinifera]"	0.557	Down	1.48E-06	58.235	113.88	32.7	17	30	16	1.237	1.275	1.282	0.707	0.716	0.69
Molecular Function	GO:0016835	4	carbon-oxygen lyase activity	0.013102	cds.Camellia_sinensis.comp33776_c0_seq1_m.19252	PREDICTED: (-)-alpha-terpineol synthase [Vitis vinifera]	0.505	Down	2.15E-05	70.02	47.141	12.2	7	10	7	1.351	1.265	1.304	0.639	0.691	0.651
Molecular Function	GO:0046906	4	tetrapyrrole binding	0.013468	cds.Camellia_sinensis.comp34996_c0_seq1_m.19097	flavonoid 3' hydroxylase [Vitis vinifera]	0.538	Down	2.09E-06	57.059	17.251	19.1	9	14	9	1.248	1.29	1.269	0.668	0.678	0.703

Molecular Function	GO:0046906	4	tetrapyrrole binding	0.013468	cds.Camellia_sinensis.comp28142_c0_seq1_m.18076	PREDICTED: trans-cinnamate 4-monooxygenase [Jatropha curcas]	0.578	Down	1.1E-06	60.624	61.458	26.1	13	20	13	1.245	1.257	1.245	0.711	0.741	0.714
Molecular Function	GO:0046906	4	tetrapyrrole binding	0.013468	cds.Camellia_sinensis.comp24956_c0_seq1_m.23294	PREDICTED: peroxidase 42 [Eucalyptus grandis]	0.609	Down	2.14E-05	37.589	27.808	24	6	6	6	1.202	1.251	1.235	0.749	0.773	0.725
Molecular Function	GO:0046906	4	tetrapyrrole binding	0.013468	cds.Camellia_sinensis.comp31451_c0_seq1_m.18500	PREDICTED: cytochrome P450 86B1-like [Sesamum indicum]	0.487	Down	0.000742	57.848	5.5293	6.8	3	3	3	1.404	1.26	1.295	0.609	0.584	0.735
Molecular Function	GO:0046906	4	tetrapyrrole binding	0.013468	cds.Camellia_sinensis.comp35044_c0_seq1_m.44426	PREDICTED: cytochrome b5 [Vitis vinifera]	0.584	Down	0.000804	17.783	29.766	39.6	4	8	4	1.317	1.204	1.242	0.773	0.657	0.768
Molecular Function	GO:0046906	4	tetrapyrrole binding	0.013468	cds.Camellia_sinensis.comp26137_c0_seq1_m.14029	"PREDICTED: flavonoid 3',5'-hydroxylase 2-like [Jatropha curcas]"	0.479	Down	0.000165	57.074	16.113	17.6	8	10	8	1.242	1.363	1.379	0.639	0.682	0.588
Biological Process	GO:0006558	11	L-phenylalanine metabolic process	0.0004	cds.Camellia_sinensis.comp24818_c0_seq1_m.28200	PREDICTED: phenylalanine ammonia-lyase 1 [Daucus carota subsp. sativus]	0.48	Down	2.07E-05	45.753	19.992	40.3	16	32	7	1.376	1.315	1.268	0.616	0.661	0.625
Biological Process	GO:0006558	11	L-phenylalanine metabolic process	0.0004	cds.Camellia_sinensis.comp32592_c3_seq4_m.29222	"PREDICTED: arogenate dehydratase 3, chloroplastic [Vitis vinifera]"	0.583	Down	0.003784	45.948	3.0291	4.3	2	2	1	1.331	1.137	1.271	0.663	0.842	0.675
Biological Process	GO:0006558	11	L-phenylalanine metabolic process	0.0004	cds.Camellia_sinensis.comp32304_c0_seq3_m.11225	PREDICTED: phenylalanine ammonia-lyase [Vitis vinifera]	0.417	Down	1.92E-07	77.077	118.08	37.8	25	54	6	1.375	1.378	1.392	0.561	0.579	0.587
Biological Process	GO:1902221	10	erythrose 4-phosphate/phosphoenolpyruvate family amino acid metabolic process	0.0004	cds.Camellia_sinensis.comp24818_c0_seq1_m.28200	PREDICTED: phenylalanine ammonia-lyase 1 [Daucus carota subsp. sativus]	0.48	Down	2.07E-05	45.753	19.992	40.3	16	32	7	1.376	1.315	1.268	0.616	0.661	0.625
Biological Process	GO:1902221	10	erythrose 4-phosphate/phosphoenolpyruvate family amino acid metabolic process	0.0004	cds.Camellia_sinensis.comp32592_c3_seq4_m.29222	"PREDICTED: arogenate dehydratase 3, chloroplastic [Vitis vinifera]"	0.583	Down	0.003784	45.948	3.0291	4.3	2	2	1	1.331	1.137	1.271	0.663	0.842	0.675
Biological Process	GO:1902221	10	erythrose 4-phosphate/phosphoenolpyruvate family amino acid metabolic process	0.0004	cds.Camellia_sinensis.comp32304_c0_seq3_m.11225	PREDICTED: phenylalanine ammonia-lyase [Vitis vinifera]	0.417	Down	1.92E-07	77.077	118.08	37.8	25	54	6	1.375	1.378	1.392	0.561	0.579	0.587
Biological Process	GO:0009072	9	aromatic amino acid family metabolic process	0.000631	cds.Camellia_sinensis.comp24818_c0_seq1_m.28200	PREDICTED: phenylalanine ammonia-lyase 1 [Daucus carota subsp. sativus]	0.48	Down	2.07E-05	45.753	19.992	40.3	16	32	7	1.376	1.315	1.268	0.616	0.661	0.625
Biological Process	GO:0009072	9	aromatic amino acid family metabolic process	0.000631	cds.Camellia_sinensis.comp30057_c1_seq2_m.50785	"PREDICTED: phospho-2-dehydro-3-deoxyheptonate aldolase 1, chloroplastic-like [Malus domestica]"	0.492	Down	3.57E-05	19.615	40.349	26.9	4	8	4	1.335	1.244	1.382	0.642	0.668	0.639
Biological Process	GO:0009072	9	aromatic amino acid family metabolic process	0.000631	cds.Camellia_sinensis.comp32592_c3_seq4_m.29222	"PREDICTED: arogenate dehydratase 3, chloroplastic [Vitis vinifera]"	0.583	Down	0.003784	45.948	3.0291	4.3	2	2	1	1.331	1.137	1.271	0.663	0.842	0.675
Biological Process	GO:0009072	9	aromatic amino acid family metabolic process	0.000631	cds.Camellia_sinensis.comp30057_c0_seq1_m.29301	2-dehydro-3-deoxyphosphoheptonate aldolase family protein [Populus trichocarpa]	0.521	Down	0.000523	43.241	36.368	32.1	13	31	7	1.294	1.258	1.324	0.676	0.671	0.673
Biological Process	GO:0009072	9	aromatic amino acid family metabolic process	0.000631	cds.Camellia_sinensis.comp32304_c0_seq3_m.11225	PREDICTED: phenylalanine ammonia-lyase [Vitis vinifera]	0.417	Down	1.92E-07	77.077	118.08	37.8	25	54	6	1.375	1.378	1.392	0.561	0.579	0.587

Biological Process	GO:1902222	11	erythrose 4-phosphate/phosphoenolpyruvate family amino acid catabolic process	0.005163	cds.Camellia_sinensis.comp24818_c0_seq1_m.28200	PREDICTED: phenylalanine ammonia-lyase 1 [Daucus carota subsp. sativus]	0.48	Down	2.07E-05	45.753	19.992	40.3	16	32	7	1.376	1.315	1.268	0.616	0.661	0.625
Biological Process	GO:1902222	11	erythrose 4-phosphate/phosphoenolpyruvate family amino acid catabolic process	0.005163	cds.Camellia_sinensis.comp32304_c0_seq3_m.11225	PREDICTED: phenylalanine ammonia-lyase [Vitis vinifera]	0.417	Down	1.92E-07	77.077	118.08	37.8	25	54	6	1.375	1.378	1.392	0.561	0.579	0.587
Biological Process	GO:0006559	12	L-phenylalanine catabolic process	0.005163	cds.Camellia_sinensis.comp24818_c0_seq1_m.28200	PREDICTED: phenylalanine ammonia-lyase 1 [Daucus carota subsp. sativus]	0.48	Down	2.07E-05	45.753	19.992	40.3	16	32	7	1.376	1.315	1.268	0.616	0.661	0.625
Biological Process	GO:0006559	12	L-phenylalanine catabolic process	0.005163	cds.Camellia_sinensis.comp32304_c0_seq3_m.11225	PREDICTED: phenylalanine ammonia-lyase [Vitis vinifera]	0.417	Down	1.92E-07	77.077	118.08	37.8	25	54	6	1.375	1.378	1.392	0.561	0.579	0.587
Biological Process	GO:0009074	10	aromatic amino acid family catabolic process	0.007628	cds.Camellia_sinensis.comp24818_c0_seq1_m.28200	PREDICTED: phenylalanine ammonia-lyase 1 [Daucus carota subsp. sativus]	0.48	Down	2.07E-05	45.753	19.992	40.3	16	32	7	1.376	1.315	1.268	0.616	0.661	0.625
Biological Process	GO:0009074	10	aromatic amino acid family catabolic process	0.007628	cds.Camellia_sinensis.comp32304_c0_seq3_m.11225	PREDICTED: phenylalanine ammonia-lyase [Vitis vinifera]	0.417	Down	1.92E-07	77.077	118.08	37.8	25	54	6	1.375	1.378	1.392	0.561	0.579	0.587
Biological Process	GO:0009073	10	aromatic amino acid family biosynthetic process	0.007766	cds.Camellia_sinensis.comp30057_c1_seq2_m.50785	"PREDICTED: phospho-2-dehydro-3-deoxyheptonate aldolase 1, chloroplastic-like [Malus domestica]"	0.492	Down	3.57E-05	19.615	40.349	26.9	4	8	4	1.335	1.244	1.382	0.642	0.668	0.639
Biological Process	GO:0009073	10	aromatic amino acid family biosynthetic process	0.007766	cds.Camellia_sinensis.comp32592_c3_seq4_m.29222	"PREDICTED: arogenate dehydratase 3, chloroplastic [Vitis vinifera]"	0.583	Down	0.003784	45.948	3.0291	4.3	2	2	1	1.331	1.137	1.271	0.663	0.842	0.675
Biological Process	GO:0009073	10	aromatic amino acid family biosynthetic process	0.007766	cds.Camellia_sinensis.comp30057_c0_seq1_m.29301	2-dehydro-3-deoxyphosphoheptonate aldolase family protein [Populus trichocarpa]	0.521	Down	0.000523	43.241	36.368	32.1	13	31	7	1.294	1.258	1.324	0.676	0.671	0.673
Biological Process	GO:0044036	6	cell wall macromolecule metabolic process	0.013816	cds.Camellia_sinensis.comp26419_c0_seq1_m.19908	PREDICTED: probable xyloglucan endotransglucosylase/hydrolase protein 6 [Juglans regia]	0.573	Down	5.66E-05	32.893	26.298	26.8	8	11	6	1.24	1.256	1.279	0.758	0.72	0.685
Biological Process	GO:0044036	6	cell wall macromolecule metabolic process	0.013816	cds.Camellia_sinensis.comp34586_c0_seq4_m.32396	PREDICTED: endochitinase PR4-like [Nelumbo nucifera]	1.537	Up	0.000316	24.711	10.503	11.5	2	2	2	0.855	0.774	0.763	1.226	1.205	1.246
Biological Process	GO:1901565	5	organonitrogen compound catabolic process	0.021679	cds.Camellia_sinensis.comp24818_c0_seq1_m.28200	PREDICTED: phenylalanine ammonia-lyase 1 [Daucus carota subsp. sativus]	0.48	Down	2.07E-05	45.753	19.992	40.3	16	32	7	1.376	1.315	1.268	0.616	0.661	0.625
Biological Process	GO:1901565	5	organonitrogen compound catabolic process	0.021679	cds.Camellia_sinensis.comp34586_c0_seq4_m.32396	PREDICTED: endochitinase PR4-like [Nelumbo nucifera]	1.537	Up	0.000316	24.711	10.503	11.5	2	2	2	0.855	0.774	0.763	1.226	1.205	1.246
Biological Process	GO:1901565	5	organonitrogen compound catabolic process	0.021679	cds.Camellia_sinensis.comp32304_c0_seq3_m.11225	PREDICTED: phenylalanine ammonia-lyase [Vitis vinifera]	0.417	Down	1.92E-07	77.077	118.08	37.8	25	54	6	1.375	1.378	1.392	0.561	0.579	0.587
Biological Process	GO:1901606	10	alpha-amino acid catabolic process	0.046652	cds.Camellia_sinensis.comp24818_c0_seq1_m.28200	PREDICTED: phenylalanine ammonia-lyase 1 [Daucus carota subsp. sativus]	0.48	Down	2.07E-05	45.753	19.992	40.3	16	32	7	1.376	1.315	1.268	0.616	0.661	0.625

Biological Process	GO:1901606	10	alpha-amino acid catabolic process	0.046652	cds.Camellia_sinensis.comp32304_c0_seq3_m.11225	PREDICTED: phenylalanine ammonia-lyase [Vitis vinifera]	0.417	Down	1.92E-07	77.077	118.08	37.8	25	54	6	1.375	1.378	1.392	0.561	0.579	0.587
