# Supplementary material for: Drought stress triggers proteomic changes involving lignin, flavonoids and fatty acids in tea plants
Source: Sci Rep. 2020 Sep 23;10:15504. doi: 10.1038/s41598-020-72596-1 (PMC7511325; doi:10.1038/s41598-020-72596-1)
Supplement: Supplementary file 4 — Supplementary Table S4. [file 41598_2020_72596_MOESM4_ESM.pdf]

Drought Stress Triggers the Proteomic Changes Involved in Lignins, Flavonoids and Fatty Acids in Tea Plants

Honglian Gu[1], Yu Wang[1], Hui Xie[1], Chen Qiu[1], Shuning Zhang[1], Jun Xiao[2], Hongyan Li[3], Liang Chen[4], Xinghui Li[5], Zhaotang Ding[1,*]
1 Tea Research Institute, Qingdao Agricultural University, Qingdao, Shandong266109, China.
2 School of Biological Science and Winery Engineering, Taishan University, Taian, Shandong271000, China.
3 Haiyang fruit technology promotion station, Yantai, Shandong265100, China.
4 Tea Research Institute, Chinese Academy of Agricultural Sciences, Hangzhou, Zhejiang310008, China.
5 Tea Research Institute, Nanjing Agricultural University, Nanjing, Jiangsu210095, China.
guhongliantea@163.com (H.G.); wangyutea@163.com (Y.W.); xiehuitea@163.com (H.X.); qiuchentea@163.com (C.Q.); zhangshuningtea@163.com (S.Z.); xiaojun8299@163.com (J.X.); sunmijia0710@163.com (H.L.); liangchen@tricaas.com (L.C.); lxh@njau.edu.cn (X.L.).
*Correspondence: dzttea@163.com; Tel: +85-15853260396

Supplementary Table S4. KEGG enrichment

KEGG pathway	Fisher's exact test p value	Protein accession	Protein description	DT/CK Ratio	Regulated Type	DT/CK P value	MW [kDa]	Score	Coverage [%]	Peptides	PSMs	Unique peptides	CK1	CK2	CK3	DT1	DT2	DT3
ath01110 Biosynthesis of secondary metabolites	0.000275	cds.Camellia_sinensis.comp25454_c0_seq1_m.28314	"PREDICTED: naringenin,2-oxoglutarate 3-dioxygenase [Ziziphus jujuba]"	0.41	Down	5.66E-07	44.491	178.49	49.4	18	49	18	1.386	1.375	1.399	0.556	0.589	0.561
ath01110 Biosynthesis of secondary metabolites	0.000275	cds.Camellia_sinensis.comp29812_c0_seq1_m.18555	PREDICTED: 4-coumarate--CoA ligase 2-like [Jatropha curcas]	0.636	Down	0.000662	60.204	4.0311	7.8	5	5	2	1.142	1.254	1.239	0.822	0.723	0.767
ath01110 Biosynthesis of secondary metabolites	0.000275	cds.Camellia_sinensis.comp28142_c0_seq1_m.18076	PREDICTED: trans-cinnamate 4-monooxygenase [Jatropha curcas]	0.578	Down	1.1E-06	60.624	61.458	26.1	13	20	13	1.245	1.257	1.245	0.711	0.741	0.714
ath01110 Biosynthesis of secondary metabolites	0.000275	cds.Camellia_sinensis.comp32592_c3_seq4_m.29222	"PREDICTED: arogenate dehydratase 3, chloroplastic [Vitis vinifera]"	0.583	Down	0.003784	45.948	3.0291	4.3	2	2	1	1.331	1.137	1.271	0.663	0.842	0.675
ath01110 Biosynthesis of secondary metabolites	0.000275	cds.Camellia_sinensis.comp12781_c0_seq1_m.25470	PREDICTED: dihydroflavonol-4-reductase-like [Daucus carota subsp. sativus]	0.379	Down	5.31E-08	38.694	20.965	19.6	9	16	9	1.433	1.386	1.417	0.53	0.54	0.534
ath01110 Biosynthesis of secondary metabolites	0.000275	cds.Camellia_sinensis.comp33776_c0_seq1_m.19252	PREDICTED: (-)-alpha-terpineol synthase [Vitis vinifera]	0.505	Down	2.15E-05	70.02	47.141	12.2	7	10	7	1.351	1.265	1.304	0.639	0.691	0.651
ath01110 Biosynthesis of secondary metabolites	0.000275	cds.Camellia_sinensis.comp24298_c0_seq1_m.23229	PREDICTED: 3-ketoacyl-CoA synthase 6 [Sesamum indicum]	0.213	Down	0.006598	56.181	4.7365	4.4	2	3	2	1.558	1.615	1.613	0.265	0.345	0.41
ath01110 Biosynthesis of secondary metabolites	0.000275	cds.Camellia_sinensis.comp32304_c0_seq3_m.11225	PREDICTED: phenylalanine ammonia-lyase [Vitis	0.417	Down	1.92E-07	77.077	118.08	37.8	25	54	6	1.375	1.378	1.392	0.561	0.579	0.587
ath01110 Biosynthesis of secondary metabolites	0.000275	cds.Camellia_sinensis.comp34400_c0_seq9_m.12194	"PREDICTED: granule-bound starch synthase 1, chloroplastic/amyloplastic isoform X2 [Theobroma	0.588	Down	4.87E-07	67.174	39.882	21.9	12	21	11	1.242	1.24	1.25	0.718	0.734	0.744
ath01110 Biosynthesis of secondary metabolites	0.000275	cds.Camellia_sinensis.comp23543_c0_seq1_m.33026	PREDICTED: cinnamoyl-CoA reductase 1 [Solanum tuberosum]	0.562	Down	4.89E-07	36.641	29.051	21.3	7	16	7	1.264	1.283	1.25	0.716	0.719	0.699
ath01110 Biosynthesis of secondary metabolites	0.000275	cds.Camellia_sinensis.comp36199_c0_seq1_m.25892	PREDICTED: very-long-chain 3-oxoacyl-CoA reductase 1 [Juglans regia]	0.604	Down	0.000562	35.595	5.1761	11.2	4	4	4	1.276	1.221	1.168	0.709	0.7	0.803
ath01110 Biosynthesis of secondary metabolites	0.000275	cds.Camellia_sinensis.comp27225_c0_seq2_m.28877	anthocyanidin reductase [Vitis vinifera]	0.525	Down	2.24E-06	37.459	8.2769	20.5	7	18	2	1.311	1.253	1.313	0.684	0.687	0.663
ath01110 Biosynthesis of secondary metabolites	0.000275	cds.Camellia_sinensis.comp27225_c0_seq1_m.28449	anthocyanidin reductase [Vitis vinifera]	0.424	Down	3.32E-06	36.273	62.198	30.3	11	25	6	1.404	1.342	1.365	0.559	0.608	0.576

ath01110 Biosynthesis of secondary metabolites	0.000275	cds.Camellia_sinensis.comp34132_c2_seq2_m.479	PREDICTED: acetyl-CoA carboxylase 1 [Theobroma cacao]	0.511	Down	5.66E-07	252.94	89.799	14.8	30	35	30	1.296	1.316	1.3	0.677	0.649	0.673
ath01110 Biosynthesis of secondary metabolites	0.000275	cds.Camellia_sinensis.comp34996_c0_seq1_m.19097	flavonoid 3' hydroxylase [Vitis vinifera]	0.538	Down	2.09E-06	57.059	17.251	19.1	9	14	9	1.248	1.29	1.269	0.668	0.678	0.703
ath01110 Biosynthesis of secondary metabolites	0.000275	cds.Camellia_sinensis.comp35217_c0_seq1_m.27870	PREDICTED: leucoanthocyanidin dioxygenase [Theobroma cacao]	0.416	Down	8.03E-07	40.057	51.661	24.5	7	9	7	1.343	1.36	1.416	0.567	0.584	0.561
ath01110 Biosynthesis of secondary metabolites	0.000275	cds.Camellia_sinensis.comp24956_c0_seq1_m.23294	PREDICTED: peroxidase 42 [Eucalyptus grandis]	0.609	Down	2.14E-05	37.589	27.808	24	6	6	6	1.202	1.251	1.235	0.749	0.773	0.725
ath01110 Biosynthesis of secondary metabolites	0.000275	cds.Camellia_sinensis.comp31922_c0_seq2_m.26835	PREDICTED: ATP-citrate synthase alpha chain protein 2 [Nicotiana attenuata]	0.554	Down	0.000581	48.023	1.21	10.3	5	9	2	1.348	1.238	1.215	0.647	0.688	0.769
ath01110 Biosynthesis of secondary metabolites	0.000275	cds.Camellia_sinensis.comp31295_c0_seq1_m.4827	PREDICTED: 4-coumarate--CoA ligase 2 [Theobroma cacao]	0.432	Down	0.00492	61.529	11.529	4.7	3	3	2	1.387	1.367	1.352	0.531	0.651	0.592
ath01110 Biosynthesis of secondary metabolites	0.000275	cds.Camellia_sinensis.comp27350_c0_seq1_m.24260	PREDICTED: chalcone synthase 1 [Juglans regia]	0.35	Down	2.17E-05	48.966	67.923	32.2	11	35	2	1.518	1.391	1.424	0.472	0.54	0.506
ath01110 Biosynthesis of secondary metabolites	0.000275	cds.Camellia_sinensis.comp24910_c0_seq1_m.13295	PREDICTED: probable chalcone--flavonone isomerase 3 [Prunus mume]	0.555	Down	0.000603	23.229	80.777	23.4	5	10	5	1.326	1.256	1.273	0.771	0.733	0.635
ath00941 Flavonoid biosynthesis	1.09E-11	cds.Camellia_sinensis.comp25454_c0_seq1_m.28314	"PREDICTED: naringenin,2-oxoglutarate 3-dioxygenase [Ziziphus jujuba]"	0.41	Down	5.66E-07	44.491	178.49	49.4	18	49	18	1.386	1.375	1.399	0.556	0.589	0.561
ath00941 Flavonoid biosynthesis	1.09E-11	cds.Camellia_sinensis.comp27225_c0_seq2_m.28877	anthocyanidin reductase [Vitis vinifera]	0.525	Down	2.24E-06	37.459	8.2769	20.5	7	18	2	1.311	1.253	1.313	0.684	0.687	0.663
ath00941 Flavonoid biosynthesis	1.09E-11	cds.Camellia_sinensis.comp28142_c0_seq1_m.18076	PREDICTED: trans-cinnamate 4-monooxygenase [Jatropha curcas]	0.578	Down	1.1E-06	60.624	61.458	26.1	13	20	13	1.245	1.257	1.245	0.711	0.741	0.714
ath00941 Flavonoid biosynthesis	1.09E-11	cds.Camellia_sinensis.comp27225_c0_seq1_m.28449	anthocyanidin reductase [Vitis vinifera]	0.424	Down	3.32E-06	36.273	62.198	30.3	11	25	6	1.404	1.342	1.365	0.559	0.608	0.576
ath00941 Flavonoid biosynthesis	1.09E-11	cds.Camellia_sinensis.comp34996_c0_seq1_m.19097	flavonoid 3' hydroxylase [Vitis vinifera]	0.538	Down	2.09E-06	57.059	17.251	19.1	9	14	9	1.248	1.29	1.269	0.668	0.678	0.703
ath00941 Flavonoid biosynthesis	1.09E-11	cds.Camellia_sinensis.comp35217_c0_seq1_m.27870	PREDICTED: leucoanthocyanidin dioxygenase [Theobroma cacao]	0.416	Down	8.03E-07	40.057	51.661	24.5	7	9	7	1.343	1.36	1.416	0.567	0.584	0.561
ath00941 Flavonoid biosynthesis	1.09E-11	cds.Camellia_sinensis.comp12781_c0_seq1_m.25470	PREDICTED: dihydroflavonol-4-reductase-like [Daucus carota subsp. sativus]	0.379	Down	5.31E-08	38.694	20.965	19.6	9	16	9	1.433	1.386	1.417	0.53	0.54	0.534
ath00941 Flavonoid biosynthesis	1.09E-11	cds.Camellia_sinensis.comp27350_c0_seq1_m.24260	PREDICTED: chalcone synthase 1 [Juglans regia]	0.35	Down	2.17E-05	48.966	67.923	32.2	11	35	2	1.518	1.391	1.424	0.472	0.54	0.506
ath00941 Flavonoid biosynthesis	1.09E-11	cds.Camellia_sinensis.comp24910_c0_seq1_m.13295	PREDICTED: probable chalcone--flavonone isomerase 3 [Prunus mume]	0.555	Down	0.000603	23.229	80.777	23.4	5	10	5	1.326	1.256	1.273	0.771	0.733	0.635

ath00940 Phenylpropanoid biosynthesis	0.000184	cds.Camellia_sinensis.comp29812_c0_seq1_m.18555	PREDICTED: 4-coumarate--CoA ligase 2-like [Jatropha curcas]	0.636	Down	0.000662	60.204	4.0311	7.8	5	5	2	1.142	1.254	1.239	0.822	0.723	0.767
ath00940 Phenylpropanoid biosynthesis	0.000184	cds.Camellia_sinensis.comp23543_c0_seq1_m.33026	PREDICTED: cinnamoyl-CoA reductase 1 [Solanum tuberosum]	0.562	Down	4.89E-07	36.641	29.051	21.3	7	16	7	1.264	1.283	1.25	0.716	0.719	0.699
ath00940 Phenylpropanoid biosynthesis	0.000184	cds.Camellia_sinensis.comp28142_c0_seq1_m.18076	PREDICTED: trans-cinnamate 4-monooxygenase [Jatropha curcas]	0.578	Down	1.1E-06	60.624	61.458	26.1	13	20	13	1.245	1.257	1.245	0.711	0.741	0.714
ath00940 Phenylpropanoid biosynthesis	0.000184	cds.Camellia_sinensis.comp24956_c0_seq1_m.23294	PREDICTED: peroxidase 42 [Eucalyptus grandis]	0.609	Down	2.14E-05	37.589	27.808	24	6	6	6	1.202	1.251	1.235	0.749	0.773	0.725
ath00940 Phenylpropanoid biosynthesis	0.000184	cds.Camellia_sinensis.comp31295_c0_seq1_m.4827	PREDICTED: 4-coumarate--CoA ligase 2 [Theobroma cacao]	0.432	Down	0.00492	61.529	11.529	4.7	3	3	2	1.387	1.367	1.352	0.531	0.651	0.592
ath00940 Phenylpropanoid biosynthesis	0.000184	cds.Camellia_sinensis.comp32304_c0_seq3_m.11225	PREDICTED: phenylalanine ammonia-lyase [Vitis	0.417	Down	1.92E-07	77.077	118.08	37.8	25	54	6	1.375	1.378	1.392	0.561	0.579	0.587
ath00360 Phenylalanine metabolism	0.000714	cds.Camellia_sinensis.comp29812_c0_seq1_m.18555	PREDICTED: 4-coumarate--CoA ligase 2-like [Jatropha curcas]	0.636	Down	0.000662	60.204	4.0311	7.8	5	5	2	1.142	1.254	1.239	0.822	0.723	0.767
ath00360 Phenylalanine metabolism	0.000714	cds.Camellia_sinensis.comp28142_c0_seq1_m.18076	PREDICTED: trans-cinnamate 4-monooxygenase [Jatropha curcas]	0.578	Down	1.1E-06	60.624	61.458	26.1	13	20	13	1.245	1.257	1.245	0.711	0.741	0.714
ath00360 Phenylalanine metabolism	0.000714	cds.Camellia_sinensis.comp31295_c0_seq1_m.4827	PREDICTED: 4-coumarate--CoA ligase 2 [Theobroma cacao]	0.432	Down	0.00492	61.529	11.529	4.7	3	3	2	1.387	1.367	1.352	0.531	0.651	0.592
ath00360 Phenylalanine metabolism	0.000714	cds.Camellia_sinensis.comp32304_c0_seq3_m.11225	PREDICTED: phenylalanine ammonia-lyase [Vitis	0.417	Down	1.92E-07	77.077	118.08	37.8	25	54	6	1.375	1.378	1.392	0.561	0.579	0.587
ath00130 Ubiquinone and other terpenoid-quinone biosynthesis	0.004942	cds.Camellia_sinensis.comp29812_c0_seq1_m.18555	PREDICTED: 4-coumarate--CoA ligase 2-like [Jatropha curcas]	0.636	Down	0.000662	60.204	4.0311	7.8	5	5	2	1.142	1.254	1.239	0.822	0.723	0.767
ath00130 Ubiquinone and other terpenoid-quinone biosynthesis	0.004942	cds.Camellia_sinensis.comp28142_c0_seq1_m.18076	PREDICTED: trans-cinnamate 4-monooxygenase [Jatropha curcas]	0.578	Down	1.1E-06	60.624	61.458	26.1	13	20	13	1.245	1.257	1.245	0.711	0.741	0.714
ath00130 Ubiquinone and other terpenoid-quinone biosynthesis	0.004942	cds.Camellia_sinensis.comp31295_c0_seq1_m.4827	PREDICTED: 4-coumarate--CoA ligase 2 [Theobroma cacao]	0.432	Down	0.00492	61.529	11.529	4.7	3	3	2	1.387	1.367	1.352	0.531	0.651	0.592
ath00062 Fatty acid elongation	0.011215	cds.Camellia_sinensis.comp36199_c0_seq1_m.25892	PREDICTED: very-long-chain 3-oxoacyl-CoA reductase 1 [Juglans regia]	0.604	Down	0.000562	35.595	5.1761	11.2	4	4	4	1.276	1.221	1.168	0.709	0.7	0.803
ath00062 Fatty acid elongation	0.011215	cds.Camellia_sinensis.comp24298_c0_seq1_m.23229	PREDICTED: 3-ketoacyl-CoA synthase 6 [Sesamum indicum]	0.213	Down	0.006598	56.181	4.7365	4.4	2	3	2	1.558	1.615	1.613	0.265	0.345	0.41
