# Supplementary material for: Drought stress triggers proteomic changes involving lignin, flavonoids and fatty acids in tea plants
Source: Sci Rep. 2020 Sep 23;10:15504. doi: 10.1038/s41598-020-72596-1 (PMC7511325; doi:10.1038/s41598-020-72596-1)
Supplement: Supplementary file 5 — Supplementary Table S5. [file 41598_2020_72596_MOESM5_ESM.pdf]

Drought Stress Triggers the Proteomic Changes Involved in Lignins, Flavonoids and Fatty Acids in Tea Plants

Honglian Gu[1], Yu Wang[1], Hui Xie[1], Chen Qiu[1], Shuning Zhang[1], Jun Xiao[2], Hongyan Li[3], Liang Chen[4], Xinghui Li[5], Zhaotang Ding[1,*]
1 Tea Research Institute, Qingdao Agricultural University, Qingdao, Shandong266109, China.
2 School of Biological Science and Winery Engineering, Taishan University, Taian, Shandong271000, China.
3 Haiyang fruit technology promotion station, Yantai, Shandong265100, China.
4 Tea Research Institute, Chinese Academy of Agricultural Sciences, Hangzhou, Zhejiang310008, China.
5 Tea Research Institute, Nanjing Agricultural University, Nanjing, Jiangsu210095, China.
guhongliantea@163.com (H.G.); wangyutea@163.com (Y.W.); xiehuitea@163.com (H.X.); qiuchentea@163.com (C.Q.); zhangshuningtea@163.com (S.Z.); xiaojun8299@163.com (J.X.); sunmijia0710@163.com (H.L.); liangchen@tricaas.com (L.C.); lxh@njau.edu.cn (X.L.).
*Correspondence: dzttea@163.com; Tel: +85-15853260396

Supplementary Table S1. Protein-protein interaction network

String name	Protein	Regulation	F/S Ratio	AverageShortestPathLength	BetweennessCentrality	Closeness Centrality	ClusteringCoefficient	Degree	Eccentricity	NeighborhoodConnectivity	NumberOfDirectedEdges	Radiality	Stress	TopologicalCoefficient	Subcellular localization	Biological Process	Cellular Component	Molecular Function	KEGG KO No.	KEGG Gene	KEGG pathway	IPR ID	Domain description
AT4G30800	cds.Camellia_sinensis.comp24911_c0_seq1_m.38588	Down	0.639	1	0.1833333	1	0.6666667	6	1	6	0	1	10	0.7222222	cytoplasm	GO:0006518 peptide metabolic process; GO:0043043 peptide biosynthetic process; GO:0044267 cellular protein metabolic process; GO:0006807 nitrogen compound metabolic process; GO:0009058 biosynthetic process; GO:0043603 cellular amide metabolic process; GO:0044238 primary metabolic process; GO:1901566 organonitrogen compound biosynthetic process; GO:1901576 organic substance biosynthetic process; GO:0044237 cellular metabolic process; GO:0044249 cellular biosynthetic process; GO:0071704 organic substance metabolic process; GO:0008152 metabolic process; GO:0044271 cellular nitrogen compound biosynthetic process; GO:0034645 cellular macromolecule biosynthetic process; GO:0044260 cellular macromolecule metabolic process; GO:0034641 cellular nitrogen compound metabolic process; GO:0010467 gene expression; GO:0043170 macromolecule metabolic process; GO:0043604 amide biosynthetic process; GO:0019538 protein metabolic process; GO:0006412 translation; GO:0009059 macromolecule biosynthetic process; GO:0009987 cellular process; GO:1901564 organonitrogen compound metabolic process;	GO:0005840 ribosome; GO:0043226 organelle; GO:0043229 intracellular organelle; GO:0043232 intracellular non-membrane-bounded organelle; GO:0044464 cell part; GO:0005622 intracellular; GO:0005737 cytoplasm; GO:0032991 macromolecular complex; GO:0044424 intracellular part; GO:1990904 ribonucleoprotein complex; GO:0005623 cell; GO:0030529 intracellular ribonucleoprotein complex; GO:0044444 cytoplasmic part; GO:0043228 non-membrane-bounded organelle;	GO:0003735 structural constituent of ribosome; GO:0005198 structural molecule activity;	K02949	RP-S11e; small subunit ribosomal protein S11e	ath03010 Ribosome - Arabidopsis thaliana (thale cress);	IPR012340; IPR032440;	Nucleic acid-binding, OB-fold; 40S ribosomal protein S11, N-terminal;
AT3G02560	cds.Camellia_sinensis.comp11716_c0_seq1_m.37295	Down	0.639	1.1666667	0.0166667	0.8571429	0.9	5	2	5	0	0.9166667	2	0.8333333	chloroplast	GO:0006518 peptide metabolic process; GO:0043043 peptide biosynthetic process; GO:0044267 cellular protein metabolic process; GO:0006807 nitrogen compound metabolic process; GO:0009058 biosynthetic process; GO:0043603 cellular amide metabolic process; GO:0044238 primary metabolic process; GO:1901566 organonitrogen compound biosynthetic process; GO:1901576 organic substance biosynthetic process; GO:0044237 cellular metabolic process; GO:0044249 cellular biosynthetic process; GO:0071704 organic substance metabolic process; GO:0008152 metabolic process; GO:0044271 cellular nitrogen compound biosynthetic process; GO:0034645 cellular macromolecule biosynthetic process; GO:0044260 cellular macromolecule metabolic process; GO:0034641 cellular nitrogen compound metabolic process; GO:0010467 gene expression; GO:0043170 macromolecule metabolic process; GO:0043604 amide biosynthetic process; GO:0019538 protein metabolic process; GO:0006412 translation; GO:0009059 macromolecule biosynthetic process; GO:0009987 cellular process; GO:1901564 organonitrogen compound metabolic process;	GO:0005840 ribosome; GO:0043226 organelle; GO:0043229 intracellular organelle; GO:0043232 intracellular non-membrane-bounded organelle; GO:0044464 cell part; GO:0005622 intracellular; GO:0005737 cytoplasm; GO:0032991 macromolecular complex; GO:0044424 intracellular part; GO:1990904 ribonucleoprotein complex; GO:0005623 cell; GO:0030529 intracellular ribonucleoprotein complex; GO:0044444 cytoplasmic part; GO:0043228 non-membrane-bounded organelle;	GO:0003735 structural constituent of ribosome; GO:0005198 structural molecule activity;	K02993	RP-S7e; small subunit ribosomal protein S7e	ath03010 Ribosome - Arabidopsis thaliana (thale cress);		
AT5G09510	cds.Camellia_sinensis.comp25442_c0_seq1_m.40798	Down	0.649	1.1666667	0.0166667	0.8571429	0.9	5	2	5	0	0.9166667	2	0.8333333	cytoplasm	GO:0006518 peptide metabolic process; GO:0043043 peptide biosynthetic process; GO:0044267 cellular protein metabolic process; GO:0006807 nitrogen compound metabolic process; GO:0009058 biosynthetic process; GO:0043603 cellular amide metabolic process; GO:0044238 primary metabolic process; GO:1901566 organonitrogen compound biosynthetic process; GO:1901576 organic substance biosynthetic process; GO:0044237 cellular metabolic process; GO:0044249 cellular biosynthetic process; GO:0071704 organic substance metabolic process; GO:0008152 metabolic process; GO:0044271 cellular nitrogen compound biosynthetic process; GO:0034645 cellular macromolecule biosynthetic process; GO:0044260 cellular macromolecule metabolic process; GO:0034641 cellular nitrogen compound metabolic process; GO:0010467 gene expression; GO:0043170 macromolecule metabolic process; GO:0043604 amide biosynthetic process; GO:0019538 protein metabolic process; GO:0006412 translation; GO:0009059 macromolecule biosynthetic process; GO:0009987 cellular process; GO:1901564 organonitrogen compound metabolic process;	GO:0005840 ribosome; GO:0043229 intracellular organelle; GO:0043232 intracellular non-membrane-bounded organelle; GO:0015935 small ribosomal subunit; GO:0044446 intracellular organelle part; GO:0005737 cytoplasm; GO:0032991 macromolecular complex; GO:0044424 intracellular part; GO:0005623 cell; GO:0044422 organelle part; GO:0043228 non-membrane-bounded organelle; GO:0043226 organelle; GO:0044391 ribosomal subunit; GO:0005622 intracellular; GO:0044464 cell part; GO:1990904 ribonucleoprotein complex; GO:0030529 intracellular ribonucleoprotein complex; GO:0044444 cytoplasmic part;	GO:0097159 organic cyclic compound binding; GO:1901363 heterocyclic compound binding; GO:0003723 RNA binding; GO:0005488 binding; GO:0003676 nucleic acid binding; GO:0005198 structural molecule activity; GO:0003735 structural constituent of ribosome;	K02958	RP-S15e; small subunit ribosomal protein S15e	ath03010 Ribosome - Arabidopsis thaliana (thale cress);	IPR023575;	Ribosomal protein S19, superfamily;
FLS1	cds.Camellia_sinensis.comp25037_c0_seq1_m.30194	Down	0.44	1.6923077	0.0308303	0.5909091	0.8	6	3	6	0	0.8615385	26	0.5757576	cytoplasm	GO:0044699 single-organism process; GO:0008152 metabolic process; GO:0055114 oxidation-reduction process; GO:0044710 single-organism metabolic process;		GO:0003824 catalytic activity; GO:0016491 oxidoreductase activity;				IPR005123; IPR027443; IPR026992;	Oxoglutarate/iron-dependent dioxygenase; Isopenicillin N synthase-like; Non-haem dioxygenase N-terminal domain;
F3H	cds.Camellia_sinensis.comp25454_c0_seq1_m.28314	Down	0.41	1.5384615	0.1718559	0.65	0.6666667	7	3	7	0	0.8923077	78	0.4880952	cytoplasm	GO:0044699 single-organism process; GO:0008152 metabolic process; GO:0055114 oxidation-reduction process; GO:0044710 single-organism metabolic process;		GO:0003824 catalytic activity; GO:0016491 oxidoreductase activity;	K00475	E1.14.11.9; naringenin 3-dioxygenase [EC:1.14.11.9]	ath01100 Metabolic pathways - Arabidopsis thaliana (thale cress); ath00941 Flavonoid biosynthesis - Arabidopsis thaliana (thale cress); ath01110 Biosynthesis of secondary metabolites - Arabidopsis thaliana (thale cress);	IPR027443; IPR005123; IPR026992;	Isopenicillin N synthase-like; Oxoglutarate/iron-dependent dioxygenase; Non-haem dioxygenase N-terminal domain;
CHIL	cds.Camellia_sinensis.comp24910_c0_seq1_m.13295	Down	0.555	1.6923077	0.0308303	0.5909091	0.8	6	3	6	0	0.8615385	26	0.5757576	cytoskeleton	GO:0008152 metabolic process; GO:0009058 biosynthetic process; GO:0009813 flavonoid biosynthetic process; GO:0009812 flavonoid metabolic process; GO:1901576 organic substance biosynthetic process; GO:0071704 organic substance metabolic process;		GO:0016872 intramolecular lyase activity; GO:0045430 chalcone isomerase activity; GO:0016853 isomerase activity; GO:0003824 catalytic activity;	K01859	E5.5.1.6; chalcone isomerase [EC:5.5.1.6]	ath01100 Metabolic pathways - Arabidopsis thaliana (thale cress); ath00941 Flavonoid biosynthesis - Arabidopsis thaliana (thale cress); ath01110 Biosynthesis of secondary metabolites - Arabidopsis thaliana (thale cress);	IPR016088; IPR016087;	Chalcone isomerase, 3-layer sandwich; Chalcone isomerase;
TT4	cds.Camellia_sinensis.comp27350_c0_seq1_m.24260	Down	0.35	1.4615385	0.3191392	0.6842105	0.4285714	8	3	8	0	0.9076923	134	0.4479167	chloroplast	GO:0009058 biosynthetic process; GO:0008152 metabolic process;		GO:0016746 transferase activity, transferring acyl groups; GO:0003824 catalytic activity; GO:0016740 transferase activity;	K00660	CHS; chalcone synthase [EC:2.3.1.74]	ath04712 Circadian rhythm - plant - Arabidopsis thaliana (thale cress); ath01100 Metabolic pathways - Arabidopsis thaliana (thale cress); ath00941 Flavonoid biosynthesis - Arabidopsis thaliana (thale cress); ath01110 Biosynthesis of secondary metabolites - Arabidopsis thaliana (thale cress);	IPR001099; IPR016039; IPR012328;	Chalcone/stilbene synthase, N-terminal; Thiolase-like; Chalcone/stilbene synthase, C-terminal;
AT1G57860.1	cds.Camellia_sinensis.comp28106_c0_seq1_m.46951	Down	0.659	1.1666667	0.0166667	0.8571429	0.9	5	2	5	0	0.9166667	2	0.8333333	cytoplasm	GO:0006518 peptide metabolic process; GO:0043043 peptide biosynthetic process; GO:0044267 cellular protein metabolic process; GO:0006807 nitrogen compound metabolic process; GO:0009058 biosynthetic process; GO:0043603 cellular amide metabolic process; GO:0044238 primary metabolic process; GO:1901566 organonitrogen compound biosynthetic process; GO:1901576 organic substance biosynthetic process; GO:0044237 cellular metabolic process; GO:0044249 cellular biosynthetic process; GO:0071704 organic substance metabolic process; GO:0008152 metabolic process; GO:0044271 cellular nitrogen compound biosynthetic process; GO:0034645 cellular macromolecule biosynthetic process; GO:0044260 cellular macromolecule metabolic process; GO:0034641 cellular nitrogen compound metabolic process; GO:0010467 gene expression; GO:0043170 macromolecule metabolic process; GO:0043604 amide biosynthetic process; GO:0019538 protein metabolic process; GO:0006412 translation; GO:0009059 macromolecule biosynthetic process; GO:0009987 cellular process; GO:1901564 organonitrogen compound metabolic process;	GO:0005840 ribosome; GO:0043226 organelle; GO:0043229 intracellular organelle; GO:0043232 intracellular non-membrane-bounded organelle; GO:0044464 cell part; GO:0005622 intracellular; GO:0005737 cytoplasm; GO:0032991 macromolecular complex; GO:0044424 intracellular part; GO:1990904 ribonucleoprotein complex; GO:0005623 cell; GO:0030529 intracellular ribonucleoprotein complex; GO:0044444 cytoplasmic part; GO:0043228 non-membrane-bounded organelle;	GO:0003735 structural constituent of ribosome; GO:0005198 structural molecule activity;	K02889	RP-L21e; large subunit ribosomal protein L21e	ath03010 Ribosome - Arabidopsis thaliana (thale cress);	IPR008991;	Translation protein SH3-like domain;
PAL1	cds.Camellia_sinensis.comp26557_c0_seq1_m.48630	Down	0.382	2.0769231	0.0273199	0.4814815	0.6666667	4	4	4	0	0.7846154	20	0.5277778	cytoplasm			GO:0003824 catalytic activity;				IPR024083; IPR008948;	Fumarase/histidase, N-terminal; L-Aspartase-like;

C4H	cds.Camellia_sinensis.comp28142_c0_seq1_m.18076	Down	0.578	1.8461539	0.1068376	0.5416667	0.4666667	6	4	6	0	0.8307692	58	0.45	cytoplasm	GO:0044699 single-organism process; GO:0008152 metabolic process; GO:0055114 oxidation-reduction process; GO:0044710 single-organism metabolic process;		GO:0097159 organic cyclic compound binding; GO:1901363 heterocyclic compound binding; GO:0020037 heme binding; GO:0005506 iron ion binding; GO:0046914 transition metal ion binding; GO:0003824 catalytic activity; GO:0005488 binding; GO:0043167 ion binding; GO:0043169 cation binding; GO:0046872 metal ion binding; GO:0016705 oxidoreductase activity, acting on paired donors, with incorporation or reduction of molecular oxygen; GO:0046906 tetrapyrrole binding; GO:0016491 oxidoreductase activity;	K00487	CYP73A; trans-cinnamate 4-monooxygenase [EC:1.14.13.11]	ath00945 Stilbenoid, diarylheptanoid and gingerol biosynthesis - Arabidopsis thaliana (thale cress); ath00130 Ubiquinone and other terpenoid-quinone biosynthesis - Arabidopsis thaliana (thale cress); ath01100 Metabolic pathways - Arabidopsis thaliana (thale cress); ath00941 Flavonoid biosynthesis - Arabidopsis thaliana (thale cress); ath00360 Phenylalanine metabolism - Arabidopsis thaliana (thale cress); ath00940 Phenylpropanoid biosynthesis - Arabidopsis thaliana (thale cress); ath01110 Biosynthesis of secondary metabolites - Arabidopsis thaliana (thale cress);		
DFR	cds.Camellia_sinensis.comp12781_c0_seq1_m.25470	Down	0.379	1.6923077	0.085928	0.5909091	0.7333333	6	3	6	0	0.8615385	44	0.5454546	chloroplast			GO:0048037 cofactor binding; GO:0003824 catalytic activity; GO:0005488 binding; GO:0050662 coenzyme binding;	K13082	DFR; bifunctional dihydroflavonol 4-reductase/flavanone 4-reductase [EC:1.1.1.219 1.1.1.234]	ath01100 Metabolic pathways - Arabidopsis thaliana (thale cress); ath00941 Flavonoid biosynthesis - Arabidopsis thaliana (thale cress); ath01110 Biosynthesis of secondary metabolites - Arabidopsis thaliana (thale cress);	IPR001509; IPR016040;	NAD-dependent epimerase/dehydratase; NAD(P)-binding domain;
Hsp70-2	cds.Camellia_sinensis.comp18938_c0_seq1_m.40311	Down	0.511	1	0	1	0	1	1	1	0	1	0	0	cytoplasm							IPR029048; IPR029047;	Heat shock protein 70kD, C-terminal domain; Heat shock protein 70kD, peptide-binding domain;
J3	cds.Camellia_sinensis.comp35775_c0_seq1_m.39035	Down	0.656	1	0	1	0	1	1	1	0	1	0	0	nucleus	GO:0006457 protein folding; GO:0009987 cellular process;		GO:0005515 protein binding; GO:0031072 heat shock protein binding; GO:0051082 unfolded protein binding; GO:0005488 binding;				IPR008971; IPR002939; IPR001305;	HSP40/DnaJ peptide-binding; Chaperone DnaJ, C-terminal; Heat shock protein DnaJ, cysteine-rich domain;
LDOX	cds.Camellia_sinensis.comp35217_c0_seq1_m.27870	Down	0.416	2.1538462	0.1538462	0.4642857	0.5	4	4	4	0	0.7692308	52	0.6428571	cytoplasm	GO:0044699 single-organism process; GO:0008152 metabolic process; GO:0055114 oxidation-reduction process; GO:0044710 single-organism metabolic process;		GO:0003824 catalytic activity; GO:0016491 oxidoreductase activity;	K05277	ANS; leucoanthocyanidin dioxygenase [EC:1.14.11.19]	ath01100 Metabolic pathways - Arabidopsis thaliana (thale cress); ath00941 Flavonoid biosynthesis - Arabidopsis thaliana (thale cress); ath01110 Biosynthesis of secondary metabolites - Arabidopsis thaliana (thale cress);	IPR027443; IPR005123; IPR026992;	Isopenicillin N synthase-like; Oxoglutarate/iron-dependent dioxygenase; Non-haem dioxygenase N-terminal domain;
TT7	cds.Camellia_sinensis.comp34996_c0_seq1_m.19097	Down	0.538	2.0769231	0.017094	0.4814815	0.8	5	4	5	0	0.7846154	8	0.725	chloroplast	GO:0044699 single-organism process; GO:0008152 metabolic process; GO:0055114 oxidation-reduction process; GO:0044710 single-organism metabolic process;		GO:0097159 organic cyclic compound binding; GO:1901363 heterocyclic compound binding; GO:0020037 heme binding; GO:0005506 iron ion binding; GO:0046914 transition metal ion binding; GO:0003824 catalytic activity; GO:0005488 binding; GO:0043167 ion binding; GO:0043169 cation binding; GO:0046872 metal ion binding; GO:0016705 oxidoreductase activity, acting on paired donors, with incorporation or reduction of molecular oxygen; GO:0046906 tetrapyrrole binding; GO:0016491 oxidoreductase activity;	K05280	E1.14.13.21; flavonoid 3'-monooxygenase [EC:1.14.13.21]	ath01100 Metabolic pathways - Arabidopsis thaliana (thale cress); ath00941 Flavonoid biosynthesis - Arabidopsis thaliana (thale cress); ath00944 Flavone and flavonol biosynthesis - Arabidopsis thaliana (thale cress); ath01110 Biosynthesis of secondary metabolites - Arabidopsis thaliana (thale cress);		
4CL3	cds.Camellia_sinensis.comp31295_c0_seq1_m.4827	Down	0.432	1.6153846	0.1562882	0.6190476	0.5333333	6	3	6	0	0.8769231	74	0.5138889	cytoplasm	GO:0008152 metabolic process;		GO:0003824 catalytic activity;	K01904	4CL; 4-coumarate--CoA ligase [EC:6.2.1.12]	ath00130 Ubiquinone and other terpenoid-quinone biosynthesis - Arabidopsis thaliana (thale cress); ath01100 Metabolic pathways - Arabidopsis thaliana (thale cress); ath00360 Phenylalanine metabolism - Arabidopsis thaliana (thale cress); ath00940 Phenylpropanoid biosynthesis - Arabidopsis thaliana (thale cress); ath01110 Biosynthesis of secondary metabolites - Arabidopsis thaliana (thale cress);	IPR025110; IPR000873;	AMP-binding enzyme C-terminal domain; AMP-dependent synthetase/ligase;
PAL2	cds.Camellia_sinensis.comp38050_c0_seq1_m.42138	Down	0.592	2.6153846	0	0.3823529	1	3	5	3	0	0.6769231	0	0.7777778	nucleus			GO:0003824 catalytic activity;				IPR008948; IPR023144;	L-Aspartase-like; Phenylalanine ammonia-lyase, shielding domain;
4CL2	cds.Camellia_sinensis.comp29812_c0_seq1_m.18555	Down	0.636	2	0.0410562	0.5	0.6666667	4	4	4	0	0.8	30	0.525	plasma membrane	GO:0008152 metabolic process;		GO:0003824 catalytic activity;	K01904	4CL; 4-coumarate--CoA ligase [EC:6.2.1.12]	ath00130 Ubiquinone and other terpenoid-quinone biosynthesis - Arabidopsis thaliana (thale cress); ath01100 Metabolic pathways - Arabidopsis thaliana (thale cress); ath00360 Phenylalanine metabolism - Arabidopsis thaliana (thale cress); ath00940 Phenylpropanoid biosynthesis - Arabidopsis thaliana (thale cress); ath01110 Biosynthesis of secondary metabolites - Arabidopsis thaliana (thale cress);	IPR025110; IPR000873;	AMP-binding enzyme C-terminal domain; AMP-dependent synthetase/ligase;
RPL15	cds.Camellia_sinensis.comp34663_c0_seq10_m.34729	Down	0.649	1.1666667	0.1	0.8571429	0.7	5	2	5	0	0.9166667	6	0.7666667	chloroplast	GO:0006518 peptide metabolic process; GO:0043043 peptide biosynthetic process; GO:0044267 cellular protein metabolic process; GO:0006807 nitrogen compound metabolic process; GO:0009058 biosynthetic process; GO:0043603 cellular amide metabolic process; GO:0044238 primary metabolic process; GO:1901566 organonitrogen compound biosynthetic process; GO:1901576 organic substance biosynthetic process; GO:0044237 cellular metabolic process; GO:0044249 cellular biosynthetic process; GO:0071704 organic substance metabolic process; GO:0008152 metabolic process; GO:0044271 cellular nitrogen compound biosynthetic process; GO:0034645 cellular macromolecule biosynthetic process; GO:0044260 cellular macromolecule metabolic process; GO:0034641 cellular nitrogen compound metabolic process; GO:0010467 gene expression; GO:0043170 macromolecule metabolic process; GO:0043604 amide biosynthetic process; GO:0019538 protein metabolic process; GO:0006412 translation; GO:0009059 macromolecule biosynthetic process; GO:0009987 cellular process; GO:1901564 organonitrogen compound metabolic process;	GO:0005840 ribosome; GO:0043229 intracellular organelle; GO:0043232 intracellular non-membrane-bounded organelle; GO:0044446 intracellular organelle part; GO:0005737 cytoplasm; GO:0032991 macromolecular complex; GO:0044424 intracellular part; GO:0005623 cell; GO:0044422 organelle part; GO:0043228 non-membrane-bounded organelle; GO:0043226 organelle; GO:0015934 large ribosomal subunit; GO:0044391 ribosomal subunit; GO:0005622 intracellular; GO:0044464 cell part; GO:1990904 ribonucleoprotein complex; GO:0030529 intracellular ribonucleoprotein complex; GO:0044444 cytoplasmic part;	GO:0003735 structural constituent of ribosome; GO:0005198 structural molecule activity;	K02876	RP-L15; large subunit ribosomal protein L15	ath03010 Ribosome - Arabidopsis thaliana (thale cress);	IPR021131;	Ribosomal protein L18e/L15P;
IRX4	cds.Camellia_sinensis.comp23543_c0_seq1_m.33026	Down	0.562	2.1538462	0	0.4642857	1	2	4	2	0	0.7692308	0	0.7	chloroplast			GO:0048037 cofactor binding; GO:0003824 catalytic activity; GO:0005488 binding; GO:0050662 coenzyme binding;	K09753	CCR; cinnamoyl-CoA reductase [EC:1.2.1.44]	ath01100 Metabolic pathways - Arabidopsis thaliana (thale cress); ath00940 Phenylpropanoid biosynthesis - Arabidopsis thaliana (thale cress); ath01110 Biosynthesis of secondary metabolites - Arabidopsis thaliana (thale cress);	IPR001509; IPR016040;	NAD-dependent epimerase/dehydratase; NAD(P)-binding domain;
BAN	cds.Camellia_sinensis.comp27225_c0_seq2_m.28877	Down	0.525	3.0769231	0	0.325	0	1	5	1	0	0.5846154	0	0	chloroplast			GO:0048037 cofactor binding; GO:0003824 catalytic activity; GO:0005488 binding; GO:0050662 coenzyme binding;	K08695	ANR; anthocyanidin reductase [EC:1.3.1.77]	ath00941 Flavonoid biosynthesis - Arabidopsis thaliana (thale cress); ath01110 Biosynthesis of secondary metabolites - Arabidopsis thaliana (thale cress);	IPR001509; IPR016040;	NAD-dependent epimerase/dehydratase; NAD(P)-binding domain;
AT5G24490	cds.Camellia_sinensis.comp24163_c0_seq1_m.42497	Down	0.654	1.6666667	0	0.6	1	2	2	2	0	0.6666667	0	0.9166667	chloroplast	GO:0008152 metabolic process; GO:0044238 primary metabolic process;							

ACLA-2	cds.Camellia_sinensis.comp31922_c0_seq2_m.26835	Down	0.554	1	0	1	0	1	1	1	0	1	0	0	chloroplast		GO:0005524 ATP binding; GO:1901265 nucleoside phosphate binding; GO:0001882 nucleoside binding; GO:0043167 ion binding; GO:0000166 nucleotide binding; GO:0097159 organic cyclic compound binding; GO:1901363 heterocyclic compound binding; GO:0032549 ribonucleoside binding; GO:0097367 carbohydrate derivative binding; GO:0032553 ribonucleotide binding; GO:0005488 binding; GO:0032550 purine ribonucleoside binding; GO:0043168 anion binding; GO:0032555 purine ribonucleotide binding; GO:0032559 adenyl ribonucleotide binding; GO:0030554 adenyl nucleotide binding; GO:0001883 purine nucleoside binding; GO:0017076 purine nucleotide binding; GO:0036094 small molecule binding; GO:0035639 purine ribonucleoside triphosphate binding;	K01648	ACLY; ATP citrate (pro-S)-lyase [EC:2.3.3.8]	ath01100 Metabolic pathways - Arabidopsis thaliana (thale cress); ath00020 Citrate cycle (TCA cycle) - Arabidopsis thaliana (thale cress); ath01110 Biosynthesis of secondary metabolites - Arabidopsis thaliana (thale cress);	IPR032263; IPR013816; IPR013650; IPR016102;	ATP-citrate synthase, citrate-binding domain; ATP-grasp fold, subdomain 2; ATP-grasp fold, succinyl-CoA synthetase-type; Succinyl-CoA synthetase-like;
ACC1	cds.Camellia_sinensis.comp34132_c2_seq2_m.479	Down	0.511	1	0	1	0	1	1	1	0	1	0	0	chloroplast	GO:0044711 single-organism biosynthetic process; GO:0016053 organic acid biosynthetic process; GO:0044281 small molecule metabolic process; GO:0044763 single-organism cellular process; GO:0044699 single-organism process; GO:0009058 biosynthetic process; GO:0044255 cellular lipid metabolic process; GO:0006631 fatty acid metabolic process; GO:0044238 primary metabolic process; GO:0044237 cellular metabolic process; GO:0006633 fatty acid biosynthetic process; GO:1901576 organic substance biosynthetic process; GO:0044249 cellular biosynthetic process; GO:0071704 organic substance metabolic process; GO:0008610 lipid biosynthetic process; GO:0046394 carboxylic acid biosynthetic process; GO:0008152 metabolic process; GO:0044710 single-organism metabolic process; GO:0019752 carboxylic acid metabolic process; GO:0044283 small molecule biosynthetic process; GO:0032787 monocarboxylic acid metabolic process; GO:0006082 organic acid metabolic process; GO:0009987 cellular process; GO:0072330 monocarboxylic acid biosynthetic process; GO:0043436 oxoacid metabolic process; GO:0006629 lipid metabolic process;	GO:0005524 ATP binding; GO:1901265 nucleoside phosphate binding; GO:0016885 ligase activity, forming carbon-carbon bonds; GO:0001882 nucleoside binding; GO:0043167 ion binding; GO:0016874 ligase activity; GO:0000166 nucleotide binding; GO:1901363 heterocyclic compound binding; GO:0003989 acetyl-CoA carboxylase activity; GO:0016879 ligase activity, forming carbon-nitrogen bonds; GO:0097159 organic cyclic compound binding; GO:0032549 ribonucleoside binding; GO:0004075 biotin carboxylase activity; GO:0097367 carbohydrate derivative binding; GO:0016421 CoA carboxylase activity; GO:0032553 ribonucleotide binding; GO:0003824 catalytic activity; GO:0005488 binding; GO:0032550 purine ribonucleoside binding; GO:0046872 metal ion binding; GO:0043169 cation binding; GO:0032555 purine ribonucleotide binding; GO:0043168 anion binding; GO:0032559 adenyl ribonucleotide binding; GO:0030554 adenyl nucleotide binding; GO:0001883 purine nucleoside binding; GO:0017076 purine nucleotide binding; GO:0036094 small molecule binding; GO:0035639 purine ribonucleoside triphosphate binding;	K11262	ACACA; acetyl-CoA carboxylase / biotin carboxylase 1 [EC:6.4.1.2 6.3.4.14]	ath00061 Fatty acid biosynthesis - Arabidopsis thaliana (thale cress); ath01212 Fatty acid metabolism - Arabidopsis thaliana (thale cress); ath00640 Propanoate metabolism - Arabidopsis thaliana (thale cress); ath00620 Pyruvate metabolism - Arabidopsis thaliana (thale cress); ath01100 Metabolic pathways - Arabidopsis thaliana (thale cress); ath01110 Biosynthesis of secondary metabolites - Arabidopsis thaliana (thale cress);	IPR011762; IPR000022; IPR029045; IPR011053; IPR005481; IPR000089; IPR005479; IPR013815; IPR013537; IPR016185; IPR011764; IPR011761; IPR005482; IPR013816; IPR011763; IPR011054	Acetyl-coenzyme A carboxyltransferase, N-terminal; Carboxyl transferase; ClpP/crotonase-like domain; Single hybrid motif; Biotin carboxylase-like, N-terminal domain; Biotin/lipoyl attachment; Carbamoyl-phosphate synthetase large subunit-
DHS1	cds.Camellia_sinensis.comp30057_c1_seq2_m.50785	Down	0.492	1	0	1	0	1	1	1	0	1	0	0	chloroplast	GO:0008652 cellular amino acid biosynthetic process; GO:0009072 aromatic amino acid family metabolic process; GO:0016053 organic acid biosynthetic process; GO:0044711 single-organism biosynthetic process; GO:0006725 cellular aromatic compound metabolic process; GO:0044281 small molecule metabolic process; GO:0044763 single-organism cellular process; GO:0044699 single-organism process; GO:0006807 nitrogen compound metabolic process; GO:1901360 organic cyclic compound metabolic process; GO:0009058 biosynthetic process; GO:0009073 aromatic amino acid family biosynthetic process; GO:0044238 primary metabolic process; GO:1901566 organonitrogen compound biosynthetic process; GO:0044237 cellular metabolic process; GO:1901576 organic substance biosynthetic process; GO:0044249 cellular biosynthetic process; GO:0071704 organic substance metabolic process; GO:0008152 metabolic process; GO:0046394 carboxylic acid biosynthetic process; GO:0006520 cellular amino acid metabolic process; GO:0019438 aromatic compound biosynthetic process; GO:0044710 single-organism metabolic process; GO:0019752 carboxylic acid metabolic process; GO:0044283 small molecule biosynthetic process; GO:0006082 organic acid metabolic process; GO:0009987 cellular process; GO:0043436 oxoacid metabolic process; GO:1901362 organic cyclic compound biosynthetic process; GO:1901564 organonitrogen compound metabolic process;	GO:0016765 transferase activity, transferring alkyl or aryl (other than methyl) groups; GO:0003849 3-deoxy-7-phosphoheptulonate synthase activity; GO:0003824 catalytic activity; GO:0016740 transferase activity;					
AT1G22410	cds.Camellia_sinensis.comp30057_c0_seq1_m.29301	Down	0.521	1	0	1	0	1	1	1	0	1	0	0	cytoplasm	GO:0008652 cellular amino acid biosynthetic process; GO:0009072 aromatic amino acid family metabolic process; GO:0016053 organic acid biosynthetic process; GO:0044711 single-organism biosynthetic process; GO:0006725 cellular aromatic compound metabolic process; GO:0044281 small molecule metabolic process; GO:0044763 single-organism cellular process; GO:0044699 single-organism process; GO:0006807 nitrogen compound metabolic process; GO:1901360 organic cyclic compound metabolic process; GO:0009058 biosynthetic process; GO:0009073 aromatic amino acid family biosynthetic process; GO:0044238 primary metabolic process; GO:1901566 organonitrogen compound biosynthetic process; GO:0044237 cellular metabolic process; GO:1901576 organic substance biosynthetic process; GO:0044249 cellular biosynthetic process; GO:0071704 organic substance metabolic process; GO:0008152 metabolic process; GO:0046394 carboxylic acid biosynthetic process; GO:0006520 cellular amino acid metabolic process; GO:0019438 aromatic compound biosynthetic process; GO:0044710 single-organism metabolic process; GO:0019752 carboxylic acid metabolic process; GO:0044283 small molecule biosynthetic process; GO:0006082 organic acid metabolic process; GO:0009987 cellular process; GO:0043436 oxoacid metabolic process; GO:1901362 organic cyclic compound biosynthetic process; GO:1901564 organonitrogen compound metabolic process;	GO:0016765 transferase activity, transferring alkyl or aryl (other than methyl) groups; GO:0003849 3-deoxy-7-phosphoheptulonate synthase activity; GO:0003824 catalytic activity; GO:0016740 transferase activity;					
AT4G16830	cds.Camellia_sinensis.comp31831_c0_seq3_m.20572	Down	0.573	1.3333333	0	0.75	1	4	2	4	0	0.8333333	0	0.875	nucleus						IPR019084; IPR006861;	Stm1-like, N-terminal; Hyaluronan/mRNA-binding
ALY4	cds.Camellia_sinensis.comp26983_c0_seq9_m.28144	Down	0.656	1	0	1	0	1	1	1	0	1	0	0	nucleus		GO:0097159 organic cyclic compound binding; GO:1901363 heterocyclic compound binding; GO:1901265 nucleoside phosphate binding; GO:0005488 binding; GO:0003676 nucleic acid binding; GO:0000166 nucleotide binding; GO:0036094 small molecule binding;	K12881	THOC4; THO complex subunit 4	ath03015 mRNA surveillance pathway - Arabidopsis thaliana (thale cress); ath03040 Spliceosome - Arabidopsis thaliana (thale cress); ath03013 RNA transport - Arabidopsis thaliana (thale cress);	IPR000504; IPR012677;	RNA recognition motif domain; Nucleotide-binding alpha-beta plait domain;
AT3G12640	cds.Camellia_sinensis.comp32371_c0_seq1_m.8400	Down	0.591	1	0	1	0	1	1	1	0	1	0	0	nucleus	GO:0006396 RNA processing; GO:0006725 cellular aromatic compound metabolic process; GO:1901360 organic cyclic compound metabolic process; GO:0006807 nitrogen compound metabolic process; GO:0044238 primary metabolic process; GO:0044237 cellular metabolic process; GO:0071704 organic substance metabolic process; GO:0006397 mRNA processing; GO:0016070 RNA metabolic process; GO:0008152 metabolic process; GO:0090304 nucleic acid metabolic process; GO:0046483 heterocycle metabolic process; GO:0006139 nucleobase-containing compound metabolic process; GO:0044260 cellular macromolecule metabolic process; GO:0010467 gene expression; GO:0034641 cellular nitrogen compound metabolic process; GO:0043170 macromolecule metabolic process; GO:0009987 cellular process; GO:0016071 mRNA metabolic process;	GO:0097159 organic cyclic compound binding; GO:1901363 heterocyclic compound binding; GO:1901265 nucleoside phosphate binding; GO:0005488 binding; GO:0003676 nucleic acid binding; GO:0000166 nucleotide binding; GO:0036094 small molecule binding;				IPR000504; IPR012677; IPR002483;	RNA recognition motif domain; Nucleotide-binding alpha-beta plait domain; PWI domain;
