# Supplementary material for: Drought stress triggers proteomic changes involving lignin, flavonoids and fatty acids in tea plants
Source: Sci Rep. 2020 Sep 23;10:15504. doi: 10.1038/s41598-020-72596-1 (PMC7511325; doi:10.1038/s41598-020-72596-1)
Supplement: Supplementary file 1 — Supplementary Table S1. [file 41598_2020_72596_MOESM1_ESM.pdf]

Drought Stress Triggers the Proteomic Changes Involved in Lignins, Flavonoids and Fatty Acids in Tea Plants

Honglian Gu[1], Yu Wang[1], Hui Xie[1], Chen Qiu[1], Shuning Zhang[1], Jun Xiao[2], Hongyan Li[3], Liang Chen[4], Xinghui Li[5], Zhaotang Ding[1,*]
1 Tea Research Institute, Qingdao Agricultural University, Qingdao, Shandong266109, China.
2 School of Biological Science and Winery Engineering, Taishan University, Taian, Shandong271000, China.
3 Haiyang fruit technology promotion station, Yantai, Shandong265100, China.
4 Tea Research Institute, Chinese Academy of Agricultural Sciences, Hangzhou, Zhejiang310008, China.
5 Tea Research Institute, Nanjing Agricultural University, Nanjing, Jiangsu210095, China.
guhongliantea@163.com (H.G.); wangyutea@163.com (Y.W.); xiehuitea@163.com (H.X.); qiuchentea@163.com (C.Q.); zhangshuningtea@163.com (S.Z.); xiaojun8299@163.com (J.X.); sunmijia0710@163.com (H.L.); liangchen@tricaas.com (L.C.); lxh@njau.edu.cn (X.L.).
*Correspondence: dzttea@163.com; Tel: +85-15853260396

Supplementary Table S1. MS/MS spetrum database search analysis summary

Total spectrums	Matched spetrums	Peptides	Unique peptides	Identified proteins	Quantifiable proteins
244124	51213	25805	23854	4789	4242

Table 2. Differentially expressed protein summary (Filtered with threshold value of expression fold change and P vlaue < 0.05)

Compare group	Regulated type	fold change >1.5
DT/CK	up-regulated	11
	down-regulated	100
